# Systematic Reviews and Meta-analyses of the Procedure-specific Risks of Thrombosis and Bleeding in General Abdominal, Colorectal, Upper Gastrointestinal, and Hepatopancreatobiliary Surgery

**DOI:** 10.1097/SLA.0000000000006059

**Published:** 2023-08-08

**Authors:** Lauri I. Lavikainen, Gordon H. Guyatt, Ville J. Sallinen, Paul J. Karanicolas, Rachel J. Couban, Tino Singh, Yung Lee, Jaana Elberkennou, Riikka Aaltonen, Kaisa Ahopelto, Ines Beilmann-Lehtonen, Marco H. Blanker, Jovita L. Cárdenas, Rufus Cartwright, Samantha Craigie, P.J. Devereaux, Herney A. Garcia-Perdomo, Fang Zhou Ge, Huda A. Gomaa, Alex L.E. Halme, Jari Haukka, Päivi K. Karjalainen, Tuomas P. Kilpeläinen, Antti J. Kivelä, Hanna Lampela, Anne K. Mattila, Borna Tadayon Najafabadi, Taina P. Nykänen, Sanjay Pandanaboyana, Negar Pourjamal, Chathura B.B. Ratnayake, Aleksi Raudasoja, Robin W.M. Vernooij, Philippe D. Violette, Yuting Wang, Yingqi Xiao, Liang Yao, Kari A. O. Tikkinen

**Affiliations:** * Faculty of Medicine, University of Helsinki, Helsinki, Finland; † Department of Health Research Methods, Evidence, and Impact, McMaster University, Hamilton, ON, Canada; ‡ Department of Medicine, McMaster University, Hamilton, ON, Canada; § Department of Transplantation and Liver Surgery, University of Helsinki and Helsinki University Hospital, Helsinki, Finland; ∥ Department of Gastroenterological Surgery, University of Helsinki and Helsinki University Hospital, Helsinki, Finland; ¶ Department of Surgery, Sunnybrook Health Sciences Centre, Toronto, ON, Canada; # Department of Surgery, University of Toronto, Toronto, ON, Canada; ** Department of Anesthesia, McMaster University, Hamilton, ON, Canada; †† Faculty of Health Sciences, University of Eastern Finland, Kuopio, Finland; ‡‡ Department of Surgery, Division of General Surgery, McMaster University, Hamilton, ON, Canada; §§ Department of Surgery, Vaasa Central Hospital, Vaasa, Finland; ∥∥ Department of Obstetrics and Gynecology, Turku University Hospital and University of Turku, Turku, Finland; ¶¶ Department of General Practice and Elderly Care Medicine, University Medical Center Groningen, University of Groningen, Groningen, The Netherlands; ## Direction of Health Technologies Assessment, National Center for Health Technology Excellence (CENETEC), Mexico City, Mexico; *** Departments of Gynecology and Gender Affirmation Surgery, Chelsea and Westminster NHS Foundation Trust, London, UK; ††† Anesthesiology, Perioperative Medicine, and Surgical Research Group, Population Health Research Institute, McMaster University, Hamilton, ON, Canada; ‡‡‡ Department of Outcomes Research, Outcomes Research Consortium, Cleveland, OH, USA; §§§ Department of Surgery, Division of Urology, School of Medicine, Universidad del Valle, Cali, Colombia; ∥∥∥ Department of Anesthesiology and Pain Medicine, University of Toronto, Toronto, ON, Canada; ¶¶¶ Department of Biostatistics, High Institute of Public Health, Alexandria University, Egypt; ### Department of Medical Pharmacology, Tanta Chest Hospital, Ministry of Health and Population, Tanta, Egypt; **** Health Sciences, Faculty of Medicine and Health Technology, Tampere University, Tampere, Finland; †††† Clinicum/Department of Public Health, University of Helsinki, Helsinki, Finland; ‡‡‡‡ Department of Obstetrics and Gynecology, Hospital Nova of Central Finland, Jyväskylä, Finland; §§§§ Department of Urology, University of Helsinki and Helsinki University Hospital, Helsinki, Finland; ∥∥∥∥ Department of Surgery, Central Finland Central Hospital, Jyväskylä, Finland; ¶¶¶¶ Department of Surgery, Hyvinkää Hospital, Hyvinkää, Finland; #### Department of HPB and Transplant Surgery, Freeman Hospital, Newcastle Upon Tyne, UK; ***** Population Health Sciences Institute, Faculty of Medical Sciences, Newcastle University, Newcastle Upon Tyne, UK; ††††† Department of Surgery, The University of Auckland, Auckland, New Zealand; ‡‡‡‡‡ Department of Surgery, Auckland City Hospital, Auckland, New Zealand; §§§§§ Julius Center for Health Sciences and Primary Care, University Medical Center Utrecht, Utrecht University, Utrecht, The Netherlands; ∥∥∥∥∥ Department of Nephrology and Hypertension, University Medical Center Utrecht, Utrecht University, Utrecht, The Netherlands; ¶¶¶¶¶ Department of Surgery, Division of Urology, McMaster University, Hamilton, ON, Canada; ##### Department of Nursing and West China School of Nursing, West China Hospital and Sichuan University, Chengdu, China; ****** Department of Surgery, South Karelian Central Hospital, Lappeenranta, Finland

**Keywords:** bariatric surgery, baseline risk, bleeding, colorectal surgery, general surgery, hepatopancreatobiliary surgery, risk of bias, surgery, thromboprophylaxis, venous thromboembolism

## Abstract

**Objective:**

To provide procedure-specific estimates of symptomatic venous thromboembolism (VTE) and major bleeding after abdominal surgery.

**Background:**

The use of pharmacological thromboprophylaxis represents a trade-off that depends on VTE and bleeding risks that vary between procedures; their magnitude remains uncertain.

**Methods:**

We identified observational studies reporting procedure-specific risks of symptomatic VTE or major bleeding after abdominal surgery, adjusted the reported estimates for thromboprophylaxis and length of follow-up, and estimated cumulative incidence at 4 weeks postsurgery, stratified by VTE risk groups, and rated evidence certainty.

**Results:**

After eligibility screening, 285 studies (8,048,635 patients) reporting on 40 general abdominal, 36 colorectal, 15 upper gastrointestinal, and 24 hepatopancreatobiliary surgery procedures proved eligible. Evidence certainty proved generally moderate or low for VTE and low or very low for bleeding requiring reintervention. The risk of VTE varied substantially among procedures: in general abdominal surgery from a median of <0.1% in laparoscopic cholecystectomy to a median of 3.7% in open small bowel resection, in colorectal from 0.3% in minimally invasive sigmoid colectomy to 10.0% in emergency open total proctocolectomy, and in upper gastrointestinal/hepatopancreatobiliary from 0.2% in laparoscopic sleeve gastrectomy to 6.8% in open distal pancreatectomy for cancer.

**Conclusions:**

VTE thromboprophylaxis provides net benefit through VTE reduction with a small increase in bleeding in some procedures (eg, open colectomy and open pancreaticoduodenectomy), whereas the opposite is true in others (eg, laparoscopic cholecystectomy and elective groin hernia repairs). In many procedures, thromboembolism and bleeding risks are similar, and decisions depend on individual risk prediction and values and preferences regarding VTE and bleeding.

Propelled by the growth and aging of the global population, every year surgeons perform an increasing number of general surgery procedures that now exceed 70 million^1^ annually worldwide. Although the safety of surgery has improved, complications—including venous thromboembolism (VTE) and major bleeding—remain an important concern.^2–6^ VTE encompasses deep vein thrombosis (DVT) and nonfatal or fatal pulmonary embolism (PE). Major bleeding can lead to transfusion, reintervention, or death.

Pharmacological thromboprophylaxis decreases the risk of VTE by ~50% but increases the risk of bleeding by a similar percentage.^7–10^ Therefore, the decision to use prophylaxis represents a trade-off between a reduction in VTE and an increase in bleeding. The risks of VTE and bleeding among patients not receiving prophylaxis (baseline risk) represent crucial information when deciding on the trade-off.^11^ When the baseline risk for VTE is high and the risk for bleeding is low, pharmacologic prophylaxis is likely of net benefit. Conversely, when bleeding risk is high and VTE risk is low, pharmacologic prophylaxis likely results in net harm. When risks are similar, the decision depends on the importance patients place on avoiding VTE versus avoiding bleeding (values and preferences).

General abdominal, colorectal, upper gastrointestinal (UGI), and hepatopancreatobiliary (HPB) surgery guidelines have failed to provide patient and procedure-specific guidance on pharmacological thromboprophylaxis,^7,12–17^ at least in part due to uncertainty regarding baseline risks that vary with patient and procedure-specific factors.^18^ The absence of procedure-specific recommendations contributes to substantial practice variation within and between centers and countries.^12,19–25^ To provide risk estimates of VTE and major bleeding for general abdominal, colorectal, UGI, and HPB procedures, and thus to fill this knowledge gap, we conducted a series of systematic reviews.^12^

## METHODS

We followed our registered (PROSPERO: CRD42021234119) and published study protocol,^12^ as well as “Preferred Reporting Items for Systematic Reviews and Meta-analyses” and “Meta-analysis Of Observational Studies in Epidemiology” guidances.^26–28^ The Supplemental Digital Content Appendix (Supplemental Digital Content 1, http://links.lww.com/SLA/E826) provides additional details regarding the methods.

### Eligibility

Through discussion and consensus building, expert panelists, including experienced general abdominal surgeons and clinician-methodologists, selected the most relevant general abdominal, colorectal, UGI, and HPB surgery procedures. We included observational studies that enrolled a minimum of 50 adult patients undergoing a target surgical procedure and reported the incidence of at least one patient-important outcome of interest: fatal PE, symptomatic PE, symptomatic DVT, symptomatic VTE, symptomatic splanchnic vein thrombosis (thrombosis of the portal, splenic, mesenteric, and/or supra-hepatic veins), fatal bleeding, bleeding requiring reintervention (including exploration and angioembolization), bleeding leading to transfusion, and bleeding leading to postoperative hemoglobin below 70 g/L.^12^

### Data Sources and Searches

With the aid of an information specialist (R.J.C.), we performed comprehensive searches, without language restrictions, on Embase, MEDLINE, Web of Science, and Google Scholar from January 1, 2004 to October 27, 2020. After completing the screening for the articles identified in the search, to identify additional eligible studies, we reviewed reference lists of eligible studies and relevant review articles. In a separate search, we sought randomized trials addressing the effects of thromboprophylaxis on risks of VTE and bleeding after surgery. The Supplemental Digital Content Appendix (pages 336–350, Supplemental Digital Content 1, http://links.lww.com/SLA/E826) provides details of the search strategies.

To gather information on current (2010–present) and earlier (2000–2010) thromboprophylaxis practices, we conducted a web-based survey of practicing abdominal surgeons (Supplemental Digital Content Appendix, pages 301–306, Supplemental Digital Content 1, http://links.lww.com/SLA/E826). To inform the modeling of VTE outcomes for studies with variable lengths of follow-up, we conducted a separate systematic review regarding the risk and time course of VTE by postoperative day.^29^

#### Study Selection and Data Collection

Pairs of reviewers independently assessed eligibility and risk of bias (RoB), as well as extracted data regarding procedure and patient characteristics and outcomes. We developed an instrument to categorize studies as very low, low, moderate, or high RoB.^12^ When, for a given procedure, we identified a sufficient number of articles with low or moderate RoB including a sufficient number of patients, we used RoB as an eligibility criterion (Supplemental Digital Content Appendix, page 291, Supplemental Digital Content 1, http://links.lww.com/SLA/E826). For instance, if, for a target procedure, we identified 5 or more articles at low RoB with a total of at least 1000 patients, we excluded studies with moderate or high RoB (Supplemental Digital Content Appendix, pages 6–136, Supplemental Digital Content 1, http://links.lww.com/SLA/E826). We sent our consensus data extraction to the authors of the original articles for confirmation or correction and asked for clarification regarding missing or unclear information.

### Analysis

#### Outcome Measures

The primary outcomes were the procedure-specific cumulative incidence of symptomatic VTE and major bleeding within 4 weeks (28 days) postsurgery (in the absence of the use of thromboprophylaxis). VTE included symptomatic PE, symptomatic DVT, or both in the same patient. We used 3 major bleeding definitions: (1) bleeding requiring reintervention (including exploration and angioembolization), (2) bleeding leading to the transfusion of one or more units of red blood cells, and (3) bleeding leading to postoperative hemoglobin below 70 g/L. We also separately recorded symptomatic splanchnic vein thrombosis, including thrombosis of the portal, splenic, mesenteric, or supra-hepatic veins, and recorded the incidence of fatal PE and fatal bleeding. We analyzed all outcomes separately for each type of procedure and approach and, if necessary, by indication.

#### Calculating the Risk of Venous Thromboembolism and Bleeding for Individual Studies

We adjusted the reported incidence of VTE and bleeding for the use of pharmacological and mechanical thromboprophylaxis. For patients who received prophylaxis, we multiplied the reported incidence by the relative risk of thromboprophylaxis for the duration of prophylaxis use. Our updated meta-analyses of randomized trials in general, gynecologic and urologic surgery informed the relative risk estimates of thromboprophylaxis (forest plots, Supplemental Digital Content Appendix, pages 321–335, Supplemental Digital Content 1, http://links.lww.com/SLA/E826).^7–9,30–32^ Our adjustments were as follows: (1) for unfractionated heparin and low–molecular-weight heparin risk ratio (RR) of 0.46 for VTE and 1.51 for bleeding, (2) for aspirin RR of 0.76 for VTE and 1.20 for bleeding, (3) for any mechanical prophylaxis RR 0.43 for VTE (no adjustment for bleeding), and (4) for combination therapy of pharmacologic plus mechanical (vs pharmacological alone) RR of 0.59 for VTE (no adjustment for bleeding). A recent systematic review and network meta-analysis of randomized trials in noncardiac surgery reported that direct oral anticoagulants had similar effects on both VTE and bleeding as low–molecular-weight heparin.^10^ We had high certainty in estimates of the effects of pharmacological prophylaxis but low certainty for mechanical prophylaxis (surrogate outcomes, very few patient-important events, unblinded patients and assessors; Supplemental Digital Content Appendix, pages 326–330, Supplemental Digital Content 1, http://links.lww.com/SLA/E826). Finally, we inferred that preoperative thromboprophylaxis provided only trivial extra benefits (for VTE prevention) or harm (bleeding).^33^ For studies that provided the number of DVT or PE events but not VTE, we modeled the number of VTE events using studies that had reported all DVT, PE, and VTE events (Supplemental Digital Content Appendix, page 307, Supplemental Digital Content 1, http://links.lww.com/SLA/E826).

#### Modeling the Risk of Venous Thromboembolism and Bleeding Over Time

We used cumulative incidence estimates at 4 weeks postsurgery (28 days) for our procedure-stratified estimates for the incidence of VTE and major bleeding. For the studies that did not report VTE estimates using this interval, to adjust the absolute VTE risk by postoperative day, we used the model developed in our separate systematic review.^29^ This review provided estimates of VTE occurrence on each day until 4 weeks postoperatively. For the timing of VTE from 4 weeks (28 days) to 3 months (90 days) postoperatively, we modeled estimates using a previously published approach.^7^ Using our new review information and the older approach, we developed a model for the time course of VTE from the day of surgery to 3 months postsurgery (Supplemental Digital Content Appendix, pages 312–315, Supplemental Digital Content 1, http://links.lww.com/SLA/E826).

For studies that did not report bleeding estimates using this interval, we created a new model using data from the placebo arm of a large, pragmatic randomized trial^30^ to adjust the absolute bleeding risk by postoperative day. However, as this study reported the risk of both intraoperative and postoperative bleeds without distinguishing their proportions, we modeled the proportion of intraoperative bleeds with data from studies included in the current review (Supplemental Digital Content Appendix, pages 316–320, Supplemental Digital Content 1, http://links.lww.com/SLA/E826). This model of bleeding risk over time shows that 86% of the 4-week bleeding events happen during the first week.

#### Choosing the Best Estimates

We used the median value of incidence from studies to estimate the baseline risk of VTE and major bleeding.^12^ As an incidence of 0.00% for VTE or major bleeding is implausible in general surgery, when the median estimate was 0.00% and the mean was not 0.00%, we used the mean rather than the median. If no studies reported on the incidence for a particular procedure, we used an estimate from the most similar procedure (Supplemental Digital Content Appendix, Evidence profiles, pages 6–136, Supplemental Digital Content 1, http://links.lww.com/SLA/E826). Finally, using studies that provided both estimates, we estimated the case fatality rates by dividing the number of fatal PE events by the number of symptomatic VTE events (Supplemental Digital Content Appendix, page 311, Supplemental Digital Content 1, http://links.lww.com/SLA/E826), and used a similar approach to estimate the case fatality for major bleeding. We estimated the fatal VTE and fatal major bleeding risks for procedures by taking case fatality rates of the overall reported risk of symptomatic events for the procedure.

#### Stratifying the Risk of Venous Thromboembolism and Bleeding According to Patient Risk Factors

After assessing the procedure-specific baseline risk of VTE, using a method described,^7–9^ we stratified the risk by patient-related risk factors.^34–42^ We categorized patients to low, medium, or high VTE risk using 4 risk factors (Table 1). Eligible studies and prior literature provided estimates of the proportion of patients with each of these risk factors, allowing estimates of the extent of overlap and thus calculation of estimates for each risk group (Supplemental Digital Content Appendix, page 308, Supplemental Digital Content 1, http://links.lww.com/SLA/E826). Because our search did not identify studies demonstrating convincing risk factors for bleeding,^12^ we did not stratify bleeding risk by patient-specific factors.

**TABLE 1:** Model for Risk of VTE According to Patient Risk Factors

Risk		
Low risk	No risk factors	1×
Medium risk	Any one of the following: Age 75 yr or more Body mass index 35 or more VTE in a first-degree relative (parent, full sibling, or child)	2×
High risk	Prior VTE or Patients with any combination of two or more risk factors	4×

#### Risk of Bias and Assessment of the Evidence Certainty

Methods to evaluate the RoB in longitudinal cohort studies are less developed than the methods in randomized trials.^43^ Through discussion and consensus building, and considering previous literature,^7–9,44–46^ we developed an instrument to categorize RoB of the studies^12^ that evaluated each study according to 6 domains: (1i) sampling of the study population, (2) reporting of thromboprophylaxis, (3) source of information, (4) whether a majority of patient recruitment years were earlier or later than 2010, (5) clear specification of duration of follow-up, and (6) study type (Supplemental Digital Content Appendix, page 146, Supplemental Digital Content 1, http://links.lww.com/SLA/E826). For each domain, we judged studies to have either a high or low RoB, classifying studies as follows: no high RoB domains as very low, 1 high RoB domain as low, 2 high RoB domains as moderate, and 3 or more high RoB domains as high overall RoB.^12^

We used the GRADE approach to rate the evidence certainty (also known as quality of evidence; Table 2; Supplemental Digital Content Appendix, page 293, Supplemental Digital Content 1, http://links.lww.com/SLA/E826).^47,48^ The evidence certainty from observational studies addressing a question of prognosis begins as high certainty^8,49^; owing to uncertainties in our modeling of risk of VTE and bleeding over time and patient risk strata, in all cases, we rated down to moderate.^12^ We further lowered certainty in fatal VTE, and fatal bleeding estimates to low because of uncertainties in the modeling of the cause of death. When identified, we further rated down for RoB, inconsistency of results, indirectness of evidence, or imprecision. In very low risk of VTE, even multiplying the risk by five times would lead to low (or very low) risk of VTE and would not change decisions on pharmacologic thromboprophylaxis. Therefore, if (1) the risk of VTE was 0.1% or less for all VTE risk strata and (2) the quality of evidence was low or moderate, we considered rating up evidence certainty.

**TABLE 2:** Principles for the Use of GRADE for Assessment of Evidence of the Risks of VTE and Bleeding Requiring Reintervention After Surgery

Domain	Criteria for Judgment in Our Study
RoB	We always rated down for RoB if most patients (>50%) came from studies at high RoB. We did not rate down for RoB if most patients (>50%) came from studies at low or very low RoB
Inconsistency	We rated down for inconsistency if more than 10% of the studies had at least a 3% difference from the median value of the VTE, or at least a 1.5% difference from the median value of the bleeding requiring reintervention. However, if removing outliers did not materially change the median estimate, we considered not to rate down for inconsistency
Indirectness	We did not usually rate down for indirectness, as the eligible studies measured relevant outcomes in representative populations
Imprecision	We rated down by 1 level if studies included <1000 patients and by 2 if they included <200 patients
Evidence certainty	Although certainty in a body of evidence from observational studies addressing a question of prognosis begins as high certainty, we rated it down to moderate owing to uncertainties in our models of the risk of VTE and bleeding over time and in our model of patient risk strata. We then further rated down as described for the other four categories

## RESULTS

### Study Identification

Our search identified 23,296 titles and abstracts for VTE and bleeding risk estimation, citations in reviews identified an additional 146, and reference lists from the eligible studies identified an additional 1512 for a total of 24,954 titles and abstracts. After screening titles and abstracts, we retrieved 2652 reports for full-text screening, of which 803 warranted a RoB screening (Supplemental Digital Content Appendix, page 353, Supplemental Digital Content 1, http://links.lww.com/SLA/E826, study flow chart) and 285 proved eligible (8,048,635 patients). Of these, 73 reports (including 5,977,998 patients) informed the baseline risk analyses regarding 40 general abdominal procedures, 53 reports (including 676,230 patients) informed 36 colorectal surgery procedures, 77 reports (1,302,366 patients) informed 15 UGI procedures, and 82 reports (92,041 patients) informed 24 HPB procedures. Of the 285 reports, 27 (9%) authors provided the additional information requested, corrected errors, or confirmed the accuracy of our data extraction (Supplemental Digital Content Appendix, pages 137–288 and 377, Supplemental Digital Content 1, http://links.lww.com/SLA/E826).

### Study Characteristics, Risk of Bias, and Quality of Evidence

Tables 3 and 4 present the characteristics of the eligible studies by procedure (additional details, Supplemental Digital Content Appendix, pages 137–145, 181–189, and 226–238, Supplemental Digital Content 1, http://links.lww.com/SLA/E826). For the baseline risk of VTE and bleeding, the median of the mean/median ages was lowest for sleeve gastrectomy (42 years) and appendectomy (43 years), and highest for rectopexy (78 years) (Tables 3 and 4). Of the 285 eligible studies, we judged 7 (2%) as very low, 66 (23%) as low, 70 (25%) as moderate, and 142 (50%) as high RoB (Supplemental Digital Content Appendix, pages 147–156, 190–198, and 239–251 provide details by procedure, Supplemental Digital Content 1, http://links.lww.com/SLA/E826). The evidence certainty was generally moderate or low for estimates of VTE and bleeding leading to transfusion, and low or very low for bleeding leading to reintervention (Supplemental Digital Content Tables 1–9, http://links.lww.com/SLA/E827 and the Supplemental Digital Content Appendix, pages 6–136, Supplemental Digital Content 1, http://links.lww.com/SLA/E826).

**TABLE 3:** Summary of the Included General Abdominal and Colorectal Surgery Studies by Procedure

Procedure	Studies (Patients)	Recruitment Period	Age	Length of Stay	Women (%)	Malignant (%)
Appendectomy, laparoscopic	6 (352,842)	1995–2014	43	2	53	NR
Appendectomy, open	4 (238,094)	2000–2014	43	3	48	0
Cholecystectomy, laparoscopic	22 (4,777,370)	1995–2019	51	3	73	0
Cholecystectomy, open	6 (69,435)	2002–2016	55	7	51	0
Groin hernia repair, laparoscopic	9 (18,335)	2001–2018	64	2	11	NR
Groin hernia repair, open	10 (190,077)	2002–2019	60	3	15	0
Ventral hernia repair, laparoscopic	8 (61,906)	1992–2016	55	2	56	1
Ventral hernia repair, open	8 (220,033)	1996–2018	55	6	49	0
Small bowel resection, laparoscopic	2 (3195)	2005–2016	56	NR	52	21
Small bowel resection, open	3 (28,148)	2005–2016	57	NR	51	28
Splenectomy, elective, laparoscopic	14 (7853)	1991–2018	42	5	55	18
Splenectomy, elective, open	7 (2902)	1976–2018	52	9	52	55
Abdominoperineal resection, laparoscopic	1 (2574)	2011–2015	NR	7	42	85
Abdominoperineal resection, open	1 (5107)	2011–2015	NR	10	42	80
Anterior resection, minimally invasive	11 (42,016)	1997–2016	65	7	36	100
Anterior resection, open	5 (96,114)	1997–2016	63	9	34	100
Colectomy, minimally invasive	35 (235,967)	1998–2017	61	6	51	92
Colectomy, open	17 (292,182)	2000–2017	59	8	51	100
Total proctocolectomy, laparoscopic	5 (6587)	1998–2016	39	6	45	0
Total proctocolectomy, open	5 (9744)	1997–2016	43	8	46	5
Rectopexy, laparoscopic	1 (3350)	2005–2017	61	NR	90	NA
Rectopexy, open	1 (3599)	2005–2017	64	NR	91	NA
Rectopexy, perineal	4 (5540)	1993–2017	79	6	95	NA

Minimally invasive include laparoscopic and robotic surgeries. Both age (yr) and the length of stay (d) are given as the median of the means or medians reported in the individual studies. Both the proportion of women and the proportion of malignant disease are given as the median of the proportions reported in the individual studies. Not all procedures are included in this table (Supplemental Digital Content Appendix, pages 137–145, 181–189, and 226–238, complete characteristics for all procedures).

NA indicates not applicable; NR, not reported.

**TABLE 4:** Summary of the Included UGI and HPB Surgery Studies by Procedure

Procedure	Studies (Patients)	Recruitment Period	Age	Length of Stay	Women (%)	Malignant (%)
Distal pancreatectomy, minimally invasive	9 (3902)	1997–2018	58	10	58	40
Distal pancreatectomy, laparoscopic, benign	2 (1146)	2004–2018	56	9	65	0
Distal pancreatectomy, laparoscopic, malignant	2 (1106)	2007–2016	61	15	57	100
Distal pancreatectomy, open	8 (5916)	1992–2017	65	6	51	83
Distal pancreatectomy, open, benign	1 (655)	2014–2016	61	7	58	0
Distal pancreatectomy, open, malignant	4 (3666)	2005–2017	65	8	51	100
Pancreaticoduodenectomy, minimally invasive	11 (3083)	2004–2019	62	16	48	80
Pancreaticoduodenectomy, open	32 (42,805)	1980–2019	65	16	40	89
Liver resection, minimally invasive	17 (4644)	1995–2018	62	5	53	81
Liver resection, minimally invasive, minor	2 (1210)	1998–2015	63	5	43	80
Liver resection, minimally invasive, major	3 (659)	1997–2013	62	NR	54	88
Liver resection, open	20 (31,906)	1980–2017	60	9	45	89
Liver resection, open, minor	2 (3915)	2001–2010	64	NR	37	100
Liver resection, open, major	8 (3846)	1980–2017	57	14	43	97
Gastrectomy, minimally invasive	3 (1553)	1995–2013	59	12	43	60
Gastrectomy, open	6 (22,989)	1988-2013	70	13	34	100
Subtotal gastrectomy, open	2 (1891)	1988–2010	70	12	45	100
Total gastrectomy, open	1 (999)	2005–2010	64	13	40	100
Gastric bypass, laparoscopic	9 (38,3541)	2004–2018	45	3	81	0
Gastric bypass, robotic	7 (7453)	2002–2016	42	2	83	0
Gastric bypass, open	19 (68,095)	1987–2014	45	4	79	0
Sleeve gastrectomy, laparoscopic	16 (725,690)	2006–2017	43	3	72	0
Sleeve gastrectomy, robotic	4 (14,197)	2008–2016	44	2	75	NR

Minimally invasive include laparoscopic and robotic surgeries. Both age (yr) and the length of stay (d) are given as the median of the means or medians reported in the individual studies. Both the proportion of women and the proportion of malignant disease are given as the median of the proportions reported in the individual studies. Not all procedures are included in this table (Supplemental Digital Content Appendix, pages 137–145, 181–189, and 226–238, complete characteristics for all procedures).

NA indicates not applicable; NR, not reported.

### Thromboprophylaxis Use

Of 285 studies, 48 (17%) reported both the use of and duration of pharmacological thromboprophylaxis, 39 (14%) studies reported only the proportion of patients receiving pharmacological thromboprophylaxis (without duration), and 198 (69%) studies did not report on prophylaxis use. Among studies that provided information on duration, thromboprophylaxis uses varied from 0 to 39 days (Supplemental Digital Content Appendix, pages 157–164, 199–207, and 252–263, Supplemental Digital Content 1, http://links.lww.com/SLA/E826). Of the eligible studies, 55 (19%) reported the use of mechanical prophylaxis, of which 7 (2%) also reported duration. The Supplemental Digital Content Appendix (pages 301–306, Supplemental Digital Content 1, http://links.lww.com/SLA/E826) provides details on reported prophylaxis, survey, and estimated prophylaxis durations.

### The Four Weeks Postoperative Risk of Symptomatic Venous Thromboembolism and Major Bleeding

**FIGURE 1:**
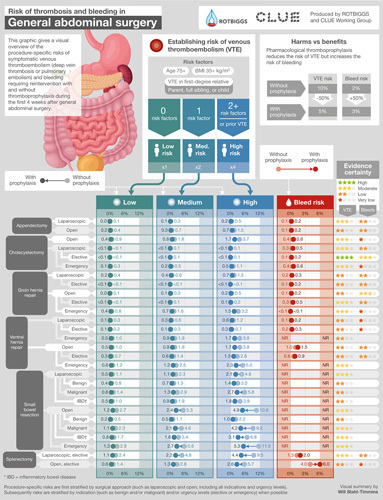
Infographic providing procedure-specific risk estimates of symptomatic VTE and bleeding requiring reintervention after general abdominal surgery procedures.

**FIGURE 2:**
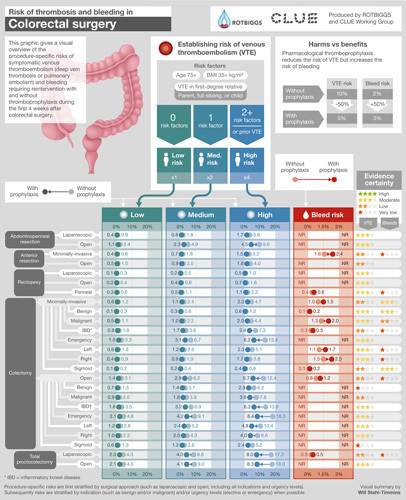
Infographic providing procedure-specific risk estimates of symptomatic VTE and bleeding requiring reintervention after colorectal surgery procedures.

**FIGURE 3:**
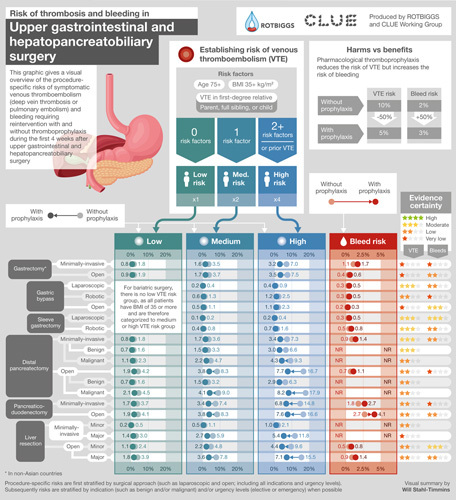
Infographic providing procedure-specific risk estimates of symptomatic VTE and bleeding requiring reintervention after upper gastrointestinal and hepatopancreatobiliary surgery procedures.

Symptomatic VTE and major bleeding risks at 4 weeks postsurgery (in the absence of thromboprophylaxis) varied widely among procedures, approaches, and indications (Figs. 1–3; Supplemental Digital Content Tables 1–9, http://links.lww.com/SLA/E827; Supplemental Digital Content Appendix Evidence Profiles pages 6–136, Supplemental Digital Content 1, http://links.lww.com/SLA/E826). Median VTE risk varied from <0.1% in laparoscopic cholecystectomy (≤0.1% across patient VTE risk groups) to 10.0% in emergency open total proctocolectomy (5.9%–-23.5% across risk groups). Of 112 procedures for which we established VTE risks, the risk was <1.0% in 36 (32%), 1.0% to 3.0% in 41 (37%), and more than 3.0% in 35 (31%).

The median risk of bleeding requiring reintervention varied from 0.1% in elective laparoscopic cholecystectomy to 4.0% in open elective splenectomy. Of 68 procedures for which we established the bleeding requiring reintervention risk, the risk was <1.0% in 46 (68%), 1.0% to 3.0% in 17 (25%), and more than 3.0% in 5 (7%) procedures. Of 85 procedures for which we established the risk of bleeding leading to transfusion, the risk varied from <0.1% in open appendectomy to 21.5% in open abdominoperineal resection. Bleeding leading to transfusion proved <1.0% in 32 (38%), 1.0% to 3.0% in 22 (26%), and more than 3.0% in 31 (36%) procedures. Studies of 4 (3%) procedures reported bleeding leading to hemoglobin below 70 g/L. The Supplemental Digital Content Appendix provides all bleeding risk estimates (evidence profiles, pages 6–136, Supplemental Digital Content 1, http://links.lww.com/SLA/E826).

#### General Abdominal Surgery

In general abdominal surgery (Fig. 1, Supplemental Digital Content Tables 1–3, http://links.lww.com/SLA/E827), none of the procedures had a median VTE risk of 2.0% or higher than the risk of bleeding requiring reintervention. For the patients with high VTE risk (Table 1), the risk of VTE was at least 2.0% higher than the risk of bleeding requiring reintervention in emergency open groin hernia repair (3.2% VTE vs <0.1% bleeding requiring reintervention), open ventral hernia repair (3.8% vs 1.0%), and elective laparoscopic splenectomy (9.5% vs 1.3%).

The median VTE and bleeding requiring reintervention risks were similar for several procedures, for instance in laparoscopic appendectomy (0.2% VTE vs 0.1% bleeding requiring reintervention), elective laparoscopic cholecystectomy (<0.1% vs 0.1%), open groin hernia repair (0.2% vs 0.1%), and elective laparoscopic ventral hernia repair (0.2% vs 0.2%). For appendectomy, the VTE risk varied from 0.1% to 1.5% across patients with VTE risk groups (Supplemental Digital Content Table 1, http://links.lww.com/SLA/E827). For cholecystectomy, the elective laparoscopic cholecystectomy had the lowest risk (≤0.1% for all VTE risk strata) and open cholecystectomy the highest (0.9%–3.7%, Supplemental Digital Content Table 1, http://links.lww.com/SLA/E827). In groin and ventral hernia repairs, the risk of VTE also varied and was lowest for elective and highest for emergency procedures (Supplemental Digital Content Table 2, http://links.lww.com/SLA/E827).

The median risk of bleeding requiring reintervention was at least 2.0% higher than the median risk of VTE for elective open splenectomy (1.8% VTE vs 4.0% bleeding requiring reintervention).

The median risk of VTE was 1.6% for laparoscopic and 3.7% for open small bowel resection (1.1%–10.6% across risk groups, Supplemental Digital Content Table 3, http://links.lww.com/SLA/E827). However, when stratified by indication and taking patient VTE risk strata into account, median VTE risk varied from 0.5% in patients with low VTE risk undergoing open small bowel resection due to benign disease to 11.5% in patients with high VTE risk undergoing open emergency small bowel resection (Supplemental Digital Content Table 3, http://links.lww.com/SLA/E827). Patients undergoing procedures performed for benign disease were generally at lower risk than patients undergoing procedures for malignant disease. We found no studies providing bleeding requiring reintervention or transfusion estimates for small bowel resections.

#### Colorectal Surgery

In colorectal surgery (Fig. 2, Supplemental Digital Content Tables 4–6, http://links.lww.com/SLA/E827), the median VTE risk was at least 2.0% points higher than the risk of bleeding requiring reintervention in open colectomy (4.4% VTE vs 0.8% bleeding requiring reintervention) and in laparoscopic total proctocolectomy (5.0% vs 0.3%). We found a 5.4% risk of symptomatic VTE after open total proctocolectomy but were unable to find bleeding requiring a reintervention estimate. For patients with high VTE risk, the VTE risk was at least 2.0% higher than the risk of bleeding requiring reintervention in minimally invasive colectomy (4.7% VTE vs 1.0% bleeding requiring reoperation), especially if the indication was inflammatory bowel disease (IBD) (7.3% vs 0.3%).

The median VTE risk was similar or somewhat higher than bleeding requiring reintervention risk for minimally invasive colectomy (1.5% VTE vs 1.0% bleeding requiring reintervention), minimally invasive left colectomy (1.9% vs 1.1%), and perineal rectopexy (1.2% vs 0.4%; Supplemental Digital Content Table 4, http://links.lww.com/SLA/E827 and Supplemental Digital Content Table 5, http://links.lww.com/SLA/E827). We found a 1.5% VTE risk for open anterior resection (including partial proctocolectomy) but did not find bleeding requiring reintervention estimate, however, bleeding leading to transfusion risk was 3.7% (Supplemental Digital Content Table 4, http://links.lww.com/SLA/E827).

The VTE and bleeding requiring reintervention risks were similar for patients with low VTE risk undergoing perineal rectopexy (0.6% vs 0.4%), minimally invasive colectomy (1.0% vs 1.0%), and minimally invasive colectomy for benign disease (0.3% vs 0.1), as well as for patients with medium VTE risk undergoing minimally invasive anterior resection (VTE 1.0% vs bleeding requiring reintervention 1.2%) (Supplemental Digital Content Table 4, http://links.lww.com/SLA/E827 and Supplemental Digital Content Table 5, http://links.lww.com/SLA/E827).

#### Upper Gastrointestinal and Hepatopancreatobiliary Surgery

In UGI and HPB surgery (Fig. 3, Supplemental Digital Content Tables 7–9, http://links.lww.com/SLA/E827), the median VTE risk was at least 2.0% points higher than the risk of bleeding requiring reintervention for minor (3.5% vs 0.5%) and major (5.3% vs 0.9%) open liver resection, open distal pancreatectomy (6.4% vs 0.7%), minimally invasive pancreaticoduodenectomy (5.3% vs 1.8%), open pancreaticoduodenectomy (6.2% vs 2.7%), and open gastrectomy (3.3% vs 0.4%).

For patients with high VTE risk, the VTE risk was at least 2.0% points higher than the risk of bleeding requiring reintervention for minimally invasive distal pancreatectomy (7.3% vs 0.9%), minimally invasive gastrectomy (7.0% vs 1.1%), and open gastric bypass (2.3% vs 0.2%).

The median risks for VTE and bleeding requiring reintervention proved similar in minimally invasive liver resection (0.8% vs 0.8%) and in minimally invasive sleeve gastrectomy (0.3% vs 0.3%) (Supplemental Digital Content Table 7, http://links.lww.com/SLA/E827 and Supplemental Digital Content Table 9, http://links.lww.com/SLA/E827). In minimally invasive gastric bypass, the median VTE risk was 0.6% and bleeding requiring reintervention risk was 0.3%. The risk of bleeding requiring reintervention was slightly higher than the risk of VTE in patients with medium VTE risk undergoing these two procedures (0.6% VTE vs 0.8% bleeding and 0.2% VTE vs 0.3% bleeding).

### The Four Weeks Postoperative Risk of Other Outcomes

We provide estimates for the procedure-specific risk of splanchnic vein thrombosis for 42 (37%) general abdominal, colorectal, UGI, and HPB surgery procedures. Splanchnic vein thrombosis risk varied between <0.1% (laparoscopic gastric bypass) and 5.2% (elective open splenectomy). The Supplemental Digital Content Appendix (pages 6–136, Supplemental Digital Content 1, http://links.lww.com/SLA/E826) provides more information: a total of 343 risk estimates for fatal VTE (n = 112), fatal major bleeding (n = 104), bleeding leading to transfusion (n = 85), and symptomatic splanchnic vein thrombosis (n = 42).

## DISCUSSION

### Main Findings

This systematic review provides the first summary of the relevant literature and current best estimates of procedure-specific risks of symptomatic VTE and major bleeding in patients not receiving thromboprophylaxis in general abdominal, colorectal, UGI, and HPB surgeries—also visually summarized in user-friendly Infographics (Figs. 1–3).

We typically found moderate to low certainty evidence for VTE and bleeding leading to transfusion and low to very low certainty in evidence for bleeding requiring reintervention. Median symptomatic VTE risk within 4 weeks after general abdominal, colorectal, UGI, and HPB surgeries varied from a <0.1% in laparoscopic cholecystectomy (≤0.1% across risk groups) to a median of 10.0% in emergency open total proctocolectomy (median 1.7%, 5.9%–23.5% across patient with VTE risk groups). Median risk of bleeding requiring reintervention within 4 weeks postsurgery varied from <0.1% to 4.0% (median, 0.5%), and bleeding leading to transfusion from <0.1% to 21.5% (median, 2.5%).

### Discussion on the Main Results, Also in Relation to Earlier Studies

#### General Surgery

Among general abdominal surgery procedures, we found a median 4-week symptomatic VTE risk of 3.7% for open small bowel resection and 2.9% for laparoscopic splenectomy (both moderate certainty). In contrast, we found that the median risk of postoperative VTE is lower in laparoscopic appendectomy (0.2%) and cholecystectomy (<0.1%), as well as in groin and ventral hernia repairs (0.6% and 0.4%) (low to moderate certainty). VTE risk also varied by indication: risk of VTE after laparoscopic and open small bowel resections was lowest when performed for benign disease (1.0% and 0.9%) compared with malignant (2.3% and 3.4%), or if performed as an emergency procedure (3.7%) (low to moderate certainty; Supplemental Digital Content Table 2, http://links.lww.com/SLA/E827). These risks may vary four-fold by patient factors (age, body mass index, and history of VTE).^7–9,12^

Bleeding requiring reintervention also varied by procedure, approach, and indication. Elective splenectomies had the highest risks of bleeding requiring reintervention (4.0% for open and 1.3% for laparoscopic), followed by open ventral hernia repair (1.0%) (very low to low certainty). Information on bleeding risk requiring reintervention was often absent (eg, laparoscopic and open small bowel resection) or very low certainty (eg, minimally invasive ventral hernia repair and open elective splenectomy).

There are no earlier comprehensive systematic reviews of the risk of VTE and bleeding in general abdominal surgeries. An earlier review article addressed the impact of thromboprophylaxis on thrombosis in hernia surgery. The authors included 2 studies (31,266 patients) and concluded that the risk of VTE is ~0.1% in groin hernia repair in patients receiving thromboprophylaxis. We included 19 studies (208,412 patients) providing 5 estimates of the risk of VTE for groin hernia repairs from <0.1% to 3.2% depending on patient risk factors and surgical approach, the median being 0.6% for laparoscopic groin hernia repair and 0.2% for open groin hernia repair (low to moderate certainty, Supplemental Digital Content Table 2, http://links.lww.com/SLA/E827). Besides VTE estimates for different approaches to groin hernia repairs, we also provided estimates for ventral hernia repairs, as well as bleeding estimates for both groin and ventral hernia repairs. An earlier systematic review and meta-analysis of 8 studies (1672 patients) addressed the incidence of VTE after laparoscopic cholecystectomy^50^ and reported 0.6% symptomatic VTE risk in patients receiving prophylaxis after laparoscopic cholecystectomy. We included 22 observational studies (4,777,370 patients) providing 3 separate estimates for laparoscopic cholecystectomy and found that the risk of symptomatic VTE varied from <0.1% to 1.1%, the median being <0.1% (moderate certainty, Supplemental Digital Content Table 1, http://links.lww.com/SLA/E827). We adjusted our estimates for the use of thromboprophylaxis and different follow-up times, stratified the VTE risk by patient risk factors, and rated the evidence certainty by the GRADE approach—none of these were performed in either of the earlier reviews.^50,51^

#### Colorectal Surgery

Among colorectal surgery procedures, symptomatic VTE risk occurred most frequently in emergency open total proctocolectomy (10.0%), followed by emergency open colectomy (6.8%) (both moderate certainty). For these procedures, information regarding bleeding requiring reintervention proved unavailable. VTE risk proved lowest after minimally invasive colectomy for benign disease and laparoscopic rectopexy (both 0.4%; both moderate certainty). Bleeding requiring reintervention risk was 0.1% after minimally invasive colectomy for benign disease (moderate certainty), risk for laparoscopic rectopexy proved unavailable. Of the 15 out of 36 colorectal procedures, for which bleeding requiring reintervention risks proved available, minimally invasive right colectomy had the highest risk (1.5%) (very low certainty), followed by minimally invasive colectomy for malignant disease (1.3%) (low certainty). For these procedures, VTE risk was 1.4% after minimally invasive right colectomy and 1.8% after laparoscopic colectomy for malignant disease. For most colorectal surgery procedures, the risks varied between these extremes (Supplemental Digital Content Tables 4–6, http://links.lww.com/SLA/E827).

VTE risk also varied by indication for surgery. After minimally invasive and open colectomies, the risk of VTE was lowest in benign disease (0.4% for minimally invasive and 2.3% for open), followed by malignant (1.8% and 3.4%) and IBD (2.1% and 4.1%), or when performed in emergency (4.8% and 6.8%) (very low to moderate certainty; Supplemental Digital Content Table 1, http://links.lww.com/SLA/E827). The risk of VTE also varied by extent of bowel resection: minimally invasive and open left (1.4%) and right (1.9%) hemicolectomies had lower VTE risk compared with total proctocolectomies or total colectomies (laparoscopic 5.0% and open 5.4%; moderate certainty; Supplemental Digital Content Table 1, http://links.lww.com/SLA/E827 and Supplemental Digital Content Table 2, http://links.lww.com/SLA/E827). Information on the risk of bleeding was unfortunately often absent. No studies provided estimates of bleeding requiring reintervention for 21 (58%) procedures or estimates of bleeding leading to transfusion for 8 (22%) procedures.

Two earlier systematic reviews have examined the risk of VTE in colorectal surgery^52,53^; neither, however, provided procedure-specific estimates. The first review examined the postoperative risk of VTE in patients with IBD undergoing colorectal surgery.^52^ The authors included 38 retrospective studies (242,992 patients) and concluded that VTE risk ranged from 0.6% to 8.9%.^52^ The second review pooled data from 8 observational studies including 344,627 colorectal cancer surgery patients^53^ and reported a pooled frequency of VTE events of 1.9% at 1-month postsurgery. We included 53 studies (676,230 patients) providing risk estimates for 36 colorectal surgery procedures. To establish clinically useful estimates, besides stratifying estimates by procedure, approach, indication, and extent of bowel resection, we adjusted for the use of thromboprophylaxis, different follow-up times, stratified the VTE risk by patient risk factors, and rated the evidence certainty by the GRADE approach—procedures not performed in the earlier reviews.^52,53^

#### Upper Gastrointestinal and Hepatopancreatobiliary Surgery

We found a high risk of VTE for open distal pancreatectomy (6.4%), as well as for minimally invasive (5.3%) and open pancreaticoduodenectomy (6.2%). Compared with the VTE risks, the risks of bleeding requiring reintervention (0.7%–2.7%) proved lower in these procedures; bleeding leading to transfusion risks were, however, larger (2.4%–9.5%). Open liver resection had appreciable VTE risk (2.6%) with a 1.1% risk of bleeding requiring reintervention and a 9.3% risk of bleeding leading to transfusion. The risks of VTE were close to risks of bleeding requiring reintervention in minimally invasive (minor) liver resection (0.8% vs 0.8%) and minimally invasive distal pancreatectomy (1.3% vs 0.8%), with risk of transfusion of 2.8% for minimally invasive liver resection and 4.4% for minimally invasive distal pancreatectomy.

We found mostly moderate or low VTE and bleeding requiring reintervention risks in bariatric surgery. After minimally invasive gastric bypass the VTE risk was 0.6% and bleeding requiring reintervention 0.3%, and after minimally invasive sleeve gastrectomy risks were 0.3% and 0.3%. The risk of VTE was appreciable for minimally invasive (2.6%) and open gastrectomy (3.3%) in non-Asian countries. We found lower VTE risks for gastrectomies when performed in Asian countries. This difference is likely multifactorial, including differences in patient population, gastric cancer screening programs, and variations in perioperative practices.

No comprehensive systematic review of the procedure-specific risk of VTE and bleeding in UGI or HPB surgery is available. A recent Cochrane review of randomized trials investigated pharmacological interventions for preventing VTE in people undergoing bariatric surgery but provided no risk estimates of VTE and bleeding.^54^ Although randomized trials provide the best estimates of treatment efficacy, they do not provide the best estimates of the baseline risks of patients in routine practice. We, therefore, included only observational studies in our review.

One systematic review and meta-analysis investigated the safety of pharmacological thromboprophylaxis after (open) liver resection.^55^ The authors included 5 observational studies (7350 patients) and reported a VTE risk of 2.6% in patients receiving pharmacological thromboprophylaxis and 4.6% risk in patients not receiving prophylaxis. We included 23 studies: 8 studies (3270 patients) providing symptomatic VTE estimates for minimally invasive liver resection and 15 studies (29,872 patients) for open liver resection. We found that the median risk of symptomatic VTE is 0.8% after minimally invasive and 2.6% after open liver resection in patients not receiving thromboprophylaxis (low certainty). We made separate estimates for minor and major liver resections from available 11 studies (8108 patients) and found a VTE risk of 3.5% after minor open liver resection and 5.3% after major open liver resection (low certainty). Reasons for differences from the previous review include our adjustment for the use of thromboprophylaxis and different follow-up times, as well as stratification by patient VTE risk factors.

### Strengths and Limitations

Strengths of this study include a comprehensive search, including screening of ~25,000 titles and abstracts and 2500 full texts. We used rigorous methods to arrive at the best estimates available for procedure-specific risks of VTE, and major bleeding and meticulously adhered to methodological standards, including duplicate independent assessment of eligibility, RoB, and data extraction. For each procedure and outcome, the GRADE system provided the framework for assessing the evidence certainty, a literature review informed assessment of patient risk factors, and our models considered the length of follow-up and the use of thromboprophylaxis. To guide estimates of VTE and bleeding risks when information on the use of thromboprophylaxis was not available, we performed a multicontinental survey addressing thromboprophylaxis use among practicing surgeons. Finally, to increase the usefulness of our work, our multidisciplinary team of practicing surgeons and methodologists, in collaboration with an information designer, created user-friendly visual and interactive infographics of the procedure-specific risks of VTE and bleeding.

This study has limitations. Observational studies have less established indexing compared with randomized trials, and we, therefore, may have missed some relevant studies. Many of our best estimates represent only low or very low-quality evidence, reflecting limitations in the available evidence. Many studies failed to provide estimates separately for procedures or approaches. Studies often reported only surrogate bleeding outcomes, such as blood loss during surgery. Most studies did not provide information regarding the use of thromboprophylaxis or the precise length of follow-up. The modeling we used to deal with inadequate reporting also has limitations,^12,29^ as a result of which we lowered our certainty in every estimate.

### Implications of the Findings

The wide variability in the use of thromboprophylaxis in general abdominal, colorectal, UGI, and HPB surgeries, both within and between countries, centers, and surgeons, reflects a lack of consensus regarding optimal practice.^12,19,20,22,23,56^ As no systematic reviews of procedure-specific risks of VTE and bleeding for general abdominal, colorectal, UGI, and HPB surgeries have heretofore been available, this variability is unsurprising.^12,15–17^ Summaries presented—and visually summarized in Infographics (Figs. 1–3)—important implications for surgery practice and will help rationalize the use of thromboprophylaxis worldwide.

When estimates clearly suggest that the benefits of VTE prevention outweigh bleeding harm, guideline panels can strongly recommend, and surgeons use pharmacologic thromboprophylaxis. For general abdominal surgery, this may be the case in laparoscopic elective splenectomy in patients with moderate and high VTE risk. Our study suggests net benefit for many colorectal procedures (eg, open colectomy and laparoscopic total proctocolectomy) and HPB procedures (eg, open distal pancreatectomy and open pancreaticoduodenectomy). When estimates suggest that VTE prevention results in net harm, guidelines may recommend against, and surgeons avoid, pharmacologic thromboprophylaxis (laparoscopic cholecystectomy, laparoscopic and open elective groin hernia repair, and patients with low risk undergoing minimally invasive sigmoid colectomy). For some procedures and patients, such as patients with moderate risk undergoing minimally invasive sleeve gastrectomy, the risk of VTE is low, and prophylaxis, therefore, results in minimal VTE reduction. In many cases, the trade-off is closer (minimally invasive right colectomy and minimally invasive liver resection) and the decision to give thromboprophylaxis will depend on individual risk prediction and values and preferences regarding VTE and bleeding consequences.

Our work has identified areas in which the published evidence is absent or of very low or low certainty; in particular, authors often omit the risk of bleeding requiring reintervention. This is true even for procedures with high volumes, such as open anterior resection and open colectomy. These areas should constitute a research priority. The methods of our review suggest methodological standards for such procedure-specific research, including comprehensive characterization and documentation of patient populations, follow-up times, thromboprophylaxis use, use of patient-important VTE, and bleeding outcomes, as well as assessment of the RoB.

## CONCLUSIONS

We performed a systematic review to provide estimates of absolute risks of symptomatic VTE and major bleeding in general, colorectal, UGI, and HPB surgery. This work represents a fundamental advance that will inform clinicians, patients, guideline developers, and policymakers in making optimal management decisions and recommendations regarding the use of thromboprophylaxis. Our results suggest a net benefit of VTE prophylaxis for some procedures, net harm for others, and a close balance in a third group. In these last situations, the decision will ideally be based on individual risk prediction and patients’ preferences regarding avoiding VTE versus bleeding.

## Supplementary Material

SUPPLEMENTARY MATERIAL
