# Supplementary material for: Systematic Reviews and Meta-analyses of the Procedure-specific Risks of Thrombosis and Bleeding in General Abdominal, Colorectal, Upper Gastrointestinal, and Hepatopancreatobiliary Surgery
Source: Ann Surg. 2023 Aug 8;279(2):213–25. doi: 10.1097/SLA.0000000000006059 (PMC10782937; doi:10.1097/SLA.0000000000006059)
Supplement: SUPPLEMENTARY MATERIAL [file sla-279-213-s001.pdf]

ROTBIGGS

Risk of Thrombosis and Bleeding
in General and Gynecologic Surgery
A study by CLUE

Systematic Reviews and Meta-analyses of the Procedure-specific Risks of Thrombosis and Bleeding in General Abdominal, Colorectal, Upper-Gastrointestinal and Hepatopancreatobiliary Surgery

Supplemental Digital Content Appendix

Table of Contents Page

1. Evidence profiles 1-40: risk of venous thromboembolism and bleeding among patients not receiving prophylaxis for general abdominal surgery procedures: procedure, approach (such as laparoscopic or open), indication (such as benign or malignant) 6

1. *Evidence profile 1. Appendectomy, laparoscopic: Absolute risk of venous thromboembolism and bleeding among patients not receiving prophylaxis 7*
2. *Evidence profile 2. Appendectomy, open: Absolute risk of venous thromboembolism and bleeding among patients not receiving prophylaxis 8*
3. *Evidence profile 3. Appendectomy, laparoscopic, emergency: Absolute risk of venous thromboembolism and bleeding among patients not receiving prophylaxis 9*
4. *Evidence profile 4. Appendectomy, open, emergency: Absolute risk of venous thromboembolism and bleeding among patients not receiving prophylaxis 10*
5. *Evidence profile 5. Cholecystectomy, laparoscopic: Absolute risk of venous thromboembolism and bleeding among patients not receiving prophylaxis 11*
6. *Evidence profile 6. Cholecystectomy, open: Absolute risk of venous thromboembolism and bleeding among patients not receiving prophylaxis 12*
7. *Evidence profile 7. Cholecystectomy, conversion to open: Absolute risk of venous thromboembolism and bleeding among patients not receiving prophylaxis 13*
8. *Evidence profile 8. Cholecystectomy, laparoscopic, elective: Absolute risk of venous thromboembolism and bleeding among patients not receiving prophylaxis 14*
9. *Evidence profile 9. Cholecystectomy, laparoscopic, emergency: Absolute risk of venous thromboembolism and bleeding among patients not receiving prophylaxis 15*
10. *Evidence profile 10. Cholecystectomy, open, emergency: Absolute risk of venous thromboembolism and bleeding among patients not receiving prophylaxis 16*
11. *Evidence profile 11. Groin hernia repair, laparoscopic: Absolute risk of venous thromboembolism and bleeding among patients not receiving prophylaxis 17*
12. *Evidence profile 12. Groin hernia repair, open: Absolute risk of venous thromboembolism and bleeding among patients not receiving prophylaxis 18*
13. *Evidence profile 13. Groin hernia repair, laparoscopic, elective: Absolute risk of venous thromboembolism and bleeding among patients not receiving prophylaxis 19*
14. *Evidence profile 14. Groin hernia repair, open, elective: Absolute risk of venous thromboembolism and bleeding among patients not receiving prophylaxis 20*
15. *Evidence profile 15. Groin hernia repair, open, emergency: Absolute risk of venous thromboembolism and bleeding among patients not receiving prophylaxis 21*

16. *Evidence profile 16. Ventral hernia repair, minimally-invasive: Absolute risk of venous thromboembolism and bleeding among patients not receiving prophylaxis* 22

17. *Evidence profile 17. Ventral hernia repair, laparoscopic: Absolute risk of venous thromboembolism and bleeding among patients not receiving prophylaxis* 23

18. *Evidence profile 18. Ventral hernia repair, robotic: Absolute risk of venous thromboembolism and bleeding among patients not receiving prophylaxis* 24

19. *Evidence profile 19. Ventral hernia repair, open: Absolute risk of venous thromboembolism and bleeding among patients not receiving prophylaxis* 25

20. *Evidence profile 20. Ventral hernia repair, laparoscopic, elective: Absolute risk of venous thromboembolism and bleeding among patients not receiving prophylaxis* 26

21. *Evidence profile 21. Ventral hernia repair, laparoscopic, emergency: Absolute risk of venous thromboembolism and bleeding among patients not receiving prophylaxis* 27

22. *Evidence profile 22. Ventral hernia repair, open, elective: Absolute risk of venous thromboembolism and bleeding among patients not receiving prophylaxis* 28

23. *Evidence profile 23. Ventral hernia repair, open, emergency: Absolute risk of venous thromboembolism and bleeding among patients not receiving prophylaxis* 29

24. *Evidence profile 24. Small bowel resection, laparoscopic: Absolute risk of venous thromboembolism and bleeding among patients not receiving prophylaxis* 30

25. *Evidence profile 25. Small bowel resection, open: Absolute risk of venous thromboembolism and bleeding among patients not receiving prophylaxis* 31

26. *Evidence profile 26. Small bowel resection, laparoscopic, benign: Absolute risk of venous thromboembolism and bleeding among patients not receiving prophylaxis* 32

27. *Evidence profile 27. Small bowel resection, laparoscopic, malignant: Absolute risk of venous thromboembolism and bleeding among patients not receiving prophylaxis* 33

28. *Evidence profile 28. Small bowel resection, laparoscopic, IBD: Absolute risk of venous thromboembolism and bleeding among patients not receiving prophylaxis* 34

29. *Evidence profile 29. Small bowel resection, laparoscopic, emergency: Absolute risk of venous thromboembolism and bleeding among patients not receiving prophylaxis* 35

30. *Evidence profile 30. Small bowel resection, open, benign: Absolute risk of venous thromboembolism and bleeding among patients not receiving prophylaxis* 36

31. *Evidence profile 31. Small bowel resection, open, malignant: Absolute risk of venous thromboembolism and bleeding among patients not receiving prophylaxis* 37

32. *Evidence profile 32. Small bowel resection, open, inflammatory bowel disease (IBD): Absolute risk of venous thromboembolism and bleeding among patients not receiving prophylaxis* 38

33. *Evidence profile 33. Small bowel resection, open, emergency: Absolute risk of venous thromboembolism and bleeding among patients not receiving prophylaxis* 39

34. *Evidence profile 34. Splenectomy, laparoscopic, elective: Absolute risk of venous thromboembolism and bleeding among patients not receiving prophylaxis* 40

35. *Evidence profile 35. Splenectomy, open, elective: Absolute risk of venous thromboembolism and bleeding among patients not receiving prophylaxis* 41

36. *Evidence profile 36. Splenectomy, laparoscopic, elective, benign: Absolute risk of venous thromboembolism and bleeding among patients not receiving prophylaxis* 42

37. *Evidence profile 37. Splenectomy, laparoscopic, elective, immune thrombocytopenia (ITP): Absolute risk of venous thromboembolism and bleeding among patients not receiving prophylaxis* 43

38. *Evidence profile 38. Splenectomy, open, elective, benign: Absolute risk of venous thromboembolism and bleeding among patients not receiving prophylaxis* 44

39. *Evidence profile 39. Splenectomy, open, elective, malignant: Absolute risk of venous thromboembolism and bleeding among patients not receiving prophylaxis* 45

40. *Evidence profile 40. Splenectomy, open, elective, immune thrombocytopenia (ITP): Absolute risk of venous thromboembolism and bleeding among patients not receiving prophylaxis* 46

2. Evidence profiles 41-74: risk of venous thromboembolism and bleeding among patients not receiving prophylaxis for colorectal surgery procedures: procedure, specification (such as left or total), approach (such as laparoscopic or open), indication (such as benign or malign) 47

41. *Evidence profile 41. Abdominoperineal resection, laparoscopic: Absolute risk of venous thromboembolism and bleeding among patients not receiving prophylaxis* 48

42. *Evidence profile 42. Abdominoperineal resection, open: Absolute risk of venous thromboembolism and bleeding among patients not receiving prophylaxis* 49

43. *Evidence profile 43. Anterior resection, minimally-invasive: Absolute risk of venous thromboembolism and bleeding among patients not receiving prophylaxis* 50

44. *Evidence profile 44. Anterior resection, laparoscopic: Absolute risk of venous thromboembolism and bleeding among patients not receiving prophylaxis* 51

45. *Evidence profile 45. Anterior resection, open: Absolute risk of venous thromboembolism and bleeding among patients not receiving prophylaxis* 52

46. *Evidence profile 46. Anterior resection, robotic: Absolute risk of venous thromboembolism and bleeding among patients not receiving prophylaxis* 53

47. *Evidence profile 47. Colectomy, minimally-invasive: Absolute risk of venous thromboembolism and bleeding among patients not receiving prophylaxis* 54

48. *Evidence profile 48. Colectomy, laparoscopic: Absolute risk of venous thromboembolism and bleeding among patients not receiving prophylaxis* 55

49. *Evidence profile 49. Colectomy, open: Absolute risk of venous thromboembolism and bleeding among patients not receiving prophylaxis* 56

50. *Evidence profile 50. Colectomy, robotic: Absolute risk of venous thromboembolism and bleeding among patients not receiving prophylaxis* 57

51. *Evidence profile 51. Colectomy, minimally-invasive, benign: Absolute risk of venous thromboembolism and bleeding among patients not receiving prophylaxis* 58

52. *Evidence profile 52. Colectomy, minimally-invasive, malignant: Absolute risk of venous thromboembolism and bleeding among patients not receiving prophylaxis* 59

53. *Evidence profile 53. Colectomy, minimally-invasive, inflammatory bowel disease: Absolute risk of venous thromboembolism and bleeding among patients not receiving prophylaxis* 60

54. *Evidence profile 54. Colectomy, minimally-invasive, emergency: Absolute risk of venous thromboembolism and bleeding among patients not receiving prophylaxis* 61

55. *Evidence profile 55. Colectomy, open, benign: Absolute risk of venous thromboembolism and bleeding among patients not receiving prophylaxis* 62

56. *Evidence profile 56. Colectomy, open, malignant: Absolute risk of venous thromboembolism and bleeding among patients not receiving prophylaxis* 63

57. *Evidence profile 57. Colectomy, open, inflammatory bowel disease: Absolute risk of venous thromboembolism and bleeding among patients not receiving prophylaxis* 64

58. *Evidence profile 58. Colectomy, open, emergency: Absolute risk of venous thromboembolism and bleeding among patients not receiving prophylaxis* 65

59. *Evidence profile 59. Colectomy, left, minimally-invasive: Absolute risk of venous thromboembolism and bleeding among patients not receiving prophylaxis* 66

60. *Evidence profile 60. Colectomy, right, minimally-invasive: Absolute risk of venous thromboembolism and bleeding among patients not receiving prophylaxis* 67

61. *Evidence profile 61. Colectomy, left, open: Absolute risk of venous thromboembolism and bleeding among patients not receiving prophylaxis* 68

62. *Evidence profile 62. Colectomy, right, open: Absolute risk of venous thromboembolism and bleeding among patients not receiving prophylaxis* 69

63. *Evidence profile 63. Total proctocolectomy, laparoscopic: Absolute risk of venous thromboembolism and bleeding among patients not receiving prophylaxis* 70

64. *Evidence profile 64. Total proctocolectomy, open: Absolute risk of venous thromboembolism and bleeding among patients not receiving prophylaxis* 71

65. *Evidence profile 65. Total proctocolectomy, laparoscopic, benign: Absolute risk of venous thromboembolism and bleeding among patients not receiving prophylaxis* 72

66. *Evidence profile 66. Total proctocolectomy, laparoscopic, malignant: Absolute risk of venous thromboembolism and bleeding among patients not receiving prophylaxis* 73

67. *Evidence profile 67. Total proctocolectomy, laparoscopic, inflammatory bowel disease: Absolute risk of venous thromboembolism and bleeding among patients not receiving prophylaxis* 74

68. *Evidence profile 68. Total proctocolectomy, open, benign: Absolute risk of venous thromboembolism and bleeding among patients not receiving prophylaxis* 75

69. *Evidence profile 69. Total proctocolectomy, open, malignant: Absolute risk of venous thromboembolism and bleeding among patients not receiving prophylaxis* 76

70. *Evidence profile 70. Total proctocolectomy, open, inflammatory bowel disease: Absolute risk of venous thromboembolism and bleeding among patients not receiving prophylaxis* 77

71. *Evidence profile 71. Total proctocolectomy, open, emergency: Absolute risk of venous thromboembolism and bleeding among patients not receiving prophylaxis* 78

72. *Evidence profile 72. Rectopexy, laparoscopic: Absolute risk of venous thromboembolism and bleeding among patients not receiving prophylaxis* 79

73. *Evidence profile 73. Rectopexy, open: Absolute risk of venous thromboembolism and bleeding among patients not receiving prophylaxis* 80

74. *Evidence profile 74. Rectopexy, perineal: Absolute risk of venous thromboembolism and bleeding among patients not receiving prophylaxis* 81

3. Evidence profiles 75-128: risk of venous thromboembolism and bleeding among patients not receiving prophylaxis for upper-gastrointestinal and hepatopancreatobiliary surgery procedures: procedure, approach (such as laparoscopic or open), specification (such as minor or major), indication (such as benign or malign) 82

75. *Evidence profile 75. Distal pancreatectomy, minimally-invasive: Absolute risk of venous thromboembolism and bleeding among patients not receiving prophylaxis* 83

76. *Evidence profile 76. Distal pancreatectomy, laparoscopic: Absolute risk of venous thromboembolism and bleeding among patients not receiving prophylaxis* 84

77. *Evidence profile 77. Distal pancreatectomy, robotic: Absolute risk of venous thromboembolism and bleeding among patients not receiving prophylaxis* 85

78. *Evidence profile 78. Distal pancreatectomy, open: Absolute risk of venous thromboembolism and bleeding among patients not receiving prophylaxis* 86

79. *Evidence profile 79. Distal pancreatectomy, laparoscopic, benign: Absolute risk of venous thromboembolism and bleeding among patients not receiving prophylaxis* 87

80. *Evidence profile 80. Distal pancreatectomy, laparoscopic, malignant: Absolute risk of venous thromboembolism and bleeding among patients not receiving prophylaxis* 88

81. *Evidence profile 81. Distal pancreatectomy, open, benign: Absolute risk of venous thromboembolism and bleeding among patients not receiving prophylaxis* 89

82. *Evidence profile 82. Distal pancreatectomy, open, malignant: Absolute risk of venous thromboembolism and bleeding among patients not receiving prophylaxis* 90

83. *Evidence profile 83. Liver resection, minimally-invasive: Absolute risk of venous thromboembolism and bleeding among patients not receiving prophylaxis* 91

84. *Evidence profile 84. Liver resection, laparoscopic: Absolute risk of venous thromboembolism and bleeding among patients not receiving prophylaxis* 92

85. *Evidence profile 85. Liver resection, robotic: Absolute risk of venous thromboembolism and bleeding among patients not receiving prophylaxis* 93

86. *Evidence profile 86. Liver resection, open: Absolute risk of venous thromboembolism and bleeding among patients not receiving prophylaxis* 94

87. *Evidence profile 87. Liver resection, laparoscopic, minor: Absolute risk of venous thromboembolism and bleeding among patients not receiving prophylaxis* 95

88. *Evidence profile 88. Liver resection, laparoscopic, major: Absolute risk of venous thromboembolism and bleeding among patients not receiving prophylaxis* 96

89. *Evidence profile 89. Liver resection, open, minor: Absolute risk of venous thromboembolism and bleeding among patients not receiving prophylaxis* 97

90. *Evidence profile 90. Liver resection, open, major: Absolute risk of venous thromboembolism and bleeding among patients not receiving prophylaxis* 98

91. *Evidence profile 91. Pancreaticoduodenectomy, minimally-invasive: Absolute risk of venous thromboembolism and bleeding among patients not receiving prophylaxis* 99

92. *Evidence profile 92. Pancreaticoduodenectomy, laparoscopic: Absolute risk of venous thromboembolism and bleeding among patients not receiving prophylaxis* 100

93. *Evidence profile 93. Pancreaticoduodenectomy, robotic: Absolute risk of venous thromboembolism and bleeding among patients not receiving prophylaxis* 101

94. *Evidence profile 94. Pancreaticoduodenectomy, open: Absolute risk of venous thromboembolism and bleeding among patients not receiving prophylaxis* 102

95. *Evidence profile 95. Pancreaticoduodenectomy without vascular resection, laparoscopic: Absolute risk of venous thromboembolism and bleeding among patients not receiving prophylaxis* 103

96. *Evidence profile 96. Pancreaticoduodenectomy with vascular resection, laparoscopic: Absolute risk of venous thromboembolism and bleeding among patients not receiving prophylaxis* 104

97. *Evidence profile 97. Pancreaticoduodenectomy without vascular resection, open: Absolute risk of venous thromboembolism and bleeding among patients not receiving prophylaxis* 105

98. *Evidence profile 98. Pancreaticoduodenectomy with vascular resection, open: Absolute risk of venous thromboembolism and bleeding among patients not receiving prophylaxis* 106

99. *Evidence profile 99. Gastrectomy, minimally-invasive: Absolute risk of venous thromboembolism and bleeding among patients not receiving prophylaxis* 107

100. *Evidence profile 100. Gastrectomy, laparoscopic: Absolute risk of venous thromboembolism and bleeding among patients not receiving prophylaxis* 108

101. *Evidence profile 101. Gastrectomy, robotic: Absolute risk of venous thromboembolism and bleeding among patients not receiving prophylaxis* 109

102. *Evidence profile 102. Gastrectomy, open: Absolute risk of venous thromboembolism and bleeding among patients not receiving prophylaxis* 110

103. *Evidence profile 103. Subtotal gastrectomy, laparoscopic: Absolute risk of venous thromboembolism and bleeding among patients not receiving prophylaxis* 111

104. Evidence profile 104. Total gastrectomy, laparoscopic: Absolute risk of venous thromboembolism and bleeding among patients not receiving prophylaxis 112

105. Evidence profile 105. Subtotal gastrectomy, open: Absolute risk of venous thromboembolism and bleeding among patients not receiving prophylaxis 113

106. Evidence profile 106. Total gastrectomy, open: Absolute risk of venous thromboembolism and bleeding among patients not receiving prophylaxis 114

107. Evidence profile 107. Gastrectomy, minimally-invasive, in Asia: Absolute risk of venous thromboembolism and bleeding among patients not receiving prophylaxis 115

108. Evidence profile 108. Gastrectomy, laparoscopic, in Asia: Absolute risk of venous thromboembolism and bleeding among patients not receiving prophylaxis 116

109. Evidence profile 109. Gastrectomy, robotic, in Asia: Absolute risk of venous thromboembolism and bleeding among patients not receiving prophylaxis 117

110. Evidence profile 110. Gastrectomy, open, in Asia: Absolute risk of venous thromboembolism and bleeding among patients not receiving prophylaxis 118

111. Evidence profile 111. Subtotal gastrectomy, laparoscopic, in Asia: Absolute risk of venous thromboembolism and bleeding among patients not receiving prophylaxis 119

112. Evidence profile 112. Total gastrectomy, laparoscopic, in Asia: Absolute risk of venous thromboembolism and bleeding among patients not receiving prophylaxis 120

113. Evidence profile 113. Subtotal gastrectomy, open, in Asia: Absolute risk of venous thromboembolism and bleeding among patients not receiving prophylaxis 121

114. Evidence profile 114. Total gastrectomy, open, in Asia: Absolute risk of venous thromboembolism and bleeding among patients not receiving prophylaxis 122

115. Evidence profile 115. Gastrectomy, minimally-invasive, in non-Asian countries: Absolute risk of venous thromboembolism and bleeding among patients not receiving prophylaxis 123

116. Evidence profile 116. Gastrectomy, laparoscopic, in non-Asian countries: Absolute risk of venous thromboembolism and bleeding among patients not receiving prophylaxis 124

117. Evidence profile 117. Gastrectomy, robotic, in non-Asian countries: Absolute risk of venous thromboembolism and bleeding among patients not receiving prophylaxis 125

118. Evidence profile 118. Gastrectomy, open, in non-Asian countries: Absolute risk of venous thromboembolism and bleeding among patients not receiving prophylaxis 126

119. Evidence profile 119. Subtotal gastrectomy, laparoscopic, in non-Asian countries: Absolute risk of venous thromboembolism and bleeding among patients not receiving prophylaxis 127

120. Evidence profile 120. Subtotal gastrectomy, open, in non-Asian countries: Absolute risk of venous thromboembolism and bleeding among patients not receiving prophylaxis 128

121. Evidence profile 121. Total gastrectomy, open, in non-Asian countries: Absolute risk of venous thromboembolism and bleeding among patients not receiving prophylaxis 129

122. Evidence profile 122. Gastric bypass, minimally-invasive: Absolute risk of venous thromboembolism and bleeding among patients not receiving prophylaxis 130

123. Evidence profile 123. Gastric bypass, laparoscopic: Absolute risk of venous thromboembolism and bleeding among patients not receiving prophylaxis 131

124. Evidence profile 124. Gastric bypass, robotic: Absolute risk of venous thromboembolism and bleeding among patients not receiving prophylaxis 132

125. Evidence profile 125. Gastric bypass, open: Absolute risk of venous thromboembolism and bleeding among patients not receiving prophylaxis 133

126. Evidence profile 126. Sleeve gastrectomy, minimally-invasive: Absolute risk of venous thromboembolism and bleeding among patients not receiving prophylaxis 134

127. Evidence profile 127. Sleeve gastrectomy, laparoscopic: Absolute risk of venous thromboembolism and bleeding among patients not receiving prophylaxis 135

128. Evidence profile 128. Sleeve gastrectomy, robotic: Absolute risk of venous thromboembolism and bleeding among patients not receiving prophylaxis 136

4. General abdominal surgery supplementary tables 1-6 **137**

1. Characteristics of individual studies in general abdominal surgery 137

2. Design features used for assessment of risk of bias 146

3. Risk of bias in individual studies in general abdominal surgery 147

4. Prophylaxis in individual studies in general abdominal surgery 157

5. Postoperative risk of symptomatic VTE and bleeding in individual studies in general abdominal surgery 165

6. Peri- and intraoperative risk of symptomatic VTE and bleeding in individual studies in general abdominal surgery 173

5. Colorectal surgery supplementary tables 7-11 **181**

7. Characteristics of individual studies in colorectal surgery 181

8. Risk of bias in individual studies in colorectal surgery 190

9. Prophylaxis in individual studies in colorectal surgery 199

10. Postoperative risk of symptomatic VTE and bleeding in individual studies in colorectal surgery 208

11. Peri- and intraoperative risk of bleeding in individual studies in colorectal surgery 217

6. Upper-gastrointestinal and hepatopancreatobiliary surgery supplementary tables 12-17 **226**

12. Characteristics of individual studies in upper-gastrointestinal and hepatopancreatobiliary surgery 226

13. Risk of bias in individual studies in upper-gastrointestinal and hepatopancreatobiliary surgery 239

14. Prophylaxis in individual studies in upper-gastrointestinal and hepatopancreatobiliary surgery 252

15. Postoperative risk of symptomatic VTE and bleeding in individual studies in upper-gastrointestinal and hepatopancreatobiliary surgery 264

16. Peri- and intraoperative risk of bleeding in individual studies in upper-gastrointestinal and hepatopancreatobiliary surgery 277

7. Supplementary methods **289**

1. Eligibility 289

2. Data sources and searches 289

3. Study selection and data collection 289

4. Analysis 290

1. Outcome measures 290
2. Calculating the risk of VTE and bleeding for individual studies 290
3. Modeling the risk of VTE and bleeding over time 290
4. Choosing the best estimates 291
5. Stratifying the risk of VTE and bleeding according to patient risk factors 291
6. Supplementary table 18. Risk of venous thromboembolism according to patient risk factors 291
7. Risk of bias and assessment of the evidence certainty 292
8. Supplementary table 19. Principles for the use of GRADE for assessment of evidence of risk of complications, and examples of GRADE use for estimating evidence of the risks of VTE and bleeding requiring reintervention after general abdominal surgery 293

5. *Calculating baseline risks* *297*

6. *Missing thromboprophylaxis information* *301*
   1. Principles 301
   2. Supplementary table 20: Missing mechanical thromboprophylaxis 305
   3. Supplementary table 21: Missing pharmacological thromboprophylaxis 306

7. *Overlap of DVT, PE, and VTE: How we dealt with studies that did not provide the number of VTE but provided DVT, PE, or both* *307*

8. *Patient risk strata* *308*

9. *Case fatality and estimates of fatal VTE and fatal bleeding* *311*

8. Timing of VTE and bleeding during the first 90 post-operative days: **312**

1. *Proportion of cumulative risk of VTE by day since surgery during the first 90 post-operative days* *312*
   1. Supplementary figure 1: Proportion of cumulative risk (%) of venous thromboembolism during the first 90 post-operative days 313
   2. Supplementary table 22: Proportion of cumulative risk (%) of venous thromboembolism during the first 90 post-operative days 314

2. *Proportion of cumulative incidence of major bleeding by day since surgery during the first 90 post-operative days* *316*
   1. Supplementary figure 2: Proportion of cumulative incidence (%) of major bleeding during the first 90 post-operative days 317
   2. Supplementary table 23: Proportion of cumulative incidence (%) of major bleeding during the first 90 post-operative days 318

9. Forest plots for effects of pharmacological and mechanical thromboprophylaxis on VTE and bleeding **321**

1. *Unfractionated heparin or low-molecular-weight heparin versus no prophylaxis: non-fatal pulmonary embolism* *321*

2. *Unfractionated heparin or low-molecular-weight heparin versus no prophylaxis: non-fatal bleeding* *322*

3. *Unfractionated heparin or low-molecular-weight heparin versus no prophylaxis: fatal pulmonary embolism* *323*

4. *Unfractionated heparin or low-molecular-weight heparin versus no prophylaxis: fatal bleeding* *324*

5. *Unfractionated heparin or low-molecular-weight heparin versus no prophylaxis: death from any cause* *325*

6. *Elastic stockings versus no prophylaxis: deep vein thrombosis on surveillance* *326*

7. *Elastic stockings versus no prophylaxis: pulmonary embolism* *326*

8. *Elastic stockings versus no prophylaxis: any venous thromboembolism* *326*

9. *Intermittent pneumatic compression device versus no prophylaxis: deep vein thrombosis on surveillance* *327*

10. *Intermittent pneumatic compression device versus no prophylaxis: pulmonary embolism* *327*

11. *Intermittent pneumatic compression device versus no prophylaxis: any venous thromboembolism* *327*

12. *Any mechanical prophylaxis versus no prophylaxis: deep vein thrombosis on surveillance* *328*

13. *Any mechanical prophylaxis versus no prophylaxis: pulmonary embolism* *329*

14. *Any mechanical prophylaxis versus no prophylaxis: any venous thromboembolism* *330*

15. *Any mechanical plus any pharmacological versus any pharmacological: deep vein thrombosis on surveillance* *331*

16. *Any mechanical plus any pharmacological versus any pharmacological: symptomatic deep vein thrombosis* *332*

17. *Any mechanical plus any pharmacological versus any pharmacological: pulmonary embolism* *333*

18. Any mechanical plus any pharmacological versus any pharmacological: any venous thromboembolism 334

19. Aspirin versus placebo: symptomatic VTE 335

10. Search histories 336

1. Search history for baseline risk of VTE and Major Bleeding 336

2. Search history update searches for baseline risk of VTE and Major Bleeding 342

3. Search history for patient related risk factors of major bleeding/bleeding requiring reintervention after surgery 348

4. Search history for effects of pharmacological and mechanical thromboprophylaxis on VTE and bleeding349

11. PRISMA 2020 Checklist 351

12. PRISMA 2020 Flow diagram 353

13. MOOSE Checklist for Meta-analyses of Observational Studies 354

14. List of included studies 356

1. General abdominal surgery 356

2. Colorectal surgery 360

3. Upper-gastrointestinal and hepatopancreatobiliary surgery 363

11. Acknowledgements of authors of original articles 377

1. Evidence profiles 1-40: risk of venous thromboembolism and bleeding among patients not receiving prophylaxis for general abdominal surgery procedures: procedure, approach (such as laparoscopic or open), indication (such as benign or malignant)

1. Evidence profile 1. Appendectomy, laparoscopic: Absolute risk of venous thromboembolism and bleeding among patients not receiving prophylaxis

Quality assessment					Summary of findings		
**No of participants (studies)**	**Risk of Bias**	**Inconsistency**	**Indirectness**	**Imprecision**	**Best (median) estimate across all risk strata (%)***	**Best (median) estimate by patient risk strata (%)†**	**Evidence certainty‡**
**Non-fatal symptomatic venous thromboembolism**							
352,842 (6)	No serious limitations	No serious limitations	No serious limitations	No serious limitations	0.15	**Low: 0.12 Medium: 0.25 High: 0.50**	**Moderate**
**Fatal venous thromboembolism**							
352,842 (6)	No serious limitations	No serious limitations	No serious limitations	No serious limitations	0.01	**Low: 0 Medium: 0.01 High: 0.02**	**Low**
**Symptomatic splanchnic vein thrombosis**							
**Non-fatal bleeding requiring reintervention¶**							
10959 (9)	Serious limitations	No serious limitations	Serious limitations	No serious limitations	0.10	**0.10**	**Low**
**Non-fatal bleeding leading to transfusion**							
22,891 (2)	No serious limitations	No serious limitations	No serious limitations	No serious limitations	0.07	**0.07**	**Moderate**
**Fatal bleeding**							
22,891 (2)	No serious limitations	No serious limitations	No serious limitations	No serious limitations	0.00	**0.00**	**Low**
**Bleeding leading to hemoglobin below 70g/L (7g/dL)**							

Blank spaces indicate absence of information

* Estimate represents absolute risk in percent. Our median best estimates include fatal and non-fatal events. Based on data from included studies, we estimated case fatality rates as follows: 3.6% for VTE, 3.6% for bleeding leading to reintervention, and 0.9% for bleeding leading to transfusion, and used this information to calculate outcome estimates. For instance, we multiplied the median VTE estimate by 0.964 for non-fatal VTE and by 0.036 for fatal VTE (if both reintervention and transfusion rates were available, we preferred reintervention estimates for calculation of fatal bleeding estimate).

† Risk factors included 1) age more than 75 years, 2) obesity (body mass index of 35 or more), 3) VTE in a first degree relative (parents, full siblings, or children), and 4) prior VTE. We assumed that patients with any combination of two or more risk factors had a risk ratio of 4. Using these risk factors, we then categorized risk of VTE as low, medium, and high risk.

‡ Options for certainty in estimates are high, moderate, low, and very low. Evidence begins as high and is rated down for serious risk of bias, inconsistency, imprecision, or indirectness. We always rated down once due to uncertainty in the patient VTE risk factors and models of timing of VTE and bleeding. For fatal VTE and fatal bleeding we always rated down once for uncertainty in our case fatality rate estimates.

¶ We did not have any studies providing estimates for bleeding requiring reintervention. To estimate the risk expert panels considered the bleeding risk to be half that of laparoscopic cholecystectomy, or same as in open groin hernia. We also had direct evidence for bleeding requiring transfusion. We therefore considered this procedure to have serious limitations due to indirectness.

2. Evidence profile 2. Appendectomy, open: Absolute risk of venous thromboembolism and bleeding among patients not receiving prophylaxis

Certainty assessment					Summary of findings		
**No of participants (studies)**	**Risk of Bias**	**Inconsistency**	**Indirectness**	**Imprecision**	**Best (median) estimate across all risk strata (%)***	**Best (median) estimate by patient risk strata (%)†**	**Evidence certainty‡**
**Non-fatal symptomatic venous thromboembolism**							
238,094 (4)	Serious limitations	No serious limitations	No serious limitations	No serious limitations	0.43	**Low: 0.35 Medium: 0.71 High: 1.42**	**Low**
**Fatal venous thromboembolism**							
238,094 (4)	Serious limitations	No serious limitations	No serious limitations	No serious limitations	0.02	**Low: 0.01 Medium: 0.03 High: 0.05**	**Very Low**
**Symptomatic splanchnic vein thrombosis**							
**Non-fatal bleeding requiring reintervention¶**							
5222 (3)	No serious limitations	No serious limitations	Serious limitations	No serious limitations	0.10	**0.10**	**Low**
**Non-fatal bleeding leading to transfusion**							
6,030 (1)	No serious limitations	No serious limitations	No serious limitations	No serious limitations	0.01	**0.01**	**Moderate**
**Fatal bleeding**							
6,030 (1)	No serious limitations	No serious limitations	No serious limitations	No serious limitations	0.00	**0.00**	**Low**
**Bleeding leading to hemoglobin below 70g/L (7g/dL)**							

Blank spaces indicate absence of information

* Estimate represents absolute risk in percent. Our median best estimates include fatal and non-fatal events. Based on data from included studies, we estimated case fatality rates as follows: 3.6% for VTE, 3.6% for bleeding leading to reintervention, and 0.9% for bleeding leading to transfusion, and used this information to calculate outcome estimates. For instance, we multiplied the median VTE estimate by 0.964 for non-fatal VTE and by 0.036 for fatal VTE (if both reintervention and transfusion rates were available, we preferred reintervention estimates for calculation of fatal bleeding estimate).

† Risk factors included 1) age more than 75 years, 2) obesity (body mass index of 35 or more), 3) VTE in a first degree relative (parents, full siblings, or children), and 4) prior VTE. We assumed that patients with any combination of two or more risk factors had a risk ratio of 4. Using these risk factors, we then categorized risk of VTE as low, medium, and high risk.

‡ Options for certainty in estimates are high, moderate, low, and very low. Evidence begins as high and is rated down for serious risk of bias, inconsistency, imprecision, or indirectness. We always rated down once due to uncertainty in the patient VTE risk factors and models of timing of VTE and bleeding. For fatal VTE and fatal bleeding we always rated down once for uncertainty in our case fatality rate estimates.

¶We did not have any studies providing estimates for bleeding requiring reintervention. To estimate the risk expert panels considered the bleeding risk to be same as in open groin hernia. We also had direct evidence for bleeding requiring transfusion. We therefore considered this estimate to have serious limitations due to indirectness.

3. Evidence profile 3. Appendectomy, laparoscopic, emergency: Absolute risk of venous thromboembolism and bleeding among patients not receiving prophylaxis

Quality assessment					Summary of findings		
**No of participants (studies)**	**Risk of Bias**	**Inconsistency**	**Indirectness**	**Imprecision**	**Best (median) estimate across all risk strata (%)***	**Best (median) estimate by patient risk strata (%)†**	**Evidence certainty‡**
**Non-fatal symptomatic venous thromboembolism**							
72,463 (2)	No serious limitations	No serious limitations	No serious limitations	No serious limitations	0.16	**Low: 0.13 Medium: 0.27 High: 0.54**	**Moderate**
**Fatal venous thromboembolism**							
72,463 (2)	No serious limitations	No serious limitations	No serious limitations	No serious limitations	0.01	**Low: 0.01 Medium: 0.01 High: 0.02**	**Low**
**Symptomatic splanchnic vein thrombosis**							
**Non-fatal bleeding requiring reintervention**							
**Non-fatal bleeding leading to transfusion**							
7,446 (1)	Serious limitations	No serious limitations	No serious limitations	No serious limitations	0.13	**0.13**	**Low**
**Fatal bleeding**							
7,446 (1)	Serious limitations	No serious limitations	No serious limitations	No serious limitations	0.00	**0.00**	**Very Low**
**Bleeding leading to hemoglobin below 70g/L (7g/dL)**							

Blank spaces indicate absence of information

* Estimate represents absolute risk in percent. Our median best estimates include fatal and non-fatal events. Based on data from included studies, we estimated case fatality rates as follows: 3.6% for VTE, 3.6% for bleeding leading to reintervention, and 0.9% for bleeding leading to transfusion, and used this information to calculate outcome estimates. For instance, we multiplied the median VTE estimate by 0.964 for non-fatal VTE and by 0.036 for fatal VTE (if both reintervention and transfusion rates were available, we preferred reintervention estimates for calculation of fatal bleeding estimate).

† Risk factors included 1) age more than 75 years, 2) obesity (body mass index of 35 or more), 3) VTE in a first degree relative (parents, full siblings, or children), and 4) prior VTE. We assumed that patients with any combination of two or more risk factors had a risk ratio of 4. Using these risk factors, we then categorized risk of VTE as low, medium, and high risk.

‡ Options for certainty in estimates are high, moderate, low, and very low. Evidence begins as high and is rated down for serious risk of bias, inconsistency, imprecision, or indirectness. We always rated down once due to uncertainty in the patient VTE risk factors and models of timing of VTE and bleeding. For fatal VTE and fatal bleeding we always rated down once for uncertainty in our case fatality rate estimates.

4. Evidence profile 4. Appendectomy, open, emergency: Absolute risk of venous thromboembolism and bleeding among patients not receiving prophylaxis

Quality assessment					Summary of findings		
**No of participants (studies)**	**Risk of Bias**	**Inconsistency**	**Indirectness**	**Imprecision**	**Best (median) estimate across all risk strata (%)***	**Best (median) estimate by patient risk strata (%)†**	**Evidence certainty‡**
**Non-fatal symptomatic venous thromboembolism**							
6,292 (1)	No serious limitations	No serious limitations	No serious limitations	No serious limitations	0.65	**Low: 0.51 Medium: 1.01 High: 2.03**	**Moderate**
**Fatal venous thromboembolism**							
6,292 (1)	No serious limitations	No serious limitations	No serious limitations	No serious limitations	0.02	**Low: 0.02 Medium: 0.04 High: 0.08**	**Low**
**Symptomatic splanchnic vein thrombosis**							
**Non-fatal bleeding requiring reintervention**							
**Non-fatal bleeding leading to transfusion**							
**Fatal bleeding**							
**Bleeding leading to hemoglobin below 70g/L (7g/dL)**							

Blank spaces indicate absence of information

* Estimate represents absolute risk in percent. Our median best estimates include fatal and non-fatal events. Based on data from included studies, we estimated case fatality rates as follows: 3.6% for VTE, 3.6% for bleeding leading to reintervention, and 0.9% for bleeding leading to transfusion, and used this information to calculate outcome estimates. For instance, we multiplied the median VTE estimate by 0.964 for non-fatal VTE and by 0.036 for fatal VTE (if both reintervention and transfusion rates were available, we preferred reintervention estimates for calculation of fatal bleeding estimate).

† Risk factors included 1) age more than 75 years, 2) obesity (body mass index of 35 or more), 3) VTE in a first degree relative (parents, full siblings, or children), and 4) prior VTE. We assumed that patients with any combination of two or more risk factors had a risk ratio of 4. Using these risk factors, we then categorized risk of VTE as low, medium, and high risk.

‡ Options for certainty in estimates are high, moderate, low, and very low. Evidence begins as high and is rated down for serious risk of bias, inconsistency, imprecision, or indirectness. We always rated down once due to uncertainty in the patient VTE risk factors and models of timing of VTE and bleeding. For fatal VTE and fatal bleeding we always rated down once for uncertainty in our case fatality rate estimates.

5. Evidence profile 5. Cholecystectomy, laparoscopic: Absolute risk of venous thromboembolism and bleeding among patients not receiving prophylaxis

Certainty assessment					Summary of findings		
**No of participants (studies)**	**Risk of Bias**	**Inconsistency**	**Indirectness**	**Imprecision**	**Best (median) estimate across all risk strata (%)***	**Best (median) estimate by patient risk strata (%)†**	**Evidence certainty‡**
**Non-fatal symptomatic venous thromboembolism**							
4,698,705 (17)	Serious limitations	No serious limitations	No serious limitations	No serious limitations	0.03	**Low: 0.02 Medium: 0.05 High: 0.10**	**Moderate§**
**Fatal venous thromboembolism**							
4,698,705 (17)	Serious limitations	No serious limitations	No serious limitations	No serious limitations	0.00	**Low: 0 Medium: 0 High: 0**	**Low§**
**Symptomatic splanchnic vein thrombosis**							
1,575 (2)	Serious limitations	No serious limitations	No serious limitations	No serious limitations	0.00	**0.00**	**Low**
**Non-fatal bleeding requiring reintervention**							
10,959 (9)	Serious limitations	No serious limitations	No serious limitations	No serious limitations	0.24	**0.24**	**Low**
**Non-fatal bleeding leading to transfusion**							
120,689 (6)	Serious limitations	No serious limitations	No serious limitations	No serious limitations	0.09	**0.09**	**Low**
**Fatal bleeding**							
10,959 (9)	Serious limitations	No serious limitations	No serious limitations	No serious limitations	0.01	**0.01**	**Very Low**
**Bleeding leading to hemoglobin below 70g/L (7g/dL)**							
90 (1)	No serious limitations	No serious limitations	No serious limitations	Very serious limitations	0.00	**0.00**	**Very low**

* Estimate represents absolute risk in percent. Our median best estimates include fatal and non-fatal events. Based on data from included studies, we estimated case fatality rates as follows: 3.6% for VTE, 3.6% for bleeding leading to reintervention, and 0.9% for bleeding leading to transfusion, and used this information to calculate outcome estimates. For instance, we multiplied the median VTE estimate by 0.964 for non-fatal VTE and by 0.036 for fatal VTE (if both reintervention and transfusion rates were available, we preferred reintervention estimates for calculation of fatal bleeding estimate).

† Risk factors included 1) age more than 75 years, 2) obesity (body mass index of 35 or more), 3) VTE in a first degree relative (parents, full siblings, or children), and 4) prior VTE. We assumed that patients with any combination of two or more risk factors had a risk ratio of 4. Using these risk factors, we then categorized risk of VTE as low, medium, and high risk.

‡ Options for evidence certainty were high, moderate, low, and very low. Evidence began as high and was rated down for serious risk of bias, inconsistency, imprecision, or indirectness. We always rated down once due to uncertainty in the patient VTE risk factors and models of timing of VTE and bleeding. For fatal VTE and fatal bleeding we always rated down once for uncertainty in our case fatality rate estimates.

§ If non-fatal VTE risk was less than 0.1%, we upgraded evidence certainty from low to moderate because even if absolute risk of VTE would have been multiplied by 5 times, it would be less than 0.5%, and would therefore unlikely change thromboprophylaxis decisions.

6. Evidence profile 6. Cholecystectomy, open: Absolute risk of venous thromboembolism and bleeding among patients not receiving prophylaxis

Certainty assessment					Summary of findings		
**No of participants (studies)**	**Risk of Bias**	**Inconsistency**	**Indirectness**	**Imprecision**	**Best (median) estimate across all risk strata (%)***	**Best (median) estimate by patient risk strata (%)†**	**Evidence certainty‡**
**Non-fatal symptomatic venous thromboembolism**							
64,493 (5)	No serious limitations	No serious limitations	No serious limitations	No serious limitations	1.23	**Low: 0.91 Medium: 1.81 High: 3.62**	**Moderate**
**Fatal venous thromboembolism**							
64,493 (5)	No serious limitations	No serious limitations	No serious limitations	No serious limitations	0.05	**Low: 0.03 Medium: 0.07 High: 0.14**	**Low**
**Symptomatic splanchnic vein thrombosis**							
**Non-fatal bleeding requiring reintervention¶**							
10,959 (9)	Serious limitations	No serious limitations	Very serious limitations	No serious limitations	0.40	**0.40**	**Very low**
**Non-fatal bleeding leading to transfusion**							
16,164 (3)	Serious limitations	Serious limitations	No serious limitations	No serious limitations	0.66	**0.66**	**Very low**
**Fatal bleeding**							
16,164 (3)	Serious limitations	Serious limitations	No serious limitations	No serious limitations	0.00	**0.00**	**Very low**
**Bleeding leading to hemoglobin below 70g/L (7g/dL)**							

Blank spaces indicate absence of information

* Estimate represents absolute risk in percent. Our median best estimates include fatal and non-fatal events. Based on data from included studies, we estimated case fatality rates as follows: 3.6% for VTE, 3.6% for bleeding leading to reintervention, and 0.9% for bleeding leading to transfusion, and used this information to calculate outcome estimates. For instance, we multiplied the median VTE estimate by 0.964 for non-fatal VTE and by 0.036 for fatal VTE (if both reintervention and transfusion rates were available, we preferred reintervention estimates for calculation of fatal bleeding estimate).

† Risk factors included 1) age more than 75 years, 2) obesity (body mass index of 35 or more), 3) VTE in a first degree relative (parents, full siblings, or children), and 4) prior VTE. We assumed that patients with any combination of two or more risk factors had a risk ratio of 4. Using these risk factors, we then categorized risk of VTE as low, medium, and high risk.

‡ Options for certainty in estimates are high, moderate, low, and very low. Evidence begins as high and is rated down for serious risk of bias, inconsistency, imprecision, or indirectness. We always rated down once due to uncertainty in the patient VTE risk factors and models of timing of VTE and bleeding. For fatal VTE and fatal bleeding we always rated down once for uncertainty in our case fatality rate estimates.

¶We did not have any studies providing bleeding requiring reintervention estimates for open cholecystectomy. Surgeon expert panel estimated risk to be same or double the risk of laparoscopic cholecystectomy (0.25-0.50%) or half of the risk of open minor liver resection (0.35%). Therefore we used 0.4% and considered this procedure to have very serious limitations due to indirectness.

7. Evidence profile 7. Cholecystectomy, conversion to open: Absolute risk of venous thromboembolism and bleeding among patients not receiving prophylaxis

Quality assessment					Summary of findings		
**No of participants (studies)**	**Risk of Bias**	**Inconsistency**	**Indirectness**	**Imprecision**	**Best (median) estimate across all risk strata (%)***	**Best (median) estimate by patient risk strata (%)†**	**Evidence certainty‡**
**Non-fatal symptomatic venous thromboembolism**							
**Fatal venous thromboembolism**							
**Symptomatic splanchnic vein thrombosis**							
**Non-fatal bleeding requiring reintervention**							
**Non-fatal bleeding leading to transfusion**							
3,768 (1)	Serious limitations	No serious limitations	No serious limitations	No serious limitations	0.00	**0.00**	**Low**
**Fatal bleeding**							
3,768 (1)	Serious limitations	No serious limitations	No serious limitations	No serious limitations	0.00	**0.00**	**Very low**
**Bleeding leading to hemoglobin below 70g/L (7g/dL)**							

Blank spaces indicate absence of information

* Estimate represents absolute risk in percent. Our median best estimates include fatal and non-fatal events. Based on data from included studies, we estimated case fatality rates as follows: 3.6% for VTE, 3.6% for bleeding leading to reintervention, and 0.9% for bleeding leading to transfusion, and used this information to calculate outcome estimates. For instance, we multiplied the median VTE estimate by 0.964 for non-fatal VTE and by 0.036 for fatal VTE (if both reintervention and transfusion rates were available, we preferred reintervention estimates for calculation of fatal bleeding estimate).

† Risk factors included 1) age more than 75 years, 2) obesity (body mass index of 35 or more), 3) VTE in a first degree relative (parents, full siblings, or children), and 4) prior VTE. We assumed that patients with any combination of two or more risk factors had a risk ratio of 4. Using these risk factors, we then categorized risk of VTE as low, medium, and high risk.

‡ Options for certainty in estimates are high, moderate, low, and very low. Evidence begins as high and is rated down for serious risk of bias, inconsistency, imprecision, or indirectness. We always rated down once due to uncertainty in the patient VTE risk factors and models of timing of VTE and bleeding. For fatal VTE and fatal bleeding we always rated down once for uncertainty in our case fatality rate estimates.

8. Evidence profile 8. Cholecystectomy, laparoscopic, elective: Absolute risk of venous thromboembolism and bleeding among patients not receiving prophylaxis

Certainty assessment					Summary of findings		
**No of participants (studies)**	**Risk of Bias**	**Inconsistency**	**Indirectness**	**Imprecision**	**Best (median) estimate across all risk strata (%)***	**Best (median) estimate by patient risk strata (%)†**	**Evidence certainty‡**
**Non-fatal symptomatic venous thromboembolism**							
2,450 (5)	No serious limitations	No serious limitations	No serious limitations	No serious limitations	0.04¶	**Low: 0.03 Medium: 0.06 High: 0.12**	**High**
**Fatal venous thromboembolism**							
2,450 (5)	No serious limitations	No serious limitations	No serious limitations	No serious limitations	0.00	**Low: 0.00 Medium: 0.00 High: 0.00**	**Moderate**
**Symptomatic splanchnic vein thrombosis**							
1,575 (2)	Serious limitations	No serious limitations	No serious limitations	No serious limitations	0.00	**0.00**	**Low**
**Non-fatal bleeding requiring reintervention**							
1,739 (3)	No serious limitations	No serious limitations	No serious limitations	No serious limitations	0.14	**0.14**	**Moderate**
**Non-fatal bleeding leading to transfusion**							
1,575 (2)	No serious limitations	No serious limitations	No serious limitations	No serious limitations	0.00	**0.00**	**Moderate**
**Fatal bleeding**							
1,739 (3)	No serious limitations	No serious limitations	No serious limitations	No serious limitations	0.01	**0.01**	**Low**
**Bleeding leading to hemoglobin below 70g/L (7g/dL)**							
90 (1)	No serious limitations	No serious limitations	No serious limitations	Very serious limitations	0.00	**0.00**	**Very low**

Blank spaces indicate absence of information
For non-fatal VTE risk we upgraded evidence certainty from moderate to high, as absolute risk of VTE would be less than 0.5% even if our best estimate would be multiplied 5 times, and would therefore not change thromboprophylaxis decisions.
* Estimate represents absolute risk in percent. Our median best estimates include fatal and non-fatal events. Based on data from included studies, we estimated case fatality rates as follows: 3.6% for VTE, 3.6% for bleeding leading to reintervention, and 0.9% for bleeding leading to transfusion, and used this information to calculate outcome estimates. For instance, we multiplied the median VTE estimate by 0.964 for non-fatal VTE and by 0.036 for fatal VTE (if both reintervention and transfusion rates were available, we preferred reintervention estimates for calculation of fatal bleeding estimate).
† Risk factors included 1) age more than 75 years, 2) obesity (body mass index of 35 or more), 3) VTE in a first degree relative (parents, full siblings, or children), and 4) prior VTE. We assumed that patients with any combination of two or more risk factors had a risk ratio of 4. Using these risk factors, we then categorized risk of VTE as low, medium, and high risk.
‡ Options for certainty in estimates are high, moderate, low, and very low. Evidence begins as high and is rated down for serious risk of bias, inconsistency, imprecision, or indirectness. We always rated down once due to uncertainty in the patient VTE risk factors and models of timing of VTE and bleeding. For fatal VTE and fatal bleeding we always rated down once for uncertainty in our case fatality rate estimates. ¶Reported median estimate was 0.0%. As real underlying risk of 0.0% is improbable we used average instead of median.

9. Evidence profile 9. Cholecystectomy, laparoscopic, emergency: Absolute risk of venous thromboembolism and bleeding among patients not receiving prophylaxis

Certainty assessment					Summary of findings		
**No of participants (studies)**	**Risk of Bias**	**Inconsistency**	**Indirectness**	**Imprecision**	**Best (median) estimate across all risk strata (%)***	**Best (median) estimate by patient risk strata (%)†**	**Evidence certainty‡**
**Non-fatal symptomatic venous thromboembolism**							
11,266 (1)	No serious limitations	No serious limitations	No serious limitations	No serious limitations	0.33	**Low: 0.26 Medium: 0.52 High: 1.04**	**Moderate**
**Fatal venous thromboembolism**							
11,266 (1)	No serious limitations	No serious limitations	No serious limitations	No serious limitations	0.01	**Low: 0.01 Medium: 0.02 High: 0.04**	**Low**
**Symptomatic splanchnic vein thrombosis**							
**Non-fatal bleeding requiring reintervention§**							
10,959 (9)	Serious limitations	No serious limitations	Very serious limitations	No serious limitations	0.43	**0.43**	**Very low**
**Non-fatal bleeding leading to transfusion**							
**Fatal bleeding**							
10,959 (9)	Serious limitations	No serious limitations	Very serious limitations	No serious limitations	0.02	**0.02**	**Very low**
**Bleeding leading to hemoglobin below 70g/L (7g/dL)**							

Blank spaces indicate absence of information

Systematic Reviews and Meta-Analyses of the Procedure-Specific Risks of Thrombosis and Bleeding in Surgery: Upper-Gastrointestinal and Hepatopancreatobiliary Surgery † Risk factors included 1) age more than 75 years, 2) obesity (body mass index of 35 or more), 3) VTE in a first degree relative (parents, full siblings, or children), and 4) prior VTE. We assumed that patients with any combination of two or more risk factors had a risk ratio of 4. Using these risk factors, we then categorized risk of VTE as low, medium, and high risk.

‡ Options for certainty in estimates are high, moderate, low, and very low. Evidence begins as high and is rated down for serious risk of bias, inconsistency, imprecision, or indirectness. We always rated down once due to uncertainty in the patient VTE risk factors and models of timing of VTE and bleeding. For fatal VTE and fatal bleeding we always rated down once for uncertainty in our case fatality rate estimates.

§ We calculated the risk for bleeding leading to reintervention with information from Persson et al. 2012 that OR of bleeding in emergency vs. elective cholecystectomy is 2.0. We know that risk is 0.25% for both elective and emergent combined. We also estimated based on information from included studies that 17% of procedures in this total estimate were emergent. Therefore: 0.83X+0.17*y=0.25. When we solve for x, we arrive in estimate of 0.43%. We considered this estimate to have very serious limitations because of indirectness.

10. Evidence profile 10. Cholecystectomy, open, emergency: Absolute risk of venous thromboembolism and bleeding among patients not receiving prophylaxis

Certainty assessment					Summary of findings		
**No of participants (studies)**	**Risk of Bias**	**Inconsistency**	**Indirectness**	**Imprecision**	**Best (median) estimate across all risk strata (%)***	**Best (median) estimate by patient risk strata (%)†**	**Evidence certainty‡**
**Non-fatal symptomatic venous thromboembolism**							
1,447 (1)	No serious limitations	No serious limitations	No serious limitations	No serious limitations	1.60	**Low: 1.25 Medium: 2.50 High: 5.00**	**Moderate**
**Fatal venous thromboembolism**							
1,447 (1)	No serious limitations	No serious limitations	No serious limitations	No serious limitations	0.06	**Low: 0.05 Medium: 0.09 High: 0.19**	**Low**
**Symptomatic splanchnic vein thrombosis**							
**Non-fatal bleeding requiring reintervention**							
**Non-fatal bleeding leading to transfusion**							
**Fatal bleeding**							
**Bleeding leading to hemoglobin below 70g/L (7g/dL)**							

Blank spaces indicate absence of information

* Estimate represents absolute risk in percent. Our median best estimates include fatal and non-fatal events. Based on data from included studies, we estimated case fatality rates as follows: 3.6% for VTE, 3.6% for bleeding leading to reintervention, and 0.9% for bleeding leading to transfusion, and used this information to calculate outcome estimates. For instance, we multiplied the median VTE estimate by 0.964 for non-fatal VTE and by 0.036 for fatal VTE (if both reintervention and transfusion rates were available, we preferred reintervention estimates for calculation of fatal bleeding estimate).

† Risk factors included 1) age more than 75 years, 2) obesity (body mass index of 35 or more), 3) VTE in a first degree relative (parents, full siblings, or children), and 4) prior VTE. We assumed that patients with any combination of two or more risk factors had a risk ratio of 4. Using these risk factors, we then categorized risk of VTE as low, medium, and high risk.

‡ Options for certainty in estimates are high, moderate, low, and very low. Evidence begins as high and is rated down for serious risk of bias, inconsistency, imprecision, or indirectness. We always rated down once due to uncertainty in the patient VTE risk factors and models of timing of VTE and bleeding. For fatal VTE and fatal bleeding we always rated down once for uncertainty in our case fatality rate estimates.

11. Evidence profile 11. Groin hernia repair, laparoscopic: Absolute risk of venous thromboembolism and bleeding among patients not receiving prophylaxis

Certainty assessment					Summary of findings		
**No of participants (studies)**	**Risk of Bias**	**Inconsistency**	**Indirectness**	**Imprecision**	**Best (median) estimate across all risk strata (%)***	**Best (median) estimate by patient risk strata (%)†**	**Evidence certainty‡**
**Non-fatal symptomatic venous thromboembolism**							
13,333 (6)	Serious limitations	No serious limitations	No serious limitations	No serious limitations	0.57	**Low: 0.37 Medium: 0.74 High: 1.49**	**Low**
**Fatal venous thromboembolism**							
13,333 (6)	Serious limitations	No serious limitations	No serious limitations	No serious limitations	0.02	**Low: 0.01 Medium: 0.03 High: 0.06**	**Very Low**
**Symptomatic splanchnic vein thrombosis**							
82 (1)	Serious limitations	No serious limitations	No serious limitations	Very serious limitations	0.00	**0.00**	**Very low**
**Non-fatal bleeding requiring reintervention**							
5,086 (3)	Serious limitations	No serious limitations	No serious limitations	No serious limitations	0.21	**0.21**	**Low**
**Non-fatal bleeding leading to transfusion**							
413 (1)	No serious limitations	No serious limitations	No serious limitations	Serious limitations	0.00	**0.00**	**Low**
**Fatal bleeding**							
5,086 (3)	Serious limitations	No serious limitations	No serious limitations	No serious limitations	0.01	**0.01**	**Very Low**
**Bleeding leading to hemoglobin below 70g/L (7g/dL)**							

Blank spaces indicate absence of information

* Estimate represents absolute risk in percent. Our median best estimates include fatal and non-fatal events. Based on data from included studies, we estimated case fatality rates as follows: 3.6% for VTE, 3.6% for bleeding leading to reintervention, and 0.9% for bleeding leading to transfusion, and used this information to calculate outcome estimates. For instance, we multiplied the median VTE estimate by 0.964 for non-fatal VTE and by 0.036 for fatal VTE (if both reintervention and transfusion rates were available, we preferred reintervention estimates for calculation of fatal bleeding estimate).

† Risk factors included 1) age more than 75 years, 2) obesity (body mass index of 35 or more), 3) VTE in a first degree relative (parents, full siblings, or children), and 4) prior VTE. We assumed that patients with any combination of two or more risk factors had a risk ratio of 4. Using these risk factors, we then categorized risk of VTE as low, medium, and high risk.

‡ Options for certainty in estimates are high, moderate, low, and very low. Evidence begins as high and is rated down for serious risk of bias, inconsistency, imprecision, or indirectness. We always rated down once due to uncertainty in the patient VTE risk factors and models of timing of VTE and bleeding. For fatal VTE and fatal bleeding we always rated down once for uncertainty in our case fatality rate estimates.

12. Evidence profile 12. Groin hernia repair, open: Absolute risk of venous thromboembolism and bleeding among patients not receiving prophylaxis

Certainty assessment					Summary of findings		
**No of participants (studies)**	**Risk of Bias**	**Inconsistency**	**Indirectness**	**Imprecision**	**Best (median) estimate across all risk strata (%)***	**Best (median) estimate by patient risk strata (%)†**	**Evidence certainty‡**
**Non-fatal symptomatic venous thromboembolism**							
189,943 (9)	No serious limitations	Serious limitations	No serious limitations	No serious limitations	0.19	**Low: 0.13 Medium: 0.26 High: 0.53**	**Low**
**Fatal venous thromboembolism**							
189,943 (9)	No serious limitations	Serious limitations	No serious limitations	No serious limitations	0.01	**Low: 0.00 Medium: 0.01 High: 0.02**	**Very Low**
**Symptomatic splanchnic vein thrombosis**							
5,004 (2)	Serious limitations	No serious limitations	No serious limitations	No serious limitations	0.00	**0.00**	**Low**
**Non-fatal bleeding requiring reintervention**							
5,222 (3)	No serious limitations	No serious limitations	No serious limitations	No serious limitations	0.08	**0.08**	**Moderate**
**Non-fatal bleeding leading to transfusion**							
4,870 (1)	No serious limitations	No serious limitations	No serious limitations	No serious limitations	0.02	**0.02**	**Moderate**
**Fatal bleeding**							
5,222 (3)	No serious limitations	No serious limitations	No serious limitations	No serious limitations	0.00	**0.00**	**Low**
**Bleeding leading to hemoglobin below 70g/L (7g/dL)**							

Blank spaces indicate absence of information

* Estimate represents absolute risk in percent. Our median best estimates include fatal and non-fatal events. Based on data from included studies, we estimated case fatality rates as follows: 3.6% for VTE, 3.6% for bleeding leading to reintervention, and 0.9% for bleeding leading to transfusion, and used this information to calculate outcome estimates. For instance, we multiplied the median VTE estimate by 0.964 for non-fatal VTE and by 0.036 for fatal VTE (if both reintervention and transfusion rates were available, we preferred reintervention estimates for calculation of fatal bleeding estimate).

† Risk factors included 1) age more than 75 years, 2) obesity (body mass index of 35 or more), 3) VTE in a first degree relative (parents, full siblings, or children), and 4) prior VTE. We assumed that patients with any combination of two or more risk factors had a risk ratio of 4. Using these risk factors, we then categorized risk of VTE as low, medium, and high risk.

‡ Options for certainty in estimates are high, moderate, low, and very low. Evidence begins as high and is rated down for serious risk of bias, inconsistency, imprecision, or indirectness. We always rated down once due to uncertainty in the patient VTE risk factors and models of timing of VTE and bleeding. For fatal VTE and fatal bleeding we always rated down once for uncertainty in our case fatality rate estimates.

13. Evidence profile 13. Groin hernia repair, laparoscopic, elective: Absolute risk of venous thromboembolism and bleeding among patients not receiving prophylaxis

Certainty assessment					Summary of findings		
**No of participants (studies)**	**Risk of Bias**	**Inconsistency**	**Indirectness**	**Imprecision**	**Best (median) estimate across all risk strata (%)***	**Best (median) estimate by patient risk strata (%)†**	**Evidence certainty‡**
**Non-fatal symptomatic venous thromboembolism**							
226 (2)	No serious limitations	No serious limitations	No serious limitations	Serious limitations	0.00	**Low: 0.00 Medium: 0.00 High: 0.00**	**Low**
**Fatal venous thromboembolism**							
226 (2)	No serious limitations	No serious limitations	No serious limitations	Serious limitations	0.00	**Low: 0.00 Medium: 0.00 High: 0.00**	**Very Low**
**Symptomatic splanchnic vein thrombosis**							
82 (1)	Serious limitations	No serious limitations	No serious limitations	Very serious limitations	0.00	**0.00**	**Very low**
**Non-fatal bleeding requiring reintervention**							
4,978 (2)	Serious limitations	No serious limitations	No serious limitations	No serious limitations	0.10	**0.10**	**Low**
**Non-fatal bleeding leading to transfusion**							
413 (1)	No serious limitations	No serious limitations	No serious limitations	Serious limitations	0.00	**0.00**	**Low**
**Fatal bleeding**							
4,978 (2)	Serious limitations	No serious limitations	No serious limitations	No serious limitations	0.00	**0.00**	**Very Low**
**Bleeding leading to hemoglobin below 70g/L (7g/dL)**							

Blank spaces indicate absence of information

* Estimate represents absolute risk in percent. Our median best estimates include fatal and non-fatal events. Based on data from included studies, we estimated case fatality rates as follows: 3.6% for VTE, 3.6% for bleeding leading to reintervention, and 0.9% for bleeding leading to transfusion, and used this information to calculate outcome estimates. For instance, we multiplied the median VTE estimate by 0.964 for non-fatal VTE and by 0.036 for fatal VTE (if both reintervention and transfusion rates were available, we preferred reintervention estimates for calculation of fatal bleeding estimate).

† Risk factors included 1) age more than 75 years, 2) obesity (body mass index of 35 or more), 3) VTE in a first degree relative (parents, full siblings, or children), and 4) prior VTE. We assumed that patients with any combination of two or more risk factors had a risk ratio of 4. Using these risk factors, we then categorized risk of VTE as low, medium, and high risk.

‡ Options for certainty in estimates are high, moderate, low, and very low. Evidence begins as high and is rated down for serious risk of bias, inconsistency, imprecision, or indirectness. We always rated down once due to uncertainty in the patient VTE risk factors and models of timing of VTE and bleeding. For fatal VTE and fatal bleeding we always rated down once for uncertainty in our case fatality rate estimates.

14. Evidence profile 14. Groin hernia repair, open, elective: Absolute risk of venous thromboembolism and bleeding among patients not receiving prophylaxis

Quality assessment					Summary of findings		
**No of participants (studies)**	**Risk of Bias**	**Inconsistency**	**Indirectness**	**Imprecision**	**Best (median) estimate across all risk strata (%)***	**Best (median) estimate by patient risk strata (%)†**	**Evidence certainty‡**
**Non-fatal symptomatic venous thromboembolism**							
133,019 (2)	No serious limitations	No serious limitations	No serious limitations	No serious limitations	0.07	**Low: 0.05 Medium: 0.09 High: 0.19**	**Moderate**
**Fatal venous thromboembolism**							
133,019 (2)	No serious limitations	No serious limitations	No serious limitations	No serious limitations	0.00	**Low: 0 Medium: 0 High: 0.01**	**Low**
**Symptomatic splanchnic vein thrombosis**							
134 (1)	Serious limitations	No serious limitations	No serious limitations	Very serious limitations	0.00	**0.00**	**Very low**
**Non-fatal bleeding requiring reintervention**							
352 (2)	No serious limitations	No serious limitations	No serious limitations	Serious limitations	0.25	**0.25**	**Low**
**Non-fatal bleeding leading to transfusion**							
**Fatal bleeding**							
352 (2)	No serious limitations	No serious limitations	No serious limitations	Serious limitations	0.01	**0.01**	**Very Low**
**Bleeding leading to hemoglobin below 70g/L (7g/dL)**							

Blank spaces indicate absence of information

* Estimate represents absolute risk in percent. Our median best estimates include fatal and non-fatal events. Based on data from included studies, we estimated case fatality rates as follows: 3.6% for VTE, 3.6% for bleeding leading to reintervention, and 0.9% for bleeding leading to transfusion, and used this information to calculate outcome estimates. For instance, we multiplied the median VTE estimate by 0.964 for non-fatal VTE and by 0.036 for fatal VTE (if both reintervention and transfusion rates were available, we preferred reintervention estimates for calculation of fatal bleeding estimate).

† Risk factors included 1) age more than 75 years, 2) obesity (body mass index of 35 or more), 3) VTE in a first degree relative (parents, full siblings, or children), and 4) prior VTE. We assumed that patients with any combination of two or more risk factors had a risk ratio of 4. Using these risk factors, we then categorized risk of VTE as low, medium, and high risk.

‡ Options for certainty in estimates are high, moderate, low, and very low. Evidence begins as high and is rated down for serious risk of bias, inconsistency, imprecision, or indirectness. We always rated down once due to uncertainty in the patient VTE risk factors and models of timing of VTE and bleeding. For fatal VTE and fatal bleeding we always rated down once for uncertainty in our case fatality rate estimates.

15. Evidence profile 15. Groin hernia repair, open, emergency: Absolute risk of venous thromboembolism and bleeding among patients not receiving prophylaxis

Certainty assessment					Summary of findings		
**No of participants (studies)**	**Risk of Bias**	**Inconsistency**	**Indirectness**	**Imprecision**	**Best (median) estimate across all risk strata (%)***	**Best (median) estimate by patient risk strata (%)†**	**Evidence certainty‡**
**Non-fatal symptomatic venous thromboembolism**							
8,403 (4)	No serious limitations	No serious limitations	No serious limitations	No serious limitations	1.39	**Low: 0.77 Medium: 1.54 High: 3.09**	**Moderate**
**Fatal venous thromboembolism**							
8,403 (4)	No serious limitations	No serious limitations	No serious limitations	No serious limitations	0.05	**Low: 0.03 Medium: 0.06 High: 0.12**	**Low**
**Symptomatic splanchnic vein thrombosis**							
**Non-fatal bleeding requiring reintervention**							
146 (1)	Serious limitations	No serious limitations	No serious limitations	Very serious limitations	0.00	**0.00**	**Very low**
**Non-fatal bleeding leading to transfusion**							
**Fatal bleeding**							
146 (1)	Serious limitations	No serious limitations	No serious limitations	Very serious limitations	0.00	**0.00**	**Very low**
**Bleeding leading to hemoglobin below 70g/L (7g/dL)**							

Blank spaces indicate absence of information

* Estimate represents absolute risk in percent. Our median best estimates include fatal and non-fatal events. Based on data from included studies, we estimated case fatality rates as follows: 3.6% for VTE, 3.6% for bleeding leading to reintervention, and 0.9% for bleeding leading to transfusion, and used this information to calculate outcome estimates. For instance, we multiplied the median VTE estimate by 0.964 for non-fatal VTE and by 0.036 for fatal VTE (if both reintervention and transfusion rates were available, we preferred reintervention estimates for calculation of fatal bleeding estimate).

† Risk factors included 1) age more than 75 years, 2) obesity (body mass index of 35 or more), 3) VTE in a first degree relative (parents, full siblings, or children), and 4) prior VTE. We assumed that patients with any combination of two or more risk factors had a risk ratio of 4. Using these risk factors, we then categorized risk of VTE as low, medium, and high risk.

‡ Options for certainty in estimates are high, moderate, low, and very low. Evidence begins as high and is rated down for serious risk of bias, inconsistency, imprecision, or indirectness. We always rated down once due to uncertainty in the patient VTE risk factors and models of timing of VTE and bleeding. For fatal VTE and fatal bleeding we always rated down once for uncertainty in our case fatality rate estimates.

16. Evidence profile 16. Ventral hernia repair, minimally-invasive: Absolute risk of venous thromboembolism and bleeding among patients not receiving prophylaxis

Quality assessment					Summary of findings		
**No of participants (studies)**	**Risk of Bias**	**Inconsistency**	**Indirectness**	**Imprecision**	**Best (median) estimate across all risk strata (%)***	**Best (median) estimate by patient risk strata (%)†**	**Evidence certainty‡**
**Non-fatal symptomatic venous thromboembolism**							
35,364 (5)	No serious limitations	Serious limitations	No serious limitations	No serious limitations	0.39	**Low: 0.29 Medium: 0.59 High: 1.17**	**Low**
**Fatal venous thromboembolism**							
35,364 (5)	No serious limitations	Serious limitations	No serious limitations	No serious limitations	0.01	**Low: 0.01 Medium: 0.02 High: 0.04**	**Very Low**
**Symptomatic splanchnic vein thrombosis**							
**Non-fatal bleeding requiring reoperation**							
517 (2)	Serious limitations	No serious limitations	No serious limitations	Serious limitations	0.11	**0.11**	**Very low**
**Non-fatal bleeding leading to transfusion**							
26,286 (1)	No serious limitations	No serious limitations	No serious limitations	No serious limitations	0.05	**0.05**	**Moderate**
**Fatal bleeding**							
517 (2)	Serious limitations	No serious limitations	No serious limitations	Serious limitations	0.00	**0.00**	**Very low**
**Bleeding leading to hemoglobin below 70g/L (7g/dL)**							

Blank spaces indicate absence of information
Minimally-invasive includes laparoscopic or robotic.
* Estimate represents absolute risk in percent. Our median best estimates include fatal and non-fatal events. Based on data from included studies, we estimated case fatality rates as follows: 3.6% for VTE, 3.6% for bleeding leading to reintervention, and 0.9% for bleeding leading to transfusion, and used this information to calculate outcome estimates. For instance, we multiplied the median VTE estimate by 0.964 for non-fatal VTE and by 0.036 for fatal VTE (if both reintervention and transfusion rates were available, we preferred reintervention estimates for calculation of fatal bleeding estimate).
† Risk factors included 1) age more than 75 years, 2) obesity (body mass index of 35 or more), 3) VTE in a first degree relative (parents, full siblings, or children), and 4) prior VTE. We assumed that patients with any combination of two or more risk factors had a risk ratio of 4. Using these risk factors, we then categorized risk of VTE as low, medium, and high risk.
‡ Options for certainty in estimates are high, moderate, low, and very low. Evidence begins as high and is rated down for serious risk of bias, inconsistency, imprecision, or indirectness. We always rated down once due to uncertainty in the patient VTE risk factors and models of timing of VTE and bleeding. For fatal VTE and fatal bleeding we always rated down once for uncertainty in our case fatality rate estimates.

17. Evidence profile 17. Ventral hernia repair, laparoscopic: Absolute risk of venous thromboembolism and bleeding among patients not receiving prophylaxis

Certainty assessment					Summary of findings		
**No of participants (studies)**	**Risk of Bias**	**Inconsistency**	**Indirectness**	**Imprecision**	**Best (median) estimate across all risk strata (%)***	**Best (median) estimate by patient risk strata (%)†**	**Evidence certainty‡**
**Non-fatal symptomatic venous thromboembolism**							
35,364 (5)	No serious limitations	Serious limitations	No serious limitations	No serious limitations	0.39	**Low: 0.29 Medium: 0.59 High: 1.17**	**Low**
**Fatal venous thromboembolism**							
35,364 (5)	No serious limitations	Serious limitations	No serious limitations	No serious limitations	0.01	**Low: 0.01 Medium: 0.02 High: 0.04**	**Very Low**
**Symptomatic splanchnic vein thrombosis**							
**Non-fatal bleeding requiring reintervention**							
464 (2)	Serious limitations	No serious limitations	No serious limitations	Serious limitations	0.11	**0.11**	**Very low**
**Non-fatal bleeding leading to transfusion**							
26,286 (1)	No serious limitations	No serious limitations	No serious limitations	No serious limitations	0.05	**0.05**	**Moderate**
**Fatal bleeding**							
464 (2)	Serious limitations	No serious limitations	No serious limitations	Serious limitations	0.00	**0.00**	**Very low**
**Bleeding leading to hemoglobin below 70g/L (7g/dL)**							

Blank spaces indicate absence of information

* Estimate represents absolute risk in percent. Our median best estimates include fatal and non-fatal events. Based on data from included studies, we estimated case fatality rates as follows: 3.6% for VTE, 3.6% for bleeding leading to reintervention, and 0.9% for bleeding leading to transfusion, and used this information to calculate outcome estimates. For instance, we multiplied the median VTE estimate by 0.964 for non-fatal VTE and by 0.036 for fatal VTE (if both reintervention and transfusion rates were available, we preferred reintervention estimates for calculation of fatal bleeding estimate).

† Risk factors included 1) age more than 75 years, 2) obesity (body mass index of 35 or more), 3) VTE in a first degree relative (parents, full siblings, or children), and 4) prior VTE. We assumed that patients with any combination of two or more risk factors had a risk ratio of 4. Using these risk factors, we then categorized risk of VTE as low, medium, and high risk.

‡ Options for certainty in estimates are high, moderate, low, and very low. Evidence begins as high and is rated down for serious risk of bias, inconsistency, imprecision, or indirectness. We always rated down once due to uncertainty in the patient VTE risk factors and models of timing of VTE and bleeding. For fatal VTE and fatal bleeding we always rated down once for uncertainty in our case fatality rate estimates.

18. Evidence profile 18. Ventral hernia repair, robotic: Absolute risk of venous thromboembolism and bleeding among patients not receiving prophylaxis

Certainty assessment					Summary of findings		
**No of participants (studies)**	**Risk of Bias**	**Inconsistency**	**Indirectness**	**Imprecision**	**Best (median) estimate across all risk strata (%)***	**Best (median) estimate by patient risk strata (%)†**	**Evidence certainty‡**
**Non-fatal symptomatic venous thromboembolism**							
**Fatal venous thromboembolism**							
**Symptomatic splanchnic vein thrombosis**							
**Non-fatal bleeding requiring reintervention**							
53 (1)	No serious limitations	No serious limitations	No serious limitations	Very serious limitations	0.00	**0.00**	**Very low**
**Non-fatal bleeding leading to transfusion**							
**Fatal bleeding**							
53 (1)	No serious limitations	No serious limitations	No serious limitations	Very serious limitations	0.00	**0.00**	**Very low**
**Bleeding leading to hemoglobin below 70g/L (7g/dL)**							

Blank spaces indicate absence of information

* Estimate represents absolute risk in percent. Our median best estimates include fatal and non-fatal events. Based on data from included studies, we estimated case fatality rates as follows: 3.6% for VTE, 3.6% for bleeding leading to reintervention, and 0.9% for bleeding leading to transfusion, and used this information to calculate outcome estimates. For instance, we multiplied the median VTE estimate by 0.964 for non-fatal VTE and by 0.036 for fatal VTE (if both reintervention and transfusion rates were available, we preferred reintervention estimates for calculation of fatal bleeding estimate).

† Risk factors included 1) age more than 75 years, 2) obesity (body mass index of 35 or more), 3) VTE in a first degree relative (parents, full siblings, or children), and 4) prior VTE. We assumed that patients with any combination of two or more risk factors had a risk ratio of 4. Using these risk factors, we then categorized risk of VTE as low, medium, and high risk.

‡ Options for certainty in estimates are high, moderate, low, and very low. Evidence begins as high and is rated down for serious risk of bias, inconsistency, imprecision, or indirectness. We always rated down once due to uncertainty in the patient VTE risk factors and models of timing of VTE and bleeding. For fatal VTE and fatal bleeding we always rated down once for uncertainty in our case fatality rate estimates.

19. Evidence profile 19. Ventral hernia repair, open: Absolute risk of venous thromboembolism and bleeding among patients not receiving prophylaxis

Certainty assessment					Summary of findings		
**No of participants (studies)**	**Risk of Bias**	**Inconsistency**	**Indirectness**	**Imprecision**	**Best (median) estimate across all risk strata (%)***	**Best (median) estimate by patient risk strata (%)†**	**Evidence certainty‡**
**Non-fatal symptomatic venous thromboembolism**							
133,803 (6)	No serious limitations	Serious limitations	No serious limitations	No serious limitations	1.22	**Low: 0.92 Medium: 1.84 High: 3.68**	**Low**
**Fatal venous thromboembolism**							
133,803 (6)	No serious limitations	Serious limitations	No serious limitations	No serious limitations	0.05	**Low: 0.03 Medium: 0.07 High: 0.14**	**Very Low**
**Symptomatic splanchnic vein thrombosis**							
126 (1)	Serious limitations	No serious limitations	No serious limitations	Very serious limitations	0.00	**0.00**	**Very low**
**Non-fatal bleeding requiring reintervention**							
618 (4)	No serious limitations	No serious limitations	No serious limitations	Serious limitations	0.96	**0.96**	**Low**
**Non-fatal bleeding leading to transfusion**							
90,721 (1)	No serious limitations	No serious limitations	No serious limitations	No serious limitations	0.08	**0.08**	**Moderate**
**Fatal bleeding**							
618 (4)	No serious limitations	No serious limitations	No serious limitations	Serious limitations	0.04	**0.04**	**Very Low**
**Bleeding leading to hemoglobin below 70g/L (7g/dL)**							

Blank spaces indicate absence of information

As we identified five or more articles at low risk of bias with a total of 1,000 or more patients, we excluded moderate and high risk of bias articles from non-fatal symptomatic venous thromboembolism estimate

* Estimate represents absolute risk in percent. Our median best estimates include fatal and non-fatal events. Based on data from included studies, we estimated case fatality rates as follows: 3.6% for VTE, 3.6% for bleeding leading to reintervention, and 0.9% for bleeding leading to transfusion, and used this information to calculate outcome estimates. For instance, we multiplied the median VTE estimate by 0.964 for non-fatal VTE and by 0.036 for fatal VTE (if both reintervention and transfusion rates were available, we preferred reintervention estimates for calculation of fatal bleeding estimate).

† Risk factors included 1) age more than 75 years, 2) obesity (body mass index of 35 or more), 3) VTE in a first degree relative (parents, full siblings, or children), and 4) prior VTE. We assumed that patients with any combination of two or more risk factors had a risk ratio of 4. Using these risk factors, we then categorized risk of VTE as low, medium, and high risk.

‡ Options for certainty in estimates are high, moderate, low, and very low. Evidence begins as high and is rated down for serious risk of bias, inconsistency, imprecision, or indirectness. We always rated down once due to uncertainty in the patient VTE risk factors and models of timing of VTE and bleeding. For fatal VTE and fatal bleeding we always rated down once for uncertainty in our case fatality rate estimates.

20. Evidence profile 20. Ventral hernia repair, laparoscopic, elective: Absolute risk of venous thromboembolism and bleeding among patients not receiving prophylaxis

Certainty assessment					Summary of findings		
**No of participants (studies)**	**Risk of Bias**	**Inconsistency**	**Indirectness**	**Imprecision**	**Best (median) estimate across all risk strata (%)***	**Best (median) estimate by patient risk strata (%)†**	**Evidence certainty‡**
**Non-fatal symptomatic venous thromboembolism**							
26,778 (4)	No serious limitations	Serious limitations	No serious limitations	No serious limitations	0.22	**Low: 0.17 Medium: 0.34 High: 0.67**	**Low**
**Fatal venous thromboembolism**							
26,778 (4)	No serious limitations	Serious limitations	No serious limitations	No serious limitations	0.01	**Low: 0.01 Medium: 0.01 High: 0.03**	**Very Low**
**Symptomatic splanchnic vein thrombosis**							
**Non-fatal bleeding requiring reintervention**							
361 (1)	Serious limitations	No serious limitations	No serious limitations	Serious limitations	0.20	**0.20**	**Very low**
**Non-fatal bleeding leading to transfusion**							
26,286 (1)	No serious limitations	No serious limitations	No serious limitations	No serious limitations	0.05	**0.05**	**Moderate**
**Fatal bleeding**							
361 (1)	Serious limitations	No serious limitations	No serious limitations	Serious limitations	0.01	**0.01**	**Very low**
**Bleeding leading to hemoglobin below 70g/L (7g/dL)**							

Blank spaces indicate absence of information

* Estimate represents absolute risk in percent. Our median best estimates include fatal and non-fatal events. Based on data from included studies, we estimated case fatality rates as follows: 3.6% for VTE, 3.6% for bleeding leading to reintervention, and 0.9% for bleeding leading to transfusion, and used this information to calculate outcome estimates. For instance, we multiplied the median VTE estimate by 0.964 for non-fatal VTE and by 0.036 for fatal VTE (if both reintervention and transfusion rates were available, we preferred reintervention estimates for calculation of fatal bleeding estimate).

† Risk factors included 1) age more than 75 years, 2) obesity (body mass index of 35 or more), 3) VTE in a first degree relative (parents, full siblings, or children), and 4) prior VTE. We assumed that patients with any combination of two or more risk factors had a risk ratio of 4. Using these risk factors, we then categorized risk of VTE as low, medium, and high risk.

‡ Options for certainty in estimates are high, moderate, low, and very low. Evidence begins as high and is rated down for serious risk of bias, inconsistency, imprecision, or indirectness. We always rated down once due to uncertainty in the patient VTE risk factors and models of timing of VTE and bleeding. For fatal VTE and fatal bleeding we always rated down once for uncertainty in our case fatality rate estimates.

21. Evidence profile 21. Ventral hernia repair, laparoscopic, emergency: Absolute risk of venous thromboembolism and bleeding among patients not receiving prophylaxis

Certainty assessment					Summary of findings		
**No of participants (studies)**	**Risk of Bias**	**Inconsistency**	**Indirectness**	**Imprecision**	**Best (median) estimate across all risk strata (%)***	**Best (median) estimate by patient risk strata (%)†**	**Evidence certainty‡**
**Non-fatal symptomatic venous thromboembolism**							
405 (1)	No serious limitations	No serious limitations	No serious limitations	Serious limitations	1.18	**Low: 0.92 Medium: 1.85 High: 3.69**	**Low**
**Fatal venous thromboembolism**							
405 (1)	No serious limitations	No serious limitations	No serious limitations	Serious limitations	0.04	**Low: 0.03 Medium: 0.07 High: 0.14**	**Very Low**
**Symptomatic splanchnic vein thrombosis**							
**Non-fatal bleeding requiring reintervention**							
**Non-fatal bleeding leading to transfusion**							
**Fatal bleeding**							
**Bleeding leading to hemoglobin below 70g/L (7g/dL)**							

Blank spaces indicate absence of information

* Estimate represents absolute risk in percent. Our median best estimates include fatal and non-fatal events. Based on data from included studies, we estimated case fatality rates as follows: 3.6% for VTE, 3.6% for bleeding leading to reintervention, and 0.9% for bleeding leading to transfusion, and used this information to calculate outcome estimates. For instance, we multiplied the median VTE estimate by 0.964 for non-fatal VTE and by 0.036 for fatal VTE (if both reintervention and transfusion rates were available, we preferred reintervention estimates for calculation of fatal bleeding estimate).

† Risk factors included 1) age more than 75 years, 2) obesity (body mass index of 35 or more), 3) VTE in a first degree relative (parents, full siblings, or children), and 4) prior VTE. We assumed that patients with any combination of two or more risk factors had a risk ratio of 4. Using these risk factors, we then categorized risk of VTE as low, medium, and high risk.

‡ Options for certainty in estimates are high, moderate, low, and very low. Evidence begins as high and is rated down for serious risk of bias, inconsistency, imprecision, or indirectness. We always rated down once due to uncertainty in the patient VTE risk factors and models of timing of VTE and bleeding. For fatal VTE and fatal bleeding we always rated down once for uncertainty in our case fatality rate estimates.

22. Evidence profile 22. Ventral hernia repair, open, elective: Absolute risk of venous thromboembolism and bleeding among patients not receiving prophylaxis

**Certainty assessment**					**Summary of findings**		
**No of participants (studies)**	**Risk of Bias**	**Inconsistency**	**Indirectness**	**Imprecision**	**Best (median) estimate across all risk strata (%)***	**Best (median) estimate by patient risk strata (%)†**	**Evidence certainty‡**
**Non-fatal symptomatic venous thromboembolism**							
91,203 (5)	No serious limitations	Serious limitations	No serious limitations	No serious limitations	0.91	**Low: 0.68 Medium: 1.37 High: 2.74**	**Low**
**Fatal venous thromboembolism**							
91,203 (5)	No serious limitations	Serious limitations	No serious limitations	No serious limitations	0.03	**Low: 0.03 Medium: 0.05 High: 0.1**	**Very Low**
**Symptomatic splanchnic vein thrombosis**							
126 (1)	Serious limitations	No serious limitations	No serious limitations	Very serious limitations	0.00	**0.00**	**Very low**
**Non-fatal bleeding requiring reintervention**							
301 (2)	No serious limitations	No serious limitations	No serious limitations	Serious limitations	0.54	**0.54**	**Low**
**Non-fatal bleeding leading to transfusion**							
90,721 (1)	No serious limitations	No serious limitations	No serious limitations	No serious limitations	0.08	**0.08**	**Moderate**
**Fatal bleeding**							
301 (2)	No serious limitations	No serious limitations	No serious limitations	Serious limitations	0.02	**0.02**	**Very Low**
**Bleeding leading to hemoglobin below 70g/L (7g/dL)**							

Blank spaces indicate absence of information

* Estimate represents absolute risk in percent. Our median best estimates include fatal and non-fatal events. Based on data from included studies, we estimated case fatality rates as follows: 3.6% for VTE, 3.6% for bleeding leading to reintervention, and 0.9% for bleeding leading to transfusion, and used this information to calculate outcome estimates. For instance, we multiplied the median VTE estimate by 0.964 for non-fatal VTE and by 0.036 for fatal VTE (if both reintervention and transfusion rates were available, we preferred reintervention estimates for calculation of fatal bleeding estimate).

† Risk factors included 1) age more than 75 years, 2) obesity (body mass index of 35 or more), 3) VTE in a first degree relative (parents, full siblings, or children), and 4) prior VTE. We assumed that patients with any combination of two or more risk factors had a risk ratio of 4. Using these risk factors, we then categorized risk of VTE as low, medium, and high risk.

‡ Options for certainty in estimates are high, moderate, low, and very low. Evidence begins as high and is rated down for serious risk of bias, inconsistency, imprecision, or indirectness. We always rated down once due to uncertainty in the patient VTE risk factors and models of timing of VTE and bleeding. For fatal VTE and fatal bleeding we always rated down once for uncertainty in our case fatality rate estimates.

23. Evidence profile 23. Ventral hernia repair, open, emergency: Absolute risk of venous thromboembolism and bleeding among patients not receiving prophylaxis

Certainty assessment					Summary of findings		
**No of participants (studies)**	**Risk of Bias**	**Inconsistency**	**Indirectness**	**Imprecision**	**Best (median) estimate across all risk strata (%)***	**Best (median) estimate by patient risk strata (%)†**	**Evidence certainty‡**
**Non-fatal symptomatic venous thromboembolism**							
4,808 (1)	No serious limitations	No serious limitations	No serious limitations	No serious limitations	1.54	**Low: 1.20 Medium: 2.40 High: 4.79**	**Moderate**
**Fatal venous thromboembolism**							
4,808 (1)	No serious limitations	No serious limitations	No serious limitations	No serious limitations	0.06	**Low: 0.04 Medium: 0.09 High: 0.18**	**Low**
**Symptomatic splanchnic vein thrombosis**							
**Non-fatal bleeding requiring reintervention**							
**Non-fatal bleeding leading to transfusion**							
**Fatal bleeding**							
**Bleeding leading to hemoglobin below 70g/L (7g/dL)**							

Blank spaces indicate absence of information

* Estimate represents absolute risk in percent. Our median best estimates include fatal and non-fatal events. Based on data from included studies, we estimated case fatality rates as follows: 3.6% for VTE, 3.6% for bleeding leading to reintervention, and 0.9% for bleeding leading to transfusion, and used this information to calculate outcome estimates. For instance, we multiplied the median VTE estimate by 0.964 for non-fatal VTE and by 0.036 for fatal VTE (if both reintervention and transfusion rates were available, we preferred reintervention estimates for calculation of fatal bleeding estimate).

† Risk factors included 1) age more than 75 years, 2) obesity (body mass index of 35 or more), 3) VTE in a first degree relative (parents, full siblings, or children), and 4) prior VTE. We assumed that patients with any combination of two or more risk factors had a risk ratio of 4. Using these risk factors, we then categorized risk of VTE as low, medium, and high risk.

‡ Options for certainty in estimates are high, moderate, low, and very low. Evidence begins as high and is rated down for serious risk of bias, inconsistency, imprecision, or indirectness. We always rated down once due to uncertainty in the patient VTE risk factors and models of timing of VTE and bleeding. For fatal VTE and fatal bleeding we always rated down once for uncertainty in our case fatality rate estimates.

24. Evidence profile 24. Small bowel resection, laparoscopic: Absolute risk of venous thromboembolism and bleeding among patients not receiving prophylaxis

Certainty assessment					Summary of findings		
**No of participants (studies)**	**Risk of Bias**	**Inconsistency**	**Indirectness**	**Imprecision**	**Best (median) estimate across all risk strata (%)***	**Best (median) estimate by patient risk strata (%)†**	**Evidence certainty‡**
**Non-fatal symptomatic venous thromboembolism**							
3,195 (2)	No serious limitations	No serious limitations	No serious limitations	No serious limitations	1.50	**Low: 1.10 Medium: 2.19 High: 4.39**	**Moderate**
**Fatal venous thromboembolism**							
3,195 (2)	No serious limitations	No serious limitations	No serious limitations	No serious limitations	0.06	**Low: 0.04 Medium: 0.08 High: 0.16**	**Low**
**Symptomatic splanchnic vein thrombosis**							
**Non-fatal bleeding requiring reintervention**							
**Non-fatal bleeding leading to transfusion**							
**Fatal bleeding**							
**Bleeding leading to hemoglobin below 70g/L (7g/dL)**							

Blank spaces indicate absence of information

* Estimate represents absolute risk in percent. Our median best estimates include fatal and non-fatal events. Based on data from included studies, we estimated case fatality rates as follows: 3.6% for VTE, 3.6% for bleeding leading to reintervention, and 0.9% for bleeding leading to transfusion, and used this information to calculate outcome estimates. For instance, we multiplied the median VTE estimate by 0.964 for non-fatal VTE and by 0.036 for fatal VTE (if both reintervention and transfusion rates were available, we preferred reintervention estimates for calculation of fatal bleeding estimate).

† Risk factors included 1) age more than 75 years, 2) obesity (body mass index of 35 or more), 3) VTE in a first degree relative (parents, full siblings, or children), and 4) prior VTE. We assumed that patients with any combination of two or more risk factors had a risk ratio of 4. Using these risk factors, we then categorized risk of VTE as low, medium, and high risk.

‡ Options for certainty in estimates are high, moderate, low, and very low. Evidence begins as high and is rated down for serious risk of bias, inconsistency, imprecision, or indirectness. We always rated down once due to uncertainty in the patient VTE risk factors and models of timing of VTE and bleeding. For fatal VTE and fatal bleeding we always rated down once for uncertainty in our case fatality rate estimates.

25. Evidence profile 25. Small bowel resection, open: Absolute risk of venous thromboembolism and bleeding among patients not receiving prophylaxis

Certainty assessment					Summary of findings		
**No of participants (studies)**	**Risk of Bias**	**Inconsistency**	**Indirectness**	**Imprecision**	**Best (median) estimate across all risk strata (%)***	**Best (median) estimate by patient risk strata (%)†**	**Evidence certainty‡**
**Non-fatal symptomatic venous thromboembolism**							
28,148 (3)	No serious limitations	No serious limitations	No serious limitations	No serious limitations	3.55	**Low: 2.57 Medium: 5.13 High: 10.27**	**Moderate**
**Fatal venous thromboembolism**							
28,148 (3)	No serious limitations	No serious limitations	No serious limitations	No serious limitations	0.13	**Low: 0.1 Medium: 0.19 High: 0.38**	**Low**
**Symptomatic splanchnic vein thrombosis**							
**Non-fatal bleeding requiring reintervention**							
**Non-fatal bleeding leading to transfusion**							
**Fatal bleeding**							
**Bleeding leading to hemoglobin below 70g/L (7g/dL)**							

Blank spaces indicate absence of information

* Estimate represents absolute risk in percent. Our median best estimates include fatal and non-fatal events. Based on data from included studies, we estimated case fatality rates as follows: 3.6% for VTE, 3.6% for bleeding leading to reintervention, and 0.9% for bleeding leading to transfusion, and used this information to calculate outcome estimates. For instance, we multiplied the median VTE estimate by 0.964 for non-fatal VTE and by 0.036 for fatal VTE (if both reintervention and transfusion rates were available, we preferred reintervention estimates for calculation of fatal bleeding estimate).

† Risk factors included 1) age more than 75 years, 2) obesity (body mass index of 35 or more), 3) VTE in a first degree relative (parents, full siblings, or children), and 4) prior VTE. We assumed that patients with any combination of two or more risk factors had a risk ratio of 4. Using these risk factors, we then categorized risk of VTE as low, medium, and high risk.

‡ Options for certainty in estimates are high, moderate, low, and very low. Evidence begins as high and is rated down for serious risk of bias, inconsistency, imprecision, or indirectness. We always rated down once due to uncertainty in the patient VTE risk factors and models of timing of VTE and bleeding. For fatal VTE and fatal bleeding we always rated down once for uncertainty in our case fatality rate estimates.

26. Evidence profile 26. Small bowel resection, laparoscopic, benign: Absolute risk of venous thromboembolism and bleeding among patients not receiving prophylaxis

Certainty assessment					Summary of findings		
**No of participants (studies)**	**Risk of Bias**	**Inconsistency**	**Indirectness**	**Imprecision**	**Best (median) estimate across all risk strata (%)***	**Best (median) estimate by patient risk strata (%)†**	**Evidence certainty‡**
**Non-fatal symptomatic venous thromboembolism**							
355 (1)	No serious limitations	No serious limitations	No serious limitations	Serious limitations	1.02	**Low: 0.73 Medium: 1.46 High: 2.93**	**Low**
**Fatal venous thromboembolism**							
355 (1)	No serious limitations	No serious limitations	No serious limitations	Serious limitations	0.04	**Low: 0.03 Medium: 0.05 High: 0.11**	**Very Low**
**Symptomatic splanchnic vein thrombosis**							
**Non-fatal bleeding requiring reintervention**							
**Non-fatal bleeding leading to transfusion**							
**Fatal bleeding**							
**Bleeding leading to hemoglobin below 70g/L (7g/dL)**							

Blank spaces indicate absence of information

* Estimate represents absolute risk in percent. Our median best estimates include fatal and non-fatal events. Based on data from included studies, we estimated case fatality rates as follows: 3.6% for VTE, 3.6% for bleeding leading to reintervention, and 0.9% for bleeding leading to transfusion, and used this information to calculate outcome estimates. For instance, we multiplied the median VTE estimate by 0.964 for non-fatal VTE and by 0.036 for fatal VTE (if both reintervention and transfusion rates were available, we preferred reintervention estimates for calculation of fatal bleeding estimate).

† Risk factors included 1) age more than 75 years, 2) obesity (body mass index of 35 or more), 3) VTE in a first degree relative (parents, full siblings, or children), and 4) prior VTE. We assumed that patients with any combination of two or more risk factors had a risk ratio of 4. Using these risk factors, we then categorized risk of VTE as low, medium, and high risk.

‡ Options for certainty in estimates are high, moderate, low, and very low. Evidence begins as high and is rated down for serious risk of bias, inconsistency, imprecision, or indirectness. We always rated down once due to uncertainty in the patient VTE risk factors and models of timing of VTE and bleeding. For fatal VTE and fatal bleeding we always rated down once for uncertainty in our case fatality rate estimates.

27. Evidence profile 27. Small bowel resection, laparoscopic, malignant: Absolute risk of venous thromboembolism and bleeding among patients not receiving prophylaxis

Certainty assessment					Summary of findings		
**No of participants (studies)**	**Risk of Bias**	**Inconsistency**	**Indirectness**	**Imprecision**	**Best (median) estimate across all risk strata (%)***	**Best (median) estimate by patient risk strata (%)†**	**Evidence certainty‡**
**Non-fatal symptomatic venous thromboembolism**							
499 (1)	No serious limitations	No serious limitations	No serious limitations	Serious limitations	2.18	**Low: 1.39 Medium: 2.79 High: 5.57**	**Low**
**Fatal venous thromboembolism**							
499 (1)	No serious limitations	No serious limitations	No serious limitations	Serious limitations	0.08	**Low: 0.05 Medium: 0.1 High: 0.21**	**Very Low**
**Symptomatic splanchnic vein thrombosis**							
**Non-fatal bleeding requiring reintervention**							
**Non-fatal bleeding leading to transfusion**							
**Fatal bleeding**							
**Bleeding leading to hemoglobin below 70g/L (7g/dL)**							

Blank spaces indicate absence of information

* Estimate represents absolute risk in percent. Our median best estimates include fatal and non-fatal events. Based on data from included studies, we estimated case fatality rates as follows: 3.6% for VTE, 3.6% for bleeding leading to reintervention, and 0.9% for bleeding leading to transfusion, and used this information to calculate outcome estimates. For instance, we multiplied the median VTE estimate by 0.964 for non-fatal VTE and by 0.036 for fatal VTE (if both reintervention and transfusion rates were available, we preferred reintervention estimates for calculation of fatal bleeding estimate).

† Risk factors included 1) age more than 75 years, 2) obesity (body mass index of 35 or more), 3) VTE in a first degree relative (parents, full siblings, or children), and 4) prior VTE. We assumed that patients with any combination of two or more risk factors had a risk ratio of 4. Using these risk factors, we then categorized risk of VTE as low, medium, and high risk.

‡ Options for certainty in estimates are high, moderate, low, and very low. Evidence begins as high and is rated down for serious risk of bias, inconsistency, imprecision, or indirectness. We always rated down once due to uncertainty in the patient VTE risk factors and models of timing of VTE and bleeding. For fatal VTE and fatal bleeding we always rated down once for uncertainty in our case fatality rate estimates.

28. Evidence profile 28. Small bowel resection, laparoscopic, IBD: Absolute risk of venous thromboembolism and bleeding among patients not receiving prophylaxis

Certainty assessment					Summary of findings		
**No of participants (studies)**	**Risk of Bias**	**Inconsistency**	**Indirectness**	**Imprecision**	**Best (median) estimate across all risk strata (%)***	**Best (median) estimate by patient risk strata (%)†**	**Evidence certainty‡**
**Non-fatal symptomatic venous thromboembolism**							
443 (1)	No serious limitations	No serious limitations	No serious limitations	Serious limitations	1.09	**Low: 0.93 Medium: 1.87 High: 3.74**	**Low**
**Fatal venous thromboembolism**							
443 (1)	No serious limitations	No serious limitations	No serious limitations	Serious limitations	0.04	**Low: 0.03 Medium: 0.07 High: 0.14**	**Very Low**
**Symptomatic splanchnic vein thrombosis**							
**Non-fatal bleeding requiring reintervention**							
**Non-fatal bleeding leading to transfusion**							
**Fatal bleeding**							
**Bleeding leading to hemoglobin below 70g/L (7g/dL)**							

Blank spaces indicate absence of information

* Estimate represents absolute risk in percent. Our median best estimates include fatal and non-fatal events. Based on data from included studies, we estimated case fatality rates as follows: 3.6% for VTE, 3.6% for bleeding leading to reintervention, and 0.9% for bleeding leading to transfusion, and used this information to calculate outcome estimates. For instance, we multiplied the median VTE estimate by 0.964 for non-fatal VTE and by 0.036 for fatal VTE (if both reintervention and transfusion rates were available, we preferred reintervention estimates for calculation of fatal bleeding estimate).

† Risk factors included 1) age more than 75 years, 2) obesity (body mass index of 35 or more), 3) VTE in a first degree relative (parents, full siblings, or children), and 4) prior VTE. We assumed that patients with any combination of two or more risk factors had a risk ratio of 4. Using these risk factors, we then categorized risk of VTE as low, medium, and high risk.

‡ Options for certainty in estimates are high, moderate, low, and very low. Evidence begins as high and is rated down for serious risk of bias, inconsistency, imprecision, or indirectness. We always rated down once due to uncertainty in the patient VTE risk factors and models of timing of VTE and bleeding. For fatal VTE and fatal bleeding we always rated down once for uncertainty in our case fatality rate estimates.

29. Evidence profile 29. Small bowel resection, laparoscopic, emergency: Absolute risk of venous thromboembolism and bleeding among patients not receiving prophylaxis

Certainty assessment					Summary of findings		
**No of participants (studies)**	**Risk of Bias**	**Inconsistency**	**Indirectness**	**Imprecision**	**Best (median) estimate across all risk strata (%)***	**Best (median) estimate by patient risk strata (%)†**	**Evidence certainty‡**
**Non-fatal symptomatic venous thromboembolism**							
118 (1)	No serious limitations	No serious limitations	No serious limitations	Very serious limitations	0.00	**Low: 0.00 Medium: 0.00 High: 0.00**	**Very low**
**Fatal venous thromboembolism**							
118 (1)	No serious limitations	No serious limitations	No serious limitations	Very serious limitations	0.00	**Low: 0.00 Medium: 0.00 High: 0.00**	**Very low**
**Symptomatic splanchnic vein thrombosis**							
**Non-fatal bleeding requiring reintervention**							
**Non-fatal bleeding leading to transfusion**							
**Fatal bleeding**							
**Bleeding leading to hemoglobin below 70g/L (7g/dL)**							

Blank spaces indicate absence of information

* Estimate represents absolute risk in percent. Our median best estimates include fatal and non-fatal events. Based on data from included studies, we estimated case fatality rates as follows: 3.6% for VTE, 3.6% for bleeding leading to reintervention, and 0.9% for bleeding leading to transfusion, and used this information to calculate outcome estimates. For instance, we multiplied the median VTE estimate by 0.964 for non-fatal VTE and by 0.036 for fatal VTE (if both reintervention and transfusion rates were available, we preferred reintervention estimates for calculation of fatal bleeding estimate).

† Risk factors included 1) age more than 75 years, 2) obesity (body mass index of 35 or more), 3) VTE in a first degree relative (parents, full siblings, or children), and 4) prior VTE. We assumed that patients with any combination of two or more risk factors had a risk ratio of 4. Using these risk factors, we then categorized risk of VTE as low, medium, and high risk.

‡ Options for certainty in estimates are high, moderate, low, and very low. Evidence begins as high and is rated down for serious risk of bias, inconsistency, imprecision, or indirectness. We always rated down once due to uncertainty in the patient VTE risk factors and models of timing of VTE and bleeding. For fatal VTE and fatal bleeding we always rated down once for uncertainty in our case fatality rate estimates.

30. Evidence profile 30. Small bowel resection, open, benign: Absolute risk of venous thromboembolism and bleeding among patients not receiving prophylaxis

Certainty assessment					Summary of findings		
**No of participants (studies)**	**Risk of Bias**	**Inconsistency**	**Indirectness**	**Imprecision**	**Best (median) estimate across all risk strata (%)***	**Best (median) estimate by patient risk strata (%)†**	**Evidence certainty‡**
**Non-fatal symptomatic venous thromboembolism**							
571 (1)	No serious limitations	No serious limitations	No serious limitations	Serious limitations	0.85	**Low: 0.52 Medium: 1.04 High: 2.08**	**Low**
**Fatal venous thromboembolism**							
571 (1)	No serious limitations	No serious limitations	No serious limitations	Serious limitations	0.03	**Low: 0.02 Medium: 0.04 High: 0.08**	**Very Low**
**Symptomatic splanchnic vein thrombosis**							
**Non-fatal bleeding requiring reintervention**							
**Non-fatal bleeding leading to transfusion**							
**Fatal bleeding**							
**Bleeding leading to hemoglobin below 70g/L (7g/dL)**							

Blank spaces indicate absence of information

* Estimate represents absolute risk in percent. Our median best estimates include fatal and non-fatal events. Based on data from included studies, we estimated case fatality rates as follows: 3.6% for VTE, 3.6% for bleeding leading to reintervention, and 0.9% for bleeding leading to transfusion, and used this information to calculate outcome estimates. For instance, we multiplied the median VTE estimate by 0.964 for non-fatal VTE and by 0.036 for fatal VTE (if both reintervention and transfusion rates were available, we preferred reintervention estimates for calculation of fatal bleeding estimate).

† Risk factors included 1) age more than 75 years, 2) obesity (body mass index of 35 or more), 3) VTE in a first degree relative (parents, full siblings, or children), and 4) prior VTE. We assumed that patients with any combination of two or more risk factors had a risk ratio of 4. Using these risk factors, we then categorized risk of VTE as low, medium, and high risk.

‡ Options for certainty in estimates are high, moderate, low, and very low. Evidence begins as high and is rated down for serious risk of bias, inconsistency, imprecision, or indirectness. We always rated down once due to uncertainty in the patient VTE risk factors and models of timing of VTE and bleeding. For fatal VTE and fatal bleeding we always rated down once for uncertainty in our case fatality rate estimates.

31. Evidence profile 31. Small bowel resection, open, malignant: Absolute risk of venous thromboembolism and bleeding among patients not receiving prophylaxis

Certainty assessment					Summary of findings		
**No of participants (studies)**	**Risk of Bias**	**Inconsistency**	**Indirectness**	**Imprecision**	**Best (median) estimate across all risk strata (%)***	**Best (median) estimate by patient risk strata (%)†**	**Evidence certainty‡**
**Non-fatal symptomatic venous thromboembolism**							
1,784 (1)	No serious limitations	No serious limitations	No serious limitations	No serious limitations	3.25	**Low: 2.22 Medium: 4.43 High: 8.86**	**Moderate**
**Fatal venous thromboembolism**							
1,784 (1)	No serious limitations	No serious limitations	No serious limitations	No serious limitations	0.12	**Low: 0.08 Medium: 0.17 High: 0.33**	**Low**
**Symptomatic splanchnic vein thrombosis**							
**Non-fatal bleeding requiring reintervention**							
**Non-fatal bleeding leading to transfusion**							
**Fatal bleeding**							
**Bleeding leading to hemoglobin below 70g/L (7g/dL)**							

Blank spaces indicate absence of information

* Estimate represents absolute risk in percent. Our median best estimates include fatal and non-fatal events. Based on data from included studies, we estimated case fatality rates as follows: 3.6% for VTE, 3.6% for bleeding leading to reintervention, and 0.9% for bleeding leading to transfusion, and used this information to calculate outcome estimates. For instance, we multiplied the median VTE estimate by 0.964 for non-fatal VTE and by 0.036 for fatal VTE (if both reintervention and transfusion rates were available, we preferred reintervention estimates for calculation of fatal bleeding estimate).

† Risk factors included 1) age more than 75 years, 2) obesity (body mass index of 35 or more), 3) VTE in a first degree relative (parents, full siblings, or children), and 4) prior VTE. We assumed that patients with any combination of two or more risk factors had a risk ratio of 4. Using these risk factors, we then categorized risk of VTE as low, medium, and high risk.

‡ Options for certainty in estimates are high, moderate, low, and very low. Evidence begins as high and is rated down for serious risk of bias, inconsistency, imprecision, or indirectness. We always rated down once due to uncertainty in the patient VTE risk factors and models of timing of VTE and bleeding. For fatal VTE and fatal bleeding we always rated down once for uncertainty in our case fatality rate estimates.

32. Evidence profile 32. Small bowel resection, open, inflammatory bowel disease (IBD): Absolute risk of venous thromboembolism and bleeding among patients not receiving prophylaxis

Certainty assessment					Summary of findings		
**No of participants (studies)**	**Risk of Bias**	**Inconsistency**	**Indirectness**	**Imprecision**	**Best (median) estimate across all risk strata (%)***	**Best (median) estimate by patient risk strata (%)†**	**Evidence certainty‡**
**Non-fatal symptomatic venous thromboembolism**							
1,237 (1)	No serious limitations	No serious limitations	No serious limitations	No serious limitations	1.95	**Low: 1.64 Medium: 3.29 High: 6.57**	**Moderate**
**Fatal venous thromboembolism**							
1,237 (1)	No serious limitations	No serious limitations	No serious limitations	No serious limitations	0.07	**Low: 0.06 Medium: 0.12 High: 0.25**	**Low**
**Symptomatic splanchnic vein thrombosis**							
**Non-fatal bleeding requiring reintervention**							
**Non-fatal bleeding leading to transfusion**							
**Fatal bleeding**							
**Bleeding leading to hemoglobin below 70g/L (7g/dL)**							

Blank spaces indicate absence of information

* Estimate represents absolute risk in percent. Our median best estimates include fatal and non-fatal events. Based on data from included studies, we estimated case fatality rates as follows: 3.6% for VTE, 3.6% for bleeding leading to reintervention, and 0.9% for bleeding leading to transfusion, and used this information to calculate outcome estimates. For instance, we multiplied the median VTE estimate by 0.964 for non-fatal VTE and by 0.036 for fatal VTE (if both reintervention and transfusion rates were available, we preferred reintervention estimates for calculation of fatal bleeding estimate).

† Risk factors included 1) age more than 75 years, 2) obesity (body mass index of 35 or more), 3) VTE in a first degree relative (parents, full siblings, or children), and 4) prior VTE. We assumed that patients with any combination of two or more risk factors had a risk ratio of 4. Using these risk factors, we then categorized risk of VTE as low, medium, and high risk.

‡ Options for certainty in estimates are high, moderate, low, and very low. Evidence begins as high and is rated down for serious risk of bias, inconsistency, imprecision, or indirectness. We always rated down once due to uncertainty in the patient VTE risk factors and models of timing of VTE and bleeding. For fatal VTE and fatal bleeding we always rated down once for uncertainty in our case fatality rate estimates.

33. Evidence profile 33. Small bowel resection, open, emergency: Absolute risk of venous thromboembolism and bleeding among patients not receiving prophylaxis

Certainty assessment					Summary of findings		
**No of participants (studies)**	**Risk of Bias**	**Inconsistency**	**Indirectness**	**Imprecision**	**Best (median) estimate across all risk strata (%)***	**Best (median) estimate by patient risk strata (%)†**	**Evidence certainty‡**
**Non-fatal symptomatic venous thromboembolism**							
6,855 (1)	No serious limitations	No serious limitations	No serious limitations	No serious limitations	3.55	**Low: 2.77 Medium: 5.54 High: 11.09**	**Moderate**
**Fatal venous thromboembolism**							
6,855 (1)	No serious limitations	No serious limitations	No serious limitations	No serious limitations	0.13	**Low: 0.1 Medium: 0.21 High: 0.41**	**Low**
**Symptomatic splanchnic vein thrombosis**							
**Non-fatal bleeding requiring reintervention**							
**Non-fatal bleeding leading to transfusion**							
**Fatal bleeding**							
**Bleeding leading to hemoglobin below 70g/L (7g/dL)**							

Blank spaces indicate absence of information

* Estimate represents absolute risk in percent. Our median best estimates include fatal and non-fatal events. Based on data from included studies, we estimated case fatality rates as follows: 3.6% for VTE, 3.6% for bleeding leading to reintervention, and 0.9% for bleeding leading to transfusion, and used this information to calculate outcome estimates. For instance, we multiplied the median VTE estimate by 0.964 for non-fatal VTE and by 0.036 for fatal VTE (if both reintervention and transfusion rates were available, we preferred reintervention estimates for calculation of fatal bleeding estimate).

† Risk factors included 1) age more than 75 years, 2) obesity (body mass index of 35 or more), 3) VTE in a first degree relative (parents, full siblings, or children), and 4) prior VTE. We assumed that patients with any combination of two or more risk factors had a risk ratio of 4. Using these risk factors, we then categorized risk of VTE as low, medium, and high risk.

‡ Options for certainty in estimates are high, moderate, low, and very low. Evidence begins as high and is rated down for serious risk of bias, inconsistency, imprecision, or indirectness. We always rated down once due to uncertainty in the patient VTE risk factors and models of timing of VTE and bleeding. For fatal VTE and fatal bleeding we always rated down once for uncertainty in our case fatality rate estimates.

34. Evidence profile 34. Splenectomy, laparoscopic, elective: Absolute risk of venous thromboembolism and bleeding among patients not receiving prophylaxis

Certainty assessment					Summary of findings		
**No of participants (studies)**	**Risk of Bias**	**Inconsistency**	**Indirectness**	**Imprecision**	**Best (median) estimate across all risk strata (%)***	**Best (median) estimate by patient risk strata (%)†**	**Evidence certainty‡**
**Non-fatal symptomatic venous thromboembolism**							
5,177 (5)	No serious limitations	No serious limitations	No serious limitations	No serious limitations	2.78	**Low: 2.29 Medium: 4.59 High: 9.18**	**Moderate**
**Fatal venous thromboembolism**							
5,177 (5)	No serious limitations	No serious limitations	No serious limitations	No serious limitations	0.10	**Low: 0.09 Medium: 0.17 High: 0.34**	**Low**
**Symptomatic splanchnic vein thrombosis§**							
2,233 (9)	Serious limitations	Serious limitations	No serious limitations	No serious limitations	1.83	**1.83**	**Very low**
**Non-fatal bleeding requiring reintervention**							
2,203 (8)	Serious limitations	No serious limitations	No serious limitations	No serious limitations	1.23	**1.23**	**Low**
**Non-fatal bleeding leading to transfusion**							
5,125 (3)	No serious limitations	No serious limitations	No serious limitations	No serious limitations	0.89	**0.89**	**Moderate**
**Fatal bleeding**							
2,203 (8)	Serious limitations	No serious limitations	No serious limitations	No serious limitations	0.05	**0.05**	**Very Low**
**Bleeding leading to hemoglobin below 70g/L (7g/dL)**							

Blank spaces indicate absence of information

* Estimate represents absolute risk in percent. Our median best estimates include fatal and non-fatal events. Based on data from included studies, we estimated case fatality rates as follows: 3.6% for VTE, 3.6% for bleeding leading to reintervention, and 0.9% for bleeding leading to transfusion, and used this information to calculate outcome estimates. For instance, we multiplied the median VTE estimate by 0.964 for non-fatal VTE and by 0.036 for fatal VTE (if both reintervention and transfusion rates were available, we preferred reintervention estimates for calculation of fatal bleeding estimate).

† Risk factors included 1) age more than 75 years, 2) obesity (body mass index of 35 or more), 3) VTE in a first degree relative (parents, full siblings, or children), and 4) prior VTE. We assumed that patients with any combination of two or more risk factors had a risk ratio of 4. Using these risk factors, we then categorized risk of VTE as low, medium, and high risk.

‡ Options for certainty in estimates are high, moderate, low, and very low. Evidence begins as high and is rated down for serious risk of bias, inconsistency, imprecision, or indirectness. We always rated down once due to uncertainty in the patient VTE risk factors and models of timing of VTE and bleeding. For fatal VTE and fatal bleeding we always rated down once for uncertainty in our case fatality rate estimates.

§The best median estimate for symptomatic splanchnic vein thrombosis is median value of reported estimates. As we did not find evidence for timing of SVT, effect of thromboprophylaxis on SVT or patient risk factors for SVT, we did not model splanchnic vein thrombosis estimates for these factors.

35. Evidence profile 35. Splenectomy, open, elective: Absolute risk of venous thromboembolism and bleeding among patients not receiving prophylaxis

Certainty assessment					Summary of findings		
**No of participants (studies)**	**Risk of Bias**	**Inconsistency**	**Indirectness**	**Imprecision**	**Best (median) estimate across all risk strata (%)***	**Best (median) estimate by patient risk strata (%)†**	**Evidence certainty‡**
**Non-fatal symptomatic venous thromboembolism**							
2,590 (3)	No serious limitations	Serious limitations	No serious limitations	No serious limitations	1.78	**Low: 1.37 Medium: 2.75 High: 5.49**	**Low**
**Fatal venous thromboembolism**							
2,590 (3)	No serious limitations	Serious limitations	No serious limitations	No serious limitations	0.07	**Low: 0.05 Medium: 0.1 High: 0.21**	**Very Low**
**Symptomatic splanchnic vein thrombosis§**							
557 (4)	Serious limitations	Serious limitations	No serious limitations	Serious limitations	5.16	**5.16**	**Very low**
**Non-fatal bleeding requiring reintervention**							
385 (2)	Serious limitations	No serious limitations	No serious limitations	Serious limitations	3.81	**3.81**	**Very low**
**Non-fatal bleeding leading to transfusion**							
2,276 (2)	No serious limitations	No serious limitations	No serious limitations	No serious limitations	1.18	**1.18**	**Moderate**
**Fatal bleeding**							
385 (2)	Serious limitations	No serious limitations	No serious limitations	Serious limitations	0.14	**0.14**	**Very low**
**Bleeding leading to hemoglobin below 70g/L (7g/dL)**							

Blank spaces indicate absence of information

* Estimate represents absolute risk in percent. Our median best estimates include fatal and non-fatal events. Based on data from included studies, we estimated case fatality rates as follows: 3.6% for VTE, 3.6% for bleeding leading to reintervention, and 0.9% for bleeding leading to transfusion, and used this information to calculate outcome estimates. For instance, we multiplied the median VTE estimate by 0.964 for non-fatal VTE and by 0.036 for fatal VTE (if both reintervention and transfusion rates were available, we preferred reintervention estimates for calculation of fatal bleeding estimate).

† Risk factors included 1) age more than 75 years, 2) obesity (body mass index of 35 or more), 3) VTE in a first degree relative (parents, full siblings, or children), and 4) prior VTE. We assumed that patients with any combination of two or more risk factors had a risk ratio of 4. Using these risk factors, we then categorized risk of VTE as low, medium, and high risk.

‡ Options for certainty in estimates are high, moderate, low, and very low. Evidence begins as high and is rated down for serious risk of bias, inconsistency, imprecision, or indirectness. We always rated down once due to uncertainty in the patient VTE risk factors and models of timing of VTE and bleeding. For fatal VTE and fatal bleeding we always rated down once for uncertainty in our case fatality rate estimates.

§The best median estimate for symptomatic splanchnic vein thrombosis is median value of reported estimates. As we did not find evidence for timing of SVT, effect of thromboprophylaxis on SVT or patient risk factors for SVT, we did not model splanchnic vein thrombosis estimates for these factors.

36. Evidence profile 36. Splenectomy, laparoscopic, elective, benign: Absolute risk of venous thromboembolism and bleeding among patients not receiving prophylaxis

Certainty assessment					Summary of findings		
**No of participants (studies)**	**Risk of Bias**	**Inconsistency**	**Indirectness**	**Imprecision**	**Best (median) estimate across all risk strata (%)***	**Best (median) estimate by patient risk strata (%)†**	**Evidence certainty‡**
**Non-fatal symptomatic venous thromboembolism**							
512 (3)	Serious limitations	No serious limitations	No serious limitations	Serious limitations	2.78	**Low: 2.34 Medium: 4.67 High: 9.35**	**Very low**
**Fatal venous thromboembolism**							
512 (3)	Serious limitations	No serious limitations	No serious limitations	Serious limitations	0.10	**Low: 0.09 Medium: 0.17 High: 0.35**	**Very low**
**Symptomatic splanchnic vein thrombosis§**							
369 (2)	Serious limitations	No serious limitations	No serious limitations	Serious limitations	2.26	**2.26**	**Very low**
**Non-fatal bleeding requiring reintervention**							
512 (3)	Serious limitations	No serious limitations	No serious limitations	Serious limitations	0.66	**0.66**	**Very low**
**Non-fatal bleeding leading to transfusion**							
454 (2)	Serious limitations	No serious limitations	No serious limitations	Serious limitations	2.52	**2.52**	**Very low**
**Fatal bleeding**							
512 (3)	Serious limitations	No serious limitations	No serious limitations	Serious limitations	0.02	**0.02**	**Very low**
**Bleeding leading to hemoglobin below 70g/L (7g/dL)**							

Blank spaces indicate absence of information

* Estimate represents absolute risk in percent. Our median best estimates include fatal and non-fatal events. Based on data from included studies, we estimated case fatality rates as follows: 3.6% for VTE, 3.6% for bleeding leading to reintervention, and 0.9% for bleeding leading to transfusion, and used this information to calculate outcome estimates. For instance, we multiplied the median VTE estimate by 0.964 for non-fatal VTE and by 0.036 for fatal VTE (if both reintervention and transfusion rates were available, we preferred reintervention estimates for calculation of fatal bleeding estimate).

† Risk factors included 1) age more than 75 years, 2) obesity (body mass index of 35 or more), 3) VTE in a first degree relative (parents, full siblings, or children), and 4) prior VTE. We assumed that patients with any combination of two or more risk factors had a risk ratio of 4. Using these risk factors, we then categorized risk of VTE as low, medium, and high risk.

‡ Options for certainty in estimates are high, moderate, low, and very low. Evidence begins as high and is rated down for serious risk of bias, inconsistency, imprecision, or indirectness. We always rated down once due to uncertainty in the patient VTE risk factors and models of timing of VTE and bleeding. For fatal VTE and fatal bleeding we always rated down once for uncertainty in our case fatality rate estimates.

§The best median estimate for symptomatic splanchnic vein thrombosis is median value of reported estimates. As we did not find evidence for timing of SVT, effect of thromboprophylaxis on SVT or patient risk factors for SVT, we did not model splanchnic vein thrombosis estimates for these factors.

37. Evidence profile 37. Splenectomy, laparoscopic, elective, immune thrombocytopenia (ITP): Absolute risk of venous thromboembolism and bleeding among patients not receiving prophylaxis

Certainty assessment					Summary of findings		
**No of participants (studies)**	**Risk of Bias**	**Inconsistency**	**Indirectness**	**Imprecision**	**Best (median) estimate across all risk strata (%)***	**Best (median) estimate by patient risk strata (%)†**	**Evidence certainty‡**
**Non-fatal symptomatic venous thromboembolism**							
512 (3)	Serious limitations	No serious limitations	No serious limitations	Serious limitations	2.78	**Low: 2.32 Medium: 4.63 High: 9.26**	**Very low**
**Fatal venous thromboembolism**							
512 (3)	Serious limitations	No serious limitations	No serious limitations	Serious limitations	0.10	**Low: 0.09 Medium: 0.17 High: 0.35**	**Very low**
**Symptomatic splanchnic vein thrombosis**							
109 (1)	Serious limitations	No serious limitations	No serious limitations	Very serious limitations	0.00	**0.00**	**Very low**
**Non-fatal bleeding requiring reintervention**							
512 (3)	Serious limitations	No serious limitations	No serious limitations	Serious limitations	0.66	**0.66**	**Very low**
**Non-fatal bleeding leading to transfusion**							
194 (1)	Serious limitations	No serious limitations	No serious limitations	Very serious limitations	3.18	**3.18**	**Very low**
**Fatal bleeding**							
512 (3)	Serious limitations	No serious limitations	No serious limitations	Serious limitations	0.02	**0.02**	**Very low**
**Bleeding leading to hemoglobin below 70g/L (7g/dL)**							

Blank spaces indicate absence of information

* Estimate represents absolute risk in percent. Our median best estimates include fatal and non-fatal events. Based on data from included studies, we estimated case fatality rates as follows: 3.6% for VTE, 3.6% for bleeding leading to reintervention, and 0.9% for bleeding leading to transfusion, and used this information to calculate outcome estimates. For instance, we multiplied the median VTE estimate by 0.964 for non-fatal VTE and by 0.036 for fatal VTE (if both reintervention and transfusion rates were available, we preferred reintervention estimates for calculation of fatal bleeding estimate).

† Risk factors included 1) age more than 75 years, 2) obesity (body mass index of 35 or more), 3) VTE in a first degree relative (parents, full siblings, or children), and 4) prior VTE. We assumed that patients with any combination of two or more risk factors had a risk ratio of 4. Using these risk factors, we then categorized risk of VTE as low, medium, and high risk.

‡ Options for certainty in estimates are high, moderate, low, and very low. Evidence begins as high and is rated down for serious risk of bias, inconsistency, imprecision, or indirectness. We always rated down once due to uncertainty in the patient VTE risk factors and models of timing of VTE and bleeding. For fatal VTE and fatal bleeding we always rated down once for uncertainty in our case fatality rate estimates.

38. Evidence profile 38. Splenectomy, open, elective, benign: Absolute risk of venous thromboembolism and bleeding among patients not receiving prophylaxis

Certainty assessment					Summary of findings		
**No of participants (studies)**	**Risk of Bias**	**Inconsistency**	**Indirectness**	**Imprecision**	**Best (median) estimate across all risk strata (%)***	**Best (median) estimate by patient risk strata (%)†**	**Evidence certainty‡**
**Non-fatal symptomatic venous thromboembolism**							
56 (1)	Serious limitations	No serious limitations	No serious limitations	Very serious limitations	1.67	**Low: 1.38 Medium: 2.76 High: 5.52**	**Very low**
**Fatal venous thromboembolism**							
56 (1)	Serious limitations	No serious limitations	No serious limitations	Very serious limitations	0.06	**Low: 0.05 Medium: 0.1 High: 0.21**	**Very low**
**Symptomatic splanchnic vein thrombosis**							
71 (1)	Serious limitations	No serious limitations	No serious limitations	Very serious limitations	0.00	**0.00**	**Very low**
**Non-fatal bleeding requiring reintervention**							
71 (1)	Serious limitations	No serious limitations	No serious limitations	Very serious limitations	3.99	**3.99**	**Very low**
**Non-fatal bleeding leading to transfusion**							
56 (1)	Serious limitations	No serious limitations	No serious limitations	Very serious limitations	0.00	**0.00**	**Very low**
**Fatal bleeding**							
71 (1)	Serious limitations	No serious limitations	No serious limitations	Very serious limitations	0.15	**0.15**	**Very low**
**Bleeding leading to hemoglobin below 70g/L (7g/dL)**							

Blank spaces indicate absence of information

* Estimate represents absolute risk in percent. Our median best estimates include fatal and non-fatal events. Based on data from included studies, we estimated case fatality rates as follows: 3.6% for VTE, 3.6% for bleeding leading to reintervention, and 0.9% for bleeding leading to transfusion, and used this information to calculate outcome estimates. For instance, we multiplied the median VTE estimate by 0.964 for non-fatal VTE and by 0.036 for fatal VTE (if both reintervention and transfusion rates were available, we preferred reintervention estimates for calculation of fatal bleeding estimate).

† Risk factors included 1) age more than 75 years, 2) obesity (body mass index of 35 or more), 3) VTE in a first degree relative (parents, full siblings, or children), and 4) prior VTE. We assumed that patients with any combination of two or more risk factors had a risk ratio of 4. Using these risk factors, we then categorized risk of VTE as low, medium, and high risk.

‡ Options for certainty in estimates are high, moderate, low, and very low. Evidence begins as high and is rated down for serious risk of bias, inconsistency, imprecision, or indirectness. We always rated down once due to uncertainty in the patient VTE risk factors and models of timing of VTE and bleeding. For fatal VTE and fatal bleeding we always rated down once for uncertainty in our case fatality rate estimates.

§We did not model splanchnic vein thrombosis estimates for timing, use of thromboprophylaxis or patient risk factors as we did not find available evidence for timing of SVT, effect of thromboprophylaxis on SVT or patient risk factors for SVT.

39. Evidence profile 39. Splenectomy, open, elective, malignant: Absolute risk of venous thromboembolism and bleeding among patients not receiving prophylaxis

Certainty assessment					Summary of findings		
**No of participants (studies)**	**Risk of Bias**	**Inconsistency**	**Indirectness**	**Imprecision**	**Best (median) estimate across all risk strata (%)***	**Best (median) estimate by patient risk strata (%)†**	**Evidence certainty‡**
**Non-fatal symptomatic venous thromboembolism**							
314 (1)	Serious limitations	No serious limitations	No serious limitations	Serious limitations	1.67	**Low: 1.06 Medium: 2.12 High: 4.24**	**Very low**
**Fatal venous thromboembolism**							
314 (1)	Serious limitations	No serious limitations	No serious limitations	Serious limitations	0.06	**Low: 0.04 Medium: 0.08 High: 0.16**	**Very low**
**Symptomatic splanchnic vein thrombosis§**							
314 (1)	Serious limitations	No serious limitations	No serious limitations	Serious limitations	8.28	**8.28**	**Very low**
**Non-fatal bleeding requiring reintervention**							
314 (1)	Serious limitations	No serious limitations	No serious limitations	Serious limitations	3.63	**3.63**	**Very low**
**Non-fatal bleeding leading to transfusion**							
**Fatal bleeding**							
314 (1)	Serious limitations	No serious limitations	No serious limitations	Serious limitations	0.14	**0.14**	**Very low**
**Bleeding leading to hemoglobin below 70g/L (7g/dL)**							

Blank spaces indicate absence of information

* Estimate represents absolute risk in percent. Our median best estimates include fatal and non-fatal events. Based on data from included studies, we estimated case fatality rates as follows: 3.6% for VTE, 3.6% for bleeding leading to reintervention, and 0.9% for bleeding leading to transfusion, and used this information to calculate outcome estimates. For instance, we multiplied the median VTE estimate by 0.964 for non-fatal VTE and by 0.036 for fatal VTE (if both reintervention and transfusion rates were available, we preferred reintervention estimates for calculation of fatal bleeding estimate).

† Risk factors included 1) age more than 75 years, 2) obesity (body mass index of 35 or more), 3) VTE in a first degree relative (parents, full siblings, or children), and 4) prior VTE. We assumed that patients with any combination of two or more risk factors had a risk ratio of 4. Using these risk factors, we then categorized risk of VTE as low, medium, and high risk.

‡ Options for certainty in estimates are high, moderate, low, and very low. Evidence begins as high and is rated down for serious risk of bias, inconsistency, imprecision, or indirectness. We always rated down once due to uncertainty in the patient VTE risk factors and models of timing of VTE and bleeding. For fatal VTE and fatal bleeding we always rated down once for uncertainty in our case fatality rate estimates.

§The best median estimate for symptomatic splanchnic vein thrombosis is median value of reported estimates. As we did not find evidence for timing of SVT, effect of thromboprophylaxis on SVT or patient risk factors for SVT, we did not model splanchnic vein thrombosis estimates for these factors.

40. Evidence profile 40. Splenectomy, open, elective, immune thrombocytopenia (ITP): Absolute risk of venous thromboembolism and bleeding among patients not receiving prophylaxis

Certainty assessment					Summary of findings		
**No of participants (studies)**	**Risk of Bias**	**Inconsistency**	**Indirectness**	**Imprecision**	**Best (median) estimate across all risk strata (%)***	**Best (median) estimate by patient risk strata (%)†**	**Evidence certainty‡**
**Non-fatal symptomatic venous thromboembolism**							
56 (1)	Serious limitations	No serious limitations	No serious limitations	Very serious limitations	1.67	**Low: 1.33 Medium: 2.66 High: 5.32**	**Very low**
**Fatal venous thromboembolism**							
56 (1)	Serious limitations	No serious limitations	No serious limitations	Very serious limitations	0.06	**Low: 0.05 Medium: 0.1 High: 0.2**	**Very low**
**Symptomatic splanchnic vein thrombosis**							
**Non-fatal bleeding requiring reintervention**							
**Non-fatal bleeding leading to transfusion**							
56 (1)	Serious limitations	No serious limitations	No serious limitations	Very serious limitations	0.00	**0.00**	**Very low**
**Fatal bleeding**							
56 (1)	Serious limitations	No serious limitations	No serious limitations	Very serious limitations	0.00	**0.00**	**Very low**
**Bleeding leading to hemoglobin below 70g/L (7g/dL)**							

Blank spaces indicate absence of information

* Estimate represents absolute risk in percent. Our median best estimates include fatal and non-fatal events. Based on data from included studies, we estimated case fatality rates as follows: 3.6% for VTE, 3.6% for bleeding leading to reintervention, and 0.9% for bleeding leading to transfusion, and used this information to calculate outcome estimates. For instance, we multiplied the median VTE estimate by 0.964 for non-fatal VTE and by 0.036 for fatal VTE (if both reintervention and transfusion rates were available, we preferred reintervention estimates for calculation of fatal bleeding estimate).

† Risk factors included 1) age more than 75 years, 2) obesity (body mass index of 35 or more), 3) VTE in a first degree relative (parents, full siblings, or children), and 4) prior VTE. We assumed that patients with any combination of two or more risk factors had a risk ratio of 4. Using these risk factors, we then categorized risk of VTE as low, medium, and high risk.

‡ Options for certainty in estimates are high, moderate, low, and very low. Evidence begins as high and is rated down for serious risk of bias, inconsistency, imprecision, or indirectness. We always rated down once due to uncertainty in the patient VTE risk factors and models of timing of VTE and bleeding. For fatal VTE and fatal bleeding we always rated down once for uncertainty in our case fatality rate estimates.

2. Evidence profiles 41-74: risk of venous thromboembolism and bleeding among patients not receiving prophylaxis for colorectal surgery procedures: procedure, specification (such as left or total), approach (such as laparoscopic or open), indication (such as benign or malign)

41. Evidence profile 41. Abdominoperineal resection, laparoscopic: Absolute risk of venous thromboembolism and bleeding among patients not receiving prophylaxis

Quality assessment					Summary of findings		
**No of participants (studies)**	**Risk of Bias**	**Inconsistency**	**Indirectness**	**Imprecision**	**Best (median) estimate across all risk strata (%)***	**Best (median estimate) by patient risk strata (%)†**	**Overall certainty in estimates‡**
**Non-fatal symptomatic venous thromboembolism**							
2,574 (1)	No serious limitations	No serious limitations	No serious limitations	No serious limitations	1.30	**Low: 0.86 Medium: 1.73 High: 3.45**	**Moderate**
**Fatal venous thromboembolism**							
2,574 (1)	No serious limitations	No serious limitations	No serious limitations	No serious limitations	0.05	**Low: 0.03 Medium: 0.06 High: 0.13**	**Low**
**Symptomatic splanchnic vein thrombosis**							
**Non-fatal bleeding requiring reintervention**							
**Non-fatal bleeding leading to transfusion**							
2,574 (1)	No serious limitations	No serious limitations	No serious limitations	No serious limitations	4.9	**4.90**	**Moderate**
**Fatal bleeding**							
2,574 (1)	No serious limitations	No serious limitations	No serious limitations	No serious limitations	0.04	**0.04**	**Low**
**Bleeding leading to hemoglobin below 70g/L (7g/dL)**							

Blank spaces indicate absence of information

* Estimate represents absolute risk in percent. Our median best estimates include fatal and non-fatal events. Based on data from included studies, we estimated case fatality rates as follows: 3.6% for VTE, 3.6% for bleeding leading to reintervention, and 0.9% for bleeding leading to transfusion, and used this information to calculate outcome estimates. For instance, we multiplied the median VTE estimate by 0.964 for non-fatal VTE and by 0.036 for fatal VTE (if both reintervention and transfusion rates were available, we preferred reintervention estimates for calculation of fatal bleeding estimate).

† Risk factors included 1) age more than 75 years, 2) obesity (body mass index of 35 or more), 3) VTE in a first degree relative (parents, full siblings, or children), and 4) prior VTE. We assumed that patients with any combination of two or more risk factors had a risk ratio of 4. Using these risk factors, we then categorized risk of VTE as low, medium, and high risk.

‡ Options for certainty in estimates are high, moderate, low, and very low. Evidence begins as high and is rated down for serious risk of bias, inconsistency, imprecision, or indirectness. We always rated down once due to uncertainty in the patient VTE risk factors and models of timing of VTE and bleeding. For fatal VTE and fatal bleeding we always rated down once for uncertainty in our case fatality rate estimates.

42. Evidence profile 42. Abdominoperineal resection, open: Absolute risk of venous thromboembolism and bleeding among patients not receiving prophylaxis

Quality assessment					Summary of findings		
**No of participants (studies)**	**Risk of Bias**	**Inconsistency**	**Indirectness**	**Imprecision**	**Best (median) estimate across all risk strata (%)***	**Best (median estimate) by patient risk strata (%)†**	**Overall certainty in estimates‡**
**Non-fatal symptomatic venous thromboembolism**							
5,107 (1)	No serious limitations	No serious limitations	No serious limitations	No serious limitations	3.52	**Low: 2.36 Medium: 4.72 High: 9.44**	**Moderate**
**Fatal venous thromboembolism**							
5,107 (1)	No serious limitations	No serious limitations	No serious limitations	No serious limitations	0.13	**Low: 0.09 Medium: 0.18 High: 0.35**	**Low**
**Symptomatic splanchnic vein thrombosis**							
**Non-fatal bleeding requiring reintervention**							
**Non-fatal bleeding leading to transfusion**							
5,107 (1)	No serious limitations	No serious limitations	No serious limitations	No serious limitations	21.28	**21.28**	**Moderate**
**Fatal bleeding**							
5,107 (1)	No serious limitations	No serious limitations	No serious limitations	No serious limitations	0.19	**0.19**	**Low**
**Bleeding leading to hemoglobin below 70g/L (7g/dL)**							

Blank spaces indicate absence of information

* Estimate represents absolute risk in percent. Our median best estimates include fatal and non-fatal events. Based on data from included studies, we estimated case fatality rates as follows: 3.6% for VTE, 3.6% for bleeding leading to reintervention, and 0.9% for bleeding leading to transfusion, and used this information to calculate outcome estimates. For instance, we multiplied the median VTE estimate by 0.964 for non-fatal VTE and by 0.036 for fatal VTE (if both reintervention and transfusion rates were available, we preferred reintervention estimates for calculation of fatal bleeding estimate).

† Risk factors included 1) age more than 75 years, 2) obesity (body mass index of 35 or more), 3) VTE in a first degree relative (parents, full siblings, or children), and 4) prior VTE. We assumed that patients with any combination of two or more risk factors had a risk ratio of 4. Using these risk factors, we then categorized risk of VTE as low, medium, and high risk.

‡ Options for certainty in estimates are high, moderate, low, and very low. Evidence begins as high and is rated down for serious risk of bias, inconsistency, imprecision, or indirectness. We always rated down once due to uncertainty in the patient VTE risk factors and models of timing of VTE and bleeding. For fatal VTE and fatal bleeding we always rated down once for uncertainty in our case fatality rate estimates.

43. Evidence profile 43. Anterior resection, minimally-invasive: Absolute risk of venous thromboembolism and bleeding among patients not receiving prophylaxis

Quality assessment					Summary of findings		
**No of participants (studies)**	**Risk of Bias**	**Inconsistency**	**Indirectness**	**Imprecision**	**Best (median) estimate across all risk strata (%)***	**Best (median estimate) by patient risk strata (%)†**	**Overall certainty in estimates‡**
**Non-fatal symptomatic venous thromboembolism**							
35,110 (6)	No serious limitations	Serious limitations	No serious limitations	No serious limitations	1.15	**Low: 0.77 Medium: 1.53 High: 3.06**	**Low**
**Fatal venous thromboembolism**							
35,110 (6)	No serious limitations	Serious limitations	No serious limitations	No serious limitations	0.04	**Low: 0.03 Medium: 0.06 High: 0.11**	**Very Low**
**Symptomatic splanchnic vein thrombosis§**							
356 (347)	Serious limitations	No serious limitations	No serious limitations	Serious limitations	0.28	**0.28**	**Very low**
**Non-fatal bleeding requiring reintervention**							
811 (4)	Serious limitations	Serious limitations	No serious limitations	Serious limitations	1.56	**1.56**	**Very low**
**Non-fatal bleeding leading to transfusion**							
811 (4)	Serious limitations	No serious limitations	No serious limitations	Serious limitations	1.63	**1.63**	**Very low**
**Fatal bleeding**							
811 (4)	Serious limitations	Serious limitations	No serious limitations	Serious limitations	0.06	**0.06**	**Very low**
**Bleeding leading to hemoglobin below 70g/L (7g/dL)**							
356 (1)	Serious limitations	No serious limitations	No serious limitations	Serious limitations	0.98	**0.98**	**Very low**

Blank spaces indicate absence of information

* Estimate represents absolute risk in percent. Our median best estimates include fatal and non-fatal events. Based on data from included studies, we estimated case fatality rates as follows: 3.6% for VTE, 3.6% for bleeding leading to reintervention, and 0.9% for bleeding leading to transfusion, and used this information to calculate outcome estimates. For instance, we multiplied the median VTE estimate by 0.964 for non-fatal VTE and by 0.036 for fatal VTE (if both reintervention and transfusion rates were available, we preferred reintervention estimates for calculation of fatal bleeding estimate).

† Risk factors included 1) age more than 75 years, 2) obesity (body mass index of 35 or more), 3) VTE in a first degree relative (parents, full siblings, or children), and 4) prior VTE. We assumed that patients with any combination of two or more risk factors had a risk ratio of 4. Using these risk factors, we then categorized risk of VTE as low, medium, and high risk.

‡ Options for certainty in estimates are high, moderate, low, and very low. Evidence begins as high and is rated down for serious risk of bias, inconsistency, imprecision, or indirectness. We always rated down once due to uncertainty in the patient VTE risk factors and models of timing of VTE and bleeding. For fatal VTE and fatal bleeding we always rated down once for uncertainty in our case fatality rate estimates.

§ The best median estimate for symptomatic splanchnic vein thrombosis is median value of reported estimates. As we did not find evidence for timing of SVT, effect of thromboprophylaxis on SVT or patient risk factors for SVT, we did not model splanchnic vein thrombosis estimates for these factors.

44. Evidence profile 44. Anterior resection, laparoscopic: Absolute risk of venous thromboembolism and bleeding among patients not receiving prophylaxis

Quality assessment					Summary of findings		
**No of participants (studies)**	**Risk of Bias**	**Inconsistency**	**Indirectness**	**Imprecision**	**Best (median) estimate across all risk strata (%)***	**Best (median estimate) by patient risk strata (%)†**	**Overall certainty in estimates‡**
**Non-fatal symptomatic venous thromboembolism**							
34,890 (6)	No serious limitations	Serious limitations	No serious limitations	No serious limitations	1.15	**Low: 0.77 Medium: 1.53 High: 3.06**	**Low**
**Fatal venous thromboembolism**							
34,890 (6)	No serious limitations	Serious limitations	No serious limitations	No serious limitations	0.04	**Low: 0.03 Medium: 0.06 High: 0.11**	**Very Low**
**Symptomatic splanchnic vein thrombosis§**							
356 (1)	Serious limitations	No serious limitations	No serious limitations	Serious limitations	0.28	**0.28**	**Very low**
**Non-fatal bleeding requiring reintervention**							
678 (4)	Serious limitations	Serious limitations	No serious limitations	Serious limitations	1.71	**1.71**	**Very low**
**Non-fatal bleeding leading to transfusion**							
6,547 (5)	No serious limitations	No serious limitations	No serious limitations	No serious limitations	2.4	**2.40**	**Moderate**
**Fatal bleeding**							
678 (4)	Serious limitations	Serious limitations	No serious limitations	Serious limitations	0.06	**0.06**	**Very low**
**Bleeding leading to hemoglobin below 70g/L (7g/dL)**							
356 (1)	Serious limitations	No serious limitations	No serious limitations	Serious limitations	0.98	**0.98**	**Very low**

Blank spaces indicate absence of information

* Estimate represents absolute risk in percent. Our median best estimates include fatal and non-fatal events. Based on data from included studies, we estimated case fatality rates as follows: 3.6% for VTE, 3.6% for bleeding leading to reintervention, and 0.9% for bleeding leading to transfusion, and used this information to calculate outcome estimates. For instance, we multiplied the median VTE estimate by 0.964 for non-fatal VTE and by 0.036 for fatal VTE (if both reintervention and transfusion rates were available, we preferred reintervention estimates for calculation of fatal bleeding estimate).

† Risk factors included 1) age more than 75 years, 2) obesity (body mass index of 35 or more), 3) VTE in a first degree relative (parents, full siblings, or children), and 4) prior VTE. We assumed that patients with any combination of two or more risk factors had a risk ratio of 4. Using these risk factors, we then categorized risk of VTE as low, medium, and high risk.

‡ Options for certainty in estimates are high, moderate, low, and very low. Evidence begins as high and is rated down for serious risk of bias, inconsistency, imprecision, or indirectness. We always rated down once due to uncertainty in the patient VTE risk factors and models of timing of VTE and bleeding. For fatal VTE and fatal bleeding we always rated down once for uncertainty in our case fatality rate estimates.

§ The best median estimate for symptomatic splanchnic vein thrombosis is median value of reported estimates. As we did not find evidence for timing of SVT, effect of thromboprophylaxis on SVT or patient risk factors for SVT, we did not model splanchnic vein thrombosis estimates for these factors.

45. Evidence profile 45. Anterior resection, open: Absolute risk of venous thromboembolism and bleeding among patients not receiving prophylaxis

Quality assessment					Summary of findings		
**No of participants (studies)**	**Risk of Bias**	**Inconsistency**	**Indirectness**	**Imprecision**	**Best (median) estimate across all risk strata (%)***	**Best (median estimate) by patient risk strata (%)†**	**Overall certainty in estimates‡**
**Non-fatal symptomatic venous thromboembolism**							
93,593 (4)	Serious limitations	No serious limitations	No serious limitations	No serious limitations	1.43	**Low: 0.96 Medium: 1.92 High: 3.83**	**Low**
**Fatal venous thromboembolism**							
93,593 (4)	Serious limitations	No serious limitations	No serious limitations	No serious limitations	0.05	**Low: 0.04 Medium: 0.07 High: 0.14**	**Very Low**
**Symptomatic splanchnic vein thrombosis**							
**Non-fatal bleeding requiring reintervention¶**							
167 (1)	Serious limitations	No serious limitations	No serious limitations	Very serious limitations	0.00	**0.00**	**Very low**
**Non-fatal bleeding leading to transfusion**							
2,601 (2)	No serious limitations	Serious limitations	No serious limitations	No serious limitations	3.7	**3.70**	**Low**
**Fatal bleeding**							
2,601 (2)	No serious limitations	Serious limitations	No serious limitations	No serious limitations	0.00	**0.00**	**Very Low**
**Bleeding leading to hemoglobin below 70g/L (7g/dL)**							

Blank spaces indicate absence of information

* Estimate represents absolute risk in percent. Our median best estimates include fatal and non-fatal events. Based on data from included studies, we estimated case fatality rates as follows: 3.6% for VTE, 3.6% for bleeding leading to reintervention, and 0.9% for bleeding leading to transfusion, and used this information to calculate outcome estimates. For instance, we multiplied the median VTE estimate by 0.964 for non-fatal VTE and by 0.036 for fatal VTE (if both reintervention and transfusion rates were available, we preferred reintervention estimates for calculation of fatal bleeding estimate).

† Risk factors included 1) age more than 75 years, 2) obesity (body mass index of 35 or more), 3) VTE in a first degree relative (parents, full siblings, or children), and 4) prior VTE. We assumed that patients with any combination of two or more risk factors had a risk ratio of 4. Using these risk factors, we then categorized risk of VTE as low, medium, and high risk.

‡ Options for certainty in estimates are high, moderate, low, and very low. Evidence begins as high and is rated down for serious risk of bias, inconsistency, imprecision, or indirectness. We always rated down once due to uncertainty in the patient VTE risk factors and models of timing of VTE and bleeding. For fatal VTE and fatal bleeding we always rated down once for uncertainty in our case fatality rate estimates.

¶We did not include this estimate in the main article (Table 3) as the evidence is very low certainty and lacks face validity.

46. Evidence profile 46. Anterior resection, robotic: Absolute risk of venous thromboembolism and bleeding among patients not receiving prophylaxis

Quality assessment					Summary of findings		
**No of participants (studies)**	**Risk of Bias**	**Inconsistency**	**Indirectness**	**Imprecision**	**Best (median) estimate across all risk strata (%)***	**Best (median estimate) by patient risk strata (%)†**	**Overall certainty in estimates‡**
**Non-fatal symptomatic venous thromboembolism**							
220 (1)	No serious limitations	No serious limitations	No serious limitations	Serious limitations	1.12	**Low: 0.78 Medium: 1.55 High: 3.11**	**Low**
**Fatal venous thromboembolism**							
220 (1)	No serious limitations	No serious limitations	No serious limitations	Serious limitations	0.04	**Low: 0.03 Medium: 0.06 High: 0.12**	**Very Low**
**Symptomatic splanchnic vein thrombosis**							
**Non-fatal bleeding requiring reintervention**							
133 (1)	Serious limitations	No serious limitations	No serious limitations	Very serious limitations	1.42	**1.42**	**Very low**
**Non-fatal bleeding leading to transfusion**							
133 (1)	Serious limitations	No serious limitations	No serious limitations	Very serious limitations	0.73	**0.73**	**Very low**
**Fatal bleeding**							
133 (1)	Serious limitations	No serious limitations	No serious limitations	Very serious limitations	0.05	**0.05**	**Very low**
**Bleeding leading to hemoglobin below 70g/L (7g/dL)**							

Blank spaces indicate absence of information

* Estimate represents absolute risk in percent. Our median best estimates include fatal and non-fatal events. Based on data from included studies, we estimated case fatality rates as follows: 3.6% for VTE, 3.6% for bleeding leading to reintervention, and 0.9% for bleeding leading to transfusion, and used this information to calculate outcome estimates. For instance, we multiplied the median VTE estimate by 0.964 for non-fatal VTE and by 0.036 for fatal VTE (if both reintervention and transfusion rates were available, we preferred reintervention estimates for calculation of fatal bleeding estimate).

† Risk factors included 1) age more than 75 years, 2) obesity (body mass index of 35 or more), 3) VTE in a first degree relative (parents, full siblings, or children), and 4) prior VTE. We assumed that patients with any combination of two or more risk factors had a risk ratio of 4. Using these risk factors, we then categorized risk of VTE as low, medium, and high risk.

‡ Options for certainty in estimates are high, moderate, low, and very low. Evidence begins as high and is rated down for serious risk of bias, inconsistency, imprecision, or indirectness. We always rated down once due to uncertainty in the patient VTE risk factors and models of timing of VTE and bleeding. For fatal VTE and fatal bleeding we always rated down once for uncertainty in our case fatality rate estimates.

47. Evidence profile 47. Colectomy, minimally-invasive: Absolute risk of venous thromboembolism and bleeding among patients not receiving prophylaxis

Quality assessment					Summary of findings		
**No of participants (studies)**	**Risk of Bias**	**Inconsistency**	**Indirectness**	**Imprecision**	**Best (median) estimate across all risk strata (%)***	**Best (median estimate) by patient risk strata (%)†**	**Overall certainty in estimates‡**
**Non-fatal symptomatic venous thromboembolism**							
189,169 (22)	No serious limitations	Serious limitations	No serious limitations	No serious limitations	1.63	**Low: 1.13 Medium: 2.27 High: 4.54**	**Low**
**Fatal venous thromboembolism**							
189,169 (22)	No serious limitations	Serious limitations	No serious limitations	No serious limitations	0.05	**Low: 0.04 Medium: 0.08 High: 0.17**	**Very Low**
**Symptomatic splanchnic vein thrombosis§**							
1,235 (2)	Serious limitations	No serious limitations	No serious limitations	No serious limitations	0.18	**0.18**	**Low**
**Non-fatal bleeding requiring reintervention**							
3,004 (7)	No serious limitations	No serious limitations	No serious limitations	No serious limitations	0.93	**0.93**	**Moderate**
**Non-fatal bleeding leading to transfusion**							
49,708 (9)	No serious limitations	No serious limitations	No serious limitations	No serious limitations	0.6	**0.60**	**Moderate**
**Fatal bleeding**							
3,004 (7)	No serious limitations	No serious limitations	No serious limitations	No serious limitations	0.03	**0.03**	**Low**
**Bleeding leading to hemoglobin below 70g/L (7g/dL)**							
956 (2)	Serious limitations	No serious limitations	No serious limitations	Serious limitations	0.66	**0.66**	**Very low**

Blank spaces indicate absence of information
* Estimate represents absolute risk in percent. Our median best estimates include fatal and non-fatal events. Based on data from included studies, we estimated case fatality rates as follows: 3.6% for VTE, 3.6% for bleeding leading to reintervention, and 0.9% for bleeding leading to transfusion, and used this information to calculate outcome estimates. For instance, we multiplied the median VTE estimate by 0.964 for non-fatal VTE and by 0.036 for fatal VTE (if both reintervention and transfusion rates were available, we preferred reintervention estimates for calculation of fatal bleeding estimate).
† Risk factors included 1) age more than 75 years, 2) obesity (body mass index of 35 or more), 3) VTE in a first degree relative (parents, full siblings, or children), and 4) prior VTE. We assumed that patients with any combination of two or more risk factors had a risk ratio of 4. Using these risk factors, we then categorized risk of VTE as low, medium, and high risk.
‡ Options for certainty in estimates are high, moderate, low, and very low. Evidence begins as high and is rated down for serious risk of bias, inconsistency, imprecision, or indirectness. We always rated down once due to uncertainty in the patient VTE risk factors and models of timing of VTE and bleeding. For fatal VTE and fatal bleeding we always rated down once for uncertainty in our case fatality rate estimates.
§ The best median estimate for symptomatic splanchnic vein thrombosis is median value of reported estimates. As we did not find evidence for timing of SVT, effect of thromboprophylaxis on SVT or patient risk factors for SVT, we did not model splanchnic vein thrombosis estimates for these factors.

48. Evidence profile 48. Colectomy, laparoscopic: Absolute risk of venous thromboembolism and bleeding among patients not receiving prophylaxis

Quality assessment					Summary of findings		
**No of participants (studies)**	**Risk of Bias**	**Inconsistency**	**Indirectness**	**Imprecision**	**Best (median) estimate across all risk strata (%)***	**Best (median estimate) by patient risk strata (%)†**	**Overall certainty in estimates‡**
**Non-fatal symptomatic venous thromboembolism**							
187,330 (20)	No serious limitations	Serious limitations	No serious limitations	No serious limitations	1.55	**Low: 1.08 Medium: 2.16 High: 4.33**	**Low**
**Fatal venous thromboembolism**							
187,330 (20)	No serious limitations	Serious limitations	No serious limitations	No serious limitations	0.06	**Low: 0.04 Medium: 0.08 High: 0.16**	**Very Low**
**Symptomatic splanchnic vein thrombosis§**							
1,235 (2)	Serious limitations	No serious limitations	No serious limitations	No serious limitations	0.18	**0.18**	**Low**
**Non-fatal bleeding requiring reintervention**							
3,004 (7)	No serious limitations	No serious limitations	No serious limitations	No serious limitations	0.93	**0.93**	**Moderate**
**Non-fatal bleeding leading to transfusion**							
51,857 (11)	No serious limitations	No serious limitations	No serious limitations	No serious limitations	1.18	**1.18**	**Moderate**
**Fatal bleeding**							
3,004 (7)	No serious limitations	No serious limitations	No serious limitations	No serious limitations	0.03	**0.03**	**Low**
**Bleeding leading to hemoglobin below 70g/L (7g/dL)**							
956 (2)	Serious limitations	No serious limitations	No serious limitations	Serious limitations	0.66	**0.66**	**Very low**

Blank spaces indicate absence of information

* Estimate represents absolute risk in percent. Our median best estimates include fatal and non-fatal events. Based on data from included studies, we estimated case fatality rates as follows: 3.6% for VTE, 3.6% for bleeding leading to reintervention, and 0.9% for bleeding leading to transfusion, and used this information to calculate outcome estimates. For instance, we multiplied the median VTE estimate by 0.964 for non-fatal VTE and by 0.036 for fatal VTE (if both reintervention and transfusion rates were available, we preferred reintervention estimates for calculation of fatal bleeding estimate).

† Risk factors included 1) age more than 75 years, 2) obesity (body mass index of 35 or more), 3) VTE in a first degree relative (parents, full siblings, or children), and 4) prior VTE. We assumed that patients with any combination of two or more risk factors had a risk ratio of 4. Using these risk factors, we then categorized risk of VTE as low, medium, and high risk.

‡ Options for certainty in estimates are high, moderate, low, and very low. Evidence begins as high and is rated down for serious risk of bias, inconsistency, imprecision, or indirectness. We always rated down once due to uncertainty in the patient VTE risk factors and models of timing of VTE and bleeding. For fatal VTE and fatal bleeding we always rated down once for uncertainty in our case fatality rate estimates.

§ The best median estimate for symptomatic splanchnic vein thrombosis is median value of reported estimates. As we did not find evidence for timing of SVT, effect of thromboprophylaxis on SVT or patient risk factors for SVT, we did not model splanchnic vein thrombosis estimates for these factors.

¶Includes one article (Krimphove 2020) that included unspecified number of robotic procedures

49. Evidence profile 49. Colectomy, open: Absolute risk of venous thromboembolism and bleeding among patients not receiving prophylaxis

Quality assessment					Summary of findings		
**No of participants (studies)**	**Risk of Bias**	**Inconsistency**	**Indirectness**	**Imprecision**	**Best (median) estimate across all risk strata (%)***	**Best (median estimate) by patient risk strata (%)†**	**Overall certainty in estimates‡**
**Non-fatal symptomatic venous thromboembolism**							
288,439 (13)	No serious limitations	Serious limitations	No serious limitations	No serious limitations	4.23	**Low: 2.98 Medium: 5.97 High: 11.94**	**Low**
**Fatal venous thromboembolism**							
288,439 (13)	No serious limitations	Serious limitations	No serious limitations	No serious limitations	0.16	**Low: 0.11 Medium: 0.22 High: 0.45**	**Very Low**
**Symptomatic splanchnic vein thrombosis**							
**Non-fatal bleeding requiring reintervention¶**							
105,013 (4)	No serious limitations	No serious limitations	Very serious limitations	No serious limitations	0.81	**0.81**	**Very low**
**Non-fatal bleeding leading to transfusion**							
7,550 (6)	No serious limitations	Serious limitations	No serious limitations	No serious limitations	1.94	**1.94**	**Low**
**Fatal bleeding**							
7,381 (5)	No serious limitations	Serious limitations	No serious limitations	No serious limitations	0.03	**0.03**	**Very Low**
**Bleeding leading to hemoglobin below 70g/L (7g/dL)**							

Blank spaces indicate absence of information
* Estimate represents absolute risk in percent. Our median best estimates include fatal and non-fatal events. Based on data from included studies, we estimated case fatality rates as follows: 3.6% for VTE, 3.6% for bleeding leading to reintervention, and 0.9% for bleeding leading to transfusion, and used this information to calculate outcome estimates. For instance, we multiplied the median VTE estimate by 0.964 for non-fatal VTE and by 0.036 for fatal VTE (if both reintervention and transfusion rates were available, we preferred reintervention estimates for calculation of fatal bleeding estimate).
† Risk factors included 1) age more than 75 years, 2) obesity (body mass index of 35 or more), 3) VTE in a first degree relative (parents, full siblings, or children), and 4) prior VTE. We assumed that patients with any combination of two or more risk factors had a risk ratio of 4. Using these risk factors, we then categorized risk of VTE as low, medium, and high risk.
‡ Options for certainty in estimates are high, moderate, low, and very low. Evidence begins as high and is rated down for serious risk of bias, inconsistency, imprecision, or indirectness. We always rated down once due to uncertainty in the patient VTE risk factors and models of timing of VTE and bleeding. For fatal VTE and fatal bleeding we always rated down once for uncertainty in our case fatality rate estimates.
¶ As we did not have any studies providing estimates of bleeding requiring reintervention for open colectomy, we had to use indirect evidence. We calculated proportion of reinterventions that were caused by bleeding from colorectal studies included in this review that provided both total number of reinterventions and reinterventions caused by bleeding. 34/188 (18%) of reinterventions were caused by bleeding in these studies. We found 4 open colectomy articles providing total reintervention estimates and estimated that 18% of those reinterventions were caused by bleeding. We rated down twice for indirectness.

50. Evidence profile 50. Colectomy, robotic: Absolute risk of venous thromboembolism and bleeding among patients not receiving prophylaxis

Quality assessment					Summary of findings		
**No of participants (studies)**	**Risk of Bias**	**Inconsistency**	**Indirectness**	**Imprecision**	**Best (median) estimate across all risk strata (%)***	**Best (median estimate) by patient risk strata (%)†**	**Overall certainty in estimates‡**
**Non-fatal symptomatic venous thromboembolism**							
1,010 (4)	No serious limitations	No serious limitations	No serious limitations	No serious limitations	1.61	**Low: 1.15 Medium: 2.3 High: 4.59**	**Moderate**
**Fatal venous thromboembolism**							
1,010 (4)	No serious limitations	No serious limitations	No serious limitations	No serious limitations	0.06	**Low: 0.04 Medium: 0.09 High: 0.17**	**Low**
**Symptomatic splanchnic vein thrombosis**							
**Non-fatal bleeding requiring reintervention**							
**Non-fatal bleeding leading to transfusion**							
742 (2)	No serious limitations	No serious limitations	No serious limitations	Serious limitations	3.68	**3.68**	**Low**
**Fatal bleeding**							
742 (2)	No serious limitations	No serious limitations	No serious limitations	Serious limitations	0.03	**0.03**	**Very Low**
**Bleeding leading to hemoglobin below 70g/L (7g/dL)**							

Blank spaces indicate absence of information

* Estimate represents absolute risk in percent. Our median best estimates include fatal and non-fatal events. Based on data from included studies, we estimated case fatality rates as follows: 3.6% for VTE, 3.6% for bleeding leading to reintervention, and 0.9% for bleeding leading to transfusion, and used this information to calculate outcome estimates. For instance, we multiplied the median VTE estimate by 0.964 for non-fatal VTE and by 0.036 for fatal VTE (if both reintervention and transfusion rates were available, we preferred reintervention estimates for calculation of fatal bleeding estimate).

† Risk factors included 1) age more than 75 years, 2) obesity (body mass index of 35 or more), 3) VTE in a first degree relative (parents, full siblings, or children), and 4) prior VTE. We assumed that patients with any combination of two or more risk factors had a risk ratio of 4. Using these risk factors, we then categorized risk of VTE as low, medium, and high risk.

‡ Options for certainty in estimates are high, moderate, low, and very low. Evidence begins as high and is rated down for serious risk of bias, inconsistency, imprecision, or indirectness. We always rated down once due to uncertainty in the patient VTE risk factors and models of timing of VTE and bleeding. For fatal VTE and fatal bleeding we always rated down once for uncertainty in our case fatality rate estimates.

51. Evidence profile 51. Colectomy, minimally-invasive, benign: Absolute risk of venous thromboembolism and bleeding among patients not receiving prophylaxis

Quality assessment					Summary of findings		
**No of participants (studies)**	**Risk of Bias**	**Inconsistency**	**Indirectness**	**Imprecision**	**Best (median) estimate across all risk strata (%)***	**Best (median estimate) by patient risk strata (%)†**	**Overall certainty in estimates‡**
**Non-fatal symptomatic venous thromboembolism**							
54,918 (6)	No serious limitations	No serious limitations	No serious limitations	No serious limitations	0.28	**Low: 0.2 Medium: 0.41 High: 0.82**	**Moderate**
**Fatal venous thromboembolism**							
54,918 (6)	No serious limitations	No serious limitations	No serious limitations	No serious limitations	0.01	**Low: 0.01 Medium: 0.02 High: 0.03**	**Low**
**Symptomatic splanchnic vein thrombosis**							
**Non-fatal bleeding requiring reintervention**							
204 (1)	Serious limitations	No serious limitations	No serious limitations	Serious limitations	0.32	**0.32**	**Very low**
**Non-fatal bleeding leading to transfusion**							
**Fatal bleeding**							
204 (1)	Serious limitations	No serious limitations	No serious limitations	Serious limitations	0.01	**0.01**	**Very low**
**Bleeding leading to hemoglobin below 70g/L (7g/dL)**							

Blank spaces indicate absence of information

We did not find any studies including patients operated robotically providing estimates for this procedure, therefore this estimate includes only patients operated laparoscopically.

* Estimate represents absolute risk in percent. Our median best estimates include fatal and non-fatal events. Based on data from included studies, we estimated case fatality rates as follows: 3.6% for VTE, 3.6% for bleeding leading to reintervention, and 0.9% for bleeding leading to transfusion, and used this information to calculate outcome estimates. For instance, we multiplied the median VTE estimate by 0.964 for non-fatal VTE and by 0.036 for fatal VTE (if both reintervention and transfusion rates were available, we preferred reintervention estimates for calculation of fatal bleeding estimate).

† Risk factors included 1) age more than 75 years, 2) obesity (body mass index of 35 or more), 3) VTE in a first degree relative (parents, full siblings, or children), and 4) prior VTE. We assumed that patients with any combination of two or more risk factors had a risk ratio of 4. Using these risk factors, we then categorized risk of VTE as low, medium, and high risk.

‡ Options for certainty in estimates are high, moderate, low, and very low. Evidence begins as high and is rated down for serious risk of bias, inconsistency, imprecision, or indirectness. We always rated down once due to uncertainty in the patient VTE risk factors and models of timing of VTE and bleeding. For fatal VTE and fatal bleeding we always rated down once for uncertainty in our case fatality rate estimates.

52. Evidence profile 52. Colectomy, minimally-invasive, malignant: Absolute risk of venous thromboembolism and bleeding among patients not receiving prophylaxis

Quality assessment					Summary of findings		
**No of participants (studies)**	**Risk of Bias**	**Inconsistency**	**Indirectness**	**Imprecision**	**Best (median) estimate across all risk strata (%)***	**Best (median estimate) by patient risk strata (%)†**	**Overall certainty in estimates‡**
**Non-fatal symptomatic venous thromboembolism**							
53,523 (4)	No serious limitations	No serious limitations	No serious limitations	No serious limitations	1.78	**Low: 1.05 Medium: 2.1 High: 4.2**	**Moderate**
**Fatal venous thromboembolism**							
53,523 (4)	No serious limitations	No serious limitations	No serious limitations	No serious limitations	0.07	**Low: 0.04 Medium: 0.08 High: 0.16**	**Low**
**Symptomatic splanchnic vein thrombosis**							
390 (1)	Serious limitations	No serious limitations	No serious limitations	Serious limitations	0.00	**0.00**	**Very low**
**Non-fatal bleeding requiring reintervention**							
470 (2)	No serious limitations	No serious limitations	No serious limitations	Serious limitations	1.25	**1.25**	**Low**
**Non-fatal bleeding leading to transfusion**							
3,801 (4)	No serious limitations	No serious limitations	No serious limitations	No serious limitations	1.59	**1.59**	**Moderate**
**Fatal bleeding**							
470 (2)	No serious limitations	No serious limitations	No serious limitations	Serious limitations	0.05	**0.05**	**Very Low**
**Bleeding leading to hemoglobin below 70g/L (7g/dL)**							

Blank spaces indicate absence of information
We did not find any studies including patients operated robotically providing estimates for this procedure except for 89 patients included to the non-fatal bleeding leading to transfusion estimate.
* Estimate represents absolute risk in percent. Our median best estimates include fatal and non-fatal events. Based on data from included studies, we estimated case fatality rates as follows: 3.6% for VTE, 3.6% for bleeding leading to reintervention, and 0.9% for bleeding leading to transfusion, and used this information to calculate outcome estimates. For instance, we multiplied the median VTE estimate by 0.964 for non-fatal VTE and by 0.036 for fatal VTE (if both reintervention and transfusion rates were available, we preferred reintervention estimates for calculation of fatal bleeding estimate).
† Risk factors included 1) age more than 75 years, 2) obesity (body mass index of 35 or more), 3) VTE in a first degree relative (parents, full siblings, or children), and 4) prior VTE. We assumed that patients with any combination of two or more risk factors had a risk ratio of 4. Using these risk factors, we then categorized risk of VTE as low, medium, and high risk.
‡ Options for certainty in estimates are high, moderate, low, and very low. Evidence begins as high and is rated down for serious risk of bias, inconsistency, imprecision, or indirectness. We always rated down once due to uncertainty in the patient VTE risk factors and models of timing of VTE and bleeding. For fatal VTE and fatal bleeding we always rated down once for uncertainty in our case fatality rate estimates.

53. Evidence profile 53. Colectomy, minimally-invasive, inflammatory bowel disease: Absolute risk of venous thromboembolism and bleeding among patients not receiving prophylaxis

Quality assessment					Summary of findings		
**No of participants (studies)**	**Risk of Bias**	**Inconsistency**	**Indirectness**	**Imprecision**	**Best (median) estimate across all risk strata (%)***	**Best (median estimate) by patient risk strata (%)†**	**Overall certainty in estimates‡**
**Non-fatal symptomatic venous thromboembolism**							
8,955 (4)	No serious limitations	Serious limitations	No serious limitations	No serious limitations	2.04	**Low: 1.75 Medium: 3.50 High: 7.00**	**Low**
**Fatal venous thromboembolism**							
8,955 (4)	No serious limitations	Serious limitations	No serious limitations	No serious limitations	0.08	**Low: 0.07 Medium: 0.13 High: 0.26**	**Very Low**
**Symptomatic splanchnic vein thrombosis**							
**Non-fatal bleeding requiring reintervention**							
204 (1)	Serious limitations	No serious limitations	No serious limitations	Serious limitations	0.32	**0.32**	**Very low**
**Non-fatal bleeding leading to transfusion**							
112 (1)	No serious limitations	No serious limitations	No serious limitations	Very serious limitations	0	**0.00**	**Very low**
**Fatal bleeding**							
204 (1)	Serious limitations	No serious limitations	No serious limitations	Serious limitations	0.01	**0.01**	**Very low**
**Bleeding leading to hemoglobin below 70g/L (7g/dL)**							

Blank spaces indicate absence of information

Three studies including 8668 patients undergoing only laparoscopic procedures reported 2.78% median baseline risk of non-fatal symptomatic venous thromboembolism. Non-fatal bleeding requiring reintervention and bleeding leading to transfusion estimates include only patients undergoing laparoscopic procedures.

* Estimate represents absolute risk in percent. Our median best estimates include fatal and non-fatal events. Based on data from included studies, we estimated case fatality rates as follows: 3.6% for VTE, 3.6% for bleeding leading to reintervention, and 0.9% for bleeding leading to transfusion, and used this information to calculate outcome estimates. For instance, we multiplied the median VTE estimate by 0.964 for non-fatal VTE and by 0.036 for fatal VTE (if both reintervention and transfusion rates were available, we preferred reintervention estimates for calculation of fatal bleeding estimate).

† Risk factors included 1) age more than 75 years, 2) obesity (body mass index of 35 or more), 3) VTE in a first degree relative (parents, full siblings, or children), and 4) prior VTE. We assumed that patients with any combination of two or more risk factors had a risk ratio of 4. Using these risk factors, we then categorized risk of VTE as low, medium, and high risk.

‡ Options for certainty in estimates are high, moderate, low, and very low. Evidence begins as high and is rated down for serious risk of bias, inconsistency, imprecision, or indirectness. We always rated down once due to uncertainty in the patient VTE risk factors and models of timing of VTE and bleeding. For fatal VTE and fatal bleeding we always rated down once for uncertainty in our case fatality rate estimates.

54. Evidence profile 54. Colectomy, minimally-invasive, emergency: Absolute risk of venous thromboembolism and bleeding among patients not receiving prophylaxis

Quality assessment					Summary of findings		
**No of participants (studies)**	**Risk of Bias**	**Inconsistency**	**Indirectness**	**Imprecision**	**Best (median) estimate across all risk strata (%)***	**Best (median estimate) by patient risk strata (%)†**	**Overall certainty in estimates‡**
**Non-fatal symptomatic venous thromboembolism**							
2,341 (2)	No serious limitations	No serious limitations	No serious limitations	No serious limitations	4.61	**Low: 3.22 Medium: 6.44 High: 12.89**	**Moderate**
**Fatal venous thromboembolism**							
2,341 (2)	No serious limitations	No serious limitations	No serious limitations	No serious limitations	0.17	**Low: 0.12 Medium: 0.24 High: 0.48**	**Low**
**Symptomatic splanchnic vein thrombosis**							
**Non-fatal bleeding requiring reintervention**							
**Non-fatal bleeding leading to transfusion**							
**Fatal bleeding**							
**Bleeding leading to hemoglobin below 70g/L (7g/dL)**							

Blank spaces indicate absence of information
We did not find any studies including patients operated robotically providing estimates for this procedure.
* Estimate represents absolute risk in percent. Our median best estimates include fatal and non-fatal events. Based on data from included studies, we estimated case fatality rates as follows: 3.6% for VTE, 3.6% for bleeding leading to reintervention, and 0.9% for bleeding leading to transfusion, and used this information to calculate outcome estimates. For instance, we multiplied the median VTE estimate by 0.964 for non-fatal VTE and by 0.036 for fatal VTE (if both reintervention and transfusion rates were available, we preferred reintervention estimates for calculation of fatal bleeding estimate).
† Risk factors included 1) age more than 75 years, 2) obesity (body mass index of 35 or more), 3) VTE in a first degree relative (parents, full siblings, or children), and 4) prior VTE. We assumed that patients with any combination of two or more risk factors had a risk ratio of 4. Using these risk factors, we then categorized risk of VTE as low, medium, and high risk.
‡ Options for certainty in estimates are high, moderate, low, and very low. Evidence begins as high and is rated down for serious risk of bias, inconsistency, imprecision, or indirectness. We always rated down once due to uncertainty in the patient VTE risk factors and models of timing of VTE and bleeding. For fatal VTE and fatal bleeding we always rated down once for uncertainty in our case fatality rate estimates.

55. Evidence profile 55. Colectomy, open, benign: Absolute risk of venous thromboembolism and bleeding among patients not receiving prophylaxis

Quality assessment					Summary of findings		
**No of participants (studies)**	**Risk of Bias**	**Inconsistency**	**Indirectness**	**Imprecision**	**Best (median) estimate across all risk strata (%)***	**Best (median estimate) by patient risk strata (%)†**	**Overall certainty in estimates‡**
**Non-fatal symptomatic venous thromboembolism**							
151,187 (5)	Serious limitations	Serious limitations	No serious limitations	No serious limitations	2.24	**Low: 1.49 Medium: 2.99 High: 5.97**	**Very low**
**Fatal venous thromboembolism**							
151,187 (5)	Serious limitations	Serious limitations	No serious limitations	No serious limitations	0.08	**Low: 0.06 Medium: 0.11 High: 0.22**	**Very low**
**Symptomatic splanchnic vein thrombosis**							
**Non-fatal bleeding requiring reintervention**							
**Non-fatal bleeding leading to transfusion**							
1,947 (2)	No serious limitations	No serious limitations	No serious limitations	No serious limitations	2.95	**2.95**	**Moderate**
**Fatal bleeding**							
1,947 (2)	No serious limitations	No serious limitations	No serious limitations	No serious limitations	0.03	**0.03**	**Low**
**Bleeding leading to hemoglobin below 70g/L (7g/dL)**							

Blank spaces indicate absence of information

* Estimate represents absolute risk in percent. Our median best estimates include fatal and non-fatal events. Based on data from included studies, we estimated case fatality rates as follows: 3.6% for VTE, 3.6% for bleeding leading to reintervention, and 0.9% for bleeding leading to transfusion, and used this information to calculate outcome estimates. For instance, we multiplied the median VTE estimate by 0.964 for non-fatal VTE and by 0.036 for fatal VTE (if both reintervention and transfusion rates were available, we preferred reintervention estimates for calculation of fatal bleeding estimate).

† Risk factors included 1) age more than 75 years, 2) obesity (body mass index of 35 or more), 3) VTE in a first degree relative (parents, full siblings, or children), and 4) prior VTE. We assumed that patients with any combination of two or more risk factors had a risk ratio of 4. Using these risk factors, we then categorized risk of VTE as low, medium, and high risk.

‡ Options for certainty in estimates are high, moderate, low, and very low. Evidence begins as high and is rated down for serious risk of bias, inconsistency, imprecision, or indirectness. We always rated down once due to uncertainty in the patient VTE risk factors and models of timing of VTE and bleeding. For fatal VTE and fatal bleeding we always rated down once for uncertainty in our case fatality rate estimates.

56. Evidence profile 56. Colectomy, open, malignant: Absolute risk of venous thromboembolism and bleeding among patients not receiving prophylaxis

Quality assessment					Summary of findings		
**No of participants (studies)**	**Risk of Bias**	**Inconsistency**	**Indirectness**	**Imprecision**	**Best (median) estimate across all risk strata (%)***	**Best (median estimate) by patient risk strata (%)†**	**Overall certainty in estimates‡**
**Non-fatal symptomatic venous thromboembolism**							
82,643 (4)	No serious limitations	Serious limitations	No serious limitations	No serious limitations	3.32	**Low: 1.89 Medium: 3.78 High: 7.56**	**Low**
**Fatal venous thromboembolism**							
82,643 (4)	No serious limitations	Serious limitations	No serious limitations	No serious limitations	0.12	**Low: 0.07 Medium: 0.14 High: 0.28**	**Very Low**
**Symptomatic splanchnic vein thrombosis**							
**Non-fatal bleeding requiring reintervention**							
**Non-fatal bleeding leading to transfusion**							
3,246 (2)	No serious limitations	Serious limitations	No serious limitations	No serious limitations	3.59	**3.59**	**Low**
**Fatal bleeding**							
3,246 (2)	No serious limitations	Serious limitations	No serious limitations	No serious limitations	0.03	**0.03**	**Very Low**
**Bleeding leading to hemoglobin below 70g/L (7g/dL)**							

Blank spaces indicate absence of information

* Estimate represents absolute risk in percent. Our median best estimates include fatal and non-fatal events. Based on data from included studies, we estimated case fatality rates as follows: 3.6% for VTE, 3.6% for bleeding leading to reintervention, and 0.9% for bleeding leading to transfusion, and used this information to calculate outcome estimates. For instance, we multiplied the median VTE estimate by 0.964 for non-fatal VTE and by 0.036 for fatal VTE (if both reintervention and transfusion rates were available, we preferred reintervention estimates for calculation of fatal bleeding estimate).

† Risk factors included 1) age more than 75 years, 2) obesity (body mass index of 35 or more), 3) VTE in a first degree relative (parents, full siblings, or children), and 4) prior VTE. We assumed that patients with any combination of two or more risk factors had a risk ratio of 4. Using these risk factors, we then categorized risk of VTE as low, medium, and high risk.

‡ Options for certainty in estimates are high, moderate, low, and very low. Evidence begins as high and is rated down for serious risk of bias, inconsistency, imprecision, or indirectness. We always rated down once due to uncertainty in the patient VTE risk factors and models of timing of VTE and bleeding. For fatal VTE and fatal bleeding we always rated down once for uncertainty in our case fatality rate estimates.

57. Evidence profile 57. Colectomy, open, inflammatory bowel disease: Absolute risk of venous thromboembolism and bleeding among patients not receiving prophylaxis

Quality assessment					Summary of findings		
**No of participants (studies)**	**Risk of Bias**	**Inconsistency**	**Indirectness**	**Imprecision**	**Best (median) estimate across all risk strata (%)***	**Best (median estimate) by patient risk strata (%)†**	**Overall certainty in estimates‡**
**Non-fatal symptomatic venous thromboembolism**							
8,128 (2)	No serious limitations	No serious limitations	No serious limitations	No serious limitations	3.97	**Low: 3.33 Medium: 6.67 High: 13.34**	**Moderate**
**Fatal venous thromboembolism**							
8,128 (2)	No serious limitations	No serious limitations	No serious limitations	No serious limitations	0.15	**Low: 0.12 Medium: 0.25 High: 0.5**	**Low**
**Symptomatic splanchnic vein thrombosis**							
**Non-fatal bleeding requiring reintervention**							
**Non-fatal bleeding leading to transfusion**							
338 (1)	No serious limitations	No serious limitations	No serious limitations	Serious limitations	0.27	**0.27**	**Low**
**Fatal bleeding**							
338 (1)	No serious limitations	No serious limitations	No serious limitations	Serious limitations	0.00	**0.00**	**Low**
**Bleeding leading to hemoglobin below 70g/L (7g/dL)**							

Blank spaces indicate absence of information

* Estimate represents absolute risk in percent. Our median best estimates include fatal and non-fatal events. Based on data from included studies, we estimated case fatality rates as follows: 3.6% for VTE, 3.6% for bleeding leading to reintervention, and 0.9% for bleeding leading to transfusion, and used this information to calculate outcome estimates. For instance, we multiplied the median VTE estimate by 0.964 for non-fatal VTE and by 0.036 for fatal VTE (if both reintervention and transfusion rates were available, we preferred reintervention estimates for calculation of fatal bleeding estimate).

† Risk factors included 1) age more than 75 years, 2) obesity (body mass index of 35 or more), 3) VTE in a first degree relative (parents, full siblings, or children), and 4) prior VTE. We assumed that patients with any combination of two or more risk factors had a risk ratio of 4. Using these risk factors, we then categorized risk of VTE as low, medium, and high risk.

‡ Options for certainty in estimates are high, moderate, low, and very low. Evidence begins as high and is rated down for serious risk of bias, inconsistency, imprecision, or indirectness. We always rated down once due to uncertainty in the patient VTE risk factors and models of timing of VTE and bleeding. For fatal VTE and fatal bleeding we always rated down once for uncertainty in our case fatality rate estimates.

58. Evidence profile 58. Colectomy, open, emergency: Absolute risk of venous thromboembolism and bleeding among patients not receiving prophylaxis

Quality assessment					Summary of findings		
**No of participants (studies)**	**Risk of Bias**	**Inconsistency**	**Indirectness**	**Imprecision**	**Best (median) estimate across all risk strata (%)***	**Best (median estimate) by patient risk strata (%)†**	**Overall certainty in estimates‡**
**Non-fatal symptomatic venous thromboembolism**							
29,874 (3)	No serious limitations	No serious limitations	No serious limitations	No serious limitations	6.57	**Low: 4.41 Medium: 8.82 High: 17.64**	**Moderate**
**Fatal venous thromboembolism**							
29,874 (3)	No serious limitations	No serious limitations	No serious limitations	No serious limitations	0.25	**Low: 0.16 Medium: 0.33 High: 0.66**	**Low**
**Symptomatic splanchnic vein thrombosis**							
**Non-fatal bleeding requiring reintervention**							
**Non-fatal bleeding leading to transfusion**							
**Fatal bleeding**							
**Bleeding leading to hemoglobin below 70g/L (7g/dL)**							

Blank spaces indicate absence of information
* Estimate represents absolute risk in percent. Our median best estimates include fatal and non-fatal events. Based on data from included studies, we estimated case fatality rates as follows: 3.6% for VTE, 3.6% for bleeding leading to reintervention, and 0.9% for bleeding leading to transfusion, and used this information to calculate outcome estimates. For instance, we multiplied the median VTE estimate by 0.964 for non-fatal VTE and by 0.036 for fatal VTE (if both reintervention and transfusion rates were available, we preferred reintervention estimates for calculation of fatal bleeding estimate).
† Risk factors included 1) age more than 75 years, 2) obesity (body mass index of 35 or more), 3) VTE in a first degree relative (parents, full siblings, or children), and 4) prior VTE. We assumed that patients with any combination of two or more risk factors had a risk ratio of 4. Using these risk factors, we then categorized risk of VTE as low, medium, and high risk.
‡ Options for certainty in estimates are high, moderate, low, and very low. Evidence begins as high and is rated down for serious risk of bias, inconsistency, imprecision, or indirectness. We always rated down once due to uncertainty in the patient VTE risk factors and models of timing of VTE and bleeding. For fatal VTE and fatal bleeding we always rated down once for uncertainty in our case fatality rate estimates.

59. Evidence profile 59. Colectomy, left, minimally-invasive: Absolute risk of venous thromboembolism and bleeding among patients not receiving prophylaxis

Quality assessment					Summary of findings		
**No of participants (studies)**	**Risk of Bias**	**Inconsistency**	**Indirectness**	**Imprecision**	**Best (median) estimate across all risk strata (%)***	**Best (median estimate) by patient risk strata (%)†**	**Overall certainty in estimates‡**
**Non-fatal symptomatic venous thromboembolism**							
48,496 (3)	No serious limitations	No serious limitations	No serious limitations	No serious limitations	1.84	**Low: 1.22 Medium: 2.44 High: 4.88**	**Moderate**
**Fatal venous thromboembolism**							
48,496 (3)	No serious limitations	No serious limitations	No serious limitations	No serious limitations	0.07	**Low: 0.05 Medium: 0.09 High: 0.18**	**Low**
**Symptomatic splanchnic vein thrombosis§**							
585 (1)	Serious limitations	No serious limitations	No serious limitations	Serious limitations	0.51	**0.51**	**Very low**
**Non-fatal bleeding requiring reintervention**							
696 (2)	Serious limitations	No serious limitations	No serious limitations	Serious limitations	1.07	**1.07**	**Very low**
**Non-fatal bleeding leading to transfusion**							
35,190 (2)	No serious limitations	No serious limitations	No serious limitations	No serious limitations	2.32	**2.32**	**Moderate**
**Fatal bleeding**							
696 (2)	Serious limitations	No serious limitations	No serious limitations	Serious limitations	0.04	**0.04**	**Very low**
**Bleeding leading to hemoglobin below 70g/L (7g/dL)**							

Blank spaces indicate absence of information
We did not find any studies including robotic procedures for this procedure.
* Estimate represents absolute risk in percent. Our median best estimates include fatal and non-fatal events. Based on data from included studies, we estimated case fatality rates as follows: 3.6% for VTE, 3.6% for bleeding leading to reintervention, and 0.9% for bleeding leading to transfusion, and used this information to calculate outcome estimates. For instance, we multiplied the median VTE estimate by 0.964 for non-fatal VTE and by 0.036 for fatal VTE (if both reintervention and transfusion rates were available, we preferred reintervention estimates for calculation of fatal bleeding estimate).
† Risk factors included 1) age more than 75 years, 2) obesity (body mass index of 35 or more), 3) VTE in a first degree relative (parents, full siblings, or children), and 4) prior VTE. We assumed that patients with any combination of two or more risk factors had a risk ratio of 4. Using these risk factors, we then categorized risk of VTE as low, medium, and high risk.
‡ Options for certainty in estimates are high, moderate, low, and very low. Evidence begins as high and is rated down for serious risk of bias, inconsistency, imprecision, or indirectness. We always rated down once due to uncertainty in the patient VTE risk factors and models of timing of VTE and bleeding. For fatal VTE and fatal bleeding we always rated down once for uncertainty in our case fatality rate estimates.
§ The best median estimate for symptomatic splanchnic vein thrombosis is median value of reported estimates. As we did not find evidence for timing of SVT, effect of thromboprophylaxis on SVT or patient risk factors for SVT, we did not model splanchnic vein thrombosis estimates for these factors.

60. Evidence profile 60. Colectomy, right, minimally-invasive: Absolute risk of venous thromboembolism and bleeding among patients not receiving prophylaxis

Quality assessment					Summary of findings		
**No of participants (studies)**	**Risk of Bias**	**Inconsistency**	**Indirectness**	**Imprecision**	**Best (median) estimate across all risk strata (%)***	**Best (median estimate) by patient risk strata (%)†**	**Overall certainty in estimates‡**
**Non-fatal symptomatic venous thromboembolism**							
20,271 (3)	No serious limitations	No serious limitations	No serious limitations	No serious limitations	1.40	**Low: 0.91 Medium: 1.81 High: 3.62**	**Moderate**
**Fatal venous thromboembolism**							
20,271 (3)	No serious limitations	No serious limitations	No serious limitations	No serious limitations	0.05	**Low: 0.03 Medium: 0.07 High: 0.14**	**Low**
**Symptomatic splanchnic vein thrombosis**							
260 (1)	Serious limitations	No serious limitations	No serious limitations	Serious limitations	0.00	**0.00**	**Very low**
**Non-fatal bleeding requiring reintervention**							
340 (2)	Serious limitations	No serious limitations	No serious limitations	Serious limitations	1.43	**1.43**	**Very low**
**Non-fatal bleeding leading to transfusion**							
11,062 (3)	No serious limitations	No serious limitations	No serious limitations	No serious limitations	2.65	**2.65**	**Moderate**
**Fatal bleeding**							
340 (2)	Serious limitations	No serious limitations	No serious limitations	Serious limitations	0.05	**0.05**	**Very low**
**Bleeding leading to hemoglobin below 70g/L (7g/dL)**							

Blank spaces indicate absence of information
Minimally-invasive: Laparoscopic or robotic.
* Estimate represents absolute risk in percent. Our median best estimates include fatal and non-fatal events. Based on data from included studies, we estimated case fatality rates as follows: 3.6% for VTE, 3.6% for bleeding leading to reintervention, and 0.9% for bleeding leading to transfusion, and used this information to calculate outcome estimates. For instance, we multiplied the median VTE estimate by 0.964 for non-fatal VTE and by 0.036 for fatal VTE (if both reintervention and transfusion rates were available, we preferred reintervention estimates for calculation of fatal bleeding estimate).
† Risk factors included 1) age more than 75 years, 2) obesity (body mass index of 35 or more), 3) VTE in a first degree relative (parents, full siblings, or children), and 4) prior VTE. We assumed that patients with any combination of two or more risk factors had a risk ratio of 4. Using these risk factors, we then categorized risk of VTE as low, medium, and high risk.
‡ Options for certainty in estimates are high, moderate, low, and very low. Evidence begins as high and is rated down for serious risk of bias, inconsistency, imprecision, or indirectness. We always rated down once due to uncertainty in the patient VTE risk factors and models of timing of VTE and bleeding. For fatal VTE and fatal bleeding we always rated down once for uncertainty in our case fatality rate estimates.

61. Evidence profile 61. Colectomy, left, open: Absolute risk of venous thromboembolism and bleeding among patients not receiving prophylaxis

Quality assessment					Summary of findings		
**No of participants (studies)**	**Risk of Bias**	**Inconsistency**	**Indirectness**	**Imprecision**	**Best (median) estimate across all risk strata (%)***	**Best (median estimate) by patient risk strata (%)†**	**Overall certainty in estimates‡**
**Non-fatal symptomatic venous thromboembolism**							
22,603 (2)	No serious limitations	No serious limitations	No serious limitations	No serious limitations	3.89	**Low: 2.5 Medium: 5.00 High: 10.00**	**Moderate**
**Fatal venous thromboembolism**							
22,603 (2)	No serious limitations	No serious limitations	No serious limitations	No serious limitations	0.15	**Low: 0.09 Medium: 0.19 High: 0.37**	**Low**
**Symptomatic splanchnic vein thrombosis**							
**Non-fatal bleeding requiring reintervention**							
**Non-fatal bleeding leading to transfusion**							
**Fatal bleeding**							
**Bleeding leading to hemoglobin below 70g/L (7g/dL)**							

Blank spaces indicate absence of information

* Estimate represents absolute risk in percent. Our median best estimates include fatal and non-fatal events. Based on data from included studies, we estimated case fatality rates as follows: 3.6% for VTE, 3.6% for bleeding leading to reintervention, and 0.9% for bleeding leading to transfusion, and used this information to calculate outcome estimates. For instance, we multiplied the median VTE estimate by 0.964 for non-fatal VTE and by 0.036 for fatal VTE (if both reintervention and transfusion rates were available, we preferred reintervention estimates for calculation of fatal bleeding estimate).

† Risk factors included 1) age more than 75 years, 2) obesity (body mass index of 35 or more), 3) VTE in a first degree relative (parents, full siblings, or children), and 4) prior VTE. We assumed that patients with any combination of two or more risk factors had a risk ratio of 4. Using these risk factors, we then categorized risk of VTE as low, medium, and high risk.

‡ Options for certainty in estimates are high, moderate, low, and very low. Evidence begins as high and is rated down for serious risk of bias, inconsistency, imprecision, or indirectness. We always rated down once due to uncertainty in the patient VTE risk factors and models of timing of VTE and bleeding. For fatal VTE and fatal bleeding we always rated down once for uncertainty in our case fatality rate estimates.

62. Evidence profile 62. Colectomy, right, open: Absolute risk of venous thromboembolism and bleeding among patients not receiving prophylaxis

Quality assessment					Summary of findings		
**No of participants (studies)**	**Risk of Bias**	**Inconsistency**	**Indirectness**	**Imprecision**	**Best (median) estimate across all risk strata (%)***	**Best (median estimate) by patient risk strata (%)†**	**Overall certainty in estimates‡**
**Non-fatal symptomatic venous thromboembolism**							
20,650 (2)	No serious limitations	No serious limitations	No serious limitations	No serious limitations	3.23	**Low: 2.08 Medium: 4.16 High: 8.32**	**Moderate**
**Fatal venous thromboembolism**							
20,650 (2)	No serious limitations	No serious limitations	No serious limitations	No serious limitations	0.12	**Low: 0.08 Medium: 0.16 High: 0.31**	**Low**
**Symptomatic splanchnic vein thrombosis**							
**Non-fatal bleeding requiring reintervention**							
**Non-fatal bleeding leading to transfusion**							
2,048 (2)	No serious limitations	No serious limitations	No serious limitations	No serious limitations	6.95	**6.95**	**Moderate**
**Fatal bleeding**							
2,048 (2)	No serious limitations	No serious limitations	No serious limitations	No serious limitations	0.06	**0.06**	**Low**
**Bleeding leading to hemoglobin below 70g/L (7g/dL)**							

Blank spaces indicate absence of information

* Estimate represents absolute risk in percent. Our median best estimates include fatal and non-fatal events. Based on data from included studies, we estimated case fatality rates as follows: 3.6% for VTE, 3.6% for bleeding leading to reintervention, and 0.9% for bleeding leading to transfusion, and used this information to calculate outcome estimates. For instance, we multiplied the median VTE estimate by 0.964 for non-fatal VTE and by 0.036 for fatal VTE (if both reintervention and transfusion rates were available, we preferred reintervention estimates for calculation of fatal bleeding estimate).

† Risk factors included 1) age more than 75 years, 2) obesity (body mass index of 35 or more), 3) VTE in a first degree relative (parents, full siblings, or children), and 4) prior VTE. We assumed that patients with any combination of two or more risk factors had a risk ratio of 4. Using these risk factors, we then categorized risk of VTE as low, medium, and high risk.

‡ Options for certainty in estimates are high, moderate, low, and very low. Evidence begins as high and is rated down for serious risk of bias, inconsistency, imprecision, or indirectness. We always rated down once due to uncertainty in the patient VTE risk factors and models of timing of VTE and bleeding. For fatal VTE and fatal bleeding we always rated down once for uncertainty in our case fatality rate estimates.

63. Evidence profile 63. Total proctocolectomy, laparoscopic: Absolute risk of venous thromboembolism and bleeding among patients not receiving prophylaxis

Quality assessment					Summary of findings		
**No of participants (studies)**	**Risk of Bias**	**Inconsistency**	**Indirectness**	**Imprecision**	**Best (median) estimate across all risk strata (%)***	**Best (median estimate) by patient risk strata (%)†**	**Overall certainty in estimates‡**
**Non-fatal symptomatic venous thromboembolism**							
6,079 (3)	No serious limitations	Serious limitations	No serious limitations	No serious limitations	4.86	**Low: 4.16 Medium: 8.32 High: 16.64**	**Low**
**Fatal venous thromboembolism**							
6,079 (3)	No serious limitations	Serious limitations	No serious limitations	No serious limitations	0.18	**Low: 0.16 Medium: 0.31 High: 0.62**	**Very Low**
**Symptomatic splanchnic vein thrombosis§**							
367 (2)	Serious limitations	No serious limitations	No serious limitations	Serious limitations	7.86	**7.86**	**Very low**
**Non-fatal bleeding requiring reintervention**							
204 (1)	Serious limitations	No serious limitations	No serious limitations	Serious limitations	0.32	**0.32**	**Very low**
**Non-fatal bleeding leading to transfusion**							
379 (2)	No serious limitations	No serious limitations	No serious limitations	Serious limitations	2.16	**2.16**	**Low**
**Fatal bleeding**							
204 (1)	Serious limitations	No serious limitations	No serious limitations	Serious limitations	0.01	**0.01**	**Very low**
**Bleeding leading to hemoglobin below 70g/L (7g/dL)**							

Blank spaces indicate absence of information

Estimates include total proctocolectomy and/or total colectomy procedures. (Gu 2013 study with 204 patients included only total colectomy procedures, McKenna 2018 included mixed population of 1601 total proctocolectomy procedures and 4155 total colectomy procedures, Causey 2013 included 148 total proctocolectomy procedures and 112 total colectomy procedures . Other studies included only total proctocolectomy procedures).

* Estimate represents absolute risk in percent. Our median best estimates include fatal and non-fatal events. Based on data from included studies, we estimated case fatality rates as follows: 3.6% for VTE, 3.6% for bleeding leading to reintervention, and 0.9% for bleeding leading to transfusion, and used this information to calculate outcome estimates. For instance, we multiplied the median VTE estimate by 0.964 for non-fatal VTE and by 0.036 for fatal VTE (if both reintervention and transfusion rates were available, we preferred reintervention estimates for calculation of fatal bleeding estimate).

† Risk factors included 1) age more than 75 years, 2) obesity (body mass index of 35 or more), 3) VTE in a first degree relative (parents, full siblings, or children), and 4) prior VTE. We assumed that patients with any combination of two or more risk factors had a risk ratio of 4. Using these risk factors, we then categorized risk of VTE as low, medium, and high risk.

‡ Options for certainty in estimates are high, moderate, low, and very low. Evidence begins as high and is rated down for serious risk of bias, inconsistency, imprecision, or indirectness. We always rated down once due to uncertainty in the patient VTE risk factors and models of timing of VTE and bleeding. For fatal VTE and fatal bleeding we always rated down once for uncertainty in our case fatality rate estimates.

§ The best median estimate for symptomatic splanchnic vein thrombosis is median value of reported estimates. As we did not find evidence for timing of SVT, effect of thromboprophylaxis on SVT or patient risk factors for SVT, we did not model splanchnic vein thrombosis estimates for these factors.

64. Evidence profile 64. Total proctocolectomy, open: Absolute risk of venous thromboembolism and bleeding among patients not receiving prophylaxis

Quality assessment					Summary of findings		
**No of participants (studies)**	**Risk of Bias**	**Inconsistency**	**Indirectness**	**Imprecision**	**Best (median) estimate across all risk strata (%)***	**Best (median estimate) by patient risk strata (%)†**	**Overall certainty in estimates‡**
**Non-fatal symptomatic venous thromboembolism**							
8,252 (2)	No serious limitations	No serious limitations	No serious limitations	No serious limitations	5.16	**Low: 4.34 Medium: 8.68 High: 17.35**	**Moderate**
**Fatal venous thromboembolism**							
8,252 (2)	No serious limitations	No serious limitations	No serious limitations	No serious limitations	0.19	**Low: 0.16 Medium: 0.32 High: 0.65**	**Low**
**Symptomatic splanchnic vein thrombosis§**							
975 (2)	Serious limitations	No serious limitations	No serious limitations	Serious limitations	3.35	**3.35**	**Very low**
**Non-fatal bleeding requiring reintervention¶**							
72 (1)	Serious limitations	No serious limitations	No serious limitations	Very serious limitations	0.00	**0.00**	**Very low**
**Non-fatal bleeding leading to transfusion**							
589 (2)	No serious limitations	No serious limitations	No serious limitations	Serious limitations	2.76	**2.76**	**Low**
**Fatal bleeding**							
589 (2)	No serious limitations	No serious limitations	No serious limitations	Serious limitations	0.00	**0.00**	**Very Low**
**Bleeding leading to hemoglobin below 70g/L (7g/dL)**							

Blank spaces indicate absence of information

Estimates include total proctocolectomy and/or total colectomy procedures. (McKenna 2018 included mixed population of 2521 total proctocolectomy procedures and 5355 total colectomy procedures, Causey 2013 included 397 total proctocolectomy procedures and 120 total colectomy procedures. Other studies included only total proctocolectomy procedures).

* Estimate represents absolute risk in percent. Our median best estimates include fatal and non-fatal events. Based on data from included studies, we estimated case fatality rates as follows: 3.6% for VTE, 3.6% for bleeding leading to reintervention, and 0.9% for bleeding leading to transfusion, and used this information to calculate outcome estimates. For instance, we multiplied the median VTE estimate by 0.964 for non-fatal VTE and by 0.036 for fatal VTE (if both reintervention and transfusion rates were available, we preferred reintervention estimates for calculation of fatal bleeding estimate).

† Risk factors included 1) age more than 75 years, 2) obesity (body mass index of 35 or more), 3) VTE in a first degree relative (parents, full siblings, or children), and 4) prior VTE. We assumed that patients with any combination of two or more risk factors had a risk ratio of 4. Using these risk factors, we then categorized risk of VTE as low, medium, and high risk.

‡ Options for certainty in estimates are high, moderate, low, and very low. Evidence begins as high and is rated down for serious risk of bias, inconsistency, imprecision, or indirectness. We always rated down once due to uncertainty in the patient VTE risk factors and models of timing of VTE and bleeding. For fatal VTE and fatal bleeding we always rated down once for uncertainty in our case fatality rate estimates.

§ The best median estimate for symptomatic splanchnic vein thrombosis is median value of reported estimates. As we did not find evidence for timing of SVT, effect of thromboprophylaxis on SVT or patient risk factors for SVT, we did not model splanchnic vein thrombosis estimates for these factors.

¶We did not include this estimate in the main article (Table 5) as the evidence is very low certainty and lacks face validity.

65. Evidence profile 65. Total proctocolectomy, laparoscopic, benign: Absolute risk of venous thromboembolism and bleeding among patients not receiving prophylaxis

Quality assessment					Summary of findings		
**No of participants (studies)**	**Risk of Bias**	**Inconsistency**	**Indirectness**	**Imprecision**	**Best (median) estimate across all risk strata (%)***	**Best (median estimate) by patient risk strata (%)†**	**Overall certainty in estimates‡**
**Non-fatal symptomatic venous thromboembolism**							
238 (1)	No serious limitations	No serious limitations	No serious limitations	Serious limitations	4.83	**Low: 4.09 Medium: 8.19 High: 16.38**	**Low**
**Fatal venous thromboembolism**							
238 (1)	No serious limitations	No serious limitations	No serious limitations	Serious limitations	0.18	**Low: 0.15 Medium: 0.31 High: 0.61**	**Very Low**
**Symptomatic splanchnic vein thrombosis§**							
119 (1)	Serious limitations	No serious limitations	No serious limitations	Very serious limitations	10.08	**10.08**	**Very low**
**Non-fatal bleeding requiring reintervention**							
**Non-fatal bleeding leading to transfusion**							
119 (1)	Serious limitations	No serious limitations	No serious limitations	Very serious limitations	3.96	**3.96**	**Very low**
**Fatal bleeding**							
119 (1)	Serious limitations	No serious limitations	No serious limitations	Very serious limitations	0.04	**0.04**	**Very low**
**Bleeding leading to hemoglobin below 70g/L (7g/dL)**							

Blank spaces indicate absence of information

Estimates include total proctocolectomy and/or total colectomy procedures. (McKenna 2018 included population of 238 total colectomy procedures. Duraes 2018 included 119 total proctocolectomy procedures.)

* Estimate represents absolute risk in percent. Our median best estimates include fatal and non-fatal events. Based on data from included studies, we estimated case fatality rates as follows: 3.6% for VTE, 3.6% for bleeding leading to reintervention, and 0.9% for bleeding leading to transfusion, and used this information to calculate outcome estimates. For instance, we multiplied the median VTE estimate by 0.964 for non-fatal VTE and by 0.036 for fatal VTE (if both reintervention and transfusion rates were available, we preferred reintervention estimates for calculation of fatal bleeding estimate).

† Risk factors included 1) age more than 75 years, 2) obesity (body mass index of 35 or more), 3) VTE in a first degree relative (parents, full siblings, or children), and 4) prior VTE. We assumed that patients with any combination of two or more risk factors had a risk ratio of 4. Using these risk factors, we then categorized risk of VTE as low, medium, and high risk.

‡ Options for certainty in estimates are high, moderate, low, and very low. Evidence begins as high and is rated down for serious risk of bias, inconsistency, imprecision, or indirectness. We always rated down once due to uncertainty in the patient VTE risk factors and models of timing of VTE and bleeding. For fatal VTE and fatal bleeding we always rated down once for uncertainty in our case fatality rate estimates.

§ The best median estimate for symptomatic splanchnic vein thrombosis is median value of reported estimates. As we did not find evidence for timing of SVT, effect of thromboprophylaxis on SVT or patient risk factors for SVT, we did not model splanchnic vein thrombosis estimates for these factors.

66. Evidence profile 66. Total proctocolectomy, laparoscopic, malignant: Absolute risk of venous thromboembolism and bleeding among patients not receiving prophylaxis

Quality assessment					Summary of findings		
**No of participants (studies)**	**Risk of Bias**	**Inconsistency**	**Indirectness**	**Imprecision**	**Best (median) estimate across all risk strata (%)***	**Best (median estimate) by patient risk strata (%)†**	**Overall certainty in estimates‡**
**Non-fatal symptomatic venous thromboembolism**							
1,307 (1)	No serious limitations	No serious limitations	No serious limitations	No serious limitations	3.27	**Low: 2.28 Medium: 4.56 High: 9.13**	**Moderate**
**Fatal venous thromboembolism**							
1,307 (1)	No serious limitations	No serious limitations	No serious limitations	No serious limitations	0.12	**Low: 0.09 Medium: 0.17 High: 0.34**	**Low**
**Symptomatic splanchnic vein thrombosis**							
**Non-fatal bleeding requiring reintervention**							
**Non-fatal bleeding leading to transfusion**							
**Fatal bleeding**							
**Bleeding leading to hemoglobin below 70g/L (7g/dL)**							

Blank spaces indicate absence of information
Estimates include total proctocolectomy and total colectomy procedures. (McKenna 2018 included population of 407 total proctocolectomy procedures and 900 total colectomy procedures)
* Estimate represents absolute risk in percent. Our median best estimates include fatal and non-fatal events. Based on data from included studies, we estimated case fatality rates as follows: 3.6% for VTE, 3.6% for bleeding leading to reintervention, and 0.9% for bleeding leading to transfusion, and used this information to calculate outcome estimates. For instance, we multiplied the median VTE estimate by 0.964 for non-fatal VTE and by 0.036 for fatal VTE (if both reintervention and transfusion rates were available, we preferred reintervention estimates for calculation of fatal bleeding estimate).
† Risk factors included 1) age more than 75 years, 2) obesity (body mass index of 35 or more), 3) VTE in a first degree relative (parents, full siblings, or children), and 4) prior VTE. We assumed that patients with any combination of two or more risk factors had a risk ratio of 4. Using these risk factors, we then categorized risk of VTE as low, medium, and high risk.
‡ Options for certainty in estimates are high, moderate, low, and very low. Evidence begins as high and is rated down for serious risk of bias, inconsistency, imprecision, or indirectness. We always rated down once due to uncertainty in the patient VTE risk factors and models of timing of VTE and bleeding. For fatal VTE and fatal bleeding we always rated down once for uncertainty in our case fatality rate estimates.

67. Evidence profile 67. Total proctocolectomy, laparoscopic, inflammatory bowel disease: Absolute risk of venous thromboembolism and bleeding among patients not receiving prophylaxis

Quality assessment					Summary of findings		
**No of participants (studies)**	**Risk of Bias**	**Inconsistency**	**Indirectness**	**Imprecision**	**Best (median) estimate across all risk strata (%)***	**Best (median estimate) by patient risk strata (%)†**	**Overall certainty in estimates‡**
**Non-fatal symptomatic venous thromboembolism**							
4,055 (1)	No serious limitations	No serious limitations	No serious limitations	No serious limitations	5.10	**Low: 4.33 Medium: 8.65 High: 17.31**	**Moderate**
**Fatal venous thromboembolism**							
4,055 (1)	No serious limitations	No serious limitations	No serious limitations	No serious limitations	0.19	**Low: 0.16 Medium: 0.32 High: 0.65**	**Low**
**Symptomatic splanchnic vein thrombosis**							
**Non-fatal bleeding requiring reintervention**							
**Non-fatal bleeding leading to transfusion**							
148 (1)	No serious limitations	No serious limitations	No serious limitations	Very serious limitations	0.75	**0.75**	**Very low**
**Fatal bleeding**							
148 (1)	No serious limitations	No serious limitations	No serious limitations	Very serious limitations	0.01	**0.01**	**Very low**
**Bleeding leading to hemoglobin below 70g/L (7g/dL)**							

Blank spaces indicate absence of information

Estimates include total proctocolectomy and/or total colectomy procedures. (McKenna 2018 included mixed population of 1194 total proctocolectomy procedures and 2861 total colectomy procedures. Other studies included only total proctocolectomy procedures).

* Estimate represents absolute risk in percent. Our median best estimates include fatal and non-fatal events. Based on data from included studies, we estimated case fatality rates as follows: 3.6% for VTE, 3.6% for bleeding leading to reintervention, and 0.9% for bleeding leading to transfusion, and used this information to calculate outcome estimates. For instance, we multiplied the median VTE estimate by 0.964 for non-fatal VTE and by 0.036 for fatal VTE (if both reintervention and transfusion rates were available, we preferred reintervention estimates for calculation of fatal bleeding estimate).

† Risk factors included 1) age more than 75 years, 2) obesity (body mass index of 35 or more), 3) VTE in a first degree relative (parents, full siblings, or children), and 4) prior VTE. We assumed that patients with any combination of two or more risk factors had a risk ratio of 4. Using these risk factors, we then categorized risk of VTE as low, medium, and high risk.

‡ Options for certainty in estimates are high, moderate, low, and very low. Evidence begins as high and is rated down for serious risk of bias, inconsistency, imprecision, or indirectness. We always rated down once due to uncertainty in the patient VTE risk factors and models of timing of VTE and bleeding. For fatal VTE and fatal bleeding we always rated down once for uncertainty in our case fatality rate estimates.

68. Evidence profile 68. Total proctocolectomy, open, benign: Absolute risk of venous thromboembolism and bleeding among patients not receiving prophylaxis

Quality assessment					Summary of findings		
**No of participants (studies)**	**Risk of Bias**	**Inconsistency**	**Indirectness**	**Imprecision**	**Best (median) estimate across all risk strata (%)***	**Best (median estimate) by patient risk strata (%)†**	**Overall certainty in estimates‡**
**Non-fatal symptomatic venous thromboembolism**							
708 (1)	No serious limitations	No serious limitations	No serious limitations	Serious limitations	5.83	**Low: 3.6 Medium: 7.2 High: 14.4**	**Low**
**Fatal venous thromboembolism**							
708 (1)	No serious limitations	No serious limitations	No serious limitations	Serious limitations	0.22	**Low: 0.13 Medium: 0.27 High: 0.54**	**Very Low**
**Symptomatic splanchnic vein thrombosis**							
**Non-fatal bleeding requiring reintervention**							
**Non-fatal bleeding leading to transfusion**							
**Fatal bleeding**							
**Bleeding leading to hemoglobin below 70g/L (7g/dL)**							

Blank spaces indicate absence of information

Estimates include total proctocolectomy and total colectomy procedures. (McKenna 2018 included population of 193 total proctocolectomy procedures and 515 total colectomy procedures)

* Estimate represents absolute risk in percent. Our median best estimates include fatal and non-fatal events. Based on data from included studies, we estimated case fatality rates as follows: 3.6% for VTE, 3.6% for bleeding leading to reintervention, and 0.9% for bleeding leading to transfusion, and used this information to calculate outcome estimates. For instance, we multiplied the median VTE estimate by 0.964 for non-fatal VTE and by 0.036 for fatal VTE (if both reintervention and transfusion rates were available, we preferred reintervention estimates for calculation of fatal bleeding estimate).

† Risk factors included 1) age more than 75 years, 2) obesity (body mass index of 35 or more), 3) VTE in a first degree relative (parents, full siblings, or children), and 4) prior VTE. We assumed that patients with any combination of two or more risk factors had a risk ratio of 4. Using these risk factors, we then categorized risk of VTE as low, medium, and high risk.

‡ Options for certainty in estimates are high, moderate, low, and very low. Evidence begins as high and is rated down for serious risk of bias, inconsistency, imprecision, or indirectness. We always rated down once due to uncertainty in the patient VTE risk factors and models of timing of VTE and bleeding. For fatal VTE and fatal bleeding we always rated down once for uncertainty in our case fatality rate estimates.

69. Evidence profile 69. Total proctocolectomy, open, malignant: Absolute risk of venous thromboembolism and bleeding among patients not receiving prophylaxis

Quality assessment					Summary of findings		
**No of participants (studies)**	**Risk of Bias**	**Inconsistency**	**Indirectness**	**Imprecision**	**Best (median) estimate across all risk strata (%)***	**Best (median estimate) by patient risk strata (%)†**	**Overall certainty in estimates‡**
**Non-fatal symptomatic venous thromboembolism**							
2,410 (1)	No serious limitations	No serious limitations	No serious limitations	No serious limitations	4.51	**Low: 3.01 Medium: 6.02 High: 12.03**	**Moderate**
**Fatal venous thromboembolism**							
2,410 (1)	No serious limitations	No serious limitations	No serious limitations	No serious limitations	0.17	**Low: 0.11 Medium: 0.22 High: 0.45**	**Low**
**Symptomatic splanchnic vein thrombosis**							
**Non-fatal bleeding requiring reintervention**							
**Non-fatal bleeding leading to transfusion**							
**Fatal bleeding**							
**Bleeding leading to hemoglobin below 70g/L (7g/dL)**							

Blank spaces indicate absence of information

Estimates include total proctocolectomy and total colectomy procedures. (McKenna 2018 included population of 890 total proctocolectomy procedures and 1520 total colectomy procedures)

* Estimate represents absolute risk in percent. Our median best estimates include fatal and non-fatal events. Based on data from included studies, we estimated case fatality rates as follows: 3.6% for VTE, 3.6% for bleeding leading to reintervention, and 0.9% for bleeding leading to transfusion, and used this information to calculate outcome estimates. For instance, we multiplied the median VTE estimate by 0.964 for non-fatal VTE and by 0.036 for fatal VTE (if both reintervention and transfusion rates were available, we preferred reintervention estimates for calculation of fatal bleeding estimate).

† Risk factors included 1) age more than 75 years, 2) obesity (body mass index of 35 or more), 3) VTE in a first degree relative (parents, full siblings, or children), and 4) prior VTE. We assumed that patients with any combination of two or more risk factors had a risk ratio of 4. Using these risk factors, we then categorized risk of VTE as low, medium, and high risk.

‡ Options for certainty in estimates are high, moderate, low, and very low. Evidence begins as high and is rated down for serious risk of bias, inconsistency, imprecision, or indirectness. We always rated down once due to uncertainty in the patient VTE risk factors and models of timing of VTE and bleeding. For fatal VTE and fatal bleeding we always rated down once for uncertainty in our case fatality rate estimates.

70. Evidence profile 70. Total proctocolectomy, open, inflammatory bowel disease: Absolute risk of venous thromboembolism and bleeding among patients not receiving prophylaxis

Quality assessment					Summary of findings		
**No of participants (studies)**	**Risk of Bias**	**Inconsistency**	**Indirectness**	**Imprecision**	**Best (median) estimate across all risk strata (%)***	**Best (median estimate) by patient risk strata (%)†**	**Overall certainty in estimates‡**
**Non-fatal symptomatic venous thromboembolism**							
3,202 (2)	No serious limitations	No serious limitations	No serious limitations	No serious limitations	4.59	**Low: 3.86 Medium: 7.72 High: 15.44**	**Moderate**
**Fatal venous thromboembolism**							
3,202 (2)	No serious limitations	No serious limitations	No serious limitations	No serious limitations	0.17	**Low: 0.14 Medium: 0.29 High: 0.58**	**Low**
**Symptomatic splanchnic vein thrombosis§**							
975 (2)	Serious limitations	No serious limitations	No serious limitations	Serious limitations	3.35	**3.35**	**Very low**
**Non-fatal bleeding requiring reintervention¶**							
72 (1)	Serious limitations	No serious limitations	No serious limitations	Very serious limitations	0.00	**0.00**	**Very low**
**Non-fatal bleeding leading to transfusion**							
469 (2)	No serious limitations	Serious limitations	No serious limitations	Serious limitations	3.7	**3.70**	**Very low**
**Fatal bleeding**							
469 (2)	No serious limitations	Serious limitations	No serious limitations	Serious limitations	0.00	**0.00**	**Very low**
**Bleeding leading to hemoglobin below 70g/L (7g/dL)**							

Blank spaces indicate absence of information

Estimates include total proctocolectomy and total colectomy procedures. (McKenna 2018 included population of 1440 total proctocolectomy procedures and 1619 total colectomy procedures. Other studies included only total proctocolectomy procedures)

* Estimate represents absolute risk in percent. Our median best estimates include fatal and non-fatal events. Based on data from included studies, we estimated case fatality rates as follows: 3.6% for VTE, 3.6% for bleeding leading to reintervention, and 0.9% for bleeding leading to transfusion, and used this information to calculate outcome estimates. For instance, we multiplied the median VTE estimate by 0.964 for non-fatal VTE and by 0.036 for fatal VTE (if both reintervention and transfusion rates were available, we preferred reintervention estimates for calculation of fatal bleeding estimate).

† Risk factors included 1) age more than 75 years, 2) obesity (body mass index of 35 or more), 3) VTE in a first degree relative (parents, full siblings, or children), and 4) prior VTE. We assumed that patients with any combination of two or more risk factors had a risk ratio of 4. Using these risk factors, we then categorized risk of VTE as low, medium, and high risk.

‡ Options for certainty in estimates are high, moderate, low, and very low. Evidence begins as high and is rated down for serious risk of bias, inconsistency, imprecision, or indirectness. We always rated down once due to uncertainty in the patient VTE risk factors and models of timing of VTE and bleeding. For fatal VTE and fatal bleeding we always rated down once for uncertainty in our case fatality rate estimates.

§ The best median estimate for symptomatic splanchnic vein thrombosis is median value of reported estimates. As we did not find evidence for timing of SVT, effect of thromboprophylaxis on SVT or patient risk factors for SVT, we did not model splanchnic vein thrombosis estimates for these factors.

¶We did not include this estimate in the main article (Table 5) as the evidence is very low certainty and lacks face validity.

71. Evidence profile 71. Total proctocolectomy, open, emergency: Absolute risk of venous thromboembolism and bleeding among patients not receiving prophylaxis

Quality assessment					Summary of findings		
**No of participants (studies)**	**Risk of Bias**	**Inconsistency**	**Indirectness**	**Imprecision**	**Best (median) estimate across all risk strata (%)***	**Best (median estimate) by patient risk strata (%)†**	**Overall certainty in estimates‡**
**Non-fatal symptomatic venous thromboembolism**							
1,932 (1)	No serious limitations	No serious limitations	No serious limitations	No serious limitations	9.68	**Low: 5.65 Medium: 11.3 High: 22.61**	**Moderate**
**Fatal venous thromboembolism**							
1,932 (1)	No serious limitations	No serious limitations	No serious limitations	No serious limitations	0.36	**Low: 0.21 Medium: 0.42 High: 0.84**	**Low**
**Symptomatic splanchnic vein thrombosis**							
**Non-fatal bleeding requiring reintervention**							
**Non-fatal bleeding leading to transfusion**							
**Fatal bleeding**							
**Bleeding leading to hemoglobin below 70g/L (7g/dL)**							

Blank spaces indicate absence of information

Estimates include total proctocolectomy and total colectomy procedures. (McKenna 2018 included population of 231 total proctocolectomy procedures and 1701 total colectomy procedures)

* Estimate represents absolute risk in percent. Our median best estimates include fatal and non-fatal events. Based on data from included studies, we estimated case fatality rates as follows: 3.6% for VTE, 3.6% for bleeding leading to reintervention, and 0.9% for bleeding leading to transfusion, and used this information to calculate outcome estimates. For instance, we multiplied the median VTE estimate by 0.964 for non-fatal VTE and by 0.036 for fatal VTE (if both reintervention and transfusion rates were available, we preferred reintervention estimates for calculation of fatal bleeding estimate).

† Risk factors included 1) age more than 75 years, 2) obesity (body mass index of 35 or more), 3) VTE in a first degree relative (parents, full siblings, or children), and 4) prior VTE. We assumed that patients with any combination of two or more risk factors had a risk ratio of 4. Using these risk factors, we then categorized risk of VTE as low, medium, and high risk.

‡ Options for certainty in estimates are high, moderate, low, and very low. Evidence begins as high and is rated down for serious risk of bias, inconsistency, imprecision, or indirectness. We always rated down once due to uncertainty in the patient VTE risk factors and models of timing of VTE and bleeding. For fatal VTE and fatal bleeding we always rated down once for uncertainty in our case fatality rate estimates.

72. Evidence profile 72. Rectopexy, laparoscopic: Absolute risk of venous thromboembolism and bleeding among patients not receiving prophylaxis

**Quality assessment**					**Summary of findings**		
**No of participants (studies)**	**Risk of Bias**	**Inconsistency**	**Indirectness**	**Imprecision**	**Best (median) estimate across all risk strata (%)***	**Best (median estimate) by patient risk strata (%)†**	**Overall certainty in estimates‡**
**Non-fatal symptomatic venous thromboembolism**							
3,350 (1)	No serious limitations	No serious limitations	No serious limitations	No serious limitations	0.38	**Low: 0.25 Medium: 0.5 High: 1.01**	**Moderate**
**Fatal venous thromboembolism**							
3,350 (1)	No serious limitations	No serious limitations	No serious limitations	No serious limitations	0.01	**Low: 0.01 Medium: 0.02 High: 0.04**	**Low**
**Symptomatic splanchnic vein thrombosis**							
**Non-fatal bleeding requiring reintervention**							
**Non-fatal bleeding leading to transfusion**							
3,350 (1)	No serious limitations	No serious limitations	No serious limitations	No serious limitations	0.90	**0.90**	**Moderate**
**Fatal bleeding**							
3,350 (1)	No serious limitations	No serious limitations	No serious limitations	No serious limitations	0.01	**0.01**	**Low**
**Bleeding leading to hemoglobin below 70g/L (7g/dL)**							

Blank spaces indicate absence of information
* Estimate represents absolute risk in percent. Our median best estimates include fatal and non-fatal events. Based on data from included studies, we estimated case fatality rates as follows: 3.6% for VTE, 3.6% for bleeding leading to reintervention, and 0.9% for bleeding leading to transfusion, and used this information to calculate outcome estimates. For instance, we multiplied the median VTE estimate by 0.964 for non-fatal VTE and by 0.036 for fatal VTE (if both reintervention and transfusion rates were available, we preferred reintervention estimates for calculation of fatal bleeding estimate).
† Risk factors included 1) age more than 75 years, 2) obesity (body mass index of 35 or more), 3) VTE in a first degree relative (parents, full siblings, or children), and 4) prior VTE. We assumed that patients with any combination of two or more risk factors had a risk ratio of 4. Using these risk factors, we then categorized risk of VTE as low, medium, and high risk.
‡ Options for certainty in estimates are high, moderate, low, and very low. Evidence begins as high and is rated down for serious risk of bias, inconsistency, imprecision, or indirectness. We always rated down once due to uncertainty in the patient VTE risk factors and models of timing of VTE and bleeding. For fatal VTE and fatal bleeding we always rated down once for uncertainty in our case fatality rate estimates.

73. Evidence profile 73. Rectopexy, open: Absolute risk of venous thromboembolism and bleeding among patients not receiving prophylaxis

Quality assessment					Summary of findings		
**No of participants (studies)**	**Risk of Bias**	**Inconsistency**	**Indirectness**	**Imprecision**	**Best (median) estimate across all risk strata (%)***	**Best (median estimate) by patient risk strata (%)†**	**Overall certainty in estimates‡**
**Non-fatal symptomatic venous thromboembolism**							
3,599 (1)	No serious limitations	No serious limitations	No serious limitations	No serious limitations	0.60	**Low: 0.3 Medium: 0.6 High: 1.2**	**Moderate**
**Fatal venous thromboembolism**							
3,599 (1)	No serious limitations	No serious limitations	No serious limitations	No serious limitations	0.02	**Low: 0.01 Medium: 0.02 High: 0.04**	**Low**
**Symptomatic splanchnic vein thrombosis**							
**Non-fatal bleeding requiring reintervention**							
**Non-fatal bleeding leading to transfusion**							
3,599 (1)	No serious limitations	No serious limitations	No serious limitations	No serious limitations	1.75	**1.75**	**Moderate**
**Fatal bleeding**							
**Bleeding leading to hemoglobin below 70g/L (7g/dL)**							

Blank spaces indicate absence of information

* Estimate represents absolute risk in percent. Our median best estimates include fatal and non-fatal events. Based on data from included studies, we estimated case fatality rates as follows: 3.6% for VTE, 3.6% for bleeding leading to reintervention, and 0.9% for bleeding leading to transfusion, and used this information to calculate outcome estimates. For instance, we multiplied the median VTE estimate by 0.964 for non-fatal VTE and by 0.036 for fatal VTE (if both reintervention and transfusion rates were available, we preferred reintervention estimates for calculation of fatal bleeding estimate).

† Risk factors included 1) age more than 75 years, 2) obesity (body mass index of 35 or more), 3) VTE in a first degree relative (parents, full siblings, or children), and 4) prior VTE. We assumed that patients with any combination of two or more risk factors had a risk ratio of 4. Using these risk factors, we then categorized risk of VTE as low, medium, and high risk.

‡ Options for certainty in estimates are high, moderate, low, and very low. Evidence begins as high and is rated down for serious risk of bias, inconsistency, imprecision, or indirectness. We always rated down once due to uncertainty in the patient VTE risk factors and models of timing of VTE and bleeding. For fatal VTE and fatal bleeding we always rated down once for uncertainty in our case fatality rate estimates.

74. Evidence profile 74. Rectopexy, perineal: Absolute risk of venous thromboembolism and bleeding among patients not receiving prophylaxis

Quality assessment					Summary of findings		
**No of participants (studies)**	**Risk of Bias**	**Inconsistency**	**Indirectness**	**Imprecision**	**Best (median) estimate across all risk strata (%)***	**Best (median estimate) by patient risk strata (%)†**	**Overall certainty in estimates‡**
**Non-fatal symptomatic venous thromboembolism**							
5,384 (2)	No serious limitations	No serious limitations	No serious limitations	No serious limitations	1.15	**Low: 0.55 Medium: 1.1 High: 2.19**	**Moderate**
**Fatal venous thromboembolism**							
5,384 (2)	No serious limitations	No serious limitations	No serious limitations	No serious limitations	0.04	**Low: 0.02 Medium: 0.04 High: 0.08**	**Low**
**Symptomatic splanchnic vein thrombosis**							
**Non-fatal bleeding requiring reintervention**							
**Non-fatal bleeding leading to transfusion**							
5,334 (2)	No serious limitations	No serious limitations	No serious limitations	No serious limitations	0.45	**0.45**	**Moderate**
**Fatal bleeding**							
5,334 (2)	No serious limitations	No serious limitations	No serious limitations	No serious limitations	0.01	**0.01**	**Low**
**Bleeding leading to hemoglobin below 70g/L (7g/dL)**							

Blank spaces indicate absence of information

* Estimate represents absolute risk in percent. Our median best estimates include fatal and non-fatal events. Based on data from included studies, we estimated case fatality rates as follows: 3.6% for VTE, 3.6% for bleeding leading to reintervention, and 0.9% for bleeding leading to transfusion, and used this information to calculate outcome estimates. For instance, we multiplied the median VTE estimate by 0.964 for non-fatal VTE and by 0.036 for fatal VTE (if both reintervention and transfusion rates were available, we preferred reintervention estimates for calculation of fatal bleeding estimate).

† Risk factors included 1) age more than 75 years, 2) obesity (body mass index of 35 or more), 3) VTE in a first degree relative (parents, full siblings, or children), and 4) prior VTE. We assumed that patients with any combination of two or more risk factors had a risk ratio of 4. Using these risk factors, we then categorized risk of VTE as low, medium, and high risk.

‡ Options for certainty in estimates are high, moderate, low, and very low. Evidence begins as high and is rated down for serious risk of bias, inconsistency, imprecision, or indirectness. We always rated down once due to uncertainty in the patient VTE risk factors and models of timing of VTE and bleeding. For fatal VTE and fatal bleeding we always rated down once for uncertainty in our case fatality rate estimates.

3. Evidence profiles 75-128: risk of venous thromboembolism and bleeding among patients not receiving prophylaxis for upper-gastrointestinal and hepatopancreatobiliary surgery procedures: procedure, approach (such as laparoscopic or open), specification (such as minor or major), indication (such as benign or malign)

75. Evidence profile 75. Distal pancreatectomy, minimally-invasive: Absolute risk of venous thromboembolism and bleeding among patients not receiving prophylaxis

Quality assessment					Summary of findings		
**No of participants (studies)**	**Risk of Bias**	**Inconsistency**	**Indirectness**	**Imprecision**	**Best (median) estimate across all risk strata (%)***	**Best (median estimate) by patient risk strata (%)†**	**Overall certainty in estimates‡**
**Non-fatal symptomatic venous thromboembolism**							
1,858 (2)	No serious limitations	No serious limitations	No serious limitations	No serious limitations	2.45¶	**Low: 1.75 Medium: 3.51 High: 7.02**	**Moderate**
**Fatal venous thromboembolism**							
1,858 (2)	No serious limitations	No serious limitations	No serious limitations	No serious limitations	0.09	**Low: 0.07 Medium: 0.13 High: 0.26**	**Low**
**Symptomatic splanchnic vein thrombosis§**							
353 (1)	Serious limitations	No serious limitations	No serious limitations	Serious limitations	0.85	**0.85**	**Very low**
**Non-fatal bleeding requiring reintervention**							
1,137 (4)	Serious limitations	No serious limitations	No serious limitations	No serious limitations	0.86	**0.86**	**Low**
**Non-fatal bleeding leading to transfusion**							
2,136 (2)	No serious limitations	No serious limitations	No serious limitations	No serious limitations	4.35	**4.35**	**Moderate**
**Fatal bleeding**							
1,137 (4)	Serious limitations	No serious limitations	No serious limitations	No serious limitations	0.03	**0.03**	**Very low**
**Bleeding leading to hemoglobin below 70g/L (7g/dL)**							

Blank spaces indicate absence of information

* Estimate represents absolute risk in percent. Our median best estimates include fatal and non-fatal events. Based on data from included studies, we estimated case fatality rates as follows: 3.6% for VTE, 3.6% for bleeding leading to reintervention, and 0.9% for bleeding leading to transfusion, and used this information to calculate outcome estimates. For instance, we multiplied the median VTE estimate by 0.964 for non-fatal VTE and by 0.036 for fatal VTE (if both reintervention and transfusion rates were available, we preferred reintervention estimates for calculation of fatal bleeding estimate).

† Risk factors included 1) age more than 75 years, 2) obesity (body mass index of 35 or more), 3) VTE in a first degree relative (parents, full siblings, or children), and 4) prior VTE. We assumed that patients with any combination of two or more risk factors had a risk ratio of 4. Using these risk factors, we then categorized risk of VTE as low, medium, and high risk.

‡ Options for certainty in estimates are high, moderate, low, and very low. Evidence begins as high and is rated down for serious risk of bias, inconsistency, imprecision, or indirectness. We always rated down once due to uncertainty in the patient VTE risk factors and models of timing of VTE and bleeding. For fatal VTE and fatal bleeding we always rated down once for uncertainty in our case fatality rate estimates.

§The best median estimate for symptomatic splanchnic vein thrombosis is median value of reported estimates. As we did not find evidence for timing of SVT, effect of thromboprophylaxis on SVT or patient risk factors for SVT, we did not model splanchnic vein thrombosis estimates for these factors.

¶We had only two studies reporting VTE, one of them large (1789 patients) with low risk of bias, and one of them small (69 patients) with high risk of bias and zero events. We used mean instead of median to give not too big emphasis on small study with high risk of bias.

76. Evidence profile 76. Distal pancreatectomy, laparoscopic: Absolute risk of venous thromboembolism and bleeding among patients not receiving prophylaxis

Quality assessment					Summary of findings		
**No of participants (studies)**	**Risk of Bias**	**Inconsistency**	**Indirectness**	**Imprecision**	**Best (median) estimate across all risk strata (%)***	**Best (median estimate) by patient risk strata (%)†**	**Overall certainty in estimates‡**
**Non-fatal symptomatic venous thromboembolism**							
1,858 (2)	No serious limitations	No serious limitations	No serious limitations	No serious limitations	2.45¶	**Low: 1.75 Medium: 3.51 High: 7.02**	**Moderate**
**Fatal venous thromboembolism**							
1,858 (2)	No serious limitations	No serious limitations	No serious limitations	No serious limitations	0.09	**Low: 0.07 Medium: 0.13 High: 0.27**	**Low**
**Symptomatic splanchnic vein thrombosis§**							
353 (1)	Serious limitations	No serious limitations	No serious limitations	Serious limitations	0.85	**0.85**	**Very low**
**Non-fatal bleeding requiring reintervention**							
971 (2)	Serious limitations	No serious limitations	No serious limitations	Serious limitations	1.10	**1.10**	**Very low**
**Non-fatal bleeding leading to transfusion**							
2,136 (2)	No serious limitations	No serious limitations	No serious limitations	No serious limitations	4.35	**4.35**	**Moderate**
**Fatal bleeding**							
971 (2)	Serious limitations	No serious limitations	No serious limitations	Serious limitations	0.04	**0.04**	**Very low**
**Bleeding leading to hemoglobin below 70g/L (7g/dL)**							

Blank spaces indicate absence of information

* Estimate represents absolute risk in percent. Our median best estimates include fatal and non-fatal events. Based on data from included studies, we estimated case fatality rates as follows: 3.6% for VTE, 3.6% for bleeding leading to reintervention, and 0.9% for bleeding leading to transfusion, and used this information to calculate outcome estimates. For instance, we multiplied the median VTE estimate by 0.964 for non-fatal VTE and by 0.036 for fatal VTE (if both reintervention and transfusion rates were available, we preferred reintervention estimates for calculation of fatal bleeding estimate).

† Risk factors included 1) age more than 75 years, 2) obesity (body mass index of 35 or more), 3) VTE in a first degree relative (parents, full siblings, or children), and 4) prior VTE. We assumed that patients with any combination of two or more risk factors had a risk ratio of 4. Using these risk factors, we then categorized risk of VTE as low, medium, and high risk.

‡ Options for certainty in estimates are high, moderate, low, and very low. Evidence begins as high and is rated down for serious risk of bias, inconsistency, imprecision, or indirectness. We always rated down once due to uncertainty in the patient VTE risk factors and models of timing of VTE and bleeding. For fatal VTE and fatal bleeding we always rated down once for uncertainty in our case fatality rate estimates.

§ The best median estimate for symptomatic splanchnic vein thrombosis is median value of reported estimates. As we did not find evidence for timing of SVT, effect of thromboprophylaxis on SVT or patient risk factors for SVT, we did not model splanchnic vein thrombosis estimates for these factors.

¶We had only two studies reporting VTE, one of them large (1789 patients) with low risk of bias, and one of them small (69 patients) with high risk of bias and zero events. We used mean instead of median to give not too big emphasis on small study with high risk of bias.

77. Evidence profile 77. Distal pancreatectomy, robotic: Absolute risk of venous thromboembolism and bleeding among patients not receiving prophylaxis

Quality assessment					Summary of findings		
**No of participants (studies)**	**Risk of Bias**	**Inconsistency**	**Indirectness**	**Imprecision**	**Best (median) estimate across all risk strata (%)***	**Best (median estimate) by patient risk strata (%)†**	**Overall certainty in estimates‡**
**Non-fatal symptomatic venous thromboembolism**							
**Fatal venous thromboembolism**							
**Symptomatic splanchnic vein thrombosis**							
**Non-fatal bleeding requiring reintervention**							
83 (1)	No serious limitations	No serious limitations	No serious limitations	Very serious limitations	0.80	**0.80**	**Very low**
**Non-fatal bleeding leading to transfusion**							
**Fatal bleeding**							
83 (1)	No serious limitations	No serious limitations	No serious limitations	Very serious limitations	0.03	**0.03**	**Very low**
**Bleeding leading to hemoglobin below 70g/L (7g/dL)**							

Blank spaces indicate absence of information

* Estimate represents absolute risk in percent. Our median best estimates include fatal and non-fatal events. Based on data from included studies, we estimated case fatality rates as follows: 3.6% for VTE, 3.6% for bleeding leading to reintervention, and 0.9% for bleeding leading to transfusion, and used this information to calculate outcome estimates. For instance, we multiplied the median VTE estimate by 0.964 for non-fatal VTE and by 0.036 for fatal VTE (if both reintervention and transfusion rates were available, we preferred reintervention estimates for calculation of fatal bleeding estimate).

† Risk factors included 1) age more than 75 years, 2) obesity (body mass index of 35 or more), 3) VTE in a first degree relative (parents, full siblings, or children), and 4) prior VTE. We assumed that patients with any combination of two or more risk factors had a risk ratio of 4. Using these risk factors, we then categorized risk of VTE as low, medium, and high risk.

‡ Options for certainty in estimates are high, moderate, low, and very low. Evidence begins as high and is rated down for serious risk of bias, inconsistency, imprecision, or indirectness. We always rated down once due to uncertainty in the patient VTE risk factors and models of timing of VTE and bleeding. For fatal VTE and fatal bleeding we always rated down once for uncertainty in our case fatality rate estimates.

78. Evidence profile 78. Distal pancreatectomy, open: Absolute risk of venous thromboembolism and bleeding among patients not receiving prophylaxis

Quality assessment					Summary of findings		
**No of participants (studies)**	**Risk of Bias**	**Inconsistency**	**Indirectness**	**Imprecision**	**Best (median) estimate across all risk strata (%)***	**Best (median estimate) by patient risk strata (%)†**	**Overall certainty in estimates‡**
**Non-fatal symptomatic venous thromboembolism**							
2,106 (4)	No serious limitations	Serious limitations	No serious limitations	No serious limitations	6.20	**Low: 4.02 Medium: 8.03 High: 16.06**	**Low**
**Fatal venous thromboembolism**							
2,106 (4)	No serious limitations	Serious limitations	No serious limitations	No serious limitations	0.23	**Low: 0.15 Medium: 0.3 High: 0.6**	**Very Low**
**Symptomatic splanchnic vein thrombosis§**							
180 (1)	Serious limitations	No serious limitations	No serious limitations	Very serious limitations	2.22	**2.22**	**Very low**
**Non-fatal bleeding requiring reintervention**							
1,485 (4)	Serious limitations	Serious limitations	No serious limitations	No serious limitations	0.64	**0.64**	**Very low**
**Non-fatal bleeding leading to transfusion**							
4,196 (2)	Serious limitations	No serious limitations	No serious limitations	No serious limitations	9.38	**9.38**	**Low**
**Fatal bleeding**							
1,485 (4)	Serious limitations	Serious limitations	No serious limitations	No serious limitations	0.02	**0.02**	**Very low**
**Bleeding leading to hemoglobin below 70g/L (7g/dL)**							

Blank spaces indicate absence of information
* Estimate represents absolute risk in percent. Our median best estimates include fatal and non-fatal events. Based on data from included studies, we estimated case fatality rates as follows: 3.6% for VTE, 3.6% for bleeding leading to reintervention, and 0.9% for bleeding leading to transfusion, and used this information to calculate outcome estimates. For instance, we multiplied the median VTE estimate by 0.964 for non-fatal VTE and by 0.036 for fatal VTE (if both reintervention and transfusion rates were available, we preferred reintervention estimates for calculation of fatal bleeding estimate).
† Risk factors included 1) age more than 75 years, 2) obesity (body mass index of 35 or more), 3) VTE in a first degree relative (parents, full siblings, or children), and 4) prior VTE. We assumed that patients with any combination of two or more risk factors had a risk ratio of 4. Using these risk factors, we then categorized risk of VTE as low, medium, and high risk.
‡ Options for certainty in estimates are high, moderate, low, and very low. Evidence begins as high and is rated down for serious risk of bias, inconsistency, imprecision, or indirectness. We always rated down once due to uncertainty in the patient VTE risk factors and models of timing of VTE and bleeding. For fatal VTE and fatal bleeding we always rated down once for uncertainty in our case fatality rate estimates.
§ The best median estimate for symptomatic splanchnic vein thrombosis is median value of reported estimates. As we did not find evidence for timing of SVT, effect of thromboprophylaxis on SVT or patient risk factors for SVT, we did not model splanchnic vein thrombosis estimates for these factors.

79. Evidence profile 79. Distal pancreatectomy, laparoscopic, benign: Absolute risk of venous thromboembolism and bleeding among patients not receiving prophylaxis

Quality assessment					Summary of findings		
**No of participants (studies)**	**Risk of Bias**	**Inconsistency**	**Indirectness**	**Imprecision**	**Best (median) estimate across all risk strata (%)***	**Best (median estimate) by patient risk strata (%)†**	**Overall certainty in estimates‡**
**Non-fatal symptomatic venous thromboembolism**							
1,030 (1)	No serious limitations	No serious limitations	No serious limitations	No serious limitations	2.12	**Low: 1.58 Medium: 3.16 High: 6.33**	**Moderate**
**Fatal venous thromboembolism**							
1,030 (1)	No serious limitations	No serious limitations	No serious limitations	No serious limitations	0.08	**Low: 0.06 Medium: 0.12 High: 0.24**	**Low**
**Symptomatic splanchnic vein thrombosis§**							
116 (1)	Serious limitations	No serious limitations	No serious limitations	Very serious limitations	0.00	**0.00**	**Very low**
**Non-fatal bleeding requiring reintervention**							
**Non-fatal bleeding leading to transfusion**							
1,030 (1)	No serious limitations	No serious limitations	No serious limitations	No serious limitations	4.18	**4.18**	**Moderate**
**Fatal bleeding**							
1,030 (1)	No serious limitations	No serious limitations	No serious limitations	No serious limitations	0.04	**0.04**	**Low**
**Bleeding leading to hemoglobin below 70g/L (7g/dL)**							

Blank spaces indicate absence of information
* Estimate represents absolute risk in percent. Our median best estimates include fatal and non-fatal events. Based on data from included studies, we estimated case fatality rates as follows: 3.6% for VTE, 3.6% for bleeding leading to reintervention, and 0.9% for bleeding leading to transfusion, and used this information to calculate outcome estimates. For instance, we multiplied the median VTE estimate by 0.964 for non-fatal VTE and by 0.036 for fatal VTE (if both reintervention and transfusion rates were available, we preferred reintervention estimates for calculation of fatal bleeding estimate).
† Risk factors included 1) age more than 75 years, 2) obesity (body mass index of 35 or more), 3) VTE in a first degree relative (parents, full siblings, or children), and 4) prior VTE. We assumed that patients with any combination of two or more risk factors had a risk ratio of 4. Using these risk factors, we then categorized risk of VTE as low, medium, and high risk.
‡ Options for certainty in estimates are high, moderate, low, and very low. Evidence begins as high and is rated down for serious risk of bias, inconsistency, imprecision, or indirectness. We always rated down once due to uncertainty in the patient VTE risk factors and models of timing of VTE and bleeding. For fatal VTE and fatal bleeding we always rated down once for uncertainty in our case fatality rate estimates.
§ The best median estimate for symptomatic splanchnic vein thrombosis is median value of reported estimates. As we did not find evidence for timing of SVT, effect of thromboprophylaxis on SVT or patient risk factors for SVT, we did not model splanchnic vein thrombosis estimates for these factors.

80. Evidence profile 80. Distal pancreatectomy, laparoscopic, malignant: Absolute risk of venous thromboembolism and bleeding among patients not receiving prophylaxis

Quality assessment					Summary of findings		
**No of participants (studies)**	**Risk of Bias**	**Inconsistency**	**Indirectness**	**Imprecision**	**Best (median) estimate across all risk strata (%)***	**Best (median estimate) by patient risk strata (%)†**	**Overall certainty in estimates‡**
**Non-fatal symptomatic venous thromboembolism**							
759 (1)	No serious limitations	No serious limitations	No serious limitations	Serious limitations	3.27	**Low: 2.24 Medium: 4.49 High: 8.97**	**Low**
**Fatal venous thromboembolism**							
759 (1)	No serious limitations	No serious limitations	No serious limitations	Serious limitations	0.12	**Low: 0.08 Medium: 0.17 High: 0.34**	**Very Low**
**Symptomatic splanchnic vein thrombosis**							
**Non-fatal bleeding requiring reintervention**							
**Non-fatal bleeding leading to transfusion**							
1,106 (2)	No serious limitations	No serious limitations	No serious limitations	No serious limitations	4.56	**4.56**	**Moderate**
**Fatal bleeding**							
1,106 (2)	No serious limitations	No serious limitations	No serious limitations	No serious limitations	0.04	**0.04**	**Low**
**Bleeding leading to hemoglobin below 70g/L (7g/dL)**							

Blank spaces indicate absence of information

* Estimate represents absolute risk in percent. Our median best estimates include fatal and non-fatal events. Based on data from included studies, we estimated case fatality rates as follows: 3.6% for VTE, 3.6% for bleeding leading to reintervention, and 0.9% for bleeding leading to transfusion, and used this information to calculate outcome estimates. For instance, we multiplied the median VTE estimate by 0.964 for non-fatal VTE and by 0.036 for fatal VTE (if both reintervention and transfusion rates were available, we preferred reintervention estimates for calculation of fatal bleeding estimate).

† Risk factors included 1) age more than 75 years, 2) obesity (body mass index of 35 or more), 3) VTE in a first degree relative (parents, full siblings, or children), and 4) prior VTE. We assumed that patients with any combination of two or more risk factors had a risk ratio of 4. Using these risk factors, we then categorized risk of VTE as low, medium, and high risk.

‡ Options for certainty in estimates are high, moderate, low, and very low. Evidence begins as high and is rated down for serious risk of bias, inconsistency, imprecision, or indirectness. We always rated down once due to uncertainty in the patient VTE risk factors and models of timing of VTE and bleeding. For fatal VTE and fatal bleeding we always rated down once for uncertainty in our case fatality rate estimates.

81. Evidence profile 81. Distal pancreatectomy, open, benign: Absolute risk of venous thromboembolism and bleeding among patients not receiving prophylaxis

Quality assessment					Summary of findings		
**No of participants (studies)**	**Risk of Bias**	**Inconsistency**	**Indirectness**	**Imprecision**	**Best (median) estimate across all risk strata (%)***	**Best (median estimate) by patient risk strata (%)†**	**Overall certainty in estimates‡**
**Non-fatal symptomatic venous thromboembolism**							
655 (1)	No serious limitations	No serious limitations	No serious limitations	Serious limitations	2.19	**Low: 1.53 Medium: 3.05 High: 6.11**	**Low**
**Fatal venous thromboembolism**							
655 (1)	No serious limitations	No serious limitations	No serious limitations	Serious limitations	0.08	**Low: 0.06 Medium: 0.11 High: 0.23**	**Very Low**
**Symptomatic splanchnic vein thrombosis**							
**Non-fatal bleeding requiring reintervention**							
**Non-fatal bleeding leading to transfusion**							
655 (1)	No serious limitations	No serious limitations	No serious limitations	Serious limitations	7.71	**7.71**	**Low**
**Fatal bleeding**							
655 (1)	No serious limitations	No serious limitations	No serious limitations	Serious limitations	0.07	**0.07**	**Very Low**
**Bleeding leading to hemoglobin below 70g/L (7g/dL)**							

Blank spaces indicate absence of information
* Estimate represents absolute risk in percent. Our median best estimates include fatal and non-fatal events. Based on data from included studies, we estimated case fatality rates as follows: 3.6% for VTE, 3.6% for bleeding leading to reintervention, and 0.9% for bleeding leading to transfusion, and used this information to calculate outcome estimates. For instance, we multiplied the median VTE estimate by 0.964 for non-fatal VTE and by 0.036 for fatal VTE (if both reintervention and transfusion rates were available, we preferred reintervention estimates for calculation of fatal bleeding estimate).
† Risk factors included 1) age more than 75 years, 2) obesity (body mass index of 35 or more), 3) VTE in a first degree relative (parents, full siblings, or children), and 4) prior VTE. We assumed that patients with any combination of two or more risk factors had a risk ratio of 4. Using these risk factors, we then categorized risk of VTE as low, medium, and high risk.
‡ Options for certainty in estimates are high, moderate, low, and very low. Evidence begins as high and is rated down for serious risk of bias, inconsistency, imprecision, or indirectness. We always rated down once due to uncertainty in the patient VTE risk factors and models of timing of VTE and bleeding. For fatal VTE and fatal bleeding we always rated down once for uncertainty in our case fatality rate estimates.

82. Evidence profile 82. Distal pancreatectomy, open, malignant: Absolute risk of venous thromboembolism and bleeding among patients not receiving prophylaxis

Quality assessment					Summary of findings		
**No of participants (studies)**	**Risk of Bias**	**Inconsistency**	**Indirectness**	**Imprecision**	**Best (median) estimate across all risk strata (%)***	**Best (median estimate) by patient risk strata (%)†**	**Overall certainty in estimates‡**
**Non-fatal symptomatic venous thromboembolism**							
1,260 (3)	No serious limitations	Serious limitations	No serious limitations	No serious limitations	6.57	**Low: 4.32 Medium: 8.64 High: 17.29**	**Low**
**Fatal venous thromboembolism**							
1,260 (3)	No serious limitations	Serious limitations	No serious limitations	No serious limitations	0.25	**Low: 0.16 Medium: 0.32 High: 0.65**	**Very Low**
**Symptomatic splanchnic vein thrombosis**							
**Non-fatal bleeding requiring reintervention¶**							
70 (1)	No serious limitations	No serious limitations	No serious limitations	Very serious limitations	0.00	**0.00**	**Very low**
**Non-fatal bleeding leading to transfusion**							
3,541 (2)	Serious limitations	No serious limitations	No serious limitations	No serious limitations	9.50	**9.50**	**Low**
**Fatal bleeding**							
3,541 (2)	Serious limitations	No serious limitations	No serious limitations	No serious limitations	0.00	**0.00**	**Very Low**
**Bleeding leading to hemoglobin below 70g/L (7g/dL)**							

Blank spaces indicate absence of information
* Estimate represents absolute risk in percent. Our median best estimates include fatal and non-fatal events. Based on data from included studies, we estimated case fatality rates as follows: 3.6% for VTE, 3.6% for bleeding leading to reintervention, and 0.9% for bleeding leading to transfusion, and used this information to calculate outcome estimates. For instance, we multiplied the median VTE estimate by 0.964 for non-fatal VTE and by 0.036 for fatal VTE (if both reintervention and transfusion rates were available, we preferred reintervention estimates for calculation of fatal bleeding estimate).
† Risk factors included 1) age more than 75 years, 2) obesity (body mass index of 35 or more), 3) VTE in a first degree relative (parents, full siblings, or children), and 4) prior VTE. We assumed that patients with any combination of two or more risk factors had a risk ratio of 4. Using these risk factors, we then categorized risk of VTE as low, medium, and high risk.
‡ Options for certainty in estimates are high, moderate, low, and very low. Evidence begins as high and is rated down for serious risk of bias, inconsistency, imprecision, or indirectness. We always rated down once due to uncertainty in the patient VTE risk factors and models of timing of VTE and bleeding. For fatal VTE and fatal bleeding we always rated down once for uncertainty in our case fatality rate estimates.
¶We did not include this estimate in the main article (Table 3) as the evidence is very low certainty and lacks face validity.

83. Evidence profile 83. Liver resection, minimally-invasive: Absolute risk of venous thromboembolism and bleeding among patients not receiving prophylaxis

Quality assessment					Summary of findings		
**No of participants (studies)**	**Risk of Bias**	**Inconsistency**	**Indirectness**	**Imprecision**	**Best (median) estimate across all risk strata (%)***	**Best (median estimate) by patient risk strata (%)†**	**Overall certainty in estimates‡**
**Non-fatal symptomatic venous thromboembolism**							
3,270 (8)	No serious limitations	Serious limitations	No serious limitations	No serious limitations	0.79	**Low: 0.54 Medium: 1.07 High: 2.14**	**Low**
**Fatal venous thromboembolism**							
3,270 (8)	No serious limitations	Serious limitations	No serious limitations	No serious limitations	0.03	**Low: 0.02 Medium: 0.04 High: 0.08**	**Very Low**
**Symptomatic splanchnic vein thrombosis§**							
435 (2)	Serious limitations	No serious limitations	No serious limitations	Serious limitations	0.43	**0.43**	**Very low**
**Non-fatal bleeding requiring reintervention**							
617 (6)	Serious limitations	No serious limitations	No serious limitations	Serious limitations	0.80	**0.80**	**Very low**
**Non-fatal bleeding leading to transfusion**							
3,924 (11)	Serious limitations	Serious limitations	No serious limitations	No serious limitations	2.77	**2.77**	**Very low**
**Fatal bleeding**							
617 (6)	Serious limitations	No serious limitations	No serious limitations	Serious limitations	0.03	**0.03**	**Very low**
**Bleeding leading to hemoglobin below 70g/L (7g/dL)**							
84 (1)	No serious limitations	No serious limitations	No serious limitations	Very serious limitations	0.00	**0.00**	**Very low**

Blank spaces indicate absence of information
* Estimate represents absolute risk in percent. Our median best estimates include fatal and non-fatal events. Based on data from included studies, we estimated case fatality rates as follows: 3.6% for VTE, 3.6% for bleeding leading to reintervention, and 0.9% for bleeding leading to transfusion, and used this information to calculate outcome estimates. For instance, we multiplied the median VTE estimate by 0.964 for non-fatal VTE and by 0.036 for fatal VTE (if both reintervention and transfusion rates were available, we preferred reintervention estimates for calculation of fatal bleeding estimate).
† Risk factors included 1) age more than 75 years, 2) obesity (body mass index of 35 or more), 3) VTE in a first degree relative (parents, full siblings, or children), and 4) prior VTE. We assumed that patients with any combination of two or more risk factors had a risk ratio of 4. Using these risk factors, we then categorized risk of VTE as low, medium, and high risk.
‡ Options for certainty in estimates are high, moderate, low, and very low. Evidence begins as high and is rated down for serious risk of bias, inconsistency, imprecision, or indirectness. We always rated down once due to uncertainty in the patient VTE risk factors and models of timing of VTE and bleeding. For fatal VTE and fatal bleeding we always rated down once for uncertainty in our case fatality rate estimates.
§ The best median estimate for symptomatic splanchnic vein thrombosis is median value of reported estimates. As we did not find evidence for timing of SVT, effect of thromboprophylaxis on SVT or patient risk factors for SVT, we did not model splanchnic vein thrombosis estimates for these factors.

84. Evidence profile 84. Liver resection, laparoscopic: Absolute risk of venous thromboembolism and bleeding among patients not receiving prophylaxis

Quality assessment					Summary of findings		
**No of participants (studies)**	**Risk of Bias**	**Inconsistency**	**Indirectness**	**Imprecision**	**Best (median) estimate across all risk strata (%)***	**Best (median estimate) by patient risk strata (%)†**	**Overall certainty in estimates‡**
**Non-fatal symptomatic venous thromboembolism**							
3,129 (6)	No serious limitations	Serious limitations	No serious limitations	No serious limitations	0.79	**Low: 0.53 Medium: 1.05 High: 2.11**	**Low**
**Fatal venous thromboembolism**							
3,129 (6)	No serious limitations	Serious limitations	No serious limitations	No serious limitations	0.03	**Low: 0.02 Medium: 0.04 High: 0.08**	**Very Low**
**Symptomatic splanchnic vein thrombosis§**							
435 (2)	Serious limitations	No serious limitations	No serious limitations	Serious limitations	0.43	**0.43**	**Very low**
**Non-fatal bleeding requiring reintervention**							
550 (5)	Serious limitations	No serious limitations	No serious limitations	Serious limitations	0.83	**0.83**	**Very low**
**Non-fatal bleeding leading to transfusion**							
3,924 (11)	Serious limitations	Serious limitations	No serious limitations	No serious limitations	2.78	**2.78**	**Very low**
**Fatal bleeding**							
550 (5)	Serious limitations	No serious limitations	No serious limitations	Serious limitations	0.03	**0.03**	**Very low**
**Bleeding leading to hemoglobin below 70g/L (7g/dL)**							
84 (1)	No serious limitations	No serious limitations	No serious limitations	Very serious limitations	0.00	**0.00**	**Very low**

Blank spaces indicate absence of information

* Estimate represents absolute risk in percent. Our median best estimates include fatal and non-fatal events. Based on data from included studies, we estimated case fatality rates as follows: 3.6% for VTE, 3.6% for bleeding leading to reintervention, and 0.9% for bleeding leading to transfusion, and used this information to calculate outcome estimates. For instance, we multiplied the median VTE estimate by 0.964 for non-fatal VTE and by 0.036 for fatal VTE (if both reintervention and transfusion rates were available, we preferred reintervention estimates for calculation of fatal bleeding estimate).

† Risk factors included 1) age more than 75 years, 2) obesity (body mass index of 35 or more), 3) VTE in a first degree relative (parents, full siblings, or children), and 4) prior VTE. We assumed that patients with any combination of two or more risk factors had a risk ratio of 4. Using these risk factors, we then categorized risk of VTE as low, medium, and high risk.

‡ Options for certainty in estimates are high, moderate, low, and very low. Evidence begins as high and is rated down for serious risk of bias, inconsistency, imprecision, or indirectness. We always rated down once due to uncertainty in the patient VTE risk factors and models of timing of VTE and bleeding. For fatal VTE and fatal bleeding we always rated down once for uncertainty in our case fatality rate estimates.

§ The best median estimate for symptomatic splanchnic vein thrombosis is median value of reported estimates. As we did not find evidence for timing of SVT, effect of thromboprophylaxis on SVT or patient risk factors for SVT, we did not model splanchnic vein thrombosis estimates for these factors.

85. Evidence profile 85. Liver resection, robotic: Absolute risk of venous thromboembolism and bleeding among patients not receiving prophylaxis

Quality assessment					Summary of findings		
**No of participants (studies)**	**Risk of Bias**	**Inconsistency**	**Indirectness**	**Imprecision**	**Best (median) estimate across all risk strata (%)***	**Best (median estimate) by patient risk strata (%)†**	**Overall certainty in estimates‡**
**Non-fatal symptomatic venous thromboembolism**							
141 (2)	No serious limitations	No serious limitations	No serious limitations	Very serious limitations	1.06	**Low: 0.72 Medium: 1.44 High: 2.88**	**Very low**
**Fatal venous thromboembolism**							
141 (2)	No serious limitations	No serious limitations	No serious limitations	Very serious limitations	0.04	**Low: 0.03 Medium: 0.05 High: 0.11**	**Very low**
**Symptomatic splanchnic vein thrombosis**							
**Non-fatal bleeding requiring reintervention**							
67 (1)	Serious limitations	No serious limitations	No serious limitations	Very serious limitations	0.00	**0.00**	**Very low**
**Non-fatal bleeding leading to transfusion**							
**Fatal bleeding**							
**Bleeding leading to hemoglobin below 70g/L (7g/dL)**							

Blank spaces indicate absence of information.
* Estimate represents absolute risk in percent. Our median best estimates include fatal and non-fatal events. Based on data from included studies, we estimated case fatality rates as follows: 3.6% for VTE, 3.6% for bleeding leading to reintervention, and 0.9% for bleeding leading to transfusion, and used this information to calculate outcome estimates. For instance, we multiplied the median VTE estimate by 0.964 for non-fatal VTE and by 0.036 for fatal VTE (if both reintervention and transfusion rates were available, we preferred reintervention estimates for calculation of fatal bleeding estimate).
† Risk factors included 1) age more than 75 years, 2) obesity (body mass index of 35 or more), 3) VTE in a first degree relative (parents, full siblings, or children), and 4) prior VTE. We assumed that patients with any combination of two or more risk factors had a risk ratio of 4. Using these risk factors, we then categorized risk of VTE as low, medium, and high risk.
‡ Options for certainty in estimates are high, moderate, low, and very low. Evidence begins as high and is rated down for serious risk of bias, inconsistency, imprecision, or indirectness. We always rated down once due to uncertainty in the patient VTE risk factors and models of timing of VTE and bleeding. For fatal VTE and fatal bleeding we always rated down once for uncertainty in our case fatality rate estimates.

86. Evidence profile 86. Liver resection, open: Absolute risk of venous thromboembolism and bleeding among patients not receiving prophylaxis

Quality assessment					Summary of findings		
**No of participants (studies)**	**Risk of Bias**	**Inconsistency**	**Indirectness**	**Imprecision**	**Best (median) estimate across all risk strata (%)***	**Best (median estimate) by patient risk strata (%)†**	**Overall certainty in estimates‡**
**Non-fatal symptomatic venous thromboembolism**							
29,872 (15)	No serious limitations	Serious limitations	No serious limitations	No serious limitations	2.54	**Low: 1.81 Medium: 3.62 High: 7.24**	**Low**
**Fatal venous thromboembolism**							
29,872 (15)	No serious limitations	Serious limitations	No serious limitations	No serious limitations	0.09	**Low: 0.07 Medium: 0.14 High: 0.27**	**Very Low**
**Symptomatic splanchnic vein thrombosis§**							
1,456 (7)	Serious limitations	Serious limitations	No serious limitations	No serious limitations	0.95	**0.95**	**Very low**
**Non-fatal bleeding requiring reintervention**							
8,649 (9)	No serious limitations	Serious limitations	No serious limitations	No serious limitations	1.05	**1.05**	**Low**
**Non-fatal bleeding leading to transfusion**							
26,511 (10)	No serious limitations	Serious limitations	No serious limitations	No serious limitations	9.21	**9.21**	**Low**
**Fatal bleeding**							
8,649 (9)	No serious limitations	Serious limitations	No serious limitations	No serious limitations	0.04	**0.04**	**Very Low**
**Bleeding leading to hemoglobin below 70g/L (7g/dL)**							
428 (1)	No serious limitations	No serious limitations	No serious limitations	Serious limitations	0.40	**0.40**	**Low**

Blank spaces indicate absence of information
We found 5 eligible studies reporting on 20,134 patients with low risk of bias and 10 studies reporting on 9,738 patients with moderate risk of bias reporting symptomatic VTE estimate for this procedure. We therefore excluded high risk of bias studies from baseline risk analyses for VTE for this procedure.
* Estimate represents absolute risk in percent. Our median best estimates include fatal and non-fatal events. Based on data from included studies, we estimated case fatality rates as follows: 3.6% for VTE, 3.6% for bleeding leading to reintervention, and 0.9% for bleeding leading to transfusion, and used this information to calculate outcome estimates. For instance, we multiplied the median VTE estimate by 0.964 for non-fatal VTE and by 0.036 for fatal VTE (if both reintervention and transfusion rates were available, we preferred reintervention estimates for calculation of fatal bleeding estimate).
† Risk factors included 1) age more than 75 years, 2) obesity (body mass index of 35 or more), 3) VTE in a first degree relative (parents, full siblings, or children), and 4) prior VTE. We assumed that patients with any combination of two or more risk factors had a risk ratio of 4. Using these risk factors, we then categorized risk of VTE as low, medium, and high risk.
‡ Options for certainty in estimates are high, moderate, low, and very low. Evidence begins as high and is rated down for serious risk of bias, inconsistency, imprecision, or indirectness. We always rated down once due to uncertainty in the patient VTE risk factors and models of timing of VTE and bleeding. For fatal VTE and fatal bleeding we always rated down once for uncertainty in our case fatality rate estimates.
§ The best median estimate for symptomatic splanchnic vein thrombosis is median value of reported estimates. As we did not find evidence for timing of SVT, effect of thromboprophylaxis on SVT or patient risk factors for SVT, we did not model splanchnic vein thrombosis estimates for these factors.

87. Evidence profile 87. Liver resection, laparoscopic, minor: Absolute risk of venous thromboembolism and bleeding among patients not receiving prophylaxis

Quality assessment					Summary of findings		
**No of participants (studies)**	**Risk of Bias**	**Inconsistency**	**Indirectness**	**Imprecision**	**Best (median) estimate across all risk strata (%)***	**Best (median estimate) by patient risk strata (%)†**	**Overall certainty in estimates‡**
**Non-fatal symptomatic venous thromboembolism**							
937 (2)	No serious limitations	No serious limitations	No serious limitations	Serious limitations	0.76	**Low: 0.51 Medium: 1.02 High: 2.05**	**Low**
**Fatal venous thromboembolism**							
937 (2)	No serious limitations	No serious limitations	No serious limitations	Serious limitations	0.03	**Low: 0.02 Medium: 0.04 High: 0.08**	**Very Low**
**Symptomatic splanchnic vein thrombosis§**							
429 (2)	Serious limitations	No serious limitations	No serious limitations	Serious limitations	0.43	**0.43**	**Very low**
**Non-fatal bleeding requiring reintervention**							
**Non-fatal bleeding leading to transfusion**							
1,288 (3)	No serious limitations	No serious limitations	No serious limitations	No serious limitations	2.50	**2.50**	**Moderate**
**Fatal bleeding**							
1,288 (3)	No serious limitations	No serious limitations	No serious limitations	No serious limitations	0.00	**0.00**	**Low**
**Bleeding leading to hemoglobin below 70g/L (7g/dL)**							

Blank spaces indicate absence of information
We accepted the definition of minor liver resection used in each study, both included studies defined minor resection as resection of at most 2 segments.
* Estimate represents absolute risk in percent. Our median best estimates include fatal and non-fatal events. Based on data from included studies, we estimated case fatality rates as follows: 3.6% for VTE, 3.6% for bleeding leading to reintervention, and 0.9% for bleeding leading to transfusion, and used this information to calculate outcome estimates. For instance, we multiplied the median VTE estimate by 0.964 for non-fatal VTE and by 0.036 for fatal VTE (if both reintervention and transfusion rates were available, we preferred reintervention estimates for calculation of fatal bleeding estimate).
† Risk factors included 1) age more than 75 years, 2) obesity (body mass index of 35 or more), 3) VTE in a first degree relative (parents, full siblings, or children), and 4) prior VTE. We assumed that patients with any combination of two or more risk factors had a risk ratio of 4. Using these risk factors, we then categorized risk of VTE as low, medium, and high risk.
‡ Options for certainty in estimates are high, moderate, low, and very low. Evidence begins as high and is rated down for serious risk of bias, inconsistency, imprecision, or indirectness. We always rated down once due to uncertainty in the patient VTE risk factors and models of timing of VTE and bleeding. For fatal VTE and fatal bleeding we always rated down once for uncertainty in our case fatality rate estimates.
§ The best median estimate for symptomatic splanchnic vein thrombosis is median value of reported estimates. As we did not find evidence for timing of SVT, effect of thromboprophylaxis on SVT or patient risk factors for SVT, we did not model splanchnic vein thrombosis estimates for these factors.

88. Evidence profile 88. Liver resection, laparoscopic, major: Absolute risk of venous thromboembolism and bleeding among patients not receiving prophylaxis

Quality assessment					Summary of findings		
**No of participants (studies)**	**Risk of Bias**	**Inconsistency**	**Indirectness**	**Imprecision**	**Best (median) estimate across all risk strata (%)***	**Best (median estimate) by patient risk strata (%)†**	**Overall certainty in estimates‡**
**Non-fatal symptomatic venous thromboembolism**							
226 (1)	Serious limitations	No serious limitations	No serious limitations	Serious limitations	4.26	**Low: 2.84 Medium: 5.69 High: 11.38**	**Very low**
**Fatal venous thromboembolism**							
226 (1)	Serious limitations	No serious limitations	No serious limitations	Serious limitations	0.16	**Low: 0.11 Medium: 0.21 High: 0.42**	**Very low**
**Symptomatic splanchnic vein thrombosis**							
**Non-fatal bleeding requiring reintervention**							
**Non-fatal bleeding leading to transfusion**							
449 (2)	Serious limitations	No serious limitations	No serious limitations	Serious limitations	7.60	**7.60**	**Very low**
**Fatal bleeding**							
449 (2)	Serious limitations	No serious limitations	No serious limitations	Serious limitations	0.07	**0.07**	**Very low**
**Bleeding leading to hemoglobin below 70g/L (7g/dL)**							

Blank spaces indicate absence of information
We accepted the definition of major liver resection used in each study, all three studies defined major resection as resection of 3 or more segments
* Estimate represents absolute risk in percent. Our median best estimates include fatal and non-fatal events. Based on data from included studies, we estimated case fatality rates as follows: 3.6% for VTE, 3.6% for bleeding leading to reintervention, and 0.9% for bleeding leading to transfusion, and used this information to calculate outcome estimates. For instance, we multiplied the median VTE estimate by 0.964 for non-fatal VTE and by 0.036 for fatal VTE (if both reintervention and transfusion rates were available, we preferred reintervention estimates for calculation of fatal bleeding estimate).
† Risk factors included 1) age more than 75 years, 2) obesity (body mass index of 35 or more), 3) VTE in a first degree relative (parents, full siblings, or children), and 4) prior VTE. We assumed that patients with any combination of two or more risk factors had a risk ratio of 4. Using these risk factors, we then categorized risk of VTE as low, medium, and high risk.
‡ Options for certainty in estimates are high, moderate, low, and very low. Evidence begins as high and is rated down for serious risk of bias, inconsistency, imprecision, or indirectness. We always rated down once due to uncertainty in the patient VTE risk factors and models of timing of VTE and bleeding. For fatal VTE and fatal bleeding we always rated down once for uncertainty in our case fatality rate estimates.

89. Evidence profile 89. Liver resection, open, minor: Absolute risk of venous thromboembolism and bleeding among patients not receiving prophylaxis

Quality assessment					Summary of findings		
**No of participants (studies)**	**Risk of Bias**	**Inconsistency**	**Indirectness**	**Imprecision**	**Best (median) estimate across all risk strata (%)***	**Best (median estimate) by patient risk strata (%)†**	**Overall certainty in estimates‡**
**Non-fatal symptomatic venous thromboembolism**							
4,165 (3)	No serious limitations	Serious limitations	No serious limitations	No serious limitations	3.41	**Low: 2.32 Medium: 4.64 High: 9.28**	**Low**
**Fatal venous thromboembolism**							
4,165 (3)	No serious limitations	Serious limitations	No serious limitations	No serious limitations	0.13	**Low: 0.09 Medium: 0.17 High: 0.35**	**Very Low**
**Symptomatic splanchnic vein thrombosis**							
**Non-fatal bleeding requiring reintervention**							
4,165 (3)	No serious limitations	No serious limitations	No serious limitations	No serious limitations	0.51	**0.51**	**Moderate**
**Non-fatal bleeding leading to transfusion**							
4,165 (3)	No serious limitations	Serious limitations	No serious limitations	No serious limitations	4.58	**4.58**	**Low**
**Fatal bleeding**							
4,165 (3)	No serious limitations	No serious limitations	No serious limitations	No serious limitations	0.02	**0.02**	**Low**
**Bleeding leading to hemoglobin below 70g/L (7g/dL)**							

Blank spaces indicate absence of information

We accepted the definition of minor liver resection used in each study, all included studies defined minor resection as resection of at most 2 segments.

* Estimate represents absolute risk in percent. Our median best estimates include fatal and non-fatal events. Based on data from included studies, we estimated case fatality rates as follows: 3.6% for VTE, 3.6% for bleeding leading to reintervention, and 0.9% for bleeding leading to transfusion, and used this information to calculate outcome estimates. For instance, we multiplied the median VTE estimate by 0.964 for non-fatal VTE and by 0.036 for fatal VTE (if both reintervention and transfusion rates were available, we preferred reintervention estimates for calculation of fatal bleeding estimate).

† Risk factors included 1) age more than 75 years, 2) obesity (body mass index of 35 or more), 3) VTE in a first degree relative (parents, full siblings, or children), and 4) prior VTE. We assumed that patients with any combination of two or more risk factors had a risk ratio of 4. Using these risk factors, we then categorized risk of VTE as low, medium, and high risk.

‡ Options for certainty in estimates are high, moderate, low, and very low. Evidence begins as high and is rated down for serious risk of bias, inconsistency, imprecision, or indirectness. We always rated down once due to uncertainty in the patient VTE risk factors and models of timing of VTE and bleeding. For fatal VTE and fatal bleeding we always rated down once for uncertainty in our case fatality rate estimates.

90. Evidence profile 90. Liver resection, open, major: Absolute risk of venous thromboembolism and bleeding among patients not receiving prophylaxis

Quality assessment					Summary of findings		
**No of participants (studies)**	**Risk of Bias**	**Inconsistency**	**Indirectness**	**Imprecision**	**Best (median) estimate across all risk strata (%)***	**Best (median estimate) by patient risk strata (%)†**	**Overall certainty in estimates‡**
**Non-fatal symptomatic venous thromboembolism**							
3,943 (8)	No serious limitations	Serious limitations	No serious limitations	No serious limitations	5.10	**Low: 3.74 Medium: 7.49 High: 14.97**	**Low**
**Fatal venous thromboembolism**							
3,943 (8)	No serious limitations	Serious limitations	No serious limitations	No serious limitations	0.19	**Low: 0.14 Medium: 0.28 High: 0.56**	**Very Low**
**Symptomatic splanchnic vein thrombosis§**							
885 (5)	Serious limitations	Serious limitations	No serious limitations	Serious limitations	0.95	**0.95**	**Very low**
**Non-fatal bleeding requiring reintervention**							
2,233 (5)	No serious limitations	Serious limitations	No serious limitations	No serious limitations	0.90	**0.90**	**Low**
**Non-fatal bleeding leading to transfusion**							
3,067 (4)	No serious limitations	Serious limitations	No serious limitations	No serious limitations	12.75	**12.75**	**Low**
**Fatal bleeding**							
2,233 (5)	No serious limitations	Serious limitations	No serious limitations	No serious limitations	0.03	**0.03**	**Very Low**
**Bleeding leading to hemoglobin below 70g/L (7g/dL)**							

Blank spaces indicate absence of information
We accepted the definition of major liver resection used in each study. 5 studies included resection of 3 or more segments and 3 studies resections of 4 or more segments.
* Estimate represents absolute risk in percent. Our median best estimates include fatal and non-fatal events. Based on data from included studies, we estimated case fatality rates as follows: 3.6% for VTE, 3.6% for bleeding leading to reintervention, and 0.9% for bleeding leading to transfusion, and used this information to calculate outcome estimates. For instance, we multiplied the median VTE estimate by 0.964 for non-fatal VTE and by 0.036 for fatal VTE (if both reintervention and transfusion rates were available, we preferred reintervention estimates for calculation of fatal bleeding estimate).
† Risk factors included 1) age more than 75 years, 2) obesity (body mass index of 35 or more), 3) VTE in a first degree relative (parents, full siblings, or children), and 4) prior VTE. We assumed that patients with any combination of two or more risk factors had a risk ratio of 4. Using these risk factors, we then categorized risk of VTE as low, medium, and high risk.
‡ Options for certainty in estimates are high, moderate, low, and very low. Evidence begins as high and is rated down for serious risk of bias, inconsistency, imprecision, or indirectness. We always rated down once due to uncertainty in the patient VTE risk factors and models of timing of VTE and bleeding. For fatal VTE and fatal bleeding we always rated down once for uncertainty in our case fatality rate estimates.
§ The best median estimate for symptomatic splanchnic vein thrombosis is median value of reported estimates. As we did not find evidence for timing of SVT, effect of thromboprophylaxis on SVT or patient risk factors for SVT, we did not model splanchnic vein thrombosis estimates for these factors.

91. Evidence profile 91. Pancreaticoduodenectomy, minimally-invasive: Absolute risk of venous thromboembolism and bleeding among patients not receiving prophylaxis

Quality assessment					Summary of findings		
**No of participants (studies)**	**Risk of Bias**	**Inconsistency**	**Indirectness**	**Imprecision**	**Best (median) estimate across all risk strata (%)***	**Best (median estimate) by patient risk strata (%)†**	**Overall certainty in estimates‡**
**Non-fatal symptomatic venous thromboembolism**							
1,872 (5)	No serious limitations	Serious limitations	No serious limitations	No serious limitations	5.15	**Low: 3.57 Medium: 7.14 High: 14.29**	**Low**
**Fatal venous thromboembolism**							
1,872 (5)	No serious limitations	Serious limitations	No serious limitations	No serious limitations	0.19	**Low: 0.13 Medium: 0.27 High: 0.53**	**Very Low**
**Symptomatic splanchnic vein thrombosis§**							
886 (3)	Serious limitations	Serious limitations	No serious limitations	Serious limitations	1.61	**1.61**	**Very low**
**Non-fatal bleeding requiring reintervention**							
896 (5)	No serious limitations	Serious limitations	No serious limitations	Serious limitations	1.72	**1.72**	**Very low**
**Non-fatal bleeding leading to transfusion**							
2,110 (4)	No serious limitations	Serious limitations	No serious limitations	No serious limitations	2.34	**2.34**	**Low**
**Fatal bleeding**							
896 (5)	No serious limitations	Serious limitations	No serious limitations	Serious limitations	0.06	**0.06**	**Very low**
**Bleeding leading to hemoglobin below 70g/L (7g/dL)**							

Blank spaces indicate absence of information

* Estimate represents absolute risk in percent. Our median best estimates include fatal and non-fatal events. Based on data from included studies, we estimated case fatality rates as follows: 3.6% for VTE, 3.6% for bleeding leading to reintervention, and 0.9% for bleeding leading to transfusion, and used this information to calculate outcome estimates. For instance, we multiplied the median VTE estimate by 0.964 for non-fatal VTE and by 0.036 for fatal VTE (if both reintervention and transfusion rates were available, we preferred reintervention estimates for calculation of fatal bleeding estimate).

† Risk factors included 1) age more than 75 years, 2) obesity (body mass index of 35 or more), 3) VTE in a first degree relative (parents, full siblings, or children), and 4) prior VTE. We assumed that patients with any combination of two or more risk factors had a risk ratio of 4. Using these risk factors, we then categorized risk of VTE as low, medium, and high risk.

‡ Options for certainty in estimates are high, moderate, low, and very low. Evidence begins as high and is rated down for serious risk of bias, inconsistency, imprecision, or indirectness. We always rated down once due to uncertainty in the patient VTE risk factors and models of timing of VTE and bleeding. For fatal VTE and fatal bleeding we always rated down once for uncertainty in our case fatality rate estimates.

§ The best median estimate for symptomatic splanchnic vein thrombosis is median value of reported estimates. As we did not find evidence for timing of SVT, effect of thromboprophylaxis on SVT or patient risk factors for SVT, we did not model splanchnic vein thrombosis estimates for these factors.

92. Evidence profile 92. Pancreaticoduodenectomy, laparoscopic: Absolute risk of venous thromboembolism and bleeding among patients not receiving prophylaxis

Quality assessment					Summary of findings		
**No of participants (studies)**	**Risk of Bias**	**Inconsistency**	**Indirectness**	**Imprecision**	**Best (median) estimate across all risk strata (%)***	**Best (median estimate) by patient risk strata (%)†**	**Overall certainty in estimates‡**
**Non-fatal symptomatic venous thromboembolism**							
1,219 (4)	No serious limitations	Serious limitations	No serious limitations	No serious limitations	6.31	**Low: 4.54 Medium: 9.07 High: 18.14**	**Low**
**Fatal venous thromboembolism**							
1,219 (4)	No serious limitations	Serious limitations	No serious limitations	No serious limitations	0.24	**Low: 0.17 Medium: 0.34 High: 0.68**	**Very Low**
**Symptomatic splanchnic vein thrombosis§**							
886 (3)	Serious limitations	Serious limitations	No serious limitations	Serious limitations	1.61	**1.61**	**Very low**
**Non-fatal bleeding requiring reintervention**							
632 (3)	No serious limitations	Serious limitations	No serious limitations	Serious limitations	1.72	**1.72**	**Very low**
**Non-fatal bleeding leading to transfusion**							
1,457 (3)	No serious limitations	Serious limitations	No serious limitations	No serious limitations	3.14	**3.14**	**Low**
**Fatal bleeding**							
632 (3)	No serious limitations	Serious limitations	No serious limitations	Serious limitations	0.06	**0.06**	**Very low**
**Bleeding leading to hemoglobin below 70g/L (7g/dL)**							

Blank spaces indicate absence of information

* Estimate represents absolute risk in percent. Our median best estimates include fatal and non-fatal events. Based on data from included studies, we estimated case fatality rates as follows: 3.6% for VTE, 3.6% for bleeding leading to reintervention, and 0.9% for bleeding leading to transfusion, and used this information to calculate outcome estimates. For instance, we multiplied the median VTE estimate by 0.964 for non-fatal VTE and by 0.036 for fatal VTE (if both reintervention and transfusion rates were available, we preferred reintervention estimates for calculation of fatal bleeding estimate).

† Risk factors included 1) age more than 75 years, 2) obesity (body mass index of 35 or more), 3) VTE in a first degree relative (parents, full siblings, or children), and 4) prior VTE. We assumed that patients with any combination of two or more risk factors had a risk ratio of 4. Using these risk factors, we then categorized risk of VTE as low, medium, and high risk.

‡ Options for certainty in estimates are high, moderate, low, and very low. Evidence begins as high and is rated down for serious risk of bias, inconsistency, imprecision, or indirectness. We always rated down once due to uncertainty in the patient VTE risk factors and models of timing of VTE and bleeding. For fatal VTE and fatal bleeding we always rated down once for uncertainty in our case fatality rate estimates.

§ The best median estimate for symptomatic splanchnic vein thrombosis is median value of reported estimates. As we did not find evidence for timing of SVT, effect of thromboprophylaxis on SVT or patient risk factors for SVT, we did not model splanchnic vein thrombosis estimates for these factors.

93. Evidence profile 93. Pancreaticoduodenectomy, robotic: Absolute risk of venous thromboembolism and bleeding among patients not receiving prophylaxis

Quality assessment					Summary of findings		
**No of participants (studies)**	**Risk of Bias**	**Inconsistency**	**Indirectness**	**Imprecision**	**Best (median) estimate across all risk strata (%)***	**Best (median estimate) by patient risk strata (%)†**	**Overall certainty in estimates‡**
**Non-fatal symptomatic venous thromboembolism**							
653 (2)	No serious limitations	Serious limitations	No serious limitations	Serious limitations	3.53	**Low: 2.26 Medium: 4.52 High: 9.04**	**Very low**
**Fatal venous thromboembolism**							
653 (2)	No serious limitations	Serious limitations	No serious limitations	Serious limitations	0.13	**Low: 0.08 Medium: 0.17 High: 0.34**	**Very low**
**Symptomatic splanchnic vein thrombosis**							
**Non-fatal bleeding requiring reintervention**							
132 (1)	No serious limitations	No serious limitations	No serious limitations	Very serious limitations	2.00	**2.00**	**Very low**
**Non-fatal bleeding leading to transfusion**							
653 (2)	No serious limitations	No serious limitations	No serious limitations	Serious limitations	3.17	**3.17**	**Low**
**Fatal bleeding**							
132 (1)	No serious limitations	No serious limitations	No serious limitations	Very serious limitations	0.07	**0.07**	**Very low**
**Bleeding leading to hemoglobin below 70g/L (7g/dL)**							

Blank spaces indicate absence of information.

* Estimate represents absolute risk in percent. Our median best estimates include fatal and non-fatal events. Based on data from included studies, we estimated case fatality rates as follows: 3.6% for VTE, 3.6% for bleeding leading to reintervention, and 0.9% for bleeding leading to transfusion, and used this information to calculate outcome estimates. For instance, we multiplied the median VTE estimate by 0.964 for non-fatal VTE and by 0.036 for fatal VTE (if both reintervention and transfusion rates were available, we preferred reintervention estimates for calculation of fatal bleeding estimate).

† Risk factors included 1) age more than 75 years, 2) obesity (body mass index of 35 or more), 3) VTE in a first degree relative (parents, full siblings, or children), and 4) prior VTE. We assumed that patients with any combination of two or more risk factors had a risk ratio of 4. Using these risk factors, we then categorized risk of VTE as low, medium, and high risk.

‡ Options for certainty in estimates are high, moderate, low, and very low. Evidence begins as high and is rated down for serious risk of bias, inconsistency, imprecision, or indirectness. We always rated down once due to uncertainty in the patient VTE risk factors and models of timing of VTE and bleeding. For fatal VTE and fatal bleeding we always rated down once for uncertainty in our case fatality rate estimates.

94. Evidence profile 94. Pancreaticoduodenectomy, open: Absolute risk of venous thromboembolism and bleeding among patients not receiving prophylaxis

Quality assessment					Summary of findings		
**No of participants (studies)**	**Risk of Bias**	**Inconsistency**	**Indirectness**	**Imprecision**	**Best (median) estimate across all risk strata (%)***	**Best (median estimate) by patient risk strata (%)†**	**Overall certainty in estimates‡**
**Non-fatal symptomatic venous thromboembolism**							
34,004 (12)	No serious limitations	Serious limitations	No serious limitations	No serious limitations	5.99	**Low: 4 Medium: 7.99 High: 15.99**	**Low**
**Fatal venous thromboembolism**							
34,004 (12)	No serious limitations	Serious limitations	No serious limitations	No serious limitations	0.22	**Low: 0.15 Medium: 0.3 High: 0.6**	**Very Low**
**Symptomatic splanchnic vein thrombosis§**							
298 (3)	Serious limitations	Serious limitations	No serious limitations	Serious limitations	1.49	**1.49**	**Very low**
**Non-fatal bleeding requiring reintervention**							
2,472 (10)	No serious limitations	Serious limitations	No serious limitations	No serious limitations	2.61	**2.61**	**Low**
**Non-fatal bleeding leading to transfusion**							
36,207 (12)	No serious limitations	Serious limitations	No serious limitations	No serious limitations	8.24	**8.24**	**Low**
**Fatal bleeding**							
2,472 (10)	No serious limitations	Serious limitations	No serious limitations	No serious limitations	0.10	**0.10**	**Very Low**
**Bleeding leading to hemoglobin below 70g/L (7g/dL)**							

Blank spaces indicate absence of information

* Estimate represents absolute risk in percent. Our median best estimates include fatal and non-fatal events. Based on data from included studies, we estimated case fatality rates as follows: 3.6% for VTE, 3.6% for bleeding leading to reintervention, and 0.9% for bleeding leading to transfusion, and used this information to calculate outcome estimates. For instance, we multiplied the median VTE estimate by 0.964 for non-fatal VTE and by 0.036 for fatal VTE (if both reintervention and transfusion rates were available, we preferred reintervention estimates for calculation of fatal bleeding estimate).

† Risk factors included 1) age more than 75 years, 2) obesity (body mass index of 35 or more), 3) VTE in a first degree relative (parents, full siblings, or children), and 4) prior VTE. We assumed that patients with any combination of two or more risk factors had a risk ratio of 4. Using these risk factors, we then categorized risk of VTE as low, medium, and high risk.

‡ Options for certainty in estimates are high, moderate, low, and very low. Evidence begins as high and is rated down for serious risk of bias, inconsistency, imprecision, or indirectness. We always rated down once due to uncertainty in the patient VTE risk factors and models of timing of VTE and bleeding. For fatal VTE and fatal bleeding we always rated down once for uncertainty in our case fatality rate estimates.

§ The best median estimate for symptomatic splanchnic vein thrombosis is median value of reported estimates. As we did not find evidence for timing of SVT, effect of thromboprophylaxis on SVT or patient risk factors for SVT, we did not model splanchnic vein thrombosis estimates for these factors.

95. Evidence profile 95. Pancreaticoduodenectomy without vascular resection, laparoscopic: Absolute risk of venous thromboembolism and bleeding among patients not receiving prophylaxis

Quality assessment					Summary of findings		
**No of participants (studies)**	**Risk of Bias**	**Inconsistency**	**Indirectness**	**Imprecision**	**Best (median) estimate across all risk strata (%)***	**Best (median estimate) by patient risk strata (%)†**	**Overall certainty in estimates‡**
**Non-fatal symptomatic venous thromboembolism**							
535 (2)	No serious limitations	No serious limitations	No serious limitations	Serious limitations	3.47	**Low: 2.52 Medium: 5.04 High: 10.07**	**Low**
**Fatal venous thromboembolism**							
535 (2)	No serious limitations	No serious limitations	No serious limitations	Serious limitations	0.13	**Low: 0.09 Medium: 0.19 High: 0.38**	**Very Low**
**Symptomatic splanchnic vein thrombosis**							
**Non-fatal bleeding requiring reintervention**							
132 (2)	No serious limitations	Serious limitations	No serious limitations	Very serious limitations	6.81	**6.81**	**Very low**
**Non-fatal bleeding leading to transfusion**							
473 (1)	No serious limitations	No serious limitations	No serious limitations	Serious limitations	2.53	**2.53**	**Low**
**Fatal bleeding**							
132 (2)	No serious limitations	Serious limitations	No serious limitations	Very serious limitations	0.25	**0.25**	**Very low**
**Bleeding leading to hemoglobin below 70g/L (7g/dL)**							

Blank spaces indicate absence of information

* Estimate represents absolute risk in percent. Our median best estimates include fatal and non-fatal events. Based on data from included studies, we estimated case fatality rates as follows: 3.6% for VTE, 3.6% for bleeding leading to reintervention, and 0.9% for bleeding leading to transfusion, and used this information to calculate outcome estimates. For instance, we multiplied the median VTE estimate by 0.964 for non-fatal VTE and by 0.036 for fatal VTE (if both reintervention and transfusion rates were available, we preferred reintervention estimates for calculation of fatal bleeding estimate).

† Risk factors included 1) age more than 75 years, 2) obesity (body mass index of 35 or more), 3) VTE in a first degree relative (parents, full siblings, or children), and 4) prior VTE. We assumed that patients with any combination of two or more risk factors had a risk ratio of 4. Using these risk factors, we then categorized risk of VTE as low, medium, and high risk.

‡ Options for certainty in estimates are high, moderate, low, and very low. Evidence begins as high and is rated down for serious risk of bias, inconsistency, imprecision, or indirectness. We always rated down once due to uncertainty in the patient VTE risk factors and models of timing of VTE and bleeding. For fatal VTE and fatal bleeding we always rated down once for uncertainty in our case fatality rate estimates.

96. Evidence profile 96. Pancreaticoduodenectomy with vascular resection, laparoscopic: Absolute risk of venous thromboembolism and bleeding among patients not receiving prophylaxis

Quality assessment					Summary of findings		
**No of participants (studies)**	**Risk of Bias**	**Inconsistency**	**Indirectness**	**Imprecision**	**Best (median) estimate across all risk strata (%)***	**Best (median estimate) by patient risk strata (%)†**	**Overall certainty in estimates‡**
**Non-fatal symptomatic venous thromboembolism**							
77 (1)	No serious limitations	No serious limitations	No serious limitations	Very serious limitations	4.23	**Low: 3.35 Medium: 6.7 High: 13.4**	**Very low**
**Fatal venous thromboembolism**							
77 (1)	No serious limitations	No serious limitations	No serious limitations	Very serious limitations	0.16	**Low: 0.13 Medium: 0.25 High: 0.5**	**Very low**
**Symptomatic splanchnic vein thrombosis**							
**Non-fatal bleeding requiring reintervention**							
**Non-fatal bleeding leading to transfusion**							
77 (1)	No serious limitations	No serious limitations	No serious limitations	Very serious limitations	6.85	**6.85**	**Very low**
**Fatal bleeding**							
77 (1)	No serious limitations	No serious limitations	No serious limitations	Very serious limitations	0.06	**0.06**	**Very low**
**Bleeding leading to hemoglobin below 70g/L (7g/dL)**							

Blank spaces indicate absence of information
* Estimate represents absolute risk in percent. Our median best estimates include fatal and non-fatal events. Based on data from included studies, we estimated case fatality rates as follows: 3.6% for VTE, 3.6% for bleeding leading to reintervention, and 0.9% for bleeding leading to transfusion, and used this information to calculate outcome estimates. For instance, we multiplied the median VTE estimate by 0.964 for non-fatal VTE and by 0.036 for fatal VTE (if both reintervention and transfusion rates were available, we preferred reintervention estimates for calculation of fatal bleeding estimate).
† Risk factors included 1) age more than 75 years, 2) obesity (body mass index of 35 or more), 3) VTE in a first degree relative (parents, full siblings, or children), and 4) prior VTE. We assumed that patients with any combination of two or more risk factors had a risk ratio of 4. Using these risk factors, we then categorized risk of VTE as low, medium, and high risk.
‡ Options for certainty in estimates are high, moderate, low, and very low. Evidence begins as high and is rated down for serious risk of bias, inconsistency, imprecision, or indirectness. We always rated down once due to uncertainty in the patient VTE risk factors and models of timing of VTE and bleeding. For fatal VTE and fatal bleeding we always rated down once for uncertainty in our case fatality rate estimates.

97. Evidence profile 97. Pancreaticoduodenectomy without vascular resection, open: Absolute risk of venous thromboembolism and bleeding among patients not receiving prophylaxis

Quality assessment					Summary of findings		
**No of participants (studies)**	**Risk of Bias**	**Inconsistency**	**Indirectness**	**Imprecision**	**Best (median) estimate across all risk strata (%)***	**Best (median estimate) by patient risk strata (%)†**	**Overall certainty in estimates‡**
**Non-fatal symptomatic venous thromboembolism**							
3,017 (3)	No serious limitations	No serious limitations	No serious limitations	No serious limitations	3.15	**Low: 2.15 Medium: 4.29 High: 8.58**	**Moderate**
**Fatal venous thromboembolism**							
3,017 (3)	No serious limitations	No serious limitations	No serious limitations	No serious limitations	0.12	**Low: 0.08 Medium: 0.16 High: 0.32**	**Low**
**Symptomatic splanchnic vein thrombosis**							
111 (1)	Serious limitations	No serious limitations	No serious limitations	Very serious limitations	0.00	**0.00**	**Very low**
**Non-fatal bleeding requiring reintervention**							
1,551 (3)	Serious limitations	Serious limitations	No serious limitations	No serious limitations	4.33	**4.33**	**Very low**
**Non-fatal bleeding leading to transfusion**							
1,551 (3)	Serious limitations	Serious limitations	No serious limitations	No serious limitations	5.91	**5.91**	**Very low**
**Fatal bleeding**							
1,551 (3)	Serious limitations	Serious limitations	No serious limitations	No serious limitations	0.16	**0.16**	**Very low**
**Bleeding leading to hemoglobin below 70g/L (7g/dL)**							

Blank spaces indicate absence of information

* Estimate represents absolute risk in percent. Our median best estimates include fatal and non-fatal events. Based on data from included studies, we estimated case fatality rates as follows: 3.6% for VTE, 3.6% for bleeding leading to reintervention, and 0.9% for bleeding leading to transfusion, and used this information to calculate outcome estimates. For instance, we multiplied the median VTE estimate by 0.964 for non-fatal VTE and by 0.036 for fatal VTE (if both reintervention and transfusion rates were available, we preferred reintervention estimates for calculation of fatal bleeding estimate).

† Risk factors included 1) age more than 75 years, 2) obesity (body mass index of 35 or more), 3) VTE in a first degree relative (parents, full siblings, or children), and 4) prior VTE. We assumed that patients with any combination of two or more risk factors had a risk ratio of 4. Using these risk factors, we then categorized risk of VTE as low, medium, and high risk.

‡ Options for certainty in estimates are high, moderate, low, and very low. Evidence begins as high and is rated down for serious risk of bias, inconsistency, imprecision, or indirectness. We always rated down once due to uncertainty in the patient VTE risk factors and models of timing of VTE and bleeding. For fatal VTE and fatal bleeding we always rated down once for uncertainty in our case fatality rate estimates.

98. Evidence profile 98. Pancreaticoduodenectomy with vascular resection, open: Absolute risk of venous thromboembolism and bleeding among patients not receiving prophylaxis

Quality assessment					Summary of findings		
**No of participants (studies)**	**Risk of Bias**	**Inconsistency**	**Indirectness**	**Imprecision**	**Best (median) estimate across all risk strata (%)***	**Best (median estimate) by patient risk strata (%)†**	**Overall certainty in estimates‡**
**Non-fatal symptomatic venous thromboembolism**							
1,076 (2)	No serious limitations	No serious limitations	No serious limitations	No serious limitations	9.57	**Low: 6.58 Medium: 13.17 High: 26.34**	**Moderate**
**Fatal venous thromboembolism**							
1,076 (2)	No serious limitations	No serious limitations	No serious limitations	No serious limitations	0.36	**Low: 0.25 Medium: 0.49 High: 0.98**	**Low**
**Symptomatic splanchnic vein thrombosis§**							
350 (2)	Serious limitations	No serious limitations	No serious limitations	Serious limitations	5.27	**5.27**	**Very low**
**Non-fatal bleeding requiring reintervention**							
120 (1)	No serious limitations	No serious limitations	No serious limitations	Very serious limitations	0.47	**0.47**	**Very low**
**Non-fatal bleeding leading to transfusion**							
990 (3)	No serious limitations	Serious limitations	No serious limitations	Serious limitations	15.98	**15.98**	**Very low**
**Fatal bleeding**							
120 (1)	No serious limitations	No serious limitations	No serious limitations	Very serious limitations	0.02	**0.02**	**Very low**
**Bleeding leading to hemoglobin below 70g/L (7g/dL)**							

Blank spaces indicate absence of information

* Estimate represents absolute risk in percent. Our median best estimates include fatal and non-fatal events. Based on data from included studies, we estimated case fatality rates as follows: 3.6% for VTE, 3.6% for bleeding leading to reintervention, and 0.9% for bleeding leading to transfusion, and used this information to calculate outcome estimates. For instance, we multiplied the median VTE estimate by 0.964 for non-fatal VTE and by 0.036 for fatal VTE (if both reintervention and transfusion rates were available, we preferred reintervention estimates for calculation of fatal bleeding estimate).

† Risk factors included 1) age more than 75 years, 2) obesity (body mass index of 35 or more), 3) VTE in a first degree relative (parents, full siblings, or children), and 4) prior VTE. We assumed that patients with any combination of two or more risk factors had a risk ratio of 4. Using these risk factors, we then categorized risk of VTE as low, medium, and high risk.

‡ Options for certainty in estimates are high, moderate, low, and very low. Evidence begins as high and is rated down for serious risk of bias, inconsistency, imprecision, or indirectness. We always rated down once due to uncertainty in the patient VTE risk factors and models of timing of VTE and bleeding. For fatal VTE and fatal bleeding we always rated down once for uncertainty in our case fatality rate estimates.

§ The best median estimate for symptomatic splanchnic vein thrombosis is median value of reported estimates. As we did not find evidence for timing of SVT, effect of thromboprophylaxis on SVT or patient risk factors for SVT, we did not model splanchnic vein thrombosis estimates for these factors.

99. Evidence profile 99. Gastrectomy, minimally-invasive: Absolute risk of venous thromboembolism and bleeding among patients not receiving prophylaxis

Quality assessment					Summary of findings		
**No of participants (studies)**	**Risk of Bias**	**Inconsistency**	**Indirectness**	**Imprecision**	**Best (median) estimate across all risk strata (%)***	**Best (median estimate) by patient risk strata (%)†**	**Overall certainty in estimates‡**
**Non-fatal symptomatic venous thromboembolism**							
22,636 (18)	Serious limitations	No serious limitations	No serious limitations	No serious limitations	0.73	**Low: 0.53 Medium: 1.06 High: 2.12**	**Low**
**Fatal venous thromboembolism**							
22,636 (18)	Serious limitations	No serious limitations	No serious limitations	No serious limitations	0.03	**Low: 0.02 Medium: 0.04 High: 0.08**	**Very Low**
**Symptomatic splanchnic vein thrombosis§**							
1,470 (2)	Serious limitations	No serious limitations	No serious limitations	No serious limitations	0.15	**0.15**	**Low**
**Non-fatal bleeding requiring reintervention**							
2,562 (7)	No serious limitations	No serious limitations	No serious limitations	No serious limitations	0.33	**0.33**	**Moderate**
**Non-fatal bleeding leading to transfusion**							
13,345 (3)	Serious limitations	Serious limitations	No serious limitations	No serious limitations	2.54	**2.54**	**Very low**
**Fatal bleeding**							
2,562 (7)	No serious limitations	No serious limitations	No serious limitations	No serious limitations	0.01	**0.01**	**Low**
**Bleeding leading to hemoglobin below 70g/L (7g/dL)**							

Blank spaces indicate absence of information
* Estimate represents absolute risk in percent. Our median best estimates include fatal and non-fatal events. Based on data from included studies, we estimated case fatality rates as follows: 3.6% for VTE, 3.6% for bleeding leading to reintervention, and 0.9% for bleeding leading to transfusion, and used this information to calculate outcome estimates. For instance, we multiplied the median VTE estimate by 0.964 for non-fatal VTE and by 0.036 for fatal VTE (if both reintervention and transfusion rates were available, we preferred reintervention estimates for calculation of fatal bleeding estimate).
† Risk factors included 1) age more than 75 years, 2) obesity (body mass index of 35 or more), 3) VTE in a first degree relative (parents, full siblings, or children), and 4) prior VTE. We assumed that patients with any combination of two or more risk factors had a risk ratio of 4. Using these risk factors, we then categorized risk of VTE as low, medium, and high risk.
‡ Options for certainty in estimates are high, moderate, low, and very low. Evidence begins as high and is rated down for serious risk of bias, inconsistency, imprecision, or indirectness. We always rated down once due to uncertainty in the patient VTE risk factors and models of timing of VTE and bleeding. For fatal VTE and fatal bleeding we always rated down once for uncertainty in our case fatality rate estimates.
§ The best median estimate for symptomatic splanchnic vein thrombosis is median value of reported estimates. As we did not find evidence for timing of SVT, effect of thromboprophylaxis on SVT or patient risk factors for SVT, we did not model splanchnic vein thrombosis estimates for these factors.

100. Evidence profile 100. Gastrectomy, laparoscopic: Absolute risk of venous thromboembolism and bleeding among patients not receiving prophylaxis

Quality assessment					Summary of findings		
**No of participants (studies)**	**Risk of Bias**	**Inconsistency**	**Indirectness**	**Imprecision**	**Best (median) estimate across all risk strata (%)***	**Best (median estimate) by patient risk strata (%)†**	**Overall certainty in estimates‡**
**Non-fatal symptomatic venous thromboembolism**							
22,182 (17)	Serious limitations	No serious limitations	No serious limitations	No serious limitations	0.59	**Low: 0.42 Medium: 0.85 High: 1.69**	**Low**
**Fatal venous thromboembolism**							
22,182 (17)	Serious limitations	No serious limitations	No serious limitations	No serious limitations	0.02	**Low: 0.02 Medium: 0.03 High: 0.06**	**Very Low**
**Symptomatic splanchnic vein thrombosis§**							
1,355 (1)	Serious limitations	No serious limitations	No serious limitations	No serious limitations	0.07	**0.07**	**Low**
**Non-fatal bleeding requiring reintervention**							
1,971 (4)	No serious limitations	No serious limitations	No serious limitations	No serious limitations	0.40	**0.40**	**Moderate**
**Non-fatal bleeding leading to transfusion**							
13,245 (2)	Serious limitations	No serious limitations	No serious limitations	No serious limitations	4.50	**4.50**	**Low**
**Fatal bleeding**							
1,971 (4)	No serious limitations	No serious limitations	No serious limitations	No serious limitations	0.01	**0.01**	**Low**
**Bleeding leading to hemoglobin below 70g/L (7g/dL)**							

Blank spaces indicate absence of information

* Estimate represents absolute risk in percent. Our median best estimates include fatal and non-fatal events. Based on data from included studies, we estimated case fatality rates as follows: 3.6% for VTE, 3.6% for bleeding leading to reintervention, and 0.9% for bleeding leading to transfusion, and used this information to calculate outcome estimates. For instance, we multiplied the median VTE estimate by 0.964 for non-fatal VTE and by 0.036 for fatal VTE (if both reintervention and transfusion rates were available, we preferred reintervention estimates for calculation of fatal bleeding estimate).

† Risk factors included 1) age more than 75 years, 2) obesity (body mass index of 35 or more), 3) VTE in a first degree relative (parents, full siblings, or children), and 4) prior VTE. We assumed that patients with any combination of two or more risk factors had a risk ratio of 4. Using these risk factors, we then categorized risk of VTE as low, medium, and high risk.

‡ Options for certainty in estimates are high, moderate, low, and very low. Evidence begins as high and is rated down for serious risk of bias, inconsistency, imprecision, or indirectness. We always rated down once due to uncertainty in the patient VTE risk factors and models of timing of VTE and bleeding. For fatal VTE and fatal bleeding we always rated down once for uncertainty in our case fatality rate estimates.

§ The best median estimate for symptomatic splanchnic vein thrombosis is median value of reported estimates. As we did not find evidence for timing of SVT, effect of thromboprophylaxis on SVT or patient risk factors for SVT, we did not model splanchnic vein thrombosis estimates for these factors.

101. Evidence profile 101. Gastrectomy, robotic: Absolute risk of venous thromboembolism and bleeding among patients not receiving prophylaxis

Quality assessment					Summary of findings		
**No of participants (studies)**	**Risk of Bias**	**Inconsistency**	**Indirectness**	**Imprecision**	**Best (median) estimate across all risk strata (%)***	**Best (median estimate) by patient risk strata (%)†**	**Overall certainty in estimates‡**
**Non-fatal symptomatic venous thromboembolism**							
1,042 (5)	Serious limitations	Serious limitations	No serious limitations	No serious limitations	3.05	**Low: 2.03 Medium: 4.05 High: 8.11**	**Very low**
**Fatal venous thromboembolism**							
1,042 (5)	Serious limitations	Serious limitations	No serious limitations	No serious limitations	0.11	**Low: 0.08 Medium: 0.15 High: 0.3**	**Very low**
**Symptomatic splanchnic vein thrombosis§**							
115 (1)	Serious limitations	No serious limitations	No serious limitations	Very serious limitations	0.43	**0.43**	**Very low**
**Non-fatal bleeding requiring reintervention**							
950 (5)	No serious limitations	No serious limitations	No serious limitations	Serious limitations	0.21¶	**0.21¶**	**Low**
**Non-fatal bleeding leading to transfusion**							
100 (1)	Serious limitations	No serious limitations	No serious limitations	Very serious limitations	0.69	**0.69**	**Very low**
**Fatal bleeding**							
100 (1)	Serious limitations	No serious limitations	No serious limitations	Very serious limitations	0.01	**0.01**	**Very low**
**Bleeding leading to hemoglobin below 70g/L (7g/dL)**							

Blank spaces indicate absence of information

* Estimate represents absolute risk in percent. Our median best estimates include fatal and non-fatal events. Based on data from included studies, we estimated case fatality rates as follows: 3.6% for VTE, 3.6% for bleeding leading to reintervention, and 0.9% for bleeding leading to transfusion, and used this information to calculate outcome estimates. For instance, we multiplied the median VTE estimate by 0.964 for non-fatal VTE and by 0.036 for fatal VTE (if both reintervention and transfusion rates were available, we preferred reintervention estimates for calculation of fatal bleeding estimate).

† Risk factors included 1) age more than 75 years, 2) obesity (body mass index of 35 or more), 3) VTE in a first degree relative (parents, full siblings, or children), and 4) prior VTE. We assumed that patients with any combination of two or more risk factors had a risk ratio of 4. Using these risk factors, we then categorized risk of VTE as low, medium, and high risk.

‡ Options for certainty in estimates are high, moderate, low, and very low. Evidence begins as high and is rated down for serious risk of bias, inconsistency, imprecision, or indirectness. We always rated down once due to uncertainty in the patient VTE risk factors and models of timing of VTE and bleeding. For fatal VTE and fatal bleeding we always rated down once for uncertainty in our case fatality rate estimates.

§ The best median estimate for symptomatic splanchnic vein thrombosis is median value of reported estimates. As we did not find evidence for timing of SVT, effect of thromboprophylaxis on SVT or patient risk factors for SVT, we did not model splanchnic vein thrombosis estimates for these factors.

¶Reported median estimate in eligible studies for this procedure was 0,0%. As a real underlying risk of 0,0% is improbable we used average instead of median.

102. Evidence profile 102. Gastrectomy, open: Absolute risk of venous thromboembolism and bleeding among patients not receiving prophylaxis

Quality assessment					Summary of findings		
**No of participants (studies)**	**Risk of Bias**	**Inconsistency**	**Indirectness**	**Imprecision**	**Best (median) estimate across all risk strata (%)***	**Best (median estimate) by patient risk strata (%)†**	**Overall certainty in estimates‡**
**Non-fatal symptomatic venous thromboembolism**							
77,629 (13)	Serious limitations	Serious limitations	No serious limitations	No serious limitations	0.34	**Low: 0.23 Medium: 0.46 High: 0.92**	**Very low**
**Fatal venous thromboembolism**							
77,629 (13)	Serious limitations	Serious limitations	No serious limitations	No serious limitations	0.01	**Low: 0.01 Medium: 0.02 High: 0.03**	**Very low**
**Symptomatic splanchnic vein thrombosis§**							
3,256 (1)	Serious limitations	No serious limitations	No serious limitations	No serious limitations	0.37	**0.37**	**Low**
**Non-fatal bleeding requiring reintervention**							
1,258 (3)	Serious limitations	No serious limitations	No serious limitations	No serious limitations	0.37	**0.37**	**Low**
**Non-fatal bleeding leading to transfusion**							
46,050 (2)	Serious limitations	Serious limitations	No serious limitations	No serious limitations	11.17	**11.17**	**Very low**
**Fatal bleeding**							
1,258 (3)	Serious limitations	No serious limitations	No serious limitations	No serious limitations	0.01	**0.01**	**Very Low**
**Bleeding leading to hemoglobin below 70g/L (7g/dL)**							

Blank spaces indicate absence of information

* Estimate represents absolute risk in percent. Our median best estimates include fatal and non-fatal events. Based on data from included studies, we estimated case fatality rates as follows: 3.6% for VTE, 3.6% for bleeding leading to reintervention, and 0.9% for bleeding leading to transfusion, and used this information to calculate outcome estimates. For instance, we multiplied the median VTE estimate by 0.964 for non-fatal VTE and by 0.036 for fatal VTE (if both reintervention and transfusion rates were available, we preferred reintervention estimates for calculation of fatal bleeding estimate).

† Risk factors included 1) age more than 75 years, 2) obesity (body mass index of 35 or more), 3) VTE in a first degree relative (parents, full siblings, or children), and 4) prior VTE. We assumed that patients with any combination of two or more risk factors had a risk ratio of 4. Using these risk factors, we then categorized risk of VTE as low, medium, and high risk.

‡ Options for certainty in estimates are high, moderate, low, and very low. Evidence begins as high and is rated down for serious risk of bias, inconsistency, imprecision, or indirectness. We always rated down once due to uncertainty in the patient VTE risk factors and models of timing of VTE and bleeding. For fatal VTE and fatal bleeding we always rated down once for uncertainty in our case fatality rate estimates.

§ The best median estimate for symptomatic splanchnic vein thrombosis is median value of reported estimates. As we did not find evidence for timing of SVT, effect of thromboprophylaxis on SVT or patient risk factors for SVT, we did not model splanchnic vein thrombosis estimates for these factors.

103. Evidence profile 103. Subtotal gastrectomy, laparoscopic: Absolute risk of venous thromboembolism and bleeding among patients not receiving prophylaxis

Quality assessment					Summary of findings		
**No of participants (studies)**	**Risk of Bias**	**Inconsistency**	**Indirectness**	**Imprecision**	**Best (median) estimate across all risk strata (%)***	**Best (median estimate) by patient risk strata (%)†**	**Overall certainty in estimates‡**
**Non-fatal symptomatic venous thromboembolism**							
1,750 (4)	No serious limitations	No serious limitations	No serious limitations	No serious limitations	0.50	**Low: 0.34 Medium: 0.68 High: 1.37**	**Moderate**
**Fatal venous thromboembolism**							
1,750 (4)	No serious limitations	No serious limitations	No serious limitations	No serious limitations	0.02	**Low: 0.01 Medium: 0.03 High: 0.05**	**Low**
**Symptomatic splanchnic vein thrombosis**							
**Non-fatal bleeding requiring reintervention**							
61 (1)	Serious limitations	No serious limitations	No serious limitations	Very serious limitations	1.09	**1.09**	**Very low**
**Non-fatal bleeding leading to transfusion**							
**Fatal bleeding**							
61 (1)	Serious limitations	No serious limitations	No serious limitations	Very serious limitations	0.04	**0.04**	**Very low**
**Bleeding leading to hemoglobin below 70g/L (7g/dL)**							

Blank spaces indicate absence of information

* Estimate represents absolute risk in percent. Our median best estimates include fatal and non-fatal events. Based on data from included studies, we estimated case fatality rates as follows: 3.6% for VTE, 3.6% for bleeding leading to reintervention, and 0.9% for bleeding leading to transfusion, and used this information to calculate outcome estimates. For instance, we multiplied the median VTE estimate by 0.964 for non-fatal VTE and by 0.036 for fatal VTE (if both reintervention and transfusion rates were available, we preferred reintervention estimates for calculation of fatal bleeding estimate).

† Risk factors included 1) age more than 75 years, 2) obesity (body mass index of 35 or more), 3) VTE in a first degree relative (parents, full siblings, or children), and 4) prior VTE. We assumed that patients with any combination of two or more risk factors had a risk ratio of 4. Using these risk factors, we then categorized risk of VTE as low, medium, and high risk.

‡ Options for certainty in estimates are high, moderate, low, and very low. Evidence begins as high and is rated down for serious risk of bias, inconsistency, imprecision, or indirectness. We always rated down once due to uncertainty in the patient VTE risk factors and models of timing of VTE and bleeding. For fatal VTE and fatal bleeding we always rated down once for uncertainty in our case fatality rate estimates.

104. Evidence profile 104. Total gastrectomy, laparoscopic: Absolute risk of venous thromboembolism and bleeding among patients not receiving prophylaxis

Quality assessment					Summary of findings		
**No of participants (studies)**	**Risk of Bias**	**Inconsistency**	**Indirectness**	**Imprecision**	**Best (median) estimate across all risk strata (%)***	**Best (median estimate) by patient risk strata (%)†**	**Overall certainty in estimates‡**
**Non-fatal symptomatic venous thromboembolism**							
15,097 (3)	Serious limitations	No serious limitations	No serious limitations	No serious limitations	0.86	**Low: 0.64 Medium: 1.28 High: 2.56**	**Low**
**Fatal venous thromboembolism**							
15,097 (3)	Serious limitations	No serious limitations	No serious limitations	No serious limitations	0.03	**Low: 0.02 Medium: 0.05 High: 0.1**	**Very Low**
**Symptomatic splanchnic vein thrombosis**							
**Non-fatal bleeding requiring reintervention**							
**Non-fatal bleeding leading to transfusion**							
13,245 (2)	Serious limitations	No serious limitations	No serious limitations	No serious limitations	4.50	**4.50**	**Low**
**Fatal bleeding**							
13245 (2)	Serious limitations	Serious limitations	No serious limitations	No serious limitations	0.04	**0.04**	**Very low**
**Bleeding leading to hemoglobin below 70g/L (7g/dL)**							

Blank spaces indicate absence of information

* Estimate represents absolute risk in percent. Our median best estimates include fatal and non-fatal events. Based on data from included studies, we estimated case fatality rates as follows: 3.6% for VTE, 3.6% for bleeding leading to reintervention, and 0.9% for bleeding leading to transfusion, and used this information to calculate outcome estimates. For instance, we multiplied the median VTE estimate by 0.964 for non-fatal VTE and by 0.036 for fatal VTE (if both reintervention and transfusion rates were available, we preferred reintervention estimates for calculation of fatal bleeding estimate).

† Risk factors included 1) age more than 75 years, 2) obesity (body mass index of 35 or more), 3) VTE in a first degree relative (parents, full siblings, or children), and 4) prior VTE. We assumed that patients with any combination of two or more risk factors had a risk ratio of 4. Using these risk factors, we then categorized risk of VTE as low, medium, and high risk.

‡ Options for certainty in estimates are high, moderate, low, and very low. Evidence begins as high and is rated down for serious risk of bias, inconsistency, imprecision, or indirectness. We always rated down once due to uncertainty in the patient VTE risk factors and models of timing of VTE and bleeding. For fatal VTE and fatal bleeding we always rated down once for uncertainty in our case fatality rate estimates.

105. Evidence profile 105. Subtotal gastrectomy, open: Absolute risk of venous thromboembolism and bleeding among patients not receiving prophylaxis

Quality assessment					Summary of findings		
**No of participants (studies)**	**Risk of Bias**	**Inconsistency**	**Indirectness**	**Imprecision**	**Best (median) estimate across all risk strata (%)***	**Best (median estimate) by patient risk strata (%)†**	**Overall certainty in estimates‡**
**Non-fatal symptomatic venous thromboembolism**							
5,568 (4)	Serious limitations	No serious limitations	No serious limitations	No serious limitations	0.46	**Low: 0.28 Medium: 0.56 High: 1.11**	**Low**
**Fatal venous thromboembolism**							
5,568 (4)	Serious limitations	No serious limitations	No serious limitations	No serious limitations	0.02	**Low: 0.01 Medium: 0.02 High: 0.04**	**Very Low**
**Symptomatic splanchnic vein thrombosis§**							
**Non-fatal bleeding requiring reintervention**							
310 (1)	No serious limitations	No serious limitations	No serious limitations	Serious limitations	0.43	**0.43**	**Low**
**Non-fatal bleeding leading to transfusion**							
403 (1)	Serious limitations	No serious limitations	No serious limitations	Serious limitations	2.56	**2.56**	**Very low**
**Fatal bleeding**							
310 (1)	No serious limitations	No serious limitations	No serious limitations	Serious limitations	0.02	**0.02**	**Very Low**
**Bleeding leading to hemoglobin below 70g/L (7g/dL)**							

Blank spaces indicate absence of information
* Estimate represents absolute risk in percent. Our median best estimates include fatal and non-fatal events. Based on data from included studies, we estimated case fatality rates as follows: 3.6% for VTE, 3.6% for bleeding leading to reintervention, and 0.9% for bleeding leading to transfusion, and used this information to calculate outcome estimates. For instance, we multiplied the median VTE estimate by 0.964 for non-fatal VTE and by 0.036 for fatal VTE (if both reintervention and transfusion rates were available, we preferred reintervention estimates for calculation of fatal bleeding estimate).
† Risk factors included 1) age more than 75 years, 2) obesity (body mass index of 35 or more), 3) VTE in a first degree relative (parents, full siblings, or children), and 4) prior VTE. We assumed that patients with any combination of two or more risk factors had a risk ratio of 4. Using these risk factors, we then categorized risk of VTE as low, medium, and high risk.
‡ Options for certainty in estimates are high, moderate, low, and very low. Evidence begins as high and is rated down for serious risk of bias, inconsistency, imprecision, or indirectness. We always rated down once due to uncertainty in the patient VTE risk factors and models of timing of VTE and bleeding. For fatal VTE and fatal bleeding we always rated down once for uncertainty in our case fatality rate estimates.
§We did not model splanchnic vein thrombosis estimates for timing, use of thromboprophylaxis or patient risk factors as we did not find available evidence for timing of SVT, effect of thromboprophylaxis on SVT or patient risk factors for SVT.

106. Evidence profile 106. Total gastrectomy, open: Absolute risk of venous thromboembolism and bleeding among patients not receiving prophylaxis

Quality assessment					Summary of findings		
**No of participants (studies)**	**Risk of Bias**	**Inconsistency**	**Indirectness**	**Imprecision**	**Best (median) estimate across all risk strata (%)***	**Best (median estimate) by patient risk strata (%)†**	**Overall certainty in estimates‡**
**Non-fatal symptomatic venous thromboembolism**							
47,938 (5)	Serious limitations	Serious limitations	No serious limitations	No serious limitations	0.81	**Low: 0.61 Medium: 1.21 High: 2.43**	**Very low**
**Fatal venous thromboembolism**							
47,938 (5)	Serious limitations	Serious limitations	No serious limitations	No serious limitations	0.03	**Low: 0.02 Medium: 0.05 High: 0.09**	**Very low**
**Symptomatic splanchnic vein thrombosis**							
**Non-fatal bleeding requiring reintervention**							
**Non-fatal bleeding leading to transfusion**							
45,647 (2)	Serious limitations	Serious limitations	No serious limitations	No serious limitations	11.16	**11.16**	**Very low**
**Fatal bleeding**							
45,647 (2)	Serious limitations	Serious limitations	No serious limitations	No serious limitations	0.10	**0.10**	**Very low**
**Bleeding leading to hemoglobin below 70g/L (7g/dL)**							

Blank spaces indicate absence of information

* Estimate represents absolute risk in percent. Our median best estimates include fatal and non-fatal events. Based on data from included studies, we estimated case fatality rates as follows: 3.6% for VTE, 3.6% for bleeding leading to reintervention, and 0.9% for bleeding leading to transfusion, and used this information to calculate outcome estimates. For instance, we multiplied the median VTE estimate by 0.964 for non-fatal VTE and by 0.036 for fatal VTE (if both reintervention and transfusion rates were available, we preferred reintervention estimates for calculation of fatal bleeding estimate).

† Risk factors included 1) age more than 75 years, 2) obesity (body mass index of 35 or more), 3) VTE in a first degree relative (parents, full siblings, or children), and 4) prior VTE. We assumed that patients with any combination of two or more risk factors had a risk ratio of 4. Using these risk factors, we then categorized risk of VTE as low, medium, and high risk.

‡ Options for certainty in estimates are high, moderate, low, and very low. Evidence begins as high and is rated down for serious risk of bias, inconsistency, imprecision, or indirectness. We always rated down once due to uncertainty in the patient VTE risk factors and models of timing of VTE and bleeding. For fatal VTE and fatal bleeding we always rated down once for uncertainty in our case fatality rate estimates.

107. Evidence profile 107. Gastrectomy, minimally-invasive, in Asia: Absolute risk of venous thromboembolism and bleeding among patients not receiving prophylaxis

Quality assessment					Summary of findings		
**No of participants (studies)**	**Risk of Bias**	**Inconsistency**	**Indirectness**	**Imprecision**	**Best (median) estimate across all risk strata (%)***	**Best (median estimate) by patient risk strata (%)†**	**Overall certainty in estimates‡**
**Non-fatal symptomatic venous thromboembolism**							
20,995 (15)	Serious limitations	No serious limitations	No serious limitations	No serious limitations	0.40	**Low: 0.29 Medium: 0.58 High: 1.16**	**Low**
**Fatal venous thromboembolism**							
20,995 (15)	Serious limitations	No serious limitations	No serious limitations	No serious limitations	0.02	**Low: 0.01 Medium: 0.02 High: 0.04**	**Very Low**
**Symptomatic splanchnic vein thrombosis§**							
1,470 (2)	Serious limitations	No serious limitations	No serious limitations	No serious limitations	0.15	**0.15**	**Low**
**Non-fatal bleeding requiring reoperation**							
2,413 (6)	No serious limitations	No serious limitations	No serious limitations	No serious limitations	0.30	**0.30**	**Moderate**
**Non-fatal bleeding leading to transfusion**							
13,345 (3)	Serious limitations	Serious limitations	No serious limitations	No serious limitations	2.54	**2.54**	**Very low**
**Fatal bleeding**							
2,413 (6)	No serious limitations	No serious limitations	No serious limitations	No serious limitations	0.01	**0.01**	**Low**
**Bleeding leading to hemoglobin below 70g/L (7g/dL)**							

Blank spaces indicate absence of information

* Estimate represents absolute risk in percent. Our median best estimates include fatal and non-fatal events. Based on data from included studies, we estimated case fatality rates as follows: 3.6% for VTE, 3.6% for bleeding leading to reintervention, and 0.9% for bleeding leading to transfusion, and used this information to calculate outcome estimates. For instance, we multiplied the median VTE estimate by 0.964 for non-fatal VTE and by 0.036 for fatal VTE (if both reintervention and transfusion rates were available, we preferred reintervention estimates for calculation of fatal bleeding estimate).

† Risk factors included 1) age more than 75 years, 2) obesity (body mass index of 35 or more), 3) VTE in a first degree relative (parents, full siblings, or children), and 4) prior VTE. We assumed that patients with any combination of two or more risk factors had a risk ratio of 4. Using these risk factors, we then categorized risk of VTE as low, medium, and high risk.

‡ Options for certainty in estimates are high, moderate, low, and very low. Evidence begins as high and is rated down for serious risk of bias, inconsistency, imprecision, or indirectness. We always rated down once due to uncertainty in the patient VTE risk factors and models of timing of VTE and bleeding. For fatal VTE and fatal bleeding we always rated down once for uncertainty in our case fatality rate estimates.

§ The best median estimate for symptomatic splanchnic vein thrombosis is median value of reported estimates. As we did not find evidence for timing of SVT, effect of thromboprophylaxis on SVT or patient risk factors for SVT, we did not model splanchnic vein thrombosis estimates for these factors.

108. Evidence profile 108. Gastrectomy, laparoscopic, in Asia: Absolute risk of venous thromboembolism and bleeding among patients not receiving prophylaxis

Quality assessment					Summary of findings		
**No of participants (studies)**	**Risk of Bias**	**Inconsistency**	**Indirectness**	**Imprecision**	**Best (median) estimate across all risk strata (%)***	**Best (median estimate) by patient risk strata (%)†**	**Overall certainty in estimates‡**
**Non-fatal symptomatic venous thromboembolism**							
20,852 (14)	Serious limitations	No serious limitations	No serious limitations	No serious limitations	0.32	**Low: 0.23 Medium: 0.45 High: 0.91**	**Low**
**Fatal venous thromboembolism**							
20,852 (14)	Serious limitations	No serious limitations	No serious limitations	No serious limitations	0.01	**Low: 0.01 Medium: 0.02 High: 0.03**	**Very Low**
**Symptomatic splanchnic vein thrombosis§**							
1,355 (1)	Serious limitations	No serious limitations	No serious limitations	No serious limitations	0.07	**0.07**	**Low**
**Non-fatal bleeding requiring reintervention**							
1,910 (3)	No serious limitations	No serious limitations	No serious limitations	No serious limitations	0.33	**0.33**	**Moderate**
**Non-fatal bleeding leading to transfusion**							
13,245 (2)	Serious limitations	Serious limitations	No serious limitations	No serious limitations	4.50	**4.50**	**Very low**
**Fatal bleeding**							
1,910 (3)	No serious limitations	No serious limitations	No serious limitations	No serious limitations	0.01	**0.01**	**Low**
**Bleeding leading to hemoglobin below 70g/L (7g/dL)**							

Blank spaces indicate absence of information

* Estimate represents absolute risk in percent. Our median best estimates include fatal and non-fatal events. Based on data from included studies, we estimated case fatality rates as follows: 3.6% for VTE, 3.6% for bleeding leading to reintervention, and 0.9% for bleeding leading to transfusion, and used this information to calculate outcome estimates. For instance, we multiplied the median VTE estimate by 0.964 for non-fatal VTE and by 0.036 for fatal VTE (if both reintervention and transfusion rates were available, we preferred reintervention estimates for calculation of fatal bleeding estimate).

† Risk factors included 1) age more than 75 years, 2) obesity (body mass index of 35 or more), 3) VTE in a first degree relative (parents, full siblings, or children), and 4) prior VTE. We assumed that patients with any combination of two or more risk factors had a risk ratio of 4. Using these risk factors, we then categorized risk of VTE as low, medium, and high risk.

‡ Options for certainty in estimates are high, moderate, low, and very low. Evidence begins as high and is rated down for serious risk of bias, inconsistency, imprecision, or indirectness. We always rated down once due to uncertainty in the patient VTE risk factors and models of timing of VTE and bleeding. For fatal VTE and fatal bleeding we always rated down once for uncertainty in our case fatality rate estimates.

§ The best median estimate for symptomatic splanchnic vein thrombosis is median value of reported estimates. As we did not find evidence for timing of SVT, effect of thromboprophylaxis on SVT or patient risk factors for SVT, we did not model splanchnic vein thrombosis estimates for these factors.

109. Evidence profile 109. Gastrectomy, robotic, in Asia: Absolute risk of venous thromboembolism and bleeding among patients not receiving prophylaxis

Quality assessment					Summary of findings		
**No of participants (studies)**	**Risk of Bias**	**Inconsistency**	**Indirectness**	**Imprecision**	**Best (median) estimate across all risk strata (%)***	**Best (median estimate) by patient risk strata (%)†**	**Overall certainty in estimates‡**
**Non-fatal symptomatic venous thromboembolism**							
819 (4)	No serious limitations	No serious limitations	No serious limitations	Serious limitations	2.14	**Low: 1.42 Medium: 2.84 High: 5.69**	**Low**
**Fatal venous thromboembolism**							
819 (4)	No serious limitations	No serious limitations	No serious limitations	Serious limitations	0.08	**Low: 0.05 Medium: 0.11 High: 0.21**	**Very Low**
**Symptomatic splanchnic vein thrombosis§**							
115 (1)	Serious limitations	No serious limitations	No serious limitations	Very serious limitations	0.43	**0.43**	**Very low**
**Non-fatal bleeding requiring reintervention**							
950 (5)	No serious limitations	No serious limitations	No serious limitations	Serious limitations	0.21¶	**0.21¶**	**Low**
**Non-fatal bleeding leading to transfusion**							
100 (1)	Serious limitations	No serious limitations	No serious limitations	Very serious limitations	0.69	**0.69**	**Very low**
**Fatal bleeding**							
100 (1)	Serious limitations	No serious limitations	No serious limitations	Very serious limitations	0.01	**0.01**	**Very low**
**Bleeding leading to hemoglobin below 70g/L (7g/dL)**							

Blank spaces indicate absence of information

* Estimate represents absolute risk in percent. Our median best estimates include fatal and non-fatal events. Based on data from included studies, we estimated case fatality rates as follows: 3.6% for VTE, 3.6% for bleeding leading to reintervention, and 0.9% for bleeding leading to transfusion, and used this information to calculate outcome estimates. For instance, we multiplied the median VTE estimate by 0.964 for non-fatal VTE and by 0.036 for fatal VTE (if both reintervention and transfusion rates were available, we preferred reintervention estimates for calculation of fatal bleeding estimate).

† Risk factors included 1) age more than 75 years, 2) obesity (body mass index of 35 or more), 3) VTE in a first degree relative (parents, full siblings, or children), and 4) prior VTE. We assumed that patients with any combination of two or more risk factors had a risk ratio of 4. Using these risk factors, we then categorized risk of VTE as low, medium, and high risk.

‡ Options for certainty in estimates are high, moderate, low, and very low. Evidence begins as high and is rated down for serious risk of bias, inconsistency, imprecision, or indirectness. We always rated down once due to uncertainty in the patient VTE risk factors and models of timing of VTE and bleeding. For fatal VTE and fatal bleeding we always rated down once for uncertainty in our case fatality rate estimates.

§ The best median estimate for symptomatic splanchnic vein thrombosis is median value of reported estimates. As we did not find evidence for timing of SVT, effect of thromboprophylaxis on SVT or patient risk factors for SVT, we did not model splanchnic vein thrombosis estimates for these factors.

¶Reported median estimate in eligible studies for this procedure was 0,0%. As a real underlying risk of 0,0% is improbable we used average instead of median..

110. Evidence profile 110. Gastrectomy, open, in Asia: Absolute risk of venous thromboembolism and bleeding among patients not receiving prophylaxis

Quality assessment					Summary of findings		
**No of participants (studies)**	**Risk of Bias**	**Inconsistency**	**Indirectness**	**Imprecision**	**Best (median) estimate across all risk strata (%)***	**Best (median estimate) by patient risk strata (%)†**	**Overall certainty in estimates‡**
**Non-fatal symptomatic venous thromboembolism**							
54,950 (8)	Serious limitations	No serious limitations	No serious limitations	No serious limitations	0.20	**Low: 0.15 Medium: 0.3 High: 0.6**	**Low**
**Fatal venous thromboembolism**							
54,950 (8)	Serious limitations	No serious limitations	No serious limitations	No serious limitations	0.01	**Low: 0.01 Medium: 0.01 High: 0.02**	**Very Low**
**Symptomatic splanchnic vein thrombosis§**							
3,256 (1)	Serious limitations	No serious limitations	No serious limitations	No serious limitations	0.37	**0.37**	**Low**
**Non-fatal bleeding requiring reintervention**							
768 (1)	Serious limitations	No serious limitations	No serious limitations	Serious limitations	0.28	**0.28**	**Very low**
**Non-fatal bleeding leading to transfusion**							
46,050 (2)	Serious limitations	Serious limitations	No serious limitations	No serious limitations	11.17	**11.17**	**Very low**
**Fatal bleeding**							
768 (1)	Serious limitations	No serious limitations	No serious limitations	Serious limitations	0.01	**0.01**	**Very low**
**Bleeding leading to hemoglobin below 70g/L (7g/dL)**							

Blank spaces indicate absence of information

* Estimate represents absolute risk in percent. Our median best estimates include fatal and non-fatal events. Based on data from included studies, we estimated case fatality rates as follows: 3.6% for VTE, 3.6% for bleeding leading to reintervention, and 0.9% for bleeding leading to transfusion, and used this information to calculate outcome estimates. For instance, we multiplied the median VTE estimate by 0.964 for non-fatal VTE and by 0.036 for fatal VTE (if both reintervention and transfusion rates were available, we preferred reintervention estimates for calculation of fatal bleeding estimate).

† Risk factors included 1) age more than 75 years, 2) obesity (body mass index of 35 or more), 3) VTE in a first degree relative (parents, full siblings, or children), and 4) prior VTE. We assumed that patients with any combination of two or more risk factors had a risk ratio of 4. Using these risk factors, we then categorized risk of VTE as low, medium, and high risk.

‡ Options for certainty in estimates are high, moderate, low, and very low. Evidence begins as high and is rated down for serious risk of bias, inconsistency, imprecision, or indirectness. We always rated down once due to uncertainty in the patient VTE risk factors and models of timing of VTE and bleeding. For fatal VTE and fatal bleeding we always rated down once for uncertainty in our case fatality rate estimates.

§ The best median estimate for symptomatic splanchnic vein thrombosis is median value of reported estimates. As we did not find evidence for timing of SVT, effect of thromboprophylaxis on SVT or patient risk factors for SVT, we did not model splanchnic vein thrombosis estimates for these factors.

111. Evidence profile 111. Subtotal gastrectomy, laparoscopic, in Asia: Absolute risk of venous thromboembolism and bleeding among patients not receiving prophylaxis

Quality assessment					Summary of findings		
No of participants (studies)	Risk of Bias	Inconsistency	Indirectness	Imprecision	Best (median) estimate across all risk strata (%)*	Best (median estimate) by patient risk strata (%)†	Overall certainty in estimates‡
**Non-fatal symptomatic venous thromboembolism**							
1,689 (3)	No serious limitations	No serious limitations	No serious limitations	No serious limitations	0.40	**Low: 0.24 Medium: 0.49 High: 0.97**	**Moderate**
**Fatal venous thromboembolism**							
1,689 (3)	No serious limitations	No serious limitations	No serious limitations	No serious limitations	0.02	**Low: 0.01 Medium: 0.02 High: 0.04**	**Low**
**Symptomatic splanchnic vein thrombosis**							
**Non-fatal bleeding requiring reintervention**							
**Non-fatal bleeding leading to transfusion**							
**Fatal bleeding**							
**Bleeding leading to hemoglobin below 70g/L (7g/dL)**							

Blank spaces indicate absence of information
We did not find any studies including robotic procedures for this procedure.
* Estimate represents absolute risk in percent. Our median best estimates include fatal and non-fatal events. Based on data from included studies, we estimated case fatality rates as follows: 3.6% for VTE, 3.6% for bleeding leading to reintervention, and 0.9% for bleeding leading to transfusion, and used this information to calculate outcome estimates. For instance, we multiplied the median VTE estimate by 0.964 for non-fatal VTE and by 0.036 for fatal VTE (if both reintervention and transfusion rates were available, we preferred reintervention estimates for calculation of fatal bleeding estimate).
† Risk factors included 1) age more than 75 years, 2) obesity (body mass index of 35 or more), 3) VTE in a first degree relative (parents, full siblings, or children), and 4) prior VTE. We assumed that patients with any combination of two or more risk factors had a risk ratio of 4. Using these risk factors, we then categorized risk of VTE as low, medium, and high risk.
‡ Options for certainty in estimates are high, moderate, low, and very low. Evidence begins as high and is rated down for serious risk of bias, inconsistency, imprecision, or indirectness. We always rated down once due to uncertainty in the patient VTE risk factors and models of timing of VTE and bleeding. For fatal VTE and fatal bleeding we always rated down once for uncertainty in our case fatality rate estimates.

112. Evidence profile 112. Total gastrectomy, laparoscopic, in Asia: Absolute risk of venous thromboembolism and bleeding among patients not receiving prophylaxis

Quality assessment					Summary of findings		
**No of participants (studies)**	**Risk of Bias**	**Inconsistency**	**Indirectness**	**Imprecision**	**Best (median) estimate across all risk strata (%)***	**Best (median estimate) by patient risk strata (%)†**	**Overall certainty in estimates‡**
**Non-fatal symptomatic venous thromboembolism**							
15,097 (3)	Serious limitations	No serious limitations	No serious limitations	No serious limitations	0.85	**Low: 0.63 Medium: 1.26 High: 2.53**	**Low**
**Fatal venous thromboembolism**							
15,097 (3)	Serious limitations	No serious limitations	No serious limitations	No serious limitations	0.03	**Low: 0.02 Medium: 0.05 High: 0.09**	**Very Low**
**Symptomatic splanchnic vein thrombosis**							
**Non-fatal bleeding requiring reintervention**							
**Non-fatal bleeding leading to transfusion**							
13,245 (2)	Serious limitations	Serious limitations	No serious limitations	No serious limitations	4.50	**4.50**	**Very low**
**Fatal bleeding**							
13245 (2)	Serious limitations	Serious limitations	No serious limitations	No serious limitations	0.04	**0.04**	**Very low**
**Bleeding leading to hemoglobin below 70g/L (7g/dL)**							

Blank spaces indicate absence of information

* Estimate represents absolute risk in percent. Our median best estimates include fatal and non-fatal events. Based on data from included studies, we estimated case fatality rates as follows: 3.6% for VTE, 3.6% for bleeding leading to reintervention, and 0.9% for bleeding leading to transfusion, and used this information to calculate outcome estimates. For instance, we multiplied the median VTE estimate by 0.964 for non-fatal VTE and by 0.036 for fatal VTE (if both reintervention and transfusion rates were available, we preferred reintervention estimates for calculation of fatal bleeding estimate).

† Risk factors included 1) age more than 75 years, 2) obesity (body mass index of 35 or more), 3) VTE in a first degree relative (parents, full siblings, or children), and 4) prior VTE. We assumed that patients with any combination of two or more risk factors had a risk ratio of 4. Using these risk factors, we then categorized risk of VTE as low, medium, and high risk.

‡ Options for certainty in estimates are high, moderate, low, and very low. Evidence begins as high and is rated down for serious risk of bias, inconsistency, imprecision, or indirectness. We always rated down once due to uncertainty in the patient VTE risk factors and models of timing of VTE and bleeding. For fatal VTE and fatal bleeding we always rated down once for uncertainty in our case fatality rate estimates.

113. Evidence profile 113. Subtotal gastrectomy, open, in Asia: Absolute risk of venous thromboembolism and bleeding among patients not receiving prophylaxis

Quality assessment					Summary of findings		
**No of participants (studies)**	**Risk of Bias**	**Inconsistency**	**Indirectness**	**Imprecision**	**Best (median) estimate across all risk strata (%)***	**Best (median estimate) by patient risk strata (%)†**	**Overall certainty in estimates‡**
**Non-fatal symptomatic venous thromboembolism**							
3,987 (3)	Serious limitations	No serious limitations	No serious limitations	No serious limitations	0.16	**Low: 0.12 Medium: 0.24 High: 0.48**	**Low**
**Fatal venous thromboembolism**							
3,987 (3)	Serious limitations	No serious limitations	No serious limitations	No serious limitations	0.01	**Low: 0 Medium: 0.01 High: 0.02**	**Very Low**
**Symptomatic splanchnic vein thrombosis**							
**Non-fatal bleeding requiring reintervention**							
**Non-fatal bleeding leading to transfusion**							
403 (1)	Serious limitations	No serious limitations	No serious limitations	Serious limitations	2.56	**2.56**	**Very low**
**Fatal bleeding**							
403 (1)	Serious limitations	No serious limitations	No serious limitations	Serious limitations	0.02	**0.02**	**Very low!**
**Bleeding leading to hemoglobin below 70g/L (7g/dL)**							

Blank spaces indicate absence of information

* Estimate represents absolute risk in percent. Our median best estimates include fatal and non-fatal events. Based on data from included studies, we estimated case fatality rates as follows: 3.6% for VTE, 3.6% for bleeding leading to reintervention, and 0.9% for bleeding leading to transfusion, and used this information to calculate outcome estimates. For instance, we multiplied the median VTE estimate by 0.964 for non-fatal VTE and by 0.036 for fatal VTE (if both reintervention and transfusion rates were available, we preferred reintervention estimates for calculation of fatal bleeding estimate).

† Risk factors included 1) age more than 75 years, 2) obesity (body mass index of 35 or more), 3) VTE in a first degree relative (parents, full siblings, or children), and 4) prior VTE. We assumed that patients with any combination of two or more risk factors had a risk ratio of 4. Using these risk factors, we then categorized risk of VTE as low, medium, and high risk.

‡ Options for certainty in estimates are high, moderate, low, and very low. Evidence begins as high and is rated down for serious risk of bias, inconsistency, imprecision, or indirectness. We always rated down once due to uncertainty in the patient VTE risk factors and models of timing of VTE and bleeding. For fatal VTE and fatal bleeding we always rated down once for uncertainty in our case fatality rate estimates.

114. Evidence profile 114. Total gastrectomy, open, in Asia: Absolute risk of venous thromboembolism and bleeding among patients not receiving prophylaxis

Quality assessment					Summary of findings		
**No of participants (studies)**	**Risk of Bias**	**Inconsistency**	**Indirectness**	**Imprecision**	**Best (median) estimate across all risk strata (%)***	**Best (median estimate) by patient risk strata (%)†**	**Overall certainty in estimates‡**
**Non-fatal symptomatic venous thromboembolism**							
46,939 (4)	Serious limitations	No serious limitations	No serious limitations	No serious limitations	0.52	**Low: 0.4 Medium: 0.8 High: 1.61**	**Low**
**Fatal venous thromboembolism**							
46,939 (4)	Serious limitations	No serious limitations	No serious limitations	No serious limitations	0.02	**Low: 0.01 Medium: 0.03 High: 0.06**	**Very Low**
**Symptomatic splanchnic vein thrombosis**							
**Non-fatal bleeding requiring reintervention**							
**Non-fatal bleeding leading to transfusion**							
45,647 (2)	Serious limitations	Serious limitations	No serious limitations	No serious limitations	11.16	**11.16**	**Very low**
**Fatal bleeding**							
45,647 (2)	Serious limitations	Serious limitations	No serious limitations	No serious limitations	0.10	**0.10**	**Very low**
**Bleeding leading to hemoglobin below 70g/L (7g/dL)**							

Blank spaces indicate absence of information

* Estimate represents absolute risk in percent. Our median best estimates include fatal and non-fatal events. Based on data from included studies, we estimated case fatality rates as follows: 3.6% for VTE, 3.6% for bleeding leading to reintervention, and 0.9% for bleeding leading to transfusion, and used this information to calculate outcome estimates. For instance, we multiplied the median VTE estimate by 0.964 for non-fatal VTE and by 0.036 for fatal VTE (if both reintervention and transfusion rates were available, we preferred reintervention estimates for calculation of fatal bleeding estimate).

† Risk factors included 1) age more than 75 years, 2) obesity (body mass index of 35 or more), 3) VTE in a first degree relative (parents, full siblings, or children), and 4) prior VTE. We assumed that patients with any combination of two or more risk factors had a risk ratio of 4. Using these risk factors, we then categorized risk of VTE as low, medium, and high risk.

‡ Options for certainty in estimates are high, moderate, low, and very low. Evidence begins as high and is rated down for serious risk of bias, inconsistency, imprecision, or indirectness. We always rated down once due to uncertainty in the patient VTE risk factors and models of timing of VTE and bleeding. For fatal VTE and fatal bleeding we always rated down once for uncertainty in our case fatality rate estimates.

115. Evidence profile 115. Gastrectomy, minimally-invasive, in non-Asian countries: Absolute risk of venous thromboembolism and bleeding among patients not receiving prophylaxis

Quality assessment					Summary of findings		
**No of participants (studies)**	**Risk of Bias**	**Inconsistency**	**Indirectness**	**Imprecision**	**Best (median) estimate across all risk strata (%)***	**Best (median estimate) by patient risk strata (%)†**	**Overall certainty in estimates‡**
**Non-fatal symptomatic venous thromboembolism**							
1,553 (3)	Serious limitations	Serious limitations	No serious limitations	No serious limitations	2.48	**Low: 1.69 Medium: 3.39 High: 6.78**	**Very low**
**Fatal venous thromboembolism**							
1,553 (3)	Serious limitations	Serious limitations	No serious limitations	No serious limitations	0.09	**Low: 0.06 Medium: 0.13 High: 0.25**	**Very low**
**Symptomatic splanchnic vein thrombosis**							
**Non-fatal bleeding requiring reintervention**							
61 (1)	Serious limitations	No serious limitations	No serious limitations	Very serious limitations	1.08	**1.08**	**Very low**
**Non-fatal bleeding leading to transfusion**							
**Fatal bleeding**							
61 (1)	Serious limitations	No serious limitations	No serious limitations	Very serious limitations	0.04	**0.04**	**Very low**
**Bleeding leading to hemoglobin below 70g/L (7g/dL)**							

Blank spaces indicate absence of information

* Estimate represents absolute risk in percent. Our median best estimates include fatal and non-fatal events. Based on data from included studies, we estimated case fatality rates as follows: 3.6% for VTE, 3.6% for bleeding leading to reintervention, and 0.9% for bleeding leading to transfusion, and used this information to calculate outcome estimates. For instance, we multiplied the median VTE estimate by 0.964 for non-fatal VTE and by 0.036 for fatal VTE (if both reintervention and transfusion rates were available, we preferred reintervention estimates for calculation of fatal bleeding estimate).

† Risk factors included 1) age more than 75 years, 2) obesity (body mass index of 35 or more), 3) VTE in a first degree relative (parents, full siblings, or children), and 4) prior VTE. We assumed that patients with any combination of two or more risk factors had a risk ratio of 4. Using these risk factors, we then categorized risk of VTE as low, medium, and high risk.

‡ Options for certainty in estimates are high, moderate, low, and very low. Evidence begins as high and is rated down for serious risk of bias, inconsistency, imprecision, or indirectness. We always rated down once due to uncertainty in the patient VTE risk factors and models of timing of VTE and bleeding. For fatal VTE and fatal bleeding we always rated down once for uncertainty in our case fatality rate estimates.

116. Evidence profile 116. Gastrectomy, laparoscopic, in non-Asian countries: Absolute risk of venous thromboembolism and bleeding among patients not receiving prophylaxis

Quality assessment					Summary of findings		
**No of participants (studies)**	**Risk of Bias**	**Inconsistency**	**Indirectness**	**Imprecision**	**Best (median) estimate across all risk strata (%)***	**Best (median estimate) by patient risk strata (%)†**	**Overall certainty in estimates‡**
**Non-fatal symptomatic venous thromboembolism**							
1,330 (3)	Serious limitations	Serious limitations	No serious limitations	No serious limitations	2.48	**Low: 1.69 Medium: 3.39 High: 6.78**	**Very low**
**Fatal venous thromboembolism**							
1,330 (3)	Serious limitations	Serious limitations	No serious limitations	No serious limitations	0.09	**Low: 0.06 Medium: 0.13 High: 0.25**	**Very low**
**Symptomatic splanchnic vein thrombosis**							
**Non-fatal bleeding requiring reoperation**							
61 (1)	Serious limitations	No serious limitations	No serious limitations	Very serious limitations	1.09	**1.09**	**Very low**
**Non-fatal bleeding leading to transfusion**							
**Fatal bleeding**							
61 (1)	Serious limitations	No serious limitations	No serious limitations	Very serious limitations	0.04	**0.04**	**Very low**
**Bleeding leading to hemoglobin below 70g/L (7g/dL)**							

Blank spaces indicate absence of information
We did not find any studies including robotic procedures for this procedure.
* Estimate represents absolute risk in percent. Our median best estimates include fatal and non-fatal events. Based on data from included studies, we estimated case fatality rates as follows: 3.6% for VTE, 3.6% for bleeding leading to reintervention, and 0.9% for bleeding leading to transfusion, and used this information to calculate outcome estimates. For instance, we multiplied the median VTE estimate by 0.964 for non-fatal VTE and by 0.036 for fatal VTE (if both reintervention and transfusion rates were available, we preferred reintervention estimates for calculation of fatal bleeding estimate).
† Risk factors included 1) age more than 75 years, 2) obesity (body mass index of 35 or more), 3) VTE in a first degree relative (parents, full siblings, or children), and 4) prior VTE. We assumed that patients with any combination of two or more risk factors had a risk ratio of 4. Using these risk factors, we then categorized risk of VTE as low, medium, and high risk.
‡ Options for certainty in estimates are high, moderate, low, and very low. Evidence begins as high and is rated down for serious risk of bias, inconsistency, imprecision, or indirectness. We always rated down once due to uncertainty in the patient VTE risk factors and models of timing of VTE and bleeding. For fatal VTE and fatal bleeding we always rated down once for uncertainty in our case fatality rate estimates.

117. Evidence profile 117. Gastrectomy, robotic, in non-Asian countries: Absolute risk of venous thromboembolism and bleeding among patients not receiving prophylaxis

Quality assessment					Summary of findings		
**No of participants (studies)**	**Risk of Bias**	**Inconsistency**	**Indirectness**	**Imprecision**	**Best (median) estimate across all risk strata (%)***	**Best (median estimate) by patient risk strata (%)†**	**Overall certainty in estimates‡**
**Non-fatal symptomatic venous thromboembolism**							
223 (1)	Serious limitations	No serious limitations	No serious limitations	Serious limitations	11.59	**Low: 7.92 Medium: 15.85 High: 31.69**	**Very low**
**Fatal venous thromboembolism**							
223 (1)	Serious limitations	No serious limitations	No serious limitations	Serious limitations	0.43	**Low: 0.3 Medium: 0.59 High: 1.18**	**Very low**
**Symptomatic splanchnic vein thrombosis**							
**Non-fatal bleeding requiring reoperation**							
**Non-fatal bleeding leading to transfusion**							
**Fatal bleeding**							
**Bleeding leading to hemoglobin below 70g/L (7g/dL)**							

Blank spaces indicate absence of information
* Estimate represents absolute risk in percent. Our median best estimates include fatal and non-fatal events. Based on data from included studies, we estimated case fatality rates as follows: 3.6% for VTE, 3.6% for bleeding leading to reintervention, and 0.9% for bleeding leading to transfusion, and used this information to calculate outcome estimates. For instance, we multiplied the median VTE estimate by 0.964 for non-fatal VTE and by 0.036 for fatal VTE (if both reintervention and transfusion rates were available, we preferred reintervention estimates for calculation of fatal bleeding estimate).
† Risk factors included 1) age more than 75 years, 2) obesity (body mass index of 35 or more), 3) VTE in a first degree relative (parents, full siblings, or children), and 4) prior VTE. We assumed that patients with any combination of two or more risk factors had a risk ratio of 4. Using these risk factors, we then categorized risk of VTE as low, medium, and high risk.
‡ Options for certainty in estimates are high, moderate, low, and very low. Evidence begins as high and is rated down for serious risk of bias, inconsistency, imprecision, or indirectness. We always rated down once due to uncertainty in the patient VTE risk factors and models of timing of VTE and bleeding. For fatal VTE and fatal bleeding we always rated down once for uncertainty in our case fatality rate estimates.
¶Reported median estimate in eligible studies for this procedure was 0,0%. As a real underlying risk of 0,0% is improbable we used average instead of median..

118. Evidence profile 118. Gastrectomy, open, in non-Asian countries: Absolute risk of venous thromboembolism and bleeding among patients not receiving prophylaxis

Quality assessment					Summary of findings		
**No of participants (studies)**	**Risk of Bias**	**Inconsistency**	**Indirectness**	**Imprecision**	**Best (median) estimate across all risk strata (%)***	**Best (median estimate) by patient risk strata (%)†**	**Overall certainty in estimates‡**
**Non-fatal symptomatic venous thromboembolism**							
22,679 (5)	Serious limitations	Serious limitations	No serious limitations	No serious limitations	3.14	**Low: 1.8 Medium: 3.61 High: 7.21**	**Very low**
**Fatal venous thromboembolism**							
22,679 (5)	Serious limitations	Serious limitations	No serious limitations	No serious limitations	0.12	**Low: 0.07 Medium: 0.13 High: 0.27**	**Very low**
**Symptomatic splanchnic vein thrombosis**							
**Non-fatal bleeding requiring reoperation**							
490 (2)	No serious limitations	No serious limitations	No serious limitations	Serious limitations	0.40	**0.40**	**Low**
**Non-fatal bleeding leading to transfusion**							
**Fatal bleeding**							
490 (2)	No serious limitations	No serious limitations	No serious limitations	Serious limitations	0.01	**0.01**	**Very Low**
**Bleeding leading to hemoglobin below 70g/L (7g/dL)**							

Blank spaces indicate absence of information

* Estimate represents absolute risk in percent. Our median best estimates include fatal and non-fatal events. Based on data from included studies, we estimated case fatality rates as follows: 3.6% for VTE, 3.6% for bleeding leading to reintervention, and 0.9% for bleeding leading to transfusion, and used this information to calculate outcome estimates. For instance, we multiplied the median VTE estimate by 0.964 for non-fatal VTE and by 0.036 for fatal VTE (if both reintervention and transfusion rates were available, we preferred reintervention estimates for calculation of fatal bleeding estimate).

† Risk factors included 1) age more than 75 years, 2) obesity (body mass index of 35 or more), 3) VTE in a first degree relative (parents, full siblings, or children), and 4) prior VTE. We assumed that patients with any combination of two or more risk factors had a risk ratio of 4. Using these risk factors, we then categorized risk of VTE as low, medium, and high risk.

‡ Options for certainty in estimates are high, moderate, low, and very low. Evidence begins as high and is rated down for serious risk of bias, inconsistency, imprecision, or indirectness. We always rated down once due to uncertainty in the patient VTE risk factors and models of timing of VTE and bleeding. For fatal VTE and fatal bleeding we always rated down once for uncertainty in our case fatality rate estimates.

119. Evidence profile 119. Subtotal gastrectomy, laparoscopic, in non-Asian countries: Absolute risk of venous thromboembolism and bleeding among patients not receiving prophylaxis

Quality assessment					Summary of findings		
**No of participants (studies)**	**Risk of Bias**	**Inconsistency**	**Indirectness**	**Imprecision**	**Best (median) estimate across all risk strata (%)***	**Best (median estimate) by patient risk strata (%)†**	**Overall certainty in estimates‡**
**Non-fatal symptomatic venous thromboembolism**							
61 (1)	Serious limitations	No serious limitations	No serious limitations	Very serious limitations	2.48	**Low: 1.69 Medium: 3.39 High: 6.78**	**Very low**
**Fatal venous thromboembolism**							
61 (1)	Serious limitations	No serious limitations	No serious limitations	Very serious limitations	0.09	**Low: 0.06 Medium: 0.13 High: 0.25**	**Very low**
**Symptomatic splanchnic vein thrombosis**							
**Non-fatal bleeding requiring reoperation**							
61 (1)	Serious limitations	No serious limitations	No serious limitations	Very serious limitations	1.09	**1.09**	**Very low**
**Non-fatal bleeding leading to transfusion**							
**Fatal bleeding**							
61 (1)	Serious limitations	No serious limitations	No serious limitations	Very serious limitations	0.04	**0.04**	**Very low**
**Bleeding leading to hemoglobin below 70g/L (7g/dL)**							

Blank spaces indicate absence of information
We did not find any studies including robotic procedures for this procedure.
* Estimate represents absolute risk in percent. Our median best estimates include fatal and non-fatal events. Based on data from included studies, we estimated case fatality rates as follows: 3.6% for VTE, 3.6% for bleeding leading to reintervention, and 0.9% for bleeding leading to transfusion, and used this information to calculate outcome estimates. For instance, we multiplied the median VTE estimate by 0.964 for non-fatal VTE and by 0.036 for fatal VTE (if both reintervention and transfusion rates were available, we preferred reintervention estimates for calculation of fatal bleeding estimate).
† Risk factors included 1) age more than 75 years, 2) obesity (body mass index of 35 or more), 3) VTE in a first degree relative (parents, full siblings, or children), and 4) prior VTE. We assumed that patients with any combination of two or more risk factors had a risk ratio of 4. Using these risk factors, we then categorized risk of VTE as low, medium, and high risk.
‡ Options for certainty in estimates are high, moderate, low, and very low. Evidence begins as high and is rated down for serious risk of bias, inconsistency, imprecision, or indirectness. We always rated down once due to uncertainty in the patient VTE risk factors and models of timing of VTE and bleeding. For fatal VTE and fatal bleeding we always rated down once for uncertainty in our case fatality rate estimates.

120. Evidence profile 120. Subtotal gastrectomy, open, in non-Asian countries: Absolute risk of venous thromboembolism and bleeding among patients not receiving prophylaxis

Quality assessment					Summary of findings		
**No of participants (studies)**	**Risk of Bias**	**Inconsistency**	**Indirectness**	**Imprecision**	**Best (median) estimate across all risk strata (%)***	**Best (median estimate) by patient risk strata (%)†**	**Overall certainty in estimates‡**
**Non-fatal symptomatic venous thromboembolism**							
1,581 (1)	No serious limitations	No serious limitations	No serious limitations	No serious limitations	2.40	**Low: 1.38 Medium: 2.75 High: 5.51**	**Moderate**
**Fatal venous thromboembolism**							
1,581 (1)	No serious limitations	No serious limitations	No serious limitations	No serious limitations	0.09	**Low: 0.05 Medium: 0.1 High: 0.21**	**Low**
**Symptomatic splanchnic vein thrombosis**							
**Non-fatal bleeding requiring reoperation**							
310 (1)	No serious limitations	No serious limitations	No serious limitations	Serious limitations	0.43	**0.43**	**Low**
**Non-fatal bleeding leading to transfusion**							
**Fatal bleeding**							
310 (1)	No serious limitations	No serious limitations	No serious limitations	Serious limitations	0.02	**0.02**	**Very Low**
**Bleeding leading to hemoglobin below 70g/L (7g/dL)**							

Blank spaces indicate absence of information

* Estimate represents absolute risk in percent. Our median best estimates include fatal and non-fatal events. Based on data from included studies, we estimated case fatality rates as follows: 3.6% for VTE, 3.6% for bleeding leading to reintervention, and 0.9% for bleeding leading to transfusion, and used this information to calculate outcome estimates. For instance, we multiplied the median VTE estimate by 0.964 for non-fatal VTE and by 0.036 for fatal VTE (if both reintervention and transfusion rates were available, we preferred reintervention estimates for calculation of fatal bleeding estimate).

† Risk factors included 1) age more than 75 years, 2) obesity (body mass index of 35 or more), 3) VTE in a first degree relative (parents, full siblings, or children), and 4) prior VTE. We assumed that patients with any combination of two or more risk factors had a risk ratio of 4. Using these risk factors, we then categorized risk of VTE as low, medium, and high risk.

‡ Options for certainty in estimates are high, moderate, low, and very low. Evidence begins as high and is rated down for serious risk of bias, inconsistency, imprecision, or indirectness. We always rated down once due to uncertainty in the patient VTE risk factors and models of timing of VTE and bleeding. For fatal VTE and fatal bleeding we always rated down once for uncertainty in our case fatality rate estimates.

121. Evidence profile 121. Total gastrectomy, open, in non-Asian countries: Absolute risk of venous thromboembolism and bleeding among patients not receiving prophylaxis

Quality assessment					Summary of findings		
**No of participants (studies)**	**Risk of Bias**	**Inconsistency**	**Indirectness**	**Imprecision**	**Best (median) estimate across all risk strata (%)***	**Best (median estimate) by patient risk strata (%)†**	**Overall certainty in estimates‡**
**Non-fatal symptomatic venous thromboembolism**							
999 (1)	No serious limitations	No serious limitations	No serious limitations	Serious limitations	4.32	**Low: 2.87 Medium: 5.74 High: 11.49**	**Low**
**Fatal venous thromboembolism**							
999 (1)	No serious limitations	No serious limitations	No serious limitations	Serious limitations	0.16	**Low: 0.11 Medium: 0.21 High: 0.43**	**Very Low**
**Symptomatic splanchnic vein thrombosis**							
**Non-fatal bleeding requiring reoperation**							
**Non-fatal bleeding leading to transfusion**							
**Fatal bleeding**							
**Bleeding leading to hemoglobin below 70g/L (7g/dL)**							

Blank spaces indicate absence of information

* Estimate represents absolute risk in percent. Our median best estimates include fatal and non-fatal events. Based on data from included studies, we estimated case fatality rates as follows: 3.6% for VTE, 3.6% for bleeding leading to reintervention, and 0.9% for bleeding leading to transfusion, and used this information to calculate outcome estimates. For instance, we multiplied the median VTE estimate by 0.964 for non-fatal VTE and by 0.036 for fatal VTE (if both reintervention and transfusion rates were available, we preferred reintervention estimates for calculation of fatal bleeding estimate).

† Risk factors included 1) age more than 75 years, 2) obesity (body mass index of 35 or more), 3) VTE in a first degree relative (parents, full siblings, or children), and 4) prior VTE. We assumed that patients with any combination of two or more risk factors had a risk ratio of 4. Using these risk factors, we then categorized risk of VTE as low, medium, and high risk.

‡ Options for certainty in estimates are high, moderate, low, and very low. Evidence begins as high and is rated down for serious risk of bias, inconsistency, imprecision, or indirectness. We always rated down once due to uncertainty in the patient VTE risk factors and models of timing of VTE and bleeding. For fatal VTE and fatal bleeding we always rated down once for uncertainty in our case fatality rate estimates.

122. Evidence profile 122. Gastric bypass, minimally-invasive: Absolute risk of venous thromboembolism and bleeding among patients not receiving prophylaxis

Quality assessment					Summary of findings		
No of participants (studies)	Risk of Bias	Inconsistency	Indirectness	Imprecision	Best (median) estimate across all risk strata (%)*	Best (median estimate) by patient risk strata (%)†	Overall certainty in estimates‡
**Non-fatal symptomatic venous thromboembolism**							
286,668 (8)	No serious limitations	No serious limitations	No serious limitations	No serious limitations	0.60	**Medium: 0.50 High: 0.99**	**Moderate**
**Fatal venous thromboembolism**							
286,668 (8)	No serious limitations	No serious limitations	No serious limitations	No serious limitations	0.02	**Medium: 0.02 High: 0.04**	**Low**
**Symptomatic splanchnic vein thrombosis**							
55 (1)	Serious limitations	No serious limitations	No serious limitations	Very serious limitations	0.00	**0.00**	**Very low**
**Non-fatal bleeding requiring reintervention**							
119,535 (6)	No serious limitations	Serious limitations	No serious limitations	No serious limitations	0.25	**0.25**	**Very low**
**Non-fatal bleeding leading to transfusion**							
109,699 (6)	No serious limitations	No serious limitations	No serious limitations	No serious limitations	0.39	**0.39**	**Moderate**
**Fatal bleeding**							
109,699 (6)	No serious limitations	No serious limitations	No serious limitations	No serious limitations	0.01	**0.01**	**Low**
**Bleeding leading to hemoglobin below 70g/L (7g/dL)**							

Blank spaces indicate absence of information
For VTE we found 2 studies with 616 patients with very low risk of bias and 6 studies with 286,052 patients with low risk of bias. For bleeding requiring reoperation we found 2 studies with 616 patients with very low risk of bias and 4 low risk of bias studies with 118,919 patients. For bleeding leading to transfusion, we found 2 studies with 616 patients with very low risk of bias and 4 low risk of bias studies with 109,083 patients. Therefore, we excluded moderate and high risk of bias studies from non-fatal and fatal symptomatic venous thromboembolism, bleeding requiring reoperation and bleeding requiring transfusion estimates.
* Estimate represents absolute risk in percent. Our median best estimates include fatal and non-fatal events. Based on data from included studies, we estimated case fatality rates as follows: 3.6% for VTE, 3.6% for bleeding leading to reintervention, and 0.9% for bleeding leading to transfusion, and used this information to calculate outcome estimates. For instance, we multiplied the median VTE estimate by 0.964 for non-fatal VTE and by 0.036 for fatal VTE (if both reintervention and transfusion rates were available, we preferred reintervention estimates for calculation of fatal bleeding estimate).
† Risk factors included 1) age more than 75 years, 2) obesity (body mass index of 35 or more), 3) VTE in a first degree relative (parents, full siblings, or children), and 4) prior VTE. We assumed that patients with any combination of two or more risk factors had a risk ratio of 4. Using these risk factors, we then categorized risk of VTE as low, medium, and high risk. For bariatric surgery, all patients are at medium or high risk of VTE (all have body mass index of 35 or more).
‡ Options for certainty in estimates are high, moderate, low, and very low. Evidence begins as high and is rated down for serious risk of bias, inconsistency, imprecision, or indirectness. We always rated down once due to uncertainty in the patient VTE risk factors and models of timing of VTE and bleeding. For fatal VTE and fatal bleeding we always rated down once for uncertainty in our case fatality rate estimates.

123. Evidence profile 123. Gastric bypass, laparoscopic: Absolute risk of venous thromboembolism and bleeding among patients not receiving prophylaxis

Quality assessment					Summary of findings		
**No of participants (studies)**	**Risk of Bias**	**Inconsistency**	**Indirectness**	**Imprecision**	**Best (median) estimate across all risk strata (%)***	**Best (median estimate) by patient risk strata (%)†**	**Overall certainty in estimates‡**
**Non-fatal symptomatic venous thromboembolism**							
280,751 (7)	No serious limitations	No serious limitations	No serious limitations	No serious limitations	0.49	**Medium: 0.41 High: 0.82**	**Moderate**
**Fatal venous thromboembolism**							
280,751 (7)	No serious limitations	No serious limitations	No serious limitations	No serious limitations	0.02	**Medium: 0.02 High: 0.03**	**Low**
**Symptomatic splanchnic vein thrombosis**							
55 (1)	Serious limitations	No serious limitations	No serious limitations	Very serious limitations	0.00	**0.00**	**Very low**
**Non-fatal bleeding requiring reintervention**							
119,435 (6)	No serious limitations	Serious limitations	No serious limitations	No serious limitations	0.25	**0.25**	**Low**
**Non-fatal bleeding leading to transfusion**							
103,882 (5)	No serious limitations	No serious limitations	No serious limitations	No serious limitations	0.51	**0.51**	**Moderate**
**Fatal bleeding**							
119,435 (6)	No serious limitations	Serious limitations	No serious limitations	No serious limitations	0.01	**0.01**	**Very Low**
**Bleeding leading to hemoglobin below 70g/L (7g/dL)**							

Blank spaces indicate absence of information

We excluded moderate and high risk of bias studies from symptomatic non-fatal and fatal venous thromboembolism, bleeding requiring reoperation and bleeding requiring transfusion estimates

* Estimate represents absolute risk in percent. Our median best estimates include fatal and non-fatal events. Based on data from included studies, we estimated case fatality rates as follows: 3.6% for VTE, 3.6% for bleeding leading to reintervention, and 0.9% for bleeding leading to transfusion, and used this information to calculate outcome estimates. For instance, we multiplied the median VTE estimate by 0.964 for non-fatal VTE and by 0.036 for fatal VTE (if both reintervention and transfusion rates were available, we preferred reintervention estimates for calculation of fatal bleeding estimate).

† Risk factors included 1) age more than 75 years, 2) obesity (body mass index of 35 or more), 3) VTE in a first degree relative (parents, full siblings, or children), and 4) prior VTE. We assumed that patients with any combination of two or more risk factors had a risk ratio of 4. Using these risk factors, we then categorized risk of VTE as low, medium, and high risk. For bariatric surgery, all patients are at medium or high risk of VTE (all have body mass index of 35 or more).

‡ Options for certainty in estimates are high, moderate, low, and very low. Evidence begins as high and is rated down for serious risk of bias, inconsistency, imprecision, or indirectness. We always rated down once due to uncertainty in the patient VTE risk factors and models of timing of VTE and bleeding. For fatal VTE and fatal bleeding we always rated down once for uncertainty in our case fatality rate estimates.

124. Evidence profile 124. Gastric bypass, robotic: Absolute risk of venous thromboembolism and bleeding among patients not receiving prophylaxis

Quality assessment					Summary of findings		
**No of participants (studies)**	**Risk of Bias**	**Inconsistency**	**Indirectness**	**Imprecision**	**Best (median) estimate across all risk strata (%)***	**Best (median estimate) by patient risk strata (%)†**	**Overall certainty in estimates‡**
**Non-fatal symptomatic venous thromboembolism**							
7,453 (7)	No serious limitations	Serious limitations	No serious limitations	No serious limitations	1.48	**Medium: 1.23 High: 2.45**	**Low**
**Fatal venous thromboembolism**							
7,453 (7)	No serious limitations	Serious limitations	No serious limitations	No serious limitations	0.06	**Medium: 0.05 High: 0.09**	**Very Low**
**Symptomatic splanchnic vein thrombosis**							
**Non-fatal bleeding requiring reintervention**							
436 (4)	Serious limitations	No serious limitations	No serious limitations	Serious limitations	0.33¶	**0.33¶**	**Very low**
**Non-fatal bleeding leading to transfusion**							
6,063 (3)	No serious limitations	No serious limitations	No serious limitations	No serious limitations	0.47	**0.47**	**Moderate**
**Fatal bleeding**							
6,063 (3)	No serious limitations	No serious limitations	No serious limitations	No serious limitations	0.01	**0.01**	**Low**
**Bleeding leading to hemoglobin below 70g/L (7g/dL)**							

Blank spaces indicate absence of information

* Estimate represents absolute risk in percent. Our median best estimates include fatal and non-fatal events. Based on data from included studies, we estimated case fatality rates as follows: 3.6% for VTE, 3.6% for bleeding leading to reintervention, and 0.9% for bleeding leading to transfusion, and used this information to calculate outcome estimates. For instance, we multiplied the median VTE estimate by 0.964 for non-fatal VTE and by 0.036 for fatal VTE (if both reintervention and transfusion rates were available, we preferred reintervention estimates for calculation of fatal bleeding estimate).

† Risk factors included 1) age more than 75 years, 2) obesity (body mass index of 35 or more), 3) VTE in a first degree relative (parents, full siblings, or children), and 4) prior VTE. We assumed that patients with any combination of two or more risk factors had a risk ratio of 4. Using these risk factors, we then categorized risk of VTE as low, medium, and high risk. For bariatric surgery, all patients are at medium or high risk of VTE (all have body mass index of 35 or more).

‡ Options for certainty in estimates are high, moderate, low, and very low. Evidence begins as high and is rated down for serious risk of bias, inconsistency, imprecision, or indirectness. We always rated down once due to uncertainty in the patient VTE risk factors and models of timing of VTE and bleeding. For fatal VTE and fatal bleeding we always rated down once for uncertainty in our case fatality rate estimates.

¶Reported median estimate in eligible studies for this outcome was 0.0%. As real underlying risk of 0.0% is improbable we used average instead of median.

125. Evidence profile 125. Gastric bypass, open: Absolute risk of venous thromboembolism and bleeding among patients not receiving prophylaxis

Quality assessment					Summary of findings		
**No of participants (studies)**	**Risk of Bias**	**Inconsistency**	**Indirectness**	**Imprecision**	**Best (median) estimate across all risk strata (%)***	**Best (median estimate) by patient risk strata (%)†**	**Overall certainty in estimates‡**
**Non-fatal symptomatic venous thromboembolism**							
68,017 (18)	Serious limitations	Serious limitations	No serious limitations	No serious limitations	1.31	**Medium: 1.09 High: 2.17**	**Very low**
**Fatal venous thromboembolism**							
68,017 (18)	Serious limitations	Serious limitations	No serious limitations	No serious limitations	0.05	**Medium: 0.04 High: 0.08**	**Very low**
**Symptomatic splanchnic vein thrombosis**							
**Non-fatal bleeding requiring reintervention**							
3,256 (4)	No serious limitations	No serious limitations	No serious limitations	No serious limitations	0.18	**0.18**	**Moderate**
**Non-fatal bleeding leading to transfusion**							
2,906 (5)	No serious limitations	No serious limitations	No serious limitations	No serious limitations	0.82	**0.82**	**Moderate**
**Fatal bleeding**							
3,256 (4)	No serious limitations	No serious limitations	No serious limitations	No serious limitations	0.01	**0.01**	**Low**
**Bleeding leading to hemoglobin below 70g/L (7g/dL)**							

Blank spaces indicate absence of information
* Estimate represents absolute risk in percent. Our median best estimates include fatal and non-fatal events. Based on data from included studies, we estimated case fatality rates as follows: 3.6% for VTE, 3.6% for bleeding leading to reintervention, and 0.9% for bleeding leading to transfusion, and used this information to calculate outcome estimates. For instance, we multiplied the median VTE estimate by 0.964 for non-fatal VTE and by 0.036 for fatal VTE (if both reintervention and transfusion rates were available, we preferred reintervention estimates for calculation of fatal bleeding estimate).
† Risk factors included 1) age more than 75 years, 2) obesity (body mass index of 35 or more), 3) VTE in a first degree relative (parents, full siblings, or children), and 4) prior VTE. We assumed that patients with any combination of two or more risk factors had a risk ratio of 4. Using these risk factors, we then categorized risk of VTE as low, medium, and high risk. For bariatric surgery, all patients are at medium or high risk of VTE (all have body mass index of 35 or more).
‡ Options for certainty in estimates are high, moderate, low, and very low. Evidence begins as high and is rated down for serious risk of bias, inconsistency, imprecision, or indirectness. We always rated down once due to uncertainty in the patient VTE risk factors and models of timing of VTE and bleeding. For fatal VTE and fatal bleeding we always rated down once for uncertainty in our case fatality rate estimates.

126. Evidence profile 126. Sleeve gastrectomy, minimally-invasive: Absolute risk of venous thromboembolism and bleeding among patients not receiving prophylaxis

Quality assessment					Summary of findings		
**No of participants (studies)**	**Risk of Bias**	**Inconsistency**	**Indirectness**	**Imprecision**	**Best (median) estimate across all risk strata (%)***	**Best (median estimate) by patient risk strata (%)†**	**Overall certainty in estimates‡**
**Non-fatal symptomatic venous thromboembolism**							
470,221 (14)	No serious limitations	No serious limitations	No serious limitations	No serious limitations	0.26	**Medium: 0.22 High: 0.44**	**Moderate**
**Fatal venous thromboembolism**							
470,221 (14)	No serious limitations	No serious limitations	No serious limitations	No serious limitations	0.01	**Medium: 0.01 High: 0.02**	**Low**
**Symptomatic splanchnic vein thrombosis§**							
6,042 (9)	Serious limitations	Serious limitations	No serious limitations	No serious limitations	0.26	**0.26**	**Very low**
**Non-fatal bleeding requiring reintervention**							
316,048 (7)	No serious limitations	No serious limitations	No serious limitations	No serious limitations	0.26¶	**0.26¶**	**Moderate**
**Non-fatal bleeding leading to transfusion**							
331,729 (8)	No serious limitations	Serious limitations	No serious limitations	No serious limitations	0.37	**0.37**	**Low**
**Fatal bleeding**							
316,048 (7)	No serious limitations	No serious limitations	No serious limitations	No serious limitations	0.01	**0.01**	**Low**
**Bleeding leading to hemoglobin below 70g/L (7g/dL)**							

Blank spaces indicate absence of information

For VTE we found 4 studies with 1061 patients with very low risk of bias and 10 studies with 469,160 patients with low risk of bias. For bleeding requiring reoperation we found 3 studies with 534 patients with very low risk of bias and 4 low risk of bias studies with 315,514 patients. For bleeding leading to transfusion, we found 3 studies with 759 patients with very low risk of bias and 5 low risk of bias studies with 330,970 patients. Therefore, we excluded moderate and high risk of bias studies from symptomatic non-fatal and fatal venous thromboembolism, bleeding requiring reoperation and bleeding requiring transfusion estimates.

* Estimate represents absolute risk in percent. Our median best estimates include fatal and non-fatal events. Based on data from included studies, we estimated case fatality rates as follows: 3.6% for VTE, 3.6% for bleeding leading to reintervention, and 0.9% for bleeding leading to transfusion, and used this information to calculate outcome estimates. For instance, we multiplied the median VTE estimate by 0.964 for non-fatal VTE and by 0.036 for fatal VTE (if both reintervention and transfusion rates were available, we preferred reintervention estimates for calculation of fatal bleeding estimate).

† Risk factors included 1) age more than 75 years, 2) obesity (body mass index of 35 or more), 3) VTE in a first degree relative (parents, full siblings, or children), and 4) prior VTE. We assumed that patients with any combination of two or more risk factors had a risk ratio of 4. Using these risk factors, we then categorized risk of VTE as low, medium, and high risk. For bariatric surgery, all patients are at medium or high risk of VTE (all have body mass index of 35 or more).

‡ Options for certainty in estimates are high, moderate, low, and very low. Evidence begins as high and is rated down for serious risk of bias, inconsistency, imprecision, or indirectness. We always rated down once due to uncertainty in the patient VTE risk factors and models of timing of VTE and bleeding. For fatal VTE and fatal bleeding we always rated down once for uncertainty in our case fatality rate estimates.

§ The best median estimate for symptomatic splanchnic vein thrombosis is median value of reported estimates. As we did not find evidence for timing of SVT, effect of thromboprophylaxis on SVT or patient risk factors for SVT, we did not model splanchnic vein thrombosis estimates for these factors.¶Reported median estimate in eligible studies for this procedure was 0,0%. As a real underlying risk of 0,0% is improbable we used average instead of median.

127. Evidence profile 127. Sleeve gastrectomy, laparoscopic: Absolute risk of venous thromboembolism and bleeding among patients not receiving prophylaxis

Quality assessment					Summary of findings		
**No of participants (studies)**	**Risk of Bias**	**Inconsistency**	**Indirectness**	**Imprecision**	**Best (median) estimate across all risk strata (%)***	**Best (median estimate) by patient risk strata (%)†**	**Overall certainty in estimates‡**
**Non-fatal symptomatic venous thromboembolism**							
457,309 (13)	No serious limitations	No serious limitations	No serious limitations	No serious limitations	0.24	**Medium: 0.20 High: 0.40**	**Moderate**
**Fatal venous thromboembolism**							
457,309 (13)	No serious limitations	No serious limitations	No serious limitations	No serious limitations	0.01	**Medium: 0.01 High: 0.01**	**Low**
**Symptomatic splanchnic vein thrombosis§**							
5,168 (7)	Serious limitations	Serious limitations	No serious limitations	No serious limitations	0.13	**0.13**	**Very low**
**Non-fatal bleeding requiring reintervention**							
316,048 (7)	No serious limitations	No serious limitations	No serious limitations	No serious limitations	0.26¶	**0.26¶**	**Moderate**
**Non-fatal bleeding leading to transfusion**							
318,817 (7)	No serious limitations	Serious limitations	No serious limitations	No serious limitations	0.49	**0.49**	**Low**
**Fatal bleeding**							
316,048 (7)	No serious limitations	No serious limitations	No serious limitations	No serious limitations	0.01	**0.01**	**Low**
**Bleeding leading to hemoglobin below 70g/L (7g/dL)**							

Blank spaces indicate absence of information
We excluded moderate and high risk of bias studies from symptomatic non-fatal and fatal venous thromboembolism, bleeding requiring reoperation and bleeding requiring transfusion estimates.
* Estimate represents absolute risk in percent. Our median best estimates include fatal and non-fatal events. Based on data from included studies, we estimated case fatality rates as follows: 3.6% for VTE, 3.6% for bleeding leading to reintervention, and 0.9% for bleeding leading to transfusion, and used this information to calculate outcome estimates. For instance, we multiplied the median VTE estimate by 0.964 for non-fatal VTE and by 0.036 for fatal VTE (if both reintervention and transfusion rates were available, we preferred reintervention estimates for calculation of fatal bleeding estimate).
† Risk factors included 1) age more than 75 years, 2) obesity (body mass index of 35 or more), 3) VTE in a first degree relative (parents, full siblings, or children), and 4) prior VTE. We assumed that patients with any combination of two or more risk factors had a risk ratio of 4. Using these risk factors, we then categorized risk of VTE as low, medium, and high risk. For bariatric surgery, all patients are at medium or high risk of VTE (all have body mass index of 35 or more).
‡ Options for certainty in estimates are high, moderate, low, and very low. Evidence begins as high and is rated down for serious risk of bias, inconsistency, imprecision, or indirectness. We always rated down once due to uncertainty in the patient VTE risk factors and models of timing of VTE and bleeding. For fatal VTE and fatal bleeding we always rated down once for uncertainty in our case fatality rate estimates.
§ The best median estimate for symptomatic splanchnic vein thrombosis is median value of reported estimates. As we did not find evidence for timing of SVT, effect of thromboprophylaxis on SVT or patient risk factors for SVT, we did not model splanchnic vein thrombosis estimates for these factors.
¶Reported median estimate in eligible studies for this procedure was 0,0%. As a real underlying risk of 0,0% is improbable we used average instead of median.

128. Evidence profile 128. Sleeve gastrectomy, robotic: Absolute risk of venous thromboembolism and bleeding among patients not receiving prophylaxis

Quality assessment					Summary of findings		
**No of participants (studies)**	**Risk of Bias**	**Inconsistency**	**Indirectness**	**Imprecision**	**Best (median) estimate across all risk strata (%)***	**Best (median estimate) by patient risk strata (%)†**	**Overall certainty in estimates‡**
**Non-fatal symptomatic venous thromboembolism**							
13,457 (3)	No serious limitations	No serious limitations	No serious limitations	No serious limitations	0.93	**Medium: 0.77 High: 1.55**	**Moderate**
**Fatal venous thromboembolism**							
13,457 (3)	No serious limitations	No serious limitations	No serious limitations	No serious limitations	0.03	**Medium: 0.03 High: 0.06**	**Low**
**Symptomatic splanchnic vein thrombosis§**							
874 (2)	Serious limitations	No serious limitations	No serious limitations	Serious limitations	0.64	**0.64**	**Very low**
**Non-fatal bleeding requiring reintervention**							
545 (2)	No serious limitations	No serious limitations	No serious limitations	Serious limitations	0.44	**0.44**	**Low**
**Non-fatal bleeding leading to transfusion**							
13,323 (2)	No serious limitations	No serious limitations	No serious limitations	No serious limitations	1.02	**1.02**	**Moderate**
**Fatal bleeding**							
545 (2)	No serious limitations	No serious limitations	No serious limitations	Serious limitations	0.02	**0.02**	**Very Low**
**Bleeding leading to hemoglobin below 70g/L (7g/dL)**							

Blank spaces indicate absence of information

* Estimate represents absolute risk in percent. Our median best estimates include fatal and non-fatal events. Based on data from included studies, we estimated case fatality rates as follows: 3.6% for VTE, 3.6% for bleeding leading to reintervention, and 0.9% for bleeding leading to transfusion, and used this information to calculate outcome estimates. For instance, we multiplied the median VTE estimate by 0.964 for non-fatal VTE and by 0.036 for fatal VTE (if both reintervention and transfusion rates were available, we preferred reintervention estimates for calculation of fatal bleeding estimate).

† Risk factors included 1) age more than 75 years, 2) obesity (body mass index of 35 or more), 3) VTE in a first degree relative (parents, full siblings, or children), and 4) prior VTE. We assumed that patients with any combination of two or more risk factors had a risk ratio of 4. Using these risk factors, we then categorized risk of VTE as low, medium, and high risk. For bariatric surgery, all patients are at medium or high risk of VTE (all have body mass index of 35 or more).

‡ Options for certainty in estimates are high, moderate, low, and very low. Evidence begins as high and is rated down for serious risk of bias, inconsistency, imprecision, or indirectness. We always rated down once due to uncertainty in the patient VTE risk factors and models of timing of VTE and bleeding. For fatal VTE and fatal bleeding we always rated down once for uncertainty in our case fatality rate estimates.

§ The best median estimate for symptomatic splanchnic vein thrombosis is median value of reported estimates. As we did not find evidence for timing of SVT, effect of thromboprophylaxis on SVT or patient risk factors for SVT, we did not model splanchnic vein thrombosis estimates for these factors.

4. General abdominal surgery supplementary tables 1-6

1. Characteristics of individual studies in general abdominal surgery

Reference	Year	Country/ Countries	Patients (n)	Age Mean (SD)*	Female (%)	Malignantancy (%)	Length of stay (Days)	Recruitment First year	Recruitment Last year	Study type
**Appendectomy, laparoscopic**										
Nguyen	2007	USA	24509		61		2	2002	2006	Multicenter in one country
Hemmila	2010	USA	15445	38 (16)	48		1†	2005	2007	Multicenter in one country
Brugger	2011	Switzerland	7446	31†	56		4	1995	2006	Multicenter in one country
Alizadeh	2017	USA	168963	48 (17)	62			2005	2014	Multicenter in one country
Chung	2019	Taiwan	52767	43 (17)	49			2000	2012	Multicenter in one country
Garcia	2019	USA	83712	44 (18)	48			2012	2014	Multicenter in one country
**Appendectomy, open**										
Nguyen	2007	USA	25554		61		3	2002	2006	Multicenter in one country
Hemmila	2010	USA	6030	41 (17)	42	0	2†	2005	2007	Multicenter in one country
Chung	2019	Taiwan	193845	43 (17)	49			2000	2012	Multicenter in one country
Garcia	2019	USA	12665	44 (18)	48			2012	2014	Multicenter in one country
**Appendectomy, laparoscopic, emergency**										
Brugger	2011	Switzerland	7446	31†	56		4	1995	2006	Multicenter in one country
Sakran	2019	USA	65017	50 (17)	51			2013	2015	Multicenter in one country
**Appendectomy, open, emergency**										
Sakran	2019	USA	6292	50 (17)	51			2013	2015	Multicenter in one country
**Cholecystectomy, conversion to open**										

Persson§	2012	Sweden	3768	50†	68	0		2005	2010	Multicenter in one country
**Cholecystectomy, laparoscopic**										
Blake	2001	USA	587	45 (20)	79		2	1996	2000	One center, multiple surgeons
Schaepkens Van Riempst§	2002	Belgium	238	55	71		5	1995	1999	Multicenter in one country
Engbaek	2006	Denmark	258					1996	2000	Multicenter in one country
Lindberg§	2006	Sweden	50	51 (9)	62			1999	2001	One center, multiple surgeons
Nguyen	2007	USA	50527		61		3	2002	2006	Multicenter in one country
Rathore	2007	UK	164	48†	80		0	2002	2004	One center, multiple surgeons
Triantafyllidis	2009	Greece	1009	48 (16)	77	0	17	2000	2008	One center, multiple surgeons
Ingraham	2010	USA	58659	48 (26)†	73		2	2005	2008	Multicenter in one country
Ntourakis	2011	Greece	119	58 (15)	59	0		2005	2006	One center, multiple surgeons
Hasbahceci§	2012	Turkey	1557	54 (12)	78		1†	2000	2010	One center, multiple surgeons
Pakaneh	2012	Iran	100	49 (4)	90			not specified	not specified	One center, multiple surgeons
Persson§	2012	Sweden	42271	50†	68	0		2005	2010	Multicenter in one country
Stein	2014	USA	4107430	52 (20)	70		6†	1998	2009	Multicenter in one country
Suuronen	2015	Finland	17175	52 (15)	73	0	3	2002	2007	Multicenter in one country
Donkervoort	2016	Netherlands	4359	50 (23)†	54			2002	2012	Multicenter in one country
Ulrych	2016	Czech Republic	90	53 (23)†		0	3†	2011	2012	One center, multiple surgeons
Gundogdu	2017	Turkey	1485	49†	75			2005	2015	One center, multiple surgeons
Rosero	2017	USA	230745		75	0	3†	2009	2011	Multicenter in one country
Sepassi	2018	USA	518				4†	2014	2015	Multicenter in one country
Coelho	2019	Brazil	1645	50 (15)	67	0		2011	2018	One center, multiple surgeons
Rysmakhanov	2019	Kazakhstan	1658	52 (9)	75			2010	2019	One center, multiple surgeons
Ross	2020	USA	256726	55 (17)	44		3	2005	2016	Multicenter in one country
**Cholecystectomy, laparoscopic, elective**										

Schaepkens Van Riempst§	2002	Belgium	238	55	71		4	1995	1999	Multicenter in one country
Rathore	2007	UK	164	48†	80		0	2002	2004	One center, multiple surgeons
Ntourakis	2011	Greece	119	58 (15)	59	0		2005	2006	One center, multiple surgeons
Ulrych	2016	Czech Republic	90	53 (23)†		0	3†	2011	2012	One center, multiple surgeons
Gundogdu	2017	Turkey	1485	49†	75			2005	2015	One center, multiple surgeons
Sepassi	2018	USA	518				4†	2014	2015	Multicenter in one country

Cholecystectomy, laparoscopic, emergency

Sakran	2019	USA	11266	50 (17)	51			2013	2015	Multicenter in one country

Cholecystectomy, open

Nguyen	2007	USA	14513		61		7	2002	2006	Multicenter in one country
Ingraham	2010	USA	6852	61 (24)†	50		6†	2005	2008	Multicenter in one country
Persson§	2012	Sweden	4370	50†	68	0		2005	2010	Multicenter in one country
Suuronen	2015	Finland	4942	63 (15)	51	0	8	2002	2007	Multicenter in one country
Sakran	2019	USA	1447	50 (17)	51			2013	2015	Multicenter in one country
Ross	2020	USA	37311	55 (17)	44			2005	2016	Multicenter in one country

Cholecystectomy, open, emergency

Sakran	2019	USA	1447	50 (17)	51			2013	2015	Multicenter in one country

Hernia repair, groin, laparoscopic

Al-Sahaf	2008	Ireland	108	55†	1			2001	2005	Single surgeon series
Srsen	2008	Croatia	82	60 (14)	2		2	2006	2006	One center, multiple surgeons
Meyer	2013	France, Japan, Spain, Brazil	4565	55 (15)	15			2001	2011	Multinational
Wakasugi	2016	Japan	365	67 (9)	11			2012	2015	One center, multiple surgeons
Wakasugi	2017	Japan	350	67 (12)	12			2012	2015	One center, multiple surgeons
Mita	2020	Japan	413	66 (1)	9			2013	2017	One center, multiple surgeons
Perez	2020	USA	5282	66 (21)†	17		3†	2009	2015	Multicenter in one country
Wang	2020	China	7110	61 (17)	11			2017	2017	Multicenter in one country
Yang§	2019	China	144	64 (16)	0			2016	2018	One center, multiple surgeons

**Hernia repair, groin, open**										
Holzheimer	2007	Germany	300	51†	27	0				One center, multiple surgeons
Srsen	2008	Croatia	134	60 (14)	2		2	2006	2006	One center, multiple surgeons
Bessa	2015	Egypt	234	56 (18)	9	0	3	2003	2013	One center, multiple surgeons
Lozano	2015	Spain	218	49 (9)	19			2007	2008	One center, multiple surgeons
Nilsson	2016	Sweden	140567	60 (15)	8			2002	2011	Multicenter in one country
Tastaldi	2019	USA	257	72 (23)†	38		3†	2005	2015	One center, multiple surgeons
Liu	2020	China	146	75†	13		5†	2013	2016	One center, multiple surgeons
Perez	2020	USA	36575	69 (25)†	19		2†	2009	2015	Multicenter in one country
Poudel§	2020	Japan	4870	59 (15)	17			2008	2019	Single surgeon series
Wang	2020	China	6776	61 (17)	11			2017	2017	Multicenter in one country
**Hernia repair, groin, minimally-invasive, elective**										
Srsen	2008	Croatia	82	60 (14)	2		2	2006	2006	One center, multiple surgeons
Meyer	2013	France, Japan, Spain, Brazil	4565	55 (15)	15			2001	2011	Multinational
Mita	2020	Japan	413	66 (1)	9			2013	2017	One center, multiple surgeons
Yang§	2019	China	144	64 (16)	0			2016	2018	One center, multiple surgeons
**Hernia repair, groin, open, elective**										
Srsen	2008	Croatia	134	60 (14)	2		2	2006	2006	One center, multiple surgeons
Lozano	2015	Spain	218	49 (9)	19			2007	2008	One center, multiple surgeons
Nilsson	2016	Sweden	132801	60 (15)	7			2002	2011	Multicenter in one country
**Hernia repair, groin, open, emergency**										
Bessa	2015	Egypt	234	56 (18)	9	0	3	2003	2013	One center, multiple surgeons
Nilsson	2016	Sweden	7766	70 (17)	24			2002	2011	Multicenter in one country
Tastaldi	2019	USA	257	72 (23)†	38		3†	2005	2015	One center, multiple surgeons
Liu	2020	China	146	75†	13		5†	2013	2016	One center, multiple surgeons
**Hernia repair, ventral, laparoscopic**										
Lomanto	2006	Singapore	50	56 (11)	84		3	2000	2004	One center, multiple surgeons
Ferrari	2008	Italy	100	64 (16)	56		5	2002	2007	One center, multiple surgeons
Sharma	2011	India	1242	46 (18)	63		2	1992	2005	One center, multiple surgeons

Aher	2015	USA	26286	55 (14)	54	1		2009	2012	Multicenter in one country
Warren	2017	USA	103	60 (13)	73		2†	2013	2015	One center, multiple surgeons
Boules	2018	USA	361	57 (13)		1		1995	2014	One center, multiple surgeons
Ross	2020	USA	33630	55 (17)	44			2005	2016	Multicenter in one country
Zolin	2020	USA	81	55 (20)†	65	0	1†	2013	2016	One center, multiple surgeons

Hernia repair, ventral, open

Schmidbauer	2005	Germany	175	58 (14)	44			1996	2001	One center, multiple surgeons
Aher	2015	USA	90721	54 (15)	47	1		2009	2012	Multicenter in one country
Basta	2016	USA	142		49		7	2007	2014	Single surgeon series
Ulrych	2016	Czech Republic	126	58 (25)†		0	3†	2011	2012	One center, multiple surgeons
Bittner	2018	USA	76	55 (14)	54		6†	2015	2016	One center, multiple surgeons
Kraft	2019	USA	175	55 (16)	57		7	2013	2018	Single surgeon series
Ross	2020	USA	128513	55 (17)	44			2005	2016	Multicenter in one country
Zolin	2020	USA	105	57 (18)†	53	0	3†	2013	2016	One center, multiple surgeons

Hernia repair, ventral, robotic

Warren	2017	USA	53	53 (12)	58		1†	2013	2015	One center, multiple surgeons

Hernia repair, ventral, laparoscopic, elective

Lomanto	2006	Singapore	50	56 (11)	84		3	2000	2004	One center, multiple surgeons
Aher	2015	USA	26286	55 (14)	54	1		2009	2012	Multicenter in one country
Boules	2018	USA	361	57 (13)		1		1995	2014	One center, multiple surgeons
Zolin	2020	USA	81	55 (20)†	65	0	1†	2013	2016	One center, multiple surgeons

Hernia repair, ventral, laparoscopic, emergency

Sakran	2019	USA	405	50 (17)	51			2013	2015	Multicenter in one country

Hernia repair, ventral, open, elective

Aher	2015	USA	90721	54 (15)	47	1		2009	2012	Multicenter in one country
Ulrych	2016	Czech Republic	126	58 (25)†		0	3†	2011	2012	One center, multiple surgeons
Bittner	2018	USA	76	55 (14)	54		6†	2015	2016	One center, multiple surgeons
Kraft	2019	USA	175	55 (16)	57		7	2013	2018	Single surgeon series
Zolin	2020	USA	105	57 (18)†	53	0	3†	2013	2016	One center, multiple surgeons
**Hernia repair, ventral, open, emergency**										
Sakran	2019	USA	4808	50 (17)	51			2013	2015	Multicenter in one country
**Small bowel resection, laparoscopic**										
Daly	2014	USA	1780	58	56	4		2007	2011	Multicenter in one country
McKenna§	2018	USA	1415	55 (21)†	48	37		2005	2016	Multicenter in one country
**Small bowel resection, open**										
Daly	2014	USA	17701	63	53	7		2007	2011	Multicenter in one country
McKenna§	2018	USA	3592	57 (20)†	48	50		2005	2016	Multicenter in one country
Sakran	2019	USA	6855	50 (17)	51			2013	2015	Multicenter in one country
**Small bowel resection, laparoscopic, malignant**										
McKenna§	2018	USA	499	66 (19)†	48	100		2005	2016	Multicenter in one country
**Small bowel resection, laparoscopic, IBD**										
McKenna§	2018	USA	443	37 (23)†	50	0		2005	2016	Multicenter in one country
**Small bowel resection, laparoscopic, benign**										
McKenna§	2018	USA	355	59 (20)†	43	0		2005	2016	Multicenter in one country
**Small bowel resection, laparoscopic, emergency**										
McKenna§	2018	USA	118	64 (28)†	47	25		2005	2016	Multicenter in one country
**Small bowel resection, open, IBD**										
McKenna§	2018	USA	1237	43 (22)†	51	0		2005	2016	Multicenter in one country
**Small bowel resection, open, benign**										
McKenna§	2018	USA	571	67 (21)†	52	0		2005	2016	Multicenter in one country

**Small bowel resection, open, malignant**										
McKenna§	2018	USA	1784	63 (18)†	44	100		2005	2016	Multicenter in one country
**Small bowel resection, open, emergency**										
Sakran	2019	USA	6855	50 (17)	51			2013	2015	Multicenter in one country
**Splenectomy, elective, laparoscopic**										
Delaitre	2002	France	209	41 (18)	66	0	6	1991	1998	Multicenter in one country
Patel	2003	UK	108	41†	47	35	3†	1992	2000	One center, multiple surgeons
Romano	2006	Italy	72	46 (20)	53	55	3	1997	2004	One center, multiple surgeons
Casaccia	2010	Italy	676	42 (20)	51	33	5	1993	2007	Multicenter in one country
Vecchio	2011	Italy	107		55	9		1998	2011	One center, multiple surgeons
Corcione	2012	Italy	300	37 (20)	67	6	5	1992	2010	One center, multiple surgeons
Wang	2013	China	260	39 (15)	64	0	7	2003	2012	Single surgeon series
Radkowiak	2018	Poland	500	46 (31)†	63	27	4†	1998	2017	One center, multiple surgeons
Tsamalaidze	2018	USA	101	58 (16)	51			1995	2016	One center, multiple surgeons
Zychowicz	2018	Poland	194	40 (17)	38	0		1998	2017	One center, multiple surgeons
Tastaldi	2019	USA	109	48 (21)	61	0	2†	2002	2016	Single surgeon series
Hernandez	2020	USA	4365	56†	55			2008	2018	Multicenter in one country
**Splenectomy, elective, open**										
Mesa	2006	USA	314	65†	46	100	9†	1976	2004	One center, multiple surgeons
Romano	2006	Italy	86	46 (20)	53	55	6	1997	2004	One center, multiple surgeons
Zhang	2012	China	69	37 (11)		0		2007	2010	One center, multiple surgeons
Jiang	2014	China	71	52 (10)	42		15	2010	2013	One center, multiple surgeons
Li	2017	China	56	48 (16)	70			1997	2014	One center, multiple surgeons
Tsamalaidze	2018	USA	86	58 (16)	51			1995	2016	One center, multiple surgeons

Hernandez	2020	USA	2220	56†	55			2008	2018	Multicenter in one country
**Splenectomy, elective, laparoscopic, benign**										
Delaitre	2002	France	209	41 (18)	66	0	6	1991	1998	Multicenter in one country
Wang	2013	China	260	39 (15)	64	0		2003	2012	Single surgeon series
Zychowicz	2018	Poland	194	40 (17)	38	0		1998	2017	One center, multiple surgeons
Tastaldi	2019	USA	109	48 (21)	61	0	2†	2002	2016	Single surgeon series
**Splenectomy, elective, open, benign**										
Zhang	2012	China	69	37 (11)		0		2007	2010	One center, multiple surgeons
Jiang	2014	China	71	52 (10)	42		15	2010	2013	One center, multiple surgeons
Li	2017	China	56	48 (16)	70			1997	2014	One center, multiple surgeons
**Splenectomy, elective, open, malignant**										
Mesa	2006	USA	314	65†	46	100	9†	1976	2004	One center, multiple surgeons

Blank spaces indicate an absence of information.

Articles are reported by procedure, so duplicate information from same study appears in this table.

Many articles reported on more than one procedure (e.g. Nguyen 2007 provided information for laparoscopic appendectomy, open appendectomy, laparoscopic cholecystectomy and open cholecystectomy).

*Age is reported as mean (SD) unless otherwise indicated

† Median (IQR)

§ Authors confirmed accuracy of our consensus data extraction and/or corrected some errors or provided additional information

Nguyen 2007: Laparoscopic and open appendectomy, laparoscopic and open cholecystectomy: Proportion of females was provided for appendectomy and cholecystectomy combined
Srsen 2008: Laparoscopic and open groin hernia repair: Age and proportion of females was provided for laparoscopic and open groin hernia combined
Persson 2012: Laparoscopic, open and conversion to open cholecystectomy: Age and proportion of females was provided for procedures combined, for female and male population separately.
Romano 2006: Laparoscopic and open splenectomy: Age, proportion of females and proportion of patients with cancer was provided for laparoscopic and open splenectomy procedures combined.
Alizadeh 2017: Appendectomy and cholecystectomy: Age and proportion of females was provided for appendectomy and cholecystectomy combined.
Chung 2019: Laparoscopic and open appendectomy: Age and proportion of females was provided for laparoscopic and open appendectomy combined.
Garcia 2019: laparoscopic and open appendectomy: Age and proportion of females was provided for appendectomies combined for patients groups: with no cirrhosis, compensated cirrhosis and decompensated cirrhosis.
Hernandez 2020: laparoscopic and open splenectomy: Age and proportion of females was provided for laparoscopic and open splenectomies combined.
Ross 2020: Laparoscopic and open cholecystectomy, laparoscopic and open ventral hernia, laparoscopic and open colectomy: Age and proportion of females was provided for procedures combined to two groups: elective and emergency.
Sakran 2019: Appendectomy, cholecystectomy, ventral hernia repair, small bowel resection: Age and proportion of females was provided for procedures combined to two groups by duration: <100min procedure and >100min procedure
Tsamalaidze 2018: Laparoscopic and open splenectomy: Age and proportion of females was provided for procedures combined.
Wang 2020: Laparoscopic and open groin hernia: Age and proportion of females was provided for procedures combined.

Studies that were excluded from some procedures but not from others:

23868 Sakran 2019:

- Included only laparoscopic emergency appendectomy estimate and not to total laparoscopic appendectomy estimate because of overlapping population
- only to open emergency open appendectomy estimate and not to total open appendectomy estimate (overlapping population)
- only to emergency laparoscopic cholecystectomy estimate and not to total laparoscopic cholecystectomy estimate (overlapping population)
- only to emergency laparoscopic ventral hernia repair estimate and not to total laparoscopic ventral hernia repair estimate (overlapping population)
- only to emergency open ventral hernia repair estimate and not to total open ventral hernia repair estimate (overlapping population)

Aher 2015: We excluded the study from VTE estimate for laparoscopic and open ventral hernia (because of overlapping population) but included the study to elective laparoscopic and open ventral hernia VTE estimate. We also included the study to bleeding leading to transfusion estimates for laparoscopic and open ventral hernia repair.

Studies where some outcomes were excluded for a procedure (but not all outcomes):

Ingraham 2010: Laparoscopic cholecystectomy: We excluded study from the VTE estimate because of overlapping population, but included it to transfusion estimate.
Basta 2016: Open ventral hernia repair: We excluded the study from the VTE estimate because of risk of bias.
Scmidbauer 2005: Open ventral hernia repair: We excluded the study from the VTE estimate because of risk of bias.

2. Design features used for assessment of risk of bias

Domain*	Low risk of bias	High risk of bias
**Sampling**	Consecutive patient recruitment or administrative database with random sampling	Non-consecutive patient recruitment or administrative database with non-random sampling
**Thromboprophylaxis documentation**	Reporting of patients' thromboprophylaxis	No reporting of patients' thromboprophylaxis
**Source of information**	Prospective data collection by study investigators	Retrospective duplicate chart reviews without documentation of agreement between reviewers
	Retrospective duplicate chart reviews with good documentation of agreement between reviewers	Administrative database information
**Recruitment years**	Studies with the majority of patient recruitment years 2010 or after	Studies with the majority of patient recruitment years 2009 or before
**Specification of length of follow-up**	Studies that clearly define the time period of follow-up (up to 3 months)	Studies that do not clearly define the time period of follow-up
**Study type**	International multicenter; Multicenter in one country; Single center, not single surgeon	Single surgeon series
**Overall risk of bias***	No high risk of bias domains: Very low risk of bias<br>One high risk of bias domain: Low risk of bias<br>Two high risk of bias domains: Moderate risk of bias<br>Three or more high risk of bias domains: High risk of bias	

*We used the overall risk of bias as eligibility criteria when there were a sufficient number of patients in studies with very low, low or moderate risk of bias for a given procedure (see the article for more details).

3. Risk of bias in individual studies in general abdominal surgery

Reference	Sampling	Thromboprophylaxis documentation	Source of information	Recruitment years	Specification of length of follow-up	Study type	Risk of Bias
**Appendectomy, laparoscopic**							
Nguyen 2007	+	-	Administrative database information	-	-	Multicenter in one country	HIGH
Hemmila 2010	+	-	Prospective data collection	-	+	Multicenter in one country	MODERATE
Brugger 2011	+	-	Administrative database information	-	-	Multicenter in one country	HIGH
Alizadeh 2017	+	-	Prospective data collection	+	+	Multicenter in one country	LOW
Chung 2019	+	-	Administrative database information	-	-	Multicenter in one country	HIGH
Garcia 2019	+	-	Administrative database information	+	-	Multicenter in one country	HIGH
**Appendectomy, open**							
Nguyen 2007	+	-	Administrative database information	-	-	Multicenter in one country	HIGH
Hemmila 2010	+	-	Prospective data collection	-	+	Multicenter in one country	MODERATE
Chung 2019	+	-	Administrative database information	-	-	Multicenter in one country	HIGH
Garcia 2019	+	-	Administrative database information	+	-	Multicenter in one country	HIGH
**Appendectomy, laparoscopic, emergency**							
Brugger 2011	+	-	Administrative database information	-	-	Multicenter in one country	HIGH
Sakran 2019	+	-	Prospective data collection	+	+	Multicenter in one country	LOW
**Appendectomy, open, emergency**							
Sakran 2019	+	-	Prospective data collection	+	+	Multicenter in one country	LOW
**Cholecystectomy, conversion to open**							
Persson 2012	+	-	Administrative database information	-	+	Multicenter in one country	HIGH

Cholecystectomy, laparoscopic

Blake 2001	+	+	Retrospective chart reviews, data collected by one investigator	-	-	One center, multiple surgeons	HIGH
Schaepkens Van Riempst 2002	+	+	Prospective data collection	-	+	Multicenter in one country	LOW
Engbaek 2006	+	-	Retrospective duplicate chart reviews without documentation of agreement between reviewers	-	+	Multicenter in one country	HIGH
Lindberg 2006	-	+	Prospective data collection	-	+	One center, multiple surgeons	MODERATE
Nguyen 2007	+	-	Administrative database information	-	-	Multicenter in one country	HIGH
Rathore 2007	+	+	Retrospective chart reviews, data collected by one investigator	-	+	One center, multiple surgeons	MODERATE
Triantafyllidis 2009	+	-	Retrospective chart reviews, data collected by one investigator	-	-	One center, multiple surgeons	HIGH
Ingraham 2010	+	-	Prospective data collection	-	+	Multicenter in one country	MODERATE
Ntourakis 2011	-	+	Prospective data collection	-	+	One center, multiple surgeons	MODERATE
Hasbahceci 2012	-	-	Retrospective chart reviews, data collected by one investigator	-	+	One center, multiple surgeons	HIGH
Pakaneh 2012	+	+	Prospective data collection	-	+	One center, multiple surgeons	LOW
Persson 2012	+	-	Administrative database information	-	+	Multicenter in one country	HIGH
Stein 2014	+	-	Administrative database information	-	-	Multicenter in one country	HIGH
Suuronen 2015	+	-	Administrative database information	-	-	Multicenter in one country	HIGH
Donkervoort 2016	+	-	Retrospective chart reviews, data collected by one investigator	-	-	Multicenter in one country	HIGH
Ulrych 2016	+	+	Prospective data collection	+	+	One center, multiple surgeons	VERY LOW
Gundogdu 2017	-	+	Retrospective duplicate chart reviews without documentation of agreement between reviewers	+	+	One center, multiple surgeons	MODERATE
Rosero 2017	-	-	Retrospective chart reviews, data collected by one investigator	+	+	Multicenter in one country	HIGH

Sepassi 2018	+	-	Administrative database information	+	-	Multicenter in one country	HIGH
Coelho 2019	+	-	Retrospective chart reviews, data collected by one investigator	+	-	One center, multiple surgeons	HIGH
Rysmakhanov 2019	+	-	Retrospective chart reviews, data collected by one investigator	+	-	One center, multiple surgeons	HIGH
Ross 2020	+	-	Prospective data collection	+	+	Multicenter in one country	LOW
**Cholecystectomy, laparoscopic, elective**							
Schaepkens Van Riempst 2002	+	+	Prospective data collection	-	+	Multicenter in one country	LOW
Rathore 2007	+	+	Retrospective chart reviews, data collected by one investigator	-	+	One center, multiple surgeons	MODERATE
Ntourakis 2011	-	+	Prospective data collection	-	+	One center, multiple surgeons	MODERATE
Ulrych 2016	+	+	Prospective data collection	+	+	One center, multiple surgeons	VERY LOW
Gundogdu 2017	-	+	Retrospective duplicate chart reviews without documentation of agreement between reviewers	+	+	One center, multiple surgeons	MODERATE
Sepassi 2018	+	-	Administrative database information	+	-	Multicenter in one country	HIGH
**Cholecystectomy, laparoscopic, emergency**							
Sakran 2019	+	-	Prospective data collection	+	+	Multicenter in one country	LOW
**Cholecystectomy, open**							
Nguyen 2007	+	-	Administrative database information	-	-	Multicenter in one country	HIGH
Ingraham 2010	+	-	Prospective data collection	-	+	Multicenter in one country	MODERATE
Persson 2012	+	-	Administrative database information	-	+	Multicenter in one country	HIGH
Suuronen 2015	+	-	Administrative database information	-	-	Multicenter in one country	HIGH
Sakran 2019	+	-	Prospective data collection	+	+	Multicenter in one country	LOW
Ross 2020	+	-	Prospective data collection	+	+	Multicenter in one country	LOW
**Cholecystectomy, open, emergency**							
Sakran 2019	+	-	Prospective data collection	+	+	Multicenter in one country	LOW

**Hernia repair, groin, laparoscopic**							
Al-Sahaf 2008	+	-	Retrospective chart reviews, data collected by one investigator	-	-	Single surgeon series	HIGH
Srsen 2008	+	-	Retrospective chart reviews, data collected by one investigator	-	-	One center, multiple surgeons	HIGH
Meyer 2013	+	-	Retrospective duplicate chart reviews with good documentation of agreement between reviewers	-	-	Multinational	HIGH
Wakasugi 2016	+	-	Retrospective chart reviews, data collected by one investigator	+	-	One center, multiple surgeons	HIGH
Wakasugi 2017	-	-	Retrospective chart reviews, data collected by one investigator	+	-	One center, multiple surgeons	HIGH
Mita 2020	+	+	Retrospective chart reviews, data collected by one investigator	+	+	One center, multiple surgeons	LOW
Perez 2020	+	-	Retrospective chart reviews, data collected by one investigator	+	-	Multicenter in one country	HIGH
Wang 2020	+	-	Retrospective chart reviews, data collected by one investigator	+	-	Multicenter in one country	HIGH
Yang 2019	+	+	Prospective data collection	+	+	One center, multiple surgeons	LOW
**Hernia repair, groin, open**							
Holzheimer 2007	+	-	Prospective data collection	-	-	One center, multiple surgeons	HIGH
Srsen 2008	+	-	Retrospective chart reviews, data collected by one investigator	-	-	One center, multiple surgeons	HIGH
Bessa 2015	+	-	Prospective data collection	-	-	One center, multiple surgeons	HIGH
Lozano 2015	-	+	Prospective data collection	-	+	One center, multiple surgeons	MODERATE
Nilsson 2016	+	-	Prospective data collection	-	+	Multicenter in one country	MODERATE
Tastaldi 2019	+	-	Administrative database information	+	+	One center, multiple surgeons	MODERATE
Liu 2020	+	-	Retrospective chart reviews, data collected by one investigator	+	-	One center, multiple surgeons	HIGH
Perez 2020	+	-	Retrospective chart reviews, data collected by one investigator	+	-	Multicenter in one country	HIGH

Poudel 2020	+	+	Retrospective chart reviews, data collected by one investigator	+	+	Single surgeon series	MODERATE
Wang 2020	+	-	Retrospective chart reviews, data collected by one investigator	+	-	Multicenter in one country	HIGH
**Hernia repair, groin, minimally-invasive, elective**							
Srsen 2008	+	-	Retrospective chart reviews, data collected by one investigator	-	-	One center, multiple surgeons	HIGH
Meyer 2013	+	-	Retrospective duplicate chart reviews with good documentation of agreement between reviewers	-	-	Multinational	HIGH
Mita 2020	+	+	Retrospective chart reviews, data collected by one investigator	+	+	One center, multiple surgeons	LOW
Yang 2019	+	+	Prospective data collection	+	+	One center, multiple surgeons	LOW
**Hernia repair, groin, open, elective**							
Srsen 2008	+	-	Retrospective chart reviews, data collected by one investigator	-	-	One center, multiple surgeons	HIGH
Lozano 2015	-	+	Prospective data collection	-	+	One center, multiple surgeons	MODERATE
Nilsson 2016	+	-	Prospective data collection	-	+	Multicenter in one country	MODERATE
**Hernia repair, groin, open, emergency**							
Bessa 2015	+	-	Prospective data collection	-	-	One center, multiple surgeons	HIGH
Nilsson 2016	+	-	Prospective data collection	-	+	Multicenter in one country	MODERATE
Tastaldi 2019	+	-	Administrative database information	+	+	One center, multiple surgeons	MODERATE
Liu 2020	+	-	Retrospective chart reviews, data collected by one investigator	+	-	One center, multiple surgeons	HIGH
**Hernia repair, ventral, laparoscopic**							
Lomanto 2006	+	+	Prospective data collection	-	-	One center, multiple surgeons	MODERATE
Ferrari 2008	+	+	Retrospective chart reviews, data collected by one investigator	-	-	One center, multiple surgeons	HIGH
Sharma 2011	+	-	Retrospective chart reviews, data collected by one investigator	-	-	One center, multiple surgeons	HIGH

Aher 2015	+	-	Prospective data collection	+	+	Multicenter in one country	LOW
Warren 2017	+	-	Prospective data collection	+	-	One center, multiple surgeons	MODERATE
Boules 2018	-	-	Retrospective chart reviews, data collected by one investigator	-	+	One center, multiple surgeons	HIGH
Ross 2020	+	-	Prospective data collection	+	+	Multicenter in one country	LOW
Zolin 2020	+	-	Prospective data collection	+	+	One center, multiple surgeons	LOW
**Hernia repair, ventral, open**							
Schmidbauer 2005	+	-	Retrospective chart reviews, data collected by one investigator	-	-	One center, multiple surgeons	HIGH
Aher 2015	+	-	Prospective data collection	+	+	Multicenter in one country	LOW
Basta 2016	+	-	Prospective data collection	+	+	Single surgeon series	MODERATE
Ulrych 2016	+	+	Prospective data collection	+	+	One center, multiple surgeons	VERY LOW
Bittner 2018	+	+	Retrospective chart reviews, data collected by one investigator	+	+	One center, multiple surgeons	LOW
Kraft 2019	+	+	Prospective data collection	+	+	Single surgeon series	LOW
Ross 2020	+	-	Prospective data collection	+	+	Multicenter in one country	LOW
Zolin 2020	+	-	Prospective data collection	+	+	One center, multiple surgeons	LOW
**Hernia repair, ventral, robotic**							
Warren 2017	+	-	Prospective data collection	+	-	One center, multiple surgeons	MODERATE
**Hernia repair, ventral, laparoscopic, elective**							
Lomanto 2006	+	+	Prospective data collection	-	-	One center, multiple surgeons	MODERATE
Aher 2015	+	-	Prospective data collection	+	+	Multicenter in one country	LOW
Boules 2018	-	-	Retrospective chart reviews, data collected by one investigator	-	+	One center, multiple surgeons	HIGH
Zolin 2020	+	-	Prospective data collection	+	+	One center, multiple surgeons	LOW
**Hernia repair, ventral, laparoscopic, emergency**							

Sakran 2019	+	-	Prospective data collection	+	+	Multicenter in one country	LOW
**Hernia repair, ventral, open, elective**							
Aher 2015	+	-	Prospective data collection	+	+	Multicenter in one country	LOW
Ulrych 2016	+	+	Prospective data collection	+	+	One center, multiple surgeons	VERY LOW
Bittner 2018	+	+	Retrospective chart reviews, data collected by one investigator	+	+	One center, multiple surgeons	LOW
Kraft 2019	+	+	Prospective data collection	+	+	Single surgeon series	LOW
Zolin 2020	+	-	Prospective data collection	+	+	One center, multiple surgeons	LOW
**Hernia repair, ventral, open, emergency**							
Sakran 2019	+	-	Prospective data collection	+	+	Multicenter in one country	LOW
**Small bowel resection, laparoscopic**							
Daly 2014	+	-	Prospective data collection	+	+	Multicenter in one country	LOW
McKenna 2018	+	-	Prospective data collection	+	+	Multicenter in one country	LOW
**Small bowel resection, open**							
Daly 2014	+	-	Prospective data collection	+	+	Multicenter in one country	LOW
McKenna 2018	+	-	Prospective data collection	+	+	Multicenter in one country	LOW
Sakran 2019	+	-	Prospective data collection	+	+	Multicenter in one country	LOW
**Small bowel resection, laparoscopic, malignant**							
McKenna 2018	+	-	Prospective data collection	+	+	Multicenter in one country	LOW
**Small bowel resection, laparoscopic, IBD**							
McKenna 2018	+	-	Prospective data collection	+	+	Multicenter in one country	LOW
**Small bowel resection, laparoscopic, benign**							
McKenna 2018	+	-	Prospective data collection	+	+	Multicenter in one country	LOW
**Small bowel resection, laparoscopic, emergency**							
McKenna 2018	+	-	Prospective data collection	+	+	Multicenter in one country	LOW

**Small bowel resection, open, IBD**							
McKenna 2018	+	-	Prospective data collection	+	+	Multicenter in one country	LOW
**Small bowel resection, open, benign**							
McKenna 2018	+	-	Prospective data collection	+	+	Multicenter in one country	LOW
**Small bowel resection, open, malignant**							
McKenna 2018	+	-	Prospective data collection	+	+	Multicenter in one country	LOW
**Small bowel resection, open, emergency**							
Sakran 2019	+	-	Prospective data collection	+	+	Multicenter in one country	LOW
**Splenectomy, elective, laparoscopic**							
Delaitre 2002	+	-	Retrospective chart reviews, data collected by one investigator	-	-	Multicenter in one country	HIGH
Patel 2003	+	-	Retrospective chart reviews, data collected by one investigator	-	-	One center, multiple surgeons	HIGH
Romano 2006	+	+	Retrospective chart reviews, data collected by one investigator	-	-	One center, multiple surgeons	HIGH
Casaccia 2010	+	-	Administrative database information	-	-	Multicenter in one country	HIGH
Vecchio 2011	-	-	Retrospective chart reviews, data collected by one investigator	-	-	One center, multiple surgeons	HIGH
Corcione 2012	+	-	Retrospective chart reviews, data collected by one investigator	-	-	One center, multiple surgeons	HIGH
Wang 2013	-	-	Retrospective chart reviews, data collected by one investigator	-	-	Single surgeon series	HIGH
Radkowiak 2018	+	-	Retrospective chart reviews, data collected by one investigator	-	+	One center, multiple surgeons	HIGH
Tsamalaidze 2018	+	-	Retrospective chart reviews, data collected by one investigator	-	+	One center, multiple surgeons	HIGH
Zychowicz 2018	+	-	Retrospective chart reviews, data collected by one investigator	-	-	One center, multiple surgeons	HIGH
Tastaldi 2019	+	-	Retrospective chart reviews, data collected by one investigator	-	+	Single surgeon series	HIGH
Hernandez 2020	+	-	Prospective data collection	+	+	Multicenter in one country	LOW

**Splenectomy, elective, open**							
Mesa 2006	-	-	Retrospective chart reviews, data collected by one investigator	-	+	One center, multiple surgeons	HIGH
Romano 2006	+	+	Retrospective chart reviews, data collected by one investigator	-	-	One center, multiple surgeons	HIGH
Zhang 2012	+	-	Retrospective duplicate chart reviews without documentation of agreement between reviewers	-	-	One center, multiple surgeons	HIGH
Jiang 2014	+	-	Retrospective chart reviews, data collected by one investigator	+	-	One center, multiple surgeons	HIGH
Li 2017	+	-	Retrospective chart reviews, data collected by one investigator	-	-	One center, multiple surgeons	HIGH
Tsamalaidze 2018	+	-	Retrospective chart reviews, data collected by one investigator	-	+	One center, multiple surgeons	HIGH
Hernandez 2020	+	-	Prospective data collection	+	+	Multicenter in one country	LOW
**Splenectomy, elective, laparoscopic, benign**							
Delaitre 2002	+	-	Retrospective chart reviews, data collected by one investigator	-	-	Multicenter in one country	HIGH
Wang 2013	-	-	Retrospective chart reviews, data collected by one investigator	-	-	Single surgeon series	HIGH
Zychowicz 2018	+	-	Retrospective chart reviews, data collected by one investigator	-	-	One center, multiple surgeons	HIGH
Tastaldi 2019	+	-	Retrospective chart reviews, data collected by one investigator	-	+	Single surgeon series	HIGH
**Splenectomy, elective, open, benign**							
Zhang 2012	+	-	Retrospective duplicate chart reviews without documentation of agreement between reviewers	-	-	One center, multiple surgeons	HIGH
Jiang 2014	+	-	Retrospective chart reviews, data collected by one investigator	+	-	One center, multiple surgeons	HIGH
Li 2017	+	-	Retrospective chart reviews, data collected by one investigator	-	-	One center, multiple surgeons	HIGH

**Splenectomy, elective, open, malignant**							
Mesa 2006	-	-	Retrospective chart reviews, data collected by one investigator	-	+	One center, multiple surgeons	HIGH

*Articles are reported by procedure, so duplicate information from same study appears in this table.

4. Prophylaxis in individual studies in general abdominal surgery

Reference	Total patients	Mechanical prophylaxis			Antiplatelet drugs			Anticoagulants		
	n	%	Type	Duration in days	%	Type	Duration in days	%	Type	Duration in days
**Appendectomy, laparoscopic**										
Nguyen 2007	24,509									
Hemmila 2010	15,445									
Brugger 2011	7,446									
Alizadeh 2017	168,963									
Chung 2019	52,767									
Garcia 2019	83,712									
**Appendectomy, open**										
Nguyen 2007	25,554									
Hemmila 2010	6,030									
Chung 2019	193,845									
Garcia 2019	12,665									
**Appendectomy, laparoscopic, emergency**										
Brugger 2011	7,446									
Sakran 2019	65,017									
**Appendectomy, open, emergency**										
Sakran 2019	6,292									
**Cholecystectomy, conversion to open**										

Persson 2012	3,768				44†	Unspecified	4†
**Cholecystectomy, laparoscopic**							
Blake 2001	587	2	IPC		1	LMWH<br>Warfarin	
Schaepkens Van Riempst 2002	238	0	none	0	44	LMWH	4
Engbaek 2006	258						
Lindberg 2006	50	0	none	14	52	LMWH	2†
Nguyen 2007	50,527						
Rathore 2007	164	100	IPC, GCS		100	LMWH	
Triantafyllidis 2009	1,009	100	GCS		100	LMWH	
Ingraham 2010	58,659						
Ntourakis 2011	119	0	none	0	0		
Hasbahceci 2012	1,557						
Pakaneh 2012	100	0	none	0	0		
Persson 2012	42,271				44†	Unspecified	4†
Stein 2014	4,107,430						
Suuronen 2015	17,175						
Donkervoort 2016	4,359						
Ulrych 2016	90	100	lower extremity bandages		100	LMWH	3
Gundogdu 2017	1,485			0	79	LMWH	2
Rosero 2017	230,745						
Sepassi 2018	518						
Coelho 2019	1,645						
Rysmakhanov 2019	1,658						
Ross 2020	256,726						
**Cholecystectomy, laparoscopic, elective**							
Schaepkens Van Riempst 2002	238	0		0	44	LMWH	4

Rathore 2007	164	100	IPC, GCS				100	LMWH	
Ntourakis 2011	119	0		0			0		
Ulrych 2016	90	100	lower extremity bandages				100	LMWH	3
Gundogdu 2017	1,485			0			79	LMWH	4
Sepassi 2018	518								
**Cholecystectomy, laparoscopic, emergency**									
Sakran 2019	11,266								
**Cholecystectomy, open**									
Nguyen 2007	14,513								
Ingraham 2010	6,852								
Persson 2012	4,370						44†	Unspecified	4†
Suuronen 2015	4,942								
Sakran 2019	1,447								
Ross 2020	37,311								
**Cholecystectomy, open, emergency**									
Sakran 2019	1,447								
**Hernia repair, groin, laparoscopic**									
Al-Sahaf 2008	108								
Srsen 2008	82						100	LMWH	
Meyer 2013	4,565								
Wakasugi 2016	365								
Wakasugi 2017	350								
Mita 2020	413	100	IPC, GCS	13	DAPT (18), aspirin (36)	30	7	UFH/Other	30
Perez 2020	5,282								
Wang 2020	7,110	25	Unspecified				3	Unspecified	
Yang 2019	144	100	IPC	0			0		0
**Hernia repair, groin, open**									

Holzheimer 2007	300	100	GCS				100	LMWH	
Srsen 2008	134						100	LMWH	
Bessa 2015	234						33	LMWH	
Lozano 2015	218						75	LMWH	7
Nilsson 2016	140,567								
Tastaldi 2019	257								
Liu 2020	146							LMWH	
Perez 2020	36,575								
Poudel 2020	4,870			10	aspirin, clopidogrel, ticlopidine, cilostazol, other	30	3	Warfarin/DOAC	Continuous†
Wang 2020	6,776	26	"Instrument"				3	Unspecified	
**Hernia repair, groin, minimally-invasive, elective**									
Srsen 2008	82						100	LMWH	
Meyer 2013	4,565								
Mita 2020	413	100	IPC, GCS	13	DAPT (18), aspirin (36)	30	7	UFH/Other	30
Yang 2019	144	100	IPC	0			0		0
**Hernia repair, groin, open, elective**									
Srsen 2008	134						100	LMWH	
Lozano 2015	218						75	LMWH	7
Nilsson 2016	132,801								
**Hernia repair, groin, open, emergency**									
Bessa 2015	234						33	LMWH	
Nilsson 2016	7,766								
Tastaldi 2019	257								
Liu 2020	146							LMWH	
**Hernia repair, ventral, laparoscopic**									
Lomanto 2006	50	0†	None†				0†		
Ferrari 2008	100	38	GCS				100	LMWH	1
Sharma 2011	1,242								

Aher 2015	26,286							
Warren 2017	103							
Boules 2018	361							
Ross 2020	33,630							
Zolin 2020	81							
**Hernia repair, ventral, open**								
Schmidbauer 2005	175					100	LMWH	10
Aher 2015	90,721							
Basta 2016	142							
Ulrych 2016	126	100	lower extremity bandages			100	LMWH	3
Bittner 2018	76		IPC					
Kraft 2019	175			10	Predominantly aspirin	99	LMWH/UFH	5
Ross 2020	128,513							
Zolin 2020	105							
**Hernia repair, ventral, robotic**								
Warren 2017	53							
**Hernia repair, ventral, laparoscopic, elective**								
Lomanto 2006	50	0†	None†			0†		
Aher 2015	26,286							
Boules 2018	361							
Zolin 2020	81							
**Hernia repair, ventral, laparoscopic, emergency**								
Sakran 2019	405							
Hernia repair, ventral, open, elective	0							

Aher 2015	90,721							
Ulrych 2016	126	100	lower extremity bandages			100	LMWH	3
Bittner 2018	76		IPC					
Kraft 2019	175			10	Predominantly aspirin	99	LMWH/UFH	5
Zolin 2020	105							
**Hernia repair, ventral, open, emergency**								
Sakran 2019	4,808							
**Small bowel resection, laparoscopic**								
Daly 2014	1,780		unknown					
McKenna 2018	1,415		unknown					
**Small bowel resection, open**								
Daly 2014	17,701		unknown					
McKenna 2018	3,592		unknown†					
Sakran 2019	6,855							
**Small bowel resection, laparoscopic, malignant**								
McKenna 2018	499		unknown					
**Small bowel resection, laparoscopic, IBD**								
McKenna 2018	443		unknown					
**Small bowel resection, laparoscopic, benign**								
McKenna 2018	355		unknown					
**Small bowel resection, laparoscopic, emergency**								
McKenna 2018	118		unknown†					
**Small bowel resection, open, IBD**								
McKenna 2018	1,237		unknown					
**Small bowel resection, open, benign**								

McKenna 2018	571		unknown							
**Small bowel resection, open, malignant**										
McKenna 2018	1,784		unknown							
**Small bowel resection, open, emergency**										
Sakran 2019	6,855									
**Splenectomy, elective, laparoscopic**										
Delaitre 2002	209							100	LMWH	
Patel 2003	108									
Romano 2006	72							100	LMWH	
Casaccia 2010	676									
Vecchio 2011	107									
Corcione 2012	300									
Wang 2013	260									
Radkowiak 2018	500									
Tsamalaidze 2018	101									
Zychowicz 2018	194									
Tastaldi 2019	109	100	IPC	2				100	LMWH	2
Hernandez 2020	4,365									
**Splenectomy, elective, open**										
Mesa 2006	314				17	Usually with aspirin and occasionally with anagrelide				
Romano 2006	86							100	LMWH	
Zhang 2012	69							0		0
Jiang 2014	71									
Li 2017	56									

Tsamalaidze 2018	86									
Hernandez 2020	2,220									
**Splenectomy, elective, laparoscopic, benign**										
Delaitre 2002	209							100	LMWH	
Wang 2013	260									
Zychowicz 2018	194									
Tastaldi 2019	109	100	IPC	2				100	LMWH	2
**Splenectomy, elective, open, benign**										
Zhang 2012	69							0		0
Jiang 2014	71									
Li 2017	56									
**Splenectomy, elective, open, malignant**										
Mesa 2006	314				17	Usually with aspirin and occasionally with anagrelide				

Mechanical thromboprophylaxis included: antithrombosis stockings, intermittent pneumatic compression devices, and foot-pumps
Aspirin or other antiplatelet drugs included: aspirin, clopidogrel, prasugrel, ticlopidine, dipyridamole, ticagrelor, cilostazol, tirofiban, vorapaxar as well as thromboxane inhibitors, thromboxane synthase inhibitors, thromboxane receptor antagonists, and terutroban
Anticoagulants included: warfarin, low molecular weight heparin, low dose unfractionated heparin, dabigatran, apixaban, betrixaban, edoxaban, rivaroxaban, fondaparinux, danaparoid and lepirudin
Blank spaces represent no information (not provided by paper or by author correspondence).
Duration in days is expressed as mean or median.
GCS=graduated compression stockings; IPC= intermittent pneumatic compression (includes "intermittent compression device, sequential compression device, pneumatic compression device, pneumatic compression stockings, pneumatic compression boots"); LMWH= low molecular weight heparin; UFH= unfractionated heparin.
† Author provided this information. §Follow up time of complications was not available from the article or author correspondence. We assumed a follow up time of 30 days.

5. Postoperative risk of symptomatic VTE and bleeding in individual studies in general abdominal surgery

Reference	Total patients	Follow-up time	Reported VTE					Reported Bleeding			Baseline cumulative incidence at 4 weeks		
	n	Days	Fatal PE	Non-Fatal PE	DVT	VTE total* (excluding SVT)	SVT	Fatal Bleeding	Bleeding requiring reintervention	Transfusion	VTE at 4 weeks (%)	Bleeding requiring reintervention at 4 weeks (%)	Bleeding requiring transfusion at 4 weeks (%)
**Appendectomy, laparoscopic**													
Nguyen 2007	24,509	30§				27					0.1%		
Hemmila 2010	15,445	30		15	22	36‡					0.2%		0.0%
Brugger 2011	7,446	30§		11	1	12‡				10	0.2%		0.1%
Alizadeh 2017	168,963	30				270					0.2%		
Chung 2019	52,767	30§		20	60	77					0.1%		
Garcia 2019	83,712	30§				151					0.2%		
**Appendectomy, open**													
Nguyen 2007	25,554	30§				72					0.3%		
Hemmila 2010	6,030	30		6	18	23‡				1	0.4%		0%
Chung 2019	193,845	30§		297	713	948					0.5%		
Garcia 2019	12,665	30§				71					0.6%		
**Appendectomy, laparoscopic, emergency**						0‡							
Brugger 2011	7,446	30§		11	1	12‡				10	0.2%		0.1%
Sakran 2019	65,017	30		40	83	119‡					0.2%		
**Appendectomy, open, emergency**													
Sakran 2019	6,292	30		19	25	43‡					0.7%		
**Cholecystectomy, conversion to open**													

Persson 2012	3,768	30								49			1.1%
**Cholecystectomy, laparoscopic**													
Blake 2001	587	28	0	0	0	0		0	3		0%	0.5%	
Schaepkens Van Riempst 2002	238	10	0	0	0	0					0%		
Engbaek 2006	258	60	0	0	0	0‡		0			0%		
Lindberg 2006	50	7†	0	0	0	0		0†	0†		0%	0%	
Nguyen 2007	50,527	30§				182					0.4%		
Rathore 2007	164	30							1			0.4%	
Triantafyllidis 2009	1,009	30§							7	2		0.5%	0.1%
Ingraham 2010	58,659					0‡							0%
Ntourakis 2011	119	8			0	0‡					0%		
Hasbahceci 2012	1,557	90†	0†	2†		8‡			4		0.3%	0.3%	
Pakaneh 2012	100	30	0	0	0	0		0			0%		
Persson 2012	42,271	30				53				85	0.1%		0.2%
Stein 2014	4,107,430	30§	780	5180	16610	21630					0.6%		
Suuronen 2015	17,175	30§											0.8%
Donkervoort 2016	4,359	30§		4		16‡			30		0.4%	0.7%	
Ulrych 2016	90	30		0	0	0	0		0	0	0%	0%	0%
Gundogdu 2017	1,485	30	0	0	0	0	0	0	0	0	0%	0%	0%
Rosero 2017	230,745	30				72					0%		
Sepassi 2018	518	30§				1					0.2%		
Coelho 2019	1,645	30§	0			9		0			0.5%		
Rysmakhanov 2019	1,658	30§	1					0	3			0.2%	
Ross 2020	256,726	30				682					0.3%		
**Cholecystectomy, laparoscopic, elective**						0‡							
Schaepkens Van Riempst 2002	238	10	0	0	0	0					0%		

Rathore 2007	164	30							1			0.4%	
Ntourakis 2011	119	8			0	0‡					0%		
Ulrych 2016	90	30		0	0	0	0		0	0	0%	0%	0%
Gundogdu 2017	1,485	30	0	0	0	0	0	0	0	0	0%	0%	0%
Sepassi 2018	518	30§				1					0.2%		
**Cholecystectomy, laparoscopic, emergency**													
Sakran 2019	11,266	30		14	25	38‡					0.3%		
Cholecystectomy, open						0‡							
Nguyen 2007	14,513	30§				149					1.3%		
Ingraham 2010	6,852	30		31	32	61‡					1.1%		0.2%
Persson 2012	4,370	30				21				35	0.5%		0.7%
Suuronen 2015	4,942	30§											5.8%
Sakran 2019	1,447	30		4	16	19‡					1.7%		
Ross 2020	37,311	30				936					3.1%		
**Cholecystectomy, open, emergency**						0‡							
Sakran 2019	1,447	30		4	16	19‡					1.7%		
**Hernia repair, groin, laparoscopic**													
Al-Sahaf 2008	108	30§							1			0.9%	
Srsen 2008	82	30§	0	0	0	0	0				0%		
Meyer 2013	4,565	30§	1						10			0.2%	
Wakasugi 2016	365	30§	0	1		4‡					1%		
Wakasugi 2017	350	30§		1		4‡					1.2%		
Mita 2020	413	30							0	0		0%	0%
Perez 2020	5,282	30§				158					3.1%		
Wang 2020	7,110	30§				10					0.1%		
Yang 2019	144	30	0	0	0	0					0%		
**Hernia repair, groin, open**						0‡							

Holzheimer 2007	300	90	0	0	0	0‡		0			0%		
Srsen 2008	134	30§					0	0	1			0.5%	
Bessa 2015	234	30§	0	1	2	3‡		0			1.3%		
Lozano 2015	218	30	0	0	0	0			0		0%	0%	
Nilsson 2016	140,567	30		73		288‡					0.2%		
Tastaldi 2019	257	30	1			4		0			1.6%		
Liu 2020	146	30§	1	0	2	3‡		0			2%		
Perez 2020	36,575	30§				1289					3.6%		
Poudel 2020	4,870	30†	0†	0†	0†	0†	0†	0†	4†	1†	0%	0.1%	0%
Wang 2020	6,776	30§				6					0.1%		
**Hernia repair, groin, minimally-invasive, elective**													
Srsen 2008	82	30§	0	0	0	0	0				0%		
Meyer 2013	4,565	30§	1						10			0.2%	
Mita 2020	413	30							0	0		0%	0%
Yang 2019	144	30	0	0	0	0					0%		
**Hernia repair, groin, open, elective**													
Srsen 2008	134	30§					0	0	1			0.5%	
Lozano 2015	218	30	0	0	0	0			0		0%	0%	
Nilsson 2016	132,801	30		47		186‡					0.1%		
**Hernia repair, groin, open, emergency**													
Bessa 2015	234	30§	0	1	2	3‡		0			1.3%		
Nilsson 2016	7,766	30		26		103‡					1.3%		
Tastaldi 2019	257	30	1			4		0			1.6%		
Liu 2020	146	30§	1	0	2	3‡		0	0		2%	0%	
**Hernia repair, ventral, laparoscopic**													
Lomanto 2006	50	30§			0	0‡					0%		
Ferrari 2008	100	30§	1					0					
Sharma 2011	1,242	90	1		10	14‡					0.7%		
Aher 2015	26,286					0‡							0.1%

Warren 2017	103	30§							0		0%	
Boules 2018	361	30	0	8	13	20‡		0	1	5.4%	0.2%	
Ross 2020	33,630	30				131				0.4%		
Zolin 2020	81	30		0		0‡				0%		
**Hernia repair, ventral, open**												
Schmidbauer 2005	175								2		0.9%	
Aher 2015	90,721					0‡						0.1%
Basta 2016	142								3		1.4%	
Ulrych 2016	126	30	0	0	1	1	0	0	2	0.8%	1.1%	
Bittner 2018	76	90		1	0	1‡				0.9%		
Kraft 2019	175	30	0	4	0	4			0	2.7%	0%	
Ross 2020	128,513	30				936				0.9%		
Zolin 2020	105	30		1		4‡				4.5%		
**Hernia repair, ventral, robotic**												
Warren 2017	53	30§							0		0%	
**Hernia repair, ventral, laparoscopic, elective**												
Lomanto 2006	50	30§			0	0‡				0%		
Aher 2015	26,286	30		57	62	115‡				0.5%		0.1%
Boules 2018	361	30	0	8	13	20‡		0	1	5.6%	0.2%	
Zolin 2020	81	30		0		0‡				0%		
**Hernia repair, ventral, laparoscopic, emergency**												
Sakran 2019	405	30		1	4	5‡				1.2%		
**Hernia repair, ventral, open, elective**						0‡						
Aher 2015	90,721	30		222	300	506‡				0.7%		0.1%

Ulrych 2016	126	30	0	0	1	1	0	0	2		0.8%	1.1%	
Bittner 2018	76	90		1	0	1‡					0.9%		
Kraft 2019	175	30	0	4	0	4			0		2.7%	0%	
Zolin 2020	105	30		1		4‡					4.5%		
**Hernia repair, ventral, open, emergency**						0‡							
Sakran 2019	4,808	30		26	51	75‡					1.6%		
**Small bowel resection, laparoscopic**						0‡							
Daly 2014	1,780	30		11	14	24‡					1.7%		2.5%
McKenna 2018	1,415	30				16†					1.4%		
**Small bowel resection, open**													
Daly 2014	17,701	30		177	443	600‡					4.3%		7.4%
McKenna 2018	3,592	30				72†					2.5%		0%
Sakran 2019	6,855	30		60	148	202‡					3.7%		
**Small bowel resection, laparoscopic, malignant**						0‡							
McKenna 2018	499	30				9†					2.3%		
**Small bowel resection, laparoscopic, IBD**						0‡							
McKenna 2018	443	30				4†					1.1%		
**Small bowel resection, laparoscopic, benign**						0‡							
McKenna 2018	355	30				3†					1.1%		
**Small bowel resection, laparoscopic, emergency**						0‡							
McKenna 2018	118	30				0†					0%		
**Small bowel resection, open, IBD**						0‡							
McKenna 2018	1,237	30				20†					2%		
**Small bowel resection, open, benign**						0‡							
McKenna 2018	571	30				4†					0.9%		

**Small bowel resection, open, malignant**						0‡							
McKenna 2018	1,784	30				48†					3.4%		
**Small bowel resection, open, emergency**						0‡							
Sakran 2019	6,855	30		60	148	202‡					3.7%		
**Splenectomy, elective, laparoscopic**													
Delaitre 2002	209	30§			1	1‡		0	2		0.9%	0.7%	
Patel 2003	108	30§	1				1	0	2			1.3%	
Romano 2006	72	30§					4						
Casaccia 2010	676	30§					14	0	26			2.6%	
Vecchio 2011	107	30§					3	0	2			1.3%	
Corcione 2012	300	30§	0	1	1	2‡	1	0	1		1%	0.2%	
Wang 2013	260	30§					7			5			0%
Radkowiak 2018	500	30	1				1	1	10	42		1.4%	3.7%
Tsamalaidze 2018	101	30					1						
Zychowicz 2018	194	30§		1		4‡			4		3.1%	1.4%	
Tastaldi 2019	109	30	0	0	3	3	2	0	1		2.9%	0.7%	
Hernandez 2020	4,365	30		34	111	140‡					3.3%		0.9%
**Splenectomy, elective, open**						0‡							
Mesa 2006	314	45				5	26	7	18		1.8%	3.8%	
Romano 2006	86	30§					7						
Zhang 2012	69	7	0										
Jiang 2014	71	30§					0		3			4.1%	
Li 2017	56	30§				1				0	1.7%		0%
Tsamalaidze 2018	86	30					2						

Hernandez 2020	2,220	30		23	75	95‡					6.1%		2.4%
**Splenectomy, elective, laparoscopic, benign**						0‡							
Delaitre 2002	209	30§			1	1‡		0	2		0.9%	0.7%	
Wang 2013	260	30§					7			5			1.9%
Zychowicz 2018	194	30§		1		4‡			4		3.1%	1.4%	3.2%
Tastaldi 2019	109	30	0	0	3	3	2	0	1		2.9%	0.7%	
**Splenectomy, elective, open, benign**													
Zhang 2012	69	7	0										
Jiang 2014	71	30§					0		3			4.1%	
Li 2017	56	30§				1				0	1.7%		0%
**Splenectomy, elective, open, malignant**						0‡							
Mesa 2006	314	45				5	26	7	18		1.7%	3.8%	

Cumulative baseline risks (risk in patients not receiving thromboprophylaxis) are given for the first four postoperative weeks, adjusted for follow-up time and thromboprophylaxis use.
Blank spaces represent no information (not provided by paper or by author correspondence).
§Follow up time of complications was not available from the article or author correspondence. We assumed a follow up time of 30 days as this was median reported follow up time in the eligible studies.
* Excluding SVT
† Authors provided value.
‡ Estimated VTE value

6. Peri- and intraoperative risk of symptomatic VTE and bleeding in individual studies in general abdominal surgery

Reference	Total patients	Peri-operative bleeding	Reported intra-operative bleeding		
	n	Peri-operative bleeding requiring transfusion	Fatal intra-operative bleeding	Intra-operative bleeding requiring conversion to open	Intra-operative bleeding requiring transfusion
**Appendectomy, laparoscopic**					
Nguyen 2007	24509				
Hemmila 2010	15445	5			
Brugger 2011	7446				
Alizadeh 2017	168963				
Chung 2019	52767				
Garcia 2019	83712				
**Appendectomy, open**					
Nguyen 2007	25554				
Hemmila 2010	6030	1			
Chung 2019	193845				
Garcia 2019	12665				
**Appendectomy, laparoscopic, emergency**					
Brugger 2011	7446				
Sakran 2019	65017				
**Appendectomy, open, emergency**					

Sakran 2019	6292				
**Cholecystectomy, conversion to open**					
Persson 2012	3768				
**Cholecystectomy, laparoscopic**					
Blake 2001	587				
Schaepkens Van Riempst 2002	238				
Engbaek 2006	258				
Lindberg 2006	50				
Nguyen 2007	50527				
Rathore 2007	164				
Triantafyllidis 2009	1009				
Ingraham 2010	58659	44			
Ntourakis 2011	119				
Hasbahceci 2012	1557				
Pakaneh 2012	100				
Persson 2012	42271				
Stein 2014	4107430				
Suuronen 2015	17175	223			
Donkervoort 2016	4359				
Ulrych 2016	90		0	0	
Gundogdu 2017	1485		0	9	
Rosero 2017	230745				
Sepassi 2018	518				
Coelho 2019	1645				
Rysmakhanov 2019	1658			8	

Ross 2020	256726				
**Cholecystectomy, laparoscopic, elective**					
Schaepkens Van Riempst 2002	238				
Rathore 2007	164				
Ntourakis 2011	119				
Ulrych 2016	90		0	0	
Gundogdu 2017	1485		0	9	
Sepassi 2018	518				
**Cholecystectomy, laparoscopic, emergency**					
Sakran 2019	11266				
Cholecystectomy, open			0	0	
Nguyen 2007	14513				
Ingraham 2010	6852	37			
Persson 2012	4370				
Suuronen 2015	4942	642			
Sakran 2019	1447				
Ross 2020	37311				
**Cholecystectomy, open, emergency**					
Sakran 2019	1447				
**Hernia repair, groin, laparoscopic**					
Al-Sahaf 2008	108			0	
Srsen 2008	82				
Meyer 2013	4565				
Wakasugi 2016	365				
Wakasugi 2017	350				
Mita 2020	413				
Perez 2020	5282				

Wang 2020	7110			
Yang 2019	144			
**Hernia repair, groin, open**				
Holzheimer 2007	300			
Srsen 2008	134			
Bessa 2015	234			
Lozano 2015	218			
Nilsson 2016	140567			
Tastaldi 2019	257			
Liu 2020	146			
Perez 2020	36575			
Poudel 2020	4870	0		0
Wang 2020	6776			
**Hernia repair, groin, minimally-invasive, elective**				
Srsen 2008	82			
Meyer 2013	4565			
Mita 2020	413			
Yang 2019	144			
**Hernia repair, groin, open, elective**				
Srsen 2008	134			
Lozano 2015	218			
Nilsson 2016	132801			
**Hernia repair, groin, open, emergency**				
Bessa 2015	234			
Nilsson 2016	7766			
Tastaldi 2019	257			
Liu 2020	146			
**Hernia repair, ventral, laparoscopic**				
Lomanto 2006	50	0	0	0

Ferrari 2008	100			0	
Sharma 2011	1242				
Aher 2015	26286	27			
Warren 2017	103				
Boules 2018	361				
Ross 2020	33630				
Zolin 2020	81				
**Hernia repair, ventral, open**					
Schmidbauer 2005	175				
Aher 2015	90721	153			
Basta 2016	142				
Ulrych 2016	126		0	0	
Bittner 2018	76				
Kraft 2019	175				
Ross 2020	128513				
Zolin 2020	105				
**Hernia repair, ventral, robotic**					
Warren 2017	53				
**Hernia repair, ventral, laparoscopic, elective**					
Lomanto 2006	50		0	0	0
Aher 2015	26286	27			
Boules 2018	361				
Zolin 2020	81				
**Hernia repair, ventral, laparoscopic, emergency**					

Sakran 2019	405			
**Hernia repair, ventral, open, elective**				
Aher 2015	90721	153		
Ulrych 2016	126		0	0
Bittner 2018	76			
Kraft 2019	175			
Zolin 2020	105			
**Hernia repair, ventral, open, emergency**				
Sakran 2019	4808			
**Small bowel resection, laparoscopic**				
Daly 2014	1780	98		
McKenna 2018	1415			
**Small bowel resection, open**				
Daly 2014	17701	2832		
McKenna 2018	3592			
Sakran 2019	6855			
**Small bowel resection, laparoscopic, malignant**				
McKenna 2018	499			
**Small bowel resection, laparoscopic, IBD**				
McKenna 2018	443			
**Small bowel resection, laparoscopic, benign**				
McKenna 2018	355			
**Small bowel resection, laparoscopic, emergency**				
McKenna 2018	118			
**Small bowel resection, open, IBD**				

McKenna 2018	1237				
**Small bowel resection, open, benign**					
McKenna 2018	571				
**Small bowel resection, open, malignant**					
McKenna 2018	1784				
**Small bowel resection, open, emergency**					
Sakran 2019	6855				
**Splenectomy, elective, laparoscopic**					
Delaitre 2002	209			25	55
Patel 2003	108			9	
Romano 2006	72				
Casaccia 2010	676			21	
Vecchio 2011	107			3	
Corcione 2012	300			2	9
Wang 2013	260				
Radkowiak 2018	500	42		5	
Tsamalaidze 2018	101				
Zychowicz 2018	194	14		2	
Tastaldi 2019	109				
Hernandez 2020	4365	80			
**Splenectomy, elective, open**					
Mesa 2006	314				
Romano 2006	86				
Zhang 2012	69				

Jiang 2014	71				
Li 2017	56				
Tsamalaidze 2018	86				
Hernandez 2020	2220	115			
**Splenectomy, elective, laparoscopic, benign**					
Delaitre 2002	209			25	55
Wang 2013	260				
Zychowicz 2018	194	14		2	
Tastaldi 2019	109				
**Splenectomy, elective, open, benign**					
Zhang 2012	69				
Jiang 2014	71				
Li 2017	56				
**Splenectomy, elective, open, malignant**					
Mesa 2006	314				

Blank spaces represent no information (not provided by paper or by author correspondence).

5. Colorectal surgery supplementary tables 7-11

7. Characteristics of individual studies in colorectal surgery

Reference	Year	Country/ Countries	Patients(n)	Age Mean (SD)*	Female (%)	Malignancy (%)	Length of stay (Days)	Recruitment First year	Recruitment Last year	Study type
**Abdominoperineal resection, laparoscopic**										
Tooley	2018	USA	2574		42	85	7	2011	2015	Multicenter in one country
**Abdominoperineal resection, open**										
Tooley	2018	USA	5107		42	80	10	2011	2015	Multicenter in one country
**Anterior resection, laparoscopic**										
Law	2006	Hong Kong	98	69†	31	100	7†	2000	2004	One center, multiple surgeons
Park	2011	Japan,Korea	130	61 (12)	32	100	13	1997	2009	Multinational
Liang	2013	Taiwan	263	62 (13)	48	100		2005	2012	One center, multiple surgeons
Osborne	2013	UK	382	70 (15)	64	45		2001	2011	Single surgeon series
Cuccurullo§	2015	Italy	356	65 (1)		1	8†	2003	2012	One center, multiple surgeons
Lacy	2015	Spain	140	66 (13)	36	100	6†	2011	2014	One center, multiple surgeons
Park	2015	Korea	84	64 (11)	29	100	7	2006	2011	Single surgeon series
Tuech	2015	France	56	65†	27	100	10†	2010	2012	Multicenter in one country
Law	2017	China	171	67†	43	100	6†	2008	2015	One center, multiple surgeons
Miyagaki	2017	USA	6137			100		2012	2014	Multicenter in one country
McKenna§	2018	USA	33846	59 (18)†	50	46		2005	2016	Multicenter in one country

**Anterior resection, open**										
Law	2006	Hong Kong	167	70†	33	100	8†	2000	2004	One center, multiple surgeons
Park	2011	Japan,Korea	80	59 (11)	34	100	18	1997	2009	Multinational
Kang	2013	USA	72055	63 (14)	45	100	7	2006	2009	Multicenter in one country
McKenna§	2018	USA	21291	62 (18)†	49	96		2005	2016	Multicenter in one country
Lee	2019	USA	2521	64 (20)†	1		10†	2012	2016	Multicenter in one country
**Anterior resection, robotic**										
Park	2015	Korea	133	59 (11)	35	100	6	2006	2011	Single surgeon series
Law	2017	China	220	65†	33	100	6†	2008	2015	One center, multiple surgeons
**Colectomy, laparoscopic**										
Yamamoto	2004	Japan	120	61 (15)	41	100	8†	2001	2003	One center, multiple surgeons
Alves§	2005	France	163	58			10	2002	2002	Multicenter in one country
Leroy§	2005	France	111	62 (12)	46	46	10	2001	2003	One center, multiple surgeons
Bilimoria	2008	USA	837	70 (19)†	52	100	6	2005	2006	Multicenter in one country
Chan	2008	Hong Kong	429	69	45	100	6†	2000	2006	One center, multiple surgeons
Garrett	2008	USA	200	55	54		5	2001	2007	One center, multiple surgeons
Umanskiy	2010	USA	55	40 (14)	64	4	6†	2002	2008	One center, multiple surgeons
Abarca	2011	USA	358	56 (20)	47	56		2004	2009	One center, multiple surgeons
Kronberg	2011	Ireland	413	58 (15)	54	45		2004	2008	One center, multiple surgeons
Masoomi	2011	USA	14562	55	53		5	2002	2007	Multicenter in one country
Henke	2012	USA	1292	65 (15)	53			2008	2009	Multicenter in one country
Tyler	2012	USA	2423		51	43	6	2008	2009	Multicenter in one country
Causey	2013	USA	112	45 (17)	45			2005	2008	Multicenter in one country
Gu	2013	USA	204	35†	49		6	1998	2010	One center, multiple surgeons
Magistro	2013	Italy	80	71 (12)	53	100	6	2009	2011	One center, multiple surgeons
Cuccurullo§	2015	Italy	845	65 (3)	37	92		2003	2012	One center, multiple surgeons

Li	2015	USA	159	36 (14)	57		6	2000	2012	One center, multiple surgeons
Miller	2016	USA	11267	60	52		6	2013	2013	Multicenter in one country
Wright	2016	USA	10853			100		2009	2013	Multicenter in one country
Denet	2017	France	507	69†	48	107	7†	2004	2014	One center, multiple surgeons
Ilyas	2017	USA	3946			50	5	2004	2011	Multicenter in one country
Franco	2018	France	473	73†	47	100		2005	2015	Multicenter in one country
Posabella	2018	Switzerland	1016	64†	28	0		2004	2014	One center, multiple surgeons
McKenna§	2018	USA	71411	62 (19)†	52	60		2005	2016	Multicenter in one country
Sakran	2019	USA	388	50 (17)	51			2013	2015	Multicenter in one country
Ross	2020	USA	62366	55 (17)	44			2005	2016	Multicenter in one country
Krimphove	2020	USA,UK,Germany,Italy	4177		50	100		2012	2017	Multinational

Colectomy, laparoscopic, benign

Alves§	2005	France	163	58			10	2002	2002	Multicenter in one country
Garrett	2008	USA	200	55	54		5	2001	2007	One center, multiple surgeons
Masoomi	2011	USA	14562	55	53		5	2002	2007	Multicenter in one country
Ilyas	2017	USA	1973			0		2004	2011	Multicenter in one country
McKenna§	2018	USA	37004	57 (17)†	53	0		2005	2016	Multicenter in one country
Posabella	2018	Switzerland	1016	64†	28	0		2004	2014	One center, multiple surgeons
Althans	2019	USA	397	65 (17)	56		6	2012	2015	Multicenter in one country

Colectomy, laparoscopic, emergency

McKenna§	2018	USA	1953	64 (29)†	51	50		2005	2016	Multicenter in one country
Sakran	2019	USA	388	50 (17)	51			2013	2015	Multicenter in one country

Colectomy, laparoscopic, IBD

Umanskiy	2010	USA	55	40 (14)	64	4	6†	2002	2008	One center, multiple surgeons
Causey	2013	USA	112	45 (17)	45			2005	2008	Multicenter in one country
Gu	2013	USA	204	35†	49		6	1998	2010	One center, multiple surgeons

Li	2015	USA	159	36 (14)	57		6	2000	2012	One center, multiple surgeons
McKenna§	2018	USA	8588	36 (23)†	54	0		2005	2016	Multicenter in one country
**Colectomy, laparoscopic, malignant**										
Yamamoto	2004	Japan	120	61 (15)	41	100	8†	2001	2003	One center, multiple surgeons
Bilimoria	2008	USA	837	70 (19)†	52	100	6	2005	2006	Multicenter in one country
Chan	2008	Hong Kong	429	69	45	100	6	2000	2006	One center, multiple surgeons
Magistro	2013	Italy	80	71 (12)	53	100		2009	2011	One center, multiple surgeons
Wright	2016	USA	10853			100		2009	2013	Multicenter in one country
Denet	2017	France	507	69†	48	107	7†	2004	2014	One center, multiple surgeons
Franco	2018	France	473	73†	47	100		2005	2015	Multicenter in one country
Haskins	2018	USA	2405	68 (13)	53	100	5	2012	2014	Multicenter in one country
McKenna§	2018	USA	42160	69 (19)†	52	100		2005	2016	Multicenter in one country
Iwamoto	2019	Japan	390	67 (11)	45	100		2010	2016	One center, multiple surgeons
**Colectomy, sigmoid, laparoscopic**										
Alves§	2005	France	163	58			10	2002	2002	Multicenter in one country
Garrett	2008	USA	200	55	54		5	2001	2007	One center, multiple surgeons
Ilyas	2017	USA	3946			50	5	2004	2011	Multicenter in one country
Posabella	2018	Switzerland	1016	64†	28	0		2004	2014	One center, multiple surgeons
**Colectomy, left, laparoscopic**										
Leroy§	2005	France	111	62 (12)	46	46	10	2001	2003	One center, multiple surgeons
Henke	2012	USA	897	65 (15)	53			2008	2009	Multicenter in one country
Cuccurullo	2015	Italy	585	67 (3)		1		2003	2012	One center, multiple surgeons
Mrdutt	2017	USA	35079				4†	2011	2014	Multicenter in one country
McKenna§	2018	USA	47488	63 (18)†	52	57		2005	2016	Multicenter in one country
**Colectomy, right, laparoscopic**										
Henke	2012	USA	395	65 (15)	53			2008	2009	Multicenter in one country
Magistro	2013	Italy	80	71 (12)	53	100	6	2009	2011	One center, multiple surgeons

Cuccurullo	2015	Italy	260	62 (3)		1		2003	2012	One center, multiple surgeons
Li	2015	USA	159	36 (14)	57		6	2000	2012	One center, multiple surgeons
Denet	2017	France	507	69†	48	107	7†	2004	2014	One center, multiple surgeons
Mrdutt	2017	USA	8488					2011	2014	Multicenter in one country
Franco	2018	France	473	73†	47	100		2005	2015	Multicenter in one country
McKenna§	2018	USA	19768	62 (19)†	54	2		2005	2016	Multicenter in one country
**Colectomy, open**										
Alves§	2005	France	169	63			18	2002	2002	Multicenter in one country
Bilimoria	2008	USA	2222	68 (21)†	49	100	9	2005	2006	Multicenter in one country
Umanskiy	2010	USA	70	41 (16)	53		8†	2002	2008	One center, multiple surgeons
Masoomi	2011	USA	110172	57	53		7	2002	2007	Multicenter in one country
Henke	2012	USA	2172	65 (15)	53			2008	2009	Multicenter in one country
Causey	2013	USA	338	44 (15)	27			2005	2008	Multicenter in one country
Li	2015	USA	159	36 (14)	57		6	2000	2012	One center, multiple surgeons
Wright	2016	USA	29215			100		2009	2013	Multicenter in one country
Ilyas	2017	USA	17252			11	7	2004	2011	Multicenter in one country
Haskins	2018	USA	1024	71 (12)	56	100	8	2012	2014	Multicenter in one country
McKenna§	2018	USA	5355	59 (23)†	46	33		2005	2016	Multicenter in one country
Althans	2019	USA	1778	65 (17)	56		8	2012	2015	Multicenter in one country
Sakran	2019	USA	9822	50 (17)	51			2013	2015	Multicenter in one country
Krimphove	2020	USA,UK,Germany,Italy	2795		51	100		2012	2017	Multinational
Ross	2020	USA	98994	55 (17)	44			2005	2016	Multicenter in one country
Weber	2020	USA	2019	61 (14)	51		10†	2005	2015	Multicenter in one country
**Colectomy, open, benign**										
Alves§	2005	France	169	63			18	2002	2002	Multicenter in one country
Masoomi	2011	USA	110172	57	53		7	2002	2007	Multicenter in one country
Ilyas	2017	USA	8626			0	7	2004	2011	Multicenter in one country
McKenna§	2018	USA	30442	62 (14)	55	0		2005	2016	Multicenter in one country

Althans	2019	USA	1778	65 (17)	56		8	2012	2015	Multicenter in one country
**Colectomy, open, emergency**										
McKenna§	2018	USA	18033	65 (17)	50	22		2005	2016	Multicenter in one country
Sakran	2019	USA	9822	50 (17)	51			2013	2015	Multicenter in one country
Weber	2020	USA	2019	61 (14)	51			2005	2015	Multicenter in one country
**Colectomy, open, IBD**										
Umanskiy	2010	USA	70	41 (16)	53		8†	2002	2008	One center, multiple surgeons
Causey	2013	USA	338	44 (15)	27			2005	2008	Multicenter in one country
Li	2015	USA	159	36 (14)	57		6	2000	2012	One center, multiple surgeons
McKenna§	2018	USA	8058	43 (18)	50	0		2005	2016	Multicenter in one country
**Colectomy, open, malignant**										
Bilimoria	2008	USA	2222	68 (21)†	49	100	9	2005	2006	Multicenter in one country
Wright	2016	USA	29215			100		2009	2013	Multicenter in one country
Ilyas	2017	USA	8626			100		2004	2011	Multicenter in one country
Haskins	2018	USA	1024	71 (12)	56	100	8	2012	2014	Multicenter in one country
McKenna§	2018	USA	42007	70 (15)	51	100		2005	2016	Multicenter in one country
Krimphove	2020	USA,UK,Germany,Italy	2795		51	100		2012	2017	Multinational
**Colectomy, sigmoid, open**										
Alves§	2005	France	169	63			18	2002	2002	Multicenter in one country
Ilyas	2017	USA	17252			11	7	2004	2011	Multicenter in one country
McKenna§	2018	USA	8270	60 (17)†	58	100		2005	2016	Multicenter in one country
**Colectomy, left, open**										
Henke	2012	USA	1334	65 (15)	53			2008	2009	Multicenter in one country
McKenna§	2018	USA	21269	64 (15)	53	5		2005	2016	Multicenter in one country
**Colectomy, right, open**										
Henke	2012	USA	838	65 (15)	53			2008	2009	Multicenter in one country
Haskins	2018	USA	1024	71 (12)	56	100	8	2012	2014	Multicenter in one country
McKenna§	2018	USA	19812	65 (16)	53	7		2005	2016	Multicenter in one country

**Colectomy, robotic**										
Tyler	2012	USA	160		50	36	6	2008	2009	Multicenter in one country
Miller	2016	USA	653	60	52		5	2013	2013	Multicenter in one country
Haskins	2018	USA	89	69 (12)	45	100	4	2012	2014	Multicenter in one country
Raskin	2019	USA	108	43 (17)	66		2†	2011	2015	Multicenter in one country
**Colectomy, robotic, IBD**										
Raskin	2019	USA	108	43 (17)	66		2†	2011	2015	Multicenter in one country
**Colectomy, robotic, malignant**										
Haskins	2018	USA	89	69 (12)	45	100	4	2012	2014	Multicenter in one country
**Colectomy, right, robotic**										
Haskins	2018	USA	89	69 (12)	45	100	4	2012	2014	Multicenter in one country
Raskin	2019	USA	108	43 (17)	66		2†	2011	2015	Multicenter in one country
**Proctocolectomy, laparoscopic**										
Causey	2013	USA	148	40 (14)	45			2005	2008	Multicenter in one country
Gu	2013	USA	204	35†	49		6	1998	2010	One center, multiple surgeons
Gu	2016	USA	248	39 (13)	0	0	15	2006	2012	One center, multiple surgeons
Duraes	2018	USA	119	37 (15)	45	0	8	1998	2014	One center, multiple surgeons
McKenna§	2018	USA	4155	44 (25)†	47	23		2005	2016	Multicenter in one country
**Proctocolectomy, open**										
Remzi	2002	USA	702			0		1997	2000	One center, multiple surgeons
Causey	2013	USA	517	44 (14)	45			2005	2008	Multicenter in one country
Ryoo	2014	Korea	72	43 (22)†	61	10		1998	2013	Single surgeon series
Gu	2016	USA	273	39 (13)	0	0	15	2006	2012	One center, multiple surgeons
McKenna§	2018	USA	8180	59 (17)	46	33		2005	2016	Multicenter in one country
**Proctocolectomy, laparoscopic, benign**										
Duraes	2018	USA	119	37 (15)	45	0	8	1998	2014	One center, multiple surgeons
McKenna§	2018	USA	238	44 (25)†	47	0		2005	2016	Multicenter in one country

**Proctocolectomy, laparoscopic, IBD**										
Causey	2013	USA	148	40 (14)	45			2005	2008	Multicenter in one country
Gu	2016	USA	248	39 (13)	0	0	15	2006	2012	One center, multiple surgeons
McKenna§	2018	USA	4055	44 (25)†	47	0		2005	2016	Multicenter in one country
**Proctocolectomy, laparoscopic, malignant**										
McKenna§	2018	USA	1307	61 (19)†	43	100		2005	2016	Multicenter in one country
**Proctocolectomy, open, benign**										
McKenna§	2018	USA	708	67 (19)†	60	0		2005	2016	Multicenter in one country
**Proctocolectomy, open, emergency**										
McKenna§	2018	USA	1932	68 (25)†	56	16		2005	2016	Multicenter in one country
**Proctocolectomy, open, IBD**										
Remzi	2002	USA	702			0		1997	2000	One center, multiple surgeons
Causey	2013	USA	397	44 (14)	45			2005	2008	Multicenter in one country
Ryoo	2014	Korea	72	43 (22)†	61	10		1998	2013	Single surgeon series
Gu	2016	USA	273	39 (13)	0	0	15	2006	2012	One center, multiple surgeons
McKenna§	2018	USA	3130	54 (23)†	55	0		2005	2016	Multicenter in one country
**Proctocolectomy, open, malignant**										
McKenna§	2018	USA	2410	62 (21)†	38	100		2005	2016	Multicenter in one country
**Rectopexy, laparoscopic**										
Vogel	2020	USA	3350	61†	90			2005	2017	Multicenter in one country
**Rectopexy, open**										
Vogel	2020	USA	3599	64†	91			2005	2017	Multicenter in one country
**Rectopexy, perineal**										
Kimmins	2001	USA	63	79 (15)	98			1993	1999	One center, multiple surgeons
Altomare	2009	Italy	93	77†	88		6†	1998	2006	Multicenter in one country
Ding	2012	USA	113	80 (17)	96		5	2000	2009	One center, multiple surgeons

Vogel	2020	USA	5271	80†	94		2005	2017	Multicenter in one country

Blank spaces indicate an absence of information.

Articles are reported by procedure, so duplicate information from same study appears in this table.

Many articles reported on more than one procedure (For instance Masoomi 2011 reported on colectomy, laparoscopic; colectomy, laparoscopic, benign; colectomy, open; and colectomy, open, benign).

*Age is reported as mean (SD) unless otherwise indicated

† Median (IQR)

§ Authors confirmed accuracy of our consensus data extraction and/or corrected some errors or provided additional information

Causey 2013: Laparoscopic and open colectomies: Proportion of females was provided for all colectomies combined, age was provided for laparoscopic and open combined.
Henke 2012: Laparoscopic left and right and open left and right colectomies: Age and proportion of females was provided for all colectomies combined.
Li 2015: Laparoscopic and open colectomies: Age and proportion of females was provided for all colectomies combined.
Althans 2019: Laparoscopic and open colectomies: Age and proportion of females was provided for all colectomies combined.
Sakran 2019: Laparoscopic and open colectomies: Age and proportion of females was provided for two groups: <100min procedure and >100min procedure.

Studies where some outcomes were excluded from baseline risk analyses for a procedure (but not all outcomes):
Miyagaki 2017,Anterior resection, laparoscopic: We did not include the study to the baseline risk analyses for VTE to because of overlapping population
Miller 2016, Colectomy, laparoscopic: We did not include the study to the baseline risk analyses for VTE because of overlapping population
Haskins 2018, Colectomy, open: We did not include the study to the baseline risk analyses for VTE because of overlapping population.
Haskins 2018, Colectomy, laparoscopic: We did not include the study to the baseline risk analyses for VTE and bleeding leading to transfusion because of overlapping population.
Haskins 2018, Colectomy, laparoscopic, malignant: We did not include the study to the baseline risk analyses for VTE because of overlapping population.
Althans 2019: Colectomy, laparoscopic: We did not include the study to the baseline risk analyses for VTE and bleeding leading to transfusion because of overlapping population.
Althans 2019: Colectomy, laparoscopic, benign: We did not include the study to the baseline risk analyses for VTE because of overlapping population.
Causey 2013: Colectomy, laparoscopic: We did not include the study to the baseline risk analyses for VTE because of overlapping population.
Causey 2013: Proctocolectomy, open: We did not include the study to the baseline risk analyses for VTE because of overlapping population.
BIlimoria 2008: Colectomy, laparoscopic and open: We did not include the study to the baseline risk analyses for VTE because of overlapping population.
Mrdutt 2017: Colectomy, laparoscopic, left and right: We did not include the study to the baseline risk analyses for VTE because of overlapping population.

8. Risk of bias in individual studies in colorectal surgery

Reference	Sampling	Thromboprophylaxis documentation	Source of information	Recruitment years	Specification of length of follow-up	Study type	Risk of Bias
**Abdominoperineal resection, laparoscopic**							
Tooley 2018	+	-	Prospective data collection	+	+	Multicenter in one country	LOW
**Abdominoperineal resection, open**							
Tooley 2018	+	-	Prospective data collection	+	+	Multicenter in one country	LOW
**Anterior resection, laparoscopic**							
Law 2006	+	-	Prospective data collection	-	-	One center, multiple surgeons	HIGH
Park 2011	+	-	Retrospective chart reviews, data collected by one investigator	-	-	Multinational	HIGH
Liang 2013	+	-	Retrospective chart reviews, data collected by one investigator	-	-	One center, multiple surgeons	HIGH
Osborne 2013	+	-	Prospective data collection	-	-	Single surgeon series	HIGH
Cuccurullo 2015	+	-	Retrospective chart reviews, data collected by one investigator	-	+	One center, multiple surgeons	HIGH
Lacy 2015	+	-	Prospective data collection	+	+	One center, multiple surgeons	LOW
Park 2015	+	-	Prospective data collection	-	+	Single surgeon series	HIGH
Tuech 2015	+	-	Prospective data collection	+	-	Multicenter in one country	MODERATE
Law 2017	+	-	Prospective data collection	+	-	One center, multiple surgeons	MODERATE
Miyagaki 2017	-	-	Prospective data collection	+	-	Multicenter in one country	HIGH
McKenna 2018	+	-	Prospective data collection	+	+	Multicenter in one country	LOW
**Anterior resection, open**							
Law 2006	+	-	Prospective data collection	-	-	One center, multiple surgeons	HIGH

Park 2011	+	-	Retrospective chart reviews, data collected by one investigator	-	-	Multinational	HIGH
Kang 2013	+	-	Administrative database information	-	-	Multicenter in one country	HIGH
McKenna 2018	+	-	Prospective data collection	+	+	Multicenter in one country	LOW
Lee 2019	+	-	Prospective data collection	+	+	Multicenter in one country	LOW
**Anterior resection, robotic**							
Park 2015	+	-	Prospective data collection	-	+	Single surgeon series	HIGH
Law 2017	+	-	Prospective data collection	+	-	One center, multiple surgeons	MODERATE
**Colectomy, laparoscopic**							
Yamamoto 2004	+	-	Retrospective chart reviews, data collected by one investigator	-	+	One center, multiple surgeons	HIGH
Alves 2005	+	+	Prospective data collection	-	-	Multicenter in one country	MODERATE
Leroy 2005	+	+	Retrospective chart reviews, data collected by one investigator	-	+	One center, multiple surgeons	MODERATE
Bilimoria 2008	+	-	Prospective data collection	-	+	Multicenter in one country	MODERATE
Chan 2008	+	-	Prospective data collection	-	-	One center, multiple surgeons	HIGH
Garrett 2008	+	-	Retrospective chart reviews, data collected by one investigator	-	+	One center, multiple surgeons	HIGH
Umanskiy 2010	+	-	Retrospective chart reviews, data collected by one investigator	-	-	One center, multiple surgeons	HIGH
Abarca 2011	+	-	Administrative database information	-	-	One center, multiple surgeons	HIGH
Kronberg 2011	+	-	Prospective data collection	-	-	One center, multiple surgeons	HIGH
Masoomi 2011	-	-	Administrative database information	-	-	Multicenter in one country	HIGH
Henke 2012	+	-	Prospective data collection	-	+	Multicenter in one country	MODERATE
Tyler 2012	-	-	Administrative database information	-	-	Multicenter in one country	HIGH
Causey 2013	+	-	Prospective data collection	-	+	Multicenter in one country	MODERATE
Gu 2013	+	-	Administrative database information	-	+	One center, multiple surgeons	HIGH
Magistro 2013	+	+	Prospective data collection	+	-	One center, multiple surgeons	LOW
Cuccurullo 2015	+	-	Retrospective chart reviews, data collected by one investigator	-	+	One center, multiple surgeons	HIGH
Li 2015	+	-	Retrospective chart reviews, data collected by one investigator	-	+	One center, multiple surgeons	HIGH

Miller 2016	+	-	Prospective data collection	+	+	Multicenter in one country	LOW
Wright 2016	-	+	Administrative database information	+	+	Multicenter in one country	MODERATE
Denet 2017	-	-	Prospective data collection	-	+	One center, multiple surgeons	HIGH
Ilyas 2017	+	-	Administrative database information	-	-	Multicenter in one country	HIGH
Franco 2018	+	-	Retrospective chart reviews, data collected by one investigator	+	+	Multicenter in one country	MODERATE
Posabella 2018	+	+	Prospective data collection	-	+	One center, multiple surgeons	LOW
McKenna 2018	+	-	Prospective data collection	+	+	Multicenter in one country	LOW
Sakran 2019	+	-	Prospective data collection	+	+	Multicenter in one country	LOW
Ross 2020	+	-	Prospective data collection	+	+	Multicenter in one country	LOW
Krimphove 2020	+	-	Administrative database information	+	+	Multinational	MODERATE
**Colectomy, laparoscopic, benign**							
Alves 2005	+	+	Prospective data collection	-	-	Multicenter in one country	MODERATE
Garrett 2008	+	-	Retrospective chart reviews, data collected by one investigator	-	+	One center, multiple surgeons	HIGH
Masoomi 2011	-	-	Administrative database information	-	-	Multicenter in one country	HIGH
Ilyas 2017	+	-	Administrative database information	-	-	Multicenter in one country	HIGH
McKenna 2018	+	-	Prospective data collection	+	+	Multicenter in one country	LOW
Posabella 2018	+	+	Prospective data collection	-	+	One center, multiple surgeons	LOW
Althans 2019	+	-	Prospective data collection	+	+	Multicenter in one country	LOW
**Colectomy, laparoscopic, emergency**							
McKenna 2018	+	-	Prospective data collection	+	+	Multicenter in one country	LOW
Sakran 2019	+	-	Prospective data collection	+	+	Multicenter in one country	LOW
**Colectomy, laparoscopic, IBD**							
Umanskiy 2010	+	-	Retrospective chart reviews, data collected by one investigator	-	-	One center, multiple surgeons	HIGH
Causey 2013	+	-	Prospective data collection	-	+	Multicenter in one country	MODERATE
Gu 2013	+	-	Administrative database information	-	+	One center, multiple surgeons	HIGH

Li 2015	+	-	Retrospective chart reviews, data collected by one investigator	-	+	One center, multiple surgeons	HIGH
McKenna 2018	+	-	Prospective data collection	+	+	Multicenter in one country	LOW
**Colectomy, laparoscopic, malignant**							
Yamamoto 2004	+	-	Retrospective chart reviews, data collected by one investigator	-	+	One center, multiple surgeons	HIGH
Bilimoria 2008	+	-	Prospective data collection	-	+	Multicenter in one country	MODERATE
Chan 2008	+	-	Prospective data collection	-	-	One center, multiple surgeons	HIGH
Magistro 2013	+	+	Prospective data collection	+	-	One center, multiple surgeons	LOW
Wright 2016	-	+	Administrative database information	+	+	Multicenter in one country	MODERATE
Denet 2017	-	-	Prospective data collection	-	+	One center, multiple surgeons	HIGH
Franco 2018	+	-	Retrospective chart reviews, data collected by one investigator	+	+	Multicenter in one country	MODERATE
Haskins 2018	+	-	Prospective data collection	+	+	Multicenter in one country	LOW
McKenna 2018	+	-	Prospective data collection	+	+	Multicenter in one country	LOW
Iwamoto 2019	+	+	Retrospective chart reviews, data collected by one investigator	+	-	One center, multiple surgeons	MODERATE
**Colectomy, sigmoid, laparoscopic**							
Alves 2005	+	+	Prospective data collection	-	-	Multicenter in one country	MODERATE
Garrett 2008	+	-	Retrospective chart reviews, data collected by one investigator	-	+	One center, multiple surgeons	HIGH
Ilyas 2017	+	-	Administrative database information	-	-	Multicenter in one country	HIGH
Posabella 2018	+	+	Prospective data collection	-	+	One center, multiple surgeons	LOW
**Colectomy, left, laparoscopic**							
Leroy 2005	+	+	Retrospective chart reviews, data collected by one investigator	-	+	One center, multiple surgeons	MODERATE
Henke 2012	+	-	Prospective data collection	-	+	Multicenter in one country	MODERATE
Cuccurullo 2015	+	-	Retrospective chart reviews, data collected by one investigator	-	+	One center, multiple surgeons	HIGH
Mrdutt 2017	-	-	Prospective data collection	+	+	Multicenter in one country	MODERATE
McKenna 2018	+	-	Prospective data collection	+	+	Multicenter in one country	LOW
**Colectomy, right, laparoscopic**							

Henke 2012	+	-	Prospective data collection	-	+	Multicenter in one country	MODERATE
Magistro 2013	+	+	Prospective data collection	+	-	One center, multiple surgeons	LOW
Cuccurullo 2015	+	-	Retrospective chart reviews, data collected by one investigator	-	+	One center, multiple surgeons	HIGH
Li 2015	+	-	Retrospective chart reviews, data collected by one investigator	-	+	One center, multiple surgeons	HIGH
Denet 2017	-	-	Prospective data collection	-	+	One center, multiple surgeons	HIGH
Mrdutt 2017	-	-	Prospective data collection	+	+	Multicenter in one country	MODERATE
Franco 2018	+	-	Retrospective chart reviews, data collected by one investigator	+	+	Multicenter in one country	MODERATE
McKenna 2018	+	-	Prospective data collection	+	+	Multicenter in one country	LOW
**Colectomy, open**							
Alves 2005	+	+	Prospective data collection	-	-	Multicenter in one country	MODERATE
Bilimoria 2008	+	-	Prospective data collection	-	+	Multicenter in one country	MODERATE
Umanskiy 2010	+	-	Retrospective chart reviews, data collected by one investigator	-	-	One center, multiple surgeons	HIGH
Masoomi 2011	-	-	Administrative database information	-	-	Multicenter in one country	HIGH
Henke 2012	+	-	Prospective data collection	-	+	Multicenter in one country	MODERATE
Causey 2013	+	-	Prospective data collection	-	+	Multicenter in one country	MODERATE
Li 2015	+	-	Retrospective chart reviews, data collected by one investigator	-	+	One center, multiple surgeons	HIGH
Wright 2016	-	+	Administrative database information	+	+	Multicenter in one country	MODERATE
Ilyas 2017	+	-	Administrative database information	-	-	Multicenter in one country	HIGH
Haskins 2018	+	-	Prospective data collection	+	+	Multicenter in one country	LOW
McKenna 2018	+	-	Prospective data collection	+	+	Multicenter in one country	LOW
Althans 2019	+	-	Prospective data collection	+	+	Multicenter in one country	LOW
Sakran 2019	+	-	Prospective data collection	+	+	Multicenter in one country	LOW
Krimphove 2020	+	-	Administrative database information	+	+	Multinational	MODERATE
Ross 2020	+	-	Prospective data collection	+	+	Multicenter in one country	LOW
Weber 2020	+	-	Prospective data collection	+	+	Multicenter in one country	LOW
**Colectomy, open, benign**							
Alves 2005	+	+	Prospective data collection	-	-	Multicenter in one country	MODERATE

Masoomi 2011	-	-	Administrative database information	-	-	Multicenter in one country	HIGH
Ilyas 2017	+	-	Administrative database information	-	-	Multicenter in one country	HIGH
McKenna 2018	+	-	Prospective data collection	+	+	Multicenter in one country	LOW
Althans 2019	+	-	Prospective data collection	+	+	Multicenter in one country	LOW
**Colectomy, open, emergency**							
McKenna 2018	+	-	Prospective data collection	+	+	Multicenter in one country	LOW
Sakran 2019	+	-	Prospective data collection	+	+	Multicenter in one country	LOW
Weber 2020	+	-	Prospective data collection	+	+	Multicenter in one country	LOW
**Colectomy, open, IBD**							
Umanskiy 2010	+	-	Retrospective chart reviews, data collected by one investigator	-	-	One center, multiple surgeons	HIGH
Causey 2013	+	-	Prospective data collection	-	+	Multicenter in one country	MODERATE
Li 2015	+	-	Retrospective chart reviews, data collected by one investigator	-	+	One center, multiple surgeons	HIGH
McKenna 2018	+	-	Prospective data collection	+	+	Multicenter in one country	LOW
**Colectomy, open, malignant**							
Bilimoria 2008	+	-	Prospective data collection	-	+	Multicenter in one country	MODERATE
Wright 2016	-	+	Administrative database information	+	+	Multicenter in one country	MODERATE
Ilyas 2017	+	-	Administrative database information	-	-	Multicenter in one country	HIGH
Haskins 2018	+	-	Prospective data collection	+	+	Multicenter in one country	LOW
McKenna 2018	+	-	Prospective data collection	+	+	Multicenter in one country	LOW
Krimphove 2020	+	-	Administrative database information	+	+	Multinational	MODERATE
**Colectomy, sigmoid, open**							
Alves 2005	+	+	Prospective data collection	-	-	Multicenter in one country	MODERATE
Ilyas 2017	+	-	Administrative database information	-	-	Multicenter in one country	HIGH
McKenna 2018	+	-	Prospective data collection	+	+	Multicenter in one country	LOW
**Colectomy, left, open**							
Henke 2012	+	-	Prospective data collection	-	+	Multicenter in one country	MODERATE
McKenna 2018	+	-	Prospective data collection	+	+	Multicenter in one country	LOW

**Colectomy, right, open**							
Henke 2012	+	-	Prospective data collection	-	+	Multicenter in one country	MODERATE
Haskins 2018	+	-	Prospective data collection	+	+	Multicenter in one country	LOW
McKenna 2018	+	-	Prospective data collection	+	+	Multicenter in one country	LOW
**Colectomy, robotic**							
Tyler 2012	-	-	Administrative database information	-	-	Multicenter in one country	HIGH
Miller 2016	+	-	Prospective data collection	+	+	Multicenter in one country	LOW
Haskins 2018	+	-	Prospective data collection	+	+	Multicenter in one country	LOW
Raskin 2019	+	-	Administrative database information	+	+	Multicenter in one country	MODERATE
**Colectomy, robotic, IBD**							
Raskin 2019	+	-	Administrative database information	+	+	Multicenter in one country	MODERATE
**Colectomy, robotic, malignant**							
Haskins 2018	+	-	Prospective data collection	+	+	Multicenter in one country	LOW
**Colectomy, right, robotic**							
Haskins 2018	+	-	Prospective data collection	+	+	Multicenter in one country	LOW
Raskin 2019	+	-	Administrative database information	+	+	Multicenter in one country	MODERATE
**Proctocolectomy, laparoscopic**							
Causey 2013	+	-	Prospective data collection	-	+	Multicenter in one country	MODERATE
Gu 2013	+	-	Administrative database information	-	+	One center, multiple surgeons	HIGH
Gu 2016	+	+	Retrospective chart reviews, data collected by one investigator	-	-	One center, multiple surgeons	HIGH
Duraes 2018	+	-	Retrospective chart reviews, data collected by one investigator	-	-	One center, multiple surgeons	HIGH
McKenna 2018	+	-	Prospective data collection	+	+	Multicenter in one country	LOW
**Proctocolectomy, open**							
Remzi 2002	+	-	Retrospective chart reviews, data collected by one investigator	-	+	One center, multiple surgeons	HIGH
Causey 2013	+	-	Prospective data collection	-	+	Multicenter in one country	MODERATE
Ryoo 2014	+	-	Retrospective chart reviews, data collected by one investigator	-	+	Single surgeon series	HIGH
Gu 2016	+	+	Retrospective chart reviews, data collected by one investigator	-	-	One center, multiple surgeons	HIGH

McKenna 2018	+	-	Prospective data collection	+	+	Multicenter in one country	LOW
**Proctocolectomy, laparoscopic, benign**							
Duraes 2018	+	-	Retrospective chart reviews, data collected by one investigator	-	-	One center, multiple surgeons	HIGH
McKenna 2018	+	-	Prospective data collection	+	+	Multicenter in one country	LOW
**Proctocolectomy, laparoscopic, IBD**							
Causey 2013	+	-	Prospective data collection	-	+	Multicenter in one country	MODERATE
Gu 2016	+	+	Retrospective chart reviews, data collected by one investigator	-	-	One center, multiple surgeons	HIGH
McKenna 2018	+	-	Prospective data collection	+	+	Multicenter in one country	LOW
**Proctocolectomy, laparoscopic, malignant**							
McKenna 2018	+	-	Prospective data collection	+	+	Multicenter in one country	LOW
**Proctocolectomy, open, benign**							
McKenna 2018	+	-	Prospective data collection	+	+	Multicenter in one country	LOW
**Proctocolectomy, open, emergency**							
McKenna 2018	+	-	Prospective data collection	+	+	Multicenter in one country	LOW
**Proctocolectomy, open, IBD**							
Remzi 2002	+	-	Retrospective chart reviews, data collected by one investigator	-	+	One center, multiple surgeons	HIGH
Causey 2013	+	-	Prospective data collection	-	+	Multicenter in one country	MODERATE
Ryoo 2014	+	-	Retrospective chart reviews, data collected by one investigator	-	+	Single surgeon series	HIGH
Gu 2016	+	+	Retrospective chart reviews, data collected by one investigator	-	-	One center, multiple surgeons	HIGH
McKenna 2018	+	-	Prospective data collection	+	+	Multicenter in one country	LOW
**Proctocolectomy, open, malignant**							
McKenna 2018	+	-	Prospective data collection	+	+	Multicenter in one country	LOW
**Rectopexy, laparoscopic**							
Vogel 2020	+	-	Prospective data collection	+	+	Multicenter in one country	LOW
**Rectopexy, open**							

Vogel 2020	+	-	Prospective data collection	+	+	Multicenter in one country	LOW
**Rectopexy, perineal**							
Kimmins 2001	+	-	Retrospective chart reviews, data collected by one investigator	-	-	One center, multiple surgeons	HIGH
Altomare 2009	+	+	Retrospective chart reviews, data collected by one investigator	-	-	Multicenter in one country	HIGH
Ding 2012	+	-	Prospective data collection	-	-	One center, multiple surgeons	HIGH
Vogel 2020	+	-	Prospective data collection	+	+	Multicenter in one country	LOW

Articles are reported by procedure, so duplicate information from same study appears in this table.

9. Prophylaxis in individual studies in colorectal surgery

Reference	Total patients	Mechanical prophylaxis			Antiplatelet drugs			Anticoagulants		
	n	%	Type	Duration in days	%	Type	Duration in days	%	Type	Duration in days
**Abdominoperineal resection, laparoscopic**										
Tooley 2018	2574									
**Abdominoperineal resection, open**										
Tooley 2018	5107									
**Anterior resection, laparoscopic**										
Law 2006	98									
Park 2011	130									
Liang 2013	263									
Osborne 2013	382									
Cuccurullo 2015	356	81†	GCS†	1†	73†	Plavix, Ticlid, Aggrastat,ASA†				
Lacy 2015	140									
Park 2015	84									
Tuech 2015	56									
Law 2017	171									
Miyagaki 2017	6137									
McKenna 2018	33846		unknown							
**Anterior resection, open**										

Law 2006	167								
Park 2011	80								
Kang 2013	72055								
McKenna 2018	21291		unknown						
Lee 2019	2521								
**Anterior resection, robotic**									
Park 2015	133								
Law 2017	220								
**Colectomy, laparoscopic**									
Yamamoto 2004	120								
Alves 2005	163						100†		30†
Leroy 2005	111	0†		0			100†	LMWH†	30†
Bilimoria 2008	837								
Chan 2008	429								
Garrett 2008	200								
Umanskiy 2010	55								
Abarca 2011	358								
Kronberg 2011	413	100	GCS				100	LMWH	
Masoomi 2011	14562								
Henke 2012	1292	13	SCD				81	UFH/LMWH	
Tyler 2012	2423								
Causey 2013	112								
Gu 2013	204								
Magistro 2013	80						100	LMWH	30
Cuccurullo 2015	845	72†	GCS†	1†	58†	Plavix, Ticlid, Aggrastat,ASA†			
Li 2015	159								

Miller 2016	11267				
Wright 2016	10853		1	LMWH	
Denet 2017	507				
Ilyas 2017	3946				
Franco 2018	473				
Posabella 2018	1016		100	LMWH	
McKenna 2018	71411	unknown			
Sakran 2019	388				
Ross 2020	62366				
Krimphove 2020	4177				

Colectomy, laparoscopic, benign

Alves 2005	163		100†		30†
Garrett 2008	200				
Masoomi 2011	14562				
Ilyas 2017	1973				
McKenna 2018	37004	unknown			
Posabella 2018	1016		100	LMWH	
Althans 2019	397				

Colectomy, laparoscopic, emergency

McKenna 2018	1953	unknown			
Sakran 2019	388				

Colectomy, laparoscopic, IBD

Umanskiy 2010	55				
Causey 2013	112				
Gu 2013	204				
Li 2015	159				

McKenna 2018	8588		unknown						
**Colectomy, laparoscopic, malignant**									
Yamamoto 2004	120								
Bilimoria 2008	837								
Chan 2008	429								
Magistro 2013	80						100	LMWH	30
Wright 2016	10853						1	LMWH	
Denet 2017	507								
Franco 2018	473								
Haskins 2018	2405								
McKenna 2018	42160		unknown						
Iwamoto 2019	390				13		9	Unspecified/UFH	30
**Colectomy, sigmoid, laparoscopic**									
Alves 2005	163						100†		30†
Garrett 2008	200								
Ilyas 2017	3946								
Posabella 2018	1016						100	LMWH	
**Colectomy, left, laparoscopic**									
Leroy 2005	111	0		0			100†	LMWH†	30†
Henke 2012	897	13	SCD				81	UFH/LMWH	
Cuccurullo 2015	585	83†	GCS†	1†	68†	Plavix, Ticlid, Aggrastat,ASA†			
Mrdutt 2017	35079								
McKenna 2018	47488		unknown						
**Colectomy, right, laparoscopic**									
Henke 2012	395	13	SCD				81	UFH/LMWH	
Magistro 2013	80						100	LMWH	30
Cuccurullo 2015	260	46†	GCS†	1†	33†	Plavix, Ticlid, Aggrastat,ASA†			

Li 2015	159					
Denet 2017	507					
Mrdutt 2017	8488					
Franco 2018	473					
McKenna 2018	19768		unknown			
**Colectomy, open**						
Alves 2005	169			100†		30†
Bilimoria 2008	2222					
Umanskiy 2010	70					
Masoomi 2011	110172					
Henke 2012	2172	13	SCD	43	UFH/LMWH	
Causey 2013	338					
Li 2015	159					
Wright 2016	29215			2	LMWH	
Ilyas 2017	17252					
Haskins 2018	1024					
McKenna 2018	5355		unknown			
Althans 2019	1778					
Sakran 2019	9822					
Krimphove 2020	2795					
Ross 2020	98994					
Weber 2020	2019					
**Colectomy, open, benign**						
Alves 2005	169			100†		30†
Masoomi 2011	110172					
Ilyas 2017	8626					
McKenna 2018	30442		unknown			
Althans 2019	1778					

**Colectomy, open, emergency**							
McKenna 2018	18033		unknown				
Sakran 2019	9822						
Weber 2020	2019						
**Colectomy, open, IBD**							
Umanskiy 2010	70						
Causey 2013	338						
Li 2015	159						
McKenna 2018	8058		unknown				
**Colectomy, open, malignant**							
Bilimoria 2008	2222						
Wright 2016	29215				2	LMWH	
Ilyas 2017	8626						
Haskins 2018	1024						
McKenna 2018	42007		unknown				
Krimphove 2020	2795						
**Colectomy, sigmoid, open**							
Alves 2005	169				100†		30†
Ilyas 2017	17252						
McKenna 2018	8270		unknown				
**Colectomy, left, open**							
Henke 2012	1334	13	SCD		81	UFH/LMWH	
McKenna 2018	21269		unknown				
**Colectomy, right, open**							
Henke 2012	838	13	SCD		81	UFH/LMWH	
Haskins 2018	1024						
McKenna 2018	19812		unknown				
**Colectomy, robotic**							

Tyler 2012	160									
Miller 2016	653									
Haskins 2018	89									
Raskin 2019	108									
**Colectomy, robotic, IBD**										
Raskin 2019	108									
**Colectomy, robotic, malignant**										
Haskins 2018	89									
**Colectomy, right, robotic**										
Haskins 2018	89									
Raskin 2019	108									
**Proctocolectomy, laparoscopic**										
Causey 2013	260									
Gu 2013	204									
Gu 2016	248	100	GCS	15				100	UFH/LMWH	15
Duraes 2018	119									
McKenna 2018	5756		unknown							
**Proctocolectomy, open**										
Remzi 2002	702							21	LMWH	
Causey 2013	517									
Ryoo 2014	72									
Gu 2016	273	100	GCS	15				100	UFH/LMWH	15
McKenna 2018	8180		unknown							
**Proctocolectomy, laparoscopic, benign**										
Duraes 2018	119									
McKenna 2018	238		unknown							
**Proctocolectomy, laparoscopic, IBD**										

Causey 2013	148							
Gu 2016	248	100	GCS	15		100	UFH/LMWH	15
McKenna 2018	4055		unknown					
**Proctocolectomy, laparoscopic, malignant**								
McKenna 2018	1307		unknown					
**Proctocolectomy, open, benign**								
McKenna 2018	708		unknown					
**Proctocolectomy, open, emergency**								
McKenna 2018	1932		unknown					
**Proctocolectomy, open, IBD**								
Remzi 2002	702					21	LMWH	
Causey 2013	397							
Ryoo 2014	72							
Gu 2016	273	100	GCS	15		100	UFH/LMWH	15
McKenna 2018	3130		unknown					
**Proctocolectomy, open, malignant**								
McKenna 2018	2410		unknown					
**Rectopexy, laparoscopic**								
Vogel 2020	3350							
**Rectopexy, open**								
Vogel 2020	3599							
**Rectopexy, perineal**								
Kimmins 2001	63							
Altomare 2009	93					100	Unspecified	4
Ding 2012	113							
Vogel 2020	5271							

Mechanical thromboprophylaxis included: antithrombosis stockings, intermittent pneumatic compression devices, and foot-pumps
Aspirin or other antiplatelet drugs included: aspirin, clopidogrel, prasugrel, ticlopidine, dipyridamole, ticagrelor, cilostazol, tirofiban, vorapaxar as well as thromboxane inhibitors, thromboxane synthase inhibitors, thromboxane receptor antagonists, and terutroban
Anticoagulants included: warfarin, low molecular weight heparin, low dose unfractionated heparin, dabigatran, apixaban, betrixaban, edoxaban, rivaroxaban, fondaparinux, danaparoid and lepirudin
Blank spaces represent no information (not provided by paper or by author correspondence).
Duration in days is expressed as mean or median.
GCS=graduated compression stockings; IPC= intermittent pneumatic compression (includes "intermittent compression device, sequential compression device, pneumatic compression device, pneumatic compression stockings, pneumatic compression boots"); LMWH= low molecular weight heparin; UFH= unfractionated heparin.
† Author provided this information.
§Follow up time of complications was not available from the article or author correspondence. We assumed a follow up time of 30 days.

10. Postoperative risk of symptomatic VTE and bleeding in individual studies in colorectal surgery

Reference	Total patients	Follow-up time	Reported VTE					Reported Bleeding			Baseline cumulative incidence at 4 weeks		
	n	Days	Fatal PE	Non-Fatal PE	DVT	VTE total* (excluding SVT)	SVT	Fatal Bleeding	Bleeding requiring reintervention	Transfusion	VTE at 4 weeks (%)	Bleeding requiring reintervention at 4 weeks (%)	Bleeding requiring transfusion at 4 weeks (%)
**Abdominoperineal resection, laparoscopic**													
Tooley 2018	2574	30		8	18	25‡					1,1%		4.9%
**Abdominoperineal resection, open**													
Tooley 2018	5107	30		45	82	123‡				1269	3.6%		21.5%
**Anterior resection, laparoscopic**													
Law 2006	98	30§	0		1	1‡			0		1.3%	0%	
Park 2011	130	30§			0	0‡		0		2	0%		1.4%
Liang 2013	263	30§			2	4					1.5%		
Osborne 2013	382	30§		6		24‡					9%		
Cuccurullo 2015	356	30	0				1	0	9			2.6%	
Lacy 2015	140	30							2	4		1%	1.9%
Park 2015	84	30						0	3	3		3.5%	3.5%
Tuech 2015	56	30§								2			2.4%
Law 2017	171	30§			0	0‡					0%		
Miyagaki 2017	6137					0‡							3.3%
McKenna 2018	33846	30				289†					1.1%		
**Anterior resection, open**													
Law 2006	167	30§	1		1	2‡			0		1.4%	0%	

Park 2011	80	30§			1	1‡				0	1.6%		0%
Kang 2013	72055	30§			375	481‡					1%		
McKenna 2018	21291	30				325†					2.1%		
Lee 2019	2521												7.5%
**Anterior resection, robotic**													
Park 2015	133	30						0	2	1		1.5%	0.7%
Law 2017	220	30§			2	3‡					1.2%		
**Colectomy, laparoscopic**													
Yamamoto 2004	120	30		1		4‡					3.3%		
Alves 2005	163	30§	0	0		0‡				4	0%		1.6%
Leroy 2005	111	30	0	0	1	1		0	2	5	1.9%	1.2%	2.9%
Bilimoria 2008	837					0‡							0.3%
Chan 2008	429	30§						1					
Garrett 2008	200	90	0	1	1	1		0			0.4%		
Umanskiy 2010	55	30§				0					0%		
Abarca 2011	358	30§							2			0.4%	
Kronberg 2011	413	30§			7	9‡					2.9%		
Masoomi 2011	14562	30§				19					0.2%		
Henke 2012	1292	30				17					1,7%		
Tyler 2012	2423	30§				11					0.7%		
Causey 2013	112					0‡							0%
Gu 2013	204	30				8			1		5.9%	0.3%	
Magistro 2013	80	30§						0	2	3		1.6%	2.4%
Cuccurullo 2015	845	8	0				3	0	9			1.1%	
Li 2015	159	30											
Miller 2016	11267					0‡							3%

Wright 2016	10853	90				317					1.9%		
Denet 2017	507	30	1					1					
Ilyas 2017	3946	30§			4	5‡					0.2%		0%
Franco 2018	473	30											
Posabella 2018	1016	30		4		16‡			1	9	2.2%	0.1%	0.6%
McKenna 2018	71411	30				941†					1.7%		0%
Sakran 2019	388	30		7	10	16‡					5.6%		
Ross 2020	62366	30				695					1.5%		
Krimphove 2020	4177	90				104					2%		
**Colectomy, laparoscopic, benign**													
Alves 2005	163	30§	0	0		0‡				4	0%		1.6%
Garrett 2008	200	90	0	1	1	1		0			0.4%		
Masoomi 2011	14562	30§				19					0.2%		
Ilyas 2017	1973	30§			2	3‡					0.2%		
McKenna 2018	37004	30				261†					0.9%		
Posabella 2018	1016	30		4		16‡			1	9	2.2%	0.1%	0.6%
Althans 2019	397					0‡							2.4%
**Colectomy, laparoscopic, emergency**													
McKenna 2018	1953	30				58†					3.9%		
Sakran 2019	388	30		7	10	16‡					5.6%		
**Colectomy, laparoscopic, IBD**													
Umanskiy 2010	55	30§				0					0%		
Causey 2013	112					0‡							0%
Gu 2013	204	30				8			1		5.9%	0.3%	
Li 2015	159	30											
McKenna 2018	8588	30				181†					2.8%		

Colectomy, laparoscopic, malignant

Yamamoto 2004	120	30		1		4‡					3.3%		
Bilimoria 2008	837												0.3%
Chan 2008	429	30§						1					
Magistro 2013	80	30§						0	2	3		1.6%	2.4%
Wright 2016	10853	90				317					1.9%		
Denet 2017	507	30	1					1					
Franco 2018	473	30											
Haskins 2018	2405					0‡							3.2%
McKenna 2018	42160	30				569†					1.8%		
Iwamoto 2019	390	30§		1	1	2	0		4		0.5%	1%	0.8%

Colectomy, sigmoid, laparoscopic

Alves 2005	163	30§	0	0		0‡				4	0%		1.6%
Garrett 2008	200	90	0	1	1	1		0			0.4%		
Ilyas 2017	3946	30§			4	5‡					0.2%		0%
Posabella 2018	1016	30		4		16‡			1	9	2.2%	0.1%	0.6%

Colectomy, left, laparoscopic

Leroy 2005	111	30	0	0	1	1		0	2	5	1.9%	1.2%	2.9%
Henke 2012	897	30				15					2.2%		
Cuccurullo 2015	585	30	0				3	0	6			1.1%	
Mrdutt 2017	35079					0‡							1.8%
McKenna 2018	47488	30				488†					1.4%		

Colectomy, right, laparoscopic

Henke 2012	395	30				2					0.6%		
Magistro 2013	80	30§						0	2	3		1.6%	1.6%
Cuccurullo 2015	260	30	0				0	0	3			1.3%	
Li 2015	159	30											

Denet 2017	507	30	1					1					
Mrdutt 2017	8488					0‡							2.7%
Franco 2018	473	30											
McKenna 2018	19768	30				286†					1.9%		

Colectomy, open

Alves 2005	169	30§		3		12‡				4	14.8%		1.5%
Bilimoria 2008	2222					0‡							0.2%
Umanskiy 2010	70	30§				2					4.4%		
Masoomi 2011	110172	30§				253					0.4%		
Henke 2012	2172	30				61					3,7%		
Causey 2013	338					0‡							0.3%
Li 2015	159	30											
Wright 2016	29215	90				1354					3%		
Ilyas 2017	17252	30§			26	33‡					0.3%		
Haskins 2018	1024					0‡							7%
McKenna 2018	5355	30				283†					7.5%		
Althans 2019	1778	30				32					2.6%		4.4%
Sakran 2019	9822	30		183	451	614‡					8.9%		
Krimphove 2020	2795	90				142					4.4%		
Ross 2020	98994	30				3177					4.6%		
Weber 2020	2019	30		25		74					5.2%		2.4%

Colectomy, open, benign

Alves 2005	169	30§		3		12‡				4	14.8%		1.5%
Masoomi 2011	110172	30§				253					0.4%		
Ilyas 2017	8626	30§			9	11‡					0.2%		
McKenna 2018	30442	30				454†					2.3%		
Althans 2019	1778	30				32					2.8%		4.4%

Colectomy, open, emergency

McKenna 2018	18033	30				790†					6.8%		
Sakran 2019	9822	30		183	451	614‡					9.7%		
Weber 2020	2019	30		25		74					5.7%		2.4%
**Colectomy, open, IBD**													
Umanskiy 2010	70	30§				2					4.4%		
Causey 2013	338					0‡							0.3%
Li 2015	159	30											
McKenna 2018	8058	30				196†					3.8%		
**Colectomy, open, malignant**													
Bilimoria 2008	2222					0‡							0.2%
Wright 2016	29215	90				1354					3%		
Ilyas 2017	8626	30§			17	22‡					0.4%		
Haskins 2018	1024					0‡							7%
McKenna 2018	42007	30				1043†					3.9%		
Krimphove 2020	2795	90				142					4.4%		
**Colectomy, sigmoid, open**													
Alves 2005	169	30§		3		12‡				4	14.8%		1.5%
Ilyas 2017	17252	30§			26	33‡					0.3%		
McKenna 2018	8270	30				112†					1.9%		
**Colectomy, left, open**													
Henke 2012	1334	30				41					4%		
McKenna 2018	21269	30				552†					4%		
**Colectomy, right, open**													
Henke 2012	838	30				19					3%		
Haskins 2018	1024					0‡							7%
McKenna 2018	19812	30				474†					3.7%		
**Colectomy, robotic**													
Tyler 2012	160	30§				5					4%		

Miller 2016	653	30		5	5	10‡					1.9%		2.3%
Haskins 2018	89	30	0	0	0	0					0%		5.2%
Raskin 2019	108	30			1	1‡					1.4%		
**Colectomy, robotic, IBD**													
Raskin 2019	108	30			1	1‡					1.4%		
**Colectomy, robotic, malignant**													
Haskins 2018	89	30	0	0	0	0					0%		5.2%
**Colectomy, right, robotic**													
Haskins 2018	89	30	0	0	0	0					0%		5.2%
Raskin 2019	108	30			1	1‡					1.4%		
**Proctocolectomy, laparoscopic**													
Causey 2013	260	30											0.4%
Gu 2013	204	30				8			1		5.9%	0.3%	
Gu 2016	248	30§					14						
Duraes 2018	119	30§		1	0	1‡	12			7	1.7%		4%
McKenna 2018	5756	30				219†					5%		
**Proctocolectomy, open**													
Remzi 2002	702	30					11						
Causey 2013	517	30			11					0			0.2%
Ryoo 2014	72	30			2	3‡			0	6	3.6%	0%	5.4%
Gu 2016	273	30§					14						
McKenna 2018	8180	30				376†					7.2%		
**Proctocolectomy, laparoscopic, benign**													
Duraes 2018	119	30§		1	0		12			7			4%
McKenna 2018	238	30				9†					5%		
**Proctocolectomy, laparoscopic, IBD**													
Causey 2013	148	30											0.8%

Gu 2016	248	30§					14						
McKenna 2018	4055	30				162†					5.3%		
**Proctocolectomy, laparoscopic, malignant**													
McKenna 2018	1307	30				34					3.4%		
**Proctocolectomy, open, benign**													
McKenna 2018	708	30				30					6%		
**Proctocolectomy, open, emergency**													
McKenna 2018	1932	30				136					10%		
**Proctocolectomy, open, IBD**													
Remzi 2002	702	30					11						
Causey 2013	397					0‡				0			0%
Ryoo 2014	72	30			2	3‡			0	6	3.6%	0%	7.5%
Gu 2016	273	30§					14						
McKenna 2018	3130	30				131					6%		
**Proctocolectomy, open, malignant**													
McKenna 2018	2410	30				79					4.7%		
**Rectopexy, laparoscopic**													
Vogel 2020	3350	30				10					0.4%		0.9%
**Rectopexy, open**													
Vogel 2020	3599	30				16					0.6%		1.8%
**Rectopexy, perineal**													
Kimmins 2001	63	30§						0	0	0		0%	0%
Altomare 2009	93	30§						0	1			0.7%	
Ding 2012	113	30§				1					1.9%		
Vogel 2020	5271	30				19					0.5%		0.9%

Cumulative risks are given for the first four postoperative weeks.
Blank spaces represent no information (not provided by paper or by author correspondence).
§Follow up time of complications was not available from the article or author correspondence. We assumed a follow up time of 30 days as this was median reported follow up time in the eligible studies.
* Excluding SVT
† Authors provided value.
‡ Estimated VTE value

11. Peri- and intraoperative risk of bleeding in individual studies in colorectal surgery

Reference	Total patients	Perioperative bleeding	Reported Intra-operative Bleeding		
	n	Peri-operative bleeding requiring transfusion	Fatal intra-operative bleeding	Intra-operative bleeding requiring conversion to open	Intra-operative bleeding requiring transfusion
**Abdominoperineal resection, laparoscopic**					
Tooley 2018	2574	276			
**Abdominoperineal resection, open**					
Tooley 2018	5107				
**Anterior resection, laparoscopic**					
Law 2006	98			1	
Park 2011	130			0	2
Liang 2013	263				
Osborne 2013	382				
Cuccurullo 2015	356			0	26
Lacy 2015	140				
Park 2015	84			0	
Tuech 2015	56				
Law 2017	171				
Miyagaki 2017	6137	434			434

McKenna 2018	33846				
**Anterior resection, open**					
Law 2006	167				
Park 2011	80			0	8
Kang 2013	72055				
McKenna 2018	21291				
Lee 2019	2521	408			
**Anterior resection, robotic**					
Park 2015	133			0	
Law 2017	220		2		
**Colectomy, laparoscopic**					
Yamamoto 2004	120				
Alves 2005	163				3
Leroy 2005	111		0	0	0
Bilimoria 2008	837	6			
Chan 2008	429			4	
Garrett 2008	200			0	
Umanskiy 2010	55			1	5
Abarca 2011	358				
Kronberg 2011	413				
Masoomi 2011	14562				
Henke 2012	1292				
Tyler 2012	2423				
Causey 2013	112	0			
Gu 2013	204				
Magistro 2013	80		0	0	

Cuccurullo 2015	845			0	27
Li 2015	159				27
Miller 2016	11267	721			
Wright 2016	10853				
Denet 2017	507				20
Ilyas 2017	3946				
Franco 2018	473				20
Posabella 2018	1016				
McKenna 2018	71411				
Sakran 2019	388				
Ross 2020	62366				
Krimphove 2020	4177				
**Colectomy, laparoscopic, benign**					
Alves 2005	163				3
Garrett 2008	200			0	
Masoomi 2011	14562				
Ilyas 2017	1973				
McKenna 2018	37004				
Posabella 2018	1016				
Althans 2019	397	21			
**Colectomy, laparoscopic, emergency**					
McKenna 2018	1953				
Sakran 2019	388				
**Colectomy, laparoscopic, IBD**					
Umanskiy 2010	55			1	5

Causey 2013	112	0			
Gu 2013	204				
Li 2015	159				27
McKenna 2018	8588				
**Colectomy, laparoscopic, malignant**					
Yamamoto 2004	120				
Bilimoria 2008	837	6			
Chan 2008	429			4	
Magistro 2013	80		0	0	
Wright 2016	10853				
Denet 2017	507				20
Franco 2018	473				20
Haskins 2018	2405	168			
McKenna 2018	42160				
Iwamoto 2019	390	5		3	
**Colectomy, sigmoid, laparoscopic**					
Alves 2005	163				3
Garrett 2008	200			0	
Ilyas 2017	3946				
Posabella 2018	1016				
**Colectomy, left, laparoscopic**					
Leroy 2005	111		0	0	0
Henke 2012	897				
Cuccurullo 2015	585			0	19
Mrdutt 2017	35079	1333			
McKenna 2018	47488				

**Colectomy, right, laparoscopic**					
Henke 2012	395				
Magistro 2013	80	3	0	0	
Cuccurullo 2015	260			0	8
Li 2015	159				27
Denet 2017	507				20
Mrdutt 2017	8488	492			
Franco 2018	473				20
McKenna 2018	19768				
**Colectomy, open**					
Alves 2005	169				10
Bilimoria 2008	2222	11			
Umanskiy 2010	70				5
Masoomi 2011	110172				
Henke 2012	2172				
Causey 2013	338	2			
Li 2015	159				25
Wright 2016	29215				
Ilyas 2017	17252				
Haskins 2018	1024	156			
McKenna 2018	5355				
Althans 2019	1778	170			
Sakran 2019	9822				
Krimphove 2020	2795				
Ross 2020	98994				
Weber 2020	2019	104			
**Colectomy, open, benign**					
Alves 2005	169				10

Masoomi 2011	110172				
Ilyas 2017	8626				
McKenna 2018	30442				
Althans 2019	1778	170			
**Colectomy, open, emergency**					
McKenna 2018	18033				
Sakran 2019	9822				
Weber 2020	2019	104			
**Colectomy, open, IBD**					
Umanskiy 2010	70				5
Causey 2013	338	2			
Li 2015	159				25
McKenna 2018	8058				
**Colectomy, open, malignant**					
Bilimoria 2008	2222	11			
Wright 2016	29215				
Ilyas 2017	8626				
Haskins 2018	1024	156			
McKenna 2018	42007				
Krimphove 2020	2795				
**Colectomy, sigmoid, open**					
Alves 2005	169				10
Ilyas 2017	17252				
McKenna 2018	8270				
**Colectomy, left, open**					
Henke 2012	1334				
McKenna 2018	21269				
**Colectomy, right, open**					

Henke 2012	838				
Haskins 2018	1024	156			
McKenna 2018	19812				
**Colectomy, robotic**					
Tyler 2012	160				
Miller 2016	653	32			
Haskins 2018	89	10			
Raskin 2019	108				
**Colectomy, robotic, IBD**					
Raskin 2019	108				
**Colectomy, robotic, malignant**					
Haskins 2018	89	10			
**Colectomy, right, robotic**					
Haskins 2018	89	10			
Raskin 2019	108				
**Proctocolectomy, laparoscopic**					
Causey 2013	260	2			
Gu 2013	204				
Gu 2016	248				
Duraes 2018	119				0
McKenna 2018	5756				
**Proctocolectomy, open**					
Remzi 2002	702				
Causey 2013	517	2			
Ryoo 2014	72				
Gu 2016	273				
McKenna 2018	8180				

**Proctocolectomy, laparoscopic, benign**					
Duraes 2018	119				0
McKenna 2018	238				
**Proctocolectomy, laparoscopic, IBD**					
Causey 2013	148	2			
Gu 2016	248				
McKenna 2018	4055				
**Proctocolectomy, laparoscopic, malignant**					
McKenna 2018	1307				
**Proctocolectomy, open, benign**					
McKenna 2018	708				
**Proctocolectomy, open, emergency**					
McKenna 2018	1932				
**Proctocolectomy, open, IBD**					
Remzi 2002	702				
Causey 2013	397				
Ryoo 2014	72				
Gu 2016	273				
McKenna 2018	3130				
**Proctocolectomy, open, malignant**					
McKenna 2018	2410				
**Rectopexy, laparoscopic**					
Vogel 2020	3350	66			
**Rectopexy, open**					
Vogel 2020	3599	138			

**Rectopexy, perineal**					
Kimmins 2001	63		0		0
Altomare 2009	93				
Ding 2012	113				
Vogel 2020	5271	103			

Blank spaces represent no information (not provided by paper or by author correspondence).

6. Upper-gastrointestinal and hepatopancreatobiliary surgery supplementary tables 12-17

12. Characteristics of individual studies in upper-gastrointestinal and hepatopancreatobiliary surgery

Reference	Year	Country/ Countries	Patients(n)	Age Mean (SD)*	Female (%)	Malignancy (%)	Length of stay (Days)	Recruitment First year	Recruitment Last year	Study type
**Distal pancreatectomy, laparoscopic**										
Anonsen	2015	Norway	69	58†	84	17	6†	1997	2009	One center, multiple surgeons
Nakamura	2015	Japan	902	57 (16)	64		19	2006	2013	Multicenter in one country
Sulpice	2015	France	347	61 (15)	57	100	15†	2007	2012	Multicenter in one country
Kwon	2016	Korea	111	50	69	20	11	1999	2012	One center, multiple surgeons
Dokmak	2017	France	165	54 (15)	62	38	16	2008	2015	One center, multiple surgeons
Daniel	2018	USA	1789	61 (14)	58	42		1999	2012	Multicenter in one country
Chen	2019	China	353	54 (14)	38	24	10	2004	2018	One center, multiple surgeons
**Distal pancreatectomy, robotic**										
Zureikat	2013	USA	83	65†	51	72	6	2008	2012	One center, multiple surgeons
**Distal pancreatectomy, open**										
Yekebas	2007	Germany	116					1992	2006	One center, multiple surgeons
Lee	2008	Korea	180		64		15	1995	2006	One center, multiple surgeons
Dedania§	2013	USA	70	66†	40	100	6†	2005	2011	One center, multiple surgeons
Nakamura	2015	Japan	1108	61 (15)	55	0	20	2006	2013	Multicenter in one country
Sulpice	2015	France	2406	65 (12)	51	100	15†	2007	2012	Multicenter in one country

Daniel	2018	USA	1790	61 (14)	58	63	7	2014	2016	Multicenter in one country
Boone	2019	USA	55	65 (7)	50	100		2007	2017	One center, multiple surgeons
Mussle§	2020	Germany	191	65 (18)†	51	67	19†	2005	2017	One center, multiple surgeons
**Distal pancreatectomy, laparoscopic, benign**										
Daniel	2018	USA	1030	61 (14)	58	0		2014	2016	Multicenter in one country
Chen	2019	China	116	50 (13)	72	0	9	2004	2018	One center, multiple surgeons
**Distal pancreatectomy, laparoscopic, malignant**										
Sulpice	2015	France	347	61 (15)	57	100	15†	2007	2012	Multicenter in one country
Daniel	2018	USA	759	61 (14)	58	100		2014	2016	Multicenter in one country
**Distal pancreatectomy, open, benign**										
Daniel	2018	USA	655	61 (14)	58	0	7	2014	2016	Multicenter in one country
**Distal pancreatectomy, open, malignant**										
Dedania§	2013	USA	70	66†	40	100	6†	2005	2011	One center, multiple surgeons
Sulpice	2015	France	2406	65 (12)	51	100	15†	2007	2012	Multicenter in one country
Daniel	2018	USA	1135	61 (14)	58	100	8	2014	2016	Multicenter in one country
Boone	2019	USA	55	65 (7)	50	100		2007	2017	One center, multiple surgeons
**Liver resection, laparoscopic**										
Vibert	2006	France	84	59†	44	73	11†	1995	2004	One center, multiple surgeons
Dagher	2009	USA,France,Italy,Australia	210	56†	65	54	6†	1997	2008	Multinational
Abu Hilal	2010	UK	80	64†		54	3†	2003	2007	One center, multiple surgeons
Dagher	2010	France,Italy	163	65†	31	100	8†	1998	2008	Multinational
Kazaryan	2010	Norway	139	62 (15)	53	81	3†	1998	2008	One center, multiple surgeons
Bhojani	2012	Canada	57	59†	60	67	5†	2006	2010	One center, multiple surgeons
Soubrane	2014	France	351	63†	26	100		1998	2010	Multicenter in one country
Cauchy	2015	France	223	64†	40	88	13†	2000	2013	Multicenter in one country
Fuks	2016	France	226	62	54	88	13†	2000	2013	Multicenter in one country
Cipriani	2018	Italy	698	62†	48	92	5†	2005	2017	One center, multiple surgeons

Sucandy	2018	USA	831		63	47	3	2001	2016	One center, multiple surgeons
Ainoa§	2020	Finland	84	63 (14)	52	82		2014	2017	One center, multiple surgeons
Triantafyllidis	2020	France	431	63 (11)	35	100		2000	2018	One center, multiple surgeons
Stiles	2017	USA	859		60	60	3†	2014	2015	Multicenter in one country
**Liver resection, robotic**										
Kingham	2016	USA	64	64†	50	78	4	2004/2010	2012/2014	Single surgeon series
Daskalaki	2017	USA	67	53 (15)	55	56	6	2009	2013	One center, multiple surgeons
Sucandy	2020	USA	77	62	57	85	3†	2016	2018	One center, multiple surgeons
**Liver resection, open**										
Stewart§	2004	UK	137	62†	39	100	10†	1988	2001	One center, multiple surgeons
Zhou§	2007	China	81	54 (11)	6	100		1995	2002	One center, multiple surgeons
Lee	2009	Hong Kong	248	54†	39	77	7	2003	2007	One center, multiple surgeons
Lordan	2009	UK	469	64†	69	83	9†	1996	2008	One center, multiple surgeons
Andres	2011	Switzerland	689	55 (15)	56	71		1991	2009	One center, multiple surgeons
Nobili	2012	France	555	56 (14)	56	1		2006	2009	One center, multiple surgeons
Tzeng§	2012	USA	5651	60†	51			2005	2009	Multicenter in one country
Barbas§	2013	USA	1281	55 (15)	53	74	9	1996	2009	One center, multiple surgeons
Aramaki§	2014	Japan	539		22	100		2001	2010	One center, multiple surgeons
Nathan	2014	USA	2147	60 (20)†	49	91	7†	2003	2011	One center, multiple surgeons
Bagante	2016	USA	2452	60 (18)†	49	83	6†	2014	2014	Multicenter in one country
de'Angelis	2016	France	329	55 (13)	47	100	16	1980	2011	One center, multiple surgeons
Fuks	2016	France	988	54	47	72	17†	2000	2013	Multicenter in one country
Yokoo	2016	Japan	14970	67 (12)	30			2011	2012	Multicenter in one country
Khandoga	2017	Germany	184	64 (1)	30	100		2003	2013	One center, multiple surgeons
Singh	2017	India	86	51 (16)	38	81		2010	2015	One center, multiple surgeons
Kron§	2019	UK	211	62†	40	91	10†	1993	2014	One center, multiple surgeons

Ainoa§	2020	Finland	428	63 (13)	43	88		2014	2017	One center, multiple surgeons
Snyder	2020	USA	388	59 (12)	39	97		2014	2016	Multicenter in one country
Tahkola	2020	Finland	73	65 (14)†	47	97	9†	2000	2017	One center, multiple surgeons
**Liver resection, laparoscopic, minor**										
Soubrane	2014	France	351	63†	26	100		1998	2010	Multicenter in one country
Stiles	2017	USA	859		60	60	5†	2014	2015	Multicenter in one country
Ainoa§	2020	Finland	78	63 (14)	52	82		2014	2017	One center, multiple surgeons
**Liver resection, laparoscopic, major**										
Dagher	2009	USA,France,Italy,Australia	210	56†	65	54	6†	1997	2008	Multinational
Cauchy	2015	France	223	64†	40	88	13†	2000	2013	Multicenter in one country
Fuks	2016	France	226	62	54	88	13†	2000	2013	Multicenter in one country
**Liver resection, open, minor**										
Tzeng§	2012	USA	3376	60†	51			2005	2009	Multicenter in one country
Aramaki§	2014	Japan	539	#N/A	22	100		2001	2010	One center, multiple surgeons
Ainoa§	2020	Finland	250	63 (13)	43	88		2014	2017	One center, multiple surgeons
**Liver resection, open, major**										
Zhou§	2007	China	81	54 (11)	6	100		1995	2002	One center, multiple surgeons
Tzeng§	2012	USA	1690	60†	51			2005	2009	Multicenter in one country
de'Angelis	2016	France	329	55 (13)	47	100	19	1980	2011	One center, multiple surgeons
Fuks	2016	France	988	54	47	72	17†	2000	2013	Multicenter in one country
Singh	2017	India	86	51 (16)	38	81		2010	2015	One center, multiple surgeons
Kron§	2019	UK	211	62†	40	91	10†	1993	2014	One center, multiple surgeons
Ainoa§	2020	Finland	178	63 (13)	43	88		2014	2017	One center, multiple surgeons
Snyder	2020	USA	388	59 (12)	39	97	14†	2014	2016	Multicenter in one country
Tahkola	2020	Finland	73	65 (14)†	47	97	9†	2000	2017	One center, multiple surgeons

**Pancreaticoduodenectomy, laparoscopic**										
Kendrick	2010	USA	62	66 (12)	48	73	7†	2007	2009	One center, multiple surgeons
Dokmak	2017	France	70	58 (13)	43	81	25	2008	2015	One center, multiple surgeons
Yu	2018	Korea	191	53 (14)	52	100	14	2008	2014	One center, multiple surgeons
Chen	2019	China	186	61 (11)	38	47	20	2004	2018	One center, multiple surgeons
Song§	2020	Korea	500	57 (14)	45	46	13	2007	2017	One center, multiple surgeons
Vining	2020	USA	407	64 (12)	46	77	7†	2014	2017	Multicenter in one country
Wang	2020	China	550	62†	40		13	2010	2019	One center, multiple surgeons
Boone	2019	USA	200	65 (7)	50	100		2007	2017	One center, multiple surgeons
**Pancreaticoduodenectomy, robotic**										
Zureikat	2013	USA	132	67†	51	80		2008	2012	One center, multiple surgeons
Rosemurgy§	2019	USA	155	69 (11)	43	81	5†	2013	2017	One center, multiple surgeons
Vining	2020	USA	498	65 (12)	48	78	7†	2014	2017	Multicenter in one country
**Pancreaticoduodenectomy, open**										
Martignoni	2001	Switzerland	257	67†	46	81	17†	1993	1999	One center, multiple surgeons
Adam	2004	Germany	301	50†	29	36	15†	1994	2001	One center, multiple surgeons
Balachandran	2004	India	218	50 (13)	27	100		1989	2002	One center, multiple surgeons
Tien	2005	Taiwan	402	59†	40	91		1995	2004	One center, multiple surgeons
Turrini	2005	France	172	59 (11)	30	100		1994	2003	One center, multiple surgeons
Koukoutsis	2006	UK	362	66†	45			2000	2005	One center, multiple surgeons
Blanc	2007	France	411			100		1992	2005	One center, multiple surgeons
Yekebas	2007	Germany	1141					1992	2006	One center, multiple surgeons
Tien	2008	Taiwan	283	61 (16)	40	81	25	2002	2007	One center, multiple surgeons
Wei	2009	Taiwan	628			81		1980	2007	One center, multiple surgeons

Pandanaboyana§	2010	UK	67	71 (10)	39	100		2004	2007	One center, multiple surgeons
Kneuertz	2011	USA	220	64 (12)	50	100	11†	2000	2008	One center, multiple surgeons
Mañas-Gómez	2011	Spain	107	65 (9)	6			2005	2008	One center, multiple surgeons
Ricci	2012	Italy	113	67 (11)	37	85		2009	2011	One center, multiple surgeons
Enomoto	2014	USA	9830	64	48		16	2005	2010	Multicenter in one country
Feng	2014	China	840	54†	35	89	35†	2000	2010	One center, multiple surgeons
Kokudo	2014	Switzerland	187			100	23†	2006	2012	One center, multiple surgeons
Ravikumar	2014	UK	1070	66†	46	100	13	1998	2011	Multicenter in one country
Flis	2016	Slovenia	111	66 (8)	52	100		2006	2014	One center, multiple surgeons
Soriano	2016	Spain	67	66 (2)	40	100		2005	2015	One center, multiple surgeons
Fujikawa	2018	Japan	100	73†	33	77	29†	2005	2016	One center, multiple surgeons
Kantor	2018	USA	9235	67 (11)	48	100	12	2006	2013	Multicenter in one country
Boone	2019	USA	327	65 (7)	50	100		2007	2017	One center, multiple surgeons
Faraj	2019	Lebanon	300	61†	36	89	12†	1994	2015	One center, multiple surgeons
Mataki	2019	Japan	315		38			2006	2018	One center, multiple surgeons
Rystedt§	2019	Sweden	1864	67 (10)	46	84		2011	2016	Multicenter in one country
Snyder	2019	USA	120	64 (11)†	53	100		2008	2015	One center, multiple surgeons
Cao	2020	China	151	59 (10)	39	88	20	2010	2017	One center, multiple surgeons
Komokata	2020	Japan	77	75†	31	73	33	2013	2019	One center, multiple surgeons
Mussle§	2020	Germany	699	65 (20)†	41	67		2005	2017	One center, multiple surgeons
Tahkola	2020	Finland	218	67 (13)†	0	86	12†	2000	2017	One center, multiple surgeons
Vining	2020	USA	12612	65 (12)	47	81	8†	2014	2017	Multicenter in one country
**Pancreaticoduodenectomy, laparoscopic, without venous resection**										

Dokmak	2017	France	70	58 (13)	43	81	25	2008	2015	One center, multiple surgeons
Kendrick	2010	USA	62	66 (12)	48	73	7†	2007	2009	One center, multiple surgeons
Yu	2018	Korea	191	53 (14)	52	100	14	2008	2014	One center, multiple surgeons
Wang	2020	China	473	62†	40		14	2010	2019	One center, multiple surgeons
**Pancreaticoduodenectomy, laparoscopic, with venous resection**										
Wang	2020	China	77	62†	40		13	2010	2019	One center, multiple surgeons
**Pancreaticoduodenectomy, open, without venous resection**										
Turrini	2005	France	172	59 (11)	30	100		1994	2003	One center, multiple surgeons
Blanc	2007	France	411			100		1992	2005	One center, multiple surgeons
Ravikumar	2014	UK	840	66†	44	100	13†	1998	2011	Multicenter in one country
Flis	2016	Slovenia	111	66 (8)	52	100		2006	2014	One center, multiple surgeons
Kantor	2018	USA	8258	66 (11)	48	100	12	2006	2013	Multicenter in one country
Faraj	2019	Lebanon	300	61†	36	89	12	1994	2015	One center, multiple surgeons
Cao	2020	China	151	59 (10)	39	88	20	2010	2017	One center, multiple surgeons
Zettervall	2020	USA	2566	64 (12)	46	95		2014	2015	Multicenter in one country
Feng	2014	China	840	54†	35	89	35†	2000	2010	One center, multiple surgeons
**Pancreaticoduodenectomy, open, with venous resection**										
Ravikumar	2014	UK	230	65†	50	100	14†	1998	2011	Multicenter in one country
Kantor	2018	USA	640	65 (10)	48	100	11	2006	2013	Multicenter in one country
Kantor	2018	USA	224	65 (10)	58	100	15	2006	2013	Multicenter in one country
Snyder	2019	USA	120	64 (11)†	53	100		2008	2015	One center, multiple surgeons
Zettervall	2020	USA	436	64 (11)	51	78		2014	2015	Multicenter in one country
**Gastrectomy, laparoscopic**										
Sexton	2008	USA	61	59 (19)	49		4	1995	2007	One center, multiple surgeons

Saka	2010	Japan	178			100		2002	2008	One center, multiple surgeons
Mamidanna	2013	UK	480		43	100	11†	2000	2010	Multicenter in one country
Son	2014	Korea	58	59 (12)	62	100	8	2003	2010	One center, multiple surgeons
Glenn	2015	USA	789		37	13	12	2008	2013	Multicenter in one country
Suda§	2015	Japan	438	68†	30	100	15†	2009	2012	One center, multiple surgeons
Chen	2016	China	253	58 (12)	32	100	9	2006	2015	One center, multiple surgeons
Chen	2016	China	379	60 (11)	47	100	10	2007	2015	One center, multiple surgeons
Han	2016	Korea	1355	60 (12)	34	100		2007	2012	One center, multiple surgeons
Nakauchi	2016	Japan	437	68 (14)	30	100	15	2009	2012	One center, multiple surgeons
Ntutumu	2016	China	1205	55 (12)	32	100	10	2004	2014	One center, multiple surgeons
Wang	2017	China	1657	62 (11)	22	100		2008	2015	Single surgeon series
Hiki	2018	Japan	1067	70 (14)†	31	100	14	2014	2015	Multicenter in one country
Osaki	2018	Japan	129	69 (10)	26	100		2014	2017	One center, multiple surgeons
Shimada	2018	Japan	243	69 (11)	28	100		2007	2014	One center, multiple surgeons
Xu	2019	China	430	56 (10)	21	100	8†	2005	2012	One center, multiple surgeons
Alzahrani	2020	Korea	207		26	100		2018	2019	One center, multiple surgeons
Sakamoto	2020	Japan	13187		28	100	14†	2010	2017	Multicenter in one country
Shibasaki§	2020	Japan	1042	70†	29	100	13†	2009	2019	One center, multiple surgeons
**Gastrectomy, robotic**										
Song	2009	Korea	100	55 (13)	46	100	8	2005	2007	One center, multiple surgeons
Son	2014	Korea	51	55 (12)	55	100	9	2003	2010	One center, multiple surgeons
Glenn	2015	USA	223		31	45	12	2008	2013	Multicenter in one country
Suda§	2015	Japan	88	64†	42	100	14†	2009	2012	One center, multiple surgeons
Nakauchi	2016	Japan	84	64 (13)	43	100	14	2009	2012	One center, multiple surgeons

Alhossaini	2019	Korea	288	56 (13)	41	100		2016	2017	One center, multiple surgeons
Okabe	2019	Japan	115	68†	35	100	12†	2012	2017	Multicenter in one country
Shibasaki§	2020	Japan	359	67†	35	100	12†	2009	2019	One center, multiple surgeons
**Gastrectomy, open**										
Park	2005	Korea	548	57 (12)	31			2002	2002	One center, multiple surgeons
Pedrazzani	2007	Italy	310	71†	46	100		1988	2003	One center, multiple surgeons
Lamb	2008	UK	180	70†	33	100	10†	1992	2005	One center, multiple surgeons
Oh	2009	Korea	410		32	100	12	2000	2003	One center, multiple surgeons
Sah	2009	China	809	58†	36	100				One center, multiple surgeons
Saka	2010	Japan	3014			100		2002	2008	One center, multiple surgeons
Mamidanna	2013	UK	10233		34	100	14†	2000	2010	Multicenter in one country
Papenfuss	2014	USA	2580	67 (13)	3	100	12	2005	2010	Multicenter in one country
Glenn	2015	USA	8585		31	1	13	2008	2013	Multicenter in one country
Han	2016	Korea	3256	60 (12)	34	99		2007	2012	One center, multiple surgeons
Chen	2017	China	124	54 (15)	35	100	11	2007	2016	One center, multiple surgeons
Kung	2017	Sweden	1101	69 (12)	43	100		2006	2013	Multicenter in one country
Hiki	2018	Japan	1067	71 (14)†	31	100	16	2014	2015	Multicenter in one country
Xu	2019	China	768	57 (11)	23	100	9†	2005	2012	One center, multiple surgeons
Sakamoto	2020	Japan	45502		25	100	15†	2010	2017	Multicenter in one country
**Gastrectomy, laparoscopic, subtotal**										
Sexton	2008	USA	61	59 (19)	49		4	1995	2007	One center, multiple surgeons
Chen	2016	China	379	60 (11)	47	100	10	2007	2015	One center, multiple surgeons
Hiki	2018	Japan	1067	70 (14)†	31	100	14	2014	2015	Multicenter in one country
Shimada	2018	Japan	243	69 (11)	28	100		2007	2014	One center, multiple surgeons
**Gastrectomy, laparoscopic, total**										

Son	2014	Korea	58	59 (12)	62	100	8	2003	2010	One center, multiple surgeons
Chen	2016	China	253	58 (12)	32	100	9	2006	2015	One center, multiple surgeons
Wang	2017	China	1657	62 (11)	22	100		2008	2015	Single surgeon series
Sakamoto	2020	Japan	13187		28	100	14†	2010	2017	Multicenter in one country
**Gastrectomy, robotic, total**										
Son	2014	Korea	51	55 (12)	55	100	9	2003	2010	One center, multiple surgeons
**Gastrectomy, open, subtotal**										
Park	2005	Korea	403	57 (12)	31			2002	2002	One center, multiple surgeons
Pedrazzani	2007	Italy	310	71†	46	100		1988	2003	One center, multiple surgeons
Sah	2009	China	809	58†	36	100				One center, multiple surgeons
Saka	2010	Japan	2111			100		2002	2008	One center, multiple surgeons
Papenfuss	2014	USA	1581	68 (13)	43	100	12	2005	2010	Multicenter in one country
Hiki	2018	Japan	1067	71 (14)†	31	100	16	2014	2015	Multicenter in one country
**Gastrectomy, open, total**										
Park	2005	Korea	145	57 (12)	31			2002	2002	One center, multiple surgeons
Oh	2009	Korea	410		32	100	12	2000	2003	One center, multiple surgeons
Saka	2010	Japan	903			100		2002	2008	One center, multiple surgeons
Papenfuss	2014	USA	999	64 (13)	40	100	13	2005	2010	Multicenter in one country
Chen	2017	China	124	54 (15)	35	100	11	2007	2016	One center, multiple surgeons
Sakamoto	2020	Japan	45502		25	100	15†	2010	2017	Multicenter in one country
**Gastric bypass, laparoscopic**										
Kothari	2007	USA	476	43 (9)		0				One center, multiple surgeons
Rabl	2011	USA	644	45 (11)	81			2004	2009	Multicenter in one country
Benizri	2013	France	100	41 (11)	83		3	2009	2011	One center, multiple surgeons
Woo	2013	Korea	55	35 (12)	90	0	3	2009	2011	One center, multiple surgeons

Inaba	2018	USA	128349	45 (17)†	0			2008	2012	Multicenter in one country
Thereaux	2018	France	33611	40 (12)	83			2012	2014	Multicenter in one country
Dugan	2020	USA	117599	45 (12)	80			2015	2016	Multicenter in one country
Gambhir	2020	USA,Canada	102146	45 (18)†	81	0	2	2015	2017	Multinational
Sada	2020	USA	561	48 (12)	81			2015	2018	One center, multiple surgeons

Gastric bypass, robotic

Yu	2006	USA	100	42 (10)	83		3	2003	2005	One center, multiple surgeons
Ayloo	2011	USA	90	39 (9)	87		2	2006	2009	Single surgeon series
Benizri	2013	France	100	41 (11)	83			2009	2011	One center, multiple surgeons
Myers	2013	USA	100	46 (10)	76		2	2009	2011	Single surgeon series
Tieu	2013	USA	1100	47	86			2002	2010	Multicenter in one country
Ayloo	2016	USA	146	40	88	0	3	2006	2013	Single surgeon series
Acevedo	2020	USA	5817	47 (12)	80		2	2015	2016	Multicenter in one country

Gastric bypass, open

Fernandez Jr	2004	USA	1431	41 (10)	78	0		1992	2003	One center, multiple surgeons
Cotter	2005	USA	107	40 (12)	79	0	4	2000	2001	Single surgeon series
Abou-Nukta	2006	USA	1225		79			1998	2003	One center, multiple surgeons
Gargiulo	2006	USA	606		71	0		1999	2001	One center, multiple surgeons
Gargiulo	2007	USA	193					1999	2003	One center, multiple surgeons
Nguyen	2007	USA	6065		79		4	2004	2006	Multicenter in one country
Martins-Filho	2008	Brazil	135	38†	47			1997	2003	One center, multiple surgeons
Weller§	2008	USA	4883		82		4	2005	2005	Multicenter in one country
Caruana	2009	USA	1652	42 (7)	84			2000	2008	One center, multiple surgeons
Consortium Longitudinal Assessment of Bariatric Surgery, Flum	2009	USA	437	46 (11)	68	0		2005	2007	Multicenter in one country

Slotman	2010	USA	61	37†	66		3†	1999	2008	Single surgeon series
Finks	2011	USA	1092	47 (11)	74	0		2006	2010	Multicenter in one country
Hutter	2011	USA	988	46	78		4	2007	2010	Multicenter in one country
Rabl	2011	USA	78	45 (11)	81			2004	2009	Multicenter in one country
Froehling	2012	USA	228	44 (10)	82			1987	2005	Multicenter in one country
Masoomi	2012	USA	42591	45 (11)	79	0		2006	2008	Multicenter in one country
Santo	2013	Brazil	538	46 (13)	83			2006	2011	One center, multiple surgeons
Lidor	2014	USA	5282	45	78	0	4	2005	2012	Multicenter in one country
Nielsen	2018	USA	503	45 (12)	79			2012	2014	Multicenter in one country
**Sleeve gastrectomy, laparoscopic**										
Woo	2013	Korea	132	35 (12)	90	0		2009	2011	One center, multiple surgeons
Alsina§	2014	Spain,Mexico	100	43 (9)	76	0		2007	2013	Multinational
Biertho	2014	Canada	378	48 (11)	66			2006	2011	One center, multiple surgeons
Sakran§	2016	Israel	3003	43 (15)	63	0	2	2006	2014	One center, multiple surgeons
Villagran	2016	Chile	1236	34	0	0	8	2009	2015	One center, multiple surgeons
Moradian	2017	USA	50					2014	2015	One center, multiple surgeons
Brunetti	2018	USA	60	43 (12)	53	0		?	?	Single surgeon series
Guerrier	2018	USA	47982	44 (4)	78			2010	2014	Multicenter in one country
Inaba	2018	USA	30257	45 (11)	75			2008	2012	Multicenter in one country
Nimeri	2018	United Arab Emirates	523	35 (10)	67	0		2010	2016	One center, multiple surgeons
Thereaux	2018	France	62266	40 (12)	83			2012	2014	Multicenter in one country
Abuoglu	2019	Turkey	302	34†	68		3†	2015	2017	One center, multiple surgeons
AlKhaldi	2019	Kuwait	187	37 (10)	72			2008	2011	One center, multiple surgeons
Dugan	2020	USA	312065	44 (12)	79			2015	2016	Multicenter in one country
Gambhir	2020	USA,Canada	266886	44 (18)†	80	0	2	2015	2017	Multinational
Johari	2020	Australia	259	43 (12)	70	0	5	2008	2015	One center, multiple surgeons

**Sleeve gastrectomy, robotic**										
Romero	2013	USA	134	43 (13)	66		2	2009	2012	One center, multiple surgeons
Ecker	2016	USA	411	44 (11)	75		3†	2011	2014	One center, multiple surgeons
Moon	2018	USA	740					2008	2016	Multicenter in one country
Acevedo	2020	USA	12912	45 (12)	80		2	2015	2016	Multicenter in one country

Blank spaces indicate an absence of information.

Articles are reported by procedure, so duplicate information from same study appears in this table.

Many articles reported on more than one procedure (For instance Masoomi 2011 reported on colectomy, laparoscopic; colectomy, laparoscopic, benign; colectomy, open; and colectomy, open, benign).

*Age is reported as mean (SD) unless otherwise indicated

† Median (IQR)

§ Authors confirmed accuracy of our consensus data extraction and/or corrected some errors or provided additional information

Bagante 2016: Open and minimally-invasive liver resection: Age was provided for procedures combined.
Benizri 2013: Laparoscopic and robotic gastric bypass: Age and proportion of females was provided for procedures combined.
Boone 2019: Open distal pancreatectomy and open and laparoscopic pancreaticoduodenectomy: Age and proportion of females was provided for procedures combined.
Froehling 2012: Open and laparoscopic gastric bypass: Age and proportion of females was provided for procedures combined.
Han 2016: Open and laparoscopic gastrectomy: Age and proportion of females was provided for procedures combined.
Lidor 2014: Open and laparoscpic gastric bypass: Age and proportion of females was provided for procedures combined.
Mussle 2020: Open distal pancreatectomy and open pancreaticoduodenectomy: Age and proportion of females was provided for procedures combined.
Nielsen 2018: Open gastric bypass, several others: Age and proportion of females was provided for procedures combined.
Park 2005: Open subtotal and total gastrectomies: Age and proportion of females was provided for procedures combined.
Rabl 2011: Laparoscopic and robotic gastric bypass: Age and proportion of females was provided for procedures combined.
Threaux 2018: Laparoscopic gastric bypass and sleeve gastrectomy: Age and proportion of females was provided for procedures combined.
Tzeng 2012: All liver resections: Age and proportion of females was provided for procedures combined.
Wang 2020: Laparoscopic pancreaticoduodenectomy and laparoscopic distal pancreatectomy: Age and proportion of females was provided for procedures combined.
Woo 2013: Laparoscopic gastric bypass and sleeve gastrectomy: Age and proportion of females was provided for procedures combined.
Zureikat 2013:Distal pancreatectomy and pancreaticoduodenectomy: Age and proportion of females was provided for all procedures combined.

13. Risk of bias in individual studies in upper-gastrointestinal and hepatopancreatobiliary surgery

Reference	Sampling	Thromboprophylaxis documentation	Source of information	Recruitment years	Specification of length of follow-up	Study type	Risk of Bias
**Distal pancreatectomy, laparoscopic**							
Anonsen 2015	+	-	Prospective data collection	-	-	One center, multiple surgeons	HIGH
Nakamura 2015	+	-	Retrospective chart reviews, data collected by one investigator	+	-	Multicenter in one country	HIGH
Sulpice 2015	+	-	Administrative database information	+	-	Multicenter in one country	HIGH
Kwon 2016	+	-	Retrospective chart reviews, data collected by one investigator	-	+	One center, multiple surgeons	HIGH
Dokmak 2017	+	-	Prospective data collection	+	-	One center, multiple surgeons	MODERATE
Daniel 2018	+	-	Prospective data collection	+	+	Multicenter in one country	LOW
Chen 2019	+	-	Retrospective chart reviews, data collected by one investigator	+	-	One center, multiple surgeons	HIGH
**Distal pancreatectomy, robotic**							
Zureikat 2013	+	-	Retrospective chart reviews, data collected by one investigator	+	+	One center, multiple surgeons	MODERATE
**Distal pancreatectomy, open**							
Yekebas 2007	+	-	Administrative database information	-	+	One center, multiple surgeons	HIGH
Lee 2008	+	-	Retrospective chart reviews, data collected by one investigator	-	-	One center, multiple surgeons	HIGH
Dedania 2013	+	+	Administrative database information	-	+	One center, multiple surgeons	MODERATE
Nakamura 2015	+	-	Retrospective chart reviews, data collected by one investigator	+	-	Multicenter in one country	HIGH
Sulpice 2015	+	-	Administrative database information	+	-	Multicenter in one country	HIGH
Daniel 2018	+	-	Prospective data collection	+	+	Multicenter in one country	LOW
Boone 2019	+	-	Retrospective chart reviews, data collected by one investigator	+	+	One center, multiple surgeons	MODERATE
Mussle 2020	+	+	Administrative database information	+	+	One center, multiple surgeons	LOW
**Distal pancreatectomy, laparoscopic, benign**							

Daniel 2018	+	-	Prospective data collection	+	+	Multicenter in one country	LOW
Chen 2019	+	-	Retrospective chart reviews, data collected by one investigator	+	-	One center, multiple surgeons	HIGH
**Distal pancreatectomy, laparoscopic, malignant**							
Sulpice 2015	+	-	Administrative database information	+	-	Multicenter in one country	HIGH
Daniel 2018	+	-	Prospective data collection	+	+	Multicenter in one country	LOW
**Distal pancreatectomy, open, benign**							
Daniel 2018	+	-	Prospective data collection	+	+	Multicenter in one country	LOW
**Distal pancreatectomy, open, malignant**							
Dedania 2013	+	+	Administrative database information	-	+	One center, multiple surgeons	MODERATE
Sulpice 2015	+	-	Administrative database information	+	-	Multicenter in one country	HIGH
Daniel 2018	+	-	Prospective data collection	+	+	Multicenter in one country	LOW
Boone 2019	+	-	Retrospective chart reviews, data collected by one investigator	+	+	One center, multiple surgeons	MODERATE
**Liver resection, laparoscopic**							
Vibert 2006	+	-	Retrospective chart reviews, data collected by one investigator	-	-	One center, multiple surgeons	HIGH
Dagher 2009	+	-	Prospective data collection	-	-	Multinational	HIGH
Abu Hilal 2010	+	-	Retrospective chart reviews, data collected by one investigator	-	-	One center, multiple surgeons	HIGH
Dagher 2010	+	-	Prospective data collection	-	-	Multinational	HIGH
Kazaryan 2010	+	-	Retrospective chart reviews, data collected by one investigator	-	-	One center, multiple surgeons	HIGH
Bhojani 2012	+	-	Retrospective chart reviews, data collected by one investigator	-	-	One center, multiple surgeons	HIGH
Soubrane 2014	+	-	Prospective data collection	-	-	Multicenter in one country	HIGH
Cauchy 2015	+	-	Retrospective chart reviews, data collected by one investigator	-	+	Multicenter in one country	HIGH
Fuks 2016	+	-	Retrospective chart reviews, data collected by one investigator	-	+	Multicenter in one country	HIGH
Cipriani 2018	+	-	Prospective data collection	+	+	One center, multiple surgeons	LOW
Sucandy 2018	+	-	Prospective data collection	-	-	One center, multiple surgeons	HIGH
Ainoa 2020	+	+	Retrospective chart reviews, data collected by one investigator	+	+	One center, multiple surgeons	LOW
Triantafyllidis 2020	+	-	Administrative database information	-	+	One center, multiple surgeons	HIGH
Stiles 2017	+	-	Prospective data collection	+	+	Multicenter in one country	LOW

**Liver resection, robotic**							
Kingham 2016	+	-	Retrospective chart reviews, data collected by one investigator	-	-	Single surgeon series	HIGH
Daskalaki 2017	+	-	Retrospective chart reviews, data collected by one investigator	+	-	One center, multiple surgeons	HIGH
Sucandy 2020	+	-	Prospective data collection	+	+	One center, multiple surgeons	LOW
**Liver resection, open**							
Stewart 2004	+	+	Prospective data collection	-	+	One center, multiple surgeons	LOW
Zhou 2007	+	-	Prospective data collection	-	-	One center, multiple surgeons	HIGH
Lee 2009	+	-	Prospective data collection	-	+	One center, multiple surgeons	MODERATE
Lordan 2009	+	-	Prospective data collection	-	+	One center, multiple surgeons	MODERATE
Andres 2011	+	-	Prospective data collection	-	+	One center, multiple surgeons	MODERATE
Nobili 2012	+	+	Prospective data collection	-	-	One center, multiple surgeons	MODERATE
Tzeng 2012	+	-	Prospective data collection	-	+	Multicenter in one country	MODERATE
Barbas 2013	+	+	Retrospective chart reviews, data collected by one investigator	-	+	One center, multiple surgeons	MODERATE
Aramaki 2014	+	+	Retrospective chart reviews, data collected by one investigator	-	+	One center, multiple surgeons	MODERATE
Nathan 2014	+	+	Prospective data collection	-	+	One center, multiple surgeons	LOW
Bagante 2016	+	-	Prospective data collection	+	+	Multicenter in one country	LOW
de'Angelis 2016	+	-	Administrative database information	-	+	One center, multiple surgeons	HIGH
Fuks 2016	+	-	Retrospective chart reviews, data collected by one investigator	-	+	Multicenter in one country	HIGH
Yokoo 2016	+	-	Prospective data collection	+	+	Multicenter in one country	LOW
Khandoga 2017	+	-	Prospective data collection	-	+	One center, multiple surgeons	MODERATE
Singh 2017	+	+	Retrospective chart reviews, data collected by one investigator	+	-	One center, multiple surgeons	MODERATE
Kron 2019	+	+	Administrative database information	-	+	One center, multiple surgeons	MODERATE
Ainoa 2020	+	+	Retrospective chart reviews, data collected by one investigator	+	+	One center, multiple surgeons	LOW
Snyder 2020	+	-	Prospective data collection	+	+	Multicenter in one country	LOW
Tahkola 2020	+	-	Prospective data collection	-	+	One center, multiple surgeons	MODERATE

**Liver resection, laparoscopic, minor**							
Soubrane 2014	+	-	Prospective data collection	-	-	Multicenter in one country	HIGH
Stiles 2017	+	-	Prospective data collection	+	+	Multicenter in one country	LOW
Ainoa 2020	+	+	Retrospective chart reviews, data collected by one investigator	+	+	One center, multiple surgeons	LOW
**Liver resection, laparoscopic, major**							
Dagher 2009	+	-	Prospective data collection	-	-	Multinational	HIGH
Cauchy 2015	+	-	Retrospective chart reviews, data collected by one investigator	-	+	Multicenter in one country	HIGH
Fuks 2016	+	-	Retrospective chart reviews, data collected by one investigator	-	+	Multicenter in one country	HIGH
**Liver resection, open, minor**							
Tzeng 2012	+	-	Prospective data collection	-	+	Multicenter in one country	MODERATE
Aramaki 2014	+	+	Retrospective chart reviews, data collected by one investigator	-	+	One center, multiple surgeons	MODERATE
Ainoa 2020	+	+	Retrospective chart reviews, data collected by one investigator	+	+	One center, multiple surgeons	LOW
**Liver resection, open, major**							
Zhou 2007	+	-	Prospective data collection	-	-	One center, multiple surgeons	HIGH
Tzeng 2012	+	-	Prospective data collection	-	+	Multicenter in one country	MODERATE
de'Angelis 2016	+	-	Administrative database information	-	+	One center, multiple surgeons	HIGH
Fuks 2016	+	-	Retrospective chart reviews, data collected by one investigator	-	+	Multicenter in one country	HIGH
Singh 2017	+	+	Retrospective chart reviews, data collected by one investigator	+	-	One center, multiple surgeons	MODERATE
Kron 2019	+	+	Administrative database information	-	+	One center, multiple surgeons	MODERATE
Ainoa 2020	+	+	Retrospective chart reviews, data collected by one investigator	+	+	One center, multiple surgeons	LOW
Snyder 2020	+	-	Prospective data collection	+	+	Multicenter in one country	LOW
Tahkola 2020	+	-	Prospective data collection	-	+	One center, multiple surgeons	MODERATE
**Pancreaticoduodenectomy, laparoscopic**							
Kendrick 2010	+	+	Retrospective chart reviews, data collected by one investigator	-	-	One center, multiple surgeons	HIGH
Dokmak 2017	+	-	Prospective data collection	+	-	One center, multiple surgeons	MODERATE

Yu 2018	+	-	Retrospective chart reviews, data collected by one investigator	+	-	One center, multiple surgeons	HIGH
Chen 2019	+	-	Retrospective chart reviews, data collected by one investigator	+	-	One center, multiple surgeons	HIGH
Song 2020	+	+	Retrospective chart reviews, data collected by one investigator	+	-	One center, multiple surgeons	MODERATE
Vining 2020	+	-	Prospective data collection	+	+	Multicenter in one country	LOW
Wang 2020	+	-	Retrospective chart reviews, data collected by one investigator	+	-	One center, multiple surgeons	HIGH
Boone 2019	+	-	Retrospective chart reviews, data collected by one investigator	+	+	One center, multiple surgeons	MODERATE
**Pancreaticoduodenectomy, robotic**							
Zureikat 2013	+	-	Retrospective chart reviews, data collected by one investigator	+	+	One center, multiple surgeons	MODERATE
Rosemurgy 2019	+	-	Prospective data collection	+	+	One center, multiple surgeons	LOW
Vining 2020	+	-	Prospective data collection	+	+	Multicenter in one country	LOW
**Pancreaticoduodenectomy, open**							
Martignoni 2001	+	-	Prospective data collection	-	-	One center, multiple surgeons	HIGH
Adam 2004	+	-	Prospective data collection	-	-	One center, multiple surgeons	HIGH
Balachandran 2004	+	-	Prospective data collection	-	-	One center, multiple surgeons	HIGH
Tien 2005	+	-	Retrospective chart reviews, data collected by one investigator	-	+	One center, multiple surgeons	HIGH
Turrini 2005	+	-	Retrospective chart reviews, data collected by one investigator	-	+	One center, multiple surgeons	HIGH
Koukoutsis 2006	+	-	Prospective data collection	-	+	One center, multiple surgeons	MODERATE
Blanc 2007	+	-	Prospective data collection	-	+	One center, multiple surgeons	MODERATE
Yekebas 2007	+	-	Administrative database information	-	+	One center, multiple surgeons	HIGH
Tien 2008	+	-	Retrospective chart reviews, data collected by one investigator	-	-	One center, multiple surgeons	HIGH
Wei 2009	+	-	Retrospective chart reviews, data collected by one investigator	-	-	One center, multiple surgeons	HIGH
Pandanaboyana 2010	+	+	Administrative database information	-	+	One center, multiple surgeons	MODERATE
Kneuertz 2011	+	-	Retrospective chart reviews, data collected by one investigator	-	-	One center, multiple surgeons	HIGH
Mañas-Gómez 2011	+	-	Prospective data collection	-	-	One center, multiple surgeons	HIGH

Ricci 2012	+	-	Prospective data collection	+	+	One center, multiple surgeons	LOW
Enomoto 2014	+	-	Retrospective chart reviews, data collected by one investigator	-	+	Multicenter in one country	HIGH
Feng 2014	+	-	Retrospective chart reviews, data collected by one investigator	-	-	One center, multiple surgeons	HIGH
Kokudo 2014	+	+	Retrospective chart reviews, data collected by one investigator	-	+	One center, multiple surgeons	MODERATE
Ravikumar 2014	+	-	Retrospective chart reviews, data collected by one investigator	-	-	Multicenter in one country	HIGH
Flis 2016	+	-	Prospective data collection	+	-	One center, multiple surgeons	MODERATE
Soriano 2016	+	-	Prospective data collection	+	+	One center, multiple surgeons	LOW
Fujikawa 2018	+	+	Retrospective chart reviews, data collected by one investigator	+	+	One center, multiple surgeons	LOW
Kantor 2018	+	-	Prospective data collection	+	+	Multicenter in one country	LOW
Boone 2019	+	-	Retrospective chart reviews, data collected by one investigator	+	+	One center, multiple surgeons	MODERATE
Faraj 2019	+	-	Retrospective chart reviews, data collected by one investigator	-	-	One center, multiple surgeons	HIGH
Mataki 2019	+	-	Retrospective chart reviews, data collected by one investigator	+	+	One center, multiple surgeons	MODERATE
Rystedt 2019	-	+	Prospective data collection	+	-	Multicenter in one country	MODERATE
Snyder 2019	+	+	Retrospective duplicate chart reviews with good documentation of agreement between reviewers	+	+	One center, multiple surgeons	VERY LOW
Cao 2020	+	-	Retrospective chart reviews, data collected by one investigator	+	-	One center, multiple surgeons	HIGH
Komokata 2020	+	-	Retrospective chart reviews, data collected by one investigator	+	-	One center, multiple surgeons	HIGH
Mussle 2020	+	+	Administrative database information	+	+	One center, multiple surgeons	LOW
Tahkola 2020	+	-	Prospective data collection	-	+	One center, multiple surgeons	MODERATE
Vining 2020	+	-	Prospective data collection	+	+	Multicenter in one country	LOW
**Pancreaticoduodenectomy, laparoscopic, without venous resection**							
Dokmak 2017	+	-	Prospective data collection	+	-	One center, multiple surgeons	MODERATE
Kendrick 2010	+	+	Retrospective chart reviews, data collected by one investigator	-	-	One center, multiple surgeons	HIGH
Yu 2018	+	-	Retrospective chart reviews, data collected by one investigator	+	-	One center, multiple surgeons	HIGH

Wang 2020	+	-	Retrospective chart reviews, data collected by one investigator	+	-	One center, multiple surgeons	HIGH
**Pancreaticoduodenectomy, laparoscopic, with venous resection**							
Wang 2020	+	-	Retrospective chart reviews, data collected by one investigator	+	-	One center, multiple surgeons	HIGH
**Pancreaticoduodenectomy, open, without venous resection**							
Turrini 2005	+	-	Retrospective chart reviews, data collected by one investigator	-	+	One center, multiple surgeons	HIGH
Blanc 2007	+	-	Prospective data collection	-	+	One center, multiple surgeons	MODERATE
Ravikumar 2014	+	-	Retrospective chart reviews, data collected by one investigator	-	-	Multicenter in one country	HIGH
Flis 2016	+	-	Prospective data collection	+	-	One center, multiple surgeons	MODERATE
Kantor 2018	+	-	Prospective data collection	+	+	Multicenter in one country	LOW
Faraj 2019	+	-	Retrospective chart reviews, data collected by one investigator	-	-	One center, multiple surgeons	HIGH
Cao 2020	+	-	Retrospective chart reviews, data collected by one investigator	+	-	One center, multiple surgeons	HIGH
Zettervall 2020	+	-	Prospective data collection	+	+	Multicenter in one country	LOW
Feng 2014	+	-	Retrospective chart reviews, data collected by one investigator	-	-	One center, multiple surgeons	HIGH
**Pancreaticoduodenectomy, open, with venous resection**							
Ravikumar 2014	+	-	Retrospective chart reviews, data collected by one investigator	-	-	Multicenter in one country	HIGH
Kantor 2018	+	-	Prospective data collection	+	+	Multicenter in one country	LOW
Kantor 2018	+	-	Prospective data collection	+	+	Multicenter in one country	LOW
Snyder 2019	+	+	Retrospective duplicate chart reviews with good documentation of agreement between reviewers	+	+	One center, multiple surgeons	VERY LOW
Zettervall 2020	+	-	Prospective data collection	+	+	Multicenter in one country	LOW
**Gastrectomy, laparoscopic**							
Sexton 2008	+	-	Retrospective chart reviews, data collected by one investigator	-	-	One center, multiple surgeons	HIGH
Saka 2010	+	+	Administrative database information	-	-	One center, multiple surgeons	HIGH
Mamidanna 2013	+	-	Administrative database information	-	+	Multicenter in one country	HIGH
Son 2014	+	-	Retrospective chart reviews, data collected by one investigator	-	-	One center, multiple surgeons	HIGH

Glenn 2015	+	-	Administrative database information	+	-	Multicenter in one country	HIGH
Suda 2015	-	+	Retrospective chart reviews, data collected by one investigator	+	+	One center, multiple surgeons	MODERATE
Chen 2016	+	-	Prospective data collection	+	-	One center, multiple surgeons	MODERATE
Chen 2016	+	-	Prospective data collection	+	-	One center, multiple surgeons	MODERATE
Han 2016	-	-	Retrospective duplicate chart reviews without documentation of agreement between reviewers	+	+	One center, multiple surgeons	HIGH
Nakauchi 2016	-	-	Retrospective chart reviews, data collected by one investigator	+	+	One center, multiple surgeons	HIGH
Ntutumu 2016	+	-	Administrative database information	-	+	One center, multiple surgeons	HIGH
Wang 2017	-	-	Retrospective chart reviews, data collected by one investigator	+	+	Single surgeon series	HIGH
Hiki 2018	+	-	Prospective data collection	+	-	Multicenter in one country	MODERATE
Osaki 2018	+	+	Retrospective chart reviews, data collected by one investigator	+	+	One center, multiple surgeons	LOW
Shimada 2018	+	-	Retrospective chart reviews, data collected by one investigator	+	+	One center, multiple surgeons	MODERATE
Xu 2019	-	-	Retrospective chart reviews, data collected by one investigator	-	+	One center, multiple surgeons	HIGH
Alzahrani 2020	+	-	Prospective data collection	+	+	One center, multiple surgeons	LOW
Sakamoto 2020	+	-	Administrative database information	+	-	Multicenter in one country	HIGH
Shibasaki 2020	+	+	Retrospective chart reviews, data collected by one investigator	+	+	One center, multiple surgeons	LOW
**Gastrectomy, robotic**							
Song 2009	+	-	Retrospective chart reviews, data collected by one investigator	-	-	One center, multiple surgeons	HIGH
Son 2014	+	-	Retrospective chart reviews, data collected by one investigator	-	-	One center, multiple surgeons	HIGH
Glenn 2015	+	-	Administrative database information	+	-	Multicenter in one country	HIGH
Suda 2015	-	+	Retrospective chart reviews, data collected by one investigator	+	+	One center, multiple surgeons	MODERATE
Nakauchi 2016	-	-	Retrospective chart reviews, data collected by one investigator	+	+	One center, multiple surgeons	HIGH
Alhossaini 2019	+	-	Retrospective chart reviews, data collected by one investigator	+	-	One center, multiple surgeons	HIGH
Okabe 2019	+	-	Prospective data collection	+	-	Multicenter in one country	MODERATE

Shibasaki 2020	+	+	Retrospective chart reviews, data collected by one investigator	+	+	One center, multiple surgeons	LOW
**Gastrectomy, open**							
Park 2005	+	-	Prospective data collection	-	-	One center, multiple surgeons	HIGH
Pedrazzani 2007	+	-	Prospective data collection	-	+	One center, multiple surgeons	MODERATE
Lamb 2008	+	-	Prospective data collection	-	+	One center, multiple surgeons	MODERATE
Oh 2009	+	-	Prospective data collection	-	-	One center, multiple surgeons	HIGH
Sah 2009	-	-	Retrospective chart reviews, data collected by one investigator	-	-	One center, multiple surgeons	HIGH
Saka 2010	+	+	Administrative database information	-	-	One center, multiple surgeons	HIGH
Mamidanna 2013	+	-	Administrative database information	-	+	Multicenter in one country	HIGH
Papenfuss 2014	+	-	Prospective data collection	-	+	Multicenter in one country	MODERATE
Glenn 2015	+	-	Administrative database information	+	-	Multicenter in one country	HIGH
Han 2016	-	-	Retrospective duplicate chart reviews without documentation of agreement between reviewers	+	+	One center, multiple surgeons	HIGH
Chen 2017	+	-	Prospective data collection	+	-	One center, multiple surgeons	MODERATE
Kung 2017	+	-	Prospective data collection	+	+	Multicenter in one country	LOW
Hiki 2018	+	-	Prospective data collection	+	-	Multicenter in one country	MODERATE
Xu 2019	-	-	Retrospective chart reviews, data collected by one investigator	-	+	One center, multiple surgeons	HIGH
Sakamoto 2020	+	-	Administrative database information	+	-	Multicenter in one country	HIGH
**Gastrectomy, laparoscopic, subtotal**							
Sexton 2008	+	-	Retrospective chart reviews, data collected by one investigator	-	-	One center, multiple surgeons	HIGH
Chen 2016	+	-	Prospective data collection	+	-	One center, multiple surgeons	MODERATE
Hiki 2018	+	-	Prospective data collection	+	-	Multicenter in one country	MODERATE
Shimada 2018	+	-	Retrospective chart reviews, data collected by one investigator	+	+	One center, multiple surgeons	MODERATE
**Gastrectomy, laparoscopic, total**							
Son 2014	+	-	Retrospective chart reviews, data collected by one investigator	-	-	One center, multiple surgeons	HIGH
Chen 2016	+	-	Prospective data collection	+	-	One center, multiple surgeons	MODERATE

Wang 2017	-	-	Retrospective chart reviews, data collected by one investigator	+	+	Single surgeon series	HIGH
Sakamoto 2020	+	-	Administrative database information	+	-	Multicenter in one country	HIGH
**Gastrectomy, robotic, total**							
Son 2014	+	-	Retrospective chart reviews, data collected by one investigator	-	-	One center, multiple surgeons	HIGH
**Gastrectomy, open, subtotal**							
Park 2005	+	-	Prospective data collection	-	-	One center, multiple surgeons	HIGH
Pedrazzani 2007	+	-	Prospective data collection	-	+	One center, multiple surgeons	MODERATE
Sah 2009	-	-	Retrospective chart reviews, data collected by one investigator	-	-	One center, multiple surgeons	HIGH
Saka 2010	+	+	Administrative database information	-	-	One center, multiple surgeons	HIGH
Papenfuss 2014	+	-	Prospective data collection	-	+	Multicenter in one country	MODERATE
Hiki 2018	+	-	Prospective data collection	+	-	Multicenter in one country	MODERATE
**Gastrectomy, open, total**							
Park 2005	+	-	Prospective data collection	-	-	One center, multiple surgeons	HIGH
Oh 2009	+	-	Prospective data collection	-	-	One center, multiple surgeons	HIGH
Saka 2010	+	+	Administrative database information	-	-	One center, multiple surgeons	HIGH
Papenfuss 2014	+	-	Prospective data collection	-	+	Multicenter in one country	MODERATE
Chen 2017	+	-	Prospective data collection	+	-	One center, multiple surgeons	MODERATE
Sakamoto 2020	+	-	Administrative database information	+	-	Multicenter in one country	HIGH
**Gastric bypass, laparoscopic**							
Kothari 2007	+	+	Prospective data collection	-	+	One center, multiple surgeons	LOW
Rabl 2011	+	+	Prospective data collection	-	+	Multicenter in one country	LOW
Benizri 2013	+	-	Prospective data collection	+	+	One center, multiple surgeons	LOW
Woo 2013	+	+	Prospective data collection	+	+	One center, multiple surgeons	VERY LOW
Inaba 2018	+	-	Prospective data collection	+	+	Multicenter in one country	LOW
Thereaux 2018	+	+	Administrative database information	+	+	Multicenter in one country	LOW

Dugan 2020	+	-	Prospective data collection	+	+	Multicenter in one country	LOW
Gambhir 2020	+	-	Prospective data collection	+	+	Multinational	LOW
Sada 2020	+	+	Prospective data collection	+	+	One center, multiple surgeons	VERY LOW
**Gastric bypass, robotic**							
Yu 2006	+	-	Administrative database information	-	-	One center, multiple surgeons	HIGH
Ayloo 2011	+	-	Retrospective chart reviews, data collected by one investigator	-	+	Single surgeon series	HIGH
Benizri 2013	+	-	Prospective data collection	+	+	One center, multiple surgeons	LOW
Myers 2013	+	-	Retrospective chart reviews, data collected by one investigator	+	+	Single surgeon series	HIGH
Tieu 2013	+	-	Administrative database information	-	+	Multicenter in one country	HIGH
Ayloo 2016	-	-	Retrospective chart reviews, data collected by one investigator	+	-	Single surgeon series	HIGH
Acevedo 2020	+	-	Prospective data collection	+	+	Multicenter in one country	LOW
**Gastric bypass, open**							
Fernandez Jr 2004	+	-	Prospective data collection	-	-	One center, multiple surgeons	HIGH
Cotter 2005	-	+	Retrospective chart reviews, data collected by one investigator	-	-	Single surgeon series	HIGH
Abou-Nukta 2006	-	-	Retrospective chart reviews, data collected by one investigator	-	-	One center, multiple surgeons	HIGH
Gargiulo 2006	-	-	Retrospective chart reviews, data collected by one investigator	-	-	One center, multiple surgeons	HIGH
Gargiulo 2007	+	+	Retrospective chart reviews, data collected by one investigator	-	+	One center, multiple surgeons	MODERATE
Nguyen 2007	+	-	Administrative database information	-	+	Multicenter in one country	HIGH
Martins-Filho 2008	+	-	Retrospective chart reviews, data collected by one investigator	-	+	One center, multiple surgeons	HIGH
Weller 2008	+	-	Administrative database information	-	-	Multicenter in one country	HIGH
Caruana 2009	+	+	Retrospective chart reviews, data collected by one investigator	-	+	One center, multiple surgeons	MODERATE
Consortium Longitudinal Assessment of Bariatric Surgery, Flum 2009	+	-	Prospective data collection	-	+	Multicenter in one country	MODERATE
Slotman 2010	-	+	Retrospective chart reviews, data collected by one investigator	-	-	Single surgeon series	HIGH
Finks 2011	+	-	Prospective data collection	-	+	Multicenter in one country	MODERATE

Hutter 2011	+	-	Prospective data collection	-	+	Multicenter in one country	MODERATE
Rabl 2011	+	+	Prospective data collection	-	+	Multicenter in one country	LOW
Froehling 2012	-	-	Administrative database information	-	+	Multicenter in one country	HIGH
Masoomi 2012	+	-	Administrative database information	-	-	Multicenter in one country	HIGH
Santo 2013	+	+	Retrospective chart reviews, data collected by one investigator	-	+	One center, multiple surgeons	MODERATE
Lidor 2014	-	-	Prospective data collection	-	+	Multicenter in one country	HIGH
Nielsen 2018	+	-	Prospective data collection	+	+	Multicenter in one country	LOW
**Sleeve gastrectomy, laparoscopic**							
Woo 2013	+	+	Prospective data collection	+	+	One center, multiple surgeons	VERY LOW
Alsina 2014	+	+	Prospective data collection	+	+	Multinational	VERY LOW
Biertho 2014	+	+	Prospective data collection	-	+	One center, multiple surgeons	LOW
Sakran 2016	+	+	Prospective data collection	-	+	One center, multiple surgeons	LOW
Villagran 2016	+	+	Prospective data collection	+	-	One center, multiple surgeons	LOW
Moradian 2017	+	+	Retrospective chart reviews, data collected by one investigator	+	+	One center, multiple surgeons	LOW
Brunetti 2018	+	+	Prospective data collection	+	+	Single surgeon series	LOW
Guerrier 2018	+	-	Prospective data collection	+	+	Multicenter in one country	LOW
Inaba 2018	+	-	Prospective data collection	+	+	Multicenter in one country	LOW
Nimeri 2018	+	+	Prospective data collection	+	+	One center, multiple surgeons	VERY LOW
Thereaux 2018	+	+	Administrative database information	+	+	Multicenter in one country	LOW
Abuoglu 2019	+	+	Prospective data collection	+	-	One center, multiple surgeons	LOW
AlKhaldi 2019	+	+	Retrospective chart reviews, data collected by one investigator	+	+	One center, multiple surgeons	LOW
Dugan 2020	+	-	Prospective data collection	+	+	Multicenter in one country	LOW
Gambhir 2020	+	-	Prospective data collection	+	+	Multinational	LOW
Johari 2020	+	-	Prospective data collection	+	+	One center, multiple surgeons	LOW
**Sleeve gastrectomy, robotic**							
Romero 2013	+	-	Retrospective chart reviews, data collected by one investigator	+	-	One center, multiple surgeons	HIGH

Ecker 2016	+	-	Administrative database information	+	+	One center, multiple surgeons	MODERATE
Moon 2018	+	-	Prospective data collection	+	-	Multicenter in one country	MODERATE
Acevedo 2020	+	-	Prospective data collection	+	+	Multicenter in one country	LOW

Articles are reported by procedure, so duplicate information from same study appears in this table.

14. Prophylaxis in individual studies in upper-gastrointestinal and hepatopancreatobiliary surgery

Reference	Total patients	Mechanical prophylaxis			Antiplatelet drugs			Anticoagulants		
	n	%	Type	Duration in days	%	Type	Duration in days	%	Type	Duration in days
**Distal pancreatectomy, laparoscopic**										
Anonsen 2015	69									
Nakamura 2015	902									
Sulpice 2015	347									
Kwon 2016	111									
Dokmak 2017	165									
Daniel 2018	1789									
Chen 2019	353									
**Distal pancreatectomy, robotic**										
Zureikat 2013	83									
**Distal pancreatectomy, open**										
Yekebas 2007	116									
Lee 2008	180									
Dedania 2013	70	100†		6†				100†	LMWH†	6†
Nakamura 2015	1108									
Sulpice 2015	2406									
Daniel 2018	1790									
Boone 2019	55		IPC					98	LMWH	
Mussle 2020	191	100†	GCS					100	LMWH/UFH	28
**Distal pancreatectomy, laparoscopic, benign**										
Daniel 2018	1030									
Chen 2019	116									
**Distal pancreatectomy, laparoscopic, malignant**										

Sulpice 2015	347								
Daniel 2018	759								
**Distal pancreatectomy, open, benign**									
Daniel 2018	655								
**Distal pancreatectomy, open, malignant**									
Dedania 2013	70	100†		6†			100†	LMWH†	6†
Sulpice 2015	2406								
Daniel 2018	1135								
Boone 2019	55		IPC				98	LMWH	
**Liver resection, laparoscopic**									
Vibert 2006	84								
Dagher 2009	210								
Abu Hilal 2010	80								
Dagher 2010	163								
Kazaryan 2010	139						100	LMWH	
Bhojani 2012	57	100	IPC, GCS				100	Unspecified	
Soubrane 2014	351								
Cauchy 2015	223								
Fuks 2016	226								
Cipriani 2018	698								
Sucandy 2018	831								
Ainoa 2020	84	100†	GCS		38†	ASA, Clopidogrel, Ticagrelor, Dipyridamole†	100†	LMWH†	27†
Triantafyllidis 2020	431								
Stiles 2017	859								
**Liver resection, robotic**									
Kingham 2016	64								
Daskalaki 2017	67								

Sucandy 2020	77								
**Liver resection, open**									
Stewart 2004	137	100†	IPC†		0†	None†	100	LMWH	7
Zhou 2007	81								
Lee 2009	248								
Lordan 2009	469								
Andres 2011	689							UFH/LMWH	
Nobili 2012	555	0	none						
Tzeng 2012	5651								
Barbas 2013	1281						100†	UFH/LMWH†	10†
Aramaki 2014	539	100†	IPC†	1	0†	None	0†	None†	0
Nathan 2014	2147	100	IPC				60	UFH/LMWH	
Bagante 2016	2452								
de'Angelis 2016	329								
Fuks 2016	988								
Yokoo 2016	14970								
Khandoga 2017	184								
Singh 2017	86	100	IPC	7	0		0		0
Kron 2019	211	100†		14†	0†		100†	LMWH†	14†
Ainoa 2020	428	100†	GCS		10†	ASA, Clopidogrel, Ticagrelor, Dipyridamole†	100†	LMWH†	27†
Snyder 2020	388								
Tahkola 2020	73								
**Liver resection, laparoscopic, minor**									
Soubrane 2014	351								
Stiles 2017	859								
Ainoa 2020	78	100†	GCS		38†	ASA, Clopidogrel, Ticagrelor, Dipyridamole†	100†	LMWH†	27†
**Liver resection, laparoscopic, major**									

Dagher 2009	210									
Cauchy 2015	223									
Fuks 2016	226									
**Liver resection, open, minor**										
Tzeng 2012	3376									
Aramaki 2014	100†		1†	0†	None†	0†	0†	None†		
Ainoa 2020	250	100†	GCS		10†	ASA, Clopidogrel, Ticagrelor, Dipyridamole†		100†	LMWH†	27†
**Liver resection, open, major**										
Zhou 2007	81									
Tzeng 2012	1690									
de'Angelis 2016	329									
Fuks 2016	988									
Singh 2017	86	100	IPC		0			0		
Kron 2019	211	100†		14†	0†			100†	LMWH†	14†
Ainoa 2020	178	100†	GCS		10†	ASA, Clopidogrel, Ticagrelor, Dipyridamole†		100†	LMWH†	27†
Snyder 2020	388									
Tahkola 2020	73									
**Pancreaticoduodenectomy, laparoscopic**										
Kendrick 2010	62							100	UFH	7
Dokmak 2017	70									
Yu 2018	191									
Chen 2019	186									
Song 2020	500	100	GCS					100	LMWH	2
Vining 2020	407									
Wang 2020	550									

Boone 2019	200		IPC		98	LMWH	
**Pancreaticoduodenectomy, robotic**							
Zureikat 2013	132						
Rosemurgy 2019	155	100†	IPC†				
Vining 2020	498						
**Pancreaticoduodenectomy, open**							
Martignoni 2001	257						
Adam 2004	301						
Balachandran 2004	218						
Tien 2005	402						
Turrini 2005	172						
Koukoutsis 2006	362						
Blanc 2007	411				100	UFH	
Yekebas 2007	1141						
Tien 2008	283						
Wei 2009	628						
Pandanaboyana 2010	67				100†	LMWH†	24†
Kneuertz 2011	220						
Mañas-Gómez 2011	107				100	LMWH	
Ricci 2012	113						
Enomoto 2014	9830						
Feng 2014	840						
Kokudo 2014	187						
Ravikumar 2014	1070						

Flis 2016	111							
Soriano 2016	67							
Fujikawa 2018	100	100	IPC, GCS	31	aspirin	26	UFH	
Kantor 2018	9235							
Boone 2019	327		GCS			98	LMWH	
Faraj 2019	300	100	IPC			40	LMWH	
Mataki 2019	315							
Rystedt 2019	1864				Unknown	100†	LMWH†	
Snyder 2019	120			100	aspirin	100	LMWH	28
Cao 2020	151							
Komokata 2020	77	100	IPC, GCS	27	mainly aspirin	26	Other/UFH	90
Mussle 2020	699	100	GCS			100	LMWH/UFH	28
Tahkola 2020	218							
Vining 2020	12612							
**Pancreaticoduodenectomy, laparoscopic, without venous resection**								
Dokmak 2017	70							
Kendrick 2010	62					100	UFH	7
Yu 2018	191							
Wang 2020	473							
**Pancreaticoduodenectomy, laparoscopic, with venous resection**								
Wang 2020	77							
**Pancreaticoduodenectomy, open, without venous resection**								
Turrini 2005	172							
Blanc 2007	411					100	UFH	

Ravikumar 2014	840							
Flis 2016	111							
Kantor 2018	8258							
Faraj 2019	300	100	IPC			40	LMWH nr nr	
Cao 2020	151							
Zettervall 2020	2566							
Feng 2014	840							

Pancreaticoduodenectomy, open, with venous resection

Ravikumar 2014	230							
Kantor 2018	640							
Kantor 2018	224							
Snyder 2019	120			100	aspirin	83	LMWH	28
Zettervall 2020	436							

Gastrectomy, laparoscopic

Sexton 2008	61							
Saka 2010	178	100	IPC, GCS			100	UFH	2
Mamidanna 2013	480							
Son 2014	58							
Glenn 2015	789							
Suda 2015	438	100†	IPC, GCS†			100†	LMWH†	3†
Chen 2016	253							
Chen 2016	379							
Han 2016	1355							
Nakauchi 2016	437							
Ntutumu 2016	1205							

Wang 2017	1657								
Hiki 2018	1067								
Osaki 2018	129	99	IPC, GCS	3			4	UFH/DOAC	
Shimada 2018	243				8				
Xu 2019	430								
Alzahrani 2020	207								
Sakamoto 2020	13187								
Shibasaki 2020	1042	100†	IPC, GCS†	2†	0	None†	80†		5†
**Gastrectomy, robotic**									
Song 2009	100								
Son 2014	51								
Glenn 2015	223								
Suda 2015	88	100†	IPC, GCS†				100†	LMWH†	3†
Nakauchi 2016	84								
Alhossaini 2019	288								
Okabe 2019	115								
Shibasaki 2020	359	100†		2†	0†	None†	80†	LMWH†	5†
**Gastrectomy, open**									
Park 2005	548								
Pedrazzani 2007	310								
Lamb 2008	180								
Oh 2009	410								
Sah 2009	809								
Saka 2010	3014	100	IPC, GCS	2			100	UFH	2
Mamidanna 2013	10233								

Papenfuss 2014	2580						
Glenn 2015	8585						
Han 2016	3256						
Chen 2017	124						
Kung 2017	1101						
Hiki 2018	1067						
Xu 2019	768						
Sakamoto 2020	45502						
**Gastrectomy, laparoscopic, subtotal**							
Sexton 2008	61						
Chen 2016	379						
Hiki 2018	1067						
Shimada 2018	243			8			
**Gastrectomy, laparoscopic, total**							
Son 2014	58						
Chen 2016	253						
Wang 2017	1657						
Sakamoto 2020	13187						
**Gastrectomy, robotic, total**							
Son 2014	51						
**Gastrectomy, open, subtotal**							
Park 2005	403						
Pedrazzani 2007	310						
Sah 2009	809						
Saka 2010	2111	100	IPC, GCS		100	UFH	2
Papenfuss 2014	1581						

Hiki 2018	1067							
**Gastrectomy, open, total**								
Park 2005	145							
Oh 2009	410							
Saka 2010	903	100	IPC, GCS			100	UFH	2
Papenfuss 2014	999							
Chen 2017	124							
Sakamoto 2020	45502							
**Gastric bypass, laparoscopic**								
Kothari 2007	476	100	IPC	2		100	LMWH/ UFH	2
Rabl 2011	644							
Benizri 2013	100							
Woo 2013	55	100	IPC, GCS	3		97	LMWH	14
Inaba 2018	128349							
Thereaux 2018	33611					74	LMWH	
Dugan 2020	117599							
Gambhir 2020	102146							
Sada 2020	561	100				100†	UFH†	
**Gastric bypass, robotic**								
Yu 2006	100							
Ayloo 2011	90							
Benizri 2013	100							
Myers 2013	100							
Tieu 2013	1100							
Ayloo 2016	146	100	IPC			100	LMWH	
Acevedo 2020	5817							
**Gastric bypass, open**								

Fernandez Jr 2004	1431								
Cotter 2005	107	1	IPC	4			100	UFH	4
Abou-Nukta 2006	1225	100	IPC				100	LMWH	
Gargiulo 2006	606	100	IPC				100	LMWH	
Gargiulo 2007	193						100	LMWH	
Nguyen 2007	6065								
Martins-Filho 2008	135								
Weller 2008	4883								
Caruana 2009	1652	100	IPC	0	0	aspirin	100	UFH/UFH	6
Consortium Longitudinal Assessment of Bariatric Surgery, Flum 2009	437								
Slotman 2010	61	100	IPC			none	100	LMWH	22
Finks 2011	1092								
Hutter 2011	988								
Rabl 2011	78								
Froehling 2012	228	100	IPC				100	LMWH/UFH/Warfarin	
Masoomi 2012	42591								
Santo 2013	538		GCS				100	LMWH	21
Lidor 2014	5282								
Nielsen 2018	503								

Sleeve gastrectomy, laparoscopic

Woo 2013	132	100	IPC, GCS				97	LMWH	14
Alsina 2014	100	100	IPC				100	LMWH	30
Biertho 2014	378						100	LMWH	
Sakran 2016	3003	100	IPC	14			100	LMWH	14
Villagran 2016	1236								
Moradian 2017	50	100	IPC	1			100	LMWH	1

Brunetti 2018	60	100	IPC					100	UFH/LMWH	
Guerrier 2018	47982									
Inaba 2018	30257									
Nimeri 2018	527	100	IPC					100	UFH/LMWH	2
Thereaux 2018	62266							79	LMWH	35
Abuoglu 2019	302	100	IPC	1				100	LMWH	15
AlKhaldi 2019	187							100	LMWH	21
Dugan 2020	312065									
Gambhir 2020	266886									
Johari 2020	259									
**Sleeve gastrectomy, robotic**										
Romero 2013	134									
Ecker 2016	411									
Moon 2018	740									
Acevedo 2020	12912									

Mechanical thromboprophylaxis included: antithrombosis stockings, intermittent pneumatic compression devices, and foot-pumps
Aspirin or other antiplatelet drugs included: aspirin, clopidogrel, prasugrel, ticlopidine, dipyridamole, ticagrelor, cilostazol, tirofiban, vorapaxar as well as thromboxane inhibitors, thromboxane synthase inhibitors, thromboxane receptor antagonists, and terutroban
Anticoagulants included: warfarin, low molecular weight heparin, low dose unfractionated heparin, dabigatran, apixaban, betrixaban, edoxaban, rivaroxaban, fondaparinux, danaparoid and lepirudin
Blank spaces represent no information (not provided by paper or by author correspondence).
Duration in days is expressed as mean or median.
GCS=graduated compression stockings; IPC= intermittent pneumatic compression (includes “intermittent compression device, sequential compression device, pneumatic compression device, pneumatic compression stockings, pneumatic compression boots”); LMWH= low molecular weight heparin; UFH= unfractionated heparin.
† Author provided this information.
§Follow up time of complications was not available from the article or author correspondence. We assumed a follow up time of 30 days.

15. Postoperative risk of symptomatic VTE and bleeding in individual studies in upper-gastrointestinal and hepatopancreatobiliary surgery

Reference	Total patients	Follow-up time	Reported VTE					Reported Bleeding			Baseline cumulative incidence at 4 weeks		
	n	Days	Fatal PE	Non-Fatal PE	DVT	VTE total* (excluding SVT)	SVT	Fatal Bleeding	Bleeding requiring reintervention	Transfusion	VTE at 4 weeks (%)	Baseline bleeding requiring reintervention at 4 weeks (%)	Bleeding requiring transfusion at 4 weeks (%)
**Distal pancreatectomy, laparoscopic**													
Anonsen 2015	69	30§				0			1		0%	1%	
Nakamura 2015	902	30§							12			1.3%	
Sulpice 2015	347	30§								22			4.2%
Kwon 2016	111	30											
Dokmak 2017	165	30§	0					0					
Daniel 2018	1,789	30				48					2.6%		4,5%
Chen 2019	353	30§					3						
**Distal pancreatectomy, robotic**													
Zureikat 2013	83	30						0	1			0.8%	
**Distal pancreatectomy, open**													
Yekebas 2007	116	30						2	4			2.3%	
Lee 2008	180	30§					4						
Dedania 2013	70	30	0†			3		0†	0		5.5%	0%	
Nakamura 2015	1,108	30§							5			0.4%	
Sulpice 2015	2,406	30§								395			10.7%
Daniel 2018	1,790	30				61					5.2%		8.2%
Boone 2019	55	90				10					16%		
Mussle 2020	191	90†	1†	3†		13‡		4†	3†		7.4%	0.9%	
**Distal pancreatectomy, laparoscopic, benign**													
Daniel 2018	1,030	30				23					2.2%		4.2%
Chen 2019	116	30§					0						

**Distal pancreatectomy, laparoscopic, malignant**												
Sulpice 2015	347	30§							22			4.2%
Daniel 2018	759	30				26				3.4%		5%
**Distal pancreatectomy, open, benign**												
Daniel 2018	655	30				10				2.3%		7.8%
**Distal pancreatectomy, open, malignant**												
Dedania 2013	70	30†	0†			3		0†	0		5.5%	0%	
Sulpice 2015	2,406	30§							395			10.7%
Daniel 2018	1,135	30				51				6.8%		8.4%
Boone 2019	55	90				10				16%		
**Liver resection, laparoscopic**												
Vibert 2006	84	30§						1	1	2		0.8%	1.6%
Dagher 2009	210	30§	1					0					
Abu Hilal 2010	80	30§						0	1	0		0.9%	0%
Dagher 2010	163	30§						0	4			1.7%	
Kazaryan 2010	139	30§							2			1%	
Bhojani 2012	57	30§	0					0		11			13.4%
Soubrane 2014	351	30§					3			12			2%
Cauchy 2015	223	30	0					1		29			7.6%
Fuks 2016	226	90		3		12‡				30	4.4%		7.7%
Cipriani 2018	698	30		1		4‡				56	0.9%		5.5%
Sucandy 2018	831	30§				5				24	0.7%		2%
Ainoa 2020	84	30	0†	0†	0†	0†	0†	0†	0†	3†	0%	0%	2.3%
Triantafyllidis 2020	431	90				4				28	0.8%		3.8%
Stiles 2017	859	30			7	9‡					1.2%		2.8%
**Liver resection, robotic**												
Kingham 2016	64	30§		0	1	1‡					2.2%		

Daskalaki 2017	67	30§							0			0%	
Sucandy 2020	77	30				0					0%		
**Liver resection, open**													
Stewart 2004	137	30	0	3	0†	3†	1	1	3†		3.6%	1.5%	
Zhou 2007	81	30§					13		5			5.5%	
Lee 2009	248	30							1			0.4%	17.5%
Lordan 2009	469	30				2				25	0.9%		3.7%
Andres 2011	689	30	0			12		2			2.6%		
Nobili 2012	555	30§		16		63‡				64	20.4%		7.9%
Tzeng 2012	5,651	30				162			25		4,9%	0,3%	0,3%
Barbas 2013	1,281	90			4	4‡			24		0.3%	1.1%	
Aramaki 2014	539	30	0			2		1	7		0.4%	1.3%	25%
Nathan 2014	2,147	30				55					3.2%		
Bagante 2016	2,452	30		38	60	95‡					6.5%		9.9%
de'Angelis 2016	329					0‡	9	1					
Fuks 2016	988					0‡				288			17%
Yokoo 2016	14,970	30	9	24		104‡				606	0.9%		3.6%
Khandoga 2017	184	30		5		20‡	2				21.3%		
Singh 2017	86	30§		0	0	0	0				0%		
Kron 2019	211	90†	0	2	0	2†	2	4	9		0.9%	2.5%	8.7%
Ainoa 2020	428	30	0†	23†	1†	28†	1†	0†	4†	67†	14.1%	0.6%	10.3%
Snyder 2020	388					0‡							
Tahkola 2020	73	30	0	1	0	1‡		0	1		2.6%	0.9%	
**Liver resection, laparoscopic, minor**													
Soubrane 2014	351	30§					3			12			2%
Stiles 2017	859	30			7	9‡					1.6%		2.8%
Ainoa 2020	78	30	0†	0†	0†	0†	0†	0†	0†	3†	0%	0%	2.5%
**Liver resection, laparoscopic, major**													

Dagher 2009	210	30§	1					0					
Cauchy 2015	223	30	0					1		29			7.6%
Fuks 2016	226	90		3		12‡				30	4.4%		7.7%
**Liver resection, open, minor**													
Tzeng 2012	3,376	30				71			10		3.5%	0.2%	0.3%
Aramaki 2014	539	30†	0			2		1	7		0.4%	1.3%	4.6%
Ainoa 2020	250	30	0†	13†	0†	13†	0†	0†	2†	26†	11.2%	0.5%	17.6%
**Liver resection, open, major**													
Zhou 2007	81	30§					13		5			5.5%	
Tzeng 2012	1,690	30				79			12		7,9%	0,5%	0,5%
de'Angelis 2016	329	90	2	1		6‡	9	1			1.8%		
Fuks 2016	988	90		44		174‡				288	17.3%		17%
Singh 2017	86	30§		0	0	0	0				0%		
Kron 2019	211	90†	0	2	0	2†	2	4	9		0.9%	2.5%	8.7%
Ainoa 2020	178	30	0†	14†	1†	15†	1†	0†	2†	41†	18.2%	0.7%	24.7%
Snyder 2020	388	30		7	17	23‡					10.1%		
Tahkola 2020	73	30	0	1	0	1‡		0	1		2.6%	0.9%	
**Pancreaticoduodenectomy, laparoscopic**													
Kendrick 2010	62	30§			2	3‡			1		5.3%	1.1%	
Dokmak 2017	70	30§	1						14			13%	
Yu 2018	191	30§											
Chen 2019	186	30§					3						
Song 2020	500	30§					0	1	12	10		1.8%	1.5%
Vining 2020	407	30		7	14	20‡					7.7%		8.1%
Wang 2020	550	90				18					2.2%		3.2%

Boone 2019	200	90				42	17				9.9%		
**Pancreaticoduodenectomy, robotic**													
Zureikat 2013	132	30							4			2.1%	
Rosemurgy 2019	155	30	0	0	0	0		0		3	0%		1.6%
Vining 2020	498	30		10	16	25‡					7.3%		4.8%
**Pancreaticoduodenectomy, open**													
Martignoni 2001	257	30§						0		6			1.6%
Adam 2004	301	30§				6		2			3.8%		
Balachandran 2004	218	30§						15		30			12.4%
Tien 2005	402	30						5					
Turrini 2005	172	30	1					9					
Koukoutsis 2006	362	30						15	23			4.3%	
Blanc 2007	411	30						3	23	9		3.8%	1.5%
Yekebas 2007	1,141	30						9					
Tien 2008	283	30§						1					
Wei 2009	628	30§						10					
Pandanaboyana 2010	67	30						3	4			3.9%	
Kneuertz 2011	220	30§								102			31.7%
Mañas-Gómez 2011	107	30§						2					
Ricci 2012	113	30							8	4		4.6%	2.3%
Enomoto 2014	9,830	30		0	39	38‡				0	0.6%		0%
Feng 2014	840	30§						12					
Kokudo 2014	187	30		13		51‡					52.6%		
Ravikumar 2014	1,070	30§								52			3.3%

Flis 2016	111	30§	1				0						
Soriano 2016	67	30		1		4‡	1		0		12.9%	0%	
Fujikawa 2018	100	30							0			0%	
Kantor 2018	9,235	30			204	262‡					4.7%		8.4%
Boone 2019	327	90				60					15%		
Faraj 2019	300	30§	0		1	1‡		0		39	0.4%		12.3%
Mataki 2019	315	30						3	11			3%	
Rystedt 2019	1,864	30§								512			24.7%
Snyder 2019	120	30					9		1			0.5%	
Cao 2020	151	30§	1	1		5‡		3			7.2%		
Komokata 2020	77	30§				4		1		8	6.1%		9%
Mussle 2020	699	90†	4†	17†		72‡		19†	30†		10,6%	2,4%	
Tahkola 2020	218	30		1	5	6‡		0	7		5.1%	2.2%	
Vining 2020	12,612	30		143	361	488‡					6.4%		8.2%
**Pancreaticoduodenectomy, laparoscopic, without venous resection**													
Dokmak 2017	70	30§	1						14			13%	
Kendrick 2010	62	30§			2	3‡			1		5.3%	1.1%	
Yu 2018	191	30§											
Wang 2020	473	90				13					1.9%		2.6%
**Pancreaticoduodenectomy, laparoscopic, with venous resection**													
Wang 2020	77	90				5					4.4%		6.9%
**Pancreaticoduodenectomy, open, without venous resection**													
Turrini 2005	172	30	1					9	16			6.3%	
Blanc 2007	411	30						3	23	9		3.8%	1.5%

Ravikumar 2014	840	30§								40			6%
Flis 2016	111	30§	1				0						
Kantor 2018	8,258	30			159	204‡					4.1%		7.6%
Faraj 2019	300	30§	0		1	1‡		0	15	39	0.4%	4.5%	11.7%
Cao 2020	151	30§	1	1		5‡		3			3.3%		
Zettervall 2020	2,566	30				85					5.4%		
Feng 2014	840	30§						12	59			6.3%	
**Pancreaticoduodenectomy, open, with venous resection**													
Ravikumar 2014	230	30§					7			12			3.5%
Kantor 2018	640	30			29	37‡					9.5%		16.1%
Kantor 2018	224	30			16	21‡					12.4%		17.3%
Snyder 2019	120	30					9		1			0.5%	25.7%
Zettervall 2020	436	30				27					10,3%		
**Gastrectomy, laparoscopic**													
Sexton 2008	61	30§			1	1‡			1		2.6%	1.1%	
Saka 2010	178	30§	0	0		0‡					0%		
Mamidanna 2013	480	30		3	1	4					1.4%		
Son 2014	58	30§								2			2.6%
Glenn 2015	789	30§				42					8.5%		
Suda 2015	438	30		2		8‡			3		2%	0.5%	
Chen 2016	253	30§		1	1	2‡					0,9%		
Chen 2016	379	30§	0	0		0‡					0%		
Han 2016	1,355	30					2						
Nakauchi 2016	437	30	0	2		8‡		1			2.1%		
Ntutumu 2016	1,205	30	0	1	2	3‡					0.3%		

Wang 2017	1,657	30	0			3		1			0.2%		
Hiki 2018	1,067	30§		1		4‡					0.4%		
Osaki 2018	129	7	0	0	0	0					0%		
Shimada 2018	243	30			1	1‡					0.6%		
Xu 2019	430	30	0		0	0‡		0	2		0%	0.3%	
Alzahrani 2020	207	30	0	0	0	0					0%		
Sakamoto 2020	13,187	30§		26		103‡				1,238	0.9%		6.5%
Shibasaki 2020	1,042	30	0†	2†		8‡		0†	4†		0.9%	0.3%	
**Gastrectomy, robotic**													
Song 2009	100	30§						0	1	1		0.7%	0.7%
Son 2014	51	30§						0					
Glenn 2015	223	30§				17					12.4%		
Suda 2015	88	30		1		4‡			0		4.9%	0%	
Nakauchi 2016	84	30	1	0		1‡		0			1.4%		
Alhossaini 2019	288	30§		2		8‡		2	0		3.2%	0%	
Okabe 2019	115	30§					1	0	0			0%	
Shibasaki 2020	359	30	1†	0†		1‡		0†	2		0.3%	0.4%	
**Gastrectomy, open**													
Park 2005	548	30§						0		19			2,6%
Pedrazzani 2007	310	30						0	2			0.4%	
Lamb 2008	180	30	0	0	0	0‡		1	1		0%	0.4%	
Oh 2009	410	30§	1	0		1‡					0.3%		
Sah 2009	809	30§			1	1‡					0.2%		
Saka 2010	3,014	30§	0	6		24‡					0.8%		
Mamidanna 2013	10,233	30		63	42	97					1.5%		

Papenfuss 2014	2,580	30		31	37	65‡					3.4%		
Glenn 2015	8,585	30§				421					8.4%		
Han 2016	3,256	90			0	0‡	12				0%		
Chen 2017	124	30§	0	0		0‡					0%		
Kung 2017	1,101	30		12		47‡					8.5%		
Hiki 2018	1,067	30§		0		0‡					0%		
Xu 2019	768	30	0		2	3‡		0	3		0.4%	0.3%	
Sakamoto 2020	45,502	30§		92		363‡				12,203	0.8%		20%
**Gastrectomy, laparoscopic, subtotal**													
Sexton 2008	61	30§			1	1‡			1		2.6%	1.1%	
Chen 2016	379	30§	0	0		0‡					0%		
Hiki 2018	1,067	30§		1		4‡					0.4%		
Shimada 2018	243	30			1	1‡					0.6%		
**Gastrectomy, laparoscopic, total**													
Son 2014	58	30§								2			2.6%
Chen 2016	253	30§		1	1	2‡					0,9%		
Wang 2017	1,657	30	0			3		1			0.2%		
Sakamoto 2020	13,187	30§		26		103‡				1,238	0.9%		6.5%
**Gastrectomy, robotic, total**													
Son 2014	51	30§						0					
**Gastrectomy, open, subtotal**													
Park 2005	403	30§						0		14			2.6%
Pedrazzani 2007	310	30						0	2			0.4%	
Sah 2009	809	30§			1	1‡					0.2%		
Saka 2010	2,111	30§	0	4		16‡					0.8%		
Papenfuss 2014	1,581	30		11	21	31‡					2.6%		

Hiki 2018	1,067	30§		0		0‡					0%		
**Gastrectomy, open, total**													
Park 2005	145	30§						0		5			2.6%
Oh 2009	410	30§	1	0		1‡					0.3%		
Saka 2010	903	30§	0	2		8‡					0.9%		
Papenfuss 2014	999	30		20	16	35‡					4.6%		
Chen 2017	124	30§	0	0		0‡					0%		
Sakamoto 2020	45,502	30§		92		363‡				12,203	0.8%		20%
**Gastric bypass, laparoscopic**													
Kothari 2007	476	30	0	1	0	1‡		0	4	17	0.2%	0.6%	2.7%
Rabl 2011	644	30							3	14		0.3%	1.6%
Benizri 2013	100	30				1		0	0		1.5%	0%	
Woo 2013	55	30	0	0	0	0	0	0	2	0	0%	2.5%	0%
Inaba 2018	128,349	30	0	208		821‡					0.7%		
Thereaux 2018	33,611	90				192					0.5%		
Dugan 2020	117,599	30		188		202			294		0.2%	0.2%	
Gambhir 2020	102,146					0‡							0.5%
Sada 2020	561	30†	0	1		4‡		0†	0†	0†	0.8%	0%	0%
**Gastric bypass, robotic**													
Yu 2006	100	30§	0	1		4‡					4.3%		
Ayloo 2011	90	30	0	1		4‡		0	0		4.7%	0%	
Benizri 2013	100	30				1		0	2		1.5%	1.4%	
Myers 2013	100	90	0	0		0‡		0	0	1	0%	0%	0.6%
Tieu 2013	1,100	90	0	2	3	5‡		0			0.3%		
Ayloo 2016	146	30§	0			2		0	0	1	1.6%	0%	0.5%
Acevedo 2020	5,817	30				38					0.7%		0.3%

**Gastric bypass, open**													
Fernandez Jr 2004	1,431	30§		17		67‡					6%		
Cotter 2005	107	30§	0	0	1	1‡					1.1%		
Abou-Nukta 2006	1,225	30§	1	11		44‡					4.6%		
Gargiulo 2006	606	30§	5	4		21‡		0			3.4%		
Gargiulo 2007	193	30	3	4		19‡					12.4%		
Nguyen 2007	6,065	30				42					0.9%		
Martins-Filho 2008	135	30	2	1	1	4‡				1	5.4%		0.5%
Weller 2008	4,883	90				13					0.2%		
Caruana 2009	1,652	63	1	5		21‡			0	20	1.1%	0%	0.8%
Consortium Longitudinal Assessment of Bariatric Surgery, Flum 2009	437	30				5					1.5%		
Slotman 2010	61	30§		0	0	0‡		0			0%		
Finks 2011	1,092	30				8					0.9%		
Hutter 2011	988	30		1	3	4‡			7		0.5%	0.5%	
Rabl 2011	78	30							0	2		0%	1.8%
Froehling 2012	228	28		2	4	6‡					3.4%		
Masoomi 2012	42,591	30§				192					0.6%		
Santo 2013	538	30	2	3		14‡		0	3	2	4.8%	0.4%	0.2%
Lidor 2014	5,282	30				52					1.3%		
Nielsen 2018	503	30				8					2%		1.3%
**Sleeve gastrectomy, laparoscopic**													
Woo 2013	132	30	0	0	0	0	0	0	2	0	0%	1%	0%
Alsina 2014	100	90			1	1‡	0	0	0		1.3%	0%	3.7%
Biertho 2014	378	30	0	0	1	1‡	1				0.3%		
Sakran 2016	3,003	30	0	0	1	1‡	4	1	13	23	0.1%	0.3%	0.5%
Villagran 2016	1,236	30§					5						

Moradian 2017	50	90	0	0	0	0					0%		
Brunetti 2018	60	30	0	0	0	0	0	0			0%		
Guerrier 2018	47,982	30		82	158	232‡					0.5%		0.5%
Inaba 2018	30,257	30		33		130‡					0.4%		
Nimeri 2018	527	30	0	0		4					0.8%		0.5%
Thereaux 2018	62,266	90				342					0.5%		
Abuoglu 2019	302	90	0	0	0	0		0	0		0%	0%	
AlKhaldi 2019	187	30		0	0	0		0	0	0	0%	0%	0%
Dugan 2020	312,065	30		256	538	770			120		0.2%	0%	
Gambhir 2020	266,886					0‡							0.3%
Johari 2020	259	30	0				2		2			0.5%	
**Sleeve gastrectomy, robotic**													
Romero 2013	134	35§		1		2	1		1		1.5%	0.6%	
Ecker 2016	411	30	0	2	2	4‡			2	16	1%	0.4%	1.9%
Moon 2018	740	30§					4						
Acevedo 2020	12,912	30				81					0.7%		0.2%

VTE=Venous thromboembolism, PE=Pulmonary embolism, DVT=Deep vein thrombosis, SVT= Splanchnic vein thrombosis.
Cumulative risks are given for the first four postoperative weeks.
Blank spaces represent no information (not provided by paper or by author correspondence).
§Follow up time of complications was not available from the article or author correspondence. We assumed a follow up time of 30 days as this was median reported follow up time in the eligible studies.
* Excluding SVT
† Authors provided value.
‡ Estimated VTE value

Balachandran 2004: Open pancreaticoduodenectomy: We did not include this study to the baseline risk analyses for bleeding requiring reoperation because of risk of bias but included it to baseline risk analyses for other outcomes.
De’Angelis 2016: Open liver resection: We did not include this study to the baseline risk analyses for VTE because of risk of bias but included it to baseline risk analyses for other outcomes.
Ecker 2016: Minimally-invasive sleeve gastrectomy: We did not include this study to the baseline risk analyses for VTE and bleeding requiring reoperation because of risk of bias but included it to baseline risk analyses for other outcomes.

Faraj 2019: Open pancreaticoduodenectomy: We did not include this study to the baseline risk analyses for bleeding requiring reoperation because of risk of bias but included it to baseline risk analyses for other outcomes.
Feng 2014: Open pancreaticoduodenectomy: We did not include this study to the baseline risk analyses for bleeding requiring reoperation because of risk of bias but included it to baseline risk analyses for other outcomes.
Fuks 2016: Open liver resection: We did not include this study to the baseline risk analyses for VTE because of risk of bias but included it to baseline risk analyses for other outcomes.
Gambhir 2020: Laparoscopic gastric bypass: We did not include this study to the baseline risk analyses for VTE because of overlapping population but included it to baseline risk analyses for other outcomes.
Gambhir 2020: Laparoscopic sleeve gastrectomy: We did not include this study to the baseline risk analyses for VTE because of overlapping population but included it to baseline risk analyses for other outcomes.
Komokata 2020: Open pancreaticoduodenectomy: We did not include this study to the baseline risk analyses for bleeding requiring reoperation because of risk of bias but included it to baseline risk analyses for other outcomes.
Mañas-Gómez 2011: Open pancreaticoduodenectomy: We did not include this study to the baseline risk analyses for bleeding requiring reoperation because of risk of bias but included it to baseline risk analyses for other outcomes.
Martignoni 2001: Open pancreaticoduodenectomy: We did not include this study to the baseline risk analyses for bleeding requiring reoperation because of risk of bias but included it to baseline risk analyses for other outcomes.
Reddy 2011: Open liver resection: We did not include this study to the baseline risk analyses for VTE because of risk of bias but included it to baseline risk analyses for other outcomes.
Romero 2013: Minimally-invasive sleeve gastrectomy: We did not include this study to the baseline risk analyses for VTE and bleeding requiring reoperation because of risk of bias but included it to baseline risk analyses for other outcomes.
Snyder 2020: Open liver resection: We did not include this study to the baseline risk analyses for VTE because of overlapping population but included it to baseline risk analyses for other outcomes.
Tien 2005: Open pancreaticoduodenectomy: We did not include this study to the baseline risk analyses for bleeding requiring reoperation because of risk of bias but included it to baseline risk analyses for other outcomes.
Tien 2008: Open pancreaticoduodenectomy: We did not include this study to the baseline risk analyses for bleeding requiring reoperation because of risk of bias but included it to baseline risk analyses for other outcomes.
Turrini 2005: Open pancreaticoduodenectomy: We did not include this study to the baseline risk analyses for bleeding requiring reoperation because of risk of bias but included it to baseline risk analyses for other outcomes.
Wei 2009: Open pancreaticoduodenectomy: We did not include this study to the baseline risk analyses for bleeding requiring reoperation because of risk of bias but included it to baseline risk analyses for other outcomes.
Yekebas 2007: Open pancreaticoduodenectomy: We did not include this study to the baseline risk analyses for bleeding requiring reoperation because of risk of bias but included it to baseline risk analyses for other outcomes.
Zettervall 2020: We excluded the study from open pancreaticoduodenectomy procedure estimate because of overlapping population with Vining 2020 study, but not from open pancreaticoduodenectomy with vascular resection and open pancreaticoduodenectomy without vascular resection procedures.

16. Peri- and intraoperative risk of bleeding in individual studies in upper-gastrointestinal and hepatopancreatobiliary surgery

Reference	Total patients	Perioperative bleeding	Reported Intra-operative Bleeding		
	n	Peri-operative bleeding requiring transfusion	Fatal intra-operative bleeding	Intra-operative bleeding requiring conversion to open	Intra-operative bleeding requiring transfusion
**Distal pancreatectomy, laparoscopic**					
Anonsen 2015	69			1	
Nakamura 2015	902				33
Sulpice 2015	347				
Kwon 2016	111			0	1
Dokmak 2017	165				6
Daniel 2018	1789	131			
Chen 2019	353			1	0
**Distal pancreatectomy, robotic**					
Zureikat 2013	83				
**Distal pancreatectomy, open**					
Yekebas 2007	116				
Lee 2008	180				14
Dedania 2013	70		0†	0†	
Nakamura 2015	1108				46
Sulpice 2015	2406				
Daniel 2018	1790	317			
Boone 2019	55				
Mussle 2020	191				
**Distal pancreatectomy, laparoscopic, benign**					
Daniel 2018	1030	70			
Chen 2019	116			0	0
**Distal pancreatectomy, laparoscopic, malignant**					

Sulpice 2015	347				
Daniel 2018	759	61			
**Distal pancreatectomy, open, benign**					
Daniel 2018	655	110			
**Distal pancreatectomy, open, malignant**					
Dedania 2013	70		0†	0†	
Sulpice 2015	2406				
Daniel 2018	1135	207			
Boone 2019	55				
**Liver resection, laparoscopic**					
Vibert 2006	84		0	3	5
Dagher 2009	210		0	9	30
Abu Hilal 2010	80		0	4	2
Dagher 2010	163			11	16
Kazaryan 2010	139			3	26
Bhojani 2012	57		0		7
Soubrane 2014	351			14	17
Cauchy 2015	223			14	
Fuks 2016	226				30
Cipriani 2018	698			17	
Sucandy 2018	831				
Ainoa 2020	84		0†	0†	1†
Triantafyllidis 2020	431				
Stiles 2017	859	52			
**Liver resection, robotic**					
Kingham 2016	64			1	1
Daskalaki 2017	67			0	9

Sucandy 2020	77				
**Liver resection, open**					
Stewart 2004	137		0†	0†	15†
Zhou 2007	81				
Lee 2009	248	19		1	
Lordan 2009	469				
Andres 2011	689		0		154
Nobili 2012	555				147
Tzeng 2012	5651	43			
Barbas 2013	1281				
Aramaki 2014	539	39			
Nathan 2014	2147				
Bagante 2016	2452	523			
de'Angelis 2016	329				
Fuks 2016	988				288
Yokoo 2016	14970				
Khandoga 2017	184				
Singh 2017	86				
Kron 2019	211	44	0†	0†	21†
Ainoa 2020	428		0†	0†	47†
Snyder 2020	388				138
Tahkola 2020	73				
**Liver resection, laparoscopic, minor**					
Soubrane 2014	351			14	17
Stiles 2017	859	52			
Ainoa 2020	78		0†	0†	1†
**Liver resection, laparoscopic, major**					

Dagher 2009	210		0	9	30
Cauchy 2015	223			14	
Fuks 2016	226				30
**Liver resection, open, minor**					
Tzeng 2012	3376	20			
Aramaki 2014	539	39			
Ainoa 2020	250		0†	0†	47†
**Liver resection, open, major**					
Zhou 2007	81				
Tzeng 2012	1690	20			
de'Angelis 2016	329				
Fuks 2016	988				288
Singh 2017	86				
Kron 2019	211	44	0†	0†	44†
Ainoa 2020	178		0†	0†	47†
Snyder 2020	388				138
Tahkola 2020	73				
**Pancreaticoduodenectomy, laparoscopic**					
Kendrick 2010	62				
Dokmak 2017	70				6
Yu 2018	191				38
Chen 2019	186			4	26
Song 2020	500		0	0	0
Vining 2020	407	71			
Wang 2020	550	36			

Boone 2019	200				
**Pancreaticoduodenectomy, robotic**					
Zureikat 2013	132				
Rosemurgy 2019	155			2	0
Vining 2020	498	52			
**Pancreaticoduodenectomy, open**					
Martignoni 2001	257				
Adam 2004	301				
Balachandran 2004	218				
Tien 2005	402				
Turrini 2005	172				
Koukoutsis 2006	362				
Blanc 2007	411				4
Yekebas 2007	1141				
Tien 2008	283				
Wei 2009	628				
Pandanaboyana 2010	67				
Kneuertz 2011	220				103
Mañas-Gómez 2011	107				
Ricci 2012	113				
Enomoto 2014	9830				
Feng 2014	840				283
Kokudo 2014	187				
Ravikumar 2014	1070				

Flis 2016	111				
Soriano 2016	67				
Fujikawa 2018	100		0		18
Kantor 2018	9235	1680			
Boone 2019	327				
Faraj 2019	300				62
Mataki 2019	315				
Rystedt 2019	1864		0	0	404
Snyder 2019	120	63			
Cao 2020	151				16
Komokata 2020	77				30
Mussle 2020	699				
Tahkola 2020	218				
Vining 2020	12612	2237			
**Pancreaticoduodenectomy, laparoscopic, without venous resection**					
Dokmak 2017	70				6
Kendrick 2010	62				
Yu 2018	191				38
Wang 2020	473	25			
**Pancreaticoduodenectomy, laparoscopic, with venous resection**					
Wang 2020	77	11			
**Pancreaticoduodenectomy, open, without venous resection**					
Turrini 2005	172				
Blanc 2007	411				4

Ravikumar 2014	840	183		
Flis 2016	111			
Kantor 2018	8258	1356		
Faraj 2019	300			62
Cao 2020	151			16
Zettervall 2020	2566			
Feng 2014	840			283

Pancreaticoduodenectomy, open, with venous resection

Ravikumar 2014	230	73		
Kantor 2018	640	223		
Kantor 2018	224	101		
Snyder 2019	120	63		
Zettervall 2020	436			

Gastrectomy, laparoscopic

Sexton 2008	61		1	
Saka 2010	178			
Mamidanna 2013	480			
Son 2014	58			
Glenn 2015	789			
Suda 2015	438			
Chen 2016	253			
Chen 2016	379			
Han 2016	1355			
Nakauchi 2016	437			
Ntutumu 2016	1205			

Wang 2017	1657		
Hiki 2018	1067		
Osaki 2018	129		
Shimada 2018	243		
Xu 2019	430		
Alzahrani 2020	207		
Sakamoto 2020	13187		
Shibasaki 2020	1042	0†	0
**Gastrectomy, robotic**			
Song 2009	100		
Son 2014	51		
Glenn 2015	223		
Suda 2015	88		
Nakauchi 2016	84		
Alhossaini 2019	288		1
Okabe 2019	115		
Shibasaki 2020	359	0†	0
**Gastrectomy, open**			
Park 2005	548		
Pedrazzani 2007	310		
Lamb 2008	180	0	
Oh 2009	410		
Sah 2009	809		
Saka 2010	3014		
Mamidanna 2013	10233		

Papenfuss 2014	2580	
Glenn 2015	8585	
Han 2016	3256	
Chen 2017	124	
Kung 2017	1101	
Hiki 2018	1067	
Xu 2019	768	
Sakamoto 2020	45502	
**Gastrectomy, laparoscopic, subtotal**		
Sexton 2008	61	1
Chen 2016	379	
Hiki 2018	1067	
Shimada 2018	243	
**Gastrectomy, laparoscopic, total**		
Son 2014	58	
Chen 2016	253	
Wang 2017	1657	
Sakamoto 2020	13187	
**Gastrectomy, robotic, total**		
Son 2014	51	
**Gastrectomy, open, subtotal**		
Park 2005	403	
Pedrazzani 2007	310	
Sah 2009	809	
Saka 2010	2111	
Papenfuss 2014	1581	

Hiki 2018	1067				
**Gastrectomy, open, total**					
Park 2005	145				
Oh 2009	410				
Saka 2010	903				
Papenfuss 2014	999				
Chen 2017	124				
Sakamoto 2020	45502				
**Gastric bypass, laparoscopic**					
Kothari 2007	476		0		
Rabl 2011	644				
Benizri 2013	100		0	0	
Woo 2013	55				
Inaba 2018	128349				
Thereaux 2018	33611				
Dugan 2020	117599				
Gambhir 2020	102146	1130			
Sada 2020	561		0	0	0
**Gastric bypass, robotic**					
Yu 2006	100				
Ayloo 2011	90		0	0	0
Benizri 2013	100		0	0	
Myers 2013	100			0	
Tieu 2013	1100			0	
Ayloo 2016	146				
Acevedo 2020	5817	36			

**Gastric bypass, open**					
Fernandez Jr 2004	1431				
Cotter 2005	107				
Abou-Nukta 2006	1225				
Gargiulo 2006	606		0		
Gargiulo 2007	193				
Nguyen 2007	6065				
Martins-Filho 2008	135				
Weller 2008	4883				
Caruana 2009	1652				
Consortium Longitudinal Assessment of Bariatric Surgery, Flum 2009	437				
Slotman 2010	61				
Finks 2011	1092				
Hutter 2011	988				
Rabl 2011	78				
Froehling 2012	228				
Masoomi 2012	42591				
Santo 2013	538		0		
Lidor 2014	5282				
Nielsen 2018	503	14			
**Sleeve gastrectomy, laparoscopic**					
Woo 2013	132				
Alsina 2014	100	4			
Biertho 2014	378				
Sakran 2016	3003		0	0	0
Villagran 2016	1236				

Moradian 2017	50				
Brunetti 2018	60				
Guerrier 2018	47982	480			
Inaba 2018	30257				
Nimeri 2018	527	5			
Thereaux 2018	62266				
Abuoglu 2019	302		0		
AlKhaldi 2019	187		0	0	0
Dugan 2020	312065				
Gambhir 2020	266886	1247			
Johari 2020	259		0		
**Sleeve gastrectomy, robotic**					
Romero 2013	134				
Ecker 2016	411	16			0
Moon 2018	740				
Acevedo 2020	12912	50			

Blank spaces represent no information (not provided by paper or by author correspondence).

7. Supplementary methods

We followed our previously registered (PROSPERO: CRD42021234119) and published study protocol[1], as well as Preferred Reporting Items for Systematic Reviews and Meta-analyses (PRISMA) and Meta-analysis Of Observational Studies in Epidemiology (MOOSE) guidance[2-4].

1. Eligibility

Through discussion and consensus building, expert panelists, including experienced general abdominal, colorectal, UGI and HPB surgeons and clinician-methodologists, selected the most relevant general abdominal, colorectal, UGI and HPB procedures for this study. We included observational studies that enrolled a minimum of 50 adult patients undergoing a target surgical procedure that reported the incidence of at least one of the patient-important outcomes of interest: fatal PE, symptomatic PE, symptomatic DVT, symptomatic VTE, fatal bleeding, bleeding requiring reintervention (including exploration and angioembolization), bleeding leading to transfusion, and bleeding leading to post-operative hemoglobin below 70 g/L.[1]

2. Data sources and searches

With the aid of an information specialist (Rachel J. Couban), we performed comprehensive searches, without language restrictions, on Embase, MEDLINE, Web of Science, and Google Scholar from January 1, 2004, to October 27, 2020. After completing the screening for the articles identified in the search, to identify additional eligible studies we reviewed reference lists of eligible studies as well as identified review articles. In addition, we performed separate searches for randomized trials addressing the effects of pharmacological and mechanical prophylaxis on risks of VTE and bleeding after surgery. Pages 138-152 provide details of the search strategies.

To inform modeling of VTE outcomes for studies with variable length of follow-up, we conducted a separate systematic review regarding the risk and time course of VTE by post-operative day[5].

To estimate thromboprophylaxis use in studies with missing thromboprophylaxis information, we used previously published studies as follows: i) if we had identified a study that reported thromboprophylaxis from the same country/region, time period and procedure, we used data from this study; ii) if information from similar time and place was not available, we used information from a large survey or population-based study of thromboprophylaxis practice. If there were no previously published studies available, our web-based survey on thromboprophylaxis use informed our decisions (pages 103-108).

3. Study selection and data collection

We developed standardized forms with detailed instructions for screening of abstracts and full texts, risk of bias, assessment of evidence certainty, and data extraction. Independently and in duplicate, two methodologically trained investigators applied the forms to screen study reports for eligibility and extracted data. In the full text screening, at least one of the investigators was a surgeon. Because of the large number of studies, we conducted our data extraction in two phases. First, we extracted data regarding procedure characteristics (procedure name, number of patients, outcomes reported) and assessed the risk of bias. In the second phase (after exclusions based on risk of bias assessments, see more in the paragraph “Choosing best estimates”), we collected information on patient characteristics and detailed data on outcomes reported. At each stage, an adjudicator (lead author or clinician-methodologist) resolved disagreements on judgments. We sent our consensus data extraction to the original authors for confirmation or correction and asked for clarification regarding missing or unclear information.

4. Analysis

1. Outcome measures

The primary outcomes were the procedure-specific cumulative incidence of symptomatic VTE and major bleeding within 4 weeks (28 days) post-surgery (in the absence of use of thromboprophylaxis). VTE included symptomatic PE, symptomatic DVT, or both in the same patient. We used three major bleeding definitions: (1) bleeding requiring reintervention (including exploration and angioembolization), (2) bleeding leading to the transfusion of one or more units of red blood cells, and (3) bleeding leading to post-operative hemoglobin below 70 g/L. We also separately recorded symptomatic splanchnic vein thrombosis (SVT), including thrombosis of the portal, splenic, mesenteric, or supra-hepatic veins. In addition, we measured the incidence of fatal pulmonary embolism and fatal bleeding.

Besides stratifying the VTE and bleeding risk estimates by procedure, we also classified them by approach (such as open, laparoscopic, or robotic), indication (such as benign vs malignant), and if procedure was elective or emergency, if necessary and possible.

2. Calculating the risk of VTE and bleeding for individual studies

We adjusted the reported incidence of VTE and bleeding for the use of pharmacological and mechanical thromboprophylaxis. For patients who received prophylaxis, we multiplied the reported incidence by the relative risk of thromboprophylaxis for the duration of prophylaxis use. Our updated meta-analyses of RCTs in general, gynecologic and urologic surgery informed the relative risk estimates of thromboprophylaxis (for forest plots, see pages 123-137)[6-8,9-11]. Our adjustments were as follows: i) for unfractionated heparin (UFH) and low-molecular weight heparin (LMWH) RR of 0.46 for VTE and 1.51 for bleeding; ii) for aspirin RR of 0.76 for VTE and 1.20 for bleeding; iii) for any mechanical prophylaxis RR 0.43 for VTE (no adjustment for bleeding); iv) for combination therapy of pharmacologic plus mechanical (versus pharmacological alone) RR of 0.59 for VTE (no adjustment for bleeding). A recent systematic review and network meta-analysis of RCTs in noncardiac surgery reported that direct oral anticoagulants (DOACs) had similar effects on both VTE and bleeding as LMWH[12]. We had high certainty in estimates of the effects of pharmacological prophylaxis but low certainty for mechanical prophylaxis (surrogate outcomes, very few patient-important events, unblinded patients and assessors; sections 9.6-9.18). Finally, we inferred that preoperative thromboprophylaxis did not provide meaningful extra benefit (for VTE prevention) or harm (bleeding)[13]. For studies that provided the number of DVT or PE events but not VTE, we modeled the number of VTE events using studies that had reported all DVT, PE, and VTE events (section 7.7 Overlap of DVT, PE, and VTE: How we dealt with studies that did not provide the number of VTE but provided DVT, PE, or both)

3. Modeling the risk of VTE and bleeding over time

We used cumulative incidence estimates at 4 weeks post-surgery (28 days) for our procedure-stratified estimates for the incidence of VTE and major bleeding. For the studies that did not report VTE estimates using this interval, we used the model developed in our separate systematic review to adjust the absolute VTE risk by post-operative day[5]. This systematic review provided estimates of the occurrence of VTE on each day until 4 weeks post-operatively. For the timing of VTE from 4 weeks (28 days) to 3 months (90 days) post-operatively, we modeled estimates using an approach we have previously published[8]. Using our new systematic review information and the older approach, we developed a model for the time course of VTE from the day of surgery to 3 months post-surgery (section 8.1 Proportion of cumulative risk of VTE by day since surgery during the first 90 post-operative days).

For the studies that did not report bleeding estimates using this interval, we created a new model using data from the placebo arm of a large pragmatic RCT[9] to adjust the absolute bleeding risk by post-operative day. However, as this study reported risk of both intraoperative and postoperative bleeds without distinguishing their proportions, we modeled the proportion of intraoperative bleeds with data from studies included in this review

(see section 8.2. Proportion of cumulative incidence of major bleeding by day since surgery during the first 90 post-operative day). This model of bleeding risk over time shows that 86% of the 4 week bleeding events happen during the first week.

4. Choosing the best estimates

We used the median value of incidence from studies to estimate the baseline risk of VTE and major bleeding[1]. When, for a target procedure, we identified five or more articles at low risk of bias with a total of 1,000 or more patients, we excluded studies with moderate or high risk of bias. When this was not the case but at least 10 articles with at least 2,000 patients from studies proved at very low, low, or moderate risk of bias, we excluded studies with high risk of bias. In other situations, we used all studies irrespective of their risk of bias. As an incidence of 0.00% for VTE or major bleeding is implausible in general surgery, when the median estimate was 0.00% and the mean was not 0.00%, we used the mean rather than the median. If no studies reported on the incidence for a particular procedure, we considered using an estimate from the most similar procedure (See evidence profiles for details). Finally, we estimated the case fatality rates by dividing the number of fatal PE events by the number of symptomatic VTE events using studies that provided both estimates (Section 7.9 Case fatality and estimates of fatal VTE and fatal bleeding). We used a similar approach to estimate the case fatality for major bleeding. We estimated the fatal VTE and fatal major bleeding risks for procedures by taking case fatality rates of the overall reported risk of symptomatic events for the procedure.

5. Stratifying the risk of VTE and bleeding according to patient risk factors

After assessing the procedure-specific baseline risk of VTE, we stratified the risk by patient-related risk factors using a method previously described[6-8]. We assessed four risk groups (1) age 75 or more, (2) body mass index (BMI) of 35 or more, (3) VTE in a first degree relative (parents, full siblings, or children)—all of these increase the risk approximately two-fold—and (4) prior VTE or patients with any combination of two or more risk factors, with risk ratio of approximately 4 (Supplementary table 18)[14-22]. Eligible studies and prior literature provided estimates of the proportion of patients with each of these risk factors, allowing estimates of the extent of overlap and thus calculation of estimates for each risk group (see section 7.8. Patient risk strata). Our search did not reveal studies demonstrating convincing and replicable risk factors for bleeding[1]. Therefore, we did not stratify bleeding risk by patient-specific factors.

6. Supplementary table 18. Risk of venous thromboembolism according to patient risk factors

Risk group	Risk factors	Risk
**Low risk**	No risk factors	1x
**Medium risk**	Any one of the following:<br>Age 75 years or more<br>Body mass index 35 or more<br>VTE in 1st degree relative (parent, full sibling, or child)	2x
**High risk**	Prior VTE<br>or<br>Patients with any combination of two or more risk factors	4x
**VTE = venous thromboembolism**		

7. Risk of bias and assessment of the evidence certainty

Methods to evaluate the risk of bias in longitudinal cohort studies are less developed than the methods in randomized trials[23]. Through discussion and consensus building, and considering previous literature[6-8,24-26], we developed an instrument to categorize risk of bias of the studies[1]. For the risk of bias assessments, we evaluated each study according to six domains: i) sampling of the study population, ii) reporting of thromboprophylaxis, iii) source of information, iv) whether a majority of patient recruitment years were earlier or later than 2010, v) clear specification of duration of follow-up, and vi) study type (Supplementary table 2, page 146). For each domain, we judged studies to have either a high or low risk of bias. We classified studies according to risk of bias domains as follows: no high risk of bias domains as very low, 1 high risk of bias domain as low, 2 high risk of bias domains as moderate, and 3 or more high risk of bias domains as high overall risk of bias[1].

We used the Grading of Recommendations Assessment, Development and Evaluation (GRADE) approach to rate the evidence certainty (also known as quality of evidence or confidence in evidence; Supplementary Table 19)[27,28]**.** The evidence certainty from observational studies addressing a question of prognosis begins as high certainty[6,29]; in all cases, we rated down to moderate owing to uncertainties in our modeling of risk of VTE and bleeding over time and patient risk strata[1]. We further lowered certainty in fatal VTE, and fatal bleeding estimates to low because of uncertainties in the modeling of cause of death**.** When identified, we further rated down for risk of bias, inconsistency of results, indirectness of evidence, or imprecision. In very low risk of VTE, even multiplying the risk by 5 times would lead to low (or very low) risk of VTE and would not change decisions on pharmacologic thromboprophylaxis. Therefore, if i) risk of VTE was 0.1% or less for all VTE risk strata and ii) quality of evidence was low or moderate, we considered rating up evidence certainty.

8. Supplementary table 19. Principles for the use of GRADE for assessment of evidence of risk of complications, and examples of GRADE use for estimating evidence of the risks of VTE and bleeding requiring reintervention after general abdominal surgery

**Domain**	**General principles in GRADE**	**Criteria for judgment in our study**
**Risk of Bias (RoB)**	The risk of misleading results is higher if studies are flawed in their design or conduct	We always rated down for RoB if most patients (>50%) came from studies at high RoB.<br><br>We did not rate down for RoB if most patients (>50%) came from studies at low or very low RoB.
**Inconsistency**	Widely differing estimates (heterogeneity or variability in results) across studies is called inconsistency. If point estimates vary substantially across studies, or confidence intervals show little or no overlap, certainty is likely to be rated down for inconsistency. Variability may arise from differences in populations or methodology.	We rated down for inconsistency if more than 10% of the studies had at least a 3% difference from the median value of the VTE, or at least a 1.5% difference from the median value of the bleeding requiring reintervention. However, if removing outliers did not materially change the median estimate, we considered not to rate down for inconsistency.

**Indirectness**	Evidence can be indirect in several ways. Indirectness may arise from differences in the population or outcome of interest between included studies and the population of interest.	We did not usually rate down for indirectness, as the eligible studies measured relevant outcomes in representative populations.
**Imprecision**	When studies have wide confidence intervals, typically because of relatively few patients or events, imprecision occurs.	We rated down by one level if studies included <1,000 patients and by two if they included <200 patients.
**Evidence certainty**	In studies of the risk of prognosis (including complications), a body of observational evidence begins as high certainty. The five GRADE domains consider in rating down certainty in estimates of treatment effect—that is RoB, imprecision, inconsistency, indirectness, and publication bias (no rating down for publication bias here) —as well as GRADE criteria for rating up certainty, also apply to estimates of the risks of complications. Evidence certainty options include high, moderate, low, and very low.	Although certainty in a body of evidence from observational studies addressing a question of prognosis begins as high certainty, we rated down to moderate owing to uncertainties in our models of the risk of VTE and bleeding over time and in our model of patient risk strata. We then further rated down as described for the other four categories.

References

1. Lavikainen LI, Guyatt GH, Lee Y, et al. Systematic reviews of observational studies of Risk of Thrombosis and Bleeding in General and Gynecologic Surgery (ROTBIGGS): introduction and methodology. *Syst Rev*. Oct 8 2021;10(1):264. doi:10.1186/s13643-021-01814-2
2. Moher D, Liberati A, Tetzlaff J, Altman DG, Group P. Preferred reporting items for systematic reviews and meta-analyses: the PRISMA statement. *Ann Intern Med*. Aug 18 2009;151(4):264-9, W64. doi:10.7326/0003-4819-151-4-200908180-00135
3. Page MJ, McKenzie JE, Bossuyt PM, et al. The PRISMA 2020 statement: an updated guideline for reporting systematic reviews. *BMJ*. Mar 29 2021;372:n71. doi:10.1136/bmj.n71
4. Stroup DF, Berlin JA, Morton SC, et al. Meta-analysis of observational studies in epidemiology: a proposal for reporting. Meta-analysis Of Observational Studies in Epidemiology (MOOSE) group. *JAMA*. Apr 19 2000;283(15):2008-12. doi:10.1001/jama.283.15.2008
5. Singh T, Lavikainen L, Halme A, et al. Timing of symptomatic venous thromboembolism after surgery: A systematic review and meta-analysis (accepted for publication; in press). *BJS*. 2023;doi:10.1093/bjs/znad035
6. Tikkinen KAO, Craigie S, Agarwal A, et al. Procedure-specific Risks of Thrombosis and Bleeding in Urological Cancer Surgery: Systematic Review and Meta-analysis. *Eur Urol*. Feb 2018;73(2):242-251. doi:10.1016/j.eururo.2017.03.008
7. Tikkinen KAO, Craigie S, Agarwal A, et al. Procedure-specific Risks of Thrombosis and Bleeding in Urological Non-cancer Surgery: Systematic Review and Meta-analysis. *Eur Urol*. Feb 2018;73(2):236-241. doi:10.1016/j.eururo.2017.02.025
8. Tikkinen KA, Agarwal A, Craigie S, et al. Systematic reviews of observational studies of risk of thrombosis and bleeding in urological surgery (ROTBUS): introduction and methodology. *Syst Rev*. Dec 23 2014;3(1):150. doi:10.1186/2046-4053-3-150
9. Devereaux PJ, Mrkobrada M, Sessler DI, et al. Aspirin in patients undergoing noncardiac surgery. *N Engl J Med*. Apr 17 2014;370(16):1494-503. doi:10.1056/NEJMoa1401105
10. Mantz J, Samama CM, Tubach F, et al. Impact of preoperative maintenance or interruption of aspirin on thrombotic and bleeding events after elective non-cardiac surgery: the multicentre, randomized, blinded, placebo-controlled, STRATAGEM trial. *Br J Anaesth*. Dec 2011;107(6):899-910. doi:10.1093/bja/aer274
11. Prevention of pulmonary embolism and deep vein thrombosis with low dose aspirin: Pulmonary Embolism Prevention (PEP) trial. *Lancet*. Apr 15 2000;355(9212):1295-302.
12. Marcucci M, Etxeandia-Ikobaltzeta I, Yang S, et al. Benefits and harms of direct oral anticoagulation and low molecular weight heparin for thromboprophylaxis in patients undergoing non-cardiac surgery: systematic review and network meta-analysis of randomised trials. *BMJ*. Mar 9 2022;376:e066785. doi:10.1136/bmj-2021-066785
13. McAlpine K, Breau RH, Werlang P, et al. Timing of Perioperative Pharmacologic Thromboprophylaxis Initiation and its Effect on Venous Thromboembolism and Bleeding Outcomes: A Systematic Review and Meta-Analysis. *J Am Coll Surg*. Nov 2021;233(5):619-631 e14. doi:10.1016/j.jamcollsurg.2021.07.687
14. Caprini JA. Thrombosis risk assessment as a guide to quality patient care. *Dis Mon*. Feb-Mar 2005;51(2-3):70-8. doi:10.1016/j.disamonth.2005.02.003
15. Edmonds MJ, Crichton TJ, Runciman WB, Pradhan M. Evidence-based risk factors for postoperative deep vein thrombosis. *ANZ J Surg*. Dec 2004;74(12):1082-97. doi:10.1111/j.1445-1433.2004.03258.x
16. Hansson PO, Welin L, Tibblin G, Eriksson H. Deep vein thrombosis and pulmonary embolism in the general population. 'The Study of Men Born in 1913'. *Arch Intern Med*. Aug 11-25 1997;157(15):1665-70.
17. Pannucci CJ, Laird S, Dimick JB, Campbell DA, Henke PK. A validated risk model to predict 90-day VTE events in postsurgical patients. *Chest*. Mar 1 2014;145(3):567-573. doi:10.1378/chest.13-1553
18. Parkin L, Sweetland S, Balkwill A, Green J, Reeves G, Beral V. Body mass index, surgery, and risk of venous thromboembolism in middle-aged women: a cohort study. *Circulation*. Apr 17 2012;125(15):1897-904. doi:10.1161/circulationaha.111.063354
19. Rogers SO, Jr., Kilaru RK, Hosokawa P, Henderson WG, Zinner MJ, Khuri SF. Multivariable predictors of postoperative venous thromboembolic events after general and vascular surgery: results from the patient safety in surgery study. *J Am Coll Surg*. Jun 2007;204(6):1211-21. doi:10.1016/j.jamcollsurg.2007.02.072
20. Stein PD, Hull RD, Kayali F, Ghali WA, Alshab AK, Olson RE. Venous thromboembolism according to age: the impact of an aging population. *Arch Intern Med*. Nov 8 2004;164(20):2260-5. doi:10.1001/archinte.164.20.2260
21. Tosetto A, Frezzato M, Rodeghiero F. Prevalence and risk factors of non-fatal venous thromboembolism in the active population of the VITA Project. *J Thromb Haemost*. Aug 2003;1(8):1724-9. doi:10.1046/j.1538-7836.2003.00313.x

22. Weill-Engerer S, Meaume S, Lahlou A, et al. Risk factors for deep vein thrombosis in inpatients aged 65 and older: a case-control multicenter study. *J Am Geriatr Soc*. Aug 2004;52(8):1299-304. doi:10.1111/j.1532-5415.2004.52359.x
23. Guyatt GH, Oxman AD, Vist G, et al. GRADE guidelines: 4. Rating the quality of evidence--study limitations (risk of bias). *J Clin Epidemiol*. Apr 2011;64(4):407-15. doi:10.1016/j.jclinepi.2010.07.017
24. Hayden JA, van der Windt DA, Cartwright JL, Cote P, Bombardier C. Assessing bias in studies of prognostic factors. *Ann Intern Med*. Feb 19 2013;158(4):280-6. doi:10.7326/0003-4819-158-4-201302190-00009
25. Sterne JA, Hernan MA, Reeves BC, et al. ROBINS-I: a tool for assessing risk of bias in non-randomised studies of interventions. *BMJ*. Oct 12 2016;355:i4919. doi:10.1136/bmj.i4919
26. Kim SY, Park JE, Lee YJ, et al. Testing a tool for assessing the risk of bias for nonrandomized studies showed moderate reliability and promising validity. *J Clin Epidemiol*. Apr 2013;66(4):408-14. doi:10.1016/j.jclinepi.2012.09.016
27. Guyatt GH, Oxman AD, Vist GE, et al. GRADE: an emerging consensus on rating quality of evidence and strength of recommendations. *BMJ*. Apr 26 2008;336(7650):924-6. doi:10.1136/bmj.39489.470347.AD
28. Guyatt GH, Oxman AD, Kunz R, et al. What is "quality of evidence" and why is it important to clinicians? *BMJ*. May 3 2008;336(7651):995-8. doi:10.1136/bmj.39490.551019.BE
29. Iorio A, Spencer FA, Falavigna M, et al. Use of GRADE for assessment of evidence about prognosis: rating confidence in estimates of event rates in broad categories of patients. *Bmj*. Mar 16 2015;350:h870. doi:10.1136/bmj.h870

5. Calculating baseline risks

We adjusted the reported incidence of VTE and bleeding for the use of pharmacological and mechanical thromboprophylaxis. We used point estimates of risk ratios (RR). For patients who received prophylaxis, we multiplied the reported risk by the relative risk of thromboprophylaxis for the duration of prophylaxis use. Our adjustments were as follows: i) for unfractionated heparin (UFH) and low-molecular weight heparin (LMWH) RR of 0.46 for VTE and 1.51 for bleeding; ii) for aspirin RR of 0.76 for VTE and 1.20 for bleeding; iii) for any mechanical prophylaxis RR 0.42 for VTE; iv) for combination therapy of pharmacologic plus mechanical (versus pharmacological alone) RR of 0.59 for VTE.

To adjust estimates of baseline risk for use of prophylaxis, we updated earlier meta-analysis of RCTs in urology, general surgery, gynecology, and gastrointestinal surgery[6-8]. We used information from RCTs about the relative risk of VTE and bleeding among those who received prophylaxis. Specifically, we used estimates from this meta-analysis that concluded that low molecular weight heparin and unfractionated heparin reduce the relative risk of VTE with risk ratio (RR) of 0.46 and increases the relative risk of major bleeding by RR 1.51 compared to no prophylaxis. We also conducted meta-analysis of effect of aspirin on symptomatic VTE versus placebo, including three RCTs [9-11]. For forest plots, see section 5. Forest plots for effects of pharmacological and mechanical thromboprophylaxis on VTE and bleeding. We used data from a network meta-analysis of 68 RCTs in noncardiac surgery for our estimate that direct oral anticoagulants had similar effects on both VTE and bleeding as low molecular weight heparin[12]. Based on meta-analysis of 46 studies in noncardiac surgery, antiplatelets increase risk of blood transfusion by 14% [30]. This meta-analysis, however, missed a large placebo-controlled RCT of 10 010 patients, which found that aspirin increases the risk of major bleeding by 23%[9]. We therefore estimated that aspirin increases the risk of major bleeding by approximately 20%. We didn't have estimates for combination prophylaxis of antiplatelets and mechanical prophylaxis, and therefore we didn't assume extra effect. Based on systematic review and meta-analysis, we estimated that inferior vena cava filters did not reduce risk of VTE [31]. We did not adjust splanchnic vein thrombosis estimates for thromboprophylaxis use, as we did not have available evidence on the effect.

For studies that did not report their VTE and bleeding estimates at 4 weeks, we modeled timing of bleeding using our timing models.

Example for VTE:

A study of 100 patients reported 2 VTE events in 30 days, and reported that LMWH was used for 21 days in 50% patients and mechanical prophylaxis was used for 7 days in the same 50% patients. Our goal is to estimate what the risk would have been if there was no LMWH or mechanical prophylaxis over a period of 28 days.

Reported risk of VTE at day 30 = 2/100 = 2.0%

At 7 days 30.5% of the baseline risk at 90 days has accumulated, and at 30 days 66.7% has accumulated.

Therefore, in the first 7 days, 45.7% (30.5/66.7%) of the risk at 30 days has accumulated and the remainder 54.3% (100.0%-45.7%) accumulates during next 23 days.

Of this 45.7%, 50% occurred in patients that used both pharmacological and mechanical prophylaxis and 50% occurred in patients that did not receive any thromboprophylaxis.

At 21 days, 58.2% of the risk at 90 days has accumulated. Therefore, in the days 8-21 41.5%((58.2%-30.5%)/66.7%) of the risk at 30 days has accumulated.

Of this 41.5%, 50% occurred in patients that used both pharmacological and mechanical prophylaxis and 50% occurred in patients that did not receive any thromboprophylaxis.

The remainder of 30 day risk, 12.8%(100.0%-45.7%-41.5%), accumulates during last 9 days. This 12.8% occurred in patients that did not receive any prophylaxis.

We estimated RR 0.46 for unfractionated heparin (UFH) and low-molecular weight heparin (LMWH) alone and RR 0.59 for combination therapy of any pharmacological plus any mechanical prophylaxis versus pharmacological alone.

One can then construct an algebraic equation to predict x, where x is the risk that would have occurred had patients not been using LMWH or mechanical prophylaxis. That equation takes the proportion of risk that would have occurred in the first 7 days without LMWH and mechanical prophylaxis, the proportion of risk that would have occurred during post-operative days 8-21, the relative risk of an event with LMWH and mechanical prophylaxis, the proportion of risk that occurred in the last 9 days, and the total risk observed and is as follows:

0.457*0.59*0.46*0.5*x+0.457*0.5*x+0.415*0.46*0.5x+0.415*0.5*x+0.128*x=2.0

x=2.0/(0.457*0.59*0.46*0.5+0.457*0.5+0.415*0.46*0.5+0.415*0.5+0.146)

x=2.772

When we solve for x, the risk that would have occurred without LMWH, we find a risk of 2.8% (at day 30).

At 28 days, 64.8% of 90 day risk has accumulated and at 30 days 66.7%.

Therefore 28 day risk in our example is:

64.8/66.7*2.8=2.7%

Example for bleeding:

A study of 100 has reported 2 bleedings requiring reintervention in 30 days and reported that LMWH was used for 21 days in all patients.

Reported risk of bleeding requiring reintervention at day 30 = 2/100 = 2.000%

Reported thromboprophylaxis: LMWH for 21 days for 100% of the population

Our risk model describes the cumulative risk of bleeding requiring reintervention up to 30 days. At 21 days, 91.2% of the risk at day 30 has accumulated, and the remainder 8.8% (100.0%-91.2%) accumulates during next 9 days.

In this example, all patients used LMWH for 21 days.

One can then construct an algebraic equation to predict x, where x is the risk that would have occurred had patients not been using LMWH. That equation takes the proportion of risk that would have occurred in the first 21 days without LMWH, the relative risk of an event with LMWH, the proportion of risk that occurred in the last 9 days, and the total risk observed and is as follows:

0.912*1.51*x+0.088*x=2.0

x=2.0/(0.912*1.51+0.088)

x= 1.365

When we solve for x, the risk that would have occurred without LMWH, we find a risk of 1.4% (at day 30).

We still need to get from the risk at 30 days to the risk at 28 days. Our model tells us that the risk at 28 days is 98.0% of the risk at 30 days and therefore the risk at 28 days is 1.4*0,98=1.3%.

References

1. Tikkinen KAO, Craigie S, Agarwal A, et al. Procedure-specific Risks of Thrombosis and Bleeding in Urological Cancer Surgery: Systematic Review and Meta-analysis. 2018; 73:242-251.
2. Tikkinen KAO, Craigie S, Agarwal A, et al. Procedure-specific Risks of Thrombosis and Bleeding in Urological Non-cancer Surgery: Systematic Review and Meta-analysis. 2018; 73:236-241.
3. Tikkinen KA, Agarwal A, Craigie S, et al. Systematic reviews of observational studies of risk of thrombosis and bleeding in urological surgery (ROTBUS): introduction and methodology. *Syst Rev* 2014; 3(1):150.
4. Marcucci M, Etxeandia-Ikobaltzeta I, Yang S, et al. Benefits and harms of direct oral anticoagulation and low molecular weight heparin for thromboprophylaxis in patients undergoing non-cardiac surgery: systematic review and network meta-analysis of randomised trials. *BMJ* 2022:e066785.
5. Devereaux PJ, Mrkobrada M, Sessler DI, et al. Aspirin in patients undergoing noncardiac surgery. *N Engl J Med* 2014; 370(16):1494-503.
6. Mantz J, Samama CM, Tubach F, et al. Impact of preoperative maintenance or interruption of aspirin on thrombotic and bleeding events after elective non-cardiac surgery: the multicentre, randomized, blinded, placebo-controlled, STRATAGEM trial †. *British Journal of Anaesthesia* 2011; 107(6):899-910.
7. Prevention of pulmonary embolism and deep vein thrombosis with low dose aspirin: Pulmonary Embolism Prevention (PEP) trial. *Lancet* 2000; 355(9212):1295-302.
8. Columbo JA, Lambour AJ, Sundling RA, et al. A Meta-analysis of the Impact of Aspirin, Clopidogrel, and Dual Antiplatelet Therapy on Bleeding Complications in Noncardiac Surgery. *Annals of Surgery* 2018; 267(1):1-10.
9. Bikdeli B, Chatterjee S, Desai NR, et al. Inferior Vena Cava Filters to Prevent Pulmonary Embolism: Systematic Review and Meta-Analysis. *J Am Coll Cardiol* 2017; 70(13):1587-1597.

6. Missing thromboprophylaxis information

1. Principles

We primarily used data from published literature and secondarily survey to estimate the use of thromboprophylaxis:

1. When we had included study that reported thromboprophylaxis from the same country/region, time period, and procedure, we used data from this study to estimate missing thromboprophylaxis.

2. If not available, we used information from a large survey or population-based study of thromboprophylaxis practice

3. If not available, we used information from our survey (we sometimes adjusted these estimates based on information on similar procedures)

Eligible studies included in review providing thromboprophylaxis estimates might not be representative of general practice as very few studies provided these estimates, and therefore we preferred using information from previously published literature on thromboprophylaxis practice.

Our survey of thromboprophylaxis practice:

We queried 32 general surgeons from 11 different countries and got 19 responses from 7 countries.

Answer options: No prophylaxis, until ambulating, hospital stay, 1 wk after discharge, 2 wks after discharge, 3 wks after discharge and 4 wks after discharge.

We collected length of stay (LOS) in our data extraction. We took median of reported LOS for the procedure in the same continent and time period (before or after 2010) and combined this information with the survey results. When we didn't have estimate of LOS from the same time period or continent, we used primarily information from the same continent but different time period, and secondarily from other continent (Information from North America for Europe, Europe for North America and Europe for Asia). We converted survey answers to days (No prophylaxis=0 days, Until ambulating=1 day, hospital stay=Median LOS for the procedure, 1 wk after discharge=Median LOS+7days, 2 wk after discharge= Median LOS + 14 days, 3 wk after discharge=Median LOS + 21 days, 4wk after discharge= Median LOS + 28 days).

We then took mean of survey answers converted to days to arrive in estimate of duration of thromboprophylaxis, separately for pharmacological and mechanical prophylaxis.

Information from previously published literature:

Colorectal procedures, North America:

We used information from Mukkamala 2020 study that analyzed 5,722 colorectal patients from Michigan MSQC registry on years 2017-2018 [1] . Of 5,722 patients, only 373 (6.5%) received extended-duration prophylaxis after discharge.

Based on our survey results we would assume 15-22 days of prophylaxis for colorectal resections in North America 2011-2021. However, based on Mukkamala study use of extended prophylaxis has not been common. Mukkamala may not include all prescriptions as paper and phone prescriptions are excluded, but also not all patients take their prescriptions.

We therefore assumed extended as meaning 14 or 21 days after discharge. We then estimated thromboprophylaxis duration as LOS + 7% receiving extended prophylaxis for 21 days. This way we arrive at days of thromboprophylaxis for colorectal procedures in North America.

Colorectal procedures, Europe:

Based on Srinivasaiah 2012 survey of 259 general surgeons from UK, we estimated the use of thromboprophylaxis for colorectal surgery procedures in Europe 2000-2010 [2]. We estimated that 78% discontinued pharmacological prophylaxis at discharge, 12% before discharge (we estimated this as LOS+1 day, divided by 2), 5% on mobilization (estimated at 1 day), 3,5% at 1-6 weeks (we estimated this as 3 weeks) and 1,5% 6 weeks after.

Hepatopancreatobiliary procedures (HPB), North America:

Based on survey results we would assume 25-33 days off prophylaxis for HPB resections in North America 2010-2021. However, based on Weiss 2014 survey, Ruff 2019 survey and Bateni 2020 study this would overestimate the use of thromboprophylaxis[3-5] .

Ruff 2019 Survey of 44 surgeons (USA and Canada): 36% discharge on thromboprophylaxis after major hepatectomy for malignancy (30% <28 days, 70% for 28days), 26% after minor hepatectomy for malignancy (40% <28 days, 60% for 28days). After discharge tpx is utilized in pancreaticoduodenectomy and distal pancreatectomy by 45% and 39% of respondants, (80% for 28 days.)

Bateni 2020 Study (USA, pancreatic cancer resections): Of the 1,003 pancreatic cancer patients who underwent pancreatic cancer resection, only 4.3% (44) were prescribed VTE ppx at discharge based on SEER and Medicare databases.

Weiss 2014 Survey, all HPB surgeries (200 surgeons, 80% from USA): 14% discharge on thromboprophylaxis (OR 0.37 for US, 28% discharge on thromboprophylaxis outside US and 10.4% in US)

Based on Weiss and Ruff we estimated for lap liver resection: We assumed extended as meaning 22 days after discharge (0,4*14+0,6*28=22,4 days). We estimated LOS (4 days) + 26% receiving extended (0,26*22= 5,72 days) and assumed 10 days of thromboprophylaxis.

Based on Weiss and Ruff estimated for open liver resection: We assumed extended as meaning 24 days after discharge (0,3*14+0,7*28=23.6 days). We then estimated LOS (6 days) + 36% receiving extended (0,36*24=8.64 days), and assume 15 days of thromboprophylaxis.

Based on survey we would assume 4 weeks after discharge of prophylaxis for pancreatic resections. Ruff reported approximately 40% as receiving extended thromboprophylaxis, Weiss 10% and Bateni 4% in North America. Based on this information we estimated that 20% of HPB patients received extended thromboprophylaxis in North America. We assumed extended meaning 21 days after discharge.

Hepatopancreatobiliary procedures (HPB), Europe:

For Europe we assumed that 28% received extended thromboprophylaxis based on Weiss. We assumed extended meaning 21 days after discharge.

Other considerations:

If authors reported mechanical thromboprophylaxis, but did not report anything on pharmacological thromboprophylaxis, we assumed that they did not use pharmacological thromboprophylaxis. Similarly, if authors reported pharmacological thromboprophylaxis, but did not report anything on mechanical thromboprophylaxis, we assumed that they did not use mechanical thromboprophylaxis.

If authors did not report duration of prophylaxis in days, but in some other way, we assumed duration that seemed most probable. For instance, Boone 2019 authors reported that "extended prophylaxis was not routinely used". We assumed length of stay as duration for pharmacological prophylaxis. If article reported duration as "until ambulation", we assumed 1 day. If article reported "during hospitalization" or "until discharge" we assumed reported length of stay, or, if unavailable, median length of stay for the procedure.

If we didn't have estimates for robotic approach use of thromboprophylaxis for some procedure, we used estimates from laparoscopic approach for the same procedure.

We shortened lap and open gastrectomy estimates from our survey by 50%, based on information from other procedures that our survey likely overestimates the use of extended thromboprophylaxis.

For lap and open proctocolectomy 2011-2021 in Europe we shortened our survey results by 50% based on information from the 2000-2010 literature.

For Lap liver resection 2000-2010 in Europe we used estimates from Lap liver resection 2011-2021 in Europe.

For Lap distal pancreatectomy 2011-2021 in Europe we used estimates from Lap distal pancreatectomy 2000-2010 in Europe.

For Australia we used data from Liu 2020 survey [6]

For studies from India we used data from Venkataram 2013 survey and ENDORSE study [7, 8]

For small bowel resection we didn't have any LOS estimates, so we used estimates from Turrentine 2021 [9]

For Martins-Filho 2008 open gastric bypass thromboprophylaxis we used data from study Santo 2013 as it was from same the country (Brazil). Otherwise, we used data from Rocha 2020 for estimates of thromboprophylaxis use in Brazil 2010-2021 [10]

For Holzheimer 2007 and Srsen 2008 open groin hernia we used Lozano 2015 duration as it was from same region and same procedure. (Our survey estimated 0 days, but as these studies reported use of thromboprophylaxis (but not duration), we determined it was not the case.)

For Li 2017 we used estimates from Zhang 2012 as it was from the same country, same time period and same procedure.

For Alves 2005 lap anterior resection we used data from Alves 2005 open anterior resection as it was from the same country and same year.

References

1. Mukkamala, A., et al., *Population-Based Analysis of Adherence to Postdischarge Extended Venous Thromboembolism Prophylaxis After Colorectal Resection.* Diseases of the Colon & Rectum, 2020. **63**(7).
2. Srinivasaiah, N., R. Arsalani-Zadeh, and J.R. Monson, *Thrombo-prophylaxis in colorectal surgery: a National Questionnaire Survey of the members of the Association of Coloproctology of Great Britain and Ireland.* Colorectal Disease, 2012. **14**(7): p. e390-e393.
3. Weiss, M.J., et al., *Venous thromboembolic prophylaxis after a hepatic resection: patterns of care among liver surgeons.* HPB (Oxford), 2014. **16**(10): p. 892-8.
4. Ruff, S.M., et al., *Practice patterns of VTE chemoprophylaxis after discharge following hepatic and pancreatic resections for cancer: A survey of hepatopancreatobiliary surgeons.* Journal of Thrombosis and Thrombolysis, 2019. **48**(1): p. 119-124.
5. Bateni, S., et al., *Venous thromboembolism prophylaxis after pancreatic cancer surgery: Are we following consensus guidelines?* Annals of surgical oncology, 2020. **27**(SUPPL 1): p. S188.
6. Liu, D.S., et al., *Variations in practice of thromboprophylaxis across general surgical subspecialties: a multicentre (PROTECTinG) study of elective major surgeries.* ANZ Journal of Surgery, 2020. **90**(12): p. 2441-2448.
7. Venkataram, A., et al., *Postoperative Venous Thromboembolism Prophylaxis by General Surgeons in a Developing Country: A Survey.* Thrombosis, 2013. **2013**: p. 1-5.
8. Cohen, A.T., et al., *Venous thromboembolism risk and prophylaxis in the acute hospital care setting (ENDORSE study): a multinational cross-sectional study.* The Lancet, 2008. **371**(9610): p. 387-394.
9. Turrentine, F.E., et al., *Determining the Association Between Unplanned Reoperation and Readmission in Selected General Surgery Operations.* Journal of Surgical Research, 2021. **267**: p. 309-319.
10. Rocha, A.T.C., et al., *Protocolos de profilaxia de tromboembolismo venoso (TEV) em hospitais brasileiros - PROTEV Brasil.* Jornal Vascular Brasileiro, 2020. **19**.

2. Supplementary table 20: Missing mechanical thromboprophylaxis

MECHANICAL thromboprophylaxis:	Europe	Europe	Europe	North America	North America	North America	Asia	Asia	Asia
	SURVEY[a]	ROTBIGGS[b]	ESTIMATE[c]	SURVEY[a]	ROTBIGGS[b]	ESTIMATE[c]	SURVEY[a]	ROTBIGGS[b]	ESTIMATE[c]
Lap appendectomy - 2011-2021	0		0	0		0	1		1
Lap appendectomy - 2000-2010	0		0	0		0			1
Open appendectomy - 2011-2021	0		0	0		0	1		1
Open appendectomy - 2000-2010	1		1	0		0			1
Lap cholecystectomy - 2011-2021	0		0	0		0	1		1
Lap cholecystectomy - 2000-2010	0		0	0		0			
Open cholecystectomy - 2011-2021	2		2	0		0			
Open cholecystectomy - 2000-2010	2		2	0		0			
Lap hernia repair (groin) - 2011-2021	0		0	0		0	1		1
Lap hernia repair (groin) - 2000-2010	0		0	0		0			
Open hernia repair (groin) - 2011-2021	0		0	0		0			
Open hernia repair (groin) - 2000-2010	0		0	0		0			
Lap hernia repair (ventral) - 2011-2021	1		1	0		0			1[d]
Lap hernia repair (ventral) - 2000-2010	1		1	0		0			1[d]
Open hernia repair (ventral) - 2011-2021	1		1	0		0			
Open hernia repair (ventral) - 2000-2010	1		1	0		0			
Lap small bowel resection - 2011-2021				1		1			
Lap small bowel resection - 2000-2010				1		1			
Open small bowel resection - 2011-2021				1		1			
Open small bowel resection - 2000-2010				1		1			
Lap splenectomy (elective) - 2011-2021				1		1			
Lap splenectomy (elective) - 2000-2010	2		2	1	2	2	0		0
Open splenectomy (elective) - 2011-2021				2		2	1		1
Open splenectomy (elective) - 2000-2010	2		2	2		2	0		0

Estimates presented as days. a Mean of survey answers; b Median of durations reported for the procedure in articles included in the review; c Assumed duration for the procedure when article did not report duration.; d Information from a large survey or population-based study of thromboprophylaxis practice.

3. Supplementary table 21: Missing pharmacological thromboprophylaxis

PHARMACOLOGICAL thromboprophylaxis:	Europe	Europe	Europe	Europe	North America	North America	North America	North America	Asia	Asia	Asia	Asia
	SURVEY[a]	ROTBIGGS[b]	LITERATURE[c]	ESTIMATE[d]	SURVEY[a]	ROTBIGGS[b]	LITERATURE[c]	ESTIMATE[d]	SURVEY[a]	ROTBIGGS[b]	LITERATURE[c]	ESTIMATE[d]
Lap appendectomy - 2011-2021	0			0	1			1	0			0
Lap appendectomy - 2000-2010	0			0	1			1				0
Open appendectomy - 2011-2021	0			0	2			2	0			0
Open appendectomy - 2000-2010	0			0	2			2				0
Lap cholecystectomy - 2011-2021	0	3		3	2			2	0	2		2
Lap cholecystectomy - 2000-2010	1	4		4	3			3				
Open cholecystectomy - 2011-2021	5			5	6			6				
Open cholecystectomy - 2000-2010	5	4		4	6			6				
Lap hernia repair (groin) - 2011-2021	0			0	2			2		30		30
Lap hernia repair (groin) - 2000-2010	0			0	2			2				
Open hernia repair (groin) - 2011-2021	0			0	2			2		31		31
Open hernia repair (groin) - 2000-2010	0	7		7	2			2				
Lap hernia repair (ventral) - 2011-2021	3			3	2			2			1	1
Lap hernia repair (ventral) - 2000-2010	3	1		1	2			2			1	1
Open hernia repair (ventral) - 2011-2021	5	3		3	5	5		5				
Open hernia repair (ventral) - 2000-2010	4	10		10	5			5				
Lap small bowel resection - 2011-2021					6			6				
Lap small bowel resection - 2000-2010					5			5				
Open small bowel resection - 2011-2021					6			6				
Open small bowel resection - 2000-2010					5			5				
Lap splenectomy (elective) - 2011-2021					2			2				
Lap splenectomy (elective) - 2000-2010	11	7		7	2	2		2				0
Open splenectomy (elective) - 2011-2021					9			9				
Open splenectomy (elective) - 2000-2010	11	7		7	8			8		0		0

Estimates presented as days. a Mean of survey answers, b Median of durations reported for the procedure in articles included in the review, c Information from a large survey or population-based study of thromboprophylaxis practice, d Assumed duration for the procedure when article did not report duration.

7. Overlap of DVT, PE, and VTE: How we dealt with studies that did not provide the number of VTE but provided DVT, PE, or both

Ideally, studies would tell us the number of patients who suffered DVT alone, the number who suffered PE alone, and either the number who suffered both DVT and PE (in which case, the number of VTE is found by adding up the three numbers) or the total number of VTE (from which one can infer the number who suffered both DVT and PE). Unfortunately, a minority of studies report in this way, and this creates a challenge.

For instance, if a study tells us that three patients suffered a DVT and three patients suffered a PE the total VTE could be anywhere from 3 (3 patients suffered both DVT and PE) to 6 (3 suffered DVT, 3 suffered PE, and 0 suffered both).

We dealt with the problem as follows.

For studies that did not provide the numbers of VTE but provided DVT, PE, or both, we estimated the numbers of VTE using the following approach. We reviewed data from studies that reported the number of DVT, the number of PE, and VTE totals from both general and gynecologic surgery.

We estimated the overlap from these studies that reported the following:

5719 PEs, 17593 DVTs, and 22584 (not 23312) VTEs. We then applied the degree of overlap to estimate the actual numbers of VTEs in studies that provided only separate reports of DVT and/or PE.

If paper provided PE, but did not report DVT or VTE: we calculated that nVTE = nPE * 22584/5719

If paper provided DVT, but did not report PE or VTE: we calculated that nVTE= nDVT * 22584/17593

If paper provided PE and DVT, but did not report VTE: we calculated that nVTE = (nPE +nDVT) * 22584/23312

However, if either nPE or nDVT was zero, nVTE was sum of nDVT+nPE.

Examples:

If 30 PE reported but DVT and VTE not reported, nVTE = 30* 22584/5719= 118.468

If 30 DVT reported but PE and VTE not reported, nVTE = 30* 22584/17593=38.512

If 30 PE and 30 DVT were reported, nVTE = (30+30)* 22584/23312=58.127

8. Patient risk strata

To estimate the proportion of patients aged more than 75 years (per procedure):

- Age distribution of each procedure was estimated by taking the age distributions (mean/median and standard deviation (SD)), where available) of all studies identified for procedure. Medians and SDs of the ages were used to create an "overall" age distribution.

- When no SDs were available we used range or inter-quartile range (IQR) rules to estimate a SD, using rules: SD is ¼ of range; and IQR is 1.35-times SD

- After we had completed the estimation of mean age and SD, we then assumed a normal distribution and calculated the proportion above 75 years using excel formula: 1-NORM.DIST(75;µ;σ;TRUE), where μ=mean and σ=SD.

To estimate the proportion of patients with BMI 35 or more:

- BMI information was not collected in our data extraction (in most cases it was unavailable)

- We used data from the earlier ROTBUS systematic reviews for our estimates of BMI by age group [6-8]. We decided that this was suitable as BMI has not changed significantly [32].

To estimate the proportion of patients with personal history of VTE:

- We used data from the earlier ROTBUS systematic reviews[6-8], that used the data from Swedish population-based study which estimated cumulative risk of a first VTE event.

To estimate the proportion of patients with family history of VTE, we used data from the earlier ROTBUS systematic reviews[6-8], and estimated that FH risk is always 3%.

Calculating risk stratification:

- After calculating the proportions for these risk factors, then we needed to calculate how much they overlap.

- To account for overlap, we estimated that the prevalence of having one or more risk factors is 80% of the sum of prevalences of the individual risk factors.

- So for example for laparoscopic cholecystectomy, we calculated the following percentages for individual risk factors: age: 5%, BMI: 14%, FH: 3%, Personal history: 0.5%. In this case total sum of prevalences is 5%+14%+3%+0.5%=22.5%. Therefore when also considering some overlap, the prevalence of having one or more risk factors is 80% * 22.5% =18.0%

- This means that 18.0% had one or more risk factor (and were in high or medium risk strata), and 82.0% had no risk factors (and were in low risk strata).

- We then assumed that among those with one or more risk factor, there was 20% overlap. As overlap means that, one has more than one risk factor, these patients were indeed among those in the high risk group.

- So for this laparoscopic cholecystectomy example more specifically, 20% of 18.0% is 3.6%, who have more than one risk factor and are at high risk. However, personal history of VTE (0,5%) also directly gives high risk. But prevalence of high risk is not 3.6% + 0.5% = 4.1% but it is 3.6% + 0.4% = 4.0%, because also 20% of those with personal history of VTE overlap.

- To get moderate (2x) estimate, amount of not overlapping patients with personal history of VTE is removed from the amount of patients with one risk factor. For laparoscopic cholecystectomy, amount of patients with one risk factor is 80% of 18.0%, that is 14.4%. Prevalence of moderate risk is therefore 14.4%-0.4%=14.0%.

- Hence, for this example low risk group was 82.0%, medium risk 14.0% and high risk 4.0%.

Our search did not reveal studies demonstrating convincing and replicable risk factors for bleeding. Therefore, we did not stratify bleeding risk by patient specific factors.

References

1. Lavikainen LI, Guyatt GH, Lee Y, et al. Systematic reviews of observational studies of Risk of Thrombosis and Bleeding in General and Gynecologic Surgery (ROTBIGGS): introduction and methodology. *Syst Rev*. Oct 8 2021;10(1):264. doi:10.1186/s13643-021-01814-2
2. Moher D, Liberati A, Tetzlaff J, Altman DG, Group P. Preferred reporting items for systematic reviews and meta-analyses: the PRISMA statement. *Ann Intern Med*. Aug 18 2009;151(4):264-9, W64. doi:10.7326/0003-4819-151-4-200908180-00135
3. Page MJ, McKenzie JE, Bossuyt PM, et al. The PRISMA 2020 statement: an updated guideline for reporting systematic reviews. *BMJ*. Mar 29 2021;372:n71. doi:10.1136/bmj.n71
4. Stroup DF, Berlin JA, Morton SC, et al. Meta-analysis of observational studies in epidemiology: a proposal for reporting. Meta-analysis Of Observational Studies in Epidemiology (MOOSE) group. *JAMA*. Apr 19 2000;283(15):2008-12. doi:10.1001/jama.283.15.2008
5. Singh T, Lavikainen L, Halme A, et al. Timing of symptomatic venous thromboembolism after surgery: A systematic review and meta-analysis (accepted for publication; in press). *BJS*. 2023;doi:10.1093/bjs/znad035
6. Tikkinen KAO, Craigie S, Agarwal A, et al. Procedure-specific Risks of Thrombosis and Bleeding in Urological Cancer Surgery: Systematic Review and Meta-analysis. *Eur Urol*. Feb 2018;73(2):242-251. doi:10.1016/j.eururo.2017.03.008
7. Tikkinen KAO, Craigie S, Agarwal A, et al. Procedure-specific Risks of Thrombosis and Bleeding in Urological Non-cancer Surgery: Systematic Review and Meta-analysis. *Eur Urol*. Feb 2018;73(2):236-241. doi:10.1016/j.eururo.2017.02.025
8. Tikkinen KA, Agarwal A, Craigie S, et al. Systematic reviews of observational studies of risk of thrombosis and bleeding in urological surgery (ROTBUS): introduction and methodology. *Syst Rev*. Dec 23 2014;3(1):150. doi:10.1186/2046-4053-3-150
9. Devereaux PJ, Mrkobrada M, Sessler DI, et al. Aspirin in patients undergoing noncardiac surgery. *N Engl J Med*. Apr 17 2014;370(16):1494-503. doi:10.1056/NEJMoa1401105
10. Mantz J, Samama CM, Tubach F, et al. Impact of preoperative maintenance or interruption of aspirin on thrombotic and bleeding events after elective non-cardiac surgery: the multicentre, randomized, blinded, placebo-controlled, STRATAGEM trial. *Br J Anaesth*. Dec 2011;107(6):899-910. doi:10.1093/bja/aer274
11. Prevention of pulmonary embolism and deep vein thrombosis with low dose aspirin: Pulmonary Embolism Prevention (PEP) trial. *Lancet*. Apr 15 2000;355(9212):1295-302.
12. Marcucci M, Etxeandia-Ikobaltzeta I, Yang S, et al. Benefits and harms of direct oral anticoagulation and low molecular weight heparin for thromboprophylaxis in patients undergoing non-cardiac surgery: systematic review and network meta-analysis of randomised trials. *BMJ*. Mar 9 2022;376:e066785. doi:10.1136/bmj-2021-066785
13. McAlpine K, Breau RH, Werlang P, et al. Timing of Perioperative Pharmacologic Thromboprophylaxis Initiation and its Effect on Venous Thromboembolism and Bleeding Outcomes: A Systematic Review and Meta-Analysis. *J Am Coll Surg*. Nov 2021;233(5):619-631 e14. doi:10.1016/j.jamcollsurg.2021.07.687
14. Caprini JA. Thrombosis risk assessment as a guide to quality patient care. *Dis Mon*. Feb-Mar 2005;51(2-3):70-8. doi:10.1016/j.disamonth.2005.02.003
15. Edmonds MJ, Crichton TJ, Runciman WB, Pradhan M. Evidence-based risk factors for postoperative deep vein thrombosis. *ANZ J Surg*. Dec 2004;74(12):1082-97. doi:10.1111/j.1445-1433.2004.03258.x
16. Hansson PO, Welin L, Tibblin G, Eriksson H. Deep vein thrombosis and pulmonary embolism in the general population. 'The Study of Men Born in 1913'. *Arch Intern Med*. Aug 11-25 1997;157(15):1665-70.
17. Pannucci CJ, Laird S, Dimick JB, Campbell DA, Henke PK. A validated risk model to predict 90-day VTE events in postsurgical patients. *Chest*. Mar 1 2014;145(3):567-573. doi:10.1378/chest.13-1553
18. Parkin L, Sweetland S, Balkwill A, Green J, Reeves G, Beral V. Body mass index, surgery, and risk of venous thromboembolism in middle-aged women: a cohort study. *Circulation*. Apr 17 2012;125(15):1897-904. doi:10.1161/circulationaha.111.063354
19. Rogers SO, Jr., Kilaru RK, Hosokawa P, Henderson WG, Zinner MJ, Khuri SF. Multivariable predictors of postoperative venous thromboembolic events after general and vascular surgery: results from the patient safety in surgery study. *J Am Coll Surg*. Jun 2007;204(6):1211-21. doi:10.1016/j.jamcollsurg.2007.02.072
20. Stein PD, Hull RD, Kayali F, Ghali WA, Alshab AK, Olson RE. Venous thromboembolism according to age: the impact of an aging population. *Arch Intern Med*. Nov 8 2004;164(20):2260-5. doi:10.1001/archinte.164.20.2260

21. Tosetto A, Frezzato M, Rodeghiero F. Prevalence and risk factors of non-fatal venous thromboembolism in the active population of the VITA Project. *J Thromb Haemost*. Aug 2003;1(8):1724-9. doi:10.1046/j.1538-7836.2003.00313.x
22. Weill-Engerer S, Meaume S, Lahlou A, et al. Risk factors for deep vein thrombosis in inpatients aged 65 and older: a case-control multicenter study. *J Am Geriatr Soc*. Aug 2004;52(8):1299-304. doi:10.1111/j.1532-5415.2004.52359.x
23. Guyatt GH, Oxman AD, Vist G, et al. GRADE guidelines: 4. Rating the quality of evidence--study limitations (risk of bias). *J Clin Epidemiol*. Apr 2011;64(4):407-15. doi:10.1016/j.jclinepi.2010.07.017
24. Hayden JA, van der Windt DA, Cartwright JL, Cote P, Bombardier C. Assessing bias in studies of prognostic factors. *Ann Intern Med*. Feb 19 2013;158(4):280-6. doi:10.7326/0003-4819-158-4-201302190-00009
25. Sterne JA, Hernan MA, Reeves BC, et al. ROBINS-I: a tool for assessing risk of bias in non-randomised studies of interventions. *BMJ*. Oct 12 2016;355:i4919. doi:10.1136/bmj.i4919
26. Kim SY, Park JE, Lee YJ, et al. Testing a tool for assessing the risk of bias for nonrandomized studies showed moderate reliability and promising validity. *J Clin Epidemiol*. Apr 2013;66(4):408-14. doi:10.1016/j.jclinepi.2012.09.016
27. Guyatt GH, Oxman AD, Vist GE, et al. GRADE: an emerging consensus on rating quality of evidence and strength of recommendations. *BMJ*. Apr 26 2008;336(7650):924-6. doi:10.1136/bmj.39489.470347.AD
28. Guyatt GH, Oxman AD, Kunz R, et al. What is "quality of evidence" and why is it important to clinicians? *BMJ*. May 3 2008;336(7651):995-8. doi:10.1136/bmj.39490.551019.BE
29. Iorio A, Spencer FA, Falavigna M, et al. Use of GRADE for assessment of evidence about prognosis: rating confidence in estimates of event rates in broad categories of patients. *Bmj*. Mar 16 2015;350:h870. doi:10.1136/bmj.h870
30. Columbo JA, Lambour AJ, Sundling RA, et al. A Meta-analysis of the Impact of Aspirin, Clopidogrel, and Dual Antiplatelet Therapy on Bleeding Complications in Noncardiac Surgery. *Ann Surg*. Jan 2018;267(1):1-10. doi:10.1097/SLA.0000000000002279
31. Bikdeli B, Chatterjee S, Desai NR, et al. Inferior Vena Cava Filters to Prevent Pulmonary Embolism: Systematic Review and Meta-Analysis. *J Am Coll Cardiol*. Sep 26 2017;70(13):1587-1597. doi:10.1016/j.jacc.2017.07.775
32. Abarca-Gómez L, Abdeen ZA, Hamid ZA, et al. Worldwide trends in body-mass index, underweight, overweight, and obesity from 1975 to 2016: a pooled analysis of 2416 population-based measurement studies in 128·9 million children, adolescents, and adults. *The Lancet*. 2017-12-01 2017;390(10113):2627-2642. doi:10.1016/s0140-6736(17)32129-3

9. Case fatality and estimates of fatal VTE and fatal bleeding

We estimated the case fatality rates by dividing the number of fatal PE events by the number of symptomatic VTE events using studies that had provided both estimates in both general and gynecologic surgery.

We estimated the case fatality for VTE from these studies that reported the following:

786 fatal VTE and 21133 symptomatic VTE.

Case fatality: 786/(786+21133)=3.585%

We used a similar approach to estimate the case fatality for major bleeding. Studies that reported the number of fatal bleeding, bleeding requiring reintervention and bleeding leading to transfusion reported the following:

7 fatal bleeding, 185 bleeding requiring reintervention and 755 bleeding leading to transfusion

Case fatality for bleeding requiring reintervention: 7/(7+185)=3.645%

Case fatality for bleeding leading to transfusion: 7/(7+755)=0.918%

For fatal bleeding we used primarily the bleeding requiring reintervention information and secondarily bleeding leading to transfusion information.

As fatal VTE and bleeding rates were very low, we estimated the fatal VTE and fatal major bleeding risks for procedures by taking case fatality rates of the overall reported risk of symptomatic events for the procedure.

Our median best estimates include fatal and non-fatal events. We therefore multiplied best estimate by 0.964 for non-fatal VTE, 0.036 for fatal VTE, 0.964 (reintervention) or 0.991 (transfusion) for non-fatal bleeding, and 0.036 (reintervention) or 0.009 (transfusion) for fatal bleeding.

8. Timing of VTE and bleeding during the first 90 post-operative days:

1. Proportion of cumulative risk of VTE by day since surgery during the first 90 post-operative days

We conducted a separate systematic review regarding the risk and time course of VTE by post-operative day[1].This systematic review provided estimates of occurrence of VTE on each day until 28 days post-operatively. Systematic review did not find any studies providing estimates for occurrence of VTE from 28 days to 90 days following surgery. Therefore, we used data from earlier ROTBUS systematic review for timing of VTE from 28 to 90 days post-operatively[2]. We combined these systematic reviews to develop model for time course of VTE 90 days post-surgery.

References

1. Singh T, Aaltonen R, Agarwal A, et al. Timing of symptomatic venous thromboembolism after surgery: A systematic review and meta-analysis (accepted for publication; in press). BJS 2023.
2. Tikkinen KA, Agarwal A, Craigie S, et al. Systematic reviews of observational studies of risk of thrombosis and bleeding in urological surgery (ROTBUS): introduction and methodology. *Syst Rev* 2014; 3(1):150.

1. Supplementary figure 1: Proportion of cumulative risk (%) of venous thromboembolism during the first 90 post-operative days

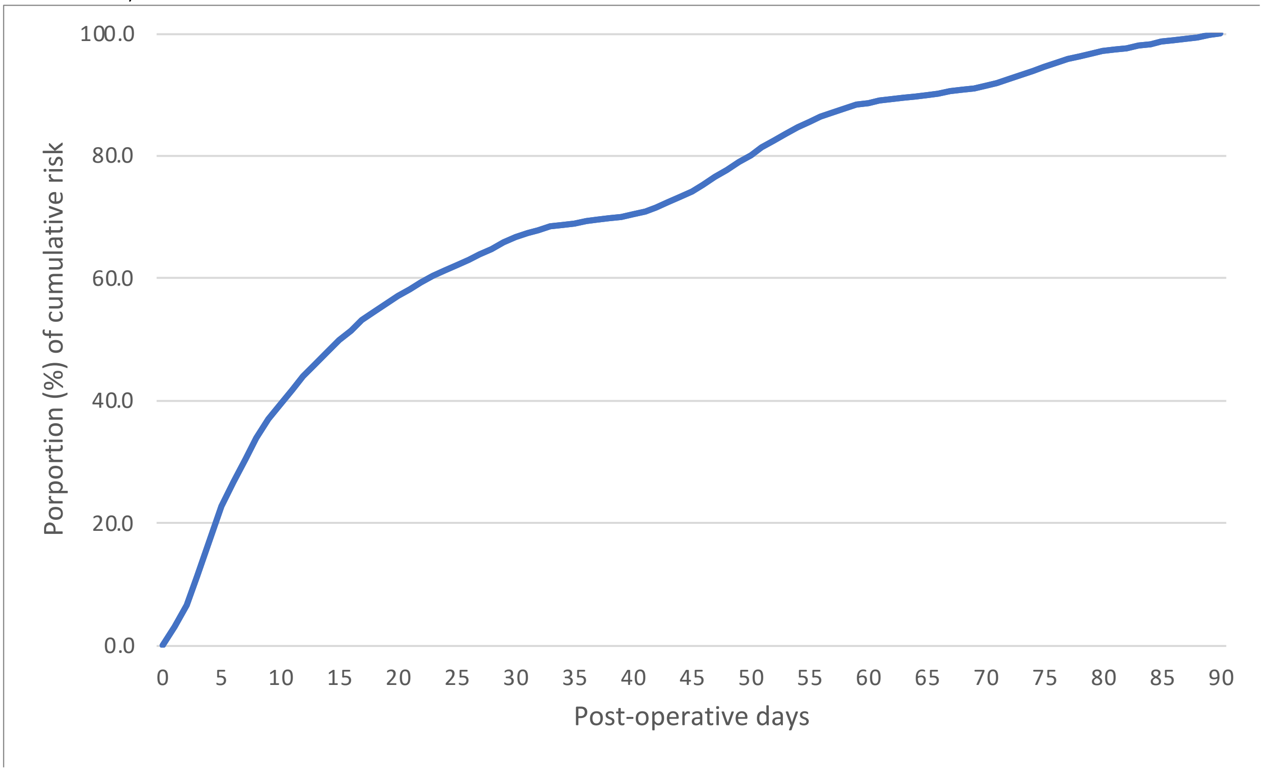

2. Supplementary table 22: Proportion of cumulative risk (%) of venous thromboembolism during the first 90 post-operative days

Day	Proportion (%)
0	0.0
1	3.0
2	6.7
3	11.4
4	17.1
5	22.7
6	26.8
7	30.5
8	33.9
9	37.0
10	39.5
11	41.9
12	44.0
13	46.0
14	48.0
15	49.8
16	51.5
17	53.1
18	54.5
19	55.9
20	57.1
21	58.2
22	59.3
23	60.3
24	61.2
25	62.1
26	63.0
27	63.9
28	64.8
29	65.8
30	66.7
31	67.4
32	67.9
33	68.4
34	68.7
35	69.0
36	69.3
37	69.5
38	69.8
39	70.1
40	70.5
41	71.0
42	71.6
43	72.4

44	73.3
45	74.2
46	75.3
47	76.5
48	77.7
49	78.9
50	80.1
51	81.3
52	82.5
53	83.6
54	84.7
55	85.6
56	86.4
57	87.2
58	87.8
59	88.3
60	88.7
61	89.0
62	89.3
63	89.5
64	89.8
65	90.0
66	90.2
67	90.5
68	90.8
69	91.1
70	91.5
71	92.0
72	92.6
73	93.2
74	93.9
75	94.6
76	95.2
77	95.8
78	96.3
79	96.7
80	97.1
81	97.4
82	97.7
83	98.0
84	98.3
85	98.6
86	98.8
87	99.1
88	99.4
89	99.7
90	100

2. Proportion of cumulative incidence of major bleeding by day since surgery during the first 90 post-operative days

We used cumulative incidence estimates at post-operative day 28 for our procedure-stratified estimates for the incidence of major bleeding. For the studies that did not report bleeding estimates using this interval, we created a new model using data from the placebo arm of a large pragmatic RCT to adjust the absolute bleeding risk by post-operative day[1]. However, as this study reported risk of both intraoperative and postoperative bleeds without distinguishing their proportions, we modeled the proportion of intraoperative bleeds with data from studies included in this ROTBIGGS review.

We identified 66 studies that reported both intraoperative and postoperative bleeds until 30 days (in both general and gynecologic surgery). In these studies, there were 964 bleeds, of which 335 (34.8%) were intraoperative bleeds leading to transfusion, 133 (13.8%) postoperative bleeds leading to reintervention, and 496 (51.5%) postoperative bleeds leading to transfusion. This suggests that 34.8% (335/964) of the intraoperative and 30-day postoperative bleeds are intraoperative.

In general surgery (27 studies), there were 394 bleeds, of which 136 (34.5%) were intraoperative bleeds leading to transfusion, 80 (20.3%) postoperative bleeds leading to reintervention, and 178 (45.2%) postoperative bleeds leading to transfusion. In gynecologic surgery (39 studies), there were 570 bleeds, of which 199 (34.9%) were intraoperative bleeds leading to transfusion, 53 (9.3%) postoperative bleeds leading to reintervention, and 318 (55.8%) postoperative bleeds leading to transfusion.

The large pragmatic RCT[1] reported that 46.8% of the 30-day bleeds happened on the day of surgery, however, without distinction of intra- and postoperative bleeds. From this estimate (46.8%) and from the total 30-day intraoperative and postoperative bleed estimate (100%), we subtracted the proportion of intraoperative bleeds (34.8%). Therefore, 18.5% of the total cumulative 30-day postoperative bleeds happen on the day of surgery (18.8% of 28-day post-operative bleeds).

This bleeding risk over time model shows that 86% of the 28-day bleeding events happen during the first week. Therefore, we assumed a constant risk of bleeding beyond the first 30 days, so that 80% of the 90 day bleeds happen during the first 30 days, and 20% during days 31-90.

References

1. Devereaux PJ, Mrkobrada M, Sessler DI, et al. Aspirin in patients undergoing noncardiac surgery. *N Engl J Med* 2014; 370(16):1494-503.

1. Supplementary figure 2: Proportion of cumulative incidence (%) of major bleeding during the first 90 post-operative days

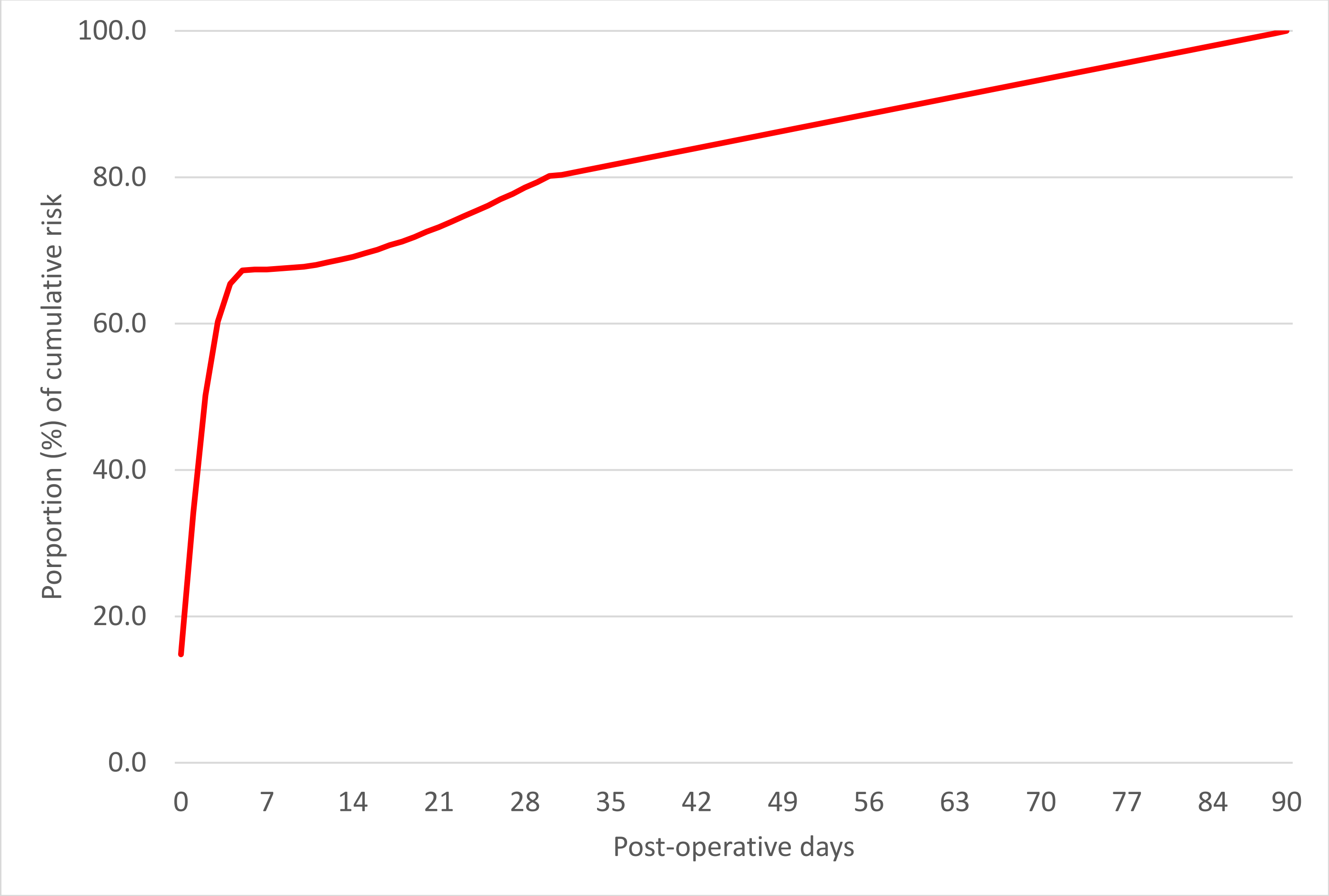

2. Supplementary table 23: Proportion of cumulative incidence (%) of major bleeding during the first 90 post-operative days

Day	Proportion (%)
0	14.8
1	34.1
2	50.2
3	60.3
4	65.4
5	67.3
6	67.4
7	67.4
8	67.5
9	67.6
10	67.8
11	68.0
12	68.4
13	68.8
14	69.1
15	69.6
16	70.1
17	70.7
18	71.2
19	71.8
20	72.6
21	73.2
22	73.9
23	74.7
24	75.4
25	76.1
26	77.0
27	77.7
28	78.6
29	79.3
30	80.2
31	80.3
32	80.7
33	81.0
34	81.3
35	81.7
36	82.0
37	82.3
38	82.7
39	83.0
40	83.3

**41**	83.7
**42**	84.0
**43**	84.3
**44**	84.7
**45**	85.0
**46**	85.3
**47**	85.7
**48**	86.0
**49**	86.3
**50**	86.7
**51**	87.0
**52**	87.3
**53**	87.7
**54**	88.0
**55**	88.3
**56**	88.7
**57**	89.0
**58**	89.3
**59**	89.7
**60**	90.0
**61**	90.3
**62**	90.6
**63**	91.0
**64**	91.3
**65**	91.6
**66**	92.0
**67**	92.3
**68**	92.6
**69**	93.0
**70**	93.3
**71**	93.6
**72**	94.0
**73**	94.3
**74**	94.6
**75**	95.0
**76**	95.3
**77**	95.6
**78**	96.0
**79**	96.3
**80**	96.6
**81**	97.0
**82**	97.3
**83**	97.6
**84**	98.0
**85**	98.3

**86**	98.6
**87**	99.0
**88**	99.3
**89**	99.6
**90**	100.0

9. Forest plots for effects of pharmacological and mechanical thromboprophylaxis on VTE and bleeding

1. Unfractionated heparin or low-molecular-weight heparin versus no prophylaxis: non-fatal pulmonary embolism

Study or Subgroup	UFH/LMWH Events	UFH/LMWH Total	Control Events	Control Total	Weight	Risk Ratio M-H, Random, 95% CI
**1.1.1 General Surgery**						
Abernethy 1974	0	63	5	62	1.2%	0.09 [0.01, 1.58]
Abraham-Inpijn 1979	0	20	4	20	1.2%	0.11 [0.01, 1.94]
Belch 1979	0	24	0	25		Not estimable
Bergqvist 1980	0	53	0	58		Not estimable
Caloghera 1984	0	40	0	40		Not estimable
Cerrato 1978	0	50	0	50		Not estimable
Clarke-Pearson 1983	4	95	0	105	1.1%	9.94 [0.54, 182.18]
Covey 1975	0	53	0	52		Not estimable
Gallus 1976	0	408	0	412		Not estimable
Gordon Smith 1972	2	105	0	51	1.0%	2.45 [0.12, 50.17]
Groote Shcuur 1979	10	323	15	323	15.5%	0.67 [0.30, 1.46]
Gruber 1977	1	119	4	113	2.0%	0.24 [0.03, 2.09]
Ho 1999	0	134	2	169	1.0%	0.25 [0.01, 5.20]
Jourdan 1984	0	21	0	21		Not estimable
Kakkar, 1977	13	2111	15	2137	17.4%	0.88 [0.42, 1.84]
Kakkar 1972	0	39	0	39		Not estimable
Kettunen 1974	0	83	4	117	1.1%	0.16 [0.01, 2.86]
Kiil 1978	0	650	6	663	1.2%	0.08 [0.00, 1.39]
Koppenhagen 1982	10	162	8	50	12.5%	0.39 [0.16, 0.92]
Kraytman 1976/77 (40/41)	0	23	2	27	1.1%	0.23 [0.01, 4.63]
Kraytman 1976 (40)	0	21	1	28	1.0%	0.44 [0.02, 10.28]
Kraytman 1977	0	25	0	22		Not estimable
Lahnborg 1974/75	9	58	24	54	21.3%	0.35 [0.18, 0.68]
Lahnborg 1976	0	24	0	24		Not estimable
Lawrence 1977	0	133	2	129	1.0%	0.19 [0.01, 4.00]
Loew1977	0	60	0	67		Not estimable
Marchetti 1983	0	30	1	30	1.0%	0.33 [0.01, 7.87]
Marchetti 1983 (48)	0	16	0	13		Not estimable
Multicentre1984/85	0	222	1	108	0.9%	0.16 [0.01, 3.97]
Multiunit 1974	1	128	2	128	1.7%	0.50 [0.05, 5.45]
Nicolaides 1972	0	128	0	122		Not estimable
Plante 1979	0	42	0	66		Not estimable
Ribaudo1975	0	75	2	75	1.0%	0.20 [0.01, 4.10]
Roberts 1975	0	39	0	45		Not estimable
Sagar 1974/75	2	264	4	236	3.4%	0.45 [0.08, 2.42]
Spebar 1981	0	24	0	19		Not estimable
Strand 1975	0	55	0	55		Not estimable
Taberner 1978	1	50	0	50	0.9%	3.00 [0.13, 71.92]
Torngren 1978/79	1	66	2	62	1.7%	0.47 [0.04, 5.05]
van Geloven 1977	2	74	9	80	4.3%	0.24 [0.05, 1.08]
Vinazzer 1980	0	402	0	404		Not estimable
Wu 1977	0	44	0	44		Not estimable
Ziemski 1979	0	30	0	20		Not estimable
**Subtotal (95% CI)**		**6586**		**6415**	**94.5%**	**0.46 [0.34, 0.64]**
Total events	56		113			
Heterogeneity: $Tau^2 = 0.00$; $Chi^2 = 18.06$, df = 22 (P = 0.70); $I^2 = 0\%$						
Test for overall effect: Z = 4.74 (P < 0.00001)						
**1.1.2 Urological**						
Allen 1978	0	30	0	30		Not estimable
Bejjani 1983	0	17	1	17	1.0%	0.33 [0.01, 7.65]
Coe 1978	0	28	1	24	1.0%	0.29 [0.01, 6.74]
Hedlund 1979/81	0	30	0	29		Not estimable
Kutnowski 1977, Kraytman	0	6	0	13		Not estimable
Patel 2020	2	251	5	250	3.6%	0.40 [0.08, 2.03]
Sebeseri 1975	0	34	0	31		Not estimable
Vandendris 1980, Kraytman	0	32	0	33		Not estimable
**Subtotal (95% CI)**		**428**		**427**	**5.5%**	**0.36 [0.10, 1.36]**
Total events	2		7			
Heterogeneity: $Tau^2 = 0.00$; $Chi^2 = 0.04$, df = 2 (P = 0.98); $I^2 = 0\%$						
Test for overall effect: Z = 1.50 (P = 0.13)						
**Total (95% CI)**		**7014**		**6842**	**100.0%**	**0.46 [0.34, 0.62]**
Total events	58		120			
Heterogeneity: $Tau^2 = 0.00$; $Chi^2 = 18.22$, df = 25 (P = 0.83); $I^2 = 0\%$						
Test for overall effect: Z = 4.96 (P < 0.00001)						
Test for subgroup differences: $Chi^2 = 0.12$, df = 1 (P = 0.73), $I^2 = 0\%$						

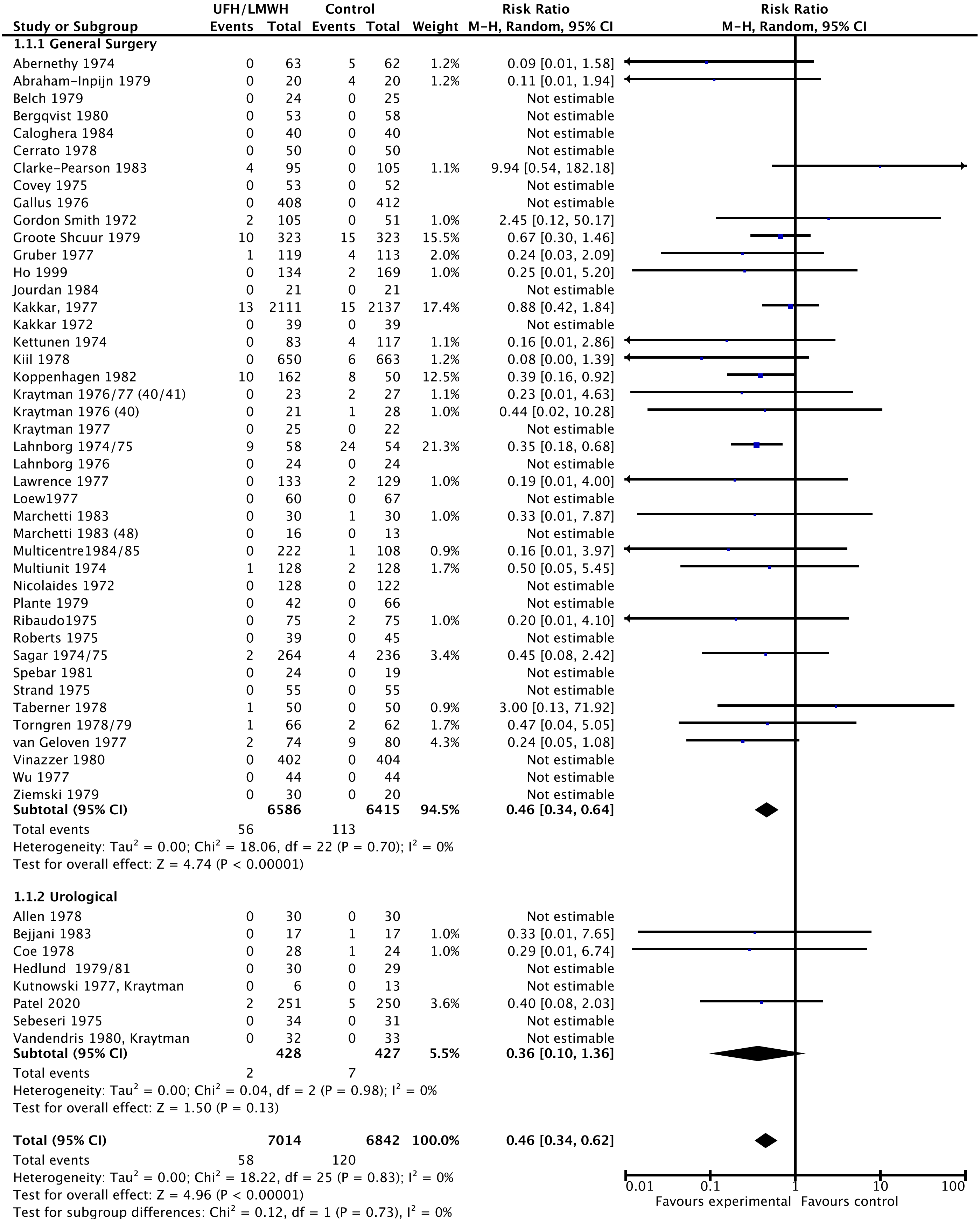

2. Unfractionated heparin or low-molecular-weight heparin versus no prophylaxis: non-fatal bleeding

Study or Subgroup	UFH/LMWH Events	UFH/LMWH Total	Control Events	Control Total	Weight	Risk Ratio M-H, Random, 95% CI
**1.2.1 General Surgery**						
Abernethy 1974	0	63	0	62		Not estimable
Abraham-Inpijn 1979	2	20	2	20	0.7%	1.00 [0.16, 6.42]
Belch 1979	8	24	1	25	0.6%	8.33 [1.13, 61.70]
Bergqvist 1980	2	53	0	58	0.3%	5.46 [0.27, 111.26]
Caloghera 1984	0	40	0	40		Not estimable
Cerrato 1978	10	50	6	50	2.8%	1.67 [0.66, 4.24]
Clarke-Pearson 1983	8	95	6	105	2.3%	1.47 [0.53, 4.09]
Covey 1975	14	53	10	52	4.7%	1.37 [0.67, 2.81]
Gordon Smith 1972	2	105	0	51	0.3%	2.45 [0.12, 50.17]
Groote Shcuur 1979	11	323	2	323	1.1%	5.50 [1.23, 24.62]
Gruber 1977	8	119	2	113	1.0%	3.80 [0.82, 17.51]
Ho 1999	9	134	3	169	1.5%	3.78 [1.04, 13.70]
Jourdan 1984	0	21	0	21		Not estimable
Kakkar, 1977	182	2111	127	2137	50.8%	1.45 [1.17, 1.80]
Kettunen 1974	2	83	0	117	0.3%	7.02 [0.34, 144.42]
Kiil 1978	3	650	8	663	1.4%	0.38 [0.10, 1.44]
Kraytman 1976/77 (40/41)	1	23	1	27	0.3%	1.17 [0.08, 17.74]
Kraytman 1976 (40)	0	21	1	28	0.2%	0.44 [0.02, 10.28]
Kraytman 1977	3	25	2	22	0.8%	1.32 [0.24, 7.19]
Kruse-Blinkenberg 1980	11	29	6	33	3.3%	2.09 [0.88, 4.93]
Lahnborg 1974/75	0	58	0	54		Not estimable
Lawrence 1977	4	133	4	129	1.3%	0.97 [0.25, 3.80]
Loew1977	0	60	0	67		Not estimable
Marchetti 1983	0	16	0	13		Not estimable
Marchetti 1983 (48)	0	30	0	30		Not estimable
Multicentre1984/85	7	222	2	108	1.0%	1.70 [0.36, 8.06]
Multiunit 1974	7	128	0	128	0.3%	15.00 [0.87, 259.91]
Nicolaides 1972	1	128	0	122	0.2%	2.86 [0.12, 69.55]
Plante 1979	0	42	0	66		Not estimable
Ribaudo1975	2	75	2	75	0.6%	1.00 [0.14, 6.91]
Roberts 1975	0	39	0	45		Not estimable
Sagar 1974/75	4	264	0	236	0.3%	8.05 [0.44, 148.72]
Spebar 1981	0	24	0	19		Not estimable
Taberner 1978	5	50	0	50	0.3%	11.00 [0.62, 193.80]
Torngren 1978/79	24	66	23	62	11.7%	0.98 [0.62, 1.54]
van Geloven 1977	1	74	1	80	0.3%	1.08 [0.07, 16.97]
Vinazzer 1980	11	402	3	404	1.5%	3.68 [1.04, 13.11]
Wu 1977	8	44	9	44	3.3%	0.89 [0.38, 2.09]
Ziemski 1979	1	30	0	20	0.2%	2.03 [0.09, 47.53]
**Subtotal (95% CI)**		**5927**		**5868**	**93.5%**	**1.52 [1.23, 1.88]**
Total events	351		221			
Heterogeneity: $Tau^2 = 0.02$; $Chi^2 = 30.44$, df = 28 (P = 0.34); $I^2 = 8\%$						
Test for overall effect: Z = 3.86 (P = 0.0001)						
**1.2.2 Urological**						
Allen 1978	6	30	0	30	0.3%	13.00 [0.76, 220.96]
Bejjani 1983	1	17	1	17	0.3%	1.00 [0.07, 14.72]
Coe 1978	14	28	6	24	3.9%	2.00 [0.91, 4.39]
Hedlund 1979/81	1	30	1	29	0.3%	0.97 [0.06, 14.74]
Kutnowski 1977, Kraytman	0	6	0	13		Not estimable
Patel 2020	4	251	2	250	0.9%	1.99 [0.37, 10.78]
Sebeseri 1975	2	34	0	31	0.3%	4.57 [0.23, 91.66]
Vandendris 1980, Kraytman	3	32	1	33	0.5%	3.09 [0.34, 28.21]
**Subtotal (95% CI)**		**428**		**427**	**6.5%**	**2.17 [1.18, 4.00]**
Total events	31		11			
Heterogeneity: $Tau^2 = 0.00$; $Chi^2 = 2.75$, df = 6 (P = 0.84); $I^2 = 0\%$						
Test for overall effect: Z = 2.49 (P = 0.01)						
**Total (95% CI)**		**6355**		**6295**	**100.0%**	**1.51 [1.29, 1.76]**
Total events	382		232			
Heterogeneity: $Tau^2 = 0.00$; $Chi^2 = 34.63$, df = 35 (P = 0.49); $I^2 = 0\%$						
Test for overall effect: Z = 5.16 (P < 0.00001)						
Test for subgroup differences: $Chi^2 = 1.18$, df = 1 (P = 0.28), $I^2 = 15.0\%$						

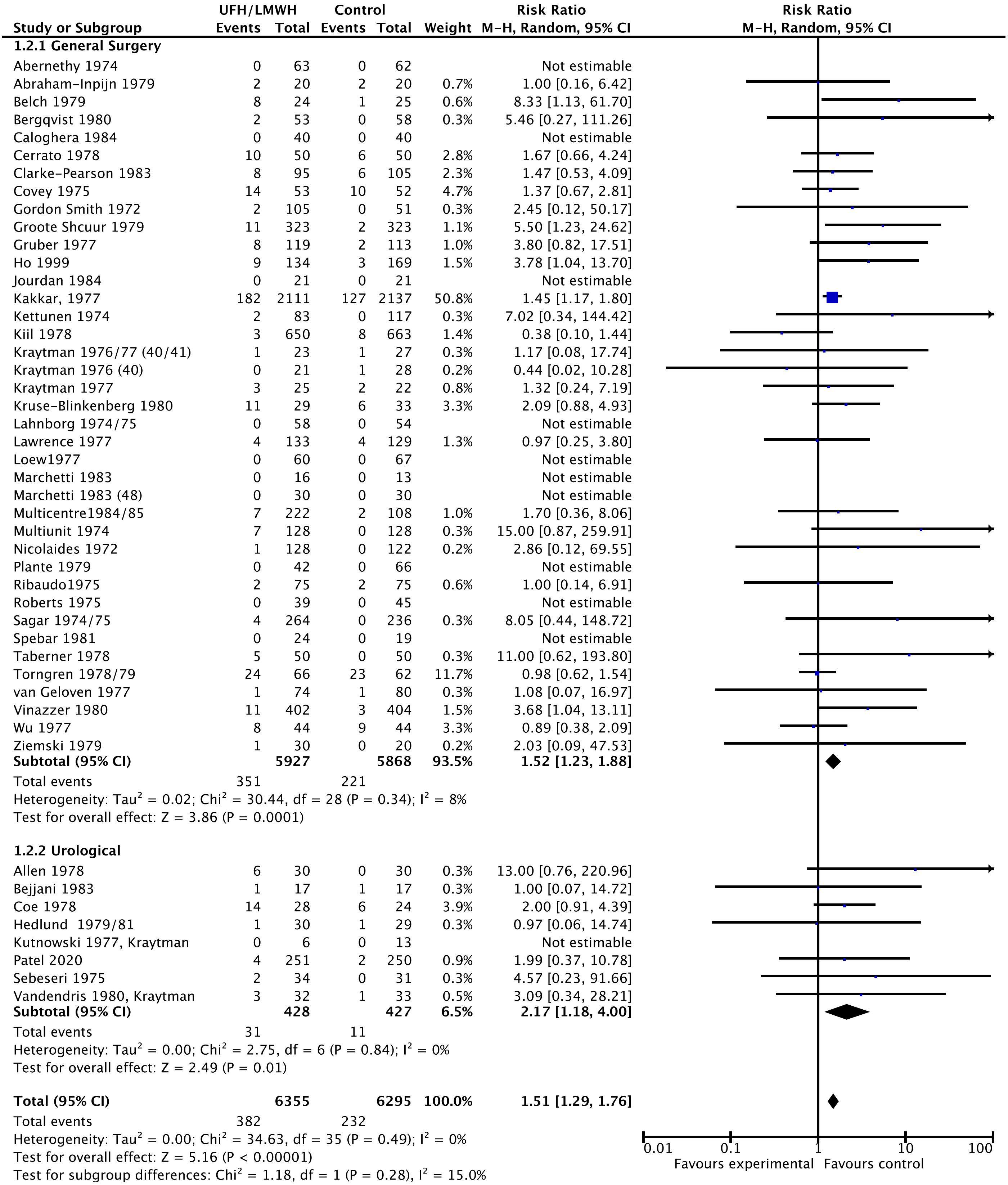

3. Unfractionated heparin or low-molecular-weight heparin versus no prophylaxis: fatal pulmonary embolism

Study or Subgroup	UFH/LMWH Events	UFH/LMWH Total	Control Events	Control Total	Weight	Risk Ratio M-H, Random, 95% CI
**1.3.1 General Surgery**						
Abernethy 1974	0	63	0	62		Not estimable
Abraham-Inpijn 1979	0	20	0	20		Not estimable
Belch 1979	0	24	0	25		Not estimable
Bergqvist 1980	0	53	0	58		Not estimable
Caloghera 1984	0	40	1	40	5.4%	0.33 [0.01, 7.95]
Cerrato 1978	0	50	0	50		Not estimable
Clarke-Pearson 1983	0	95	1	105	5.4%	0.37 [0.02, 8.93]
Covey 1975	0	53	0	52		Not estimable
Gordon Smith 1972	0	105	0	51		Not estimable
Groote Shcuur 1979	2	323	0	323	5.9%	5.00 [0.24, 103.74]
Gruber 1977	6	119	4	113	20.5%	1.42 [0.41, 4.92]
Ho 1999	0	134	1	169	5.4%	0.42 [0.02, 10.22]
Jourdan 1984	0	21	0	21		Not estimable
Kakkar, 1977	0	2111	15	2137	6.7%	0.03 [0.00, 0.55]
Kakkar 1972	0	39	0	39		Not estimable
Kettunen 1974	0	83	0	117		Not estimable
Kiil 1978	5	650	7	663	22.1%	0.73 [0.23, 2.28]
Kraytman 1976/77 (40/41)	0	23	0	27		Not estimable
Kraytman 1976 (40)	0	21	0	28		Not estimable
Kraytman 1977	0	25	0	22		Not estimable
Kruse-Blinkenberg 1980	0	29	0	33		Not estimable
Lahnborg 1974/75	0	58	0	54		Not estimable
Lahnborg 1976	0	24	0	24		Not estimable
Lawrence 1977	0	133	0	129		Not estimable
Loew1977	0	60	0	67		Not estimable
Marchetti 1983	0	16	0	13		Not estimable
Marchetti 1983 (48)	0	30	0	30		Not estimable
Multicentre1984/85	0	222	0	108		Not estimable
Multiunit 1974	0	128	2	128	5.9%	0.20 [0.01, 4.13]
Nicolaides 1972	0	128	0	122		Not estimable
Plante 1979	0	42	0	66		Not estimable
Ribaudo1975	0	75	0	75		Not estimable
Roberts 1975	0	39	0	45		Not estimable
Sagar 1974/75	0	264	8	236	6.5%	0.05 [0.00, 0.91]
Spebar 1981	1	24	0	19	5.5%	2.40 [0.10, 55.79]
Strand 1975	0	55	0	55		Not estimable
Taberner 1978	0	50	0	50		Not estimable
Torngren 1978/79	0	66	0	62		Not estimable
Vinazzer 1980	0	402	1	404	5.4%	0.33 [0.01, 8.20]
Wu 1977	0	44	0	44		Not estimable
Ziemski 1979	0	30	0	20		Not estimable
**Subtotal (95% CI)**		**5971**		**5906**	**94.5%**	**0.52 [0.22, 1.23]**
Total events	14		40			
Heterogeneity: $Tau^2 = 0.55$; $Chi^2 = 14.05$, df = 10 (P = 0.17); $I^2 = 29\%$						
Test for overall effect: Z = 1.49 (P = 0.14)						
**1.3.2 Urological**						
Allen 1978	0	30	0	30		Not estimable
Bejjani 1983	0	17	0	17		Not estimable
Coe 1978	0	28	0	24		Not estimable
Hedlund 1979/81	0	30	1	29	5.5%	0.32 [0.01, 7.61]
Kutnowski 1977, Kraytman	0	6	0	13		Not estimable
Patel 2020	0	251	0	250		Not estimable
Sebeseri 1975	0	34	0	31		Not estimable
Vandendris 1980, Kraytman	0	32	0	33		Not estimable
**Subtotal (95% CI)**		**428**		**427**	**5.5%**	**0.32 [0.01, 7.61]**
Total events	0		1			
Heterogeneity: Not applicable						
Test for overall effect: Z = 0.70 (P = 0.48)						
**Total (95% CI)**		**6399**		**6333**	**100.0%**	**0.52 [0.24, 1.15]**
Total events	14		41			
Heterogeneity: $Tau^2 = 0.40$; $Chi^2 = 14.13$, df = 11 (P = 0.23); $I^2 = 22\%$						
Test for overall effect: Z = 1.60 (P = 0.11)						
Test for subgroup differences: $Chi^2 = 0.08$, df = 1 (P = 0.78), $I^2 = 0\%$						

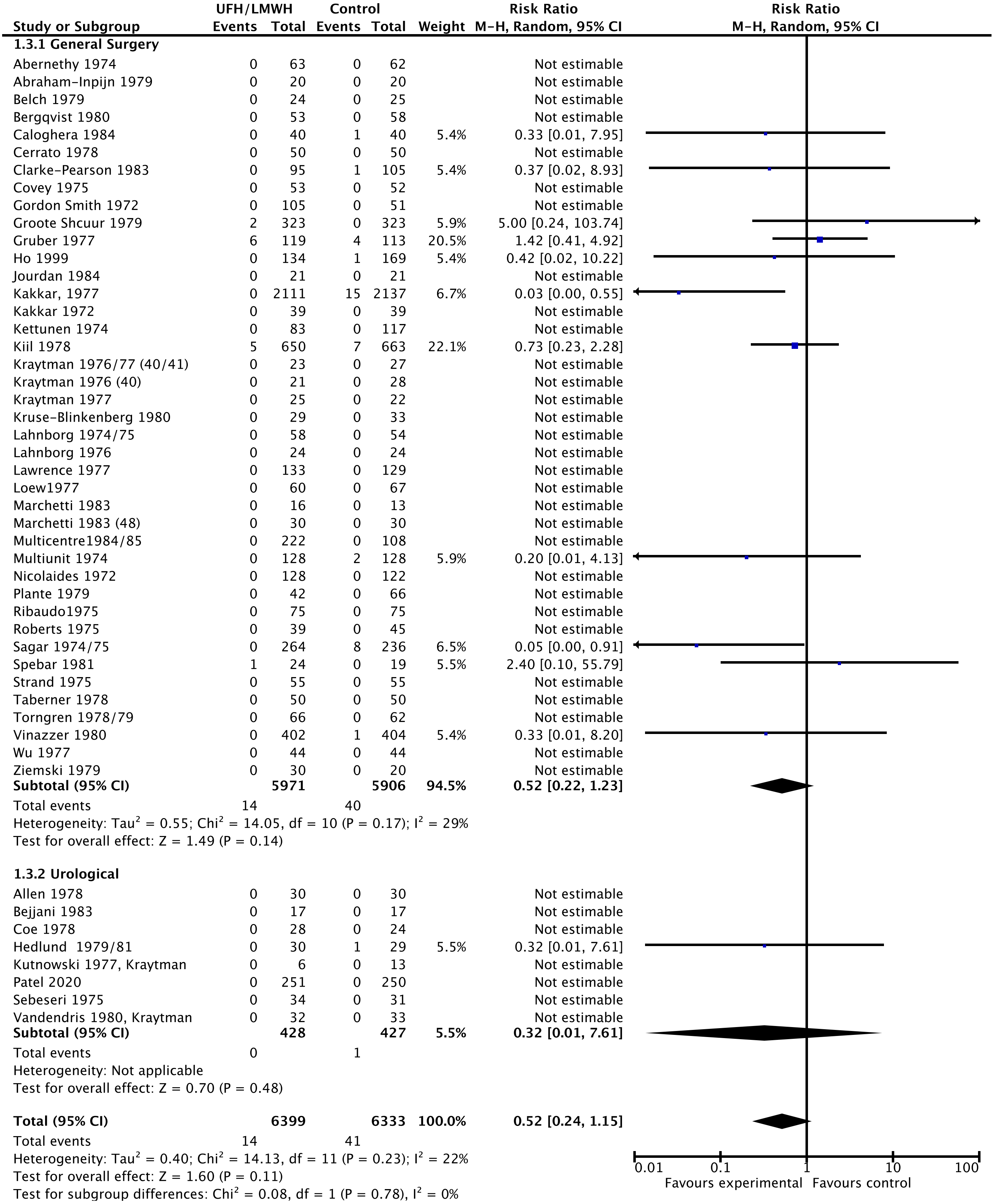

4. Unfractionated heparin or low-molecular-weight heparin versus no prophylaxis: fatal bleeding

Study or Subgroup	UFH/LMWH Events	UFH/LMWH Total	Control Events	Control Total	Weight	Risk Ratio M-H, Random, 95% CI
**1.4.1 General Surgery**						
Abernethy 1974	0	63	0	62		Not estimable
Abraham-Inpijn 1979	0	20	0	20		Not estimable
Belch 1979	1	24	0	25	10.2%	3.12 [0.13, 73.04]
Bergqvist 1980	0	53	0	58		Not estimable
Caloghera 1984	0	40	0	40		Not estimable
Cerrato 1978	0	50	0	50		Not estimable
Clarke-Pearson 1983	0	95	0	105		Not estimable
Covey 1975	0	53	0	52		Not estimable
Gordon Smith 1972	0	105	0	51		Not estimable
Groote Shcuur 1979	0	323	0	323		Not estimable
Gruber 1977	1	119	2	113	17.8%	0.47 [0.04, 5.16]
Jourdan 1984	0	21	0	21		Not estimable
Kakkar, 1977	0	2111	0	2137		Not estimable
Kakkar 1972	0	39	0	39		Not estimable
Kettunen 1974	0	83	0	117		Not estimable
Kiil 1978	2	650	3	663	31.7%	0.68 [0.11, 4.06]
Kraytman 1976/77 (40/41)	1	23	0	27	10.2%	3.50 [0.15, 81.99]
Kraytman 1976 (40)	0	21	1	28	10.2%	0.44 [0.02, 10.28]
Kraytman 1977	0	25	0	22		Not estimable
Kruse-Blinkenberg 1980	0	29	0	33		Not estimable
Lahnborg 1974/75	0	58	0	54		Not estimable
Lahnborg 1976	0	24	0	24		Not estimable
Lawrence 1977	0	133	0	129		Not estimable
Loew1977	0	60	0	67		Not estimable
Marchetti 1983	0	16	0	13		Not estimable
Marchetti 1983 (48)	0	30	0	30		Not estimable
Multicentre1984/85	0	222	0	108		Not estimable
Multiunit 1974	1	128	0	128	9.9%	3.00 [0.12, 72.96]
Nicolaides 1972	0	128	0	122		Not estimable
Plante 1979	0	42	0	66		Not estimable
Ribaudo1975	0	75	0	75		Not estimable
Roberts 1975	0	39	0	45		Not estimable
Sagar 1974/75	0	264	0	236		Not estimable
Spebar 1981	0	24	0	19		Not estimable
Strand 1975	0	55	0	55		Not estimable
Taberner 1978	0	50	0	50		Not estimable
Torngren 1978/79	0	66	0	62		Not estimable
Vinazzer 1980	0	402	0	404		Not estimable
Wu 1977	0	44	0	44		Not estimable
Ziemski 1979	0	30	0	20		Not estimable
**Subtotal (95% CI)**		**5837**		**5737**	**89.9%**	**1.02 [0.35, 2.93]**
Total events	6		6			
Heterogeneity: Tau² = 0.00; Chi² = 2.38, df = 5 (P = 0.79); I² = 0%						
Test for overall effect: Z = 0.03 (P = 0.98)						
**1.4.2 Urological**						
Allen 1978	0	30	0	30		Not estimable
Bejjani 1983	0	17	0	17		Not estimable
Coe 1978	0	28	0	24		Not estimable
Hedlund 1979/81	0	30	0	29		Not estimable
Kutnowski 1977, Kraytman	0	6	0	13		Not estimable
Patel 2020	0	250	0	251		Not estimable
Sebeseri 1975	0	34	0	31		Not estimable
Vandendris 1980, Kraytman	1	32	0	33	10.1%	3.09 [0.13, 73.19]
**Subtotal (95% CI)**		**427**		**428**	**10.1%**	**3.09 [0.13, 73.19]**
Total events	1		0			
Heterogeneity: Not applicable						
Test for overall effect: Z = 0.70 (P = 0.48)						
**Total (95% CI)**		**6264**		**6165**	**100.0%**	**1.14 [0.42, 3.11]**
Total events	7		6			
Heterogeneity: Tau² = 0.00; Chi² = 2.81, df = 6 (P = 0.83); I² = 0%						
Test for overall effect: Z = 0.25 (P = 0.80)						
Test for subgroup differences: Chi² = 0.43, df = 1 (P = 0.51), I² = 0%						

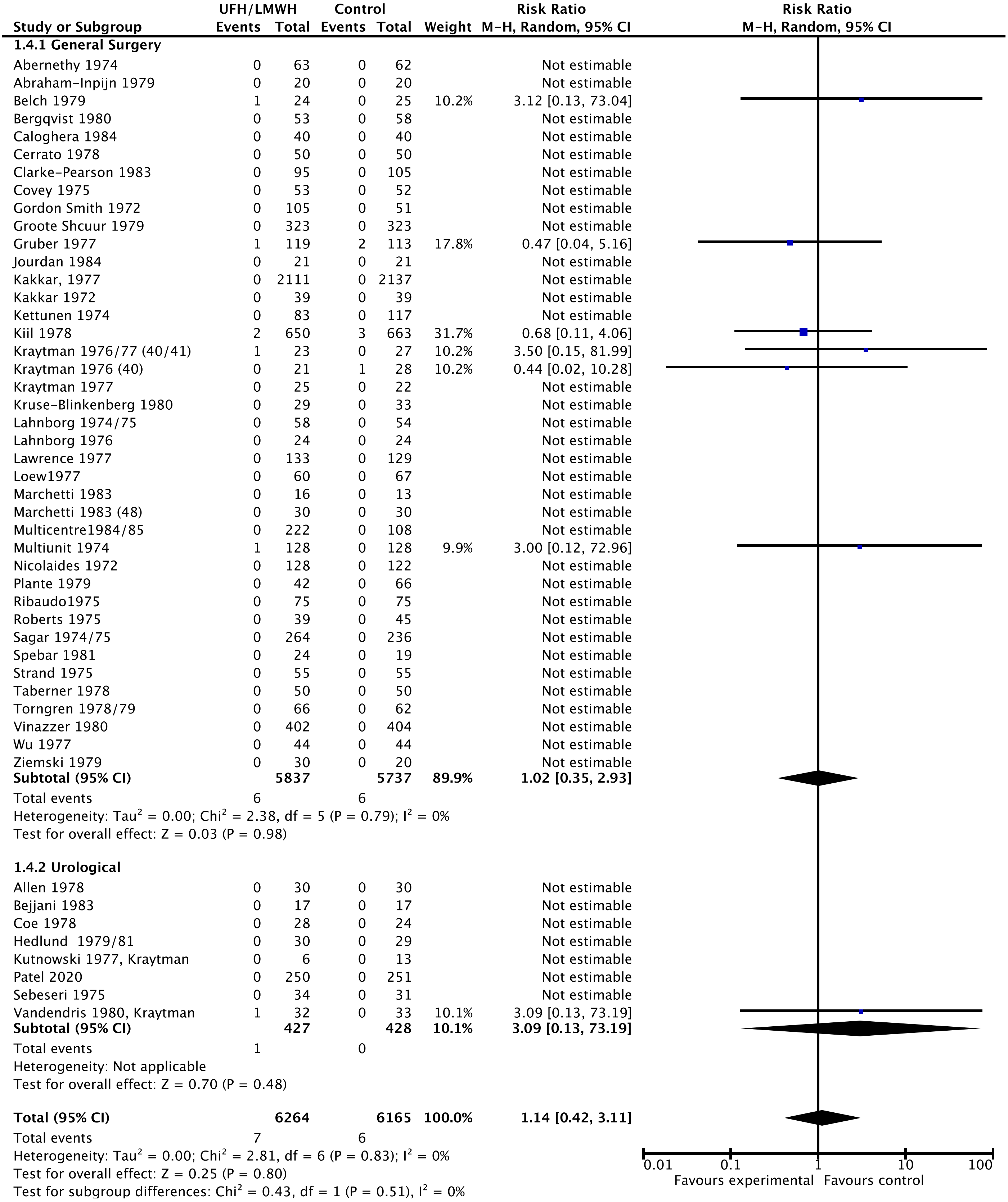

5. Unfractionated heparin or low-molecular-weight heparin versus no prophylaxis: death from any cause

Study or Subgroup	UFH/LMWH Events	UFH/LMWH Total	Control Events	Control Total	Weight	Risk Ratio M-H, Random, 95% CI
**1.5.1 General surgery**						
Abernethy 1974	0	63	0	62		Not estimable
Abraham-Inpijn 1979	0	20	0	20		Not estimable
Belch 1979	2	24	1	25	0.6%	2.08 [0.20, 21.50]
Bergqvist 1980	2	53	7	58	1.3%	0.31 [0.07, 1.44]
Caloghera 1984	0	40	1	0		Not estimable
Cerrato 1978	0	50	0	50		Not estimable
Clarke-Pearson 1983	1	95	2	105	0.5%	0.55 [0.05, 6.00]
Covey 1975	1	53	1	52	0.4%	0.98 [0.06, 15.28]
Gordon Smith 1972	0	105	0	51		Not estimable
Groote Shcuur 1979	13	323	17	323	6.2%	0.76 [0.38, 1.55]
Gruber 1977	10	119	13	113	5.0%	0.73 [0.33, 1.60]
Jourdan 1984	0	21	0	21		Not estimable
Kakkar, 1977	77	2111	94	2137	35.3%	0.83 [0.62, 1.11]
Kakkar 1972	0	39	0	39		Not estimable
Kettunen 1974	0	83	0	117		Not estimable
Kiil 1978	45	650	50	663	20.4%	0.92 [0.62, 1.35]
Kraytman 1976/77 (40/41)	2	23	3	27	1.1%	0.78 [0.14, 4.29]
Kraytman 1976 (40)	2	21	3	28	1.1%	0.89 [0.16, 4.85]
Kraytman 1977	3	25	1	22	0.6%	2.64 [0.30, 23.58]
Kruse-Blinkenberg 1980	0	29	0	33		Not estimable
Lahnborg 1974/75	0	58	0	54		Not estimable
Lahnborg 1976	0	24	0	24		Not estimable
Lawrence 1977	0	133	0	129		Not estimable
Loew1977	0	60	0	67		Not estimable
Marchetti 1983	0	16	0	13		Not estimable
Marchetti 1983 (48)	0	30	1	30	0.3%	0.33 [0.01, 7.87]
Multicentre1984/85	0	222	0	108		Not estimable
Multiunit 1974	7	128	7	128	3.0%	1.00 [0.36, 2.77]
Nicolaides 1972	0	128	0	122		Not estimable
Plante 1979	0	42	0	66		Not estimable
Ribaudo1975	0	75	0	75		Not estimable
Roberts 1975	1	39	0	45	0.3%	3.45 [0.14, 82.34]
Sagar 1974/75	28	264	38	236	14.8%	0.66 [0.42, 1.04]
Spebar 1981	1	24	0	19	0.3%	2.40 [0.10, 55.79]
Strand 1975	0	55	0	55		Not estimable
Taberner 1978	0	50	0	50		Not estimable
Torngren 1978/79	1	66	2	62	0.5%	0.47 [0.04, 5.05]
Vinazzer 1980	17	402	18	404	7.3%	0.95 [0.50, 1.82]
Wu 1977	1	44	0	44	0.3%	3.00 [0.13, 71.70]
Ziemski 1979	0	30	0	20		Not estimable
**Subtotal (95% CI)**		**5837**		**5697**	**99.4%**	**0.82 [0.69, 0.98]**
Total events	214		259			
Heterogeneity: $Tau^2 = 0.00$; $Chi^2 = 7.45$, df = 18 (P = 0.99); $I^2 = 0\%$						
Test for overall effect: Z = 2.15 (P = 0.03)						
**1.5.2 Urological**						
Allen 1978	0	30	0	30		Not estimable
Bejjani 1983	0	17	0	17		Not estimable
Coe 1978	0	28	0	24		Not estimable
Hedlund 1979/81	0	30	1	29	0.3%	0.32 [0.01, 7.61]
Kutnowski 1977, Kraytman	0	6	0	13		Not estimable
Sebeseri 1975	0	34	0	31		Not estimable
Vandendris 1980, Kraytman	1	32	0	33	0.3%	3.09 [0.13, 73.19]
**Subtotal (95% CI)**		**177**		**177**	**0.6%**	**1.00 [0.11, 9.33]**
Total events	1		1			
Heterogeneity: $Tau^2 = 0.00$; $Chi^2 = 0.98$, df = 1 (P = 0.32); $I^2 = 0\%$						
Test for overall effect: Z = 0.00 (P = 1.00)						
**Total (95% CI)**		**6014**		**5874**	**100.0%**	**0.83 [0.69, 0.98]**
Total events	215		260			
Heterogeneity: $Tau^2 = 0.00$; $Chi^2 = 8.46$, df = 20 (P = 0.99); $I^2 = 0\%$						
Test for overall effect: Z = 2.14 (P = 0.03)						
Test for subgroup differences: $Chi^2 = 0.03$, df = 1 (P = 0.87), $I^2 = 0\%$						

Risk Ratio
M-H, Random, 95% CI

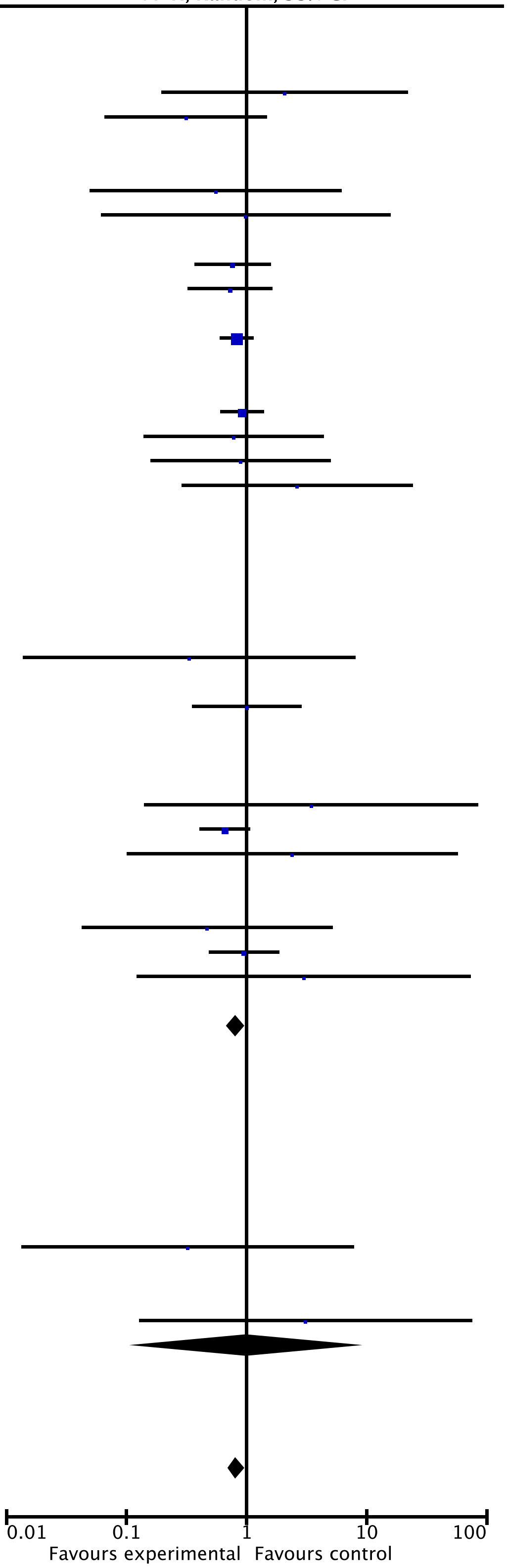

6. Elastic stockings versus no prophylaxis: deep vein thrombosis on surveillance

Study or Subgroup	ES Events	ES Total	Control Events	Control Total	Weight	Risk Ratio M-H, Random, 95% CI
**1.1.2 Abd, Gyn, or urology**						
Allan, 1983	15	97	37	103	40.1%	0.43 [0.25, 0.73]
Holford, 1976	11	48	23	47	32.0%	0.47 [0.26, 0.85]
Scurr, 1977	8	70	26	70	21.9%	0.31 [0.15, 0.63]
Tsapogas, 1981	2	51	6	44	4.7%	0.29 [0.06, 1.35]
Turner, 1984	0	104	4	92	1.3%	0.10 [0.01, 1.80]
**Subtotal (95% CI)**		**370**		**356**	**100.0%**	**0.40 [0.28, 0.55]**
Total events	36		96			
Heterogeneity: Tau² = 0.00; Chi² = 1.96, df = 4 (P = 0.74); I² = 0%						
Test for overall effect: Z = 5.40 (P < 0.00001)						
**Total (95% CI)**		**370**		**356**	**100.0%**	**0.40 [0.28, 0.55]**
Total events	36		96			
Heterogeneity: Tau² = 0.00; Chi² = 1.96, df = 4 (P = 0.74); I² = 0%						
Test for overall effect: Z = 5.40 (P < 0.00001)						
Test for subgroup differences: Not applicable						

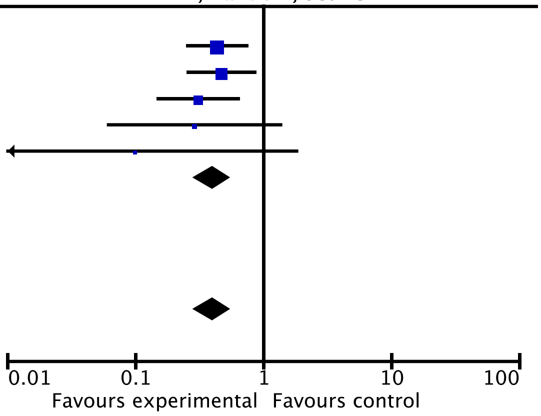

7. Elastic stockings versus no prophylaxis: pulmonary embolism

Study or Subgroup	ES Events	ES Total	Control Events	Control Total	Weight	Risk Ratio M-H, Random, 95% CI
**1.3.3 Abd, Gyn, or urology**						
Holford, 1976	1	48	0	47	100.0%	2.94 [0.12, 70.37]
Turner, 1984	0	104	0	92		Not estimable
**Subtotal (95% CI)**		**152**		**139**	**100.0%**	**2.94 [0.12, 70.37]**
Total events	1		0			
Heterogeneity: Not applicable						
Test for overall effect: Z = 0.67 (P = 0.51)						
**Total (95% CI)**		**152**		**139**	**100.0%**	**2.94 [0.12, 70.37]**
Total events	1		0			
Heterogeneity: Not applicable						
Test for overall effect: Z = 0.67 (P = 0.51)						
Test for subgroup differences: Not applicable						

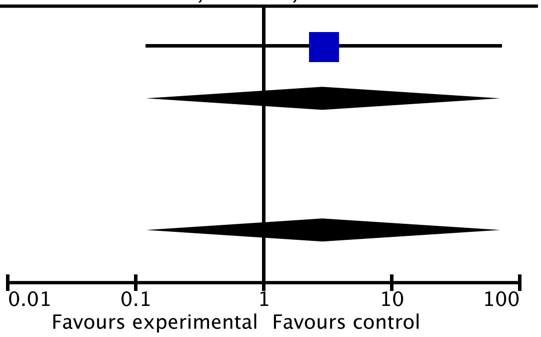

8. Elastic stockings versus no prophylaxis: any venous thromboembolism

Study or Subgroup	ES Events	ES Total	Control Events	Control Total	Weight	Risk Ratio M-H, Random, 95% CI
**1.4.3 Abd, Gyn, or urology**						
Allan, 1983	15	97	37	103	39.8%	0.43 [0.25, 0.73]
Holford, 1976	11	48	24	47	32.4%	0.45 [0.25, 0.81]
Scurr, 1977	8	70	26	70	21.7%	0.31 [0.15, 0.63]
Tsapogas, 1981	2	51	6	44	4.7%	0.29 [0.06, 1.35]
Turner, 1984	0	104	4	92	1.3%	0.10 [0.01, 1.80]
**Subtotal (95% CI)**		**370**		**356**	**100.0%**	**0.39 [0.28, 0.55]**
Total events	36		97			
Heterogeneity: Tau² = 0.00; Chi² = 1.82, df = 4 (P = 0.77); I² = 0%						
Test for overall effect: Z = 5.49 (P < 0.00001)						
**Total (95% CI)**		**370**		**356**	**100.0%**	**0.39 [0.28, 0.55]**
Total events	36		97			
Heterogeneity: Tau² = 0.00; Chi² = 1.82, df = 4 (P = 0.77); I² = 0%						
Test for overall effect: Z = 5.49 (P < 0.00001)						
Test for subgroup differences: Not applicable						

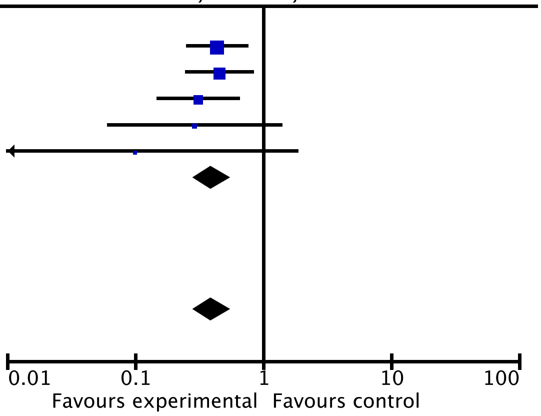

9. Intermittent pneumatic compression device versus no prophylaxis: deep vein thrombosis on surveillance

Study or Subgroup	IPC Events	IPC Total	Control Events	Control Total	Weight	Risk Ratio M-H, Random, 95% CI
**2.1.3 Abd, Gyn, or urology**						
Butson, 1982	6	62	4	57	18.3%	1.38 [0.41, 4.64]
Clarke-Pearson, 1984 (1)	14	97	11	97	24.5%	1.27 [0.61, 2.66]
Clarke-Pearson, 1984 (2)	5	55	17	52	22.0%	0.28 [0.11, 0.70]
Coe, 1978	2	29	6	24	15.0%	0.28 [0.06, 1.24]
Inada, 1983	4	110	16	110	20.2%	0.25 [0.09, 0.72]
**Subtotal (95% CI)**		**353**		**340**	**100.0%**	**0.53 [0.23, 1.20]**
Total events	31		54			
Heterogeneity: $Tau^2 = 0.56$; $Chi^2 = 12.19$, df = 4 (P = 0.02); $I^2 = 67\%$						
Test for overall effect: Z = 1.53 (P = 0.13)						
**Total (95% CI)**		**353**		**340**	**100.0%**	**0.53 [0.23, 1.20]**
Total events	31		54			
Heterogeneity: $Tau^2 = 0.56$; $Chi^2 = 12.19$, df = 4 (P = 0.02); $I^2 = 67\%$						
Test for overall effect: Z = 1.53 (P = 0.13)						
Test for subgroup differences: Not applicable						

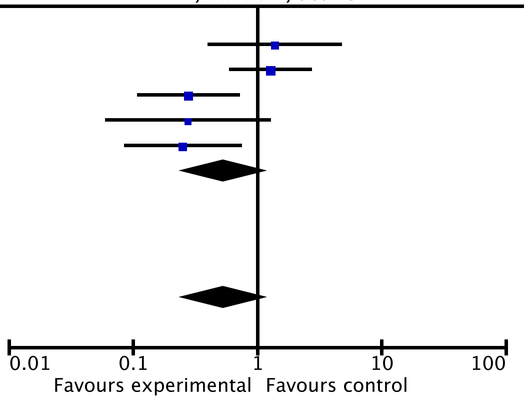

10. Intermittent pneumatic compression device versus no prophylaxis: pulmonary embolism

Study or Subgroup	IPC Events	IPC Total	Control Events	Control Total	Weight	Risk Ratio M-H, Random, 95% CI
**2.3.3 Abd, Gyn, or urology**						
Butson, 1982	1	62	1	57	20.1%	0.92 [0.06, 14.36]
Clarke-Pearson, 1984 (1)	4	97	1	97	32.2%	4.00 [0.46, 35.14]
Clarke-Pearson, 1984 (2)	2	55	1	52	27.1%	1.89 [0.18, 20.23]
Coe, 1978	1	29	1	24	20.6%	0.83 [0.05, 12.54]
**Subtotal (95% CI)**		**243**		**230**	**100.0%**	**1.76 [0.51, 6.03]**
Total events	8		4			
Heterogeneity: $Tau^2 = 0.00$; $Chi^2 = 1.07$, df = 3 (P = 0.78); $I^2 = 0\%$						
Test for overall effect: Z = 0.89 (P = 0.37)						
**Total (95% CI)**		**243**		**230**	**100.0%**	**1.76 [0.51, 6.03]**
Total events	8		4			
Heterogeneity: $Tau^2 = 0.00$; $Chi^2 = 1.07$, df = 3 (P = 0.78); $I^2 = 0\%$						
Test for overall effect: Z = 0.89 (P = 0.37)						
Test for subgroup differences: Not applicable						

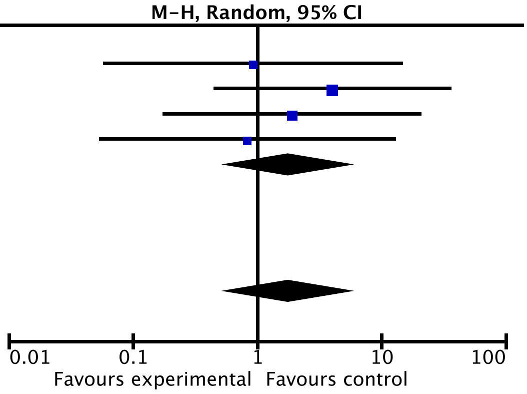

11. Intermittent pneumatic compression device versus no prophylaxis: any venous thromboembolism

Study or Subgroup	IPC Events	IPC Total	Control Events	Control Total	Weight	Risk Ratio M-H, Random, 95% CI
**2.4.3 Abd, Gyn, or urology**						
Butson, 1982	7	62	5	57	18.4%	1.29 [0.43, 3.83]
Clarke-Pearson, 1984 (1)	18	97	12	97	23.9%	1.50 [0.76, 2.94]
Clarke-Pearson, 1984 (2)	7	55	18	52	22.4%	0.37 [0.17, 0.81]
Coe, 1978	3	29	7	24	16.6%	0.35 [0.10, 1.23]
Inada, 1983	4	110	16	110	18.7%	0.25 [0.09, 0.72]
**Subtotal (95% CI)**		**353**		**340**	**100.0%**	**0.60 [0.28, 1.29]**
Total events	39		58			
Heterogeneity: $Tau^2 = 0.53$; $Chi^2 = 13.48$, df = 4 (P = 0.009); $I^2 = 70\%$						
Test for overall effect: Z = 1.31 (P = 0.19)						
**Total (95% CI)**		**353**		**340**	**100.0%**	**0.60 [0.28, 1.29]**
Total events	39		58			
Heterogeneity: $Tau^2 = 0.53$; $Chi^2 = 13.48$, df = 4 (P = 0.009); $I^2 = 70\%$						
Test for overall effect: Z = 1.31 (P = 0.19)						
Test for subgroup differences: Not applicable						

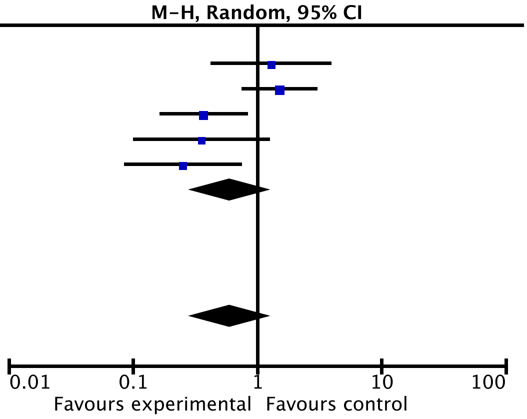

12. Any mechanical prophylaxis versus no prophylaxis: deep vein thrombosis on surveillance

Study or Subgroup	Mechanical Events	Mechanical Total	Control Events	Control Total	Weight	Risk Ratio M-H, Random, 95% CI
**4.1.1 ES**						
Allan, 1983	15	97	37	103	10.6%	0.43 [0.25, 0.73]
Borow, 1981	14	91	32	89	10.2%	0.43 [0.25, 0.75]
Holford, 1976	11	48	23	47	9.6%	0.47 [0.26, 0.85]
Rosengarten, 1970	8	25	8	25	7.1%	1.00 [0.45, 2.24]
Scurr, 1977	8	70	26	70	8.1%	0.31 [0.15, 0.63]
Tsapogas, 1981	2	51	6	44	2.8%	0.29 [0.06, 1.35]
Turner, 1984	0	104	4	92	0.9%	0.10 [0.01, 1.80]
**Subtotal (95% CI)**		**486**		**470**	**49.3%**	**0.45 [0.34, 0.59]**
Total events	58		136			
Heterogeneity: $Tau^2 = 0.01$; $Chi^2 = 6.37$, df = 6 (P = 0.38); $I^2 = 6\%$						
Test for overall effect: Z = 5.56 (P < 0.00001)						
**4.1.2 IPC**						
Borow, 1981	9	79	32	89	8.6%	0.32 [0.16, 0.62]
Butson, 1982	6	62	4	57	4.1%	1.38 [0.41, 4.64]
Clark, 1974	0	36	7	36	1.0%	0.07 [0.00, 1.13]
Clarke-Pearson, 1984 (1)	14	97	11	97	7.9%	1.27 [0.61, 2.66]
Clarke-Pearson, 1984 (2)	5	55	17	52	6.1%	0.28 [0.11, 0.70]
Coe, 1978	2	29	6	24	2.9%	0.28 [0.06, 1.24]
Hillis, 1972 (Barnett H)	1	20	8	20	1.8%	0.13 [0.02, 0.91]
Hillis, 1972 (Hamrsmith)	6	50	15	50	6.6%	0.40 [0.17, 0.95]
Inada, 1983	4	110	16	110	5.0%	0.25 [0.09, 0.72]
Roberts, 1974	6	94	27	104	6.8%	0.25 [0.11, 0.57]
**Subtotal (95% CI)**		**632**		**639**	**50.7%**	**0.38 [0.23, 0.63]**
Total events	53		143			
Heterogeneity: $Tau^2 = 0.32$; $Chi^2 = 19.85$, df = 9 (P = 0.02); $I^2 = 55\%$						
Test for overall effect: Z = 3.80 (P = 0.0001)						
**Total (95% CI)**		**1118**		**1109**	**100.0%**	**0.42 [0.32, 0.56]**
Total events	111		279			
Heterogeneity: $Tau^2 = 0.12$; $Chi^2 = 26.45$, df = 16 (P = 0.05); $I^2 = 40\%$						
Test for overall effect: Z = 6.01 (P < 0.00001)						
Test for subgroup differences: $Chi^2 = 0.29$, df = 1 (P = 0.59), $I^2 = 0\%$						

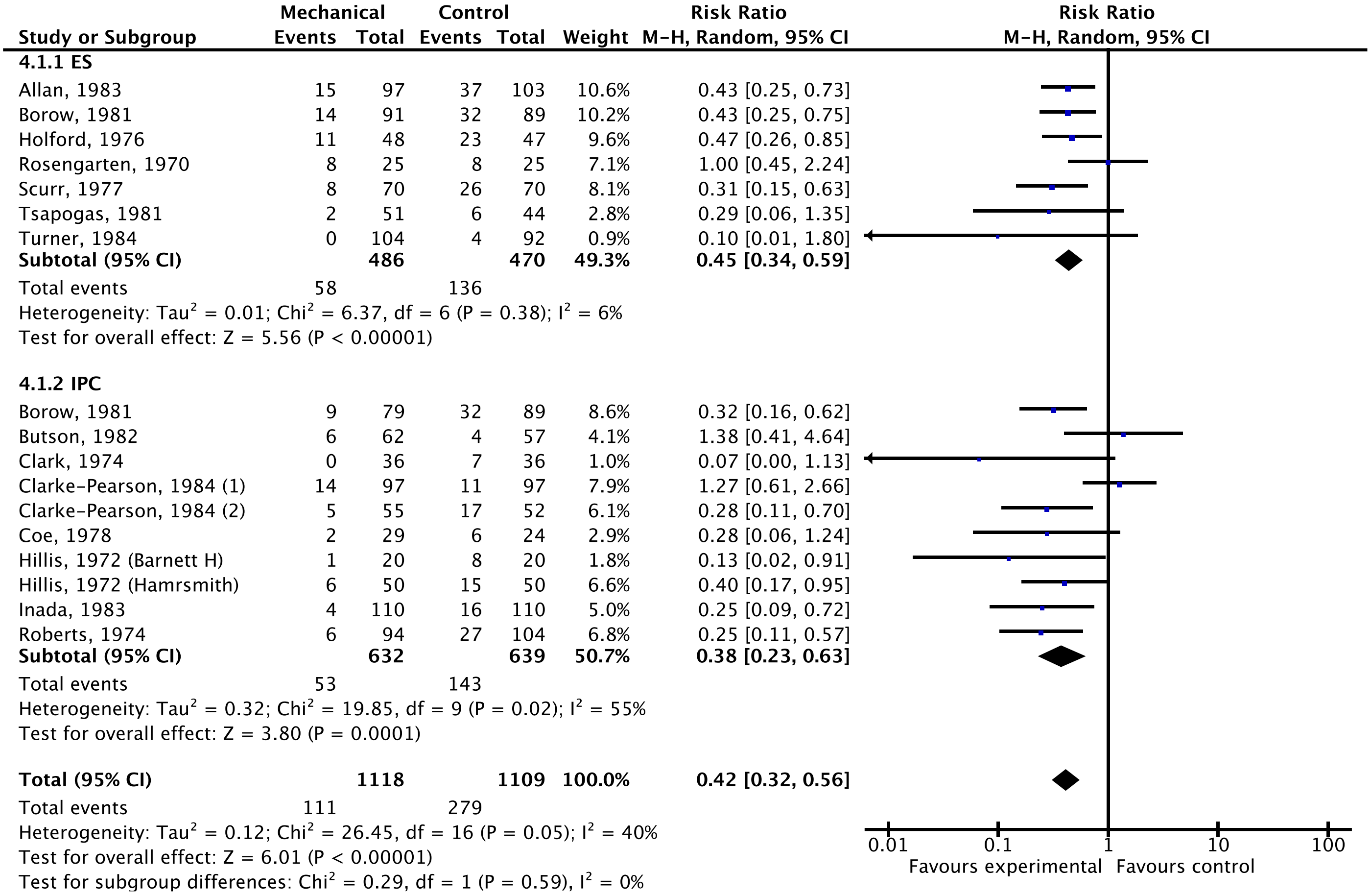

13. Any mechanical prophylaxis versus no prophylaxis: pulmonary embolism

Study or Subgroup	Mechanical Events	Mechanical Total	Control Events	Control Total	Weight	Risk Ratio M-H, Random, 95% CI
**4.3.1 ES**						
Allan, 1983	0	91	1	89	9.5%	0.33 [0.01, 7.90]
Borow, 1981	1	48	0	47	9.6%	2.94 [0.12, 70.37]
Turner, 1984	0	104	0	92		Not estimable
**Subtotal (95% CI)**		**243**		**228**	**19.2%**	**0.98 [0.10, 9.32]**
Total events	1		1			
Heterogeneity: Tau² = 0.00; Chi² = 0.92, df = 1 (P = 0.34); I² = 0%						
Test for overall effect: Z = 0.02 (P = 0.99)						
**4.3.2 IPC**						
Borow, 1981	2	79	1	89	17.1%	2.25 [0.21, 24.38]
Butson, 1982	1	62	1	57	12.8%	0.92 [0.06, 14.36]
Clarke-Pearson, 1984 (1)	4	97	1	97	20.5%	4.00 [0.46, 35.14]
Clarke-Pearson, 1984 (2)	2	55	1	52	17.3%	1.89 [0.18, 20.23]
Coe, 1978	1	29	1	24	13.1%	0.83 [0.05, 12.54]
**Subtotal (95% CI)**		**322**		**319**	**80.8%**	**1.85 [0.62, 5.53]**
Total events	10		5			
Heterogeneity: Tau² = 0.00; Chi² = 1.10, df = 4 (P = 0.89); I² = 0%						
Test for overall effect: Z = 1.10 (P = 0.27)						
**Total (95% CI)**		**565**		**547**	**100.0%**	**1.64 [0.61, 4.39]**
Total events	11		6			
Heterogeneity: Tau² = 0.00; Chi² = 2.26, df = 6 (P = 0.89); I² = 0%						
Test for overall effect: Z = 0.98 (P = 0.32)						
Test for subgroup differences: Chi² = 0.25, df = 1 (P = 0.62), I² = 0%						

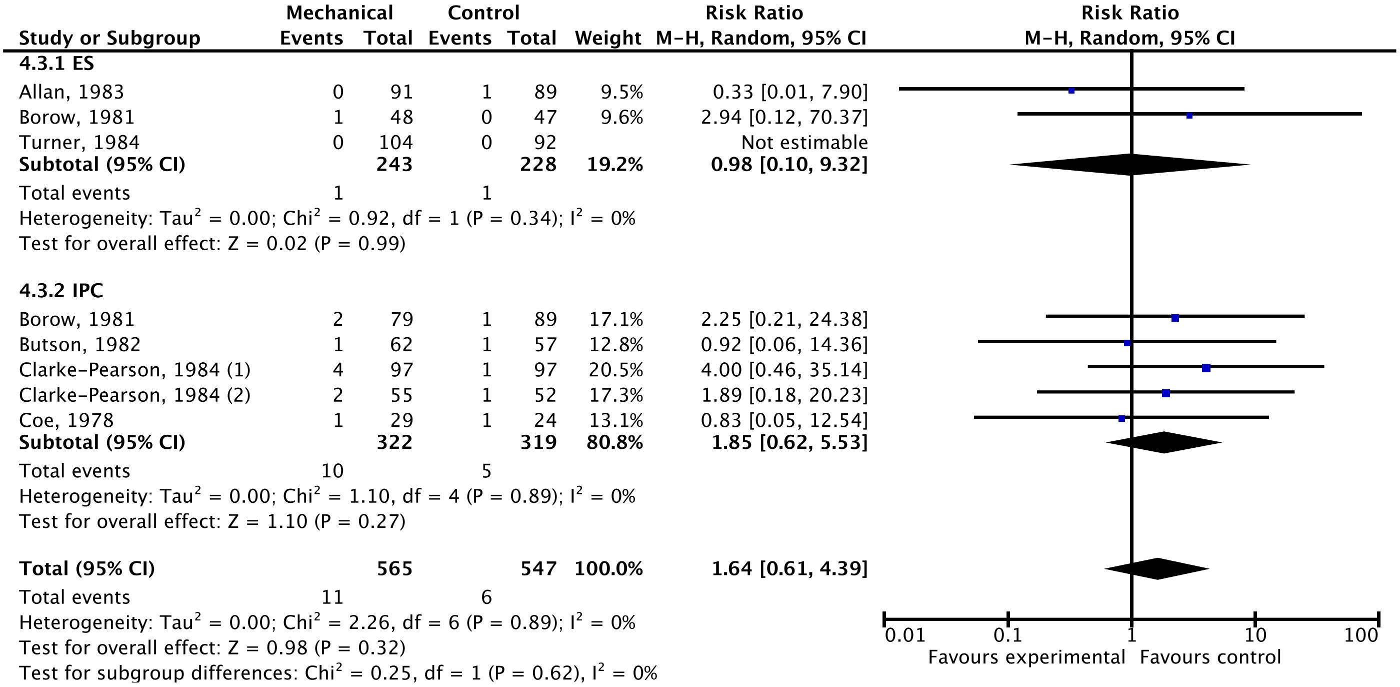

14. Any mechanical prophylaxis versus no prophylaxis: any venous thromboembolism

Study or Subgroup	Mechanical Events	Mechanical Total	Control Events	Control Total	Weight	Risk Ratio M-H, Random, 95% CI
**4.4.1 ES**						
Allan, 1983	15	97	37	103	9.6%	0.43 [0.25, 0.73]
Borow, 1981	14	91	33	89	9.3%	0.41 [0.24, 0.72]
Holford, 1976	11	48	24	47	8.9%	0.45 [0.25, 0.81]
Rosengarten, 1970	8	25	8	25	6.8%	1.00 [0.45, 2.24]
Scurr, 1977	8	70	26	70	7.6%	0.31 [0.15, 0.63]
Tsapogas, 1981	2	51	6	44	2.8%	0.29 [0.06, 1.35]
Turner, 1984	0	104	4	92	0.9%	0.10 [0.01, 1.80]
**Subtotal (95% CI)**		**486**		**470**	**46.0%**	**0.44 [0.33, 0.58]**
Total events	58		138			
Heterogeneity: Tau² = 0.01; Chi² = 6.37, df = 6 (P = 0.38); I² = 6%						
Test for overall effect: Z = 5.69 (P < 0.00001)						
**4.4.2 IPC**						
Borow, 1981	11	79	33	89	8.7%	0.38 [0.20, 0.69]
Butson, 1982	7	62	5	57	4.8%	1.29 [0.43, 3.83]
Bynke, 1987	0	31	6	31	1.0%	0.08 [0.00, 1.31]
Clark, 1974	0	36	7	36	1.0%	0.07 [0.00, 1.13]
Clarke-Pearson, 1984 (1)	18	97	12	97	8.0%	1.50 [0.76, 2.94]
Clarke-Pearson, 1984 (2)	7	55	18	52	7.0%	0.37 [0.17, 0.81]
Coe, 1978	3	29	7	24	4.0%	0.35 [0.10, 1.23]
Hillis, 1972 (Barnett H)	1	20	8	20	1.9%	0.13 [0.02, 0.91]
Hillis, 1972 (Hamrsmith)	6	50	15	50	6.3%	0.40 [0.17, 0.95]
Inada, 1983	4	110	16	110	4.9%	0.25 [0.09, 0.72]
Roberts, 1974	6	94	27	104	6.5%	0.25 [0.11, 0.57]
**Subtotal (95% CI)**		**663**		**670**	**54.0%**	**0.40 [0.24, 0.67]**
Total events	63		154			
Heterogeneity: Tau² = 0.37; Chi² = 25.01, df = 10 (P = 0.005); I² = 60%						
Test for overall effect: Z = 3.56 (P = 0.0004)						
**Total (95% CI)**		**1149**		**1140**	**100.0%**	**0.43 [0.32, 0.58]**
Total events	121		292			
Heterogeneity: Tau² = 0.16; Chi² = 31.37, df = 17 (P = 0.02); I² = 46%						
Test for overall effect: Z = 5.64 (P < 0.00001)						
Test for subgroup differences: Chi² = 0.09, df = 1 (P = 0.77), I² = 0%						

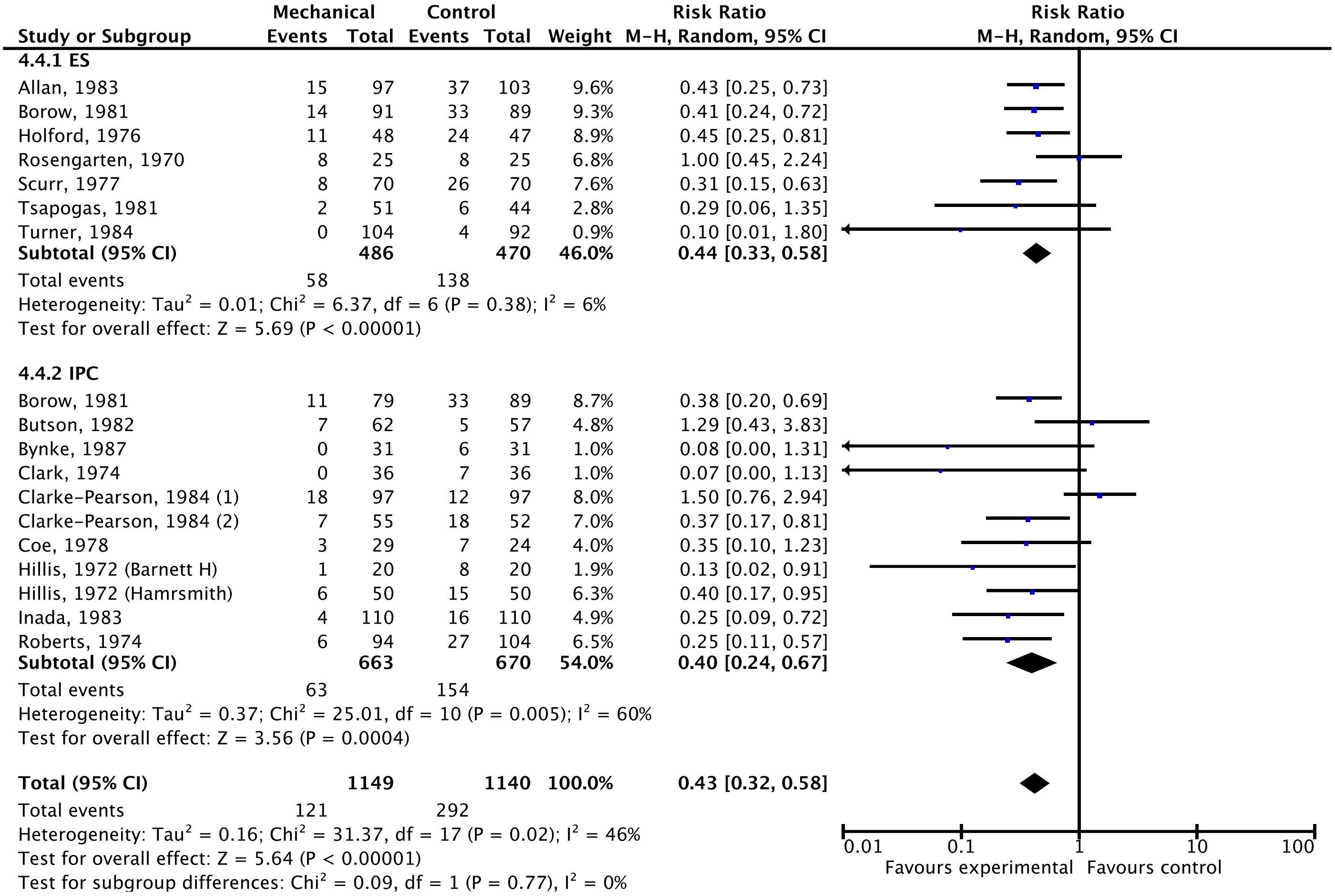

15. Any mechanical plus any pharmacological versus any pharmacological: deep vein thrombosis on surveillance

Study or Subgroup	Mech+Pharm Events	Mech+Pharm Total	Pharmacologic Events	Pharmacologic Total	Weight	Risk Ratio M-H, Random, 95% CI
**7.5.2 ES + UFH or LMWH vs UFH or LMWH alone**						
Kalodiki, 1996	8	32	12	32	12.4%	0.67 [0.32, 1.41]
Rasmussen, 1988	23	89	25	85	22.4%	0.88 [0.54, 1.42]
Shalhoub, 2020	12	921	14	937	12.0%	0.87 [0.41, 1.88]
Torngren, 1980	4	98	12	98	6.6%	0.33 [0.11, 1.00]
Wille-Jorgensen, 1985	1	86	7	90	2.0%	0.15 [0.02, 1.19]
**Subtotal (95% CI)**		**1226**		**1242**	**55.4%**	**0.68 [0.45, 1.04]**
Total events	48		70			
Heterogeneity: $Tau^2 = 0.05$; $Chi^2 = 5.19$, df = 4 (P = 0.27); $I^2 = 23\%$						
Test for overall effect: Z = 1.80 (P = 0.07)						
**7.5.3 IPC + UFH or LMWH vs. UFH or LMWH alone**						
Arabi, 2019	37	957	41	985	25.0%	0.93 [0.60, 1.44]
**Subtotal (95% CI)**		**957**		**985**	**25.0%**	**0.93 [0.60, 1.44]**
Total events	37		41			
Heterogeneity: Not applicable						
Test for overall effect: Z = 0.33 (P = 0.74)						
**7.5.4 ES + Dextran versus Dextran Alone**						
Bergqvist, 1984	0	80	8	80	1.1%	0.06 [0.00, 1.00]
**Subtotal (95% CI)**		**80**		**80**	**1.1%**	**0.06 [0.00, 1.00]**
Total events	0		8			
Heterogeneity: Not applicable						
Test for overall effect: Z = 1.96 (P = 0.05)						
**7.5.5 IPC + Dextran versus Dextran Alone**						
Smith, 1978	18	97	21	97	18.5%	0.86 [0.49, 1.51]
**Subtotal (95% CI)**		**97**		**97**	**18.5%**	**0.86 [0.49, 1.51]**
Total events	18		21			
Heterogeneity: Not applicable						
Test for overall effect: Z = 0.54 (P = 0.59)						
**Total (95% CI)**		**2360**		**2404**	**100.0%**	**0.75 [0.56, 1.02]**
Total events	103		140			
Heterogeneity: $Tau^2 = 0.05$; $Chi^2 = 9.44$, df = 7 (P = 0.22); $I^2 = 26\%$						
Test for overall effect: Z = 1.85 (P = 0.06)						
Test for subgroup differences: $Chi^2 = 4.29$, df = 3 (P = 0.23), $I^2 = 30.1\%$						

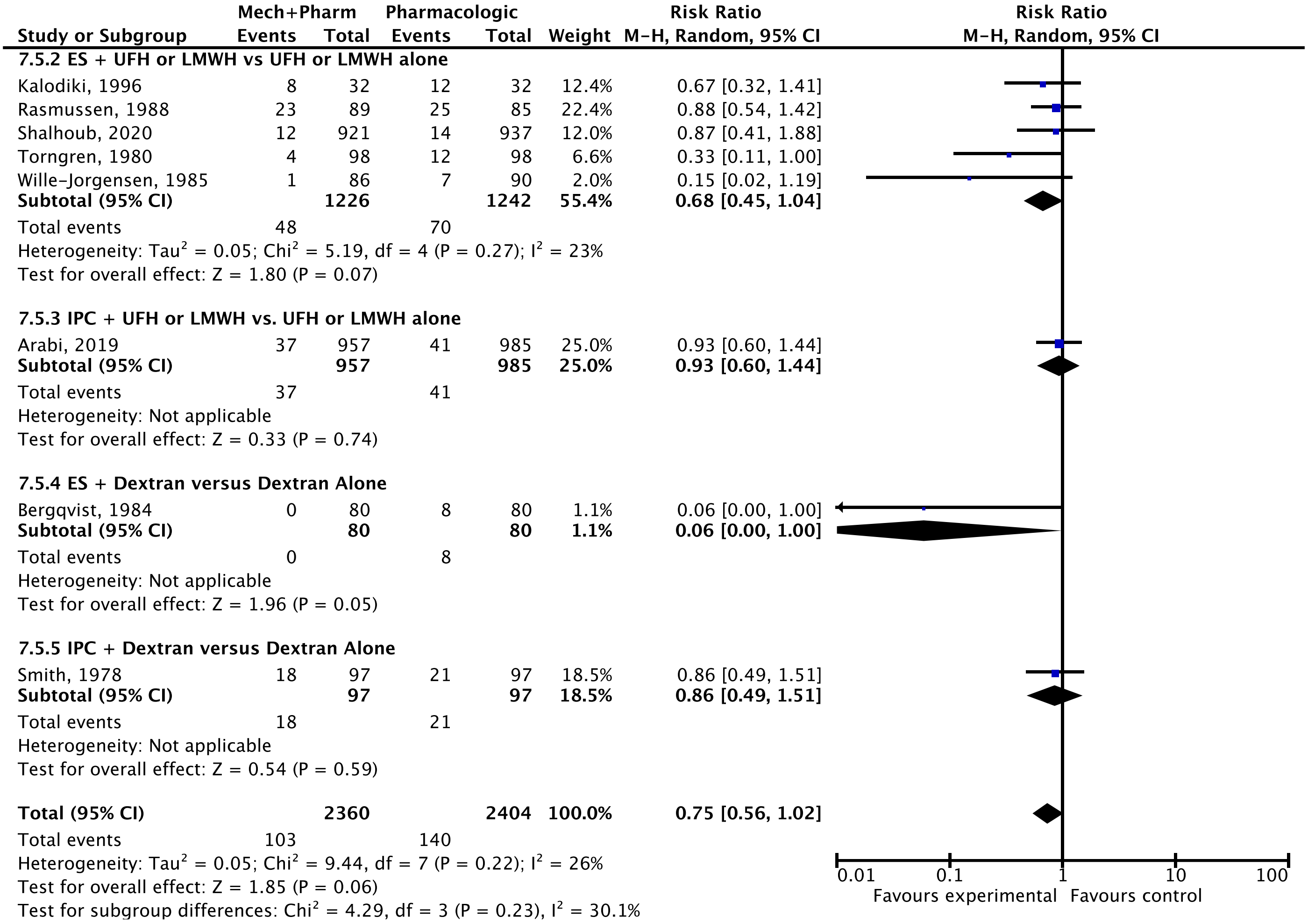

16. Any mechanical plus any pharmacological versus any pharmacological: symptomatic deep vein thrombosis

Study or Subgroup	Mech+Pharm Events	Mech+Pharm Total	Pharmacologic Events	Pharmacologic Total	Weight	Risk Ratio M-H, Random, 95% CI
**7.6.2 ES + UFH or LMWH vs UFH or LMWH alone**						
Rasmussen, 1988	1	89	0	85	26.6%	2.87 [0.12, 69.41]
Shalhoub, 2020	1	921	2	937	46.9%	0.51 [0.05, 5.60]
**Subtotal (95% CI)**		**1010**		**1022**	**73.4%**	**0.95 [0.14, 6.46]**
Total events	2		2			
Heterogeneity: Tau^2 = 0.00; Chi^2 = 0.72, df = 1 (P = 0.39); I^2 = 0%						
Test for overall effect: Z = 0.05 (P = 0.96)						
**7.6.4 ES + Dextran versus Dextran Alone**						
Bergqvist, 1984	0	80	1	80	26.6%	0.33 [0.01, 8.06]
**Subtotal (95% CI)**		**80**		**80**	**26.6%**	**0.33 [0.01, 8.06]**
Total events	0		1			
Heterogeneity: Not applicable						
Test for overall effect: Z = 0.68 (P = 0.50)						
**Total (95% CI)**		**1090**		**1102**	**100.0%**	**0.72 [0.14, 3.72]**
Total events	2		3			
Heterogeneity: Tau^2 = 0.00; Chi^2 = 1.03, df = 2 (P = 0.60); I^2 = 0%						
Test for overall effect: Z = 0.39 (P = 0.69)						
Test for subgroup differences: Chi^2 = 0.31, df = 1 (P = 0.58), I^2 = 0%						

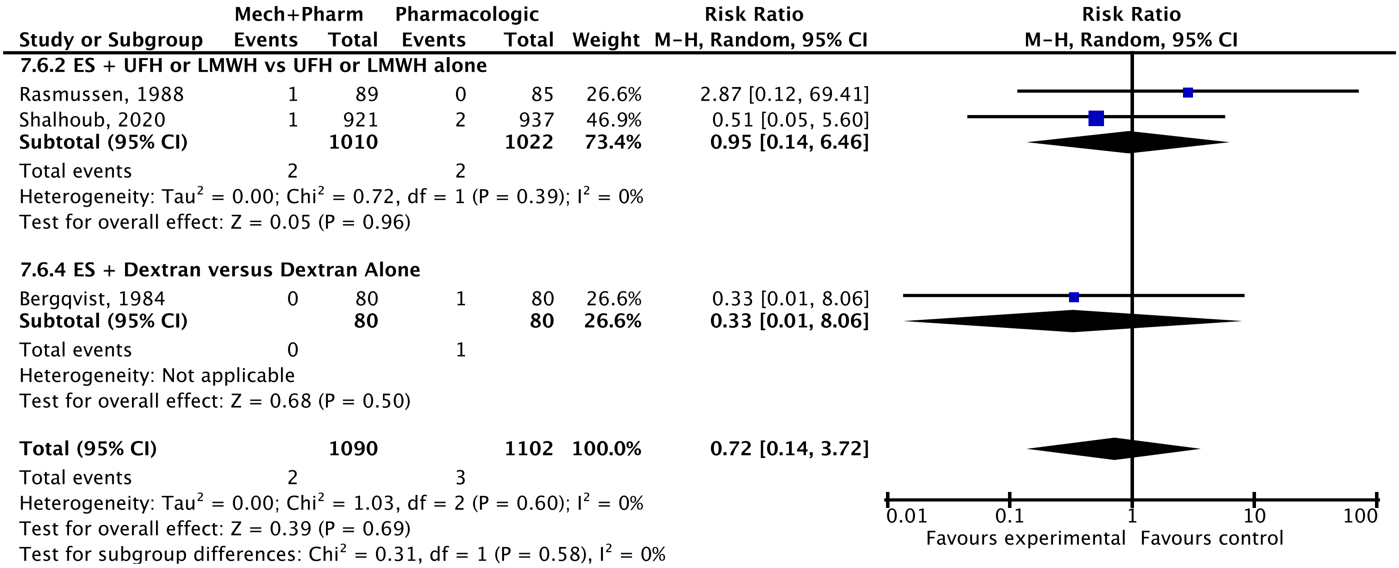

17. Any mechanical plus any pharmacological versus any pharmacological: pulmonary embolism

Study or Subgroup	Mech+Pharm Events	Mech+Pharm Total	Pharmacologic Events	Pharmacologic Total	Weight	Risk Ratio M-H, Random, 95% CI
**7.7.2 ES + UFH or LMWH vs UFH or LMWH alone**						
Rasmussen, 1988	0	89	0	85		Not estimable
Shalhoub, 2020	1	921	2	937	3.5%	0.51 [0.05, 5.60]
Wille-Jorgensen, 1985	2	86	6	90	8.1%	0.35 [0.07, 1.68]
**Subtotal (95% CI)**		**1096**		**1112**	**11.6%**	**0.39 [0.10, 1.46]**
Total events	3		8			
Heterogeneity: $Tau^2 = 0.00$; $Chi^2 = 0.07$, df = 1 (P = 0.80); $I^2 = 0\%$						
Test for overall effect: Z = 1.40 (P = 0.16)						
**7.7.3 IPC + UFH or LMWH vs. UFH or LMWH alone**						
Ramos, 1996	21	1355	48	1196	78.2%	0.39 [0.23, 0.64]
**Subtotal (95% CI)**		**1355**		**1196**	**78.2%**	**0.39 [0.23, 0.64]**
Total events	21		48			
Heterogeneity: Not applicable						
Test for overall effect: Z = 3.68 (P = 0.0002)						
**7.7.5 IPC + Dextran versus Dextran Alone**						
Smith, 1978	3	97	5	97	10.2%	0.60 [0.15, 2.44]
**Subtotal (95% CI)**		**97**		**97**	**10.2%**	**0.60 [0.15, 2.44]**
Total events	3		5			
Heterogeneity: Not applicable						
Test for overall effect: Z = 0.71 (P = 0.48)						
**Total (95% CI)**		**2548**		**2405**	**100.0%**	**0.40 [0.26, 0.63]**
Total events	27		61			
Heterogeneity: $Tau^2 = 0.00$; $Chi^2 = 0.40$, df = 3 (P = 0.94); $I^2 = 0\%$						
Test for overall effect: Z = 3.96 (P < 0.0001)						
Test for subgroup differences: $Chi^2 = 0.34$, df = 2 (P = 0.84), $I^2 = 0\%$						

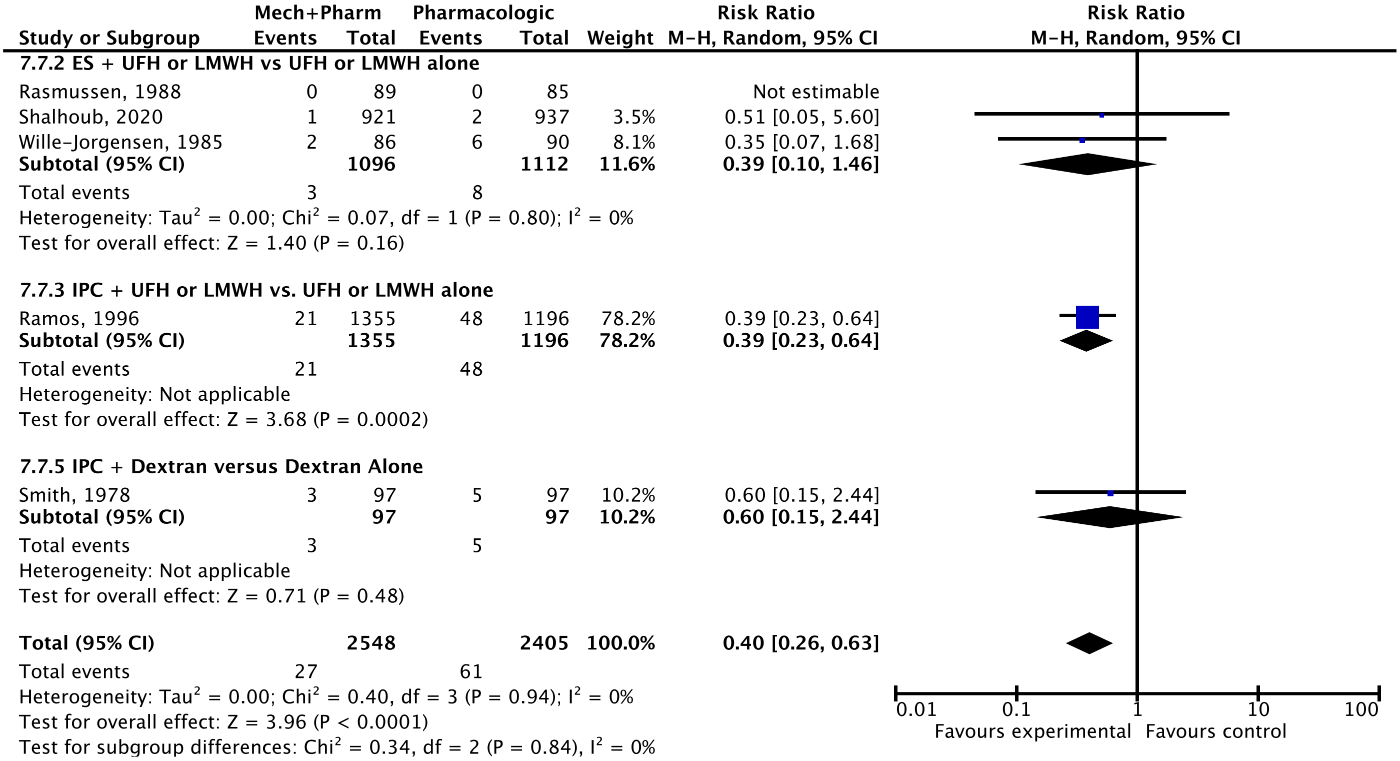

18. Any mechanical plus any pharmacological versus any pharmacological: any venous thromboembolism

Study or Subgroup	Mech+Pharm Events	Mech+Pharm Total	Pharmacologic Events	Pharmacologic Total	Weight	Risk Ratio M-H, Random, 95% CI
**7.8.2 ES + UFH or LMWH vs UFH or LMWH alone**						
Kalodiki, 1996	8	32	12	32	11.0%	0.67 [0.32, 1.41]
Rasmussen, 1988	23	89	25	85	14.3%	0.88 [0.54, 1.42]
Shalhoub, 2020	13	921	16	937	11.2%	0.83 [0.40, 1.71]
Torngren, 1980	4	98	12	98	7.5%	0.33 [0.11, 1.00]
Wille-Jorgensen, 1985	2	86	11	90	5.1%	0.19 [0.04, 0.83]
Wille-Jorgensen, 1991	2	79	12	81	5.2%	0.17 [0.04, 0.74]
**Subtotal (95% CI)**		**1305**		**1323**	**54.3%**	**0.55 [0.33, 0.90]**
Total events	52		88			
Heterogeneity: Tau^2 = 0.17; Chi^2 = 9.64, df = 5 (P = 0.09); I^2 = 48%						
Test for overall effect: Z = 2.38 (P = 0.02)						
**7.8.3 IPC + UFH or LMWH vs. UFH or LMWH alone**						
Arabi, 2019	103	991	95	1012	16.8%	1.11 [0.85, 1.44]
Ramos, 1996	21	1355	48	1196	14.0%	0.39 [0.23, 0.64]
**Subtotal (95% CI)**		**2346**		**2208**	**30.7%**	**0.67 [0.24, 1.88]**
Total events	124		143			
Heterogeneity: Tau^2 = 0.52; Chi^2 = 13.12, df = 1 (P = 0.0003); I^2 = 92%						
Test for overall effect: Z = 0.76 (P = 0.45)						
**7.8.4 ES + Dextran versus Dextran Alone**						
Bergqvist, 1984	0	80	9	80	1.8%	0.05 [0.00, 0.89]
**Subtotal (95% CI)**		**80**		**80**	**1.8%**	**0.05 [0.00, 0.89]**
Total events	0		9			
Heterogeneity: Not applicable						
Test for overall effect: Z = 2.04 (P = 0.04)						
**7.8.5 IPC + Dextran versus Dextran Alone**						
Smith, 1978	18	97	21	97	13.2%	0.86 [0.49, 1.51]
**Subtotal (95% CI)**		**97**		**97**	**13.2%**	**0.86 [0.49, 1.51]**
Total events	18		21			
Heterogeneity: Not applicable						
Test for overall effect: Z = 0.54 (P = 0.59)						
**Total (95% CI)**		**3828**		**3708**	**100.0%**	**0.59 [0.40, 0.87]**
Total events	194		261			
Heterogeneity: Tau^2 = 0.22; Chi^2 = 28.72, df = 9 (P = 0.0007); I^2 = 69%						
Test for overall effect: Z = 2.64 (P = 0.008)						
Test for subgroup differences: Chi^2 = 4.44, df = 3 (P = 0.22), I^2 = 32.4%						

Risk Ratio
M-H, Random, 95% CI

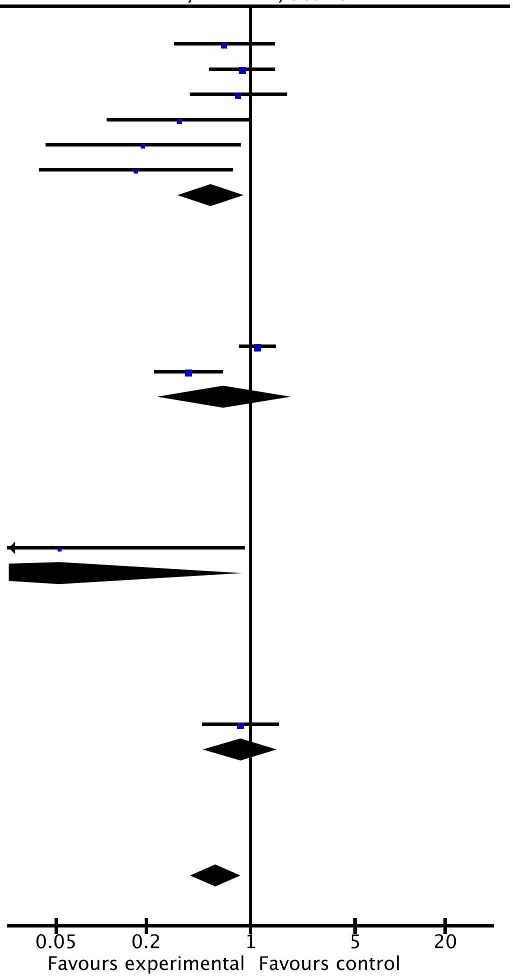

19. Aspirin versus placebo: symptomatic VTE

Study	Treatment Yes	Treatment No	Control Yes	Control No	Risk Ratio with 95% CI	Weight (%)
PEP-trial, hip fracture-group	87	6,592	122	6,555	0.71 [0.54, 0.94]	56.39
PEP-trial, arthroplasty-group	22	2,025	28	2,013	0.78 [0.45, 1.36]	14.71
POISE-2-trial	45	4,953	53	4,959	0.85 [0.57, 1.26]	28.30
STRATAGEM-trial	1	144	1	145	1.01 [0.06, 15.94]	0.61
**Overall**					0.76 [0.61, 0.94]	

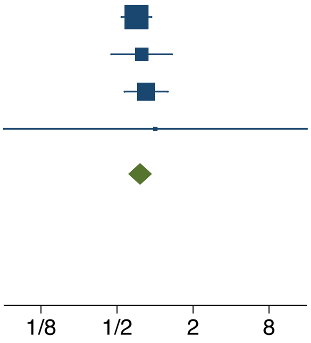

Heterogeneity: $\tau^2 = 0.00$, $I^2 = 3.37\%$, $H^2 = 1.03$

Test of $\theta_i = \theta_j$: $Q(3) = 0.58$, $p = 0.90$

Test of $\theta = 0$: $z = -2.47$, $p = 0.01$

Random-effects Sidik-Jonkman model

10. Search histories

1. Search history for baseline risk of VTE and Major Bleeding

Database: OVID Medline Epub Ahead of Print, In-Process & Other Non-Indexed Citations, Ovid MEDLINE(R) Daily and Ovid MEDLINE(R) 1946 March 15, 2019
Search Strategy:

((chemoprophylax* or chemoprophylactic* or prophylax* or prophylactic*) and (venous or vein or thromb*)).ti,ab.
(prevent* adj3 (venous or vein or thromb*)).mp.
(thromboprophylax* or thromboprophylactic*).mp.
*Postoperative Complications/
Postoperative Complications/ep, et, pc
Risk Factors/
(ep or ae).fs. and (venous or thromb* or bleed* or haemorr* or hemorr*).ti,ab.
(risk* or high-risk or incidence* or meta?analysis or analysis or complication* or outcome* or safety or versus or thrombosis or transfusion* or adverse or bleed* or haemorr* or hemorr*).ti.
or/1-8
embolism/ or exp pulmonary embolism/ or exp thromboembolism/
exp Thrombosis/
(DVT or VTE or PE).ti,ab.
((venous or vein or pulmonary or lung) adj3 (emboli* or thromb*)).mp.
(DVT or VTE or PE or PTE).ti,ab.
or/10-14
9 and 15
Appendectomy/ or exp Bariatric Surgery/ or exp Cholecystectomy/ or exp Colectomy/ or exp Gastrectomy/ or Hepatectomy/ or Herniorrhaphy/ or pancreatectomy/ or Pancreaticoduodenectomy/ or pancreaticojejunostomy/ or Splenectomy/
General Surgery/ or exp digestive system surgical procedures/
exp Digestive System/su or Cholecystitis/su or Gallbladder/su or exp Gallbladder Diseases/su or Hernia, Abdominal/su or Hernia, Inguinal/su or exp Hernia, Ventral/su or exp Intestinal Diseases/su or exp Liver Diseases/su or exp Pancreas/su or exp Pancreatic Diseases/su or Spleen/su or exp Splenic Diseases/su or exp Stomach Diseases/su
(appendectom* or appendicectom* or colectomy* or proctocolectom* or cholecystectom* or duodenectom* or gastrectom* or hernioplast* or herniorrhaph* or herniotom* or jejunectom* or pancreatectom* or pancreaticojejunostom* or pancreaticoduodenectom* or duodenopancreatectom*).mp.
((surgery or resection* or excision* or repair* or operation* or laproscop* or laparoscop* or sleeve*) adj3 (abdominoperineal or perineal or anal* or anus or appendix or bowel* or colon* or duoden* or jejun* or ileal* or ileum* or jejuno?ileal or intestine* or gall bladder or gall?bladder or gastric or bariatric* or stomach or hernia or liver or adenoma or hepatoma* or hepatocellular* or rectal* or rectum)).mp.
((general or abdominal or major) adj3 (surgery or surgical)).mp.
(prolapse adj3 rectal).mp.
(Rectopexy or rectosigmoidectom* or sigmoidectom* or DHoore or d'hoore or Delorme or Altemeier).mp.
or/17-24
16 and 25
9 and 15 and 25

EMBASE

Database: Embase <1974 to 2019 March 11>
Search Strategy:

((chemoprophylax* or chemoprophylactic* or prophylax* or prophylactic*) and (venous or vein or thromb*)).ti,ab. (28841)
(prevent* adj3 (venous or vein or thromb*)).mp. (28252)
(thromboprophylax* or thromboprophylactic*).mp. (7355)
*postoperative complication/co, ep, et, pc [Complication, Epidemiology, Etiology, Prevention] (33531)
exp *venous thromboembolism/co, ep, et, pc [Complication, Epidemiology, Etiology, Prevention] (18251)
thrombosis prevention/ (10458)
postoperative complication/ep [Epidemiology] (9157)
*venous thromboembolism/ (14535)
*deep vein thrombosis/ (15764)
venous thromboembolism/ep [Epidemiology] (1367)
risk factor/ (925628)
(ep or co).fs. and (venous or thromb* or bleed* or haemorr* or hemorr*).ti,ab.

(220502)
(risk* or high-risk or incidence* or meta?analysis or analysis or complication* or outcome* or safety or versus or thrombosis or transfusion* or adverse or bleed* or haemorr* or hemorr*).ti. (2707778)
or/1-13 (3486260)
Annotation: post op VTE comp
exp thromboembolism/ (440725)
(DVT or VTE or PE or PTE).ti,ab. (82850)
((venous or vein or pulmonary or lung) adj3 (emboli* or thromb*)).mp. (216179)
or/15-17 (506092)
Annotation: VTE broad
14 and 18 (224773)
Annotation: risk of post-op VTE
general surgery/ (13528)
exp abdominal surgery/ (708663)
exp gastrointestinal surgery/ (321622)
cholecystitis/su [Surgery] (3221)
gallbladder disease/su [Surgery] (1698)
exp abdominal wall hernia/su [Surgery] (15449)
exp enteropathy/su [Surgery] (124538)
exp enteropathy/su [Surgery] (124538)
exp liver disease/su [Surgery] (61513)
exp pancreas disease/su [Surgery] (33926)
exp spleen disease/su [Surgery] (6989)
exp stomach disease/su [Surgery] (42619)
((general or abdominal or major) adj3 (surgery or surgical)).mp. (102145)
(prolapse adj3 rectal).mp. [mp=title, abstract, heading word, drug trade name, original title, device manufacturer, drug manufacturer, device trade name, keyword, floating subheading word, candidate term word] (3266)
(rectopexy or proctopexy or rectosigmoidectom* or sigmoidectom* or DHoore or d'hoore or Delorme or Altemeier).mp. (5621)
(surgery or resection* or excision* or repair* or operation* or laproscop* or sleeve*).mp. and (exp digestive system/ or exp spleen/) [mp=title, abstract, heading word, drug trade name, original title, device manufacturer, drug manufacturer, device trade name, keyword, floating subheading word, candidate term word] (233115)
(appendectom* or appendicectom* or colectomy* or proctocolectom* or cholecystectom* or duodenectom* or gastrectom* or hernioplast* or herniorrhaph* or herniotom* or jejunectom* or pancreatectom* or pancreaticojejunostom* or pancreaticoduodenectom* or duodenopancreatectom*).mp. (189962)
((surgery or resection* or excision* or repair* or operation* or laproscop* or laparoscop* or sleeve*) adj3 (abdominoperineal or anal* or anus or appendix or bowel* or colon* or duoden* or jejun* or ileal* or ileum* or jejuno?ileal or intestine* or gall bladder or gall?bladder or gastric or bariatric* or stomach or hernia or liver or adenoma or hepatoma* or hepatocellular* or rectal* or rectum)).mp. (273066)
or/20-37 (1022761)
14 and 18 and 38 (22794)
exp animals/ or exp invertebrate/ or animal experiment/ or animal model/ or

animal tissue/ or animal cell/ or nonhuman/ (25472734)

human/ or normal human/ or human cell/ (19402712)
40 and 41 (19349027)
40 not 42 (6123707)
39 not 43 (22544)
exp controlled clinical trial/ (718277)
44 not 45 (21607)
clinical study/ (151683)
case control study/ (136785)
family study/ (25001)
longitudinal study/ (121983)
retrospective study/ (740307)
prospective study/ (500661)
cohort analysis/ (442572)
(Cohort adj (study or studies)).mp. (251414)
(Case control adj (study or studies)).tw. (119867)
(follow up adj (study or studies)).tw. (58795)
(observational adj (study or studies)).tw. (139036)
(epidemiologic$ adj (study or studies)).tw. (98776)
(cross sectional adj (study or studies)).tw. (180909)
or/47-59 (2283762)
46 and 60 (5348)
(prognosis or prognostic or predict* or risk*).mp. [mp=title, abstract, heading word, drug trade name, original title, device manufacturer, drug manufacturer, device trade name, keyword, floating subheading word, candidate term word] (5487179)
(incidence* or outcome* or comparison* or complication*).ti. (1083287)
prevalence.mp. or prevalence/ (939978)
baseline.mp. (829438)
or/62-65 (7111926)
46 and 66 (14542)
61 or 67 (15593)
transplant*.ti,kw,jw. (479040)
transplant*.ab. /freq=2 (315144)
69 or 70 (555136)
68 not 71 (12241)
→73 remove 209 duplicates in Endnote (12032)
→74 limit 73 to year =>2004 in Endnote (10467)

Web of Science

→#20 **7304** **remove** duplicates in endnote
19 7,323 #17 NOT #18
Indexes=SCI-EXPANDED, SSCI, A&HCI, CPCI-S, CPCI-SSH, ESCI Timespan=2004-2019
18 410,495 TS=transplant*
Indexes=SCI-EXPANDED, SSCI, A&HCI, CPCI-S, CPCI-SSH, ESCI Timespan=2004-2019
17 8,098 #16
Indexes=SCI-EXPANDED, SSCI, A&HCI, CPCI-S, CPCI-SSH, ESCI Timespan=2004-2019
16 8,098 #15 AND #14
Indexes=SCI-EXPANDED, SSCI, A&HCI, CPCI-S, CPCI-SSH, ESCI Timespan=2004-2019
15 11,374,071 TS=(cohort or observational or cross-sectional or longitudinal NEAR/2 study or studies)
Indexes=SCI-EXPANDED, SSCI, A&HCI, CPCI-S, CPCI-SSH, ESCI Timespan=2004-2019
14 20,183 #13 AND #8
Indexes=SCI-EXPANDED, SSCI, A&HCI, CPCI-S, CPCI-SSH, ESCI Timespan=2004-2019
13 1,171,802 #12 OR #11 OR #10 OR #9
Indexes=SCI-EXPANDED, SSCI, A&HCI, CPCI-S, CPCI-SSH, ESCI Timespan=2004-2019
12 875,276 TI=(complication* or outcome* or safety or versus or thrombosis or transfusion* or adverse or bleed* or haemorr* or hemorr*)
Indexes=SCI-EXPANDED, SSCI, A&HCI, CPCI-S, CPCI-SSH, ESCI Timespan=2004-2019
11 4,861 TS=(thromboprophylax* or thromboprophylactic*)
Indexes=SCI-EXPANDED, SSCI, A&HCI, CPCI-S, CPCI-SSH, ESCI Timespan=2004-2019
10 358,947 TS=(prevent* NEAR/3 venous or vein or thromb*)
Indexes=SCI-EXPANDED, SSCI, A&HCI, CPCI-S, CPCI-SSH, ESCI Timespan=2004-2019
9 4,145 TS=((chemoprophylax* or chemoprophylactic*or prophylax* or prophylactic*) and (venous or vein or thromb*))
Indexes=SCI-EXPANDED, SSCI, A&HCI, CPCI-S, CPCI-SSH, ESCI Timespan=2004-2019
8 31,477 #7 AND #4
Indexes=SCI-EXPANDED, SSCI, A&HCI, CPCI-S, CPCI-SSH, ESCI Timespan=2004-2019
7 282,903 #6 OR #5
Indexes=SCI-EXPANDED, SSCI, A&HCI, CPCI-S, CPCI-SSH, ESCI Timespan=2004-2019
6 271,002 TI=(venous or vein or pulmonary or lung NEAR/3 emboli* or thromb*)
Indexes=SCI-EXPANDED, SSCI, A&HCI, CPCI-S, CPCI-SSH, ESCI Timespan=2004-2019
5 22,655 TS=(DVT or VTE or PE or PTE)
Indexes=SCI-EXPANDED, SSCI, A&HCI, CPCI-S, CPCI-SSH, ESCI Timespan=2004-2019
4 2,747,833 #3 OR #2 OR #1
Indexes=SCI-EXPANDED, SSCI, A&HCI, CPCI-S, CPCI-SSH, ESCI Timespan=2004-2019
3 25,450 TI=(appendectom* or appendicectom* or colectomy* or proctocolectom* or cholecystectom* or duodenectom* or gastrectom* or hernioplast* or herniorrhaph* or herniotom* or jejunectom* or pancreatecom* or pancreaticojejunostom* or pancreaticoduodenectom* or duodenopancreatectom* or rectopexy or rectosigmoidectom* or sigmoidectom* or DHoore or d'hoore or Delorme or Altemeier)
Indexes=SCI-EXPANDED, SSCI, A&HCI, CPCI-S, CPCI-SSH, ESCI Timespan=2004-2019
2 2,541,613 TI=(surgery or resection* or excision* or repair* or operation* or prolapse* or laproscop* or laparoscop* or sleeve* NEAR/3 abdominoperineal or anal* or anus or appendix or bowel* or colon* or duoden* or jejun* ileal* or ileum* or jejuno?ileal or intestine* or gall bladder or gall?bladder or gastric or bariatric* or stomach or hernia or liver or adenoma or hepatoma* or hepatocellular* or rectal* or rectum)
Indexes=SCI-EXPANDED, SSCI, A&HCI, CPCI-S, CPCI-SSH, ESCI Timespan=2004-2019
1 248,183 TI= (general or abdominal or major NEAR/3 surgery or surgical)
Indexes=SCI-EXPANDED, SSCI, A&HCI, CPCI-S, CPCI-SSH, ESCI Timespan=2004-2019

Google Scholar

We queried Google scholar using Harzig's PublishorPerish version 6.49.6406
https://harzing.com/resources/publish-or-perish
We ran two queries (details below) and selected the highest-ranked records for each query (rank=>750) and combined the results in Endnote. NB there was a database error in Q2 and only 980 records were downloaded (instead of 1000)

Query 1
risk, embolism thrombosis DVT VTE PE PTE, general surgery
Publish or Perish 6.49.6406.7079
Search terms
All of the words: risk
Any of the words: embolism thrombosis DVT VTE PE PTE
The phrase: general surgery
Years: all
Data retrieval
Data source: Google Scholar
Query date: 28/05/2019 11:21:52 AM
Cache date: 28/05/2019 11:44:03 AM
Query result: [0] The operation completed successfully.

Metrics
Reference date: 28/05/2019 11:21:52 AM
Publication years: 1945-2018
Citation years: 74 (1945-2019)
Papers: 999
Citations: 118931
Citations/year: 1607.18
Citations/paper: 119.05 (*count=188)
Citations/author: 39958.01
Papers/author: 381.79
Authors/paper: 3.46/4.0/4 (mean/median/mode)
Age-weighed citation rate: 8904.22 (sqrt=94.36), 2856.27/author
Hirsch h-index: 158 (a=4.76, m=2.14, 80933 cites=68.1% coverage)
Egghe g-index: 311 (g/h=1.97, 97225 cites=81.7% coverage)
PoP hI,norm: 92
PoP hI,annual: 1.24

Google Scholar

Query 2
thromboembolism incidence, surgery resection excision operation
Publish or Perish 6.49.6406.7079
Search terms
All of the words: thromboembolism incidence
Any of the words: surgery resection excision operation
Years: all

Data retrieval
Data source: Google Scholar
Query date: 28/05/2019 2:01:25 PM
Cache date: 28/05/2019 2:23:46 PM
Query result: [12152] The server returned an invalid or unrecognized response

Metrics
Reference date: 28/05/2019 2:01:25 PM
Publication years: 1947-2018
Citation years: 72 (1947-2019)
Papers: 980
Citations: 125726
Citations/year: 1746.19
Citations/paper: 128.29 (*count=217)
Citations/author: 40777.78
Papers/author: 331.91
Authors/paper: 3.71/4.0/4 (mean/median/mode)
Age-weighed citation rate: 8931.95 (sqrt=94.51), 2815.63/author
Hirsch h-index: 171 (a=4.30, m=2.38, 82333 cites=65.5% coverage)
Egghe g-index: 317 (g/h=1.85, 100935 cites=80.3% coverage)
PoP hI,norm: 94
PoP hI,annual: 1.31

2. Search history update searches for baseline risk of VTE and Major Bleeding

MEDLINE

Database: OVID Medline Epub Ahead of Print, In-Process & Other Non-Indexed Citations, Ovid MEDLINE(R) Daily and Ovid MEDLINE(R) 1946 to October 27, 2020

Search Strategy:

----

1 ((chemoprophylax* or chemoprophylactic* or prophylax* or prophylactic*) and (venous or vein or thromb*)).ti,ab. (18812)
2 (prevent* adj3 (venous or vein or thromb*)).mp. (15559)
3 (thromboprophylax* or thromboprophylactic*).mp. [mp=title, abstract, original title, name of substance word, subject heading word, floating sub-heading word, keyword heading word, organism supplementary concept word, protocol supplementary concept word, rare disease supplementary concept word, unique identifier, synonyms] (5164)
4 *Postoperative Complications/ (161247)
5 Postoperative Complications/ep, et, pc [Epidemiology, Etiology, Prevention & Control] (146329)
6 Risk Factors/ (837286)
7 (ep or ae).fs. and (venous or thromb* or bleed* or haemorr* or hemorr*).ti,ab. (209217)
8 (risk* or high-risk or incidence* or meta?analysis or analysis or complication* or outcome* or safety or versus or thrombosis or transfusion* or adverse or bleed* or haemorr* or hemorr*).ti. (2394352)
9 or/1-8 (3165211)
10 embolism/ or exp pulmonary embolism/ or exp thromboembolism/ (103380)
11 exp Thrombosis/ (130421)
12 (DVT or VTE or PE).ti,ab. (63995)
13 ((venous or vein or pulmonary or lung) adj3 (emboli* or thromb*)).mp. [mp=title, abstract, original title, name of substance word, subject heading word, floating sub-heading word, keyword heading word, organism supplementary concept word, protocol supplementary concept word, rare disease supplementary concept word, unique identifier, synonyms] (128907)
14 (DVT or VTE or PE or PTE).ti,ab. (66040)
15 or/10-14 (295147)
Annotation: VTE block
16 9 and 15 (131905)
Annotation: post op comps AND VTE
17 Appendectomy/ or exp Bariatric Surgery/ or exp Cholecystectomy/ or exp Colectomy/ or exp Gastrectomy/ or Hepatectomy/ or Herniorrhaphy/ or pancreatectomy/ or Pancreaticoduodenectomy/ or pancreaticojejunostomy/ or Splenectomy/ (194233)
18 General Surgery/ or exp digestive system surgical procedures/ (407450)
19 exp Digestive System/su or Cholecystitis/su or Gallbladder/su or exp Gallbladder Diseases/su or Hernia, Abdominal/su or Hernia, Inguinal/su or exp Hernia, Ventral/su or exp Intestinal Diseases/su or exp Liver Diseases/su or exp Pancreas/su or exp Pancreatic Diseases/su or Spleen/su or exp Splenic Diseases/su or exp Stomach Diseases/su (334048)
20 (appendectom* or appendicectom* or colectomy* or proctocolectom* or cholecystectom* or duodenectom* or gastrectom* or hernioplast* or herniorrhaph* or herniotom* or jejunectom* or pancreatectom* or pancreaticojejunostom* or pancreaticoduodenectom* or duodenopancreatectom*).mp. [mp=title, abstract, original title, name of substance word, subject heading word, floating sub-heading word, keyword heading word, organism supplementary concept word, protocol supplementary concept word, rare disease supplementary concept word, unique identifier, synonyms] (162568)
21 ((surgery or resection* or excision* or repair* or operation* or laproscop* or laparoscop* or sleeve*) adj3 (abdominoperineal or perineal or anal* or anus or appendix or bowel* or colon* or duoden* or jejun* or ileal* or ileum* or jejuno?ileal or intestine* or gall bladder or gall?bladder or gastric or bariatric* or stomach or hernia or liver or adenoma or hepatoma* or hepatocellular* or rectal* or rectum)).mp. [mp=title, abstract, original title, name of substance word, subject heading word, floating sub-heading word, keyword heading word, organism supplementary concept word, protocol supplementary concept word, rare disease supplementary concept word, unique identifier, synonyms] (154351)
22 ((general or abdominal or major) adj3 (surgery or surgical)).mp. (100037)
23 (prolapse adj3 rectal).mp. [mp=title, abstract, original title, name of substance word, subject heading word, floating sub-heading word, keyword heading word, organism supplementary concept word, protocol supplementary concept word, rare disease supplementary concept word, unique identifier, synonyms] (3729)
24 (Rectopexy or rectosigmoidectom* or sigmoidectom* or DHoore or d'hoore or Delorme or Altemeier).mp. (2266)
25 or/17-24 (754910)
26 16 and 25 (9517)
27 9 and 15 and 25 (9517)
28 limit 27 to ed=20190301-20201027 (749)
29 limit 27 to yr="2019 -Current" (779)
30 28 or 29 (978)
→31 search for transplant* in title or keyword field in Endnote (150)
→32 30 NOT 31 in Endnote (828)

EMBASE
Database: Embase <1974 to 2020 October 26>
Search Strategy:
----
1 ((chemoprophylax* or chemoprophylactic* or prophylax* or prophylactic*) and (venous or vein or thromb*)).ti,ab. (32192)
2 (prevent* adj3 (venous or vein or thromb*)).mp. (31383)
3 (thromboprophylax* or thromboprophylactic*).mp. (8518)
4 *postoperative complication/co, ep, et, pc [Complication, Epidemiology, Etiology, Prevention] (38244)
5 exp *venous thromboembolism/co, ep, et, pc [Complication, Epidemiology, Etiology, Prevention] (19508)
6 thrombosis prevention/ (11876)
7 postoperative complication/ep [Epidemiology] (10491)
8 *venous thromboembolism/ (16821)
9 *deep vein thrombosis/ (17253)
10 venous thromboembolism/ep [Epidemiology] (1520)
11 risk factor/ (1068091)
12 (ep or co).fs. and (venous or thromb* or bleed* or haemorr* or hemorr*).ti,ab. (234412)
13 (risk* or high-risk or incidence* or meta?analysis or analysis or complication* or outcome* or safety or versus or thrombosis or transfusion* or adverse or bleed* or haemorr* or hemorr*).ti. (3136902)
14 or/1-13 (4013485)
Annotation: post op VTE comp
15 exp thromboembolism/ (490180)
16 (DVT or VTE or PE or PTE).ti,ab. (97278)
17 ((venous or vein or pulmonary or lung) adj3 (emboli* or thromb*)).mp. (243933)
18 or/15-17 (565928)
Annotation: VTE broad
19 14 and 18 (252700)
Annotation: risk of post-op VTE
20 general surgery/ (16045)
21 exp abdominal surgery/ (795673)
22 exp gastrointestinal surgery/ (366003)
23 cholecystitis/su [Surgery] (3367)
24 gallbladder disease/su [Surgery] (1815)
25 exp abdominal wall hernia/su [Surgery] (16913)
26 exp enteropathy/su [Surgery] (135296)
27 exp enteropathy/su [Surgery] (135296)
28 exp liver disease/su [Surgery] (66737)
29 exp pancreas disease/su [Surgery] (37125)
30 exp spleen disease/su [Surgery] (7420)
31 exp stomach disease/su [Surgery] (46514)
32 ((general or abdominal or major) adj3 (surgery or surgical)).mp. (115366)
33 (prolapse adj3 rectal).mp. [mp=title, abstract, heading word, drug trade name, original title, device manufacturer, drug manufacturer, device trade name, keyword, floating subheading word, candidate term word] (3629)
34 (rectopexy or proctopexy or rectosigmoidectom* or sigmoidectom* or DHoore or d'hoore or Delorme or Altemeier).mp. (6344)
35 (surgery or resection* or excision* or repair* or operation* or laproscop* or sleeve*).mp. and (exp digestive system/ or exp spleen/) [mp=title, abstract, heading word, drug trade name, original title, device manufacturer, drug manufacturer, device trade name, keyword, floating subheading word, candidate term word] (258623)
36 (appendectom* or appendicectom* or colectomy* or proctocolectom* or cholecystectom* or duodenectom* or gastrectom* or hernioplast* or herniorrhaph* or herniotom* or jejunectom* or pancreatectom* or pancreaticojejunostom* or pancreaticoduodenectom* or duodenopancreatectom*).mp. (217703)
37 ((surgery or resection* or excision* or repair* or operation* or laproscop* or laparoscop* or sleeve*) adj3 (abdominoperineal or anal* or anus or appendix or bowel* or colon* or duoden* or jejun* or ileal* or ileum* or jejuno?ileal or intestine* or gall bladder or gall?bladder or gastric or bariatric* or stomach or hernia or liver or adenoma or hepatoma* or hepatocellular* or rectal* or rectum)).mp. (312571)
38 or/20-37 (1141588)
39 14 and 18 and 38 (26028)
40 exp animals/ or exp invertebrate/ or animal experiment/ or animal model/ or animal tissue/ or animal cell/ or nonhuman/ (28182878)
41 human/ or normal human/ or human cell/ (21665531)
42 40 and 41 (21599953)
43 40 not 42 (6582925)
44 39 not 43 (25755)

45 exp controlled clinical trial/ (817708)
46 44 not 45 (24679)
47 clinical study/ (156238)
48 case control study/ (163048)
49 family study/ (26140)
50 longitudinal study/ (146898)
51 retrospective study/ (984448)
52 prospective study/ (638840)
53 cohort analysis/ (631612)
54 (Cohort adj (study or studies)).mp. (322816)
55 (Case control adj (study or studies)).tw. (137939)
56 (follow up adj (study or studies)).tw. (64541)
57 (observational adj (study or studies)).tw. (175801)
58 (epidemiologic$ adj (study or studies)).tw. (108420)
59 (cross sectional adj (study or studies)).tw. (231148)
60 or/47-59 (2849250)
61 46 and 60 (6858)
62 (prognosis or prognostic or predict* or risk*).mp. [mp=title, abstract, heading word, drug trade name, original title, device manufacturer, drug manufacturer, device trade name, keyword, floating subheading word, candidate term word] (6352775)
63 (incidence* or outcome* or comparison* or complication*).ti. (1244456)
64 prevalence.mp. or prevalence/ (1092785)
65 baseline.mp. (980378)
66 or/62-65 (8208047)
67 46 and 66 (16930)
68 61 or 67 (18189)
69 transplant*.ti,kw,jw. (528976)
70 transplant*.ab. /freq=2 (353485)
71 69 or 70 (614781)
72 68 not 71 (14352)
73 limit 72 to em=201911-202052 (1333)
74 limit 72 to yr="2019 -Current" (2118)
75 73 or 74 (2274)

Web of Science

# 19	**1,917**	#18<br>*Indexes=SCI-EXPANDED, SSCI, A&HCI, CPCI-S, CPCI-SSH, BKCI-S, BKCI-SSH, ESCI, CCR-EXPANDED, IC Timespan=2019-2020*
# 18	**11,210**	#16 not #17<br>*Indexes=SCI-EXPANDED, SSCI, A&HCI, CPCI-S, CPCI-SSH, BKCI-S, BKCI-SSH, ESCI, CCR-EXPANDED, IC Timespan=All years*
# 17	**668,584**	TS=transplant*<br>*Indexes=SCI-EXPANDED, SSCI, A&HCI, CPCI-S, CPCI-SSH, BKCI-S, BKCI-SSH, ESCI, CCR-EXPANDED, IC Timespan=All years*
# 16	**12,310**	#15 AND #14<br>*Indexes=SCI-EXPANDED, SSCI, A&HCI, CPCI-S, CPCI-SSH, BKCI-S, BKCI-SSH, ESCI, CCR-EXPANDED, IC Timespan=All years*
# 15	**18,007,315**	TS=(cohort or observational or cross-sectional or longitudinal NEAR/2 study or studies)<br>*Indexes=SCI-EXPANDED, SSCI, A&HCI, CPCI-S, CPCI-SSH, BKCI-S, BKCI-SSH, ESCI, CCR-EXPANDED, IC Timespan=All years*
# 14	**34,017**	#13 AND #8<br>*Indexes=SCI-EXPANDED, SSCI, A&HCI, CPCI-S, CPCI-SSH, BKCI-S, BKCI-SSH, ESCI, CCR-EXPANDED, IC Timespan=All years*
# 13	**1,948,488**	#12 OR #11 OR #10 OR #9<br>*Indexes=SCI-EXPANDED, SSCI, A&HCI, CPCI-S, CPCI-SSH, BKCI-S, BKCI-SSH, ESCI, CCR-EXPANDED, IC Timespan=All years*
# 12	**1,417,082**	TI=(complication* or outcome* or safety or versus or thrombosis or transfusion* or adverse or bleed* or haemorr* or hemorr*)<br>*Indexes=SCI-EXPANDED, SSCI, A&HCI, CPCI-S, CPCI-SSH, BKCI-S, BKCI-SSH, ESCI, CCR-EXPANDED, IC Timespan=All years*
# 11	**6,802**	TS=(thromboprophylax* or thromboprophylactic*)<br>*Indexes=SCI-EXPANDED, SSCI, A&HCI, CPCI-S, CPCI-SSH, BKCI-S, BKCI-SSH, ESCI, CCR-EXPANDED, IC Timespan=All years*
# 10	**636,253**	TS=(prevent* NEAR/3 venous or vein or thromb*)<br>*Indexes=SCI-EXPANDED, SSCI, A&HCI, CPCI-S, CPCI-SSH, BKCI-S, BKCI-SSH, ESCI, CCR-EXPANDED, IC Timespan=All years*
# 9	**6,619**	TS=((chemoprophylax* or chemoprophylactic*or prophylax* or prophylactic*) and (venous or vein or thromb*))<br>*Indexes=SCI-EXPANDED, SSCI, A&HCI, CPCI-S, CPCI-SSH, BKCI-S, BKCI-SSH, ESCI, CCR-EXPANDED, IC Timespan=All years*
# 8	**54,900**	#7 AND #4<br>*Indexes=SCI-EXPANDED, SSCI, A&HCI, CPCI-S, CPCI-SSH, BKCI-S, BKCI-SSH, ESCI, CCR-EXPANDED, IC Timespan=All years*
# 7	**548,889**	#6 OR #5<br>*Indexes=SCI-EXPANDED, SSCI, A&HCI, CPCI-S, CPCI-SSH, BKCI-S, BKCI-SSH, ESCI, CCR-EXPANDED, IC Timespan=All years*
# 6	**530,835**	TI=(venous or vein or pulmonary or lung NEAR/3 emboli* or thromb*)<br>*Indexes=SCI-EXPANDED, SSCI, A&HCI, CPCI-S, CPCI-SSH, BKCI-S, BKCI-SSH, ESCI, CCR-EXPANDED, IC Timespan=All years*
# 5	**33,498**	TS=(DVT or VTE or PE or PTE)<br>*Indexes=SCI-EXPANDED, SSCI, A&HCI, CPCI-S, CPCI-SSH, BKCI-S, BKCI-SSH, ESCI, CCR-EXPANDED, IC Timespan=All years*
# 4	**5,084,933**	#3 OR #2 OR #1<br>*Indexes=SCI-EXPANDED, SSCI, A&HCI, CPCI-S, CPCI-SSH, BKCI-S, BKCI-SSH, ESCI, CCR-EXPANDED, IC Timespan=All years*

# 3	**44,375**	TI=(appendectom* or appendicectom* or colectomy* or proctocolectom* or cholecystect om* or duodenectom* or gastrectom* or hernioplast* or herniorrhaph* or herniotom* or jejunectom* or pancreatecom* or pancreaticojejunostom* or pancreaticoduodenectom* or duodenopancreatectom* or rectopexy or rectosigmoidectom* or sigmoidectom* or D Hoore or d'hoore or Delorme or Altemeier)<br>*Indexes=SCI-EXPANDED, SSCI, A&HCI, CPCI-S, CPCI-SSH, BKCI-S, BKCI-SSH, ESCI, CCR-EXPANDED, IC Timespan=All years*
# 2	**4,670,456**	TI=(surgery or resection* or excision* or repair* or operation* or prolapse* or laproscop * or laparoscop* or sleeve* NEAR/3 abdominoperineal or anal* or anus or appendix or b owel* or colon* or duoden* or jejun* ileal* or ileum* or jejuno?ileal or intestine* or gall b ladder or gall?bladder or gastric or bariatric* or stomach or hernia or liver or adenoma o r hepatoma* or hepatocellular* or rectal* or rectum)<br>*Indexes=SCI-EXPANDED, SSCI, A&HCI, CPCI-S, CPCI-SSH, BKCI-S, BKCI-SSH, ESCI, CCR-EXPANDED, IC Timespan=All years*
# 1	**485,031**	TI= (general or abdominal or major NEAR/3 surgery or surgical)<br>*Indexes=SCI-EXPANDED, SSCI, A&HCI, CPCI-S, CPCI-SSH, BKCI-S, BKCI-SSH, ESCI, CCR-EXPANDED, IC Timespan=All years*

Google Scholar

Google Search update Aug 5, 2021
We queried Google Scholar using Harzig's PublishorPerish for macOSVersion: 7.33.3373 (28 May 2021)
https://harzing.com/resources/publish-or-perish/os-x
risk AND (embolism or thrombosis or DVT or VTE or PE or PTE) and "general surgery"
and years 2019-2020
yield =26

3. Search history for patient related risk factors of major bleeding/bleeding requiring reintervention after surgery

Database: Ovid MEDLINE(R) and Epub Ahead of Print, In-Process, In-Data-Review & Other Non-Indexed Citations and Daily <1946 to June 28, 2021>

Search Strategy:

----

1 exp Embolism/ (62107)
2 exp Thromboembolism/ (59004)
3 exp Venous Thrombosis/ (56480)
4 exp Thrombophlebitis/ (21854)
5 1 or 2 or 3 or 4 (157453)
6 exp Colorectal Surgery/ (3715)
7 exp General Surgery/ (39711)
8 exp Gynecology/ (19511)
9 exp Urology/ (12092)
10 6 or 7 or 8 or 9 (73691)
11 5 and 10 (313)
12 limit 11 to yr="2014 -Current" (57)
13 "32755462".an. (1)
14 "32496331".an. (1)
15 "25213583".an. (1)
16 hemorrhage/ or blood loss, surgical/ or exsanguination/ or hematocele/ or hematoma/ or hemoperitoneum/ or postoperative hemorrhage/ or shock, hemorrhagic/ (138221)
17 exp Colorectal Surgery/ (3715)
18 exp General Surgery/ (39711)
19 exp Gynecology/ (19511)
20 exp Urology/ (12092)
21 17 or 18 or 19 or 20 (73691)
22 16 and 21 (387)
23 limit 22 to yr="2000 -Current" (187)
24 16 and 21 (387)
25 limit 22 to yr="2014-Current" (82)

4. Search history for effects of pharmacological and mechanical thromboprophylaxis on VTE and bleeding

Database: Ovid MEDLINE(R) and Epub Ahead of Print, In-Process & Other Non-Indexed Citations and Daily <1946 to June 15, 2020>
Search Strategy:

----

1 exp Embolism/ (59798)
2 exp Thromboembolism/ (55955)
3 exp Venous Thrombosis/ (54628)
4 exp Thrombophlebitis/ (21765)
5 1 or 2 or 3 or 4 (151132)
6 exp Bariatric Surgery/ (25571)
7 exp Colorectal Surgery/ (3406)
8 exp General Surgery/ (38702)
9 exp Gynecology/ (18901)
10 exp Neurosurgery/ (14914)
11 exp Otolaryngology/ (13042)
12 exp Surgery, Plastic/ (26219)
13 exp Thoracic Surgery/ (12640)
14 exp Traumatology/ (3485)
15 exp Urology/ (11384)
16 exp "Wounds and Injuries"/ (900022)
17 exp Abdominal Injuries/ (20336)
18 exp Amputation, Traumatic/ (4747)
19 exp Arm Injuries/ (30844)
20 exp Asphyxia/ (6192)
21 exp Athletic Injuries/ (27237)
22 exp Back Injuries/ (24580)
23 exp Barotrauma/ (9096)
24 exp Burns/ (57428)
25 exp Craniocerebral Trauma/ (157616)
26 exp Joint Dislocations/ (38769)
27 exp Drowning/ (3995)
28 exp Electric Injuries/ (5435)
29 exp Esophageal Perforation/ (4274)
30 exp Fractures, Bone/ (183091)
31 exp Fractures, Cartilage/ (751)
32 exp Hip Injuries/ (30623)
33 exp Lacerations/ (3288)
34 exp Leg Injuries/ (96269)
35 exp Multiple Trauma/ (12815)
36 exp Neck Injuries/ (8002)
37 exp Radiation Injuries/ (69323)
38 exp Retropneumoperitoneum/ (692)

39 exp Rupture/ (48173)
40 exp Shock, Traumatic/ (5129)
41 exp Soft Tissue Injuries/ (5513)
42 exp Spinal Cord Injuries/ (48410)
43 exp Spinal Injuries/ (23080)
44 exp Thoracic Injuries/ (26993)
45 exp Trauma, Nervous System/ (207594)
46 exp Wounds, Nonpenetrating/ (37174)
47 exp Wounds, Penetrating/ (36213)
48 exp Cardiovascular Surgical Procedures/ (388701)
49 6 or 7 or 8 or 9 or 10 or 11 or 12 or 13 or 14 or 15 (164892)
50 16 or 17 or 18 or 19 or 20 or 21 or 22 or 23 or 24 or 25 or 26 or 27 or 28 or 29 or 30 or 31 or 32 or 33 or 34 or 35 or 36 or 37 or 38 or 39 or 40 or 41 or 42 or 43 or 44 or 45 or 46 or 47 or 48 (1273965)
51 49 or 50 (1422692)
52 prophylax$.mp. (110750)
53 exp Primary Prevention/ (150980)
54 exp Secondary Prevention/ (20252)
55 prevent$.mp. (2359560)
56 52 or 53 or 54 or 55 (2468434)
57 5 and 51 and 56 (6888)
58 limit 57 to yr="2010 -Current" (2582)
59 limit 58 to yr="2014 -Current" (1435)

****

11. PRISMA 2020 Checklist

Section and Topic	Item #	Checklist item	Location where item is reported
**TITLE**			
Title	1	Identify the report as a systematic review.	1
**ABSTRACT**			
Abstract	2	See the PRISMA 2020 for Abstracts checklist.	6
**INTRODUCTION**			
Rationale	3	Describe the rationale for the review in the context of existing knowledge.	8-9
Objectives	4	Provide an explicit statement of the objective(s) or question(s) the review addresses.	8-9
**METHODS**			
Eligibility criteria	5	Specify the inclusion and exclusion criteria for the review and how studies were grouped for the syntheses.	9
Information sources	6	Specify all databases, registers, websites, organisations, reference lists and other sources searched or consulted to identify studies. Specify the date when each source was last searched or consulted.	9-10
Search strategy	7	Present the full search strategies for all databases, registers and websites, including any filters and limits used.	9-10, supplement 336-350
Selection process	8	Specify the methods used to decide whether a study met the inclusion criteria of the review, including how many reviewers screened each record and each report retrieved, whether they worked independently, and if applicable, details of automation tools used in the process.	10-11
Data collection process	9	Specify the methods used to collect data from reports, including how many reviewers collected data from each report, whether they worked independently, any processes for obtaining or confirming data from study investigators, and if applicable, details of automation tools used in the process.	10-11
Data items	10a	List and define all outcomes for which data were sought. Specify whether all results that were compatible with each outcome domain in each study were sought (e.g. for all measures, time points, analyses), and if not, the methods used to decide which results to collect.	10-11, supplement 291
	10b	List and define all other variables for which data were sought (e.g. participant and intervention characteristics, funding sources). Describe any assumptions made about any missing or unclear information.	10-11, supplement 289
Study risk of bias assessment	11	Specify the methods used to assess risk of bias in the included studies, including details of the tool(s) used, how many reviewers assessed each study and whether they worked independently, and if applicable, details of automation tools used in the process.	10-11, supplement 146
Effect measures	12	Specify for each outcome the effect measure(s) (e.g. risk ratio, mean difference) used in the synthesis or presentation of results.	11-12, supplement 92,99-103
Synthesis methods	13a	Describe the processes used to decide which studies were eligible for each synthesis (e.g. tabulating the study intervention characteristics and comparing against the planned groups for each synthesis (item #5)).	9-14
	13b	Describe any methods required to prepare the data for presentation or synthesis, such as handling of missing summary statistics, or data conversions.	9-15
	13c	Describe any methods used to tabulate or visually display results of individual studies and syntheses.	14
	13d	Describe any methods used to synthesize results and provide a rationale for the choice(s). If meta-analysis was performed, describe the model(s), method(s) to identify the presence and extent of statistical heterogeneity, and software package(s) used.	14
	13e	Describe any methods used to explore possible causes of heterogeneity among study results (e.g. subgroup analysis, meta-regression).	NA
	13f	Describe any sensitivity analyses conducted to assess robustness of the synthesized results.	NA
Reporting bias assessment	14	Describe any methods used to assess risk of bias due to missing results in a synthesis (arising from reporting biases).	-
Certainty assessment	15	Describe any methods used to assess certainty (or confidence) in the body of evidence for an outcome.	15
**RESULTS**			

Section and Topic	Item #	Checklist item	Location where item is reported
Study selection	16a	Describe the results of the search and selection process, from the number of records identified in the search to the number of studies included in the review, ideally using a flow diagram.	16, Supplement 353
	16b	Cite studies that might appear to meet the inclusion criteria, but which were excluded, and explain why they were excluded.	16, Supplement 353
Study characteristics	17	Cite each included study and present its characteristics.	16, supplement 356-376
Risk of bias in studies	18	Present assessments of risk of bias for each included study.	16-17, supplement 137-145, 181-189, 226-238
Results of individual studies	19	For all outcomes, present, for each study: (a) summary statistics for each group (where appropriate) and (b) an effect estimate and its precision (e.g. confidence/credible interval), ideally using structured tables or plots.	17, supplement 6-136, 165-172, 208-216, 264-275
Results of syntheses	20a	For each synthesis, briefly summarise the characteristics and risk of bias among contributing studies.	17, supplement 147-156, 190-198, and 239-251
	20b	Present results of all statistical syntheses conducted. If meta-analysis was done, present for each the summary estimate and its precision (e.g. confidence/credible interval) and measures of statistical heterogeneity. If comparing groups, describe the direction of the effect.	18-22,, supplement 321-335
	20c	Present results of all investigations of possible causes of heterogeneity among study results.	NA
	20d	Present results of all sensitivity analyses conducted to assess the robustness of the synthesized results.	NA
Reporting biases	21	Present assessments of risk of bias due to missing results (arising from reporting biases) for each synthesis assessed.	NA
Certainty of evidence	22	Present assessments of certainty (or confidence) in the body of evidence for each outcome assessed.	18-22
**DISCUSSION**			
Discussion	23a	Provide a general interpretation of the results in the context of other evidence.	23-28
	23b	Discuss any limitations of the evidence included in the review.	27-29
	23c	Discuss any limitations of the review processes used.	28-29
	23d	Discuss implications of the results for practice, policy, and future research.	29
**OTHER INFORMATION**			
Registration and protocol	24a	Provide registration information for the review, including register name and registration number, or state that the review was not registered.	9
	24b	Indicate where the review protocol can be accessed, or state that a protocol was not prepared.	9
	24c	Describe and explain any amendments to information provided at registration or in the protocol.	9
Support	25	Describe sources of financial or non-financial support for the review, and the role of the funders or sponsors in the review.	3
Competing interests	26	Declare any competing interests of review authors.	3
Availability of data, code and other materials	27	Report which of the following are publicly available and where they can be found: template data collection forms; data extracted from included studies; data used for all analyses; analytic code; any other materials used in the review.	3

From: Page MJ, McKenzie JE, Bossuyt PM, Boutron I, Hoffmann TC, Mulrow CD, et al. The PRISMA 2020 statement: an updated guideline for reporting systematic reviews. BMJ 2021;372:n71. doi: 10.1136/bmj.n71

12. PRISMA 2020 Flow diagram

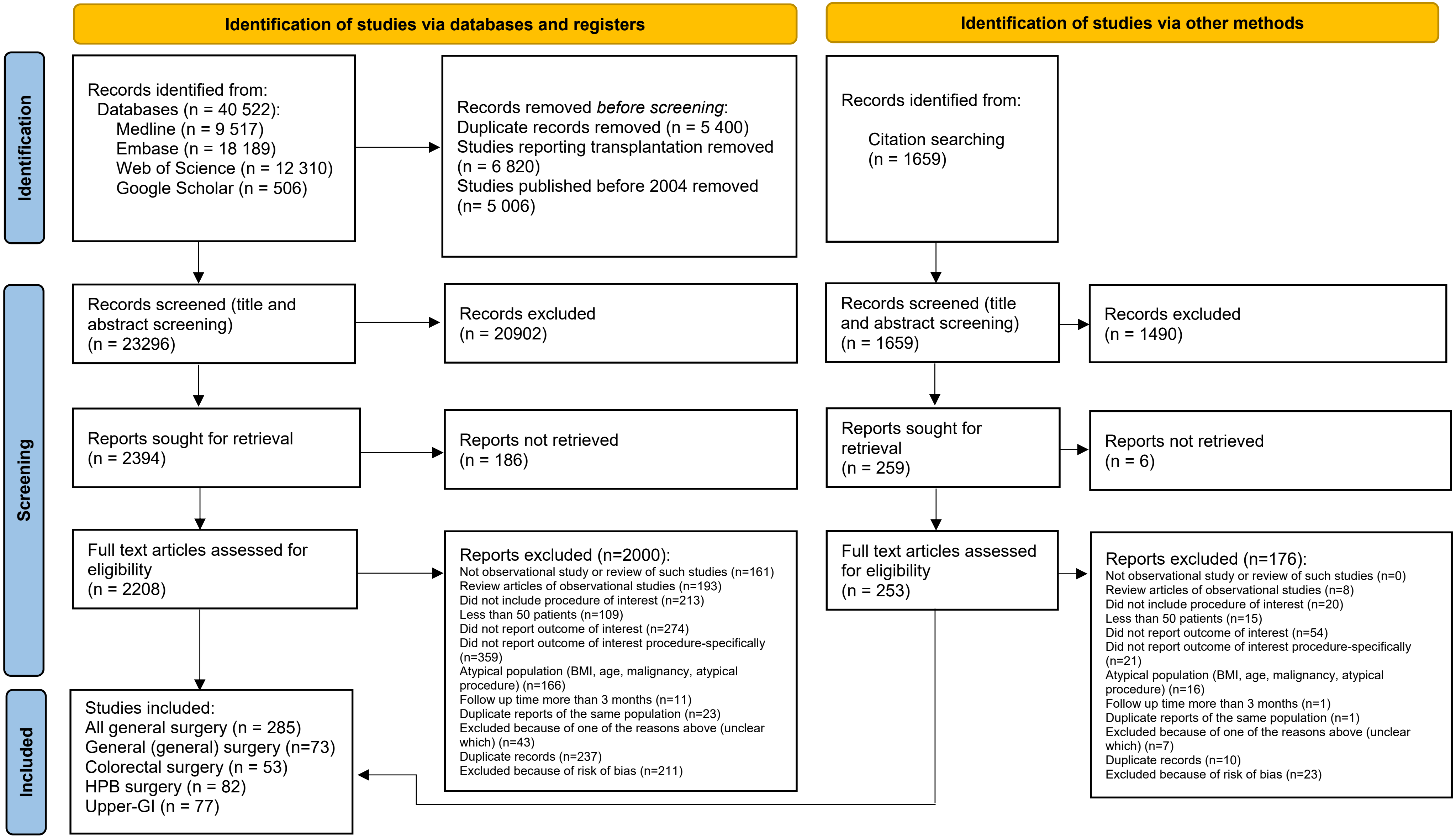

HPB, Hepatopancreatobiliary; Upper-GI, Upper-Gastrointestinal. *From:* Page MJ, McKenzie JE, Bossuyt PM, Boutron I, Hoffmann TC, Mulrow CD, et al. The PRISMA 2020 statement: an updated guideline for reporting systematic reviews. BMJ 2021;372:n71. doi: 10.1136/bmj.n71. For more information, visit: http://www.prisma-statement.org/.

13. MOOSE Checklist for Meta-analyses of Observational Studies

Item No	Recommendation	Reported on Page No
Reporting of background should include		
1	Problem definition	8
2	Hypothesis statement	8
3	Description of study outcome(s)	8
4	Type of exposure or intervention used	8,10
5	Type of study designs used	9
6	Study population	9
Reporting of search strategy should include		
7	Qualifications of searchers (eg, librarians and investigators)	10
8	Search strategy, including time period included in the synthesis and key words	10
9	Effort to include all available studies, including contact with authors	10-11
10	Databases and registries searched	10
11	Search software used, name and version, including special features used (eg, explosion)	10, supplement 336-350
12	Use of hand searching (eg, reference lists of obtained articles)	10
13	List of citations located and those excluded, including justification	16, Supplement 353
14	Method of addressing articles published in languages other than English	10
15	Method of handling abstracts and unpublished studies	10
16	Description of any contact with authors	10-11
Reporting of methods should include		
17	Description of relevance or appropriateness of studies assembled for assessing the hypothesis to be tested	9
18	Rationale for the selection and coding of data (eg, sound clinical principles or convenience)	9
19	Documentation of how data were classified and coded (eg, multiple raters, blinding and interrater reliability)	10
20	Assessment of confounding (eg, comparability of cases and controls in studies where appropriate)	NA
21	Assessment of study quality, including blinding of quality assessors, stratification or regression on possible predictors of study results	15, supplement 146
22	Assessment of heterogeneity	9-10
23	Description of statistical methods (eg, complete description of fixed or random effects models, justification of whether the chosen models account for predictors of study results, dose-response models, or cumulative meta-analysis) in sufficient detail to be replicated	14
24	Provision of appropriate tables and graphics	14
Reporting of results should include		
25	Graphic summarizing individual study estimates and overall estimate	18-22
26	Table giving descriptive information for each study included	17
27	Results of sensitivity testing (eg, subgroup analysis)	NA
28	Indication of statistical uncertainty of findings	17
Reporting of discussion should include		

29	Quantitative assessment of bias (eg, publication bias)	17
30	Justification for exclusion (eg, exclusion of non-English language citations)	9-10, supplement 353
31	Assessment of quality of included studies	17, supplement 147-156, 190-198, and 239-251
Reporting of conclusions should include		
32	Consideration of alternative explanations for observed results	22
33	Generalization of the conclusions (ie, appropriate for the data presented and within the domain of the literature review)	29
34	Guidelines for future research	30
35	Disclosure of funding source	2

From: Stroup DF, Berlin JA, Morton SC, et al, for the Meta-analysis Of Observational Studies in Epidemiology (MOOSE) Group. Meta-analysis of Observational Studies in Epidemiology. A Proposal for Reporting. *JAMA*.

14. List of included studies

1. General abdominal surgery

Author	Year	Citation
Aher	2015	Aher CV, Kubasiak JC, Daly SC, et al. The utilization of laparoscopy in ventral hernia repair: an update of outcomes analysis using ACS-NSQIP data. Surgical Endoscopy. 2015;29(5):1099-104.
Al-Sahaf	2008	Al-Sahaf O, Al-Azawi D, Fauzi MZ, et al. Totally extraperitoneal laparoscopic inguinal hernia repair is a safe option in patients with previous lower abdominal surgery. J Laparoendosc Adv Surg Tech A. 2008;18(3):353-6.
Alizadeh	2017	Alizadeh RF, Sujatha-Bhaskar S, Li S, et al. Venous thromboembolism in common laparoscopic abdominal surgical operations. American Journal of Surgery. 2017;214(6):1127-32.
Basta	2016	Basta MN, Bauder AR, Kovach SJ, et al. Assessing the predictive accuracy of the American College of Surgeons National Surgical Quality Improvement Project Surgical Risk Calculator in open ventral hernia repair. American Journal of Surgery. 2016;212(2):272-81.
Bessa	2015	Bessa SS, Abdel-fattah MR, Al-Sayes IA, et al. Results of prosthetic mesh repair in the emergency management of the acutely incarcerated and/or strangulated groin hernias: a 10-year study. Hernia. 2015;19(6):909-14.
Bittner	2018	Bittner JG, Alrefai S, Vy M, et al. Comparative analysis of open and robotic transversus abdominis release for ventral hernia repair. Surgical Endoscopy. 2018;32(2):727-34.
Blake	2001	Blake AM, Toker SI, Dunn E. Deep venous thrombosis prophylaxis is not indicated for laparoscopic cholecystectomy. Jsls. 2001;5(3):215-9.
Boules	2018	Boules M, Strong AT, Corcelles R, et al. Single-center ventral hernia repair with porcine dermis collagen implant. Surgical Endoscopy. 2018;32(4):1820-7.
Brugger	2011	Brugger L, Rosella L, Candinas D, et al. Improving outcomes after laparoscopic appendectomy: A population-based, 12-year trend analysis of 7446 patients. Annals of Surgery. 2011;253(2):309-13.
Casaccia	2010	Casaccia M, Torelli P, Pasa A, et al. Putative predictive parameters for the outcome of laparoscopic splenectomy: a multicenter analysis performed on the Italian Registry of Laparoscopic Surgery of the Spleen. Ann Surg. 2009;251(2):287-91.
Chung	2019	Chung WS, Chen Y, Chen W, et al. Incidence and risk of venous thromboembolism in patients following appendectomy: a nationwide cohort study. Journal of Thrombosis & Thrombolysis. 2019;48(3):483-90.
Coelho	2019	Coelho JCU, Dalledone GO, Martins Filho EL, et al. Feasibility of Routine Ambulatory Laparoscopic Cholecystectomy in Brazil. Journal of the Society of Laparoendoscopic Surgeons. 2019;23(2).
Corcione	2012	Corcione F, Pirozzi F, Aragiusto G, et al. Laparoscopic splenectomy: experience of a single center in a series of 300 cases. Surg Endosc. 2012;26(10):2870-6.
Daly	2014	Daly SC, Popoff AM, Fogg L, et al. Minimally invasive technique leads to decreased morbidity and mortality in small bowel resections compared to an open technique: an ACS-NSQIP identified target for improvement. J Gastrointest Surg. 2014;18(6):1171-1175. doi:10.1007/s11605-014-2493-5
Delaitre	2002	Delaitre B, Blezel E, Samama G, et al. Laparoscopic splenectomy for idiopathic thrombocytopenic purpura. Surg Laparosc Endosc Percutan Tech. 2002;12(6):412-9.
Donkervoort	2016	Donkervoort SC, Kortram K, Dijksman LM, et al. Anticipation of complications after laparoscopic cholecystectomy: prediction of individual outcome. Surgical Endoscopy. 2016;30(12):5388-94.
Engbaek	2006	Engbaek J, Bartholdy J, Hjortso NC. Return hospital visits and morbidity within 60 days after day surgery: a retrospective study of 18,736 day surgical procedures. Acta Anaesthesiol Scand. 2006;50(8):911-9.
Ferrari	2008	Ferrari GC, Miranda A, Lernia SD, et al. Laparoscopic repair of incisional hernia: Outcomes of 100 consecutive cases comprising 25 wall defects larger than 15 cm. Surgical Endoscopy. 2008;22(5):1173-9.
Garcia	2019	Garcia M, Gerber A, Zakhary B, et al. Management and outcomes of acute appendicitis in the presence of cirrhosis: A nationwide analysis. American Surgeon. 2019;85(1):1129-33.
Gundogdu	2017	Gundogdu RH, Oduncu M, Bozkirli BO, et al. Does thromboprophylaxis cause bleeding after laparoscopic cholecystectomy? Bratisl Lek Listy. 2017;118(3):156-9.
Hasbahceci	2012	Hasbahceci M, Uludag M, Erol C, et al. Laparoscopic cholecystectomy in a single, non-teaching hospital: An analysis of 1557 patients. Journal of Laparoendoscopic and Advanced Surgical Techniques. 2012;22(6):527-32.

Hemmila	2010	Hemmila MR, Birkmeyer NJ, Arbabi S, et al. Introduction to Propensity Scores: A Case Study on the Comparative Effectiveness of Laparoscopic vs Open Appendectomy. Archives of Surgery. 2010;145(10):939-45.
Hernandez	2020	Hernandez S, York TJ, Glencer A, et al. Minimally Invasive Splenectomy Is Associated with Decreased Serious Complications: A 2008-2018 NSQIP Analysis. Journal of the American College of Surgeons. 2020;231.
Holzheimer	2007	Holzheimer RG. Low recurrence rate in hernia repair--results in 300 patients with open mesh repair of primary inguinal hernia. Eur J Med Res. 2007;12(1):1-5.
Ingraham	2010	Ingraham AM, Cohen ME, Ko CY, et al. A current profile and assessment of north american cholecystectomy: results from the american college of surgeons national surgical quality improvement program. J Am Coll Surg. 2010;211(2):176-86.
Jiang	2014	Jiang GQ, Chen P, Qian JJ, et al. Perioperative advantages of modified laparoscopic vs open splenectomy and azygoportal disconnection. World J Gastroenterol. 2014;20(27):9146-53.
Kraft	2019	Kraft CT, Janis JE. Venous Thromboembolism After Abdominal Wall Reconstruction: A Prospective Analysis and Review of the Literature. Plast Reconstr Surg. 2019;15:15.
Li	2017	Li Y, Zhang D, Hua F, et al. Factors associated with the effect of open splenectomy for immune thrombocytopenic purpura. European Journal of Haematology. 2017;98(1):44-51.
Lindberg	2006	Lindberg F, Bjorck M, Rasmussen I, et al. Low frequency of phlebographic deep vein thrombosis after laparoscopic cholecystectomy--a pilot study. Clin Appl Thromb Hemost. 2006;12(4):421-6.
Liu	2020	Liu J, Chen J, Shen Y. The results of open preperitoneal prosthetic mesh repair for acutely incarcerated or strangulated inguinal hernia: a retrospective study of 146 cases. Surgical Endoscopy. 2020;34(1):47-52.
Lomanto	2006	Lomanto D, Iyer SG, Shabbir A, et al. Laparoscopic versus open ventral hernia mesh repair: a prospective study. Surg Endosc. 2006;20(7):1030-5.
Lozano	2015	Lozano FS, Sanchez-Fernandez J, Gonzalez-Porras JR, et al. Slow femoral venous flow and venous thromboembolism following inguinal hernioplasty in patients without or with low molecular weight heparin prophylaxis. Hernia. 2015;19(6):901-8.
McKenna	2018	McKenna NP, Bews KA, Behm KT, et al. Do Patients With Inflammatory Bowel Disease Have a Higher Postoperative Risk of Venous Thromboembolism or Do They Undergo More High-risk Operations? Annals of Surgery. 2018;30:30.
Mesa	2006	Mesa RA, Nagorney DS, Schwager S, et al. Palliative goals, patient selection, and perioperative platelet management: Outcomes and lessons from 3 decades of splenectomy for myelofibrosis with myeloid metaplasia at the Mayo Clinic. Cancer. 2006;107(2):361-70.
Meyer	2013	Meyer A, Blanc P, Balique JG, et al. Laparoscopic totally extraperitoneal inguinal hernia repair: twenty-seven serious complications after 4565 consecutive operations. Rev. 2013;40(1):32-6.
Mita	2020	Mita K, Fujino K, Asakawa H, et al. Postoperative bleeding complications after endoscopic inguinal hernia repair in patients receiving anticoagulation agents, antiplatelet agents, or both. Asian Journal of Endoscopic Surgery. 2020;13(1):71-6.
Nguyen	2007	Nguyen NT, Hinojosa MW, Fayad C, et al. Laparoscopic surgery is associated with a lower incidence of venous thromboembolism compared with open surgery. Annals of Surgery. 2007;246(6):1021-7.
Nilsson	2016	Nilsson H, Angerås U, Sandblom G, et al. Serious adverse events within 30 days of groin hernia surgery. Hernia. 2016;20(3):377-85.
Ntourakis	2011	Ntourakis D, Sergentanis TN, Georgiopoulos I, et al. Subclinical activation of coagulation and fibrinolysis in laparoscopic cholecystectomy: do risk factors exist? International Journal Of Surgery. 2011;9(5):374-7.
Pakaneh	2012	Pakaneh MA, Pazouki A, Tamannaie Z, et al. Results of post-laparoscopic cholecystectomy duplex scan without deep vein thrombosis prophylaxis prior to surgery. Med J Islam Repub Iran. 2012;26(4):164-6.
Patel	2003	Patel AG, Parker JE, Wallwork B, et al. Massive splenomegaly is associated with significant morbidity after laparoscopic splenectomy. Ann Surg. 2003;238(2):235-40.
Perez	2020	Perez AJ, Strassle PD, Sadava EE, et al. Nationwide analysis of inpatient laparoscopic versus open inguinal hernia repair. Journal of Laparoendoscopic and Advanced Surgical Techniques. 2020;30(3):292-8.
Persson	2012	Persson G, Stromberg J, Svennblad B, et al. Risk of bleeding associated with use of systemic thromboembolic prophylaxis during laparoscopic cholecystectomy. Br J Surg. 2012;99(7):979-86.
Poudel	2020	Poudel S, Miyazaki K, Hirano S. Continuation of antithrombotic therapy increases minor bleeding but does not increase the risk other morbidities in open inguinal hernia repair: A propensity score-matched analysis. Hernia. 2020;24(4):857-65.

Radkowiak	2018	Radkowiak D, Zychowicz A, Lasek A, et al. 20 years' experience with laparoscopic splenectomy. Single center outcomes of a cohort study of 500 cases. International Journal of Surgery. 2018;52:285-92.
Rathore	2007	Rathore MA, Andrabi SIH, Mansha M, et al. Day case laparoscopic cholecystectomy is safe and feasible: A case controlled study. International Journal of Surgery. 2007;5(4):255-9.
Romano	2006	Romano F, Caprotti R, Conti M, et al. Thrombosis of the splenoportal axis after splenectomy. Langenbecks Arch Surg. 2006;391(5):483-8.
Rosero	2017	Rosero EB, Joshi GP. Hospital readmission after ambulatory laparoscopic cholecystectomy: incidence and predictors. Journal of Surgical Research. 2017;219:108-15.
Ross	2020	Ross SW, Kuhlenschmidt KM, Kubasiak JC, et al. Association of the Risk of a Venous Thromboembolic Event in Emergency vs Elective General Surgery. JAMA Surgery. 2020;155(6):503-11.
Rysmakhanov	2019	Rysmakhanov M, Aubakirov G, Abdin Z, et al. Laparoscopic Cholecystectomy Complications - Our Expiriens. Hpb. 2019;21.
Sakran	2019	Sakran JV, Ezzeddine H, Haut ER, et al. Prolonged operating room time in emergency general surgery is associated with venous thromboembolic complications. American Journal of Surgery. 2019;218(5):836-41.
Schaepkens Van Riempst	2002	Schaepkens Van Riempst JT, Van Hee RH, Weyler JJ. Deep venous thrombosis after laparoscopic cholecystectomy and prevention with nadroparin. Surg Endosc. 2002;16(1):184-7.
Schmidbauer	2005	Schmidbauer S, Ladurner R, Hallfeldt KK, et al. Heavy-weight versus low-weight polypropylene meshes for open sublay mesh repair of incisional hernia. Eur J Med Res. 2005;10(6):247-53.
Sepassi	2018	Sepassi A, Chingcuanco F, Gordon R, et al. Resource utilization and charges of patients with and without diagnosed venous thromboembolism during primary hospitalization and after elective inpatient surgery: a retrospective study. Journal of Medical Economics. 2018;21(6):595-602.
Sharma	2011	Sharma A, Mehrotra M, Khullar R, et al. Laparoscopic ventral/incisional hernia repair: a single centre experience of 1,242 patients over a period of 13 years. Hernia. 2011;15(2):131-9.
Srsen	2008	Srsen D, Druzijanic N, Pogorelic Z, et al. Quality of life analysis after open and laparoscopic inguinal hernia repair - Retrospective study. Hepato-Gastroenterology. 2008;55(88):2112-5.
Stein	2014	Stein PD, Matta F, Sabra MJ. Pulmonary embolism and deep venous thrombosis following laparoscopic cholecystectomy. Clin Appl Thromb Hemost. 2014;20(3):233-7.
Suuronen	2015	Suuronen S, Kivivuori A, Tuimala J, et al. Bleeding complications in cholecystectomy: a register study of over 22,000 cholecystectomies in Finland. BMC Surg. 2015;15:97.
Tastaldi	2019	Tastaldi L, Krpata DM, Prabhu AS, et al. Emergent groin hernia repair: A single center 10-year experience. Surgery. 2019;165(2):398-405.
Tastaldi	2019	Tastaldi L, Krpata DM, Prabhu AS, et al. Laparoscopic splenectomy for immune thrombocytopenia (ITP): long-term outcomes of a modern cohort. Surgical Endoscopy. 2019;33(2):475-85.
Triantafyllidis	2009	Triantafyllidis I, Nikoloudis N, Sapidis N, et al. Complications of laparoscopic cholecystectomy: our experience in a district general hospital. Surg Laparosc Endosc Percutan Tech. 2009;19(6):449-58.
Tsamalaidze	2018	Tsamalaidze L, Stauffer JA, Brigham T, et al. Postsplenectomy thrombosis of splenic, mesenteric, and portal vein (PST-SMPv): A single institutional series, comprehensive systematic review of a literature and suggested classification. American Journal of Surgery. 2018;216(6):1192-204.
Ulrych	2016	Ulrych J, Kvasnicka T, Fryba V, et al. 28 day post-operative persisted hypercoagulability after surgery for benign diseases: a prospective cohort study. BMC surg. 2016;16:16.
Vecchio	2011	Vecchio R, Marchese S, Swehli E, et al. Splenic hilum management during laparoscopic splenectomy. J Laparoendosc Adv Surg Tech A. 2011;21(8):717-20.
Wakasugi	2016	Wakasugi M, Tei M, Anno K, et al. Single-incision totally extraperitoneal inguinal hernia repair is safe and feasible in elderly patients: A single-center experience of 365 procedures. Asian Journal of Endoscopic Surgery. 2016;9(4):281-4.
Wakasugi	2017	Wakasugi M, Suzuki Y, Tei M, et al. The feasibility and safety of single-incision totally extraperitoneal inguinal hernia repair after previous lower abdominal surgery: 350 procedures at a single center. Surgery Today. 2017;47(3):307-12.
Wang	2020	Wang M, Zhang G, Chen J, et al. Current prevalence of perioperative early venous thromboembolism and risk factors in Chinese adult patients with inguinal hernia (CHAT-1). Scientific Reports. 2020;10(1):12667.
Wang	2013	Wang X, Li Y, Crook N, et al. Laparoscopic splenectomy: A surgeon's experience of 302 patients with analysis of postoperative complications. Surgical Endoscopy. 2013;27(10):3564-71.
Warren	2017	Warren JA, Cobb WS, Ewing JA, et al. Standard laparoscopic versus robotic retromuscular ventral hernia repair. Surg Endosc. 2016;31(1):324-32.

Yang	2019	Yang C, Zhu L. Coagulation and deep vein flow changes following laparoscopic total extraperitoneal inguinal hernia repair: a single-center, prospective cohort study. Surgical Endoscopy. 2019;11:11.
Zhang	2012	Zhang Y, Wen TF, Yan LN, et al. Preoperative predictors of portal vein thrombosis after splenectomy with periesophagogastric devascularization. World Journal of Gastroenterology. 2012;18(15):1834-9.
Zolin	2020	Zolin SJ, Tastaldi L, Alkhatib H, et al. Open retromuscular versus laparoscopic ventral hernia repair for medium-sized defects: where is the value? Hernia. 2020;24(4):759-70.
Zychowicz	2018	Zychowicz A, Radkowiak D, Lasek A, et al. Laparoscopic splenectomy for immune thrombocytopenia in patients with a very low platelet count. Wideochirurgia I Inne Techniki Maloinwazyjne. 2018;13(2):157-63.

2. Colorectal surgery

Author	Year	Reference
Abarca	2011	Abarca F, Saclarides TJ, Brand MI. Laparoscopic colectomy: Complications causing reintervention or emergency room/hospital readmissions. American Surgeon. 2011;77(1):65-9.
Althans	2019	Althans AR, Aiello A, Steele SR, et al. Colectomy for caecal and sigmoid volvulus: a national analysis of outcomes and risk factors for postoperative complications. Colorectal Disease. 2019;21(1):1445-52.
Altomare	2009	Altomare DF, Binda G, Ganio E, et al. Long-term outcome of Altemeier's procedure for rectal prolapse. Dis Colon Rectum. 2009;52(4):698-703.
Alves	2005	Alves A, Panis Y, Slim K, et al. French multicentre prospective observational study of laparoscopic versus open colectomy for sigmoid diverticular disease. Br J Surg. 2005;92(12):1520-5.
Bilimoria	2008	Bilimoria KY, Bentrem DJ, Merkow RP, et al. Laparoscopic-assisted vs. open colectomy for cancer: comparison of short-term outcomes from 121 hospitals. J Gastrointest Surg. 2008;12(11):2001-9.
Causey	2013	Causey MW, Stoddard D, Johnson EK, et al. Laparoscopy impacts outcomes favorably following colectomy for ulcerative colitis: A critical analysis of the ACS-NSQIP database. Surgical Endoscopy. 2013;27(2):603-9.
Chan	2008	Chan AC, Poon JT, Fan JK, et al. Impact of conversion on the long-term outcome in laparoscopic resection of colorectal cancer. Surg Endosc. 2008;22(12):2625-30.
Cuccurullo	2015	Cuccurullo D, Pirozzi F, Sciuto A, et al. Relaparoscopy for management of postoperative complications following colorectal surgery: ten years experience in a single center. Surgical Endoscopy. 2015;29(7):1795-803.
Denet	2017	Denet C, Fuks D, Cocco F, et al. Effects of age after laparoscopic right colectomy for cancer: Are there any specific outcomes? Digestive and Liver Disease. 2017;49(5):562-7.
Ding	2012	Ding JH, Canedo J, Lee SH, et al. Perineal rectosigmoidectomy for primary and recurrent rectal prolapse: Are the results comparable the second time? Diseases of the Colon and Rectum. 2012;55(6):666-70.
Duraes	2018	Duraes LC, Schroeder DA, Dietz DW. Modified pfannenstiel open approach as an alternative to laparoscopic total proctocolectomy and IPAA: Comparison of short- and long-term outcomes and quality of life. Diseases of the Colon and Rectum. 2018;61(5):573-8.
Franco	2018	Franco I, De'Angelis N, Canoui-Poitrine F, et al. Feasibility and safety of laparoscopic right colectomy in oldest-old patients with colon cancer: Results of the CLIMHET Study Group. Journal of Laparoendoscopic and Advanced Surgical Techniques. 2018;28(11):1326-33.
Garrett	2008	Garrett KA, Champagne BJ, Valerian BT, et al. A single training center's experience with 200 consecutive cases of diverticulitis: Can all patients be approached laparoscopically? Surgical Endoscopy and Other Interventional Techniques. 2008;22(11):2503-8.
Gu	2013	Gu J, Stocchi L, Remzi F, et al. Factors associated with postoperative morbidity, reintervention and readmission rates after laparoscopic total abdominal colectomy for ulcerative colitis. Colorectal Dis. 2013;15(9):1123-9.
Gu	2016	Gu J, Stocchi L, Gorgun E, et al. Risk factors associated with portomesenteric venous thrombosis in patients undergoing restorative proctocolectomy for medically refractory ulcerative colitis. Colorectal Disease. 2016;18(4):393-9.
Haskins	2018	Haskins IN, Ju T, Skancke M, et al. Right Colon Resection for Colon Cancer: Does Surgical Approach Matter? J Laparoendosc Adv Surg Tech A. 2018;28(10):1202-6.
Henke	2012	P. K. Henke, S. Arya, C. Pannucci, J. Kubus, S. Hendren, M. Engelsbe, D. Campbell
Ilyas	2017	Ilyas MI, Zangbar B, Nfonsam VN, et al. Are there differences in outcome after elective sigmoidectomy for diverticular disease and for cancer? A national inpatient study. Colorectal Disease. 2017;19(3):260-5.
Iwamoto	2019	Iwamoto K, Takahashi H, Fujii M, et al. Safety of Single-Site Laparoscopic Surgery Requiring Perioperative Heparinization in Colorectal Cancer: Propensity Score-Matched Analysis. Annals of Surgical Oncology. 2019;26(1):4390-6.
Kang	2013	Kang CY, Halabi WJ, Chaudhry OO, et al. Risk factors for anastomotic leakage after anterior resection for rectal cancer. JAMA Surgery. 2013;148(1):65-71.
Kimmins	2001	Kimmins MH, Evetts BK, Isler J, et al. The Altemeier repair: outpatient treatment of rectal prolapse. Dis Colon Rectum. 2001;44(4):565-70.
Krimphove	2020	Krimphove MJ, Reese S, Chen X, et al. Minimally invasive cancer surgery is associated with a lower risk of venous thromboembolic events. Journal of Surgical Oncology. 2020;121(3):578-83.
Kronberg	2011	Kronberg U, Kiran RP, Soliman MS, et al. A characterization of factors determining postoperative ileus after laparoscopic colectomy enables the generation of a novel predictive score. Annals of Surgery. 2011;253(1):78-81.

Lacy	2015	Lacy AM, Tasende MM, Delgado S, et al. Transanal Total Mesorectal Excision for Rectal Cancer: Outcomes after 140 Patients. J Am Coll Surg. 2015;221(2):415-23.
Law	2006	Law WL, Lee YM, Choi HK, et al. Laparoscopic and open anterior resection for upper and mid rectal cancer: an evaluation of outcomes. Dis Colon Rectum. 2006;49(8):1108-15.
Law	2017	Law WL, Foo DCC. Comparison of short-term and oncologic outcomes of robotic and laparoscopic resection for mid- and distal rectal cancer. Surg Endosc. 2016;31(7):2798-807.
Lee	2019	Lee JM, Bai PCJ, El Hechi M, et al. Hartmann's Procedure vs Primary Anastomosis with Diverting Loop Ileostomy for Acute Diverticulitis: Nationwide Analysis of 2,729 Emergency Surgery Patients. Journal of the American College of Surgeons. 2019;229(1):48-55.
Leroy	2005	Leroy J, Ananian P, Rubino F, et al. The impact of obesity on technical feasibility and postoperative outcomes of laparoscopic left colectomy. Ann Surg. 2004;241(1):69-76.
Li	2015	Li Y, Stocchi L, Rui Y, et al. Perioperative Blood Transfusion and Postoperative Outcome in Patients with Crohn's Disease Undergoing Primary Ileocolonic Resection in the "Biological Era". J Gastrointest Surg. 2015;19(10):1842-51.
Liang	2013	Liang JT, Cheng JC, Huang KC, et al. Comparison of tumor recurrence between laparoscopic total mesorectal excision with sphincter preservation and laparoscopic abdominoperineal resection for low rectal cancer. Surg Endosc. 2013;27(9):3452-64.
Magistro	2013	Magistro C, Lernia SD, Ferrari G, et al. Totally laparoscopic versus laparoscopic-assisted right colectomy for colon cancer: is there any advantage in short-term outcomes? A prospective comparative assessment in our center. Surg Endosc. 2013;27(7):2613-8.
Masoomi	2011	Masoomi H, Buchberg B, Nguyen B, et al. Outcomes of laparoscopic versus open colectomy in elective surgery for diverticulitis. World J Surg. 2011;35(9):2143-8.
McKenna	2018	McKenna NP, Bews KA, Behm KT, et al. Do Patients With Inflammatory Bowel Disease Have a Higher Postoperative Risk of Venous Thromboembolism or Do They Undergo More High-risk Operations? Annals of Surgery. 2018;30:30.
Miller	2016	Miller PE, Dao H, Paluvoi N, et al. Comparison of 30-Day Postoperative Outcomes after Laparoscopic vs Robotic Colectomy. Journal of the American College of Surgeons. 2016;223(2):369-73.
Miyagaki	2017	Miyagaki H, Mudiyanselage CH, Pettke E, et al. Fecal diversion in rectal cancer patients undergoing sphincter saving resection is associated with a higher morbidity and readmission rate but a lower reintervention rate vs non-diverted patients. Gastroenterology. 2017;152 (5 Supplement 1):S1298.
Mrdutt	2017	Mrdutt MM, Isbell CL, Thomas JS, et al. Impact of complications on length of stay in elective laparoscopic colectomies. Journal of Surgical Research. 2017;219:180-7.
Osborne	2013	Osborne AJ, Lim J, Gash KJ, et al. Comparison of single-incision laparoscopic high anterior resection with standard laparoscopic high anterior resection. Colorectal Dis. 2012;15(3):329-33.
Park	2015	Park EJ, Cho MS, Baek SJ, et al. Long-term oncologic outcomes of robotic low anterior resection for rectal cancer: a comparative study with laparoscopic surgery. Ann Surg. 2014;261(1):129-37.
Park	2011	Park JS, Choi GS, Jun SH, et al. Laparoscopic versus open intersphincteric resection and coloanal anastomosis for low rectal cancer: intermediate-term oncologic outcomes. Ann Surg. 2011;254(6):941-6.
Posabella	2018	Posabella A, Rotigliano N, Tampakis A, et al. Peripheral vs pedicle division in laparoscopic resection of sigmoid diverticulitis: a 10-year experience. International Journal of Colorectal Disease. 2018;33(7):887-94.
Raskin	2019	Raskin ER, Gorrepati ML, Mehendale S, et al. Robotic-assisted ileocolic resection for Crohn's disease: outcomes from an early national experience. Journal of Robotic Surgery. 2019;13(3):429-34.
Remzi	2002	Remzi FH, Fazio VW, Oncel M, et al. Portal vein thrombi after restorative proctocolectomy. Surgery. 2002;132(4):655-61; discussion 61-2.
Ross	2020	Ross SW, Kuhlenschmidt KM, Kubasiak JC, et al. Association of the Risk of a Venous Thromboembolic Event in Emergency vs Elective General Surgery. JAMA Surgery. 2020;155(6):503-11.
Ryoo	2014	Ryoo SB, Oh HK, Han EC, et al. Complications after ileal pouch-anal anastomosis in Korean patients with ulcerative colitis. World Journal of Gastroenterology. 2014;20(23):7488-96.
Sakran	2019	Sakran JV, Ezzeddine H, Haut ER, et al. Prolonged operating room time in emergency general surgery is associated with venous thromboembolic complications. American Journal of Surgery. 2019;218(5):836-41.
Tooley	2018	Tooley JE, Sceats LA, Bohl DD, et al. Frequency and timing of short-term complications following abdominoperineal resection. Journal of Surgical Research. 2018;231:69-76.
Tuech	2015	Tuech JJ, Karoui M, Lelong B, et al. A step toward NOTES total mesorectal excision for rectal cancer: endoscopic transanal proctectomy. Ann Surg. 2014;261(2):228-33.

Tyler	2012	Tyler J, Fox J, Desai M, et al. Outcomes and costs associated with robotic colectomy in the minimally invasive era. Diseases of the Colon and Rectum. 2012;55 (5):e76.
Umanskiy	2010	Umanskiy K, Malhotra G, Chase A, et al. Laparoscopic colectomy for Crohn's colitis. A large prospective comparative study. J Gastrointest Surg. 2010;14(4):658-63.
Vogel	2020	Vogel JD, e Campos-Lobato LF, Chapman BC, et al. Rectal prolapse surgery in males and females: An ACS NSQIP-based comparative analysis of over 12,000 patients. American Journal of Surgery. 2020;220(3):697-705.
Weber	2020	Weber KT, Chung PJ, La Gamma N, et al. Effect of Body Mass Index on Outcomes After Surgery for Perforated Diverticulitis. Journal of Surgical Research. 2020;247:220-6.
Wright	2016	Wright JD, Chen L, Jorge S, et al. Prescription of extended-duration thromboprophylaxis after high-risk, abdominopelvic cancer surgery. Gynecol Oncol. 2016;141(3):531-7.
Yamamoto	2004	Yamamoto S, Fujita S, Akasu T, et al. A comparison of the complication rates between laparoscopic colectomy and laparoscopic low anterior resection. Surgical Endoscopy and Other Interventional Techniques. 2004;18(10):1447-51.

3. Upper-gastrointestinal and hepatopancreatobiliary surgery

Author	Year	Reference
Abou-Nukta	2006	Abou-Nukta F, Alkhoury F, Arroyo K, et al. Clinical pulmonary embolus after gastric bypass surgery. Surg. 2006;2(1):24-8; discussion 9.
Abu Hilal	2010	Abu Hilal M, Underwood T, Taylor MG, et al. Bleeding and hemostasis in laparoscopic liver surgery. Surg Endosc. 2009;24(3):572-7.
Abuoglu	2019	Abuoglu HH, Muftuoglu MAT, Odabasi M. A New Protocol for Venous Thromboembolism Prophylaxis in Bariatric Surgery. Obesity Surgery. 2019;29(2):729-34.
Acevedo	2020	Acevedo E, Mazzei M, Zhao H, et al. Outcomes in conventional laparoscopic versus robotic-assisted primary bariatric surgery: a retrospective, case-controlled study of the MBSAQIP database. Surgical Endoscopy. 2020;34(3):1353-65.
Adam	2004	Adam U, Makowiec F, Riediger H, et al. Risk factors for complications after pancreatic head resection. American Journal of Surgery. 2004;187(2):201-8.
Ainoa	2020	Ainoa E, Uutela A, Nordin A, et al. Perioperative vs. postoperative thromboprophylaxis in liver surgery. Hpb. 2020;22.
Alhossaini	2019	Alhossaini RM, Altamran AA, Choi S, et al. Similar operative outcomes between the da vinci xi and da vinci si systems in robotic gastrectomy for gastric cancer. Journal of Gastric Cancer. 2019;19(2):165-72.
AlKhaldi	2019	AlKhaldi LK, AlSaffar NA, AlHamdan F, et al. Long-term outcomes after laparoscopic sleeve gastrectomy in Kuwait. Annals of Saudi Medicine. 2019;39(2):100-3.
Alsina	2014	Alsina E, Ruiz-Tovar J, Alpera MR, et al. Incidence of Deep Vein Thrombosis and Thrombosis of the Portal Mesenteric Axis After Laparoscopic Sleeve Gastrectomy. J Laparoendosc Adv Surg Tech. 2014;24(9):601-5.
Alzahrani	2020	Alzahrani SM, Ko CS, Yoo MW. Validation of the ACS NSQIP Surgical Risk Calculator for Patients with Early Gastric Cancer Treated with Laparoscopic Gastrectomy. Journal of Gastric Cancer. 2020;20(3):267-76.
Andres	2011	Andres A, Toso C, Moldovan B, et al. Complications of elective liver resections in a center with low mortality: a simple score to predict morbidity. Arch Surg. 2011;146(11):1246-52.
Anonsen	2015	Anonsen KV, Buanes T, Rosok BI, et al. Outcome of laparoscopic surgery in patients with cystic lesions in the distal pancreas. Journal of the Pancreas. 2015;16(3):266-70.

Aramaki	2014	Aramaki O, Takayama T, Higaki T, et al. Decreased blood loss reduces postoperative complications in resection for hepatocellular carcinoma. Journal of Hepato-Biliary-Pancreatic Sciences. 2014;21(8):585-91.
Ayloo	2011	Ayloo SM, Addeo P, Buchs NC, et al. Robot-assisted versus laparoscopic Roux-en-Y gastric bypass: is there a difference in outcomes? World J Surg. 2010;35(3):637-42.
Ayloo	2016	Ayloo S, Roh Y, Choudhury N. Laparoscopic, hybrid, and totally robotic Roux-en-Y gastric bypass. Journal of Robotic Surgery. 2016;10(1):41-7.
Bagante	2016	Bagante F, Spolverato G, Strasberg SM, et al. Minimally Invasive vs. Open Hepatectomy: a Comparative Analysis of the National Surgical Quality Improvement Program Database. Journal of Gastrointestinal Surgery. 2016;20(9):1608-17.
Balachandran	2004	Balachandran P, Sikora SS, Raghavendra Rao RV, et al. Haemorrhagic complications of pancreaticoduodenectomy. ANZ J Surg. 2004;74(11):945-50.
Barbas	2013	Barbas AS, Turley RS, Mallipeddi MK, et al. Examining reoperation and readmission after hepatic surgery. Journal of the American College of Surgeons. 2013;216(5):915-23.
Benizri	2013	Benizri EI, Renaud M, Reibel N, et al. Perioperative outcomes after totally robotic gastric bypass: a prospective nonrandomized controlled study. Am J Surg. 2013;206(2):145-51.
Bhojani	2012	Bhojani FD, Fox A, Pitzul K, et al. Clinical and economic comparison of laparoscopic to open liver resections using a 2-to-1 matched pair analysis: an institutional experience. J Am Coll Surg. 2011;214(2):184-95.
Biertho	2014	Biertho L, Lebel S, Marceau S, et al. Laparoscopic sleeve gastrectomy: with or without duodenal switch? A consecutive series of 800 cases. Dig Surg. 2014;31(1):48-54.
Blanc	2007	Blanc T, Cortes A, Goere D, et al. Hemorrhage after pancreaticoduodenectomy: when is surgery still indicated? Am J Surg. 2007;194(1):3-9.
Boone	2019	Boone BA, Zenati MS, Rieser C, et al. Risk of Venous Thromboembolism for Patients with Pancreatic Ductal Adenocarcinoma Undergoing Preoperative Chemotherapy Followed by Surgical Resection. Annals of Surgical Oncology. 2019;26(5):1503-11.
Brunetti	2018	Brunetti L, Wassef A, Sadek R, et al. Anticoagulant activity of enoxaparin and unfractionated heparin for venous thromboembolism prophylaxis in obese patients undergoing sleeve gastrectomy. Surg. 2018;20:20.

Cao	2020	Cao X, Wang X, Zhao B, et al. Correlation between Intraoperative Fluid Administration and Outcomes of Pancreatoduodenectomy. Gastroenterology Research and Practice. 2020;2020.
Caruana	2009	Caruana JA, Anain PM, Pham DT. The pulmonary embolism risk score system reduces the incidence and mortality of pulmonary embolism after gastric bypass. Surgery. 2009;146(4):678-83; discussion 83-5.
Cauchy	2015	Cauchy F, Fuks D, Nomi T, et al. Risk factors and consequences of conversion in laparoscopic major liver resection. Br J Surg. 2015;102(7):785-95.
Chen	2016	Chen K, Pan Y, Cai JQ, et al. Totally laparoscopic versus laparoscopicassisted total gastrectomy for upper and middle gastric cancer: A single-unit experience of 253 cases with meta-analysis. World Journal of Surgical Oncology. 2016;14 (1) (no pagination)(96).
Chen	2016	Chen K, Wu D, Pan Y, et al. Totally laparoscopic gastrectomy using intracorporeally stapler or hand-sewn anastomosis for gastric cancer: a single-center experience of 478 consecutive cases and outcomes. World J Surg Oncol. 2016;14:115.
Chen	2017	Chen K, Pan Y, Zhai ST, et al. Totally laparoscopic versus open total gastrectomy for gastric cancer: A case-matched study about short-term outcomes. Medicine (United States). 2017;96 (38) (no pagination)(e8061).
Chen	2019	Chen K, Pan Y, Mou YP, et al. Evolution of Laparoscopic Pancreatic Resections for Pancreatic and Periampullary Diseases: Perioperative Outcomes of 605 Patients at a High-Volume Center. Journal of Laparoendoscopic and Advanced Surgical Techniques. 2019;29(9):1085-92.
Cipriani	2018	Cipriani F, Ratti F, Fiorentini G, et al. Effect of previous abdominal surgery on laparoscopic liver resection: Analysis of feasibility and risk factors for conversion. Journal of Laparoendoscopic and Advanced Surgical Techniques. 2018;28(7):785-91.
Consortium Longitudinal Assessment of Bariatric Surgery, Flum	2009	Consortium Longitudinal Assessment of Bariatric S, Flum DR, Belle SH, et al. Perioperative safety in the longitudinal assessment of bariatric surgery. N Engl J Med. 2009;361(5):445-54.
Cotter	2005	Cotter SA, Cantrell W, Fisher B, et al. Efficacy of venous thromboembolism prophylaxis in morbidly obese patients undergoing gastric bypass surgery. Obesity Surgery. 2005;15(9):1316-20.

Dagher	2009	Dagher I, O'Rourke N, Geller DA, et al. Laparoscopic major hepatectomy: an evolution in standard of care. Ann Surg. 2009;250(5):856-60.
Dagher	2010	Dagher I, Belli G, Fantini C, et al. Laparoscopic hepatectomy for hepatocellular carcinoma: a European experience. J Am Coll Surg. 2010;211(1):16-23.
Daniel	2018	Daniel F, Tamim H, Hosni M, et al. Short-term surgical morbidity and mortality of distal pancreatectomy performed for benign vs malignant diseases: A NSQIP analysis. United European Gastroenterology Journal. 2018;6 (8 Supplement):A423.
Daskalaki	2017	Daskalaki D, Gonzalez-Heredia R, Brown M, et al. Financial Impact of the Robotic Approach in Liver Surgery: A Comparative Study of Clinical Outcomes and Costs Between the Robotic and Open Technique in a Single Institution. J Laparoendosc Adv Surg Tech A. 2017;27(4):375-82.
de'Angelis	2016	e'Angelis N, Pascal G, Salloum C, et al. Central Hepatectomy versus Extended Hepatectomy for Malignant Tumors: A Propensity Score Analysis of Postoperative Complications. World J Surg. 2016;40(11):2745-57.
Dedania	2013	Dedania N, Agrawal N, Winter JM, et al. Splenic vein thrombosis is associated with an increase in pancreas-specific complications and reduced survival in patients undergoing distal pancreatectomy for pancreatic exocrine cancer. Journal of Gastrointestinal Surgery. 2013;17(8):1392-8.
Dokmak	2017	Dokmak S, Fteriche FS, Aussilhou B, et al. The Largest European Single-Center Experience: 300 Laparoscopic Pancreatic Resections. Journal of the American College of Surgeons. 2017;225(2):226-34.e2.
Dugan	2020	Dugan N, Thompson KJ, Barbat S, et al. Male gender is an independent risk factor for patients undergoing laparoscopic sleeve gastrectomy or Roux-en-Y gastric bypass: an MBSAQIP R database analysis. Surgical Endoscopy. 2020;34(8):3574-83.
Ecker	2016	Ecker BL, Maduka R, Ramdon A, et al. Resident education in robotic-assisted vertical sleeve gastrectomy: outcomes and cost-analysis of 411 consecutive cases. Surg. 2016;12(2):313-20.
Enomoto	2014	Enomoto LM, Hollenbeak CS, Bhayani NH, et al. Measuring surgical quality: a national clinical registry versus administrative claims data. Journal of Gastrointestinal Surgery. 2014;18(8):1416-22.
Faraj	2019	Faraj W, Nassar H, Zaghal A, et al. Pancreaticoduodenectomy in the Middle East: Achieving optimal results through specialization and standardization. Hepatobiliary and Pancreatic Diseases International. 2019;18(5):478-83.

Feng	2014	Feng J, Chen YL, Dong JH, et al. Post-pancreaticoduodenectomy hemorrhage: Risk factors, managements and outcomes. Hepatobiliary and Pancreatic Diseases International. 2014;13(5):513-22.
Fernandez Jr	2004	A. Z. Fernandez J, Demaria EJ, Tichansky DS, et al. Multivariate Analysis of Risk Factors for Death Following Gastric Bypass for Treatment of Morbid Obesity. Annals of Surgery. 2004;239(5):698-703.
Finks	2011	J. F. Finks KLK, P. R. Yenumula WJE, K. R. Krause AMC, et al. Predicting risk for serious complications with bariatric surgery: results from the Michigan Bariatric Surgery Collaborative. Annals of Surgery. 2011;254(4):633-40.
Flis	2016	Flis V, Potrc S, Kobilica N, et al. Pancreaticoduodenectomy for ductal adenocarcinoma of the pancreatic head with venous resection. Radiol Oncol. 2016;50(3):321-8.
Froehling	2012	Froehling DA, Daniels P, Mauck KF, et al. Incidence of venous thromboembolism (VTE) after bariatric surgery: A population-based cohort study. Journal of General Internal Medicine. 2012;2):S227-S8.
Fujikawa	2018	Fujikawa T, Kawamoto H, Tanaka A. Effect of antiplatelet therapy on surgical blood loss and post-pancreatectomy hemorrhage in patients undergoing pancreaticoduodenectomy. Journal of Gastroenterology and Hepatology Research. 2018;7(2):2561-8.
Fuks	2016	Fuks D, Cauchy F, Fteriche S, et al. Laparoscopy Decreases Pulmonary Complications in Patients Undergoing Major Liver Resection: A Propensity Score Analysis. Annals of Surgery. 2016;263(2):353-61.
Gambhir	2020	Gambhir S, Inaba CS, Alizadeh RF, et al. Venous thromboembolism risk for the contemporary bariatric surgeon. Surgical Endoscopy. 2020;34(8):3521-6.
Gargiulo	2006	Gargiulo NJ, Veith FJ, Lipsitz EC, et al. Experience with inferior vena cava filter placement in patients undergoing open gastric bypass procedures. J Vasc Surg. 2006;44(6):1301-5.
Gargiulo	2007	Gargiulo NJ, Veith FJ, Lipsitz EC, et al. The incidence of pulmonary embolism in open versus laparoscopic gastric bypass. Ann Vasc Surg. 2007;21(5):556-9.
Glenn	2015	Glenn JA, Turaga KK, Gamblin TC, et al. Minimally invasive gastrectomy for cancer: current utilization in US academic medical centers. Surgical Endoscopy. 2015;29(12):3768-75.

Guerrier	2018	Guerrier JB, Dietch ZC, Schirmer BD, et al. Laparoscopic Sleeve Gastrectomy Is Associated with Lower 30-Day Morbidity Versus Laparoscopic Gastric Bypass: an Analysis of the American College of Surgeons NSQIP. Obesity Surgery. 2018;28(11):3567-72.
Han	2016	Han JW, Kong SH, Shin CI, et al. Portomesenteric vein thrombosis after gastric surgery. Gastric Cancer. 2016;19(4):1135-43.
Hiki	2018	Hiki N, Honda M, Etoh T, et al. Higher incidence of pancreatic fistula in laparoscopic gastrectomy. Real-world evidence from a nationwide prospective cohort study. Gastric Cancer. 2018;21(1):162-70.
Hutter	2011	Hutter MM, Schirmer BD, Jones DB, et al. First report from the American College of Surgeons Bariatric Surgery Center Network: laparoscopic sleeve gastrectomy has morbidity and effectiveness positioned between the band and the bypass. Ann Surg. 2011;254(3):410-20; discussion 20-2.
Inaba	2018	Inaba CS, Koh CY, Sujatha-Bhaskar S, et al. One-Year Mortality after Contemporary Laparoscopic Bariatric Surgery: An Analysis of the Bariatric Outcomes Longitudinal Database. Journal of the American College of Surgeons. 2018;226(6):1166-74.
Johari	2020	Johari Y, Ooi G, Burton P, et al. Long-Term Matched Comparison of Adjustable Gastric Banding Versus Sleeve Gastrectomy: Weight Loss, Quality of Life, Hospital Resource Use and Patient-Reported Outcome Measures. Obesity Surgery. 2020;30(1):214-23.
Kantor	2018	Kantor O, Talamonti MS, Wang CH, et al. The extent of vascular resection is associated with perioperative outcome in patients undergoing pancreaticoduodenectomy. Hpb. 2018;20(2):140-6.
Kazaryan	2010	Kazaryan AM, Pavlik Marangos I, Rosseland AR, et al. Laparoscopic liver resection for malignant and benign lesions: ten-year Norwegian single-center experience. Arch Surg. 2010;145(1):34-40.
Kendrick	2010	Kendrick ML, Cusati D. Total laparoscopic pancreaticoduodenectomy: feasibility and outcome in an early experience. Archives of Surgery. 2010;145(1):19-23.
Khandoga	2017	Khandoga A, Drefs M, Schoenberg M, et al. Differential significance of early surgical complications for acute and long-term recurrence-free survival following surgical resection of hepatocellular carcinoma: Do comorbidities play a role? European Journal of Gastroenterology and Hepatology. 2017;29(9):1045-53.
Kingham	2016	Kingham TP, Leung U, Kuk D, et al. Robotic Liver Resection: A Case-Matched Comparison. World J Surg. 2016;40(6):1422-8.

Kneuertz	2011	Kneuertz PJ, Patel SH, Chu CK, et al. Effects of perioperative red blood cell transfusion on disease recurrence and survival after pancreaticoduodenectomy for ductal adenocarcinoma. Ann Surg Oncol. 2011;18(5):1327-34.
Kokudo	2014	Kokudo T, Hasegawa K, Sugawara Y, et al. Hepatic vein tumor thrombus of hepatocellular carcinoma is not contraindication for surgery. Hpb. 2014;2):213.
Komokata	2020	Komokata T, Aryal B, Tada N, et al. Impact of antithrombotic therapy on the outcomes with focus on bleeding and thromboembolic events in patients undergoing pancreticoduodenectomy. ANZ journal of surgery. 2020;07.
Kothari	2007	Kothari SN, Lambert PJ, Mathiason MA. A comparison of thromboembolic and bleeding events following laparoscopic gastric bypass in patients treated with prophylactic regimens of unfractionated heparin or enoxaparin. American Journal of Surgery. 2007;194(6):709-11.
Koukoutsis	2006	Koukoutsis I, Bellagamba R, Morris-Stiff G, et al. Haemorrhage following pancreaticoduodenectomy: risk factors and the importance of sentinel bleed. Dig Surg. 2006;23(4):224-8.
Kron	2019	Kron P, Kimura N, Farid S, et al. Current role of trisectionectomy for hepatopancreatobiliary malignancies. Annals of Gastroenterological Surgery. 2019;3(6):606-19.
Kung	2017	Kung CH, Song H, Ye W, et al. Extent of lymphadenectomy has no impact on postoperative complications after gastric cancer surgery in Sweden. Chinese Journal of Cancer Research. 2017;29(4):313-22.
Kwon	2016	Kwon W, Jang JY, Kim JH, et al. An analysis of complications, quality of life, and nutritional index after laparoscopic distal pancreatectomy with regard to spleen preservation. Journal of Laparoendoscopic and Advanced Surgical Techniques. 2016;26(5):335-42.
Lamb	2008	Lamb P, Sivashanmugam T, White M, et al. Gastric cancer surgery - A balance of risk and radically. Annals of the Royal College of Surgeons of England. 2008;90(3):235-42.
Lee	2008	Lee SE, Jang JY, Lee KU, et al. Clinical comparison of distal pancreatectomy with or without splenectomy. J Korean Med Sci. 2008;23(6):1011-4.
Lee	2009	Lee KF, Wong J, Ng W, et al. Feasibility of liver resection without the use of the routine Pringle manoeuver: an analysis of 248 consecutive cases. Hpb. 2009;11(4):332-8.
Lidor	2014	Lidor AO, Moran-Atkin E, Stem M, et al. Hospital-acquired conditions after bariatric surgery: we can predict, but can we prevent? Surgical Endoscopy. 2014;28(12):3285-92.

Lordan	2009	Lordan JT, Worthington TR, Quiney N, et al. Early postoperative outcomes following hepatic resection for benign liver disease in 79 consecutive patients. Hpb. 2009;11(4):321-5.
Mamidanna	2013	Mamidanna R, Almoudaris AM, Bottle A, et al. National outcomes and uptake of laparoscopic gastrectomy for cancer in England. Surgical Endoscopy. 2013;27(9):3348-58.
Mañas-Gómez	2011	Mañas-Gómez MJ, Rodríguez-Revuelto R, Balsells-Valls J, et al. Post-pancreaticoduodenectomy hemorrhage. Incidence, diagnosis, and treatment. World J Surg. 2011;35(11):2543-8.
Martignoni	2001	Martignoni ME, Wagner M, Krähenbühl L, et al. Effect of preoperative biliary drainage on surgical outcome after pancreatoduodenectomy. Am J Surg. 2001;181(1):52-9; discussion 87.
Martins-Filho	2008	Martins-Filho ED, Camara-Neto JB, Ferraz AA, et al. Evaluation of risk factors in superobese patients submitted to conventional Fobi-Capella surgery. Arq Gastroenterol. 2008;45(1):3-10.
Masoomi	2012	Masoomi H, Nguyen NT, Stamos MJ, et al. Overview of outcomes of laparoscopic and open Roux-en-Y gastric bypass in the United States. Surg Technol Int. 2012;22:72-6.
Mataki	2019	Mataki Y. Examination for gastrointestinal hemorrhage after pancreatoduodenectomy. Pancreas. 2019;48:1486-7.
Moon	2018	Moon RC, Ghanem M, Teixeira AF, et al. Assessing risk factors, presentation, and management of portomesenteric vein thrombosis after sleeve gastrectomy: a multicenter case-control study. Surg. 2018;14(4):478-83.
Moradian	2017	Moradian S, Daneshpajouh A, Patel A, et al. Laparoscopic sleeve gastrectomy without over-sewing the staple line: A case series demonstrating efficacy and minimization of both intra- and post-operative complications. International Journal of Surgery Open. 2017;8:7-10.
Mussle	2020	Mussle B, Buck N, Schade S, et al. Impact of pulmonary embolism on morbidity and mortality in patients undergoing pancreatic surgery. Langenbeck's Archives of Surgery. 2020.
Myers	2013	Myers SR, McGuirl J, Wang J. Robot-assisted versus laparoscopic gastric bypass: Comparison of short-term outcomes. Obesity Surgery. 2013;23(4):467-73.
Nakamura	2015	Nakamura M, Wakabayashi G, Miyasaka Y, et al. Multicenter comparative study of laparoscopic and open distal pancreatectomy using propensity score-matching. J Hepatobiliary Pancreat Sci. 2015;22(10):731-6.

Nakauchi	2016	Nakauchi M, Suda K, Susumu S, et al. Comparison of the long-term outcomes of robotic radical gastrectomy for gastric cancer and conventional laparoscopic approach: a single institutional retrospective cohort study. Surgical Endoscopy. 2016;30(12):5444-52.
Nathan	2014	Nathan H, Weiss MJ, Soff GA, et al. Pharmacologic prophylaxis, postoperative INR, and risk of venous thromboembolism after hepatectomy. Journal of Gastrointestinal Surgery. 2014;18(2):295-302; discussion -3.
Nguyen	2007	Nguyen NT, Hinojosa M, Fayad C, et al. Use and outcomes of laparoscopic versus open gastric bypass at academic medical centers. Journal of the American College of Surgeons. 2007;205(2):248-55.
Nielsen	2018	Nielsen AW, Helm MC, Kindel T, et al. Perioperative bleeding and blood transfusion are major risk factors for venous thromboembolism following bariatric surgery. Surg Endosc. 2018;32(5):2488-2495. doi:10.1007/s00464-017-5951-9
Nimeri	2018	Nimeri AA, Bautista J, Ibrahim M, et al. Mandatory Risk Assessment Reduces Venous Thromboembolism in Bariatric Surgery Patients. Obesity Surgery. 2018;28(2):541-7.
Nobili	2012	Nobili C, Marzano E, Oussoultzoglou E, et al. Multivariate analysis of risk factors for pulmonary complications after hepatic resection. Annals of Surgery. 2012;255(3):540-50.
Ntutumu	2016	Ntutumu R, Liu H, Zhen L, et al. Risk factors for pulmonary complications following laparoscopic gastrectomy: A single-center study. Medicine (Baltimore). 2016;95(32):e4567.
Oh	2009	Oh SJ, Hyung WJ, Li C, et al. Effect of being overweight on postoperative morbidity and long-term surgical outcomes in proximal gastric carcinoma. J Gastroenterol Hepatol. 2008;24(3):475-9.
Okabe	2019	Okabe H, Obama K, Tsunoda S, et al. Feasibility of robotic radical gastrectomy using a monopolar device for gastric cancer. Surgery Today. 2019;49(1):820-7.
Osaki	2018	Osaki T, Saito H, Fukumoto Y, et al. Risk and incidence of perioperative deep vein thrombosis in patients undergoing gastric cancer surgery. Surgery Today. 2018;48(5):525-33.
Pandanaboyana	2010	Pandanaboyana, S., Fawzi A, Fulke JL, et al. Late post pancreatectomy haemorrhage. Risk factors and modern management. Jop. 2010;11(3):220-5.
Papenfuss	2014	Papenfuss WA, Kukar M, Oxenberg J, et al. Morbidity and mortality associated with gastrectomy for gastric cancer. Ann Surg Oncol. 2014;21(9):3008-14.

Park	2005	Park DJ, Lee HJ, Kim HH, et al. Predictors of operative morbidity and mortality in gastric cancer surgery. Br J Surg. 2005;92(9):1099-102.
Pedrazzani	2007	Pedrazzani C, Marrelli D, Rampone B, et al. Postoperative complications and functional results after subtotal gastrectomy with Billroth II reconstruction for primary gastric cancer. Dig Dis Sci. 2007;52(8):1757-63.
Rabl	2011	Rabl C, Peeva S, Prado K, et al. Early and late abdominal bleeding after Roux-en-Y gastric bypass: sources and tailored therapeutic strategies. Obes Surg. 2011;21(4):413-20.
Ravikumar	2014	Ravikumar R, Sabin C, Abu Hilal M, et al. Portal vein resection in borderline resectable pancreatic cancer: a United Kingdom multicenter study. J Am Coll Surg. 2014;218(3):401-11.
Ricci	2012	Ricci C, Casadei R, Buscemi S, et al. Late postpancreatectomy hemorrhage after pancreaticoduodenectomy: is it possible to recognize risk factors? Jop. 2012;13(2):193-8.
Romero	2013	Romero RJ, Kosanovic R, Rabaza JR, et al. Robotic sleeve gastrectomy: experience of 134 cases and comparison with a systematic review of the laparoscopic approach. Obes Surg. 2013;23(11):1743-52.
Rosemurgy	2019	Rosemurgy A, Ross S, Bourdeau T, et al. Robotic Pancreaticoduodenectomy Is the Future: Here and Now. Journal of the American College of Surgeons. 2019;228(4):613-24.
Rystedt	2019	Rystedt J, Tingstedt B, Ansorge C, et al. Major intraoperative bleeding during pancreatoduodenectomy - preoperative biliary drainage is the only modifiable risk factor. Hpb. 2019;21(3):268-74.
Sada	2020	Sada A, Asaad M, Reidt WS, et al. Are In-Person Post-operative Clinic Visits Necessary to Detect Complications Among Bariatric Surgery Patients? Obesity Surgery. 2020;30(5):2062-5.
Sah	2009	Sah BK, Chen MM, Yan M, et al. Gastric cancer surgery: Billroth I or Billroth II for distal gastrectomy? BMC Cancer. 2009;9 (no pagination)(428).
Saka	2010	Saka M, Morita S, Fukagawa T, et al. Incidence of pulmonary thromboembolism in gastric cancer surgery using routine thromboprophylaxis. Gastric Cancer. 2010;13(2):117-22.
Sakamoto	2020	Sakamoto T, Fujiogi M, Matsui H, et al. Short-Term Outcomes of Laparoscopic and Open Total Gastrectomy for Gastric Cancer: A Nationwide Retrospective Cohort Analysis. Annals of Surgical Oncology. 2020;27(2):518-26.
Sakran	2016	Sakran N, Raziel A, Goitein O, et al. Laparoscopic Sleeve Gastrectomy for Morbid Obesity in 3003 Patients: Results at a High-Volume Bariatric Center. Obesity Surgery. 2016;26(9):2045-50.

Santo	2013	Santo MA, Pajecki D, Riccioppo D, et al. Early complications in bariatric surgery: incidence, diagnosis and treatment. Arq Gastroenterol. 2013;50(1):50-5.
Sexton	2008	Sexton JA, Pierce RA, Halpin VJ, et al. Laparoscopic gastric resection for gastrointestinal stromal tumors. Surgical Endoscopy. 2008;22(12):2583-7.
Shibasaki	2020	Shibasaki S, Suda K, Nakauchi M, et al. Non-robotic minimally invasive gastrectomy as an independent risk factor for postoperative intra-abdominal infectious complications: A single-center, retrospective and propensity score-matched analysis. World Journal of Gastroenterology. 2020;26(1):1172-84.
Shimada	2018	Shimada S, Sawada N, Ishiyama Y, et al. Impact of obesity on short- and long-term outcomes of laparoscopy assisted distal gastrectomy for gastric cancer. Surgical Endoscopy. 2018;32(1):358-66.
Singh	2017	Singh SA, Vivekananthan P, Sharma A, et al. Retrospective analysis of post-operative coagulopathy after major hepatic resection at a tertiary care centre in Northern India. Indian J. 2017;61(7):575-80.
Slotman	2010	Slotman GJ. Non-transectional open gastric bypass as the definitive bariatric procedure for 61 patients with BMI of 70 and higher. Obesity Surgery. 2010;20(1):7-12.
Snyder	2019	Snyder RA, Prakash LR, Nogueras-Gonzalez GM, et al. Perioperative blood transfusions for vein resection during pancreaticoduodenectomy for pancreatic adenocarcinoma: Identification of clinical targets for optimization. Hpb. 2019;21(7):841-8.
Snyder	2020	Snyder RA, Ewing JA, Parikh AA. Preoperative Portal Vein Embolization Is Not Associated with Increased Postoperative Complications After Major Hepatectomy: a Study of the National Surgical Quality Improvement Database. Journal of Gastrointestinal Surgery. 2020;24(7):1561-70.
Son	2014	Son T, Lee JH, Kim YM, et al. Robotic spleen-preserving total gastrectomy for gastric cancer: comparison with conventional laparoscopic procedure. Surg Endosc. 2014;28(9):2606-15.
Song	2009	Song J, Oh SJ, Kang WH, et al. Robot-assisted gastrectomy with lymph node dissection for gastric cancer: lessons learned from an initial 100 consecutive procedures. Ann Surg. 2009;249(6):927-32.
Song	2020	Song KB, Kim SC, Lee W, et al. Laparoscopic pancreaticoduodenectomy for periampullary tumors: lessons learned from 500 consecutive patients in a single center. Surgical Endoscopy. 2020;34(3):1343-52.

Soriano	2016	Soriano RM, Pino JCR, Juan CD, et al. Influence of portal vein/superior mesenteric vein resection on morbility, mortality and survival of patients with pancreatic ductal adenocarcinoma in the Balearic Islands. Med Balear. 2016;31(3):25-38.
Soubrane	2014	Soubrane O, Goumard C, Laurent A, et al. Laparoscopic resection of hepatocellular carcinoma: a French survey in 351 patients. HPB (Oxford). 2013;16(4):357-65.
Stewart	2004	Stewart GD, Suilleabhain CBO, Madhavan KK, et al. The extent of resection influences outcome following hepatectomy for colorectal liver metastases. Eur J Surg Oncol. 2004;30(4):370-6.
Stiles	2017	Stiles ZE, Behrman SW, Glazer ES, et al. Predictors and implications of unplanned conversion during minimally invasive hepatectomy: an analysis of the ACS-NSQIP database. Hpb. 2017;19(11):957-65.
Sucandy	2018	Sucandy I, Cheek S, Tsung A, et al. Minimally invasive liver resection for primary and metastatic liver tumors: influence of age on perioperative complications and mortality. Surgical Endoscopy. 2018;32(4):1885-91.
Sucandy	2020	Sucandy I, Wecowski J, Schlosser S, et al. Institutional experience of robotic liver resection: Outcome comparison with NSQIP data. American Surgeon. 2020;86(3).
Suda	2015	Suda K, Man IM, Ishida Y, et al. Potential advantages of robotic radical gastrectomy for gastric adenocarcinoma in comparison with conventional laparoscopic approach: a single institutional retrospective comparative cohort study. Surg Endosc. 2014;29(3):673-85.
Sulpice	2015	Sulpice L, Farges O, Goutte N, et al. Laparoscopic Distal Pancreatectomy for Pancreatic Ductal Adenocarcinoma: Time for a Randomized Controlled Trial? Results of an All-inclusive National Observational Study. Ann Surg. 2015;262(5):868-73; discussion 73-4.
Tahkola	2020	Tahkola K, Vayrynen V, Kellokumpu I, et al. Critical evaluation of quality of hepatopancreatic surgery in a medium-volume center in Finland using the accordion severity grading system and the postoperative morbidity index. Journal of Gastrointestinal Oncology. 2020;11(4):724-37.
Thereaux	2018	Thereaux J, Lesuffleur T, Czernichow S, et al. To What Extent Does Posthospital Discharge Chemoprophylaxis Prevent Venous Thromboembolism after Bariatric Surgery? Annals of Surgery. 2018;267(4):727-33.
Tien	2005	Tien YW, Lee PH, Yang CY, et al. Risk factors of massive bleeding related to pancreatic leak after pancreaticoduodenectomy. J Am Coll Surg. 2005;201(4):554-9.

Tien	2008	Tien YW, Wu YM, Liu KL, et al. Angiography is indicated for every sentinel bleed after pancreaticoduodenectomy. Ann Surg Oncol. 2008;15(7):1855-61.
Tieu	2013	Tieu K, Allison N, Snyder B, et al. Robotic-assisted Roux-en-Y gastric bypass: update from 2 high-volume centers. Surg. 2013;9(2):284-8.
Triantafyllidis	2020	Triantafyllidis I, Gayet B, Tsiakyroudi S, et al. Perioperative and long-term outcomes of laparoscopic liver resections for non-colorectal liver metastases. Surgical Endoscopy. 2020;34(9):3833-44.
Turrini	2005	Turrini O, Moutardier V, Guiramand J, et al. Hemorrhage after duodenopancreatectomy: impact of neoadjuvant radiochemotherapy and experience with sentinel bleeding. World J Surg. 2005;29(2):212-6.
Tzeng	2012	Tzeng CW, Katz MH, Fleming JB, et al. Risk of venous thromboembolism outweighs post-hepatectomy bleeding complications: analysis of 5651 National Surgical Quality Improvement Program patients. HPB (Oxford). 2012;14(8):506-13.
Vibert	2006	Vibert E, Perniceni T, Levard H, et al. Laparoscopic liver resection. Br J Surg. 2005;93(1):67-72.
Villagran	2016	Villagran R, Smith G, Rodriguez W, et al. Portomesenteric Vein Thrombosis After Laparoscopic Sleeve Gastrectomy: Incidence, Analysis and Follow-Up in 1236 Consecutive Cases. Obesity Surgery. 2016;26(11):2555-61.
Vining	2020	Vining CC, Kuchta K, Schuitevoerder D, et al. Risk factors for complications in patients undergoing pancreaticoduodenectomy: A NSQIP analysis with propensity score matching. Journal of Surgical Oncology. 2020;122(2):183-94.
Wang	2017	Wang JB, Zheng CH, Li P, et al. Effect of comorbidities on postoperative complications in patients with gastric cancer after laparoscopy-assisted total gastrectomy: results from an 8-year experience at a large-scale single center. Surgical Endoscopy. 2017;31(6):2651-60.
Wang	2020	Wang X, Cai Y, Jiang J, et al. Laparoscopic Pancreaticoduodenectomy: Outcomes and Experience of 550 Patients in a Single Institution. Annals of Surgical Oncology. 2020;27(1):4562-73.
Wei	2009	Wei HK, Wang SE, Shyr YM, et al. Risk factors for post-pancreaticoduodenectomy bleeding and finding an innovative approach to treatment. Dig Surg. 2009;26(4):297-305.

Weller	2008	Weller WE, Rosati C. Comparing outcomes of laparoscopic versus open bariatric surgery. Annals of Surgery. 2008;248(1):10-5.
Woo	2013	Woo HD, Kim YJ. Prevention of venous thromboembolism with enoxaparin in bariatirc surgery. J Korean Surg Soc. 2013;84(5):298-303.
Xu	2019	Xu Y, Hua J, Li J, et al. Long-term outcomes of laparoscopic versus open gastrectomy for advanced gastric cancer: A large cohort study. American Journal of Surgery. 2019;217(4):750-6.
Yekebas	2007	Yekebas EF, Wolfram L, Cataldegirmen G, et al. Postpancreatectomy hemorrhage: diagnosis and treatment: an analysis in 1669 consecutive pancreatic resections. Ann Surg. 2007;246(2):269-80.
Yokoo	2016	Yokoo H, Miyata H, Konno H, et al. Models predicting the risks of six life-threatening morbidities and bile leakage in 14,970 hepatectomy patients registered in the National Clinical Database of Japan. Medicine (United States). 2016;95(49):e5466.
Yu	2006	Yu SC, Clapp BL, Lee MJ, et al. Robotic assistance provides excellent outcomes during the learning curve for laparoscopic Roux-en-Y gastric bypass: results from 100 robotic-assisted gastric bypasses. American Journal of Surgery. 2006;192(6):746-9.
Yu	2018	Yu J, Seo H, Kim HK, et al. Risk Factors for Pulmonary Complications After Laparoscopic Pylorus-preserving Pancreaticoduodenectomy: A Retrospective Observational Analysis. Surg Laparosc Endosc Pct Tech. 2018;28(2):128-32.
Zettervall	2020	Zettervall SL, Ju T, Holzmacher JL, et al. Arterial, but Not Venous, Reconstruction Increases 30-Day Morbidity and Mortality in Pancreaticoduodenectomy. Journal of Gastrointestinal Surgery. 2020;24(3):578-84.
Zhou	2007	Zhou L, Rui JA, Wang SB, et al. Outcomes and prognostic factors of cirrhotic patients with hepatocellular carcinoma after radical major hepatectomy. World J Surg. 2007;31(9):1782-7.
Zureikat	2013	Zureikat AH, Moser AJ, Boone BA, et al. 250 robotic pancreatic resections safety and feasibility. Annals of Surgery. 2013;258(4):554-9.

11. Acknowledgements of authors of original articles

Below is a list of authors, or colleagues of authors, that responded to our requests for data to guide eligibility decisions, confirm the accuracy of data, and gather additional data for our review.

Eppu Ainoa
Bodil Andersson
Osamu Aramaki
Andrew Barbas
Katherine A. Bews
Martin Björck
Ki Byung Song
Diego Cuccurullo
James Garden
Elizabeth Habermann
Mustafa Hasbahceci
Hajime Imamura
Janis Jeffrey
Casey Kraft
Harish Lavu
Fredrik Lindberg
Peter A. Lodge
Nicholas P. McKenna
Emmanuel Melloul
Kyosuke Miyazaki
Benjamin Müssle
Didier Mutter
Yves Panis
Saseem Poudel
Asnat Raziel
Alex Rosemurgy
Jaime Ruiz Tovar
Ville Sallinen
Gabriel Sandblom
Susumu Shibasaki
Koichi Suda
Ching-Wei D. Tzeng
Bob Van Hee
Wendy Weller
Li Zhou
Leiming Zhu
