# Supplementary material for: Systematic Reviews and Meta-analyses of the Procedure-specific Risks of Thrombosis and Bleeding in General Abdominal, Colorectal, Upper Gastrointestinal, and Hepatopancreatobiliary Surgery
Source: Ann Surg. 2023 Aug 8;279(2):213–25. doi: 10.1097/SLA.0000000000006059 (PMC10782937; doi:10.1097/SLA.0000000000006059)
Supplement: SUPPLEMENTARY MATERIAL [file sla-279-213-s002.docx]

**Supplementary legends**

**Supplementary table 1.** The 4-week postoperative incidence of symptomatic venous thromboembolism and bleeding requiring reintervention after appendectomy and cholecystectomy

**Supplementary table 2.** The 4-week postoperative risk of symptomatic venous thromboembolism and bleeding requiring reintervention after hernia repair

**Supplementary table 3.** The 4-week postoperative risk of symptomatic venous thromboembolism and bleeding requiring reintervention after small bowel resection and splenectomy

**Supplementary table 4.** The 4-week postoperative risk of symptomatic venous thromboembolism and bleeding requiring reintervention after abdominoperineal resection, anterior resection and rectopexy

**Supplementary table 5.** The 4-week postoperative risk of symptomatic venous thromboembolism and bleeding requiring reintervention after colectomy

**Supplementary table 6.** The 4-week postoperative risk of symptomatic venous thromboembolism and bleeding requiring reintervention after total proctocolectomy/total colectomy

**Supplementary table 7.** The 4-week postoperative risk of symptomatic venous thromboembolism and bleeding requiring reintervention after pancreatic surgery

**Supplementary table 8.** The 4-week postoperative risk of symptomatic venous thromboembolism and bleeding requiring reintervention after liver surgery

**Supplementary table 9.** The 4-week postoperative risk of symptomatic venous thromboembolism and bleeding requiring reintervention after upper gastrointestinal surgery

**Supplementary table 1.** The 4-week postoperative incidence of symptomatic venous thromboembolism and bleeding requiring reintervention after appendectomy and cholecystectomy

| **Procedure** | **Outcome** | **Patients (studies)** | **Estimate** | **Patient VTE risk strata** | **Evidence certainty** |
| --- | --- | --- | --- | --- | --- |
|  |  |  |  |  |  |
|  |  |  | **Median (%)** | **Low - Medium – High (%)** |  |
| Appendectomy, laparoscopic | Venous thromboembolism | 352,842 (6) | 0.2 | 0.1 - 0.3 - 0.5 | Moderate |
|  | Bleeding requiring reintervention | 10,959 (9) | 0.1 |  | Low |
| Appendectomy, open | Venous thromboembolism | 238,094 (4) | 0.4 | 0.4 - 0.7 - 1.5 | Low |
|  | Bleeding requiring reintervention | 5222 (3) | 0.1 |  | Low |
|  |  |  |  |  |  |
| Cholecystectomy, laparoscopic | Venous thromboembolism | 4,698,705 (17) | <0.1 | <0.1 - 0.1 - 0.1 | Moderate |
|  | Bleeding requiring reintervention | 10,959 (9) | 0.3 |  | Low |
| Cholecystectomy, laparoscopic, elective | Venous thromboembolism | 2,450 (5) | <0.1 | <0.1 - 0.1 - 0.1 | High |
|  | Bleeding requiring reintervention | 1,739 (3) | 0.1 |  | Moderate |
| Cholecystectomy, laparoscopic, emergency | Venous thromboembolism | 11,266 (1) | 0.3 | 0.3 - 0.5 - 1.1 | Moderate |
|  | Bleeding requiring reintervention | 10,959 (9) | 0.4 |  | Very low |
| Cholecystectomy, open | Venous thromboembolism | 64,493 (5) | 1.3 | 0.9 - 1.8 - 3.7 | Moderate |
|  | Bleeding requiring reintervention | 10,959 (9) | 0.4 |  | Very low |
| In the Estimate column, we present median estimates for venous thromboembolism (VTE) and bleeding requiring reintervention by procedure. In the Patient VTE risk strata column, we present VTE estimates by patient VTE risk strata. In the VTE risk strata, patients with no VTE risk factor are classified as low VTE risk, patients with one VTE risk factor (age 75 or more; body mass index of 35 or more, or history of VTE in parents, full siblings, or children) as medium VTE risk, and patients with two risk factors and those with personal history of VTE as high VTE risk. For more details, see the Supplemental Digital Content Appendix, pages 7-16. | | | | | |

**Supplementary table 2.** The 4-week postoperative risk of symptomatic venous thromboembolism and bleeding requiring reintervention after hernia repair

| **Procedure** | **Outcome** | **Patients (studies)** | **Estimate** | **Patient VTE risk strata** | **Evidence certainty** |  |
| --- | --- | --- | --- | --- | --- | --- |
|  |  |  |  |  |  |  |
|  |  |  | **Median (%)** | **Low - Medium – High (%)** |  |  |
| Groin hernia repair, laparoscopic | Venous thromboembolism | 13,333 (6) | 0.6 | 0.4 - 0.8 - 1.5 | Low |  |
|  | Bleeding requiring reintervention | 5,086 (3) | 0.2 |  | Low |  |
| Groin hernia repair, laparoscopic, elective | Venous thromboembolism | 226 (2) | <0.1 | <0.1 - <0.1 - <0.1 | Low |  |
|  | Bleeding requiring reintervention | 4,978 (2) | 0.1 |  | Low |  |
| Groin hernia repair, open | Venous thromboembolism | 189,943 (9) | 0.2 | 0.1 - 0.3 - 0.5 | Low |  |
|  | Bleeding requiring reintervention | 5,222 (3) | 0.1 |  | Moderate |  |
| Groin hernia repair, open, elective | Venous thromboembolism | 133,019 (2) | 0.1 | <0.1 - 0.1 - 0.2 | Moderate |  |
|  | Bleeding requiring reintervention | 352 (2) | 0.3 |  | Low |  |
| Groin hernia repair, open, emergency | Venous thromboembolism | 8,403 (4) | 1.4 | 0.8 - 1.6 - 3.2 | Moderate |  |
|  | Bleeding requiring reintervention | 146 (1) | <0.1 |  | Very low |  |
|  |  |  |  |  |  |  |
| Ventral hernia repair, laparoscopic* | Venous thromboembolism | 35,364 (5) | 0.4 | 0.3 - 0.6 - 1.2 | Low |  |
|  | Bleeding requiring reintervention | 517 (2) | 0.1 |  | Very low |  |
| Ventral hernia repair, laparoscopic, elective | Venous thromboembolism | 26,778 (4) | 0.2 | 0.2 - 0.3 - 0.7 | Low |  |
|  | Bleeding requiring reintervention | 361 (1) | 0.2 |  | Very low |  |
| Ventral hernia repair, laparoscopic, emergency | Venous thromboembolism | 405 (1) | 1.2 | 0.9 - 1.9 - 3.8 | Low |  |
|  | Bleeding requiring reintervention | 0 (0) | NR |  |  |  |
| Ventral hernia repair, open | Venous thromboembolism | 133,803 (6) | 1.3 | 0.9 - 1.9 - 3.8 | Low |  |
|  | Bleeding requiring reintervention | 618 (4) | 1.0 |  | Low |  |
| Ventral hernia repair, open, elective | Venous thromboembolism | 91,203 (5) | 0.9 | 0.7 - 1.4 - 2.8 | Low |  |
|  | Bleeding requiring reintervention | 301 (2) | 0.6 |  | Low |  |
| Ventral hernia repair, open, emergency | Venous thromboembolism | 4,808 (1) | 1.6 | 1.2 - 2.5 – 5.0 | Moderate |  |
|  | Bleeding requiring reintervention | 0 (0) | NR |  |  |  |
| *Bleeding requiring reintervention estimate included 53 (10%) robotic ventral hernia repair patients; venous thromboembolism (VTE) estimate included only laparoscopic ventral hernia repair patients. NR = Not reported. In the Estimate column, we present median estimates for VTE and bleeding requiring reintervention by procedure. In the Patient VTE risk strata column, we present VTE estimates by patient VTE risk strata. In the VTE risk strata, patients with no VTE risk factor are classified as low VTE risk, patients with one VTE risk factor (age 75 or more; body mass index of 35 or more, or history of VTE in parents, full siblings, or children) as medium VTE risk, and patients with two risk factors and those with personal history of VTE as high VTE risk. For more details, see the Supplemental Digital Content Appendix, pages 17-29. | | | | | |  |

**Supplementary table 3.** The 4-week postoperative risk of symptomatic venous thromboembolism and bleeding requiring reintervention after small bowel resection and splenectomy

| **Procedure** | **Outcome** | **Patients (studies)** | **Estimate** | **Patient VTE risk strata** | **Evidence certainty** |
| --- | --- | --- | --- | --- | --- |
|  |  |  |  |  |  |
|  |  |  | **Median (%)** | **Low - Medium – High (%)** |  |
| Small bowel resection, laparoscopic | Venous thromboembolism | 3,195 (2) | 1.6 | 1.1 - 2.3 - 4.6 | Moderate |
| Small bowel resection, open | Venous thromboembolism | 28,148 (3) | 3.7 | 2.7 - 5.3 - 10.6 | Moderate |
| *By indication* |  |  |  |  |  |
| Small bowel resection, laparoscopic, benign | Venous thromboembolism | 355 (1) | 1.1 | 0.8 - 1.5 – 3.0 | Low |
| Small bowel resection, laparoscopic, malignant | Venous thromboembolism | 499 (1) | 2.3 | 1.4 - 2.9 - 5.8 | Low |
| Small bowel resection, laparoscopic, IBD | Venous thromboembolism | 443 (1) | 1.1 | 1.0 - 1.9 - 3.9 | Low |
| Small bowel resection, open, benign | Venous thromboembolism | 571 (1) | 0.9 | 0.5 - 1.1 - 2.2 | Low |
| Small bowel resection, open, malignant | Venous thromboembolism | 1,784 (1) | 3.4 | 2.3 - 4.6 - 9.2 | Moderate |
| Small bowel resection, open, IBD | Venous thromboembolism | 1,237 (1) | 2.0 | 1.7 - 3.4 - 6.8 | Moderate |
| Small bowel resection, open, emergency | Venous thromboembolism | 6,855 (1) | 3.7 | 2.9 - 5.8 - 11.5 | Moderate |
|  |  |  |  |  |  |
| Splenectomy, laparoscopic, elective | Venous thromboembolism | 5,177 (5) | 2.9 | 2.4 - 4.8 - 9.5 | Moderate |
|  | Bleeding requiring reintervention | 2,203 (8) | 1.3 |  | Low |
| Splenectomy, open, elective | Venous thromboembolism | 2,590 (3) | 1.8 | 1.4 - 2.8 - 5.7 | Low |
|  | Bleeding requiring reintervention | 385 (2) | 4.0 |  | Very low |
| IBD = Inflammatory bowel disease. We did not find bleeding requiring reintervention estimates for small bowel resections. In the Estimate column, we present median estimates for venous thromboembolism (VTE) and bleeding requiring reintervention by procedure. In the Patient VTE risk strata column, we present VTE estimates by patient VTE risk strata. In the VTE risk strata, patients with no VTE risk factor are classified as low VTE risk, patients with one VTE risk factor (age 75 or more; body mass index of 35 or more, or history of VTE in parents, full siblings, or children) as medium VTE risk, and patients with two risk factors and those with personal history of VTE as high VTE risk. For more details, see the Supplemental Digital Content Appendix, pages 30-46. | | | | | |

**Supplementary table 4.** The 4-week postoperative risk of symptomatic venous thromboembolism and bleeding requiring reintervention after abdominoperineal resection, anterior resection and rectopexy

| **Procedure** | **Outcome** | **Patients (studies)** | **Estimate** | **Patient VTE risk strata** | **Evidence certainty** |
| --- | --- | --- | --- | --- | --- |
|  |  |  |  |  |  |
|  |  |  | **Median (%)** | **Low - Medium - High (%)** |  |
| Abdominoperineal resection, laparoscopic | Venous thromboembolism | 2,574 (1) | 1.3 | 0.9 - 1.8 - 3.6 | Moderate |
|  | Bleeding requiring reintervention | 0 (0) | NR |  |  |
| Abdominoperineal resection, open | Venous thromboembolism | 5,107 (1) | 3.6 | 2.4 - 4.9 - 9.8 | Moderate |
|  | Bleeding requiring reintervention | 0 (0) | NR |  |  |
| Anterior resection, minimally-invasive* | Venous thromboembolism | 35,110 (6) | 1.2 | 0.8 - 1.6 - 3.2 | Low |
|  | Bleeding requiring reintervention | 811 (4) | 1.6 |  | Very low |
| Anterior resection, open* | Venous thromboembolism | 93,593 (4) | 1.5 | 1.0 - 2.0 - 4.0 | Low |
|  | Bleeding requiring reintervention | 0 (0) | NR |  |  |
| Rectopexy, laparoscopic | Venous thromboembolism | 3,350 (1) | 0.4 | 0.3 - 0.5 - 1.0 | Moderate |
|  | Bleeding requiring reintervention | 0 (0) | NR |  |  |
| Rectopexy, open | Venous thromboembolism | 3,599 (1) | 0.6 | 0.4 - 0.8 - 1.6 | Moderate |
|  | Bleeding requiring reintervention | 0 (0) | NR |  |  |
| Rectopexy, perineal | Venous thromboembolism | 5,384 (2) | 1.2 | 0.6 - 1.1 - 2.3 | Moderate |
|  | Bleeding requiring reintervention | 156 (2) | 0.4 |  | Very low |
| IBD = Inflammatory bowel disease, NR = Not reported. *Including partial proctocolectomy. In the Estimate column, we present median estimates for venous thromboembolism (VTE) and bleeding requiring reintervention by procedure. In the Patient VTE risk strata column, we present VTE estimates by patient VTE risk strata. In the VTE risk strata, patients with no VTE risk factor are classified as low VTE risk, patients with one VTE risk factor (age 75 or more; body mass index of 35 or more, or history of VTE in parents, full siblings, or children) as medium VTE risk, and patients with two risk factors and those with personal history of VTE as high VTE risk. For more details, see the Supplemental Digital Content Appendix, pages 48-53 and 79-81. | | | | | |

**Supplementary table 5.** The 4-week postoperative risk of symptomatic venous thromboembolism and bleeding requiring reintervention after colectomy

| **Procedure** | **Outcome** | **Patients (studies)** | **Estimate** | **Patient VTE risk strata** | **Evidence certainty** |
| --- | --- | --- | --- | --- | --- |
|  |  |  |  |  |  |
|  |  |  | **Median (%)** | **Low - Medium - High (%)** |  |
| Colectomy, minimally-invasive | Venous thromboembolism | 189,169 (22) | 1.7 | 1.2 - 2.4 - 4.7 | Low |
|  | Bleeding requiring reintervention | 3,004 (7) | 1.0 |  | Moderate |
| Colectomy, laparoscopic | Venous thromboembolism | 187,330 (20) | 1.6 | 1.1 - 2.2 - 4.5 | Low |
|  | Bleeding requiring reintervention | 3,004 (7) | 1.0 |  | Moderate |
| Colectomy, robotic | Venous thromboembolism | 1,010 (4) | 1.7 | 1.2 - 2.4 - 4.8 | Moderate |
|  | Bleeding requiring reintervention | 0 (0) | NR |  |  |
| Colectomy, open | Venous thromboembolism | 288,439 (13) | 4.4 | 3.1 - 6.2 - 12.4 | Low |
|  | Bleeding requiring reintervention | 105,013 (4) | 0.8 |  | Very low |
| *Colectomy by indication* |  |  |  |  |  |
| Colectomy, minimally-invasive, benign | Venous thromboembolism | 52,945 (5) | 0.4 | 0.3 - 0.6 - 1.2 | Moderate |
|  | Bleeding requiring reintervention | 1,016 (1) | 0.1 |  | Moderate |
| Colectomy, minimally-invasive, malign | Venous thromboembolism | 53,523 (4) | 1.8 | 1.1 - 2.2 - 4.4 | Moderate |
|  | Bleeding requiring reintervention | 470 (2) | 1.3 |  | Low |
| Colectomy, minimally-invasive, IBD | Venous thromboembolism | 8,955 (4) | 2.1 | 1.8 - 3.6 - 7.3 | Low |
|  | Bleeding requiring reintervention | 204 (1) | 0.3 |  | Very low |
| Colectomy, minimally-invasive, emergency | Venous thromboembolism | 2,341 (2) | 4.8 | 3.3 - 6.7 - 13.4 | Moderate |
|  | Bleeding requiring reintervention | 0 (0) | NR |  |  |
| Colectomy, open, benign | Venous thromboembolism | 151,187 (5) | 2.3 | 1.5 - 3.1 - 6.2 | Very low |
|  | Bleeding requiring reintervention | 0 (0) | NR |  |  |
| Colectomy, open, malignant | Venous thromboembolism | 82,643 (4) | 3.4 | 2.0 - 3.9 - 7.8 | Low |
|  | Bleeding requiring reintervention | 0 (0) | NR |  |  |
| Colectomy, open, IBD | Venous thromboembolism | 8,128 (2) | 4.1 | 3.5 - 6.9 - 13.8 | Moderate |
|  | Bleeding requiring reintervention | 0 (0) | NR |  |  |
| Colectomy, open, emergency | Venous thromboembolism | 29,874 (3) | 6.8 | 4.6 - 9.1 - 18.3 | Moderate |
|  | Bleeding requiring reintervention | 0 (0) | NR |  |  |
| *Colectomy by extent* |  |  |  |  |  |
| Colectomy, minimally-invasive, left | Venous thromboembolism | 48,496 (3) | 1.9 | 1.3 - 2.5 - 5.1 | Moderate |
|  | Bleeding requiring reintervention | 696 (2) | 1.1 |  | Very low |
| Colectomy, minimally-invasive, right | Venous thromboembolism | 20,271 (3) | 1.4 | 0.9 - 1.9 - 3.8 | Moderate |
|  | Bleeding requiring reintervention | 340 (2) | 1.5 |  | Very low |
| Colectomy, minimally-invasive, sigmoid | Venous thromboembolism | 5,325 (4) | 0.3 | 0.2 - 0.4 - 0.9 | Low |
|  | Bleeding requiring reintervention | 1,016 (1) | 0.1 |  | Moderate |
| Colectomy, open, left | Venous thromboembolism | 22,603 (2) | 4.0 | 2.6 - 5.2 - 10.4 | Moderate |
|  | Bleeding requiring reintervention | 0 (0) | NR |  |  |
| Colectomy, open, right | Venous thromboembolism | 20,650 (2) | 3.4 | 2.2 - 4.3 - 8.6 | Moderate |
|  | Bleeding requiring reintervention | 0 (0) | NR |  |  |
| Colectomy, open, sigmoid | Venous thromboembolism | 25,691 (3) | 1.9 | 1.3 - 2.6 - 5.2 | Very low |
|  | Bleeding requiring reintervention | 0 (0) | NR |  |  |
| IBD = Inflammatory bowel disease, NR = Not reported. Minimally-invasive includes laparoscopic and robotic procedures. Colectomy includes studies that included patients undergoing colectomy procedures other than total colectomy. In the Estimate column, we present median estimates for venous thromboembolism (VTE) and bleeding requiring reintervention by procedure. In the Patient VTE risk strata column, we present VTE estimates by patient VTE risk strata. In the VTE risk strata, patients with no VTE risk factor are classified as low VTE risk, patients with one VTE risk factor (age 75 or more; body mass index of 35 or more, or history of VTE in parents, full siblings, or children) as medium VTE risk, and patients with two risk factors and those with personal history of VTE as high VTE risk. For more details, see the Supplemental Digital Content Appendix, pages 54-69. | | | | | |

**Supplementary table 6.** The 4-week postoperative risk of symptomatic venous thromboembolism and bleeding requiring reintervention after total proctocolectomy/total colectomy

| **Procedure** | **Outcome** | **Patients (studies)** | **Estimate** | **Patient VTE risk strata** | **Evidence certainty** |
| --- | --- | --- | --- | --- | --- |
|  |  |  |  |  |  |
|  |  |  | **Median (%)** | **Low - Medium - High (%)** |  |
| Total proctocolectomy, laparoscopic | Venous thromboembolism | 6,079 (3) | 5.0 | 4.3 - 8.6 - 17.3 | Low |
|  | Bleeding requiring reintervention | 204 (1) | 0.3 |  | Very low |
| Total proctocolectomy, open | Venous thromboembolism | 8,252 (2) | 5.4 | 4.5 - 9.0 - 18.0 | Moderate |
|  | Bleeding requiring reintervention | 0 (0) | NR |  |  |
| *Total proctocolectomy by indication* |  |  |  |  |  |
| Total proctocolectomy, laparoscopic, benign | Venous thromboembolism | 238 (1) | 5.0 | 4.2 - 8.5 - 17.0 | Low |
|  | Bleeding requiring reintervention | 0 (0) | NR |  |  |
| Total proctocolectomy, laparoscopic, malignant | Venous thromboembolism | 1,307 (1) | 3.4 | 2.4 - 4.7 - 9.5 | Moderate |
|  | Bleeding requiring reintervention | 0 (0) | NR |  |  |
| Total proctocolectomy, laparoscopic, IBD | Venous thromboembolism | 4,055 (1) | 5.3 | 4.5 - 9.0 - 18.0 | Moderate |
|  | Bleeding requiring reintervention | 0 (0) | NR |  |  |
| Total proctocolectomy, open, benign | Venous thromboembolism | 708 (1) | 6.0 | 3.7 - 7.5 - 14.9 | Low |
|  | Bleeding requiring reintervention | 0 (0) | NR |  |  |
| Total proctocolectomy, open, malignant | Venous thromboembolism | 2,410 (1) | 4.7 | 3.1 - 6.2 - 12.5 | Moderate |
|  | Bleeding requiring reintervention | 0 (0) | NR |  |  |
| Total proctocolectomy, open, IBD | Venous thromboembolism | 3,202 (2) | 4.8 | 4.0 - 8.0 - 16.0 | Moderate |
|  | Bleeding requiring reintervention | 0 (0) | NR |  |  |
| Total proctocolectomy, open, emergency | Venous thromboembolism | 1,932 (1) | 10.0 | 5.9 - 11.7 - 23.5 | Moderate |
|  | Bleeding requiring reintervention | 0 (0) | NR |  |  |
| IBD = Inflammatory bowel disease, NR = Not reported. Estimates include both total proctocolectomy and total colectomy. In the Estimate column, we present median estimates for venous thromboembolism (VTE) and bleeding requiring reintervention by procedure. In the Patient VTE risk strata column, we present VTE estimates by patient VTE risk strata. In the VTE risk strata, patients with no VTE risk factor are classified as low VTE risk, patients with one VTE risk factor (age 75 or more; body mass index of 35 or more, or history of VTE in parents, full siblings, or children) as medium VTE risk, and patients with two risk factors and those with personal history of VTE as high VTE risk. For more details, see the Supplemental Digital Content Appendix, pages 70-78. | | | | | |

**Supplementary table 7.** The 4-week postoperative risk of symptomatic venous thromboembolism and bleeding requiring reintervention after pancreatic surgery

| **Procedure** | **Outcome** | **Patients (studies)** | **Estimate** | **Patient VTE risk strata** | **Certainty in estimate** |
| --- | --- | --- | --- | --- | --- |
|  |  |  |  |  |  |
|  |  |  | **Median (%)** | **Low - Medium - High (%)** |  |
| Distal pancreatectomy, minimally-invasive | Venous thromboembolism | 1,858 (2) | 2.5 | 1.8 - 3.6 - 7.3 | Moderate |
|  | Bleeding requiring reintervention | 1,137 (4) | 0.9 |  | Low |
| Distal pancreatectomy, laparoscopic, benign | Venous thromboembolism | 1,030 (1) | 2.2 | 1.6 - 3.3 - 6.6 | Moderate |
|  | Bleeding requiring reintervention | 0 (0) | NR |  |  |
| Distal pancreatectomy, laparoscopic, malignant | Venous thromboembolism | 759 (1) | 3.4 | 2.3 - 4.7 - 9.3 | Low |
|  | Bleeding requiring reintervention | 0 (0) | NR |  |  |
| Distal pancreatectomy, open | Venous thromboembolism | 2,106 (4) | 6.4 | 4.2 - 8.3 - 16.7 | Low |
|  | Bleeding requiring reintervention | 1,485 (4) | 0.7 |  | Very low |
| Distal pancreatectomy, open, benign | Venous thromboembolism | 655 (1) | 2.3 | 1.6 - 3.2 - 6.3 | Low |
|  | Bleeding requiring reintervention | 0 (0) | NR |  |  |
| Distal pancreatectomy, open, malignant | Venous thromboembolism | 1,260 (3) | 6.8 | 4.5 - 9 - 17.9 | Low |
|  | Bleeding requiring reintervention | 0 (0) | NR |  |  |
| Pancreaticoduodenectomy, minimally-invasive | Venous thromboembolism | 1,872 (5) | 5.3 | 3.7 - 7.4 - 14.8 | Low |
|  | Bleeding requiring reintervention | 896 (5) | 1.8 |  | Very low |
| Pancreaticoduodenectomy, open | Venous thromboembolism | 34,004 (12) | 6.2 | 4.1 - 8.3 - 16.6 | Low |
|  | Bleeding requiring reintervention | 2,472 (10) | 2.7 |  | Low |
| Minimally-invasive includes laparoscopic and robotic surgeries. In the Estimate column, we present median estimates for venous thromboembolism (VTE) and bleeding requiring reintervention by procedure. In the Patient VTE risk strata column, we present VTE estimates by patient VTE risk strata. In the VTE risk strata, patients with no VTE risk factor are classified as low VTE risk, patients with one VTE risk factor (age 75 or more; body mass index of 35 or more, or history of VTE in parents, full siblings, or children) are classified as medium VTE risk, and patients with two risk factors and those with personal history of VTE are classified as high VTE risk. For more details, see the Supplemental Digital Content Appendix, pages 83-106. | | | | | |

**Supplementary table 8.** The 4-week postoperative risk of symptomatic venous thromboembolism and bleeding requiring reintervention after liver surgery

| **Procedure** | **Outcome** | **Patients (studies)** | **Estimate** | **Patient VTE risk strata** | **Certainty in estimate** |
| --- | --- | --- | --- | --- | --- |
|  |  |  |  |  |  |
|  |  |  | **Median (%)** | **Low - Medium - High (%)** |  |
| Liver resection, minimally-invasive | Venous thromboembolism | 3,270 (8) | 0.8 | 0.6 - 1.1 - 2.2 | Low |
|  | Bleeding requiring reintervention | 617 (6) | 0.8 |  | Very low |
| Liver resection, minimally-invasive, minor | Venous thromboembolism | 937 (2) | 0.8 | 0.5 - 1.1 - 2.1 | Low |
|  | Bleeding requiring reintervention | 0 (0) | NR |  |  |
| Liver resection, minimally-invasive, major | Venous thromboembolism | 226 (1) | 4.4 | 3.0 - 5.9 - 11.8 | Very low |
|  | Bleeding requiring reintervention | 0 (0) | NR |  |  |
| Liver resection, open | Venous thromboembolism | 29,872 (15) | 2.6 | 1.9 - 3.8 - 7.5 | Low |
|  | Bleeding requiring reintervention | 8,649 (9) | 1.1 |  | Low |
| Liver resection, open, minor | Venous thromboembolism | 4,165 (3) | 3.5 | 2.4 - 4.8 - 9.6 | Low |
|  | Bleeding requiring reintervention | 4,165 (3) | 0.5 |  | Moderate |
| Liver resection, open, major | Venous thromboembolism | 3,943 (8) | 5.3 | 3.9 - 7.8 - 15.5 | Low |
|  | Bleeding requiring reintervention | 2,233 (5) | 0.9 |  | Low |
| Minimally-invasive includes laparoscopic and robotic surgeries. We accepted the definition of major liver resection used in each study (in 7 out of 10 studies defined as resection of 3 or more segments). In the Estimate column, we present median estimates for venous thromboembolism (VTE) and bleeding requiring reintervention by procedure. In the Patient VTE risk strata column, we present VTE estimates by patient VTE risk strata. In the VTE risk strata, patients with no VTE risk factor are classified as low VTE risk, patients with one VTE risk factor (age 75 or more; body mass index of 35 or more, or history of VTE in parents, full siblings, or children) are classified as medium VTE risk, and patients with two risk factors and those with personal history of VTE are classified as high VTE risk. For more details, see the Supplemental Digital Content Appendix, pages 91-98. | | | | | |

**Supplementary table 9.** The 4-week postoperative risk of symptomatic venous thromboembolism and bleeding requiring reintervention after upper gastrointestinal surgery

| **Procedure** | **Outcome** | **Patients (studies)** | **Estimate** | **Patient VTE risk strata** | **Certainty in estimate** |
| --- | --- | --- | --- | --- | --- |
|  |  |  |  |  |  |
|  |  |  | **Median (%)** | **Low - Medium – High (%)** |  |
| Gastrectomy, minimally-invasive | Venous thromboembolism | 1,553 (3) | 2.6 | 1.8 - 3.5 – 7.0 | Very low |
|  | Bleeding requiring reintervention | 61 (1) | 1.1 |  | Very low |
| Gastrectomy, open | Venous thromboembolism | 22,679 (5) | 3.3 | 1.9 - 3.7 - 7.5 | Very low |
|  | Bleeding requiring reintervention | 490 (2) | 0.4 |  | Low |
| Subtotal gastrectomy, open | Venous thromboembolism | 1,581 (1) | 2.5 | 1.4 - 2.9 - 5.7 | Moderate |
|  | Bleeding requiring reintervention | 310 (1) | 0.4 |  | Low |
| Total gastrectomy, open | Venous thromboembolism | 999 (1) | 4.5 | 3.0 – 6.0 - 11.9 | Low |
|  | Bleeding requiring reintervention | 0 (0) | NR |  |  |
| Gastric bypass, minimally-invasive | Venous thromboembolism | 286,668 (8) | 0.6 | NA - 0.5 – 1.0 | Moderate |
|  | Bleeding requiring reintervention | 119,535 (6) | 0.3 |  | Low |
| Gastric bypass, laparoscopic | Venous thromboembolism | 280,751 (7) | 0.5 | NA - 0.4 - 0.9 | Moderate |
|  | Bleeding requiring reintervention | 119,435 (6) | 0.3 |  | Low |
| Gastric bypass, robotic | Venous thromboembolism | 7,453 (7) | 1.5 | NA - 1.3 - 2.5 | Low |
|  | Bleeding requiring reintervention | 436 (4) | 0.3 |  | Very low |
| Gastric bypass, open | Venous thromboembolism | 68,017 (18) | 1.4 | NA - 1.1 - 2.3 | Very low |
|  | Bleeding requiring reintervention | 3,256 (4) | 0.2 |  | Moderate |
| Sleeve gastrectomy, minimally-invasive | Venous thromboembolism | 470,221 (14) | 0.3 | NA - 0.2 - 0.5 | Moderate |
|  | Bleeding requiring reintervention | 316,048 (7) | 0.3 |  | Moderate |
| Sleeve gastrectomy, laparoscopic | Venous thromboembolism | 457,309 (13) | 0.2 | NA - 0.2 - 0.4 | Moderate |
|  | Bleeding requiring reintervention | 316,048 (7) | 0.3 |  | Moderate |
| Sleeve gastrectomy, robotic | Venous thromboembolism | 13,457 (3) | 1.0 | NA - 0.8 - 1.6 | Moderate |
|  | Bleeding requiring reintervention | 545 (2) | 0.5 |  | Low |
| Minimally-invasive includes laparoscopic and robotic surgeries. In the Estimate column, we present median estimates for venous thromboembolism (VTE) and bleeding requiring reintervention by procedure. In the Patient VTE risk strata column, we present VTE estimates by patient VTE risk strata. In the VTE risk strata, patients with no VTE risk factor are classified as low VTE risk, patients with one VTE risk factor (age 75 or more; body mass index of 35 or more, or history of VTE in parents, full siblings, or children) are classified as medium VTE risk, and patients with two risk factors and those with personal history of VTE are classified as high VTE risk. For bariatric surgery, there is no low VTE risk group, as all patients have BMI of 35 or more and are therefore categorized to medium or high VTE risk group. Open and minimally-invasive (subtotal and total) gastrectomy estimates include only studies conducted in non-Asian countries. We found lower VTE risks in studies conducted in Asian countries. For more details, see the Supplemental Digital Content Appendix, pages 107-129. | | | | | |
